# Supplementary material for: Functional characterization of all CDKN2A missense variants and comparison to in silico models of pathogenicity
Source: bioRxiv. 2025 Feb 11:2023.12.28.573507. Originally published 2023 Dec 28. Preprint. [Version 3] doi: 10.1101/2023.12.28.573507 (PMC10793438; doi:10.1101/2023.12.28.573507)
Supplement: Supplement 1 [file media-1.pdf]

Appendix 1-table 1. Assay outputs for CellTag experiments.

CellTag number	Experiment 1				Experiment 2				Experiment 3				Proprotion			
	Read count		Proportion		Read count		Proportion		Read count		Proportion					
	Day 9	Day 45	Day 9	Day 45	Day 9	Day 45	Day 9	Day 45	Day 9	Day 45	Day 9	Day 45	Mean Day 9	Mean Day 45	Standard deviation Day 9	Standard deviation Day 45
CellTag1	18886	7358	4.68	4.40	9135	7518	4.49	4.24	9860	6608	4.38	4.42	4.52	4.35	0.15	0.10
CellTag2	22571	9930	5.60	5.93	11498	10239	5.65	5.78	12373	8001	5.50	5.35	5.58	5.69	0.08	0.30
CellTag3	18731	8318	4.65	4.97	9439	9057	4.64	5.11	11058	7363	4.91	4.93	4.73	5.00	0.16	0.10
CellTag4	25031	10273	6.21	6.14	13022	10865	6.40	6.13	13515	9318	6.00	6.24	6.20	6.17	0.20	0.06
CellTag5	18034	8229	4.47	4.92	9654	8455	4.74	4.77	10665	6944	4.74	4.65	4.65	4.78	0.15	0.13
CellTag6	21130	8923	5.24	5.33	10702	9912	5.26	5.60	12738	8179	5.66	5.47	5.39	5.47	0.24	0.13
CellTag7	17421	8023	4.32	4.79	9086	8475	4.46	4.79	9867	7113	4.38	4.76	4.39	4.78	0.07	0.02
CellTag8	19780	8156	4.91	4.87	9708	8955	4.77	5.06	11174	7404	4.96	4.95	4.88	4.96	0.10	0.09
CellTag9	20872	8931	5.18	5.33	10628	9194	5.22	5.19	11859	7849	5.27	5.25	5.22	5.26	0.05	0.07
CellTag10	19441	7610	4.82	4.55	10308	7461	5.07	4.21	10458	7297	4.65	4.88	4.84	4.55	0.21	0.34
CellTag11	24278	10397	6.02	6.21	12258	11333	6.02	6.40	14086	9108	6.26	6.09	6.10	6.23	0.14	0.15
CellTag12	26268	11069	6.52	6.61	13209	11494	6.49	6.49	14782	9871	6.57	6.61	6.52	6.57	0.04	0.07
CellTag13	18378	7440	4.56	4.44	8926	8197	4.39	4.63	9626	6363	4.28	4.26	4.41	4.44	0.14	0.19
CellTag14	26288	9801	6.52	5.85	12418	11095	6.10	6.26	14185	8737	6.30	5.85	6.31	5.99	0.21	0.24
CellTag15	23881	9917	5.92	5.92	12073	11203	5.93	6.33	13775	8986	6.12	6.01	5.99	6.09	0.11	0.21
CellTag16	15131	6019	3.75	3.60	7421	6208	3.65	3.51	8175	5532	3.63	3.70	3.68	3.60	0.07	0.10
CellTag17	5094	1861	1.26	1.11	2598	1787	1.28	1.01	2788	1607	1.24	1.08	1.26	1.07	0.02	0.05
CellTag18	25447	10259	6.31	6.13	12235	10417	6.01	5.88	13537	9539	6.01	6.38	6.11	6.13	0.17	0.25
CellTag19	25280	10523	6.27	6.29	13419	10627	6.59	6.00	14407	9221	6.40	6.17	6.42	6.15	0.16	0.14
CellTag20	11225	4371	2.78	2.61	5774	4611	2.84	2.60	6170	4396	2.74	2.94	2.79	2.72	0.05	0.19
