# Supplementary material for: Functional characterization of all CDKN2A missense variants and comparison to in silico models of pathogenicity
Source: bioRxiv. 2025 Feb 11:2023.12.28.573507. Originally published 2023 Dec 28. Preprint. [Version 3] doi: 10.1101/2023.12.28.573507 (PMC10793438; doi:10.1101/2023.12.28.573507)
Supplement: Supplement 2 [file media-2.pdf]

Appendix 1-table 2. Proportion of each variant in the initial plasmid library.

Residue	Variant	Respresentation in library (%)
1	p.Met1Asn	5.0
1	p.Met1Lys	6.7
1	p.Met1Thr	5.2
1	p.Met1Arg	4.5
1	p.Met1Ser	4.6
1	p.Met1Ile	6.2
1	p.Met1Met	0.0
1	p.Met1His	4.6
1	p.Met1Gln	4.2
1	p.Met1Pro	4.5
1	p.Met1Leu	10.6
1	p.Met1Asp	4.3
1	p.Met1Glu	4.4
1	p.Met1Ala	4.6
1	p.Met1Gly	4.1
1	p.Met1Val	8.6
1	p.Met1Tyr	4.4
1	p.Met1Cys	4.5
1	p.Met1Trp	4.5
1	p.Met1Phe	4.6
2	p.Glu2Asn	3.3
2	p.Glu2Lys	3.4
2	p.Glu2Thr	2.6
2	p.Glu2Arg	2.8
2	p.Glu2Ser	2.0
2	p.Glu2Ile	3.9
2	p.Glu2Met	2.9
2	p.Glu2His	3.7
2	p.Glu2Gln	4.8
2	p.Glu2Pro	3.7
2	p.Glu2Leu	3.4
2	p.Glu2Asp	26.3
2	p.Glu2Glu	12.6
2	p.Glu2Ala	4.1
2	p.Glu2Gly	3.4
2	p.Glu2Val	3.4
2	p.Glu2Tyr	2.8
2	p.Glu2Cys	3.3
2	p.Glu2Trp	3.7
2	p.Glu2Phe	3.8
3	p.Pro3Asn	4.7

3	p.Pro3Lys	3.5
3	p.Pro3Thr	7.5
3	p.Pro3Arg	4.2
3	p.Pro3Ser	4.3
3	p.Pro3Ile	3.9
3	p.Pro3Met	3.3
3	p.Pro3His	9.4
3	p.Pro3Gln	3.6
3	p.Pro3Pro	9.6
3	p.Pro3Leu	4.3
3	p.Pro3Asp	4.6
3	p.Pro3Glu	4.5
3	p.Pro3Ala	6.0
3	p.Pro3Gly	5.4
3	p.Pro3Val	4.7
3	p.Pro3Tyr	3.4
3	p.Pro3Cys	4.4
3	p.Pro3Trp	3.8
3	p.Pro3Phe	4.9
4	p.Ala4Asn	4.8
4	p.Ala4Lys	3.4
4	p.Ala4Thr	8.7
4	p.Ala4Arg	4.3
4	p.Ala4Ser	4.1
4	p.Ala4Ile	3.4
4	p.Ala4Met	3.6
4	p.Ala4His	5.8
4	p.Ala4Gln	3.9
4	p.Ala4Pro	4.6
4	p.Ala4Leu	3.9
4	p.Ala4Asp	10.1
4	p.Ala4Glu	3.7
4	p.Ala4Ala	8.5
4	p.Ala4Gly	6.0
4	p.Ala4Val	5.2
4	p.Ala4Tyr	3.6
4	p.Ala4Cys	4.2
4	p.Ala4Trp	4.5
4	p.Ala4Phe	3.7
5	p.Ala5Asn	6.2
5	p.Ala5Lys	4.0
5	p.Ala5Thr	4.5
5	p.Ala5Arg	4.5
5	p.Ala5Ser	4.3
5	p.Ala5Ile	6.0

5	p.Ala5Met	4.4
5	p.Ala5His	5.1
5	p.Ala5Gln	5.7
5	p.Ala5Pro	5.1
5	p.Ala5Leu	6.4
5	p.Ala5Asp	5.7
5	p.Ala5Glu	4.5
5	p.Ala5Ala	7.9
5	p.Ala5Gly	3.6
5	p.Ala5Val	6.2
5	p.Ala5Tyr	2.8
5	p.Ala5Cys	3.6
5	p.Ala5Trp	5.9
5	p.Ala5Phe	3.4
6	p.Gly6Asn	4.3
6	p.Gly6Lys	4.5
6	p.Gly6Thr	4.0
6	p.Gly6Arg	4.0
6	p.Gly6Ser	6.7
6	p.Gly6Ile	2.7
6	p.Gly6Met	4.3
6	p.Gly6His	2.7
6	p.Gly6Gln	3.5
6	p.Gly6Pro	3.6
6	p.Gly6Leu	4.3
6	p.Gly6Asp	8.4
6	p.Gly6Glu	3.8
6	p.Gly6Ala	6.6
6	p.Gly6Gly	10.0
6	p.Gly6Val	5.0
6	p.Gly6Tyr	3.3
6	p.Gly6Cys	11.0
6	p.Gly6Trp	4.3
6	p.Gly6Phe	3.1
7	p.Ser7Asn	5.6
7	p.Ser7Lys	5.9
7	p.Ser7Thr	7.3
7	p.Ser7Arg	6.7
7	p.Ser7Ser	3.4
7	p.Ser7Ile	5.6
7	p.Ser7Met	7.3
7	p.Ser7His	6.7
7	p.Ser7Gln	6.1
7	p.Ser7Pro	8.9
7	p.Ser7Leu	6.6

7	p.Ser7Asp	3.7
7	p.Ser7Glu	1.5
7	p.Ser7Ala	4.3
7	p.Ser7Gly	0.9
7	p.Ser7Val	2.6
7	p.Ser7Tyr	4.1
7	p.Ser7Cys	4.3
7	p.Ser7Trp	3.6
7	p.Ser7Phe	4.8
8	p.Ser8Asn	5.5
8	p.Ser8Lys	4.3
8	p.Ser8Thr	6.0
8	p.Ser8Arg	5.9
8	p.Ser8Ser	6.1
8	p.Ser8Ile	6.2
8	p.Ser8Met	3.4
8	p.Ser8His	3.7
8	p.Ser8Gln	4.7
8	p.Ser8Pro	4.8
8	p.Ser8Leu	4.8
8	p.Ser8Asp	5.6
8	p.Ser8Glu	4.6
8	p.Ser8Ala	4.2
8	p.Ser8Gly	4.7
8	p.Ser8Val	5.6
8	p.Ser8Tyr	4.4
8	p.Ser8Cys	4.5
8	p.Ser8Trp	6.1
8	p.Ser8Phe	4.6
9	p.Met9Asn	4.6
9	p.Met9Lys	7.0
9	p.Met9Thr	5.1
9	p.Met9Arg	6.4
9	p.Met9Ser	4.0
9	p.Met9Ile	5.9
9	p.Met9Met	0.0
9	p.Met9His	3.1
9	p.Met9Gln	4.1
9	p.Met9Pro	3.9
9	p.Met9Leu	6.8
9	p.Met9Asp	4.9
9	p.Met9Glu	4.6
9	p.Met9Ala	4.6
9	p.Met9Gly	4.2
9	p.Met9Val	8.6

9	p.Met9Tyr	6.1
9	p.Met9Cys	5.6
9	p.Met9Trp	5.2
9	p.Met9Phe	5.5
10	p.Glu10Asn	3.5
10	p.Glu10Lys	4.9
10	p.Glu10Thr	2.5
10	p.Glu10Arg	4.9
10	p.Glu10Ser	2.6
10	p.Glu10Ile	3.4
10	p.Glu10Met	5.9
10	p.Glu10His	3.2
10	p.Glu10Gln	4.8
10	p.Glu10Pro	4.8
10	p.Glu10Leu	3.2
10	p.Glu10Asp	13.4
10	p.Glu10Glu	10.8
10	p.Glu10Ala	2.9
10	p.Glu10Gly	4.4
10	p.Glu10Val	4.6
10	p.Glu10Tyr	5.7
10	p.Glu10Cys	5.8
10	p.Glu10Trp	4.0
10	p.Glu10Phe	4.5
11	p.Pro11Asn	4.8
11	p.Pro11Lys	3.7
11	p.Pro11Thr	4.9
11	p.Pro11Arg	4.0
11	p.Pro11Ser	4.9
11	p.Pro11Ile	5.1
11	p.Pro11Met	5.4
11	p.Pro11His	7.0
11	p.Pro11Gln	5.1
11	p.Pro11Pro	5.9
11	p.Pro11Leu	4.3
11	p.Pro11Asp	5.6
11	p.Pro11Glu	4.6
11	p.Pro11Ala	6.1
11	p.Pro11Gly	6.0
11	p.Pro11Val	5.3
11	p.Pro11Tyr	4.6
11	p.Pro11Cys	3.8
11	p.Pro11Trp	4.7
11	p.Pro11Phe	4.4
12	p.Ser12Asn	3.8

12	p.Ser12Lys	4.8
12	p.Ser12Thr	4.0
12	p.Ser12Arg	4.9
12	p.Ser12Ser	4.5
12	p.Ser12Ile	4.1
12	p.Ser12Met	6.3
12	p.Ser12His	4.9
12	p.Ser12Gln	6.4
12	p.Ser12Pro	5.9
12	p.Ser12Leu	6.5
12	p.Ser12Asp	5.4
12	p.Ser12Glu	4.6
12	p.Ser12Ala	3.5
12	p.Ser12Gly	4.4
12	p.Ser12Val	3.5
12	p.Ser12Tyr	4.8
12	p.Ser12Cys	5.3
12	p.Ser12Trp	6.1
12	p.Ser12Phe	6.2
13	p.Ala13Asn	4.9
13	p.Ala13Lys	4.3
13	p.Ala13Thr	5.1
13	p.Ala13Arg	6.3
13	p.Ala13Ser	5.3
13	p.Ala13Ile	4.6
13	p.Ala13Met	5.8
13	p.Ala13His	3.5
13	p.Ala13Gln	4.9
13	p.Ala13Pro	5.2
13	p.Ala13Leu	4.6
13	p.Ala13Asp	5.7
13	p.Ala13Glu	5.5
13	p.Ala13Ala	8.6
13	p.Ala13Gly	4.6
13	p.Ala13Val	4.0
13	p.Ala13Tyr	4.5
13	p.Ala13Cys	3.7
13	p.Ala13Trp	3.6
13	p.Ala13Phe	5.2
14	p.Asp14Asn	4.3
14	p.Asp14Lys	4.6
14	p.Asp14Thr	4.4
14	p.Asp14Arg	4.8
14	p.Asp14Ser	4.6
14	p.Asp14Ile	4.1

14	p.Asp14Met	3.6
14	p.Asp14His	3.9
14	p.Asp14Gln	5.6
14	p.Asp14Pro	4.3
14	p.Asp14Leu	5.2
14	p.Asp14Asp	7.1
14	p.Asp14Glu	11.9
14	p.Asp14Ala	4.3
14	p.Asp14Gly	5.6
14	p.Asp14Val	4.6
14	p.Asp14Tyr	4.5
14	p.Asp14Cys	4.4
14	p.Asp14Trp	5.1
14	p.Asp14Phe	3.3
15	p.Trp15Asn	5.6
15	p.Trp15Lys	5.3
15	p.Trp15Thr	3.3
15	p.Trp15Arg	5.8
15	p.Trp15Ser	6.9
15	p.Trp15Ile	3.4
15	p.Trp15Met	2.6
15	p.Trp15His	4.1
15	p.Trp15Gln	5.0
15	p.Trp15Pro	4.1
15	p.Trp15Leu	7.3
15	p.Trp15Asp	7.6
15	p.Trp15Glu	5.4
15	p.Trp15Ala	6.1
15	p.Trp15Gly	4.1
15	p.Trp15Val	3.0
15	p.Trp15Tyr	5.8
15	p.Trp15Cys	7.4
15	p.Trp15Trp	0.0
15	p.Trp15Phe	7.1
16	p.Leu16Asn	4.1
16	p.Leu16Lys	6.6
16	p.Leu16Thr	4.3
16	p.Leu16Arg	4.7
16	p.Leu16Ser	4.6
16	p.Leu16Ile	4.2
16	p.Leu16Met	4.0
16	p.Leu16His	7.6
16	p.Leu16Gln	5.6
16	p.Leu16Pro	10.2
16	p.Leu16Leu	2.8

16	p.Leu16Asp	3.4
16	p.Leu16Glu	5.4
16	p.Leu16Ala	3.9
16	p.Leu16Gly	4.7
16	p.Leu16Val	5.1
16	p.Leu16Tyr	4.3
16	p.Leu16Cys	4.5
16	p.Leu16Trp	4.8
16	p.Leu16Phe	5.2
17	p.Ala17Asn	4.6
17	p.Ala17Lys	6.3
17	p.Ala17Thr	4.8
17	p.Ala17Arg	5.1
17	p.Ala17Ser	5.6
17	p.Ala17Ile	4.1
17	p.Ala17Met	3.3
17	p.Ala17His	3.7
17	p.Ala17Gln	5.4
17	p.Ala17Pro	6.2
17	p.Ala17Leu	3.1
17	p.Ala17Asp	4.7
17	p.Ala17Glu	4.3
17	p.Ala17Ala	9.9
17	p.Ala17Gly	7.6
17	p.Ala17Val	5.1
17	p.Ala17Tyr	3.9
17	p.Ala17Cys	2.9
17	p.Ala17Trp	4.7
17	p.Ala17Phe	4.9
18	p.Thr18Asn	7.0
18	p.Thr18Lys	5.3
18	p.Thr18Thr	5.9
18	p.Thr18Arg	4.2
18	p.Thr18Ser	4.7
18	p.Thr18Ile	4.8
18	p.Thr18Met	7.2
18	p.Thr18His	2.7
18	p.Thr18Gln	5.7
18	p.Thr18Pro	5.0
18	p.Thr18Leu	6.2
18	p.Thr18Asp	3.8
18	p.Thr18Glu	5.5
18	p.Thr18Ala	6.8
18	p.Thr18Gly	3.1
18	p.Thr18Val	5.9

18	p.Thr18Tyr	3.4
18	p.Thr18Cys	4.2
18	p.Thr18Trp	5.8
18	p.Thr18Phe	2.7
19	p.Ala19Asn	4.3
19	p.Ala19Lys	4.8
19	p.Ala19Thr	5.3
19	p.Ala19Arg	3.6
19	p.Ala19Ser	4.3
19	p.Ala19Ile	5.1
19	p.Ala19Met	3.5
19	p.Ala19His	6.3
19	p.Ala19Gln	4.7
19	p.Ala19Pro	5.2
19	p.Ala19Leu	5.2
19	p.Ala19Asp	4.2
19	p.Ala19Glu	6.9
19	p.Ala19Ala	11.5
19	p.Ala19Gly	4.4
19	p.Ala19Val	3.7
19	p.Ala19Tyr	4.3
19	p.Ala19Cys	3.4
19	p.Ala19Trp	5.0
19	p.Ala19Phe	4.5
20	p.Ala20Asn	4.7
20	p.Ala20Lys	6.6
20	p.Ala20Thr	6.5
20	p.Ala20Arg	3.8
20	p.Ala20Ser	3.3
20	p.Ala20Ile	4.2
20	p.Ala20Met	5.4
20	p.Ala20His	3.1
20	p.Ala20Gln	4.3
20	p.Ala20Pro	5.4
20	p.Ala20Leu	4.5
20	p.Ala20Asp	7.0
20	p.Ala20Glu	6.7
20	p.Ala20Ala	6.8
20	p.Ala20Gly	4.0
20	p.Ala20Val	5.7
20	p.Ala20Tyr	3.8
20	p.Ala20Cys	5.5
20	p.Ala20Trp	4.5
20	p.Ala20Phe	4.3
21	p.Ala21Asn	2.3

21	p.Ala21Lys	4.0
21	p.Ala21Thr	3.1
21	p.Ala21Arg	2.7
21	p.Ala21Ser	2.4
21	p.Ala21Ile	1.8
21	p.Ala21Met	4.6
21	p.Ala21His	4.7
21	p.Ala21Gln	5.8
21	p.Ala21Pro	4.1
21	p.Ala21Leu	4.9
21	p.Ala21Asp	4.7
21	p.Ala21Glu	15.6
21	p.Ala21Ala	7.6
21	p.Ala21Gly	5.8
21	p.Ala21Val	10.4
21	p.Ala21Tyr	4.1
21	p.Ala21Cys	4.8
21	p.Ala21Trp	3.7
21	p.Ala21Phe	2.9
22	p.Arg22Asn	5.1
22	p.Arg22Lys	5.0
22	p.Arg22Thr	4.7
22	p.Arg22Arg	39.7
22	p.Arg22Ser	2.8
22	p.Arg22Ile	4.2
22	p.Arg22Met	4.9
22	p.Arg22His	1.5
22	p.Arg22Gln	5.1
22	p.Arg22Pro	1.8
22	p.Arg22Leu	3.4
22	p.Arg22Asp	1.4
22	p.Arg22Glu	3.6
22	p.Arg22Ala	1.5
22	p.Arg22Gly	1.5
22	p.Arg22Val	1.7
22	p.Arg22Tyr	3.8
22	p.Arg22Cys	1.9
22	p.Arg22Trp	4.1
22	p.Arg22Phe	2.4
23	p.Gly23Asn	5.1
23	p.Gly23Lys	4.7
23	p.Gly23Thr	5.4
23	p.Gly23Arg	8.4
23	p.Gly23Ser	4.0
23	p.Gly23Ile	4.4

23	p.Gly23Met	5.6
23	p.Gly23His	5.1
23	p.Gly23Gln	4.8
23	p.Gly23Pro	4.7
23	p.Gly23Leu	1.6
23	p.Gly23Asp	4.6
23	p.Gly23Glu	5.3
23	p.Gly23Ala	5.3
23	p.Gly23Gly	5.1
23	p.Gly23Val	6.2
23	p.Gly23Tyr	4.8
23	p.Gly23Cys	5.1
23	p.Gly23Trp	4.7
23	p.Gly23Phe	5.1
24	p.Arg24Asn	4.1
24	p.Arg24Lys	4.9
24	p.Arg24Thr	3.9
24	p.Arg24Arg	12.1
24	p.Arg24Ser	5.1
24	p.Arg24Ile	3.6
24	p.Arg24Met	4.6
24	p.Arg24His	4.5
24	p.Arg24Gln	5.4
24	p.Arg24Pro	3.5
24	p.Arg24Leu	4.2
24	p.Arg24Asp	6.6
24	p.Arg24Glu	4.2
24	p.Arg24Ala	4.7
24	p.Arg24Gly	3.3
24	p.Arg24Val	5.7
24	p.Arg24Tyr	4.3
24	p.Arg24Cys	4.2
24	p.Arg24Trp	6.2
24	p.Arg24Phe	4.9
25	p.Val25Asn	3.9
25	p.Val25Lys	5.6
25	p.Val25Thr	4.2
25	p.Val25Arg	7.4
25	p.Val25Ser	4.2
25	p.Val25Ile	3.9
25	p.Val25Met	10.1
25	p.Val25His	2.6
25	p.Val25Gln	2.2
25	p.Val25Pro	1.5
25	p.Val25Leu	5.1

25	p.Val25Asp	5.9
25	p.Val25Glu	9.8
25	p.Val25Ala	3.3
25	p.Val25Gly	5.3
25	p.Val25Val	6.4
25	p.Val25Tyr	4.6
25	p.Val25Cys	4.3
25	p.Val25Trp	5.8
25	p.Val25Phe	3.6
26	p.Glu26Asn	2.1
26	p.Glu26Lys	3.4
26	p.Glu26Thr	1.7
26	p.Glu26Arg	9.3
26	p.Glu26Ser	2.0
26	p.Glu26Ile	5.3
26	p.Glu26Met	5.8
26	p.Glu26His	3.1
26	p.Glu26Gln	3.6
26	p.Glu26Pro	1.0
26	p.Glu26Leu	4.0
26	p.Glu26Asp	15.7
26	p.Glu26Glu	15.1
26	p.Glu26Ala	5.5
26	p.Glu26Gly	2.4
26	p.Glu26Val	6.2
26	p.Glu26Tyr	3.4
26	p.Glu26Cys	0.8
26	p.Glu26Trp	5.7
26	p.Glu26Phe	3.8
27	p.Glu27Asn	1.2
27	p.Glu27Lys	14.1
27	p.Glu27Thr	0.2
27	p.Glu27Arg	5.1
27	p.Glu27Ser	3.1
27	p.Glu27Ile	0.6
27	p.Glu27Met	7.4
27	p.Glu27His	0.0
27	p.Glu27Gln	6.4
27	p.Glu27Pro	0.8
27	p.Glu27Leu	4.4
27	p.Glu27Asp	6.8
27	p.Glu27Glu	14.1
27	p.Glu27Ala	0.7
27	p.Glu27Gly	2.3
27	p.Glu27Val	10.5

27	p.Glu27Tyr	2.5
27	p.Glu27Cys	2.4
27	p.Glu27Trp	9.2
27	p.Glu27Phe	8.1
28	p.Val28Asn	4.1
28	p.Val28Lys	4.4
28	p.Val28Thr	3.7
28	p.Val28Arg	4.3
28	p.Val28Ser	4.7
28	p.Val28Ile	7.0
28	p.Val28Met	3.8
28	p.Val28His	2.9
28	p.Val28Gln	4.2
28	p.Val28Pro	3.8
28	p.Val28Leu	5.0
28	p.Val28Asp	6.5
28	p.Val28Glu	5.7
28	p.Val28Ala	7.5
28	p.Val28Gly	8.9
28	p.Val28Val	7.1
28	p.Val28Tyr	3.8
28	p.Val28Cys	3.8
28	p.Val28Trp	4.3
28	p.Val28Phe	4.6
29	p.Arg29Asn	4.4
29	p.Arg29Lys	5.7
29	p.Arg29Thr	3.9
29	p.Arg29Arg	16.6
29	p.Arg29Ser	9.7
29	p.Arg29Ile	4.0
29	p.Arg29Met	4.8
29	p.Arg29His	2.0
29	p.Arg29Gln	5.7
29	p.Arg29Pro	1.4
29	p.Arg29Leu	5.3
29	p.Arg29Asp	4.7
29	p.Arg29Glu	3.5
29	p.Arg29Ala	4.7
29	p.Arg29Gly	5.7
29	p.Arg29Val	3.2
29	p.Arg29Tyr	4.1
29	p.Arg29Cys	5.7
29	p.Arg29Trp	3.0
29	p.Arg29Phe	1.8
30	p.Ala30Asn	5.0

30	p.Ala30Lys	4.1
30	p.Ala30Thr	5.0
30	p.Ala30Arg	5.6
30	p.Ala30Ser	3.6
30	p.Ala30Ile	3.6
30	p.Ala30Met	6.4
30	p.Ala30His	3.6
30	p.Ala30Gln	4.4
30	p.Ala30Pro	3.6
30	p.Ala30Leu	3.3
30	p.Ala30Asp	3.8
30	p.Ala30Glu	12.6
30	p.Ala30Ala	4.4
30	p.Ala30Gly	3.6
30	p.Ala30Val	11.6
30	p.Ala30Tyr	3.6
30	p.Ala30Cys	2.7
30	p.Ala30Trp	6.8
30	p.Ala30Phe	2.8
31	p.Leu31Asn	2.5
31	p.Leu31Lys	3.5
31	p.Leu31Thr	3.1
31	p.Leu31Arg	3.3
31	p.Leu31Ser	3.3
31	p.Leu31Ile	10.3
31	p.Leu31Met	4.7
31	p.Leu31His	6.8
31	p.Leu31Gln	4.4
31	p.Leu31Pro	15.8
31	p.Leu31Leu	11.4
31	p.Leu31Asp	3.8
31	p.Leu31Glu	1.7
31	p.Leu31Ala	3.0
31	p.Leu31Gly	4.0
31	p.Leu31Val	2.6
31	p.Leu31Tyr	4.3
31	p.Leu31Cys	2.5
31	p.Leu31Trp	2.0
31	p.Leu31Phe	6.9
32	p.Leu32Asn	4.6
32	p.Leu32Lys	4.9
32	p.Leu32Thr	4.2
32	p.Leu32Arg	4.6
32	p.Leu32Ser	4.9
32	p.Leu32Ile	5.8

32	p.Leu32Met	6.4
32	p.Leu32His	4.4
32	p.Leu32Gln	5.7
32	p.Leu32Pro	6.5
32	p.Leu32Leu	5.5
32	p.Leu32Asp	2.7
32	p.Leu32Glu	4.3
32	p.Leu32Ala	4.9
32	p.Leu32Gly	3.9
32	p.Leu32Val	6.4
32	p.Leu32Tyr	5.6
32	p.Leu32Cys	4.3
32	p.Leu32Trp	4.5
32	p.Leu32Phe	5.9
33	p.Glu33Asn	7.1
33	p.Glu33Lys	4.0
33	p.Glu33Thr	4.6
33	p.Glu33Arg	3.8
33	p.Glu33Ser	1.8
33	p.Glu33Ile	5.6
33	p.Glu33Met	4.2
33	p.Glu33His	5.0
33	p.Glu33Gln	5.0
33	p.Glu33Pro	2.8
33	p.Glu33Leu	5.2
33	p.Glu33Asp	8.8
33	p.Glu33Glu	9.0
33	p.Glu33Ala	3.4
33	p.Glu33Gly	4.4
33	p.Glu33Val	5.6
33	p.Glu33Tyr	4.8
33	p.Glu33Cys	4.7
33	p.Glu33Trp	5.5
33	p.Glu33Phe	4.6
34	p.Ala34Asn	3.6
34	p.Ala34Lys	6.4
34	p.Ala34Thr	5.3
34	p.Ala34Arg	6.3
34	p.Ala34Ser	3.4
34	p.Ala34Ile	2.5
34	p.Ala34Met	4.8
34	p.Ala34His	3.4
34	p.Ala34Gln	4.4
34	p.Ala34Pro	4.6
34	p.Ala34Leu	4.8

34	p.Ala34Asp	10.6
34	p.Ala34Glu	5.6
34	p.Ala34Ala	7.2
34	p.Ala34Gly	5.1
34	p.Ala34Val	4.1
34	p.Ala34Tyr	4.9
34	p.Ala34Cys	4.5
34	p.Ala34Trp	4.0
34	p.Ala34Phe	4.4
35	p.Gly35Asn	3.3
35	p.Gly35Lys	4.3
35	p.Gly35Thr	4.2
35	p.Gly35Arg	4.9
35	p.Gly35Ser	3.4
35	p.Gly35Ile	3.1
35	p.Gly35Met	5.2
35	p.Gly35His	5.1
35	p.Gly35Gln	5.4
35	p.Gly35Pro	4.4
35	p.Gly35Leu	5.2
35	p.Gly35Asp	4.6
35	p.Gly35Glu	5.2
35	p.Gly35Ala	3.5
35	p.Gly35Gly	10.4
35	p.Gly35Val	6.6
35	p.Gly35Tyr	4.7
35	p.Gly35Cys	4.8
35	p.Gly35Trp	6.3
35	p.Gly35Phe	5.4
36	p.Ala36Asn	4.9
36	p.Ala36Lys	5.4
36	p.Ala36Thr	3.4
36	p.Ala36Arg	4.0
36	p.Ala36Ser	3.4
36	p.Ala36Ile	5.3
36	p.Ala36Met	4.8
36	p.Ala36His	4.5
36	p.Ala36Gln	5.8
36	p.Ala36Pro	5.5
36	p.Ala36Leu	5.5
36	p.Ala36Asp	4.6
36	p.Ala36Glu	6.4
36	p.Ala36Ala	8.0
36	p.Ala36Gly	2.6
36	p.Ala36Val	5.9

36	p.Ala36Tyr	4.4
36	p.Ala36Cys	4.2
36	p.Ala36Trp	6.4
36	p.Ala36Phe	5.1
37	p.Leu37Asn	5.4
37	p.Leu37Lys	4.9
37	p.Leu37Thr	3.1
37	p.Leu37Arg	3.3
37	p.Leu37Ser	3.7
37	p.Leu37Ile	5.0
37	p.Leu37Met	5.9
37	p.Leu37His	4.8
37	p.Leu37Gln	7.4
37	p.Leu37Pro	3.6
37	p.Leu37Leu	7.3
37	p.Leu37Asp	3.4
37	p.Leu37Glu	4.6
37	p.Leu37Ala	4.7
37	p.Leu37Gly	3.0
37	p.Leu37Val	9.3
37	p.Leu37Tyr	3.8
37	p.Leu37Cys	3.2
37	p.Leu37Trp	6.3
37	p.Leu37Phe	7.1
38	p.Pro38Asn	4.2
38	p.Pro38Lys	3.5
38	p.Pro38Thr	3.4
38	p.Pro38Arg	3.9
38	p.Pro38Ser	3.2
38	p.Pro38Ile	3.1
38	p.Pro38Met	5.2
38	p.Pro38His	3.3
38	p.Pro38Gln	10.8
38	p.Pro38Pro	9.5
38	p.Pro38Leu	6.3
38	p.Pro38Asp	4.3
38	p.Pro38Glu	6.9
38	p.Pro38Ala	3.4
38	p.Pro38Gly	4.6
38	p.Pro38Val	6.0
38	p.Pro38Tyr	2.7
38	p.Pro38Cys	5.0
38	p.Pro38Trp	4.4
38	p.Pro38Phe	6.5
39	p.Asn39Asn	7.6

39	p.Asn39Lys	8.2
39	p.Asn39Thr	3.0
39	p.Asn39Arg	4.0
39	p.Asn39Ser	5.0
39	p.Asn39Ile	4.1
39	p.Asn39Met	6.3
39	p.Asn39His	4.2
39	p.Asn39Gln	2.9
39	p.Asn39Pro	3.8
39	p.Asn39Leu	6.5
39	p.Asn39Asp	6.4
39	p.Asn39Glu	6.0
39	p.Asn39Ala	3.9
39	p.Asn39Gly	6.1
39	p.Asn39Val	5.2
39	p.Asn39Tyr	3.1
39	p.Asn39Cys	5.2
39	p.Asn39Trp	3.8
39	p.Asn39Phe	4.6
40	p.Ala40Asn	5.0
40	p.Ala40Lys	6.2
40	p.Ala40Thr	4.3
40	p.Ala40Arg	4.5
40	p.Ala40Ser	2.5
40	p.Ala40Ile	3.1
40	p.Ala40Met	3.4
40	p.Ala40His	2.9
40	p.Ala40Gln	4.0
40	p.Ala40Pro	3.9
40	p.Ala40Leu	4.0
40	p.Ala40Asp	5.1
40	p.Ala40Glu	14.6
40	p.Ala40Ala	5.8
40	p.Ala40Gly	4.5
40	p.Ala40Val	11.0
40	p.Ala40Tyr	4.1
40	p.Ala40Cys	3.3
40	p.Ala40Trp	3.8
40	p.Ala40Phe	4.1
41	p.Pro41Asn	4.9
41	p.Pro41Lys	4.3
41	p.Pro41Thr	4.0
41	p.Pro41Arg	3.4
41	p.Pro41Ser	6.0
41	p.Pro41Ile	3.9

41	p.Pro41Met	6.1
41	p.Pro41His	3.9
41	p.Pro41Gln	5.7
41	p.Pro41Pro	4.9
41	p.Pro41Leu	5.1
41	p.Pro41Asp	6.5
41	p.Pro41Glu	5.2
41	p.Pro41Ala	6.0
41	p.Pro41Gly	2.9
41	p.Pro41Val	6.6
41	p.Pro41Tyr	5.5
41	p.Pro41Cys	5.2
41	p.Pro41Trp	4.0
41	p.Pro41Phe	5.9
42	p.Asn42Asn	4.8
42	p.Asn42Lys	6.4
42	p.Asn42Thr	7.5
42	p.Asn42Arg	5.4
42	p.Asn42Ser	5.8
42	p.Asn42Ile	5.8
42	p.Asn42Met	3.6
42	p.Asn42His	7.8
42	p.Asn42Gln	4.4
42	p.Asn42Pro	3.0
42	p.Asn42Leu	3.6
42	p.Asn42Asp	5.5
42	p.Asn42Glu	4.6
42	p.Asn42Ala	3.0
42	p.Asn42Gly	4.4
42	p.Asn42Val	5.5
42	p.Asn42Tyr	5.4
42	p.Asn42Cys	4.3
42	p.Asn42Trp	4.2
42	p.Asn42Phe	5.1
43	p.Ser43Asn	2.8
43	p.Ser43Lys	4.6
43	p.Ser43Thr	7.4
43	p.Ser43Arg	3.8
43	p.Ser43Ser	3.4
43	p.Ser43Ile	2.3
43	p.Ser43Met	4.0
43	p.Ser43His	4.3
43	p.Ser43Gln	4.0
43	p.Ser43Pro	7.7
43	p.Ser43Leu	2.7

43	p.Ser43Asp	4.0
43	p.Ser43Glu	3.5
43	p.Ser43Ala	13.7
43	p.Ser43Gly	2.0
43	p.Ser43Val	4.3
43	p.Ser43Tyr	10.9
43	p.Ser43Cys	4.4
43	p.Ser43Trp	3.7
43	p.Ser43Phe	6.5
44	p.Tyr44Asn	6.1
44	p.Tyr44Lys	5.3
44	p.Tyr44Thr	5.3
44	p.Tyr44Arg	3.6
44	p.Tyr44Ser	4.6
44	p.Tyr44Ile	4.8
44	p.Tyr44Met	4.0
44	p.Tyr44His	6.7
44	p.Tyr44Gln	2.9
44	p.Tyr44Pro	3.5
44	p.Tyr44Leu	4.0
44	p.Tyr44Asp	3.0
44	p.Tyr44Glu	6.1
44	p.Tyr44Ala	4.4
44	p.Tyr44Gly	4.7
44	p.Tyr44Val	6.4
44	p.Tyr44Tyr	10.5
44	p.Tyr44Cys	3.9
44	p.Tyr44Trp	6.1
44	p.Tyr44Phe	4.0
45	p.Gly45Asn	4.3
45	p.Gly45Lys	4.7
45	p.Gly45Thr	3.0
45	p.Gly45Arg	8.9
45	p.Gly45Ser	6.3
45	p.Gly45Ile	5.1
45	p.Gly45Met	4.3
45	p.Gly45His	4.1
45	p.Gly45Gln	4.6
45	p.Gly45Pro	3.2
45	p.Gly45Leu	5.5
45	p.Gly45Asp	5.4
45	p.Gly45Glu	4.7
45	p.Gly45Ala	3.6
45	p.Gly45Gly	9.8
45	p.Gly45Val	4.3

45	p.Gly45Tyr	4.0
45	p.Gly45Cys	5.0
45	p.Gly45Trp	3.8
45	p.Gly45Phe	5.4
46	p.Arg46Asn	4.9
46	p.Arg46Lys	3.4
46	p.Arg46Thr	5.0
46	p.Arg46Arg	4.4
46	p.Arg46Ser	9.2
46	p.Arg46Ile	6.0
46	p.Arg46Met	2.7
46	p.Arg46His	9.1
46	p.Arg46Gln	3.9
46	p.Arg46Pro	7.5
46	p.Arg46Leu	5.7
46	p.Arg46Asp	3.3
46	p.Arg46Glu	2.9
46	p.Arg46Ala	3.2
46	p.Arg46Gly	5.1
46	p.Arg46Val	2.7
46	p.Arg46Tyr	5.7
46	p.Arg46Cys	7.3
46	p.Arg46Trp	4.0
46	p.Arg46Phe	4.0
47	p.Arg47Asn	3.6
47	p.Arg47Lys	3.5
47	p.Arg47Thr	4.0
47	p.Arg47Arg	4.8
47	p.Arg47Ser	10.4
47	p.Arg47Ile	3.3
47	p.Arg47Met	4.0
47	p.Arg47His	9.5
47	p.Arg47Gln	4.5
47	p.Arg47Pro	4.8
47	p.Arg47Leu	3.8
47	p.Arg47Asp	4.3
47	p.Arg47Glu	4.9
47	p.Arg47Ala	3.3
47	p.Arg47Gly	5.0
47	p.Arg47Val	3.8
47	p.Arg47Tyr	5.0
47	p.Arg47Cys	8.1
47	p.Arg47Trp	4.5
47	p.Arg47Phe	4.7
48	p.Pro48Asn	4.7

48	p.Pro48Lys	5.5
48	p.Pro48Thr	5.1
48	p.Pro48Arg	5.6
48	p.Pro48Ser	4.6
48	p.Pro48Ile	6.7
48	p.Pro48Met	6.6
48	p.Pro48His	4.4
48	p.Pro48Gln	5.3
48	p.Pro48Pro	5.2
48	p.Pro48Leu	7.1
48	p.Pro48Asp	4.6
48	p.Pro48Glu	4.7
48	p.Pro48Ala	3.5
48	p.Pro48Gly	4.7
48	p.Pro48Val	4.8
48	p.Pro48Tyr	3.8
48	p.Pro48Cys	3.4
48	p.Pro48Trp	5.6
48	p.Pro48Phe	4.3
49	p.Ile49Asn	3.5
49	p.Ile49Lys	5.2
49	p.Ile49Thr	3.9
49	p.Ile49Arg	8.3
49	p.Ile49Ser	4.8
49	p.Ile49Ile	7.3
49	p.Ile49Met	7.5
49	p.Ile49His	4.7
49	p.Ile49Gln	4.8
49	p.Ile49Pro	4.5
49	p.Ile49Leu	4.6
49	p.Ile49Asp	5.5
49	p.Ile49Glu	5.4
49	p.Ile49Ala	3.7
49	p.Ile49Gly	5.2
49	p.Ile49Val	5.5
49	p.Ile49Tyr	3.6
49	p.Ile49Cys	3.0
49	p.Ile49Trp	4.9
49	p.Ile49Phe	4.2
50	p.Gln50Asn	3.8
50	p.Gln50Lys	4.2
50	p.Gln50Thr	4.3
50	p.Gln50Arg	3.9
50	p.Gln50Ser	3.9
50	p.Gln50Ile	4.2

50	p.Gln50Met	5.4
50	p.Gln50His	9.2
50	p.Gln50Gln	7.5
50	p.Gln50Pro	5.7
50	p.Gln50Leu	5.1
50	p.Gln50Asp	4.7
50	p.Gln50Glu	6.0
50	p.Gln50Ala	4.4
50	p.Gln50Gly	4.1
50	p.Gln50Val	6.3
50	p.Gln50Tyr	4.6
50	p.Gln50Cys	3.7
50	p.Gln50Trp	3.8
50	p.Gln50Phe	5.1
51	p.Val51Asn	2.1
51	p.Val51Lys	3.6
51	p.Val51Thr	2.7
51	p.Val51Arg	3.2
51	p.Val51Ser	3.1
51	p.Val51Ile	7.2
51	p.Val51Met	4.8
51	p.Val51His	4.1
51	p.Val51Gln	6.2
51	p.Val51Pro	5.7
51	p.Val51Leu	6.3
51	p.Val51Asp	6.2
51	p.Val51Glu	4.9
51	p.Val51Ala	3.7
51	p.Val51Gly	2.5
51	p.Val51Val	14.3
51	p.Val51Tyr	4.5
51	p.Val51Cys	7.5
51	p.Val51Trp	4.0
51	p.Val51Phe	3.5
52	p.Met52Asn	4.3
52	p.Met52Lys	7.2
52	p.Met52Thr	4.5
52	p.Met52Arg	4.2
52	p.Met52Ser	5.5
52	p.Met52Ile	5.5
52	p.Met52Met	0.0
52	p.Met52His	4.5
52	p.Met52Gln	4.3
52	p.Met52Pro	3.9
52	p.Met52Leu	9.7

52	p.Met52Asp	3.7
52	p.Met52Glu	4.8
52	p.Met52Ala	5.7
52	p.Met52Gly	7.2
52	p.Met52Val	7.0
52	p.Met52Tyr	4.7
52	p.Met52Cys	5.1
52	p.Met52Trp	3.9
52	p.Met52Phe	4.3
53	p.Met53Asn	4.9
53	p.Met53Lys	6.9
53	p.Met53Thr	5.1
53	p.Met53Arg	4.7
53	p.Met53Ser	4.1
53	p.Met53Ile	4.7
53	p.Met53Met	0.0
53	p.Met53His	5.9
53	p.Met53Gln	6.5
53	p.Met53Pro	4.4
53	p.Met53Leu	8.6
53	p.Met53Asp	3.9
53	p.Met53Glu	5.7
53	p.Met53Ala	5.5
53	p.Met53Gly	4.2
53	p.Met53Val	6.3
53	p.Met53Tyr	4.5
53	p.Met53Cys	3.4
53	p.Met53Trp	6.6
53	p.Met53Phe	4.2
54	p.Met54Asn	5.1
54	p.Met54Lys	4.7
54	p.Met54Thr	5.1
54	p.Met54Arg	3.5
54	p.Met54Ser	5.8
54	p.Met54Ile	7.4
54	p.Met54Met	0.0
54	p.Met54His	1.4
54	p.Met54Gln	4.4
54	p.Met54Pro	3.4
54	p.Met54Leu	14.3
54	p.Met54Asp	5.1
54	p.Met54Glu	4.9
54	p.Met54Ala	3.0
54	p.Met54Gly	3.2
54	p.Met54Val	9.6

54	p.Met54Tyr	2.3
54	p.Met54Cys	4.4
54	p.Met54Trp	4.7
54	p.Met54Phe	7.8
55	p.Gly55Asn	3.3
55	p.Gly55Lys	4.4
55	p.Gly55Thr	5.4
55	p.Gly55Arg	11.8
55	p.Gly55Ser	4.6
55	p.Gly55Ile	5.1
55	p.Gly55Met	4.9
55	p.Gly55His	4.0
55	p.Gly55Gln	3.5
55	p.Gly55Pro	6.1
55	p.Gly55Leu	5.8
55	p.Gly55Asp	4.6
55	p.Gly55Glu	5.8
55	p.Gly55Ala	2.8
55	p.Gly55Gly	8.6
55	p.Gly55Val	3.4
55	p.Gly55Tyr	4.9
55	p.Gly55Cys	4.5
55	p.Gly55Trp	3.4
55	p.Gly55Phe	3.1
56	p.Ser56Asn	4.9
56	p.Ser56Lys	7.2
56	p.Ser56Thr	3.6
56	p.Ser56Arg	3.9
56	p.Ser56Ser	5.0
56	p.Ser56Ile	4.7
56	p.Ser56Met	3.0
56	p.Ser56His	5.3
56	p.Ser56Gln	4.8
56	p.Ser56Pro	4.8
56	p.Ser56Leu	6.1
56	p.Ser56Asp	4.7
56	p.Ser56Glu	6.1
56	p.Ser56Ala	5.3
56	p.Ser56Gly	5.9
56	p.Ser56Val	5.7
56	p.Ser56Tyr	2.6
56	p.Ser56Cys	4.5
56	p.Ser56Trp	6.5
56	p.Ser56Phe	5.6
57	p.Ala57Asn	5.6

57	p.Ala57Lys	6.2
57	p.Ala57Thr	3.2
57	p.Ala57Arg	4.0
57	p.Ala57Ser	5.2
57	p.Ala57Ile	4.5
57	p.Ala57Met	3.9
57	p.Ala57His	5.4
57	p.Ala57Gln	4.2
57	p.Ala57Pro	5.1
57	p.Ala57Leu	5.0
57	p.Ala57Asp	5.5
57	p.Ala57Glu	5.7
57	p.Ala57Ala	6.1
57	p.Ala57Gly	5.4
57	p.Ala57Val	6.7
57	p.Ala57Tyr	4.3
57	p.Ala57Cys	5.4
57	p.Ala57Trp	4.0
57	p.Ala57Phe	4.5
58	p.Arg58Asn	5.1
58	p.Arg58Lys	7.3
58	p.Arg58Thr	2.3
58	p.Arg58Arg	5.6
58	p.Arg58Ser	5.4
58	p.Arg58Ile	5.3
58	p.Arg58Met	11.8
58	p.Arg58His	3.7
58	p.Arg58Gln	3.5
58	p.Arg58Pro	3.5
58	p.Arg58Leu	5.1
58	p.Arg58Asp	3.8
58	p.Arg58Glu	5.2
58	p.Arg58Ala	3.4
58	p.Arg58Gly	4.1
58	p.Arg58Val	5.7
58	p.Arg58Tyr	4.6
58	p.Arg58Cys	4.8
58	p.Arg58Trp	5.3
58	p.Arg58Phe	4.5
59	p.Val59Asn	4.8
59	p.Val59Lys	6.2
59	p.Val59Thr	5.1
59	p.Val59Arg	5.0
59	p.Val59Ser	5.0
59	p.Val59Ile	4.7

59	p.Val59Met	5.2
59	p.Val59His	4.4
59	p.Val59Gln	5.4
59	p.Val59Pro	4.3
59	p.Val59Leu	5.1
59	p.Val59Asp	5.3
59	p.Val59Glu	6.5
59	p.Val59Ala	3.5
59	p.Val59Gly	3.6
59	p.Val59Val	7.8
59	p.Val59Tyr	4.1
59	p.Val59Cys	4.5
59	p.Val59Trp	5.8
59	p.Val59Phe	3.9
60	p.Ala60Asn	3.6
60	p.Ala60Lys	5.3
60	p.Ala60Thr	5.4
60	p.Ala60Arg	4.5
60	p.Ala60Ser	3.6
60	p.Ala60Ile	5.0
60	p.Ala60Met	6.0
60	p.Ala60His	4.7
60	p.Ala60Gln	6.1
60	p.Ala60Pro	4.6
60	p.Ala60Leu	3.7
60	p.Ala60Asp	4.7
60	p.Ala60Glu	5.5
60	p.Ala60Ala	10.6
60	p.Ala60Gly	4.2
60	p.Ala60Val	4.5
60	p.Ala60Tyr	3.5
60	p.Ala60Cys	4.3
60	p.Ala60Trp	4.9
60	p.Ala60Phe	5.2
61	p.Glu61Asn	4.5
61	p.Glu61Lys	4.6
61	p.Glu61Thr	4.5
61	p.Glu61Arg	4.2
61	p.Glu61Ser	4.4
61	p.Glu61Ile	5.1
61	p.Glu61Met	5.7
61	p.Glu61His	4.1
61	p.Glu61Gln	5.1
61	p.Glu61Pro	3.7
61	p.Glu61Leu	5.8

61	p.Glu61Asp	8.7
61	p.Glu61Glu	8.1
61	p.Glu61Ala	4.2
61	p.Glu61Gly	4.2
61	p.Glu61Val	4.6
61	p.Glu61Tyr	4.8
61	p.Glu61Cys	4.7
61	p.Glu61Trp	5.0
61	p.Glu61Phe	3.7
62	p.Leu62Asn	3.6
62	p.Leu62Lys	4.9
62	p.Leu62Thr	4.8
62	p.Leu62Arg	4.9
62	p.Leu62Ser	4.2
62	p.Leu62Ile	4.5
62	p.Leu62Met	3.9
62	p.Leu62His	4.7
62	p.Leu62Gln	4.2
62	p.Leu62Pro	4.8
62	p.Leu62Leu	8.1
62	p.Leu62Asp	3.0
62	p.Leu62Glu	4.6
62	p.Leu62Ala	4.1
62	p.Leu62Gly	6.2
62	p.Leu62Val	6.4
62	p.Leu62Tyr	4.9
62	p.Leu62Cys	5.5
62	p.Leu62Trp	4.7
62	p.Leu62Phe	7.8
63	p.Leu63Asn	4.2
63	p.Leu63Lys	3.8
63	p.Leu63Thr	3.6
63	p.Leu63Arg	3.8
63	p.Leu63Ser	4.1
63	p.Leu63Ile	12.4
63	p.Leu63Met	4.8
63	p.Leu63His	7.3
63	p.Leu63Gln	3.0
63	p.Leu63Pro	10.7
63	p.Leu63Leu	4.9
63	p.Leu63Asp	3.8
63	p.Leu63Glu	4.8
63	p.Leu63Ala	2.9
63	p.Leu63Gly	3.2
63	p.Leu63Val	4.6

63	p.Leu63Tyr	4.5
63	p.Leu63Cys	2.5
63	p.Leu63Trp	5.1
63	p.Leu63Phe	6.0
64	p.Leu64Asn	3.9
64	p.Leu64Lys	4.0
64	p.Leu64Thr	3.9
64	p.Leu64Arg	4.9
64	p.Leu64Ser	3.8
64	p.Leu64Ile	10.3
64	p.Leu64Met	3.2
64	p.Leu64His	9.1
64	p.Leu64Gln	4.4
64	p.Leu64Pro	5.1
64	p.Leu64Leu	5.7
64	p.Leu64Asp	3.4
64	p.Leu64Glu	4.4
64	p.Leu64Ala	4.8
64	p.Leu64Gly	4.8
64	p.Leu64Val	3.5
64	p.Leu64Tyr	3.9
64	p.Leu64Cys	4.4
64	p.Leu64Trp	5.3
64	p.Leu64Phe	7.2
65	p.Leu65Asn	3.1
65	p.Leu65Lys	5.2
65	p.Leu65Thr	4.4
65	p.Leu65Arg	5.0
65	p.Leu65Ser	5.3
65	p.Leu65Ile	4.5
65	p.Leu65Met	6.5
65	p.Leu65His	3.5
65	p.Leu65Gln	4.8
65	p.Leu65Pro	3.2
65	p.Leu65Leu	6.9
65	p.Leu65Asp	5.6
65	p.Leu65Glu	5.4
65	p.Leu65Ala	4.0
65	p.Leu65Gly	5.8
65	p.Leu65Val	7.4
65	p.Leu65Tyr	4.6
65	p.Leu65Cys	4.4
65	p.Leu65Trp	6.0
65	p.Leu65Phe	4.7
66	p.His66Asn	4.6

66	p.His66Lys	5.6
66	p.His66Thr	3.6
66	p.His66Arg	4.8
66	p.His66Ser	4.9
66	p.His66Ile	4.6
66	p.His66Met	4.7
66	p.His66His	5.8
66	p.His66Gln	5.7
66	p.His66Pro	4.8
66	p.His66Leu	5.9
66	p.His66Asp	2.8
66	p.His66Glu	6.9
66	p.His66Ala	5.3
66	p.His66Gly	5.6
66	p.His66Val	5.1
66	p.His66Tyr	4.4
66	p.His66Cys	4.6
66	p.His66Trp	6.0
66	p.His66Phe	4.2
67	p.Gly67Asn	4.2
67	p.Gly67Lys	5.2
67	p.Gly67Thr	4.3
67	p.Gly67Arg	7.7
67	p.Gly67Ser	3.9
67	p.Gly67Ile	4.0
67	p.Gly67Met	4.2
67	p.Gly67His	5.6
67	p.Gly67Gln	4.7
67	p.Gly67Pro	5.3
67	p.Gly67Leu	4.3
67	p.Gly67Asp	3.8
67	p.Gly67Glu	4.2
67	p.Gly67Ala	4.7
67	p.Gly67Gly	9.1
67	p.Gly67Val	4.4
67	p.Gly67Tyr	5.3
67	p.Gly67Cys	4.3
67	p.Gly67Trp	6.2
67	p.Gly67Phe	4.7
68	p.Ala68Asn	3.2
68	p.Ala68Lys	6.0
68	p.Ala68Thr	5.3
68	p.Ala68Arg	3.3
68	p.Ala68Ser	4.3
68	p.Ala68Ile	3.5

68	p.Ala68Met	6.6
68	p.Ala68His	4.1
68	p.Ala68Gln	3.7
68	p.Ala68Pro	5.3
68	p.Ala68Leu	5.5
68	p.Ala68Asp	5.6
68	p.Ala68Glu	6.1
68	p.Ala68Ala	7.4
68	p.Ala68Gly	4.8
68	p.Ala68Val	6.6
68	p.Ala68Tyr	4.1
68	p.Ala68Cys	5.1
68	p.Ala68Trp	4.9
68	p.Ala68Phe	4.9
69	p.Glu69Asn	4.7
69	p.Glu69Lys	5.7
69	p.Glu69Thr	4.2
69	p.Glu69Arg	5.2
69	p.Glu69Ser	5.9
69	p.Glu69Ile	5.5
69	p.Glu69Met	4.7
69	p.Glu69His	5.2
69	p.Glu69Gln	5.4
69	p.Glu69Pro	5.1
69	p.Glu69Leu	4.7
69	p.Glu69Asp	4.9
69	p.Glu69Glu	7.5
69	p.Glu69Ala	0.5
69	p.Glu69Gly	4.8
69	p.Glu69Val	4.8
69	p.Glu69Tyr	4.9
69	p.Glu69Cys	6.4
69	p.Glu69Trp	6.1
69	p.Glu69Phe	3.9
70	p.Pro70Asn	5.9
70	p.Pro70Lys	3.4
70	p.Pro70Thr	6.1
70	p.Pro70Arg	5.7
70	p.Pro70Ser	5.7
70	p.Pro70Ile	6.7
70	p.Pro70Met	5.9
70	p.Pro70His	2.3
70	p.Pro70Gln	2.4
70	p.Pro70Pro	8.4
70	p.Pro70Leu	7.4

70	p.Pro70Asp	4.8
70	p.Pro70Glu	5.2
70	p.Pro70Ala	5.3
70	p.Pro70Gly	4.5
70	p.Pro70Val	5.0
70	p.Pro70Tyr	3.5
70	p.Pro70Cys	3.0
70	p.Pro70Trp	4.2
70	p.Pro70Phe	4.7
71	p.Asn71Asn	6.8
71	p.Asn71Lys	12.4
71	p.Asn71Thr	4.1
71	p.Asn71Arg	4.5
71	p.Asn71Ser	3.9
71	p.Asn71Ile	3.5
71	p.Asn71Met	2.6
71	p.Asn71His	4.7
71	p.Asn71Gln	4.3
71	p.Asn71Pro	5.1
71	p.Asn71Leu	4.1
71	p.Asn71Asp	4.9
71	p.Asn71Glu	6.6
71	p.Asn71Ala	5.7
71	p.Asn71Gly	3.6
71	p.Asn71Val	3.8
71	p.Asn71Tyr	5.6
71	p.Asn71Cys	4.0
71	p.Asn71Trp	4.0
71	p.Asn71Phe	5.6
72	p.Cys72Asn	4.4
72	p.Cys72Lys	4.4
72	p.Cys72Thr	4.9
72	p.Cys72Arg	4.0
72	p.Cys72Ser	4.4
72	p.Cys72Ile	3.6
72	p.Cys72Met	3.6
72	p.Cys72His	3.0
72	p.Cys72Gln	3.9
72	p.Cys72Pro	4.9
72	p.Cys72Leu	2.8
72	p.Cys72Asp	5.6
72	p.Cys72Glu	5.5
72	p.Cys72Ala	3.3
72	p.Cys72Gly	3.1
72	p.Cys72Val	4.4

72	p.Cys72Tyr	5.6
72	p.Cys72Cys	9.9
72	p.Cys72Trp	13.7
72	p.Cys72Phe	4.8
73	p.Ala73Asn	4.4
73	p.Ala73Lys	3.5
73	p.Ala73Thr	8.3
73	p.Ala73Arg	4.4
73	p.Ala73Ser	5.5
73	p.Ala73Ile	4.3
73	p.Ala73Met	4.4
73	p.Ala73His	5.4
73	p.Ala73Gln	3.3
73	p.Ala73Pro	5.1
73	p.Ala73Leu	5.4
73	p.Ala73Asp	3.0
73	p.Ala73Glu	5.6
73	p.Ala73Ala	9.6
73	p.Ala73Gly	4.0
73	p.Ala73Val	5.2
73	p.Ala73Tyr	5.1
73	p.Ala73Cys	4.7
73	p.Ala73Trp	5.0
73	p.Ala73Phe	3.9
74	p.Asp74Asn	5.2
74	p.Asp74Lys	5.8
74	p.Asp74Thr	4.7
74	p.Asp74Arg	4.5
74	p.Asp74Ser	4.5
74	p.Asp74Ile	4.6
74	p.Asp74Met	5.1
74	p.Asp74His	4.0
74	p.Asp74Gln	3.5
74	p.Asp74Pro	3.2
74	p.Asp74Leu	5.7
74	p.Asp74Asp	8.6
74	p.Asp74Glu	9.2
74	p.Asp74Ala	3.5
74	p.Asp74Gly	4.2
74	p.Asp74Val	4.5
74	p.Asp74Tyr	4.9
74	p.Asp74Cys	4.2
74	p.Asp74Trp	5.8
74	p.Asp74Phe	4.3
75	p.Pro75Asn	4.3

75	p.Pro75Lys	5.6
75	p.Pro75Thr	4.8
75	p.Pro75Arg	5.8
75	p.Pro75Ser	4.0
75	p.Pro75Ile	4.0
75	p.Pro75Met	4.3
75	p.Pro75His	4.5
75	p.Pro75Gln	5.0
75	p.Pro75Pro	8.8
75	p.Pro75Leu	4.4
75	p.Pro75Asp	4.3
75	p.Pro75Glu	5.6
75	p.Pro75Ala	4.3
75	p.Pro75Gly	4.6
75	p.Pro75Val	5.5
75	p.Pro75Tyr	5.7
75	p.Pro75Cys	4.0
75	p.Pro75Trp	5.3
75	p.Pro75Phe	5.2
76	p.Ala76Asn	3.8
76	p.Ala76Lys	5.3
76	p.Ala76Thr	4.5
76	p.Ala76Arg	3.6
76	p.Ala76Ser	4.5
76	p.Ala76Ile	2.5
76	p.Ala76Met	5.2
76	p.Ala76His	5.1
76	p.Ala76Gln	4.0
76	p.Ala76Pro	5.3
76	p.Ala76Leu	3.3
76	p.Ala76Asp	4.3
76	p.Ala76Glu	12.4
76	p.Ala76Ala	5.0
76	p.Ala76Gly	4.4
76	p.Ala76Val	9.4
76	p.Ala76Tyr	3.4
76	p.Ala76Cys	4.5
76	p.Ala76Trp	5.8
76	p.Ala76Phe	3.5
77	p.Thr77Asn	10.6
77	p.Thr77Lys	5.2
77	p.Thr77Thr	14.2
77	p.Thr77Arg	3.9
77	p.Thr77Ser	5.1
77	p.Thr77Ile	6.4

77	p.Thr77Met	2.6
77	p.Thr77His	2.8
77	p.Thr77Gln	3.1
77	p.Thr77Pro	6.2
77	p.Thr77Leu	4.6
77	p.Thr77Asp	2.4
77	p.Thr77Glu	4.2
77	p.Thr77Ala	5.5
77	p.Thr77Gly	5.1
77	p.Thr77Val	5.2
77	p.Thr77Tyr	3.1
77	p.Thr77Cys	5.0
77	p.Thr77Trp	2.6
77	p.Thr77Phe	2.1
78	p.Leu78Asn	5.4
78	p.Leu78Lys	6.5
78	p.Leu78Thr	4.0
78	p.Leu78Arg	4.1
78	p.Leu78Ser	2.9
78	p.Leu78Ile	6.4
78	p.Leu78Met	9.5
78	p.Leu78His	5.5
78	p.Leu78Gln	7.0
78	p.Leu78Pro	4.4
78	p.Leu78Leu	6.1
78	p.Leu78Asp	8.3
78	p.Leu78Glu	4.1
78	p.Leu78Ala	1.8
78	p.Leu78Gly	1.7
78	p.Leu78Val	5.8
78	p.Leu78Tyr	4.6
78	p.Leu78Cys	3.8
78	p.Leu78Trp	3.9
78	p.Leu78Phe	4.2
79	p.Thr79Asn	6.1
79	p.Thr79Lys	5.0
79	p.Thr79Thr	8.2
79	p.Thr79Arg	2.9
79	p.Thr79Ser	3.8
79	p.Thr79Ile	4.1
79	p.Thr79Met	5.8
79	p.Thr79His	5.2
79	p.Thr79Gln	4.6
79	p.Thr79Pro	3.8
79	p.Thr79Leu	4.7

79	p.Thr79Asp	3.8
79	p.Thr79Glu	3.7
79	p.Thr79Ala	5.6
79	p.Thr79Gly	6.7
79	p.Thr79Val	6.8
79	p.Thr79Tyr	2.9
79	p.Thr79Cys	5.8
79	p.Thr79Trp	6.1
79	p.Thr79Phe	4.5
80	p.Arg80Asn	4.5
80	p.Arg80Lys	5.3
80	p.Arg80Thr	2.6
80	p.Arg80Arg	12.6
80	p.Arg80Ser	9.9
80	p.Arg80Ile	3.3
80	p.Arg80Met	4.0
80	p.Arg80His	3.8
80	p.Arg80Gln	4.3
80	p.Arg80Pro	4.1
80	p.Arg80Leu	4.5
80	p.Arg80Asp	5.3
80	p.Arg80Glu	4.2
80	p.Arg80Ala	4.4
80	p.Arg80Gly	5.1
80	p.Arg80Val	4.4
80	p.Arg80Tyr	5.2
80	p.Arg80Cys	3.4
80	p.Arg80Trp	5.8
80	p.Arg80Phe	3.4
81	p.Pro81Asn	5.8
81	p.Pro81Lys	3.9
81	p.Pro81Thr	5.0
81	p.Pro81Arg	5.7
81	p.Pro81Ser	5.2
81	p.Pro81Ile	3.1
81	p.Pro81Met	4.5
81	p.Pro81His	5.3
81	p.Pro81Gln	5.0
81	p.Pro81Pro	9.4
81	p.Pro81Leu	3.8
81	p.Pro81Asp	3.8
81	p.Pro81Glu	4.8
81	p.Pro81Ala	4.7
81	p.Pro81Gly	3.5
81	p.Pro81Val	4.4

81	p.Pro81Tyr	5.0
81	p.Pro81Cys	5.9
81	p.Pro81Trp	5.7
81	p.Pro81Phe	5.4
82	p.Val82Asn	3.3
82	p.Val82Lys	4.1
82	p.Val82Thr	6.5
82	p.Val82Arg	5.4
82	p.Val82Ser	4.5
82	p.Val82Ile	4.1
82	p.Val82Met	4.4
82	p.Val82His	5.5
82	p.Val82Gln	5.2
82	p.Val82Pro	4.7
82	p.Val82Leu	6.0
82	p.Val82Asp	3.8
82	p.Val82Glu	4.5
82	p.Val82Ala	5.4
82	p.Val82Gly	4.0
82	p.Val82Val	11.1
82	p.Val82Tyr	3.2
82	p.Val82Cys	4.6
82	p.Val82Trp	3.2
82	p.Val82Phe	6.4
83	p.His83Asn	5.4
83	p.His83Lys	5.1
83	p.His83Thr	3.7
83	p.His83Arg	4.3
83	p.His83Ser	4.5
83	p.His83Ile	4.1
83	p.His83Met	4.1
83	p.His83His	9.3
83	p.His83Gln	8.1
83	p.His83Pro	4.7
83	p.His83Leu	4.4
83	p.His83Asp	4.2
83	p.His83Glu	5.1
83	p.His83Ala	4.8
83	p.His83Gly	5.9
83	p.His83Val	3.6
83	p.His83Tyr	4.9
83	p.His83Cys	3.6
83	p.His83Trp	4.1
83	p.His83Phe	5.9
84	p.Asp84Asn	2.9

84	p.Asp84Lys	5.2
84	p.Asp84Thr	3.1
84	p.Asp84Arg	4.6
84	p.Asp84Ser	4.3
84	p.Asp84Ile	4.8
84	p.Asp84Met	4.2
84	p.Asp84His	4.9
84	p.Asp84Gln	4.3
84	p.Asp84Pro	4.9
84	p.Asp84Leu	5.3
84	p.Asp84Asp	7.6
84	p.Asp84Glu	9.1
84	p.Asp84Ala	5.6
84	p.Asp84Gly	4.3
84	p.Asp84Val	4.4
84	p.Asp84Tyr	5.0
84	p.Asp84Cys	5.6
84	p.Asp84Trp	4.5
84	p.Asp84Phe	5.1
85	p.Ala85Asn	3.7
85	p.Ala85Lys	4.0
85	p.Ala85Thr	7.2
85	p.Ala85Arg	2.8
85	p.Ala85Ser	4.3
85	p.Ala85Ile	4.5
85	p.Ala85Met	2.7
85	p.Ala85His	3.9
85	p.Ala85Gln	3.0
85	p.Ala85Pro	6.6
85	p.Ala85Leu	4.6
85	p.Ala85Asp	10.4
85	p.Ala85Glu	4.3
85	p.Ala85Ala	10.3
85	p.Ala85Gly	7.5
85	p.Ala85Val	5.3
85	p.Ala85Tyr	2.7
85	p.Ala85Cys	4.0
85	p.Ala85Trp	3.3
85	p.Ala85Phe	4.9
86	p.Ala86Asn	3.9
86	p.Ala86Lys	3.0
86	p.Ala86Thr	2.2
86	p.Ala86Arg	4.7
86	p.Ala86Ser	3.1
86	p.Ala86Ile	4.8

86	p.Ala86Met	4.9
86	p.Ala86His	5.7
86	p.Ala86Gln	6.9
86	p.Ala86Pro	4.2
86	p.Ala86Leu	6.0
86	p.Ala86Asp	4.5
86	p.Ala86Glu	13.1
86	p.Ala86Ala	4.4
86	p.Ala86Gly	3.4
86	p.Ala86Val	7.9
86	p.Ala86Tyr	4.1
86	p.Ala86Cys	4.1
86	p.Ala86Trp	3.8
86	p.Ala86Phe	5.1
87	p.Arg87Asn	6.8
87	p.Arg87Lys	3.4
87	p.Arg87Thr	3.8
87	p.Arg87Arg	5.1
87	p.Arg87Ser	4.5
87	p.Arg87Ile	4.3
87	p.Arg87Met	6.0
87	p.Arg87His	4.2
87	p.Arg87Gln	5.5
87	p.Arg87Pro	4.9
87	p.Arg87Leu	4.2
87	p.Arg87Asp	3.6
87	p.Arg87Glu	5.9
87	p.Arg87Ala	4.9
87	p.Arg87Gly	4.0
87	p.Arg87Val	5.1
87	p.Arg87Tyr	3.4
87	p.Arg87Cys	6.7
87	p.Arg87Trp	6.6
87	p.Arg87Phe	7.2
88	p.Glu88Asn	4.6
88	p.Glu88Lys	5.3
88	p.Glu88Thr	2.7
88	p.Glu88Arg	4.5
88	p.Glu88Ser	3.3
88	p.Glu88Ile	3.1
88	p.Glu88Met	5.8
88	p.Glu88His	4.2
88	p.Glu88Gln	3.8
88	p.Glu88Pro	3.3
88	p.Glu88Leu	4.3

88	p.Glu88Asp	11.0
88	p.Glu88Glu	8.4
88	p.Glu88Ala	3.1
88	p.Glu88Gly	4.5
88	p.Glu88Val	7.8
88	p.Glu88Tyr	5.9
88	p.Glu88Cys	4.6
88	p.Glu88Trp	5.6
88	p.Glu88Phe	4.3
89	p.Gly89Asn	4.0
89	p.Gly89Lys	5.3
89	p.Gly89Thr	4.1
89	p.Gly89Arg	4.5
89	p.Gly89Ser	5.0
89	p.Gly89Ile	4.1
89	p.Gly89Met	4.3
89	p.Gly89His	4.1
89	p.Gly89Gln	5.1
89	p.Gly89Pro	4.8
89	p.Gly89Leu	4.5
89	p.Gly89Asp	5.2
89	p.Gly89Glu	6.9
89	p.Gly89Ala	3.6
89	p.Gly89Gly	4.9
89	p.Gly89Val	7.7
89	p.Gly89Tyr	3.8
89	p.Gly89Cys	5.0
89	p.Gly89Trp	7.4
89	p.Gly89Phe	5.7
90	p.Phe90Asn	7.3
90	p.Phe90Lys	5.6
90	p.Phe90Thr	4.0
90	p.Phe90Arg	5.8
90	p.Phe90Ser	5.0
90	p.Phe90Ile	4.3
90	p.Phe90Met	4.1
90	p.Phe90His	3.0
90	p.Phe90Gln	3.3
90	p.Phe90Pro	4.8
90	p.Phe90Leu	4.8
90	p.Phe90Asp	4.3
90	p.Phe90Glu	5.4
90	p.Phe90Ala	3.0
90	p.Phe90Gly	4.7
90	p.Phe90Val	5.4

90	p.Phe90Tyr	5.4
90	p.Phe90Cys	5.8
90	p.Phe90Trp	5.3
90	p.Phe90Phe	8.8
91	p.Leu91Asn	4.1
91	p.Leu91Lys	3.3
91	p.Leu91Thr	3.6
91	p.Leu91Arg	4.7
91	p.Leu91Ser	4.5
91	p.Leu91Ile	9.0
91	p.Leu91Met	4.8
91	p.Leu91His	5.7
91	p.Leu91Gln	3.2
91	p.Leu91Pro	11.3
91	p.Leu91Leu	6.3
91	p.Leu91Asp	3.3
91	p.Leu91Glu	4.1
91	p.Leu91Ala	4.1
91	p.Leu91Gly	4.0
91	p.Leu91Val	4.2
91	p.Leu91Tyr	3.2
91	p.Leu91Cys	3.7
91	p.Leu91Trp	4.4
91	p.Leu91Phe	8.4
92	p.Asp92Asn	3.7
92	p.Asp92Lys	3.8
92	p.Asp92Thr	3.8
92	p.Asp92Arg	3.5
92	p.Asp92Ser	2.7
92	p.Asp92Ile	5.8
92	p.Asp92Met	6.3
92	p.Asp92His	2.8
92	p.Asp92Gln	5.5
92	p.Asp92Pro	5.5
92	p.Asp92Leu	5.3
92	p.Asp92Asp	6.8
92	p.Asp92Glu	8.1
92	p.Asp92Ala	5.8
92	p.Asp92Gly	5.0
92	p.Asp92Val	7.3
92	p.Asp92Tyr	3.8
92	p.Asp92Cys	6.2
92	p.Asp92Trp	3.6
92	p.Asp92Phe	4.8
93	p.Thr93Asn	9.2

93	p.Thr93Lys	3.1
93	p.Thr93Thr	13.5
93	p.Thr93Arg	4.0
93	p.Thr93Ser	3.7
93	p.Thr93Ile	6.2
93	p.Thr93Met	3.8
93	p.Thr93His	4.1
93	p.Thr93Gln	3.9
93	p.Thr93Pro	10.3
93	p.Thr93Leu	3.1
93	p.Thr93Asp	2.8
93	p.Thr93Glu	1.6
93	p.Thr93Ala	6.5
93	p.Thr93Gly	4.9
93	p.Thr93Val	3.3
93	p.Thr93Tyr	3.8
93	p.Thr93Cys	4.3
93	p.Thr93Trp	3.7
93	p.Thr93Phe	4.2
94	p.Leu94Asn	3.7
94	p.Leu94Lys	5.7
94	p.Leu94Thr	5.4
94	p.Leu94Arg	6.4
94	p.Leu94Ser	4.1
94	p.Leu94Ile	4.5
94	p.Leu94Met	2.1
94	p.Leu94His	5.0
94	p.Leu94Gln	3.8
94	p.Leu94Pro	3.7
94	p.Leu94Leu	10.6
94	p.Leu94Asp	4.9
94	p.Leu94Glu	3.8
94	p.Leu94Ala	3.7
94	p.Leu94Gly	3.2
94	p.Leu94Val	6.1
94	p.Leu94Tyr	5.0
94	p.Leu94Cys	5.2
94	p.Leu94Trp	6.6
94	p.Leu94Phe	6.5
95	p.Val95Asn	3.9
95	p.Val95Lys	4.7
95	p.Val95Thr	3.7
95	p.Val95Arg	5.1
95	p.Val95Ser	4.4
95	p.Val95Ile	8.2

95	p.Val95Met	2.8
95	p.Val95His	0.8
95	p.Val95Gln	1.5
95	p.Val95Pro	3.1
95	p.Val95Leu	2.8
95	p.Val95Asp	5.7
95	p.Val95Glu	4.6
95	p.Val95Ala	5.3
95	p.Val95Gly	8.5
95	p.Val95Val	8.5
95	p.Val95Tyr	2.7
95	p.Val95Cys	3.2
95	p.Val95Trp	4.0
95	p.Val95Phe	16.5
96	p.Val96Asn	4.0
96	p.Val96Lys	4.5
96	p.Val96Thr	2.1
96	p.Val96Arg	3.5
96	p.Val96Ser	4.4
96	p.Val96Ile	7.2
96	p.Val96Met	3.4
96	p.Val96His	3.1
96	p.Val96Gln	3.9
96	p.Val96Pro	2.2
96	p.Val96Leu	4.6
96	p.Val96Asp	7.2
96	p.Val96Glu	3.8
96	p.Val96Ala	6.9
96	p.Val96Gly	11.5
96	p.Val96Val	5.3
96	p.Val96Tyr	3.0
96	p.Val96Cys	3.8
96	p.Val96Trp	4.2
96	p.Val96Phe	11.5
97	p.Leu97Asn	4.3
97	p.Leu97Lys	5.7
97	p.Leu97Thr	3.2
97	p.Leu97Arg	3.8
97	p.Leu97Ser	5.7
97	p.Leu97Ile	5.2
97	p.Leu97Met	5.4
97	p.Leu97His	3.5
97	p.Leu97Gln	5.5
97	p.Leu97Pro	3.4
97	p.Leu97Leu	7.2

97	p.Leu97Asp	5.1
97	p.Leu97Glu	6.0
97	p.Leu97Ala	3.9
97	p.Leu97Gly	5.7
97	p.Leu97Val	5.3
97	p.Leu97Tyr	4.4
97	p.Leu97Cys	4.6
97	p.Leu97Trp	6.6
97	p.Leu97Phe	5.3
98	p.His98Asn	4.1
98	p.His98Lys	4.1
98	p.His98Thr	2.3
98	p.His98Arg	4.2
98	p.His98Ser	4.7
98	p.His98Ile	3.8
98	p.His98Met	4.4
98	p.His98His	7.3
98	p.His98Gln	12.0
98	p.His98Pro	3.1
98	p.His98Leu	4.1
98	p.His98Asp	4.7
98	p.His98Glu	6.5
98	p.His98Ala	4.0
98	p.His98Gly	5.0
98	p.His98Val	6.5
98	p.His98Tyr	6.0
98	p.His98Cys	4.0
98	p.His98Trp	5.3
98	p.His98Phe	4.1
99	p.Arg99Asn	4.2
99	p.Arg99Lys	4.3
99	p.Arg99Thr	4.5
99	p.Arg99Arg	8.9
99	p.Arg99Ser	5.7
99	p.Arg99Ile	5.4
99	p.Arg99Met	4.2
99	p.Arg99His	4.8
99	p.Arg99Gln	3.0
99	p.Arg99Pro	5.1
99	p.Arg99Leu	3.9
99	p.Arg99Asp	4.9
99	p.Arg99Glu	5.0
99	p.Arg99Ala	6.2
99	p.Arg99Gly	4.9
99	p.Arg99Val	4.0

99	p.Arg99Tyr	4.2
99	p.Arg99Cys	5.4
99	p.Arg99Trp	6.9
99	p.Arg99Phe	4.5
100	p.Ala100Asn	6.8
100	p.Ala100Lys	8.6
100	p.Ala100Thr	6.2
100	p.Ala100Arg	8.2
100	p.Ala100Ser	2.7
100	p.Ala100Ile	5.8
100	p.Ala100Met	8.7
100	p.Ala100His	5.3
100	p.Ala100Gln	4.4
100	p.Ala100Pro	4.7
100	p.Ala100Leu	4.9
100	p.Ala100Asp	1.2
100	p.Ala100Glu	4.6
100	p.Ala100Ala	4.1
100	p.Ala100Gly	0.2
100	p.Ala100Val	5.4
100	p.Ala100Tyr	4.6
100	p.Ala100Cys	3.5
100	p.Ala100Trp	6.8
100	p.Ala100Phe	3.4
101	p.Gly101Asn	4.1
101	p.Gly101Lys	5.8
101	p.Gly101Thr	4.4
101	p.Gly101Arg	5.2
101	p.Gly101Ser	5.8
101	p.Gly101Ile	4.4
101	p.Gly101Met	5.9
101	p.Gly101His	3.6
101	p.Gly101Gln	5.3
101	p.Gly101Pro	3.7
101	p.Gly101Leu	4.7
101	p.Gly101Asp	5.5
101	p.Gly101Glu	4.8
101	p.Gly101Ala	3.4
101	p.Gly101Gly	5.7
101	p.Gly101Val	4.7
101	p.Gly101Tyr	5.2
101	p.Gly101Cys	7.0
101	p.Gly101Trp	5.5
101	p.Gly101Phe	5.4
102	p.Ala102Asn	2.4

102	p.Ala102Lys	5.0
102	p.Ala102Thr	5.3
102	p.Ala102Arg	4.5
102	p.Ala102Ser	2.1
102	p.Ala102Ile	4.3
102	p.Ala102Met	4.2
102	p.Ala102His	3.5
102	p.Ala102Gln	5.5
102	p.Ala102Pro	3.3
102	p.Ala102Leu	6.0
102	p.Ala102Asp	11.0
102	p.Ala102Glu	6.8
102	p.Ala102Ala	9.3
102	p.Ala102Gly	5.1
102	p.Ala102Val	7.2
102	p.Ala102Tyr	2.3
102	p.Ala102Cys	2.4
102	p.Ala102Trp	7.6
102	p.Ala102Phe	2.2
103	p.Arg103Asn	8.0
103	p.Arg103Lys	0.1
103	p.Arg103Thr	4.6
103	p.Arg103Arg	2.6
103	p.Arg103Ser	9.1
103	p.Arg103Ile	4.7
103	p.Arg103Met	2.0
103	p.Arg103His	4.9
103	p.Arg103Gln	1.0
103	p.Arg103Pro	2.7
103	p.Arg103Leu	0.8
103	p.Arg103Asp	5.3
103	p.Arg103Glu	1.1
103	p.Arg103Ala	5.8
103	p.Arg103Gly	10.5
103	p.Arg103Val	2.1
103	p.Arg103Tyr	5.4
103	p.Arg103Cys	16.6
103	p.Arg103Trp	3.4
103	p.Arg103Phe	9.3
104	p.Leu104Asn	3.4
104	p.Leu104Lys	3.5
104	p.Leu104Thr	3.0
104	p.Leu104Arg	4.5
104	p.Leu104Ser	4.1
104	p.Leu104Ile	13.2

104	p.Leu104Met	5.2
104	p.Leu104His	7.3
104	p.Leu104Gln	2.6
104	p.Leu104Pro	6.2
104	p.Leu104Leu	7.4
104	p.Leu104Asp	3.1
104	p.Leu104Glu	5.1
104	p.Leu104Ala	3.4
104	p.Leu104Gly	4.2
104	p.Leu104Val	5.6
104	p.Leu104Tyr	3.2
104	p.Leu104Cys	3.8
104	p.Leu104Trp	3.8
104	p.Leu104Phe	7.4
105	p.Asp105Asn	6.6
105	p.Asp105Lys	6.5
105	p.Asp105Thr	4.6
105	p.Asp105Arg	6.5
105	p.Asp105Ser	1.6
105	p.Asp105Ile	3.4
105	p.Asp105Met	2.4
105	p.Asp105His	4.7
105	p.Asp105Gln	5.3
105	p.Asp105Pro	2.9
105	p.Asp105Leu	5.0
105	p.Asp105Asp	6.7
105	p.Asp105Glu	8.3
105	p.Asp105Ala	4.9
105	p.Asp105Gly	1.9
105	p.Asp105Val	7.3
105	p.Asp105Tyr	6.5
105	p.Asp105Cys	2.1
105	p.Asp105Trp	6.7
105	p.Asp105Phe	6.1
106	p.Val106Asn	3.8
106	p.Val106Lys	4.2
106	p.Val106Thr	3.5
106	p.Val106Arg	2.0
106	p.Val106Ser	4.4
106	p.Val106Ile	7.2
106	p.Val106Met	4.4
106	p.Val106His	4.0
106	p.Val106Gln	3.8
106	p.Val106Pro	2.9
106	p.Val106Leu	2.4

106	p.Val106Asp	7.5
106	p.Val106Glu	1.4
106	p.Val106Ala	8.6
106	p.Val106Gly	12.1
106	p.Val106Val	5.8
106	p.Val106Tyr	3.5
106	p.Val106Cys	5.5
106	p.Val106Trp	1.4
106	p.Val106Phe	11.7
107	p.Arg107Asn	3.2
107	p.Arg107Lys	3.2
107	p.Arg107Thr	4.9
107	p.Arg107Arg	4.6
107	p.Arg107Ser	3.7
107	p.Arg107Ile	2.4
107	p.Arg107Met	5.3
107	p.Arg107His	3.4
107	p.Arg107Gln	11.5
107	p.Arg107Pro	5.1
107	p.Arg107Leu	11.5
107	p.Arg107Asp	3.2
107	p.Arg107Glu	2.6
107	p.Arg107Ala	5.9
107	p.Arg107Gly	3.7
107	p.Arg107Val	5.4
107	p.Arg107Tyr	4.0
107	p.Arg107Cys	2.1
107	p.Arg107Trp	10.2
107	p.Arg107Phe	4.1
108	p.Asp108Asn	6.3
108	p.Asp108Lys	4.5
108	p.Asp108Thr	5.2
108	p.Asp108Arg	6.4
108	p.Asp108Ser	6.5
108	p.Asp108Ile	4.4
108	p.Asp108Met	4.9
108	p.Asp108His	3.7
108	p.Asp108Gln	3.1
108	p.Asp108Pro	4.1
108	p.Asp108Leu	3.0
108	p.Asp108Asp	5.1
108	p.Asp108Glu	8.1
108	p.Asp108Ala	3.2
108	p.Asp108Gly	5.2
108	p.Asp108Val	4.9

108	p.Asp108Tyr	7.3
108	p.Asp108Cys	4.3
108	p.Asp108Trp	4.4
108	p.Asp108Phe	5.3
109	p.Ala109Asn	4.9
109	p.Ala109Lys	6.3
109	p.Ala109Thr	4.3
109	p.Ala109Arg	5.2
109	p.Ala109Ser	6.7
109	p.Ala109Ile	3.1
109	p.Ala109Met	5.1
109	p.Ala109His	5.4
109	p.Ala109Gln	5.8
109	p.Ala109Pro	6.2
109	p.Ala109Leu	4.9
109	p.Ala109Asp	0.8
109	p.Ala109Glu	5.8
109	p.Ala109Ala	7.3
109	p.Ala109Gly	3.7
109	p.Ala109Val	4.6
109	p.Ala109Tyr	3.3
109	p.Ala109Cys	5.2
109	p.Ala109Trp	6.3
109	p.Ala109Phe	5.0
110	p.Trp110Asn	4.5
110	p.Trp110Lys	5.8
110	p.Trp110Thr	5.0
110	p.Trp110Arg	4.5
110	p.Trp110Ser	7.0
110	p.Trp110Ile	8.0
110	p.Trp110Met	4.6
110	p.Trp110His	2.4
110	p.Trp110Gln	5.1
110	p.Trp110Pro	0.3
110	p.Trp110Leu	5.8
110	p.Trp110Asp	4.4
110	p.Trp110Glu	6.4
110	p.Trp110Ala	3.3
110	p.Trp110Gly	5.1
110	p.Trp110Val	10.8
110	p.Trp110Tyr	6.3
110	p.Trp110Cys	5.6
110	p.Trp110Trp	0.0
110	p.Trp110Phe	5.0
111	p.Gly111Asn	5.5

111	p.Gly111Lys	5.9
111	p.Gly111Thr	2.4
111	p.Gly111Arg	8.1
111	p.Gly111Ser	5.6
111	p.Gly111Ile	3.5
111	p.Gly111Met	4.2
111	p.Gly111His	4.6
111	p.Gly111Gln	4.2
111	p.Gly111Pro	3.0
111	p.Gly111Leu	3.3
111	p.Gly111Asp	4.7
111	p.Gly111Glu	6.5
111	p.Gly111Ala	3.0
111	p.Gly111Gly	8.7
111	p.Gly111Val	5.1
111	p.Gly111Tyr	3.7
111	p.Gly111Cys	7.0
111	p.Gly111Trp	6.1
111	p.Gly111Phe	4.9
112	p.Arg112Asn	5.7
112	p.Arg112Lys	4.7
112	p.Arg112Thr	4.8
112	p.Arg112Arg	4.2
112	p.Arg112Ser	4.9
112	p.Arg112Ile	5.0
112	p.Arg112Met	4.7
112	p.Arg112His	4.3
112	p.Arg112Gln	8.9
112	p.Arg112Pro	4.1
112	p.Arg112Leu	6.5
112	p.Arg112Asp	3.9
112	p.Arg112Glu	4.7
112	p.Arg112Ala	5.2
112	p.Arg112Gly	4.2
112	p.Arg112Val	4.5
112	p.Arg112Tyr	4.9
112	p.Arg112Cys	2.7
112	p.Arg112Trp	7.4
112	p.Arg112Phe	4.6
113	p.Leu113Asn	2.7
113	p.Leu113Lys	4.3
113	p.Leu113Thr	4.2
113	p.Leu113Arg	2.2
113	p.Leu113Ser	3.8
113	p.Leu113Ile	8.8

113	p.Leu113Met	2.6
113	p.Leu113His	8.3
113	p.Leu113Gln	3.1
113	p.Leu113Pro	22.7
113	p.Leu113Leu	5.3
113	p.Leu113Asp	3.7
113	p.Leu113Glu	3.4
113	p.Leu113Ala	2.7
113	p.Leu113Gly	3.6
113	p.Leu113Val	4.5
113	p.Leu113Tyr	1.8
113	p.Leu113Cys	3.3
113	p.Leu113Trp	3.2
113	p.Leu113Phe	5.7
114	p.Pro114Asn	5.1
114	p.Pro114Lys	3.6
114	p.Pro114Thr	3.7
114	p.Pro114Arg	5.1
114	p.Pro114Ser	4.7
114	p.Pro114Ile	3.6
114	p.Pro114Met	3.4
114	p.Pro114His	4.4
114	p.Pro114Gln	6.1
114	p.Pro114Pro	6.5
114	p.Pro114Leu	5.1
114	p.Pro114Asp	6.6
114	p.Pro114Glu	5.1
114	p.Pro114Ala	4.1
114	p.Pro114Gly	5.2
114	p.Pro114Val	5.7
114	p.Pro114Tyr	5.1
114	p.Pro114Cys	5.0
114	p.Pro114Trp	6.7
114	p.Pro114Phe	5.4
115	p.Val115Asn	4.1
115	p.Val115Lys	3.0
115	p.Val115Thr	3.8
115	p.Val115Arg	2.9
115	p.Val115Ser	4.0
115	p.Val115Ile	4.8
115	p.Val115Met	4.8
115	p.Val115His	3.7
115	p.Val115Gln	4.2
115	p.Val115Pro	5.1
115	p.Val115Leu	6.1

115	p.Val115Asp	6.8
115	p.Val115Glu	3.7
115	p.Val115Ala	6.5
115	p.Val115Gly	12.5
115	p.Val115Val	7.0
115	p.Val115Tyr	2.7
115	p.Val115Cys	0.4
115	p.Val115Trp	4.0
115	p.Val115Phe	9.9
116	p.Asp116Asn	3.2
116	p.Asp116Lys	5.9
116	p.Asp116Thr	3.0
116	p.Asp116Arg	3.8
116	p.Asp116Ser	4.8
116	p.Asp116Ile	5.0
116	p.Asp116Met	3.8
116	p.Asp116His	5.3
116	p.Asp116Gln	3.7
116	p.Asp116Pro	2.2
116	p.Asp116Leu	4.5
116	p.Asp116Asp	9.8
116	p.Asp116Glu	14.4
116	p.Asp116Ala	3.8
116	p.Asp116Gly	5.1
116	p.Asp116Val	4.2
116	p.Asp116Tyr	4.1
116	p.Asp116Cys	5.6
116	p.Asp116Trp	3.4
116	p.Asp116Phe	4.3
117	p.Leu117Asn	4.0
117	p.Leu117Lys	5.6
117	p.Leu117Thr	3.9
117	p.Leu117Arg	4.1
117	p.Leu117Ser	4.4
117	p.Leu117Ile	10.9
117	p.Leu117Met	4.8
117	p.Leu117His	4.9
117	p.Leu117Gln	4.5
117	p.Leu117Pro	7.6
117	p.Leu117Leu	7.0
117	p.Leu117Asp	3.9
117	p.Leu117Glu	4.5
117	p.Leu117Ala	1.9
117	p.Leu117Gly	4.5
117	p.Leu117Val	4.4

117	p.Leu117Tyr	4.7
117	p.Leu117Cys	3.8
117	p.Leu117Trp	3.5
117	p.Leu117Phe	7.0
118	p.Ala118Asn	5.1
118	p.Ala118Lys	4.1
118	p.Ala118Thr	4.6
118	p.Ala118Arg	6.0
118	p.Ala118Ser	4.1
118	p.Ala118Ile	4.4
118	p.Ala118Met	4.7
118	p.Ala118His	3.2
118	p.Ala118Gln	5.0
118	p.Ala118Pro	4.0
118	p.Ala118Leu	5.2
118	p.Ala118Asp	5.7
118	p.Ala118Glu	3.7
118	p.Ala118Ala	12.8
118	p.Ala118Gly	4.6
118	p.Ala118Val	5.7
118	p.Ala118Tyr	3.7
118	p.Ala118Cys	5.4
118	p.Ala118Trp	3.9
118	p.Ala118Phe	3.9
119	p.Glu119Asn	4.3
119	p.Glu119Lys	4.5
119	p.Glu119Thr	5.3
119	p.Glu119Arg	4.6
119	p.Glu119Ser	4.4
119	p.Glu119Ile	3.7
119	p.Glu119Met	5.7
119	p.Glu119His	5.4
119	p.Glu119Gln	3.5
119	p.Glu119Pro	4.6
119	p.Glu119Leu	4.6
119	p.Glu119Asp	9.8
119	p.Glu119Glu	8.5
119	p.Glu119Ala	4.0
119	p.Glu119Gly	5.3
119	p.Glu119Val	5.1
119	p.Glu119Tyr	4.2
119	p.Glu119Cys	5.1
119	p.Glu119Trp	3.9
119	p.Glu119Phe	3.8
120	p.Glu120Asn	7.3

120	p.Glu120Lys	5.0
120	p.Glu120Thr	5.4
120	p.Glu120Arg	3.2
120	p.Glu120Ser	4.0
120	p.Glu120Ile	4.6
120	p.Glu120Met	3.7
120	p.Glu120His	5.2
120	p.Glu120Gln	5.0
120	p.Glu120Pro	3.7
120	p.Glu120Leu	3.6
120	p.Glu120Asp	10.2
120	p.Glu120Glu	8.7
120	p.Glu120Ala	3.5
120	p.Glu120Gly	2.9
120	p.Glu120Val	5.1
120	p.Glu120Tyr	2.7
120	p.Glu120Cys	4.7
120	p.Glu120Trp	5.0
120	p.Glu120Phe	6.4
121	p.Leu121Asn	3.6
121	p.Leu121Lys	4.2
121	p.Leu121Thr	4.8
121	p.Leu121Arg	4.2
121	p.Leu121Ser	3.9
121	p.Leu121Ile	4.5
121	p.Leu121Met	5.4
121	p.Leu121His	4.0
121	p.Leu121Gln	5.1
121	p.Leu121Pro	4.4
121	p.Leu121Leu	10.1
121	p.Leu121Asp	5.7
121	p.Leu121Glu	5.6
121	p.Leu121Ala	3.1
121	p.Leu121Gly	6.1
121	p.Leu121Val	6.2
121	p.Leu121Tyr	2.9
121	p.Leu121Cys	4.6
121	p.Leu121Trp	4.8
121	p.Leu121Phe	6.7
122	p.Gly122Asn	4.3
122	p.Gly122Lys	5.0
122	p.Gly122Thr	5.6
122	p.Gly122Arg	5.3
122	p.Gly122Ser	5.4
122	p.Gly122Ile	4.3

122	p.Gly122Met	4.8
122	p.Gly122His	4.6
122	p.Gly122Gln	3.9
122	p.Gly122Pro	3.7
122	p.Gly122Leu	3.9
122	p.Gly122Asp	5.0
122	p.Gly122Glu	7.0
122	p.Gly122Ala	5.4
122	p.Gly122Gly	4.9
122	p.Gly122Val	6.2
122	p.Gly122Tyr	5.5
122	p.Gly122Cys	3.8
122	p.Gly122Trp	7.1
122	p.Gly122Phe	4.4
123	p.His123Asn	11.7
123	p.His123Lys	5.8
123	p.His123Thr	4.1
123	p.His123Arg	3.5
123	p.His123Ser	4.0
123	p.His123Ile	4.4
123	p.His123Met	5.1
123	p.His123His	3.8
123	p.His123Gln	5.8
123	p.His123Pro	5.0
123	p.His123Leu	4.1
123	p.His123Asp	5.1
123	p.His123Glu	5.3
123	p.His123Ala	3.7
123	p.His123Gly	3.1
123	p.His123Val	4.5
123	p.His123Tyr	6.1
123	p.His123Cys	5.4
123	p.His123Trp	5.3
123	p.His123Phe	4.0
124	p.Arg124Asn	3.9
124	p.Arg124Lys	6.7
124	p.Arg124Thr	3.7
124	p.Arg124Arg	8.4
124	p.Arg124Ser	5.4
124	p.Arg124Ile	4.7
124	p.Arg124Met	6.9
124	p.Arg124His	4.2
124	p.Arg124Gln	4.4
124	p.Arg124Pro	4.2
124	p.Arg124Leu	5.5

124	p.Arg124Asp	3.7
124	p.Arg124Glu	5.3
124	p.Arg124Ala	4.4
124	p.Arg124Gly	3.4
124	p.Arg124Val	5.0
124	p.Arg124Tyr	4.2
124	p.Arg124Cys	4.2
124	p.Arg124Trp	6.7
124	p.Arg124Phe	5.3
125	p.Asp125Asn	6.5
125	p.Asp125Lys	5.1
125	p.Asp125Thr	5.4
125	p.Asp125Arg	4.7
125	p.Asp125Ser	4.0
125	p.Asp125Ile	4.5
125	p.Asp125Met	4.4
125	p.Asp125His	4.8
125	p.Asp125Gln	4.2
125	p.Asp125Pro	2.1
125	p.Asp125Leu	3.4
125	p.Asp125Asp	5.0
125	p.Asp125Glu	6.8
125	p.Asp125Ala	6.6
125	p.Asp125Gly	7.1
125	p.Asp125Val	4.7
125	p.Asp125Tyr	6.7
125	p.Asp125Cys	4.6
125	p.Asp125Trp	4.7
125	p.Asp125Phe	4.7
126	p.Val126Asn	4.2
126	p.Val126Lys	4.8
126	p.Val126Thr	3.8
126	p.Val126Arg	6.4
126	p.Val126Ser	4.6
126	p.Val126Ile	6.3
126	p.Val126Met	7.2
126	p.Val126His	4.9
126	p.Val126Gln	6.0
126	p.Val126Pro	5.2
126	p.Val126Leu	4.1
126	p.Val126Asp	3.5
126	p.Val126Glu	5.1
126	p.Val126Ala	4.9
126	p.Val126Gly	4.0
126	p.Val126Val	6.3

126	p.Val126Tyr	4.7
126	p.Val126Cys	3.5
126	p.Val126Trp	5.4
126	p.Val126Phe	5.1
127	p.Ala127Asn	4.3
127	p.Ala127Lys	4.1
127	p.Ala127Thr	5.5
127	p.Ala127Arg	5.0
127	p.Ala127Ser	4.6
127	p.Ala127Ile	4.5
127	p.Ala127Met	5.0
127	p.Ala127His	4.0
127	p.Ala127Gln	4.5
127	p.Ala127Pro	2.9
127	p.Ala127Leu	4.4
127	p.Ala127Asp	7.6
127	p.Ala127Glu	5.2
127	p.Ala127Ala	6.8
127	p.Ala127Gly	5.2
127	p.Ala127Val	6.7
127	p.Ala127Tyr	3.9
127	p.Ala127Cys	4.6
127	p.Ala127Trp	5.6
127	p.Ala127Phe	5.5
128	p.Arg128Asn	4.1
128	p.Arg128Lys	4.8
128	p.Arg128Thr	4.7
128	p.Arg128Arg	5.2
128	p.Arg128Ser	8.5
128	p.Arg128Ile	4.7
128	p.Arg128Met	4.1
128	p.Arg128His	3.8
128	p.Arg128Gln	4.7
128	p.Arg128Pro	4.3
128	p.Arg128Leu	3.0
128	p.Arg128Asp	5.2
128	p.Arg128Glu	7.4
128	p.Arg128Ala	4.5
128	p.Arg128Gly	5.8
128	p.Arg128Val	5.4
128	p.Arg128Tyr	4.6
128	p.Arg128Cys	4.5
128	p.Arg128Trp	5.8
128	p.Arg128Phe	4.8
129	p.Tyr129Asn	3.3

129	p.Tyr129Lys	4.5
129	p.Tyr129Thr	4.0
129	p.Tyr129Arg	4.7
129	p.Tyr129Ser	3.6
129	p.Tyr129Ile	4.4
129	p.Tyr129Met	4.6
129	p.Tyr129His	4.8
129	p.Tyr129Gln	6.6
129	p.Tyr129Pro	4.3
129	p.Tyr129Leu	3.8
129	p.Tyr129Asp	6.2
129	p.Tyr129Glu	4.7
129	p.Tyr129Ala	6.6
129	p.Tyr129Gly	5.0
129	p.Tyr129Val	6.8
129	p.Tyr129Tyr	6.5
129	p.Tyr129Cys	4.3
129	p.Tyr129Trp	5.8
129	p.Tyr129Phe	5.1
130	p.Leu130Asn	2.8
130	p.Leu130Lys	3.3
130	p.Leu130Thr	3.4
130	p.Leu130Arg	3.1
130	p.Leu130Ser	4.8
130	p.Leu130Ile	5.7
130	p.Leu130Met	6.1
130	p.Leu130His	2.7
130	p.Leu130Gln	3.0
130	p.Leu130Pro	2.1
130	p.Leu130Leu	7.5
130	p.Leu130Asp	3.5
130	p.Leu130Glu	4.4
130	p.Leu130Ala	3.2
130	p.Leu130Gly	3.7
130	p.Leu130Val	12.3
130	p.Leu130Tyr	4.1
130	p.Leu130Cys	4.4
130	p.Leu130Trp	11.1
130	p.Leu130Phe	8.8
131	p.Arg131Asn	5.1
131	p.Arg131Lys	5.5
131	p.Arg131Thr	5.3
131	p.Arg131Arg	4.5
131	p.Arg131Ser	4.6
131	p.Arg131Ile	4.8

131	p.Arg131Met	5.0
131	p.Arg131His	3.9
131	p.Arg131Gln	5.7
131	p.Arg131Pro	4.1
131	p.Arg131Leu	4.6
131	p.Arg131Asp	5.9
131	p.Arg131Glu	5.1
131	p.Arg131Ala	5.9
131	p.Arg131Gly	4.6
131	p.Arg131Val	3.6
131	p.Arg131Tyr	4.6
131	p.Arg131Cys	5.3
131	p.Arg131Trp	6.0
131	p.Arg131Phe	5.9
132	p.Ala132Asn	3.4
132	p.Ala132Lys	6.1
132	p.Ala132Thr	1.1
132	p.Ala132Arg	5.9
132	p.Ala132Ser	3.7
132	p.Ala132Ile	3.4
132	p.Ala132Met	4.6
132	p.Ala132His	2.8
132	p.Ala132Gln	8.9
132	p.Ala132Pro	4.9
132	p.Ala132Leu	5.0
132	p.Ala132Asp	4.0
132	p.Ala132Glu	5.0
132	p.Ala132Ala	10.1
132	p.Ala132Gly	3.9
132	p.Ala132Val	7.3
132	p.Ala132Tyr	4.3
132	p.Ala132Cys	5.8
132	p.Ala132Trp	4.4
132	p.Ala132Phe	5.5
133	p.Ala133Asn	4.0
133	p.Ala133Lys	3.7
133	p.Ala133Thr	8.5
133	p.Ala133Arg	4.7
133	p.Ala133Ser	6.6
133	p.Ala133Ile	5.2
133	p.Ala133Met	3.9
133	p.Ala133His	2.1
133	p.Ala133Gln	3.8
133	p.Ala133Pro	8.1
133	p.Ala133Leu	4.4

133	p.Ala133Asp	8.7
133	p.Ala133Glu	4.6
133	p.Ala133Ala	7.7
133	p.Ala133Gly	5.4
133	p.Ala133Val	4.4
133	p.Ala133Tyr	5.4
133	p.Ala133Cys	2.6
133	p.Ala133Trp	2.9
133	p.Ala133Phe	3.3
134	p.Ala134Asn	4.8
134	p.Ala134Lys	2.6
134	p.Ala134Thr	6.5
134	p.Ala134Arg	2.2
134	p.Ala134Ser	4.7
134	p.Ala134Ile	5.1
134	p.Ala134Met	3.9
134	p.Ala134His	4.7
134	p.Ala134Gln	3.6
134	p.Ala134Pro	4.3
134	p.Ala134Leu	4.1
134	p.Ala134Asp	7.9
134	p.Ala134Glu	6.6
134	p.Ala134Ala	6.0
134	p.Ala134Gly	7.5
134	p.Ala134Val	5.9
134	p.Ala134Tyr	4.8
134	p.Ala134Cys	5.7
134	p.Ala134Trp	4.6
134	p.Ala134Phe	4.5
135	p.Gly135Asn	3.9
135	p.Gly135Lys	4.1
135	p.Gly135Thr	3.6
135	p.Gly135Arg	4.9
135	p.Gly135Ser	2.7
135	p.Gly135Ile	3.8
135	p.Gly135Met	6.3
135	p.Gly135His	6.5
135	p.Gly135Gln	4.3
135	p.Gly135Pro	5.1
135	p.Gly135Leu	4.0
135	p.Gly135Asp	3.8
135	p.Gly135Glu	3.6
135	p.Gly135Ala	4.9
135	p.Gly135Gly	10.5
135	p.Gly135Val	5.4

135	p.Gly135Tyr	4.3
135	p.Gly135Cys	5.3
135	p.Gly135Trp	7.6
135	p.Gly135Phe	5.4
136	p.Gly136Asn	6.1
136	p.Gly136Lys	4.0
136	p.Gly136Thr	5.2
136	p.Gly136Arg	5.6
136	p.Gly136Ser	5.2
136	p.Gly136Ile	5.9
136	p.Gly136Met	4.6
136	p.Gly136His	4.1
136	p.Gly136Gln	5.6
136	p.Gly136Pro	5.1
136	p.Gly136Leu	5.2
136	p.Gly136Asp	4.9
136	p.Gly136Glu	6.3
136	p.Gly136Ala	4.6
136	p.Gly136Gly	9.7
136	p.Gly136Val	6.3
136	p.Gly136Tyr	3.0
136	p.Gly136Cys	0.6
136	p.Gly136Trp	3.7
136	p.Gly136Phe	4.5
137	p.Thr137Asn	3.1
137	p.Thr137Lys	22.3
137	p.Thr137Thr	3.7
137	p.Thr137Arg	4.6
137	p.Thr137Ser	4.7
137	p.Thr137Ile	4.2
137	p.Thr137Met	9.2
137	p.Thr137His	3.0
137	p.Thr137Gln	3.1
137	p.Thr137Pro	4.8
137	p.Thr137Leu	4.0
137	p.Thr137Asp	3.5
137	p.Thr137Glu	3.7
137	p.Thr137Ala	3.7
137	p.Thr137Gly	3.2
137	p.Thr137Val	4.7
137	p.Thr137Tyr	3.0
137	p.Thr137Cys	2.6
137	p.Thr137Trp	4.3
137	p.Thr137Phe	4.5
138	p.Arg138Asn	4.3

138	p.Arg138Lys	7.1
138	p.Arg138Thr	4.2
138	p.Arg138Arg	7.9
138	p.Arg138Ser	5.2
138	p.Arg138Ile	4.4
138	p.Arg138Met	14.4
138	p.Arg138His	3.8
138	p.Arg138Gln	4.2
138	p.Arg138Pro	3.2
138	p.Arg138Leu	4.9
138	p.Arg138Asp	2.7
138	p.Arg138Glu	3.6
138	p.Arg138Ala	1.7
138	p.Arg138Gly	2.4
138	p.Arg138Val	3.7
138	p.Arg138Tyr	3.3
138	p.Arg138Cys	3.6
138	p.Arg138Trp	11.1
138	p.Arg138Phe	4.1
139	p.Gly139Asn	4.0
139	p.Gly139Lys	5.1
139	p.Gly139Thr	3.7
139	p.Gly139Arg	7.9
139	p.Gly139Ser	5.5
139	p.Gly139Ile	4.7
139	p.Gly139Met	4.7
139	p.Gly139His	3.8
139	p.Gly139Gln	4.8
139	p.Gly139Pro	4.8
139	p.Gly139Leu	4.9
139	p.Gly139Asp	4.5
139	p.Gly139Glu	5.4
139	p.Gly139Ala	3.9
139	p.Gly139Gly	7.1
139	p.Gly139Val	5.3
139	p.Gly139Tyr	3.7
139	p.Gly139Cys	5.9
139	p.Gly139Trp	5.7
139	p.Gly139Phe	4.4
140	p.Ser140Asn	5.8
140	p.Ser140Lys	4.9
140	p.Ser140Thr	6.5
140	p.Ser140Arg	4.8
140	p.Ser140Ser	3.3
140	p.Ser140Ile	5.3

140	p.Ser140Met	4.5
140	p.Ser140His	5.3
140	p.Ser140Gln	5.1
140	p.Ser140Pro	4.0
140	p.Ser140Leu	4.5
140	p.Ser140Asp	5.7
140	p.Ser140Glu	4.9
140	p.Ser140Ala	3.8
140	p.Ser140Gly	2.9
140	p.Ser140Val	6.5
140	p.Ser140Tyr	5.7
140	p.Ser140Cys	6.0
140	p.Ser140Trp	5.5
140	p.Ser140Phe	5.1
141	p.Asn141Asn	21.3
141	p.Asn141Lys	19.1
141	p.Asn141Thr	2.9
141	p.Asn141Arg	3.6
141	p.Asn141Ser	3.0
141	p.Asn141Ile	3.5
141	p.Asn141Met	4.3
141	p.Asn141His	2.0
141	p.Asn141Gln	3.3
141	p.Asn141Pro	2.1
141	p.Asn141Leu	4.8
141	p.Asn141Asp	2.7
141	p.Asn141Glu	3.6
141	p.Asn141Ala	3.6
141	p.Asn141Gly	3.4
141	p.Asn141Val	4.0
141	p.Asn141Tyr	2.7
141	p.Asn141Cys	2.1
141	p.Asn141Trp	4.1
141	p.Asn141Phe	4.0
142	p.His142Asn	19.9
142	p.His142Lys	2.6
142	p.His142Thr	2.8
142	p.His142Arg	2.4
142	p.His142Ser	1.9
142	p.His142Ile	2.6
142	p.His142Met	1.6
142	p.His142His	5.0
142	p.His142Gln	3.8
142	p.His142Pro	29.0
142	p.His142Leu	3.0

142	p.His142Asp	3.8
142	p.His142Glu	2.8
142	p.His142Ala	2.4
142	p.His142Gly	2.3
142	p.His142Val	2.8
142	p.His142Tyr	4.7
142	p.His142Cys	2.3
142	p.His142Trp	2.4
142	p.His142Phe	1.7
143	p.Ala143Asn	5.2
143	p.Ala143Lys	6.3
143	p.Ala143Thr	5.9
143	p.Ala143Arg	4.3
143	p.Ala143Ser	4.2
143	p.Ala143Ile	4.3
143	p.Ala143Met	4.8
143	p.Ala143His	3.5
143	p.Ala143Gln	5.9
143	p.Ala143Pro	3.4
143	p.Ala143Leu	3.9
143	p.Ala143Asp	4.8
143	p.Ala143Glu	5.4
143	p.Ala143Ala	8.9
143	p.Ala143Gly	4.3
143	p.Ala143Val	4.6
143	p.Ala143Tyr	5.8
143	p.Ala143Cys	4.2
143	p.Ala143Trp	5.0
143	p.Ala143Phe	5.3
144	p.Arg144Asn	6.1
144	p.Arg144Lys	4.1
144	p.Arg144Thr	5.3
144	p.Arg144Arg	5.0
144	p.Arg144Ser	5.4
144	p.Arg144Ile	3.8
144	p.Arg144Met	4.5
144	p.Arg144His	4.0
144	p.Arg144Gln	5.1
144	p.Arg144Pro	3.8
144	p.Arg144Leu	6.4
144	p.Arg144Asp	4.1
144	p.Arg144Glu	5.7
144	p.Arg144Ala	4.7
144	p.Arg144Gly	5.7
144	p.Arg144Val	5.1

144	p.Arg144Tyr	4.9
144	p.Arg144Cys	6.5
144	p.Arg144Trp	4.9
144	p.Arg144Phe	4.9
145	p.Ile145Asn	7.0
145	p.Ile145Lys	2.4
145	p.Ile145Thr	10.7
145	p.Ile145Arg	2.7
145	p.Ile145Ser	21.9
145	p.Ile145Ile	18.9
145	p.Ile145Met	6.5
145	p.Ile145His	1.5
145	p.Ile145Gln	2.2
145	p.Ile145Pro	1.9
145	p.Ile145Leu	2.2
145	p.Ile145Asp	1.8
145	p.Ile145Glu	2.3
145	p.Ile145Ala	1.4
145	p.Ile145Gly	2.0
145	p.Ile145Val	2.0
145	p.Ile145Tyr	1.9
145	p.Ile145Cys	2.0
145	p.Ile145Trp	1.7
145	p.Ile145Phe	7.1
146	p.Asp146Asn	8.5
146	p.Asp146Lys	2.3
146	p.Asp146Thr	2.0
146	p.Asp146Arg	2.7
146	p.Asp146Ser	2.6
146	p.Asp146Ile	1.7
146	p.Asp146Met	2.6
146	p.Asp146His	6.3
146	p.Asp146Gln	1.9
146	p.Asp146Pro	2.4
146	p.Asp146Leu	2.1
146	p.Asp146Asp	8.3
146	p.Asp146Glu	8.2
146	p.Asp146Ala	21.1
146	p.Asp146Gly	8.2
146	p.Asp146Val	2.5
146	p.Asp146Tyr	10.6
146	p.Asp146Cys	1.4
146	p.Asp146Trp	2.2
146	p.Asp146Phe	2.5
147	p.Ala147Asn	3.8

147	p.Ala147Lys	2.6
147	p.Ala147Thr	4.5
147	p.Ala147Arg	4.1
147	p.Ala147Ser	2.2
147	p.Ala147Ile	5.0
147	p.Ala147Met	4.8
147	p.Ala147His	4.5
147	p.Ala147Gln	5.0
147	p.Ala147Pro	3.7
147	p.Ala147Leu	4.4
147	p.Ala147Asp	3.5
147	p.Ala147Glu	7.2
147	p.Ala147Ala	20.2
147	p.Ala147Gly	2.9
147	p.Ala147Val	5.3
147	p.Ala147Tyr	4.2
147	p.Ala147Cys	3.7
147	p.Ala147Trp	4.8
147	p.Ala147Phe	3.6
148	p.Ala148Asn	4.3
148	p.Ala148Lys	4.1
148	p.Ala148Thr	6.0
148	p.Ala148Arg	4.5
148	p.Ala148Ser	4.9
148	p.Ala148Ile	4.9
148	p.Ala148Met	4.2
148	p.Ala148His	3.8
148	p.Ala148Gln	3.7
148	p.Ala148Pro	5.8
148	p.Ala148Leu	4.7
148	p.Ala148Asp	9.7
148	p.Ala148Glu	5.4
148	p.Ala148Ala	8.4
148	p.Ala148Gly	6.1
148	p.Ala148Val	4.1
148	p.Ala148Tyr	4.8
148	p.Ala148Cys	4.3
148	p.Ala148Trp	1.1
148	p.Ala148Phe	5.2
149	p.Glu149Asn	3.2
149	p.Glu149Lys	12.7
149	p.Glu149Thr	3.6
149	p.Glu149Arg	3.5
149	p.Glu149Ser	2.3
149	p.Glu149Ile	4.3

149	p.Glu149Met	3.1
149	p.Glu149His	3.7
149	p.Glu149Gln	7.3
149	p.Glu149Pro	4.4
149	p.Glu149Leu	4.5
149	p.Glu149Asp	8.0
149	p.Glu149Glu	7.6
149	p.Glu149Ala	3.7
149	p.Glu149Gly	3.9
149	p.Glu149Val	10.0
149	p.Glu149Tyr	4.3
149	p.Glu149Cys	2.4
149	p.Glu149Trp	4.6
149	p.Glu149Phe	2.8
150	p.Gly150Asn	3.3
150	p.Gly150Lys	2.3
150	p.Gly150Thr	2.6
150	p.Gly150Arg	4.2
150	p.Gly150Ser	5.6
150	p.Gly150Ile	4.2
150	p.Gly150Met	3.6
150	p.Gly150His	2.4
150	p.Gly150Gln	1.3
150	p.Gly150Pro	3.5
150	p.Gly150Leu	2.7
150	p.Gly150Asp	8.0
150	p.Gly150Glu	3.2
150	p.Gly150Ala	16.1
150	p.Gly150Gly	13.1
150	p.Gly150Val	2.8
150	p.Gly150Tyr	4.1
150	p.Gly150Cys	11.0
150	p.Gly150Trp	2.5
150	p.Gly150Phe	3.5
151	p.Pro151Asn	5.7
151	p.Pro151Lys	6.9
151	p.Pro151Thr	4.4
151	p.Pro151Arg	4.2
151	p.Pro151Ser	1.2
151	p.Pro151Ile	4.7
151	p.Pro151Met	5.9
151	p.Pro151His	6.1
151	p.Pro151Gln	5.1
151	p.Pro151Pro	10.3
151	p.Pro151Leu	5.4

151	p.Pro151Asp	2.6
151	p.Pro151Glu	5.5
151	p.Pro151Ala	2.9
151	p.Pro151Gly	0.3
151	p.Pro151Val	6.3
151	p.Pro151Tyr	6.6
151	p.Pro151Cys	4.1
151	p.Pro151Trp	6.6
151	p.Pro151Phe	5.3
152	p.Ser152Asn	5.1
152	p.Ser152Lys	2.3
152	p.Ser152Thr	6.5
152	p.Ser152Arg	21.3
152	p.Ser152Ser	9.3
152	p.Ser152Ile	2.5
152	p.Ser152Met	2.3
152	p.Ser152His	2.6
152	p.Ser152Gln	2.4
152	p.Ser152Pro	1.5
152	p.Ser152Leu	2.6
152	p.Ser152Asp	2.3
152	p.Ser152Glu	1.8
152	p.Ser152Ala	18.5
152	p.Ser152Gly	2.8
152	p.Ser152Val	2.7
152	p.Ser152Tyr	3.2
152	p.Ser152Cys	5.1
152	p.Ser152Trp	2.6
152	p.Ser152Phe	2.7
153	p.Asp153Asn	13.3
153	p.Asp153Lys	5.2
153	p.Asp153Thr	4.0
153	p.Asp153Arg	4.2
153	p.Asp153Ser	4.6
153	p.Asp153Ile	4.0
153	p.Asp153Met	4.1
153	p.Asp153His	4.9
153	p.Asp153Gln	3.9
153	p.Asp153Pro	2.5
153	p.Asp153Leu	4.3
153	p.Asp153Asp	4.9
153	p.Asp153Glu	5.4
153	p.Asp153Ala	5.3
153	p.Asp153Gly	6.2
153	p.Asp153Val	3.3

153	p.Asp153Tyr	6.0
153	p.Asp153Cys	4.8
153	p.Asp153Trp	4.3
153	p.Asp153Phe	4.6
154	p.Ile154Asn	3.8
154	p.Ile154Lys	3.0
154	p.Ile154Thr	3.2
154	p.Ile154Arg	13.0
154	p.Ile154Ser	3.0
154	p.Ile154Ile	34.2
154	p.Ile154Met	7.7
154	p.Ile154His	2.3
154	p.Ile154Gln	2.5
154	p.Ile154Pro	1.6
154	p.Ile154Leu	2.7
154	p.Ile154Asp	2.9
154	p.Ile154Glu	3.0
154	p.Ile154Ala	2.2
154	p.Ile154Gly	0.5
154	p.Ile154Val	3.2
154	p.Ile154Tyr	2.6
154	p.Ile154Cys	1.9
154	p.Ile154Trp	2.8
154	p.Ile154Phe	4.0
155	p.Pro155Asn	4.6
155	p.Pro155Lys	3.4
155	p.Pro155Thr	5.4
155	p.Pro155Arg	5.6
155	p.Pro155Ser	4.3
155	p.Pro155Ile	3.9
155	p.Pro155Met	4.3
155	p.Pro155His	5.4
155	p.Pro155Gln	4.8
155	p.Pro155Pro	20.7
155	p.Pro155Leu	5.4
155	p.Pro155Asp	4.7
155	p.Pro155Glu	4.3
155	p.Pro155Ala	2.5
155	p.Pro155Gly	3.9
155	p.Pro155Val	3.8
155	p.Pro155Tyr	4.0
155	p.Pro155Cys	3.3
155	p.Pro155Trp	2.8
155	p.Pro155Phe	3.3
156	p.Asp156Asn	13.4

156	p.Asp156Lys	2.7
156	p.Asp156Thr	2.5
156	p.Asp156Arg	2.0
156	p.Asp156Ser	2.6
156	p.Asp156Ile	1.7
156	p.Asp156Met	2.2
156	p.Asp156His	5.6
156	p.Asp156Gln	1.9
156	p.Asp156Pro	2.1
156	p.Asp156Leu	2.1
156	p.Asp156Asp	5.1
156	p.Asp156Glu	8.0
156	p.Asp156Ala	17.7
156	p.Asp156Gly	6.7
156	p.Asp156Val	1.9
156	p.Asp156Tyr	14.6
156	p.Asp156Cys	2.2
156	p.Asp156Trp	1.9
156	p.Asp156Phe	3.1
