# Supplementary material for: Functional characterization of all CDKN2A missense variants and comparison to in silico models of pathogenicity
Source: bioRxiv. 2025 Feb 11:2023.12.28.573507. Originally published 2023 Dec 28. Preprint. [Version 3] doi: 10.1101/2023.12.28.573507 (PMC10793438; doi:10.1101/2023.12.28.573507)
Supplement: Supplement 3 [file media-3.pdf]

Appendix 1-table 3. Proportion of each variant in residues R24, H66, and A127.

Residue	Variant	ACMG Guidline Classification	Percent (%)		Read count	
			Amplified plasmid library	Day 9 cells	Amplified plasmid library	Day 9 cells
24	p.Arg24Asn	VUS	4.56	4.34	8516	4567
24	p.Arg24Lys	VUS	5.15	4.81	9629	5056
24	p.Arg24Thr	VUS	4.42	4.18	8250	4398
24	p.Arg24Arg	Synonymous	4.92	6.32	9196	6651
24	p.Arg24Ser	VUS	5.09	5.00	9504	5259
24	p.Arg24Ile	VUS	4.02	4.13	7503	4338
24	p.Arg24Met	VUS	4.84	5.22	9048	5493
24	p.Arg24His	VUS	5.24	5.07	9788	5328
24	p.Arg24Gln	VUS	5.64	5.29	10542	5563
24	p.Arg24Pro	Pathogenic	3.64	3.79	6803	3981
24	p.Arg24Leu	VUS	4.45	4.32	8319	4538
24	p.Arg24Asp	VUS	7.13	6.91	13323	7271
24	p.Arg24Glu	VUS	5.19	5.09	9692	5349
24	p.Arg24Ala	VUS	4.52	4.28	8447	4498
24	p.Arg24Gly	VUS	4.06	4.01	7595	4217
24	p.Arg24Val	VUS	6.48	6.42	12100	6754
24	p.Arg24Tyr	VUS	4.58	4.56	8553	4797
24	p.Arg24Cys	VUS	4.48	4.40	8366	4627
24	p.Arg24Trp	VUS	6.23	6.65	11641	6995
24	p.Arg24Phe	VUS	5.38	5.21	10044	5482
66	p.His66Asn	VUS	4.71	3.52	5574	1413
66	p.His66Lys	VUS	5.85	4.48	6935	1796
66	p.His66Thr	VUS	4.70	3.60	5573	1442
66	p.His66Arg	VUS	5.25	4.85	6216	1944
66	p.His66Ser	VUS	5.10	4.57	6038	1831
66	p.His66Ile	VUS	4.83	5.04	5726	2019
66	p.His66Met	VUS	5.30	3.81	6278	1527
66	p.His66His	Synonymous	3.88	4.94	4591	1982
66	p.His66Gln	VUS	5.28	8.89	6250	3565
66	p.His66Pro	VUS	6.13	6.07	7260	2432
66	p.His66Leu	VUS	5.55	5.83	6575	2338
66	p.His66Asp	VUS	2.77	2.92	3286	1169
66	p.His66Glu	VUS	6.10	6.04	7229	2420
66	p.His66Ala	VUS	5.41	5.95	6414	2387
66	p.His66Gly	VUS	5.56	5.24	6587	2100
66	p.His66Val	VUS	4.58	4.47	5430	1793
66	p.His66Tyr	VUS	4.55	4.66	5384	1869
66	p.His66Cys	VUS	4.53	4.63	5367	1856
66	p.His66Trp	VUS	5.40	5.55	6397	2225
66	p.His66Phe	VUS	4.51	4.94	5346	1981
127	p.Ala127Asn	Benign	4.62	4.37	3844	6274
127	p.Ala127Lys	VUS	4.37	4.36	3631	6261
127	p.Ala127Thr	VUS	4.44	3.99	3693	5737
127	p.Ala127Arg	VUS	5.85	5.79	4862	8321
127	p.Ala127Ser	VUS	4.95	5.38	4117	7732
127	p.Ala127Ile	VUS	4.81	4.92	4000	7070
127	p.Ala127Met	VUS	5.79	6.01	4818	8638
127	p.Ala127His	VUS	4.24	4.31	3529	6189
127	p.Ala127Gln	VUS	5.09	5.50	4233	7900
127	p.Ala127Pro	VUS	2.84	2.63	2364	3778
127	p.Ala127Leu	VUS	5.06	4.74	4209	6805
127	p.Ala127Asp	VUS	6.04	6.53	5023	9386
127	p.Ala127Glu	VUS	6.07	5.60	5052	8045
127	p.Ala127Ala	Synonymous	4.27	3.68	3548	5292
127	p.Ala127Gly	VUS	4.75	4.74	3949	6809
127	p.Ala127Val	VUS	6.33	6.83	5268	9821
127	p.Ala127Tyr	VUS	4.33	3.86	3599	5544
127	p.Ala127Cys	VUS	4.44	4.93	3696	7087
127	p.Ala127Trp	VUS	5.97	5.92	4962	8513
127	p.Ala127Phe	VUS	5.73	5.92	4767	8512
