# Supplementary material for: Functional characterization of all CDKN2A missense variants and comparison to in silico models of pathogenicity
Source: bioRxiv. 2025 Feb 11:2023.12.28.573507. Originally published 2023 Dec 28. Preprint. [Version 3] doi: 10.1101/2023.12.28.573507 (PMC10793438; doi:10.1101/2023.12.28.573507)
Supplement: Supplement 4 [file media-4.pdf]

Appendix 1-table 4. Assay outputs and functional classifications for all possible *CDKN2A* missense and synonymous variants.

Residue	Variant	Benchmark	Functionally reported VUS	Experiment_1				Experiment_2				Experiment_1		Experiment_2		Merged	
				Read count		Proportion		Read count		Proportion							
				Day 9_1	Confluent_1	Day 9_1	Confluent_1	Day 9_2	Confluent_2	Day 9_2	Confluent_2	Log P value_1	Fucntional characterization_1	Log P value_2	Fucntional characterization_2	Log P value_merged	Fucntional characterization _merged
1	p.Met1Asn			7917	3240	4.45	4.27					-1.53E-01	Neutral			-1.53E-01	Neutral
1	p.Met1Lys			10656	3866	5.99	5.10					-8.17E-03	Neutral			-8.17E-03	Neutral
1	p.Met1Thr			8682	3625	4.88	4.78					-1.36E-01	Neutral			-1.36E-01	Neutral
1	p.Met1Arg			4562	1607	2.56	2.12					-2.18E-01	Neutral			-2.18E-01	Neutral
1	p.Met1Ser			14850	6066	8.35	8.00					-9.32E-03	Neutral			-9.32E-03	Neutral
1	p.Met1Ile			5660	2304	3.18	3.04					-4.38E-01	Neutral			-4.38E-01	Neutral
1	p.Met1Met	Synonymous		5258	2332	2.96	3.08					-1.06E+00	Neutral			-1.06E+00	Neutral
1	p.Met1His			5078	2285	2.85	3.01					-1.27E+00	Neutral			-1.27E+00	Neutral
1	p.Met1Gln			9585	4102	5.39	5.41					-1.24E-01	Neutral			-1.24E-01	Neutral
1	p.Met1Pro			5141	1985	2.89	2.62					-3.62E-01	Neutral			-3.62E-01	Neutral
1	p.Met1Leu			14664	6252	8.24	8.25					-1.90E-02	Neutral			-1.90E-02	Neutral
1	p.Met1Asp			14894	5681	8.37	7.49					-2.98E-03	Neutral			-2.98E-03	Neutral
1	p.Met1Glu			5078	2195	2.85	2.89					-9.57E-01	Neutral			-9.57E-01	Neutral
1	p.Met1Ala			8804	3502	4.95	4.62					-7.30E-02	Neutral			-7.30E-02	Neutral
1	p.Met1Gly			6523	2720	3.67	3.59					-3.55E-01	Neutral			-3.55E-01	Neutral
1	p.Met1Val			11052	7911	6.21	10.43					-4.16E+00	Neutral			-4.16E+00	Neutral
1	p.Met1Tyr			12992	5282	7.30	6.97					-1.70E-02	Neutral			-1.70E-02	Neutral
1	p.Met1Cys			14675	5858	8.25	7.73					-6.89E-03	Neutral			-6.89E-03	Neutral
1	p.Met1Trp			3926	1599	2.21	2.11					-1.15E+00	Neutral			-1.15E+00	Neutral
1	p.Met1Phe			7910	3411	4.45	4.50					-2.65E-01	Neutral			-2.65E-01	Neutral
2	p.Glu2Asn			6727	2813	4.99	2.50					-2.75E-14	Neutral			0.00E+00	Neutral
2	p.Glu2Lys			6744	5413	5.01	4.81					-2.54E-06	Neutral			-2.54E-06	Neutral
2	p.Glu2Thr			5212	4425	3.87	3.93					-6.21E-05	Neutral			-6.21E-05	Neutral
2	p.Glu2Arg			5222	2299	3.88	2.04					-1.88E-12	Neutral			0.00E+00	Neutral
2	p.Glu2Ser			4630	6041	3.44	5.36					-1.34E-01	Neutral			-1.34E-01	Neutral
2	p.Glu2Ile			7541	3779	5.60	3.36					-2.35E-12	Neutral			0.00E+00	Neutral
2	p.Glu2Met			6300	7369	4.68	6.54					-7.63E-03	Neutral			-7.63E-03	Neutral
2	p.Glu2His			7815	6349	5.80	5.64					-1.05E-06	Neutral			-1.05E-06	Neutral
2	p.Glu2Gln			9277	5087	6.89	4.52					-4.48E-12	Neutral			0.00E+00	Neutral
2	p.Glu2Pro			8059	5092	5.98	4.52					-1.09E-09	Neutral			-9.43E-10	Neutral
2	p.Glu2Leu			7335	3699	5.44	3.28					-3.77E-12	Neutral			0.00E+00	Neutral
2	p.Glu2Asp			4954	1515	3.68	1.35					0.00E+00	Neutral			0.00E+00	Neutral
2	p.Glu2Glu	Synonymous		9092	18647	6.75	16.56					-1.06E+00	Neutral			-1.06E+00	Neutral
2	p.Glu2Ala			6504	8164	4.83	7.25					-1.91E-02	Neutral			-1.91E-02	Neutral
2	p.Glu2Gly			5629	4118	4.18	3.66					-1.16E-06	Neutral			-1.16E-06	Neutral
2	p.Glu2Val			6747	3223	5.01	2.86					-1.69E-12	Neutral			0.00E+00	Neutral
2	p.Glu2Tyr			4650	2961	3.45	2.63					-1.78E-07	Neutral			-1.78E-07	Neutral
2	p.Glu2Cys			6088	7985	4.52	7.09					-4.74E-02	Neutral			-4.74E-02	Neutral
2	p.Glu2Trp			7642	6902	5.67	6.13					-1.49E-05	Neutral			-1.49E-05	Neutral
2	p.Glu2Phe			8566	6750	6.36	5.99					-2.31E-07	Neutral			-2.31E-07	Neutral
3	p.Pro3Asn			10479	4032	5.98	5.43					-7.64E+00	Indeterminate			-7.64E+00	Indeterminate
3	p.Pro3Lys			6030	2500	3.44	3.37					-1.84E+01	Indeterminate			-1.84E+01	Indeterminate
3	p.Pro3Thr			8211	3197	4.69	4.31					-1.08E+01	Indeterminate			-1.08E+01	Indeterminate
3	p.Pro3Arg			8154	5051	4.66	6.81					-4.02E+01	Indeterminate			-3.32E+01	Indeterminate
3	p.Pro3Ser			7465	3568	4.26	4.81					-2.25E+01	Indeterminate			-2.25E+01	Indeterminate
3	p.Pro3Ile			6870	2552	3.92	3.44					-1.13E+01	Indeterminate			-1.13E+01	Indeterminate
3	p.Pro3Met			6862	3240	3.92	4.37					-2.35E+01	Indeterminate			-2.35E+01	Indeterminate
3	p.Pro3His			6669	2007	3.81	2.71					-5.11E+00	Neutral			-5.11E+00	Neutral
3	p.Pro3Gln			7887	1624	4.50	2.19					-2.77E-01	Neutral			-2.77E-01	Neutral
3	p.Pro3Pro	Synonymous		12225	3414	6.98	4.60					-1.06E+00	Neutral			-1.06E+00	Neutral
3	p.Pro3Leu			7911	3359	4.52	4.53					-1.50E+01	Indeterminate			-1.50E+01	Indeterminate
3	p.Pro3Asp			11034	5168	6.30	6.97					-1.44E+01	Indeterminate			-1.44E+01	Indeterminate
3	p.Pro3Glu			8543	2741	4.88	3.69					-4.70E+00	Neutral			-4.70E+00	Neutral
3	p.Pro3Ala			7836	2325	4.47	3.13					-3.70E+00	Neutral			-3.70E+00	Neutral
3	p.Pro3Gly			9287	7121	5.30	9.60					-5.32E+01	Deleterious			-5.32E+01	Deleterious
3	p.Pro3Val			11755	5610	6.71	7.56					-1.43E+01	Indeterminate			-1.43E+01	Indeterminate
3	p.Pro3Tyr			8286	2722	4.73	3.67					-5.47E+00	Neutral			-5.47E+00	Neutral
3	p.Pro3Cys			9700	4045	5.54	5.45					-1.13E+01	Indeterminate			-1.13E+01	Indeterminate
3	p.Pro3Trp			7181	3898	4.10	5.25					-3.25E+01	Indeterminate			-3.18E+01	Indeterminate
3	p.Pro3Phe			12773	6021	7.29	8.12					-1.26E+01	Indeterminate			-1.26E+01	Indeterminate
4	p.Ala4Asn			6496	5119	5.55	5.97					-5.71E+00	Neutral			-5.71E+00	Neutral
4	p.Ala4Lys			5599	3201	4.79	3.73					-1.31E+00	Neutral			-1.31E+00	Neutral
4	p.Ala4Thr			6049	9770	5.17	11.39					-5.14E+01	Indeterminate			-3.32E+01	Indeterminate
4	p.Ala4Arg			6946	4316	5.94	5.03					-1.41E+00	Neutral			-1.41E+00	Neutral
4	p.Ala4Ser			5204	8721	4.45	10.16					-5.32E+01	Deleterious			-5.32E+01	Deleterious
4	p.Ala4Ile			5102	2244	4.36	2.62					-1.79E-01	Neutral			-1.79E-01	Neutral
4	p.Ala4Met			6306	5309	5.39	6.19					-7.81E+00	Indeterminate			-7.81E+00	Indeterminate
4	p.Ala4His			9180	6186	7.85	7.21					-1.31E+00	Neutral			-1.31E+00	Neutral
4	p.Ala4Gln			4555	2044	3.89	2.38					-3.21E-01	Neutral			-3.21E-01	Neutral
4	p.Ala4Pro			4683	2290	4.00	2.67					-6.33E-01	Neutral			-6.33E-01	Neutral
4	p.Ala4Leu			6006	3343	5.13	3.90					-9.14E-01	Neutral			-9.14E-01	Neutral
4	p.Ala4Asp			4923	3133	4.21	3.65					-3.20E+00	Neutral			-3.20E+00	Neutral
4	p.Ala4Glu			5399	2024	4.62	2.36					-1.89E-02	Neutral			-1.89E-02	Neutral
4	p.Ala4Ala	Synonymous		6423	3731	5.49	4.35					-1.06E+00	Neutral			-1.06E+00	Neutral
4	p.Ala4Gly			5628	4372	4.81	5.10					-6.58E+00	Indeterminate			-6.58E+00	Indeterminate
4	p.Ala4Val			6269	2830	5.36	3.30					-1.12E-01	Neutral			-1.12E-01	Neutral
4	p.Ala4Tyr			5640	2955	4.82	3.44					-6.72E-01	Neutral			-6.72E-01	Neutral
4	p.Ala4Cys			4485	5419	3.83	6.32					-3.33E+01	Indeterminate			-3.23E+01	Indeterminate
4	p.Ala4Trp			6700	4351	5.73	5.07					-2.02E+00	Neutral			-2.02E+00	Neutral
4	p.Ala4Phe			5374	4447	4.59	5.18					-8.96E+00	Indeterminate			-8.96E+00	Indeterminate
5	p.Ala5Asn			10398	3217	7.08	4.47					-1.02E-02	Neutral			-1.02E-02	Neutral
5	p.Ala5Lys			6616	943	4.50	1.31					-1.32E-08	Neutral			-1.31E-08	Neutral
5	p.Ala5Thr			7235	2897	4.92	4.02					-7.68E-01	Neutral			-7.68E-01	Neutral
5	p.Ala5Arg			6763	1495	4.60	2.08					-3.35E-04	Neutral			-3.35E-04	Neutral
5	p.Ala5Ser			6901	7643	4.70	10.62					-4.59E+01	Indeterminate			-3.32E+01	Indeterminate
5	p.Ala5Ile			9416	1133	6.41	1.57					-3.62E-12	Neutral			0.00E+00	Neutral
5	p.Ala5Met			6059	2031	4.12	2.82					-2.70E-01	Neutral			-2.70E-01	Neutral
5	p.Ala5His			9217	3925	6.27	5.45					-6.61E-01	Neutral			-6.61E-01	Neutral
5	p.Ala5Gln			8603	3685	5.86	5.12					-8.29E-01	Neutral			-8.29E-01	Neutral
5	p.Ala5Pro			8547	2852	5.82	3.96					-7.23E-02	Neutral			-7.23E-02	Neutral
5	p.Ala5Leu			10779	7833	7.34	10.88					-9.21E+00	Indeterminate			-9.21E+00	Indeterminate
5	p.Ala5Asp			7000	3082	4.76	4.28					-1.63E+00	Neutral			-1.63E+00	Neutral
5	p.Ala5Glu			5477	2907	3.73	4.04					-6.48E+00	Indeterminate			-6.48E+00	Indeterminate
5	p.Ala5Ala	Synonymous		5728	2216	3.90	3.08					-1.06E+00	Neutral			-1.06E+00	Neutral
5	p.Ala5Gly			4494	1339	3.06	1.86					-2.24E-01	Neutral			-2.24E-01	Neutral
5	p.Ala5Val			8831	3044	6.01	4.23					-9.39E-02	Neutral			-9.39E-02	Neutral
5	p.Ala5Tyr			4625	8628	3.15	11.99					-5.32E+01	Deleterious			-5.32E+01	Deleterious
5	p.Ala5Cys			5753	2808	3.92	3.90					-4.14E+00	Neutral			-4.14E+00	Neutral
5	p.Ala5Trp			8851	8353	6.02	11.60					-2.55E+01	Indeterminate			-2.55E+01	Indeterminate
5	p.Ala5Phe			5632	1954	3.83	2.71					-4.66E-01	Neutral			-4.66E-01	Neutral
6	p.Gly6Asn			5526	2196	5.53	2.96					-9.29E-02	Neutral			-9.29E-02	Neutral
6	p.Gly6Lys			5673	3312	5.68	4.46					-2.25E+00	Neutral			-2.25E+00	Neutral
6	p.Gly6Thr			4626	2843	4.63	3.83					-4.22E+00	Neutral			-4.22E+00	Neutral
6	p.Gly6Arg			4400	8247	4.40	11.12					-5.32E+01	Deleterious			-5.32E+01	Deleterious
6	p.Gly6Ser			5199	7507	5.20	10.12					-5.20E+01	Indeterminate			-3.32E+01	Indeterminate
6	p.Gly6Ile			4539	7126	4.54	9.61					-5.32E+01	Deleterious			-5.32E+01	Deleterious
6	p.Gly6Met			7887	11562	7.89	15.59					-3.98E+01	Indeterminate			-3.32E+01	Indeterminate
6	p.Gly6His			4522	1360	4.53	1.83					-4.50E-03	Neutral			-4.50E-03	Neutral
6	p.Gly6Gln			4363	1586	4.37	2.14					-8.17E-02	Neutral			-8.17E-02	Neutral
6	p.Gly6Pro			3564	3083	3.57	4.16					-2.03E+01	Indeterminate			-2.03E+01	Indeterminate
6	p.Gly6Leu			6157	3203	6.16	4.32					-8.72E-01	Neutral			-8.72E-01	Neutral
6	p.Gly6Asp			4366	1905	4.37	2.57					-5.31E-01	Neutral			-5.31E-01	Neutral
6	p.Gly6Glu			5386	2616	5.39	3.53					-7.22E-01	Neutral			-7.22E-01	Neutral
6	p.Gly6Ala			6037	2648	6.04	3.57					-2.01E-01	Neutral			-2.01E-01	Neutral
6	p.Gly6Gly	Synonymous		2942	1230	2.94	1.66					-1.06E+00	Neutral			-1.06E+00	Neutral
6	p.Gly6Val			7551	3158	7.56	4.26					-4.82E-02	Neutral			-4.82E-02	Neutral
6	p.Gly6Tyr			3436	1449	3.44	1.95					-7.69E-01	Neutral			-7.69E-01	Neutral
6	p.Gly6Cys			3736	2856	3.74	3.85					-1.32E+01	Indeterminate			-1.32E+01	Indeterminate
6	p.Gly6Trp			5083	3058	5.09	4.12					-3.24E+00	Neutral			-3.24E+00	Neutral
6	p.Gly6Phe			4916	3241	4.92	4.37					-5.31E+00	Neutral			-5.31E+00	Neutral
7	p.Ser7Asn			21503	2770	5.64	3.85					-7.01E-02	Neutral			-7.01E-02	Neutral
7	p.Ser7Lys			17957	2366	4.71	3.28					-1.81E-01	Neutral			-1.81E-01	Neutral
7	p.Ser7Thr			26503	7810	6.95	10.84					-1.12E+01	Indeterminate			-1.12E+01	Indeterminate
7	p.Ser7Arg			20121	2384	5.28	3.31					-3.13E-02	Neutral			-3.13E-02	Neutral
7	p.Ser7Ser	Synonymous		13032	1887	3.42	2.62					-1.06E+00	Neutral			-1.06E+00	Neutral
7	p.Ser7Ile			21968	3908	5.76	5.43					-1.33E+00	Neutral			-1.33E+00	Neutral
7	p.Ser7Met			25433	4362	6.67	6.06					-7.12E-01	Neutral			-7.12E-01	Neutral
7	p.Ser7His			23901	5103	6.27	7.08					-3.32E+00	Neutral			-3.32E+00	Neutral
7	p.Ser7Gln			25626	4097	6.72	5.69					-3.82E-01	Neutral			-3.82E-01	Neutral
7	p.Ser7Pro			30865	5648	8.09	7.84					-7.15E-01	Neutral			-7.15E-01	Neutral
7	p.Ser7Leu			22752	5920	5.97	8.22					-8.57E+00	Indeterminate			-8.57E+00	Indeterminate
7	p.Ser7Asp			13045	1864	3.42	2.59					-9.61E-01	Neutral			-9.61E-01	Neutral

7	p.Ser7Glu			5274	1263	1.38	1.75	-2.95E+01	Indeterminate	-2.94E+01	Indeterminate
7	p.Ser7Ala			13797	3900	3.62	5.41	-1.95E+01	Indeterminate	-1.95E+01	Indeterminate
7	p.Ser7Gly			34546	4664	9.06	6.48	-1.55E-02	Neutral	-1.55E-02	Neutral
7	p.Ser7Val			6451	937	1.69	1.30	-4.25E+00	Neutral	-4.25E+00	Neutral
7	p.Ser7Tyr			14033	5213	3.68	7.24	-3.89E+01	Indeterminate	-3.32E+01	Indeterminate
7	p.Ser7Cys			15237	2771	4.00	3.85	-3.14E+00	Neutral	-3.14E+00	Neutral
7	p.Ser7Trp			10664	2020	2.80	2.80	-6.60E+00	Indeterminate	-6.60E+00	Indeterminate
7	p.Ser7Phe			18617	3141	4.88	4.36	-1.35E+00	Neutral	-1.35E+00	Neutral
8	p.Ser8Asn			5102	2754	5.51	4.26	-1.03E-03	Neutral	-1.03E-03	Neutral
8	p.Ser8Lys			3518	5765	3.80	8.91	-2.11E+01	Indeterminate	-2.11E+01	Indeterminate
8	p.Ser8Thr			5291	4626	5.72	7.15	-5.41E-01	Neutral	-5.41E-01	Neutral
8	p.Ser8Arg			4648	3049	5.02	4.71	-3.81E-02	Neutral	-3.81E-02	Neutral
8	p.Ser8Ser	Synonymous		5221	4953	5.64	7.66	-1.06E+00	Neutral	-1.06E+00	Neutral
8	p.Ser8Ile			6586	4481	7.11	6.93	-1.22E-02	Neutral	-1.22E-02	Neutral
8	p.Ser8Met			3576	1885	3.86	2.91	-5.33E-03	Neutral	-5.33E-03	Neutral
8	p.Ser8His			4006	2293	4.33	3.54	-1.09E-02	Neutral	-1.09E-02	Neutral
8	p.Ser8Gln			5026	3177	5.43	4.91	-1.56E-02	Neutral	-1.56E-02	Neutral
8	p.Ser8Pro			3368	2661	3.64	4.11	-8.03E-01	Neutral	-8.03E-01	Neutral
8	p.Ser8Leu			4268	3385	4.61	5.23	-4.31E-01	Neutral	-4.31E-01	Neutral
8	p.Ser8Asp			5645	3906	6.10	6.04	-3.28E-02	Neutral	-3.28E-02	Neutral
8	p.Ser8Glu			5235	2231	5.65	3.45	-6.17E-06	Neutral	-6.17E-06	Neutral
8	p.Ser8Ala			3572	2303	3.86	3.56	-9.28E-02	Neutral	-9.28E-02	Neutral
8	p.Ser8Gly			4071	3170	4.40	4.90	-4.21E-01	Neutral	-4.21E-01	Neutral
8	p.Ser8Val			5367	3622	5.80	5.60	-2.92E-02	Neutral	-2.92E-02	Neutral
8	p.Ser8Tyr			4234	2444	4.57	3.78	-9.30E-03	Neutral	-9.30E-03	Neutral
8	p.Ser8Cys			4875	2414	5.27	3.73	-2.62E-04	Neutral	-2.62E-04	Neutral
8	p.Ser8Trp			5137	2823	5.55	4.36	-1.37E-03	Neutral	-1.37E-03	Neutral
8	p.Ser8Phe			3833	2741	4.14	4.24	-2.25E-01	Neutral	-2.25E-01	Neutral
9	p.Met9Asn			7469	3933	4.84	4.81	-2.11E-01	Neutral	-2.11E-01	Neutral
9	p.Met9Lys			9609	5540	6.23	6.77	-2.27E-01	Neutral	-2.27E-01	Neutral
9	p.Met9Thr		Neutral	8791	5010	5.70	6.12	-2.72E-01	Neutral	-2.72E-01	Neutral
9	p.Met9Arg			10762	5831	6.97	7.12	-7.40E-02	Neutral	-7.40E-02	Neutral
9	p.Met9Ser			5046	2796	3.27	3.42	-1.02E+00	Neutral	-1.02E+00	Neutral
9	p.Met9Ile			8383	4731	5.43	5.78	-2.88E-01	Neutral	-2.88E-01	Neutral
9	p.Met9Met	Synonymous		3169	1512	2.05	1.85	-1.06E+00	Neutral	-1.06E+00	Neutral
9	p.Met9His			4318	2163	2.80	2.64	-7.05E-01	Neutral	-7.05E-01	Neutral
9	p.Met9Gln			5190	2565	3.36	3.13	-3.71E-01	Neutral	-3.71E-01	Neutral
9	p.Met9Pro			6382	3588	4.14	4.38	-6.27E-01	Neutral	-6.27E-01	Neutral
9	p.Met9Leu			8726	3851	5.66	4.71	-1.14E-02	Neutral	-1.14E-02	Neutral
9	p.Met9Asp			9850	5446	6.38	6.65	-1.33E-01	Neutral	-1.33E-01	Neutral
9	p.Met9Glu			8636	4204	5.60	5.14	-4.81E-02	Neutral	-4.81E-02	Neutral
9	p.Met9Ala			8064	4131	5.23	5.05	-1.19E-01	Neutral	-1.19E-01	Neutral
9	p.Met9Gly			6394	3368	4.14	4.12	-3.52E-01	Neutral	-3.52E-01	Neutral
9	p.Met9Val			8528	5087	5.53	6.22	-4.58E-01	Neutral	-4.58E-01	Neutral
9	p.Met9Tyr			9867	4712	6.39	5.76	-2.01E-02	Neutral	-2.01E-02	Neutral
9	p.Met9Cys			8011	4120	5.19	5.03	-1.27E-01	Neutral	-1.27E-01	Neutral
9	p.Met9Trp			9040	4855	5.86	5.93	-1.32E-01	Neutral	-1.32E-01	Neutral
9	p.Met9Phe			8063	4400	5.23	5.38	-2.35E-01	Neutral	-2.35E-01	Neutral
10	p.Glu10Asn			6098	2242	4.21	3.54	-2.65E+00	Neutral	-2.65E+00	Neutral
10	p.Glu10Lys			5462	4301	3.77	6.80	-4.13E+01	Indeterminate	-3.32E+01	Indeterminate
10	p.Glu10Thr			5428	2183	3.75	3.45	-5.04E+00	Neutral	-5.04E+00	Neutral
10	p.Glu10Arg			6859	2202	4.74	3.48	-8.52E-01	Neutral	-8.52E-01	Neutral
10	p.Glu10Ser			4238	2388	2.93	3.77	-2.20E+01	Indeterminate	-2.20E+01	Indeterminate
10	p.Glu10Ile			5736	3004	3.96	4.75	-1.30E+01	Indeterminate	-1.30E+01	Indeterminate
10	p.Glu10Met			10083	5755	6.96	9.09	-9.12E+00	Indeterminate	-9.12E+00	Indeterminate
10	p.Glu10His			7085	5545	4.89	8.76	-3.32E+01	Indeterminate	-3.22E+01	Indeterminate
10	p.Glu10Gln			8938	2836	6.17	4.48	-3.68E-01	Neutral	-3.68E-01	Neutral
10	p.Glu10Pro			9004	3151	6.22	4.98	-8.25E-01	Neutral	-8.25E-01	Neutral
10	p.Glu10Leu			4693	1833	3.24	2.90	-5.49E+00	Neutral	-5.49E+00	Neutral
10	p.Glu10Asp			7860	3023	5.43	4.78	-2.12E+00	Neutral	-2.12E+00	Neutral
10	p.Glu10Glu	Synonymous		7526	2567	5.20	4.06	-1.06E+00	Neutral	-1.06E+00	Neutral
10	p.Glu10Ala			3045	1836	2.10	2.90	-3.48E+01	Indeterminate	-3.28E+01	Indeterminate
10	p.Glu10Gly			7598	2700	5.25	4.27	-1.38E+00	Neutral	-1.38E+00	Neutral
10	p.Glu10Val			8257	2931	5.70	4.63	-1.13E+00	Neutral	-1.13E+00	Neutral
10	p.Glu10Tyr			11783	5357	8.14	8.47	-2.56E+00	Neutral	-2.56E+00	Neutral
10	p.Glu10Cys			11223	4410	7.75	6.97	-1.13E+00	Neutral	-1.13E+00	Neutral
10	p.Glu10Trp			7320	2533	5.05	4.00	-1.25E+00	Neutral	-1.25E+00	Neutral
10	p.Glu10Phe			6589	2486	4.55	3.93	-2.65E+00	Neutral	-2.65E+00	Neutral
11	p.Pro11Asn			8506	1300	5.16	5.15	-1.32E+00	Neutral	-1.32E+00	Neutral
11	p.Pro11Lys			6439	1122	3.91	4.44	-4.63E+00	Neutral	-4.63E+00	Neutral
11	p.Pro11Thr			5725	1120	3.47	4.43	-8.84E+00	Indeterminate	-8.84E+00	Indeterminate
11	p.Pro11Arg			6357	933	3.86	3.69	-1.90E+00	Neutral	-1.90E+00	Neutral
11	p.Pro11Ser			8111	1330	4.92	5.27	-2.27E+00	Neutral	-2.27E+00	Neutral
11	p.Pro11Ile			8262	1268	5.01	5.02	-1.45E+00	Neutral	-1.45E+00	Neutral
11	p.Pro11Met			12247	1773	7.43	7.02	-3.21E-01	Neutral	-3.21E-01	Neutral
11	p.Pro11His			7706	1174	4.68	4.65	-1.60E+00	Neutral	-1.60E+00	Neutral
11	p.Pro11Gln			8805	1147	5.34	4.54	-3.33E-01	Neutral	-3.33E-01	Neutral
11	p.Pro11Pro	Synonymous		6518	884	3.95	3.50	-1.06E+00	Neutral	-1.06E+00	Neutral
11	p.Pro11Leu		Neutral	7222	1095	4.38	4.34	-1.78E+00	Neutral	-1.78E+00	Neutral
11	p.Pro11Asp			10405	1506	6.31	5.96	-5.22E-01	Neutral	-5.22E-01	Neutral
11	p.Pro11Glu			7910	1079	4.80	4.27	-6.80E-01	Neutral	-6.80E-01	Neutral
11	p.Pro11Ala			12124	1807	7.36	7.15	-4.33E-01	Neutral	-4.33E-01	Neutral
11	p.Pro11Gly			8624	1400	5.23	5.54	-1.89E+00	Neutral	-1.89E+00	Neutral
11	p.Pro11Val			8786	1298	5.33	5.14	-9.61E-01	Neutral	-9.61E-01	Neutral
11	p.Pro11Tyr			7352	1214	4.46	4.81	-2.83E+00	Neutral	-2.83E+00	Neutral
11	p.Pro11Cys			7776	1069	4.72	4.23	-7.57E-01	Neutral	-7.57E-01	Neutral
11	p.Pro11Trp			7900	1402	4.79	5.55	-3.64E+00	Neutral	-3.64E+00	Neutral
11	p.Pro11Phe			8057	1336	4.89	5.29	-2.45E+00	Neutral	-2.45E+00	Neutral
12	p.Ser12Asn			4278	1302	3.95	3.63	-1.24E+00	Neutral	-1.24E+00	Neutral
12	p.Ser12Lys			4907	1159	4.53	3.23	-7.52E-02	Neutral	-7.52E-02	Neutral
12	p.Ser12Thr			5668	1434	5.24	4.00	-9.67E-02	Neutral	-9.67E-02	Neutral
12	p.Ser12Arg			4891	1365	4.52	3.81	-4.41E-01	Neutral	-4.41E-01	Neutral
12	p.Ser12Ser	Synonymous		5874	1944	5.43	5.42	-1.06E+00	Neutral	-1.06E+00	Neutral
12	p.Ser12Ile			4931	1318	4.56	3.67	-2.87E-01	Neutral	-2.87E-01	Neutral
12	p.Ser12Met			5639	1332	5.21	3.71	-4.17E-02	Neutral	-4.17E-02	Neutral
12	p.Ser12His			5177	1913	4.78	5.33	-2.76E+00	Neutral	-2.76E+00	Neutral
12	p.Ser12Gln			6165	2351	5.70	6.55	-2.38E+00	Neutral	-2.38E+00	Neutral
12	p.Ser12Pro			6926	1787	6.40	4.98	-5.42E-02	Neutral	-5.42E-02	Neutral
12	p.Ser12Leu			7110	5519	6.57	15.38	-2.85E+01	Indeterminate	-2.84E+01	Indeterminate
12	p.Ser12Asp			6223	1364	5.75	3.80	-8.73E-03	Neutral	-8.73E-03	Neutral
12	p.Ser12Glu			3816	1115	3.53	3.11	-1.21E+00	Neutral	-1.21E+00	Neutral
12	p.Ser12Ala			4304	1215	3.98	3.39	-6.95E-01	Neutral	-6.95E-01	Neutral
12	p.Ser12Gly			6165	1672	5.70	4.66	-1.55E-01	Neutral	-1.55E-01	Neutral
12	p.Ser12Val			3830	1433	3.54	3.99	-4.85E+00	Neutral	-4.85E+00	Neutral
12	p.Ser12Tyr			5082	1268	4.70	3.53	-1.25E-01	Neutral	-1.25E-01	Neutral
12	p.Ser12Cys			5540	2071	5.12	5.77	-2.60E+00	Neutral	-2.60E+00	Neutral
12	p.Ser12Trp			4602	1720	4.25	4.79	-3.60E+00	Neutral	-3.60E+00	Neutral
12	p.Ser12Phe			7080	2591	6.54	7.22	-1.39E+00	Neutral	-1.39E+00	Neutral
13	p.Ala13Asn			4218	1562	5.37	4.39	-1.90E-03	Neutral	-1.90E-03	Neutral
13	p.Ala13Lys			3029	1313	3.85	3.69	-9.89E-02	Neutral	-9.89E-02	Neutral
13	p.Ala13Thr			3969	1691	5.05	4.75	-2.47E-02	Neutral	-2.47E-02	Neutral
13	p.Ala13Arg			4846	1789	6.17	5.03	-7.73E-04	Neutral	-7.73E-04	Neutral
13	p.Ala13Ser			6020	2315	7.66	6.51	-4.26E-04	Neutral	-4.26E-04	Neutral
13	p.Ala13Ile			3905	1553	4.97	4.37	-9.58E-03	Neutral	-9.58E-03	Neutral
13	p.Ala13Met			5301	3446	6.75	9.69	-8.15E-01	Neutral	-8.15E-01	Neutral
13	p.Ala13His			3118	1431	3.97	4.02	-1.69E-01	Neutral	-1.69E-01	Neutral
13	p.Ala13Gln			3692	1490	4.70	4.19	-1.59E-02	Neutral	-1.59E-02	Neutral
13	p.Ala13Pro			4393	1429	5.59	4.02	-1.21E-04	Neutral	-1.21E-04	Neutral
13	p.Ala13Leu			2886	1301	3.67	3.66	-1.84E-01	Neutral	-1.84E-01	Neutral
13	p.Ala13Asp			4736	1965	6.03	5.52	-6.79E-03	Neutral	-6.79E-03	Neutral
13	p.Ala13Glu			3561	1393	4.53	3.92	-1.19E-02	Neutral	-1.19E-02	Neutral
13	p.Ala13Ala	Synonymous		3906	2377	4.97	6.68	-1.06E+00	Neutral	-1.06E+00	Neutral
13	p.Ala13Gly			3294	1146	4.19	3.22	-2.68E-03	Neutral	-2.68E-03	Neutral
13	p.Ala13Val			3458	1473	4.40	4.14	-4.60E-02	Neutral	-4.60E-02	Neutral
13	p.Ala13Tyr			3988	2503	5.08	7.04	-1.26E+00	Neutral	-1.26E+00	Neutral
13	p.Ala13Cys			2683	1335	3.41	3.75	-5.86E-01	Neutral	-5.86E-01	Neutral
13	p.Ala13Trp			2538	1184	3.23	3.33	-3.92E-01	Neutral	-3.92E-01	Neutral
13	p.Ala13Phe			5038	2876	6.41	8.09	-3.08E-01	Neutral	-3.08E-01	Neutral
14	p.Asp14Asn			3574	1772	3.96	3.01	-3.70E+00	Neutral	-3.70E+00	Neutral
14	p.Asp14Lys			4011	1920	4.44	3.26	-2.50E+00	Neutral	-2.50E+00	Neutral
14	p.Asp14Thr			5279	2173	5.85	3.69	-4.49E-01	Neutral	-4.49E-01	Neutral
14	p.Asp14Arg			3601	2556	3.99	4.34	-1.51E+01	Indeterminate	-1.51E+01	Indeterminate
14	p.Asp14Ser			4983	1986	5.52	3.37	-3.99E-01	Neutral	-3.99E-01	Neutral
14	p.Asp14Ile			4429	4433	4.91	7.53	-3.23E+01	Indeterminate	-3.17E+01	Indeterminate
14	p.Asp14Met			3172	2437	3.51	4.14	-2.16E+01	Indeterminate	-2.16E+01	Indeterminate
14	p.Asp14His			4685	1942	5.19	3.30	-6.67E-01	Neutral	-6.67E-01	Neutral
14	p.Asp14Gln			5284	2584	5.85	4.39	-1.64E+00	Neutral	-1.64E+00	Neutral
14	p.Asp14Pro			4429	4941	4.91	8.39	-4.17E+01	Indeterminate	-3.32E+01	Indeterminate
14	p.Asp14Leu			6098	4767	6.76	8.09	-1.16E+01	Indeterminate	-1.16E+01	Indeterminate

14	p.Asp14Asp	Synonymous		4519	1967	5.01	3.34					-1.06E+00	Neutral			-1.06E+00	Neutral
14	p.Asp14Glu			4387	2080	4.86	3.53					-1.99E+00	Neutral			-1.99E+00	Neutral
14	p.Asp14Ala			3103	1857	3.44	3.15					-1.00E+01	Indeterminate			-1.00E+01	Indeterminate
14	p.Asp14Gly			5701	2590	6.32	4.40					-8.22E-01	Neutral			-8.22E-01	Neutral
14	p.Asp14Val			5632	6061	6.24	10.29					-3.17E+01	Indeterminate			-3.13E+01	Indeterminate
14	p.Asp14Tyr			4781	4014	5.30	6.81					-1.88E+01	Indeterminate			-1.88E+01	Indeterminate
14	p.Asp14Cys			4284	1950	4.75	3.31					-1.62E+00	Neutral			-1.62E+00	Neutral
14	p.Asp14Trp			4208	3422	4.66	5.81					-1.94E+01	Indeterminate			-1.94E+01	Indeterminate
14	p.Asp14Phe			4096	3448	4.54	5.85					-2.20E+01	Indeterminate			-2.20E+01	Indeterminate
15	p.Trp15Asn			6806	1782	4.70	5.32	11345	3522	4.53	3.40	-7.59E+00	Indeterminate	-2.14E-02	Neutral	-4.97E+00	Neutral
15	p.Trp15Lys			7236	1725	5.00	5.15	13768	4227	5.49	4.09	-4.66E+00	Neutral	-6.78E-03	Neutral	-2.58E+00	Neutral
15	p.Trp15Thr			5550	1595	3.84	4.76	9633	4223	3.84	4.08	-1.35E+01	Indeterminate	-1.29E+00	Neutral	-1.13E+01	Indeterminate
15	p.Trp15Arg			5767	1195	3.99	3.57	9685	2686	3.86	2.60	-3.39E+00	Neutral	-8.64E-03	Neutral	-1.65E+00	Neutral
15	p.Trp15Ser			7958	1900	5.50	5.67	13848	4083	5.52	3.95	-4.04E+00	Neutral	-3.36E-03	Neutral	-2.11E+00	Neutral
15	p.Trp15Ile			5763	1034	3.98	3.09	9127	3846	3.64	3.72	-1.47E+00	Neutral	-1.11E+00	Neutral	-1.10E+00	Neutral
15	p.Trp15Met			5274	1033	3.64	3.08	9117	3643	3.64	3.52	-2.95E+00	Neutral	-7.47E-01	Neutral	-1.86E+00	Neutral
15	p.Trp15His			4145	1068	2.86	3.19	7396	5106	2.95	4.93	-1.30E+01	Indeterminate	-1.53E+01	Indeterminate	-2.39E+01	Indeterminate
15	p.Trp15Gln			7425	1883	5.13	5.62	12925	6246	5.16	6.04	-5.91E+00	Neutral	-1.29E+00	Neutral	-4.61E+00	Neutral
15	p.Trp15Pro			4346	1381	3.00	4.12	8773	5495	3.50	5.31	-2.33E+01	Indeterminate	-9.05E+00	Indeterminate	-2.78E+01	Indeterminate
15	p.Trp15Leu			12335	2792	8.52	8.34	22595	11054	9.01	10.68	-1.28E+00	Neutral	-3.16E-01	Neutral	-5.23E-01	Neutral
15	p.Trp15Asp			11922	2648	8.24	7.91	18631	8445	7.43	8.16	-1.22E+00	Neutral	-2.81E-01	Neutral	-4.71E-01	Neutral
15	p.Trp15Glu			10331	1960	7.14	5.85	16533	7828	6.60	7.57	-5.20E-01	Neutral	-5.96E-01	Neutral	-2.89E-01	Neutral
15	p.Trp15Ala			8846	2089	6.11	6.24	15101	4990	6.02	4.82	-3.20E+00	Neutral	-1.32E-02	Neutral	-1.52E+00	Neutral
15	p.Trp15Gly			5208	1067	3.60	3.19	9097	5665	3.63	5.48	-3.80E+00	Neutral	-8.46E+00	Indeterminate	-9.01E+00	Indeterminate
15	p.Trp15Val			4607	847	3.18	2.53	7933	2119	3.16	2.05	-2.67E+00	Neutral	-1.35E-02	Neutral	-1.17E+00	Neutral
15	p.Trp15Tyr			8482	2468	5.86	7.37	14113	5497	5.63	5.31	-8.61E+00	Indeterminate	-1.51E-01	Neutral	-5.94E+00	Indeterminate
15	p.Trp15Cys			6277	1646	4.34	4.91	12412	4374	4.95	4.23	-8.48E+00	Indeterminate	-7.78E-02	Neutral	-5.76E+00	Neutral
15	p.Trp15Trp	Synonymous		6045	1051	4.18	3.14	9713	4153	3.87	4.01	-1.06E+00	Neutral	-1.06E+00	Neutral	-8.18E-01	Neutral
15	p.Trp15Phe			10391	2331	7.18	6.96	18937	6268	7.55	6.06	-1.76E+00	Neutral	-4.10E-03	Neutral	-6.10E-01	Neutral
16	p.Leu16Asn			3055	6743	4.59	5.25					-5.32E+01	Deleterious			-5.32E+01	Deleterious
16	p.Leu16Lys			5427	16967	8.15	13.21					-5.32E+01	Deleterious			-5.32E+01	Deleterious
16	p.Leu16Thr			2209	660	3.32	0.51					-2.76E+01	Indeterminate			-2.76E+01	Indeterminate
16	p.Leu16Arg	Pathogenic		3583	13291	5.38	10.35					-5.32E+01	Deleterious			-5.32E+01	Deleterious
16	p.Leu16Ser			3934	6544	5.91	5.09					-5.32E+01	Deleterious			-5.32E+01	Deleterious
16	p.Leu16Ile			1553	269	2.33	0.21					-6.96E+00	Indeterminate			-6.96E+00	Indeterminate
16	p.Leu16Met			1995	306	3.00	0.24					-2.63E+00	Neutral			-2.63E+00	Neutral
16	p.Leu16His			4913	10696	7.38	8.33					-5.32E+01	Deleterious			-5.32E+01	Deleterious
16	p.Leu16Gln			4828	10448	7.25	8.13					-5.32E+01	Deleterious			-5.32E+01	Deleterious
16	p.Leu16Pro	Pathogenic		2946	6033	4.43	4.70					-5.32E+01	Deleterious			-5.32E+01	Deleterious
16	p.Leu16Leu	Synonymous		1846	239	2.77	0.19					-1.06E+00	Neutral			-1.06E+00	Neutral
16	p.Leu16Asp			3226	10025	4.85	7.80					-5.32E+01	Deleterious			-5.32E+01	Deleterious
16	p.Leu16Glu			4055	11396	6.09	8.87					-5.32E+01	Deleterious			-5.32E+01	Deleterious
16	p.Leu16Ala			3236	2061	4.86	1.60					-5.32E+01	Deleterious			-5.32E+01	Deleterious
16	p.Leu16Gly			4238	10949	6.37	8.52					-5.32E+01	Deleterious			-5.32E+01	Deleterious
16	p.Leu16Val			3046	804	4.58	0.63					-1.42E+01	Indeterminate			-1.42E+01	Indeterminate
16	p.Leu16Tyr			3070	8610	4.61	6.70					-5.32E+01	Deleterious			-5.32E+01	Deleterious
16	p.Leu16Cys			2604	687	3.91	0.53					-1.67E+01	Indeterminate			-1.67E+01	Indeterminate
16	p.Leu16Trp			4013	11211	6.03	8.73					-5.32E+01	Deleterious			-5.32E+01	Deleterious
16	p.Leu16Phe			2774	530	4.17	0.41					-4.61E+00	Neutral			-4.61E+00	Neutral
17	p.Ala17Asn			3109	1043	5.30	1.20					-9.84E+00	Indeterminate			-9.84E+00	Indeterminate
17	p.Ala17Lys			3328	25579	5.67	29.52					-5.32E+01	Deleterious			-5.32E+01	Deleterious
17	p.Ala17Thr			2004	476	3.41	0.55					-4.30E+00	Neutral			-4.30E+00	Neutral
17	p.Ala17Arg			3397	27882	5.79	32.18					-5.32E+01	Deleterious			-5.32E+01	Deleterious
17	p.Ala17Ser			3222	774	5.49	0.89					-1.96E+00	Neutral			-1.96E+00	Neutral
17	p.Ala17Ile			3809	1359	6.49	1.57					-9.63E+00	Indeterminate			-9.63E+00	Indeterminate
17	p.Ala17Met			1845	1285	3.14	1.48					-5.32E+01	Deleterious			-5.32E+01	Deleterious
17	p.Ala17His			2589	622	4.41	0.72					-2.96E+00	Neutral			-2.96E+00	Neutral
17	p.Ala17Gln			3620	2691	6.17	3.11					-5.32E+01	Deleterious			-5.32E+01	Deleterious
17	p.Ala17Pro			3101	714	5.28	0.82					-1.63E+00	Neutral			-1.63E+00	Neutral
17	p.Ala17Leu			2565	3353	4.37	3.87					-5.32E+01	Deleterious			-5.32E+01	Deleterious
17	p.Ala17Asp			3473	2181	5.92	2.52					-4.98E+01	Indeterminate			-3.32E+01	Indeterminate
17	p.Ala17Glu			2642	8434	4.50	9.73					-5.32E+01	Deleterious			-5.32E+01	Deleterious
17	p.Ala17Ala	Synonymous		2835	595	4.83	0.69					-1.06E+00	Neutral			-1.06E+00	Neutral
17	p.Ala17Gly			3409	720	5.81	0.83					-7.00E-01	Neutral			-7.00E-01	Neutral
17	p.Ala17Val			3266	716	5.56	0.83					-1.04E+00	Neutral			-1.04E+00	Neutral
17	p.Ala17Tyr			2603	2585	4.43	2.98					-5.32E+01	Deleterious			-5.32E+01	Deleterious
17	p.Ala17Cys			1754	279	2.99	0.32					-4.10E-01	Neutral			-4.10E-01	Neutral
17	p.Ala17Trp			2913	3306	4.96	3.82					-5.32E+01	Deleterious			-5.32E+01	Deleterious
17	p.Ala17Phe			3210	2051	5.47	2.37					-5.32E+01	Deleterious			-5.32E+01	Deleterious
18	p.Thr18Asn			1065	1200	4.51	5.39					-4.09E+01	Indeterminate			-3.32E+01	Indeterminate
18	p.Thr18Lys			1322	1076	5.60	4.83					-1.40E+01	Indeterminate			-1.40E+01	Indeterminate
18	p.Thr18Thr	Synonymous		1088	483	4.61	2.17					-1.06E+00	Neutral			-1.06E+00	Neutral
18	p.Thr18Arg			1372	1100	5.81	4.94					-1.28E+01	Indeterminate			-1.28E+01	Indeterminate
18	p.Thr18Ser			1365	878	5.78	3.94					-5.57E+00	Neutral			-5.57E+00	Neutral
18	p.Thr18Ile			921	852	3.90	3.82					-2.85E+01	Indeterminate			-2.85E+01	Indeterminate
18	p.Thr18Met			1963	2078	8.31	9.33					-2.08E+01	Indeterminate			-2.08E+01	Indeterminate
18	p.Thr18His			697	420	2.95	1.89					-1.04E+01	Indeterminate			-1.04E+01	Indeterminate
18	p.Thr18Gln			1320	973	5.59	4.37					-1.00E+01	Indeterminate			-1.00E+01	Indeterminate
18	p.Thr18Pro		Deleterious	762	3836	3.23	17.22					-5.32E+01	Deleterious			-5.32E+01	Deleterious
18	p.Thr18Leu			1397	1503	5.92	6.75					-2.94E+01	Indeterminate			-2.93E+01	Indeterminate
18	p.Thr18Asp			1125	829	4.76	3.72					-1.20E+01	Indeterminate			-1.20E+01	Indeterminate
18	p.Thr18Glu			1271	825	5.38	3.70					-6.41E+00	Indeterminate			-6.41E+00	Indeterminate
18	p.Thr18Ala			1230	1342	5.21	6.02					-3.39E+01	Indeterminate			-3.25E+01	Indeterminate
18	p.Thr18Gly			842	544	3.57	2.44					-1.06E+01	Indeterminate			-1.06E+01	Indeterminate
18	p.Thr18Val			1693	1130	7.17	5.07					-4.77E+00	Neutral			-4.77E+00	Neutral
18	p.Thr18Tyr			785	884	3.32	3.97					-5.10E+01	Indeterminate			-3.32E+01	Indeterminate
18	p.Thr18Cys			1182	574	5.01	2.58					-1.62E+00	Neutral			-1.62E+00	Neutral
18	p.Thr18Trp			1679	1410	7.11	6.33					-1.19E+01	Indeterminate			-1.19E+01	Indeterminate
18	p.Thr18Phe			533	340	2.26	1.53					-1.68E+01	Indeterminate			-1.68E+01	Indeterminate
19	p.Ala19Asn			2103	1690	6.11	2.62					-7.89E-08	Neutral			-7.87E-08	Neutral
19	p.Ala19Lys			1123	2217	3.26	3.44					-2.42E+00	Neutral			-2.42E+00	Neutral
19	p.Ala19Thr			1850	2296	5.38	3.56					-2.73E-03	Neutral			-2.73E-03	Neutral
19	p.Ala19Arg			1712	3057	4.98	4.75					-4.32E-01	Neutral			-4.32E-01	Neutral
19	p.Ala19Ser			1700	1327	4.94	2.06					-2.29E-07	Neutral			-2.29E-07	Neutral
19	p.Ala19Ile			2099	2243	6.10	3.48					-6.78E-05	Neutral			-6.78E-05	Neutral
19	p.Ala19Met			1357	1021	3.94	1.59					-5.92E-07	Neutral			-5.92E-07	Neutral
19	p.Ala19His			2393	3205	6.96	4.98					-2.33E-03	Neutral			-2.33E-03	Neutral
19	p.Ala19Gln			1314	1013	3.82	1.57					-1.39E-06	Neutral			-1.39E-06	Neutral
19	p.Ala19Pro			2107	27833	6.12	43.22					-5.32E+01	Deleterious			-5.32E+01	Deleterious
19	p.Ala19Leu			1886	1318	5.48	2.05					-5.00E-09	Neutral			-4.86E-09	Neutral
19	p.Ala19Asp			1341	2749	3.90	4.27					-2.14E+00	Neutral			-2.14E+00	Neutral
19	p.Ala19Glu			2092	1938	6.08	3.01					-2.81E-06	Neutral			-2.81E-06	Neutral
19	p.Ala19Ala	Synonymous		1342	2472	3.90	3.84					-1.06E+00	Neutral			-1.06E+00	Neutral
19	p.Ala19Gly			1446	1223	4.20	1.90					-5.89E-06	Neutral			-5.89E-06	Neutral
19	p.Ala19Val			1157	1160	3.36	1.80					-9.74E-04	Neutral			-9.74E-04	Neutral
19	p.Ala19Tyr			1572	1657	4.57	2.57					-3.63E-04	Neutral			-3.63E-04	Neutral
19	p.Ala19Cys			1675	3514	4.87	5.46					-1.56E+00	Neutral			-1.56E+00	Neutral
19	p.Ala19Trp			1976	1448	5.74	2.25					-1.19E-08	Neutral			-1.18E-08	Neutral
19	p.Ala19Phe			2158	1024	6.27	1.59					-1.28E-14	Neutral			0.00E+00	Neutral
20	p.Ala20Asn			9582	6012	5.43	6.63	5392	5033	5.32	6.92	-5.32E+01	Deleterious	-5.32E+01	Deleterious	-5.32E+01	Deleterious
20	p.Ala20Lys			13641	10495	7.72	11.57	7118	8147	7.02	11.21	-5.32E+01	Deleterious	-5.32E+01	Deleterious	-5.32E+01	Deleterious
20	p.Ala20Thr			6922	432	3.92	0.48	4653	296	4.59	0.41	-1.61E+01	Indeterminate	-1.01E+01	Indeterminate	-2.19E+01	Indeterminate
20	p.Ala20Arg			8181	5927	4.63	6.54	4101	4835	4.05	6.65	-5.32E+01	Deleterious	-5.32E+01	Deleterious	-5.32E+01	Deleterious
20	p.Ala20Ser			4887	170	2.77	0.19	3069	235	3.03	0.32	-2.25E+00	Neutral	-2.71E+01	Indeterminate	-2.49E+01	Indeterminate
20	p.Ala20Ile			7212	2564	4.08	2.83	4277	1841	4.22	2.53	-5.32E+01	Deleterious	-5.32E+01	Deleterious	-5.32E+01	Deleterious
20	p.Ala20Met			8089	2111	4.58	2.33	4693	1696	4.63	2.33	-5.32E+01	Deleterious	-5.32E+01	Deleterious	-5.32E+01	Deleterious
20	p.Ala20His			6923	4925	3.92	5.43	3697	4261	3.65	5.86	-5.32E+01	Deleterious	-5.32E+01	Deleterious	-5.32E+01	Deleterious
20	p.Ala20Gln			10148	7891	5.75	8.70	5584	6254	5.51	8.60	-5.32E+01	Deleterious	-5.32E+01	Deleterious	-5.32E+01	Deleterious
20	p.Ala20Pro		Deleterious	9331	7379	5.28	8.14	5105	5579	5.04	7.67	-5.32E+01	Deleterious	-5.32E+01	Deleterious	-5.32E+01	Deleterious
20	p.Ala20Leu			9121	2392	5.16	2.64	5598	1953	5.52	2.69	-5.32E+01	Deleterious	-5.32E+01	Deleterious	-5.32E+01	Deleterious
20	p.Ala20Asp			10217	7096	5.78	7.82	5645	6060	5.57	8.33	-5.32E+01	Deleterious	-5.32E+01	Deleterious	-5.32E+01	Deleterious
20	p.Ala20Glu			12616	9358	7.14	10.32	6694	7998	6.60	11.00	-5.32E+01	Deleterious	-5.32E+01	Deleterious	-5.32E+01	Deleterious
20	p.Ala20Ala	Synonymous		8085	296	4.58	0.33	5293	225	5.22	0.31	-1.06E+00	Neutral	-1.06E+00	Neutral	-8.18E-01	Neutral
20	p.Ala20Gly		Deleterious	7764	1542	4.40	1.70	5051	413	4.98	0.57	-5.32E+01	Deleterious	-2.06E+01	Indeterminate	-5.32E+01	Deleterious
20	p.Ala20Val			7744	325	4.38	0.36	5039	329	4.97	0.45	-2.74E+00	Neutral	-1.01E+01	Indeterminate	-9.53E+00	Indeterminate
20	p.Ala20Tyr			8424	6722	4.77	7.41	4712	5356	4.65	7.37	-5.32E+01	Deleterious	-5.32E+01	Deleterious	-5.32E+01	Deleterious
20	p.Ala20Cys			8337	361	4.72	0.40	5338	304	5.27	0.42	-2.84E+00	Neutral	-5.43E+00	Neutral	-5.52E+00	Neutral
20	p.Ala20Trp			9412	7408	5.33	8.17	4993	5865	4.93	8.07	-5.32E+01	Deleterious	-5.32E+01	Deleterious	-5.32E+01	Deleterious
20	p.Ala20Phe			9980	7279	5.65	8.03	5306	6026	5.23	8.29	-5.32E+01	Deleterious	-5.32E+01	Deleterious	-5.32E+01	Deleterious
21	p.Ala21Asn			1371	1131	2.99	1.73					-5.32E+01	Deleterious			-5.32E+01	Deleterious
21	p.Ala21Lys			2163	10988	4.72	16.83					-5.32E+01	Deleterious			-5.32E+01	Deleterious
21	p.Ala21Thr			1828	453	3.99	0.69					-9.18E+00	Indeterminate			-9.18E+00	Indeterminate
21	p.Ala21Arg			1481	2986	3.23	4.57					-5.32E+01	Deleterious			-5.32E+01	Deleterious
21	p.Ala21Ser			1688	297	3.68	0.45					-2.09E+00	Neutral			-2.09E+00	Neutral
21	p.Ala21Ile			1041	426	2.27	0.65					-5.32E+01	Deleterious			-5.32E+01	Deleterious
21	p.Ala21Met			2168	1207	4.73	1.85					-5.32E+01	Deleterious			-5.32E+01	Deleterious
21	p.Ala21His			3495	5459	7.62	8.36					-5.32E+01	Deleterious			-5.32E+01	Deleterious
21	p.Ala21Gln			3485	3708	7.60	5.68					-5.32E+01	Deleterious			-5.32E+01	Deleterious
21	p.Ala21Pro			2404	15276	5.24	23.40					-5.32E+01	Deleterious			-5.32E+01	Deleterious

21	p.Ala21Leu		3758	1034	8.19	1.58					-5.31E+00	Neutral			-5.31E+00	Neutral
21	p.Ala21Asp		2436	9530	5.31	14.60					-5.32E+01	Deleterious			-5.32E+01	Deleterious
21	p.Ala21Glu		1636	2674	3.57	4.10					-5.32E+01	Deleterious			-5.32E+01	Deleterious
21	p.Ala21Ala	Synonymous	1979	331	4.31	0.51					-1.06E+00	Neutral			-1.06E+00	Neutral
21	p.Ala21Gly		2395	390	5.22	0.60					-5.23E-01	Neutral			-5.23E-01	Neutral
21	p.Ala21Val		2784	547	6.07	0.84					-1.47E+00	Neutral			-1.47E+00	Neutral
21	p.Ala21Tyr		2718	3295	5.93	5.05					-5.32E+01	Deleterious			-5.32E+01	Deleterious
21	p.Ala21Cys		2659	468	5.80	0.72					-7.45E-01	Neutral			-7.45E-01	Neutral
21	p.Ala21Trp		2399	3082	5.23	4.72					-5.32E+01	Deleterious			-5.32E+01	Deleterious
21	p.Ala21Phe		1984	1999	4.33	3.06					-5.32E+01	Deleterious			-5.32E+01	Deleterious
22	p.Arg22Asn		1177	1020	8.01	2.54					-1.24E-07	Neutral			-1.24E-07	Neutral
22	p.Arg22Lys		1049	2391	7.14	5.96					-1.44E+00	Neutral			-1.44E+00	Neutral
22	p.Arg22Thr		1153	5650	7.85	14.09					-2.77E+01	Indeterminate			-2.77E+01	Indeterminate
22	p.Arg22Arg	Synonymous	1233	2837	8.39	7.08					-1.06E+00	Neutral			-1.06E+00	Neutral
22	p.Arg22Ser		473	1630	3.22	4.07					-2.39E+01	Indeterminate			-2.39E+01	Indeterminate
22	p.Arg22Ile		819	1627	5.58	4.06					-9.80E-01	Neutral			-9.80E-01	Neutral
22	p.Arg22Met		1029	3315	7.01	8.27					-8.42E+00	Indeterminate			-8.42E+00	Indeterminate
22	p.Arg22His		338	167	2.30	0.42					-2.42E-09	Neutral			-2.28E-09	Neutral
22	p.Arg22Gln		1275	1139	8.68	2.84					-1.37E-07	Neutral			-1.37E-07	Neutral
22	p.Arg22Pro		424	6555	2.89	16.35					-5.32E+01	Deleterious			-5.32E+01	Deleterious
22	p.Arg22Leu		617	764	4.20	1.91					-1.65E-02	Neutral			-1.65E-02	Neutral
22	p.Arg22Asp		324	671	2.21	1.67					-6.69E+00	Indeterminate			-6.69E+00	Indeterminate
22	p.Arg22Glu		737	522	5.02	1.30					-3.73E-08	Neutral			-3.72E-08	Neutral
22	p.Arg22Ala		310	287	2.11	0.72					-6.27E-03	Neutral			-6.27E-03	Neutral
22	p.Arg22Gly		323	2465	2.20	6.15					-5.32E+01	Deleterious			-5.32E+01	Deleterious
22	p.Arg22Val		312	367	2.12	0.92					-1.56E-01	Neutral			-1.56E-01	Neutral
22	p.Arg22Tyr		596	1333	4.06	3.32					-3.79E+00	Neutral			-3.79E+00	Neutral
22	p.Arg22Cys		639	324	4.35	0.81					-1.16E-11	Neutral			0.00E+00	Neutral
22	p.Arg22Trp		1319	4784	8.98	11.93					-9.64E+00	Indeterminate			-9.64E+00	Indeterminate
22	p.Arg22Phe		542	2250	3.69	5.61					-3.42E+01	Indeterminate			-3.26E+01	Indeterminate
23	p.Gly23Asn		1971	217	4.60	0.67					-2.16E+01	Indeterminate			-2.16E+01	Indeterminate
23	p.Gly23Lys		2291	2054	5.34	6.32					-5.32E+01	Deleterious			-5.32E+01	Deleterious
23	p.Gly23Thr		2549	2313	5.94	7.12					-5.32E+01	Deleterious			-5.32E+01	Deleterious
23	p.Gly23Arg	Likely pathogenic	2176	1816	5.07	5.59					-5.32E+01	Deleterious			-5.32E+01	Deleterious
23	p.Gly23Ser	Likely pathogenic	1609	285	3.75	0.88					-5.32E+01	Deleterious			-5.32E+01	Deleterious
23	p.Gly23Ile		2654	2752	6.19	8.47					-5.32E+01	Deleterious			-5.32E+01	Deleterious
23	p.Gly23Met		2276	2025	5.31	6.23					-5.32E+01	Deleterious			-5.32E+01	Deleterious
23	p.Gly23His		2587	2185	6.03	6.72					-5.32E+01	Deleterious			-5.32E+01	Deleterious
23	p.Gly23Gln		2634	2152	6.14	6.62					-5.32E+01	Deleterious			-5.32E+01	Deleterious
23	p.Gly23Pro		2042	1771	4.76	5.45					-5.32E+01	Deleterious			-5.32E+01	Deleterious
23	p.Gly23Leu		724	730	1.69	2.25					-5.32E+01	Deleterious			-5.32E+01	Deleterious
23	p.Gly23Asp	Pathogenic	1669	1066	3.89	3.28					-5.32E+01	Deleterious			-5.32E+01	Deleterious
23	p.Gly23Glu		2812	2666	6.56	8.20					-5.32E+01	Deleterious			-5.32E+01	Deleterious
23	p.Gly23Ala		1966	112	4.58	0.34					-1.29E+00	Neutral			-1.29E+00	Neutral
23	p.Gly23Gly	Synonymous	1029	46	2.40	0.14					-1.06E+00	Neutral			-1.06E+00	Neutral
23	p.Gly23Val		2948	2588	6.87	7.96					-5.32E+01	Deleterious			-5.32E+01	Deleterious
23	p.Gly23Tyr		1763	1578	4.11	4.85					-5.32E+01	Deleterious			-5.32E+01	Deleterious
23	p.Gly23Cys		2210	1286	5.15	3.96					-5.32E+01	Deleterious			-5.32E+01	Deleterious
23	p.Gly23Trp		2196	2273	5.12	6.99					-5.32E+01	Deleterious			-5.32E+01	Deleterious
23	p.Gly23Phe		2787	2593	6.50	7.98					-5.32E+01	Deleterious			-5.32E+01	Deleterious
24	p.Arg24Asn		2933	1156	4.19	2.75	7521	6903	4.31	3.38	-9.14E-02	Neutral	-1.70E+00	Neutral	-6.25E-01	Neutral
24	p.Arg24Lys		3506	1119	5.01	2.66	8038	8566	4.61	4.19	-1.60E-03	Neutral	-3.49E+00	Neutral	-1.72E+00	Neutral
24	p.Arg24Thr		3180	1226	4.54	2.92	6972	8076	4.00	3.95	-4.97E-02	Neutral	-6.38E+00	Neutral	-3.98E+00	Neutral
24	p.Arg24Arg	Synonymous	3351	1793	4.79	4.27	8843	7990	5.07	3.91	-1.06E+00	Neutral	-1.06E+00	Neutral	-8.18E-01	Neutral
24	p.Arg24Ser		3650	1132	5.21	2.69	8814	7806	5.06	3.82	-7.40E-04	Neutral	-9.24E-01	Neutral	-2.10E-01	Neutral
24	p.Arg24Ile		2629	1067	3.76	2.54	6448	5232	3.70	2.56	-1.91E-01	Neutral	-1.04E+00	Neutral	-3.42E-01	Neutral
24	p.Arg24Met		3751	1815	5.36	4.32	9977	9768	5.72	4.78	-3.40E-01	Neutral	-1.40E+00	Neutral	-6.00E-01	Neutral
24	p.Arg24His		3570	1006	5.10	2.39	9360	7018	5.37	3.44	-1.29E-04	Neutral	-1.72E-01	Neutral	-9.56E-03	Neutral
24	p.Arg24Gln		3595	2952	5.14	7.02	8545	12341	4.90	6.04	-8.69E+00	Indeterminate	-1.14E+01	Indeterminate	-1.62E+01	Indeterminate
24	p.Arg24Pro	Pathogenic	2814	13992	4.02	33.29	7157	46361	4.11	22.70	-5.32E+01	Deleterious	-5.32E+01	Deleterious	-5.32E+01	Deleterious
24	p.Arg24Leu		2863	1249	4.09	2.97	7372	7029	4.23	3.44	-2.98E-01	Neutral	-2.23E+00	Neutral	-1.07E+00	Neutral
24	p.Arg24Asp		4737	1883	6.77	4.48	12409	9485	7.12	4.64	-1.18E-02	Neutral	-7.32E-02	Neutral	-2.41E-03	Neutral
24	p.Arg24Glu		3650	1301	5.21	3.09	8804	9043	5.05	4.43	-8.28E-03	Neutral	-2.43E+00	Neutral	-1.01E+00	Neutral
24	p.Arg24Ala		3165	902	4.52	2.15	8126	6212	4.66	3.04	-3.62E-04	Neutral	-3.36E-01	Neutral	-3.41E-02	Neutral
24	p.Arg24Gly		2844	1050	4.06	2.50	7234	8057	4.15	3.94	-4.58E-02	Neutral	-5.10E+00	Neutral	-2.96E+00	Neutral
24	p.Arg24Val		4782	1725	6.83	4.10	11195	10279	6.42	5.03	-2.26E-03	Neutral	-6.60E-01	Neutral	-1.17E-01	Neutral
24	p.Arg24Tyr		3352	1179	4.79	2.80	8524	8763	4.89	4.29	-1.05E-02	Neutral	-2.60E+00	Neutral	-1.12E+00	Neutral
24	p.Arg24Cys		2826	1270	4.04	3.02	6942	4683	3.98	2.29	-4.09E-01	Neutral	-1.61E-01	Neutral	-8.97E-02	Neutral
24	p.Arg24Trp		4945	2525	7.06	6.01	11886	12036	6.82	5.89	-2.34E-01	Neutral	-1.19E+00	Neutral	-4.32E-01	Neutral
24	p.Arg24Phe		3862	1694	5.52	4.03	10145	8607	5.82	4.21	-1.10E-01	Neutral	-4.45E-01	Neutral	-8.53E-02	Neutral
25	p.Val25Asn		8478	1522	4.84	3.94					-4.24E+00	Neutral			-4.24E+00	Neutral
25	p.Val25Lys		9210	2493	5.25	6.45					-1.78E+01	Indeterminate			-1.78E+01	Indeterminate
25	p.Val25Thr		8108	1182	4.62	3.06					-1.42E+00	Neutral			-1.42E+00	Neutral
25	p.Val25Arg		13506	2689	7.70	6.96					-3.28E+00	Neutral			-3.28E+00	Neutral
25	p.Val25Ser		8701	1111	4.96	2.87					-4.26E-01	Neutral			-4.26E-01	Neutral
25	p.Val25Ile		6987	845	3.98	2.19					-5.01E-01	Neutral			-5.01E-01	Neutral
25	p.Val25Met		10879	3296	6.20	8.53					-2.10E+01	Indeterminate			-2.10E+01	Indeterminate
25	p.Val25His		7159	812	4.08	2.10					-2.56E-01	Neutral			-2.56E-01	Neutral
25	p.Val25Gln		5328	592	3.04	1.53					-5.25E-01	Neutral			-5.25E-01	Neutral
25	p.Val25Pro		4054	267	2.31	0.69					-1.91E-03	Neutral			-1.91E-03	Neutral
25	p.Val25Leu		4540	391	2.59	1.01					-6.34E-02	Neutral			-6.34E-02	Neutral
25	p.Val25Asp		11884	3484	6.78	9.01					-1.76E+01	Indeterminate			-1.76E+01	Indeterminate
25	p.Val25Glu		11601	1495	6.62	3.87					-1.83E-01	Neutral			-1.83E-01	Neutral
25	p.Val25Ala		6216	5757	3.55	14.90					-5.32E+01	Deleterious			-5.32E+01	Deleterious
25	p.Val25Gly		10784	3197	6.15	8.27					-1.99E+01	Indeterminate			-1.99E+01	Indeterminate
25	p.Val25Val	Synonymous	9182	1337	5.24	3.46					-1.06E+00	Neutral			-1.06E+00	Neutral
25	p.Val25Tyr		9422	3948	5.37	10.21					-5.20E+01	Indeterminate			-3.32E+01	Indeterminate
25	p.Val25Cys		9741	871	5.56	2.25					-2.47E-03	Neutral			-2.47E-03	Neutral
25	p.Val25Trp		11563	2037	6.59	5.27					-2.22E+00	Neutral			-2.22E+00	Neutral
25	p.Val25Phe		7991	1324	4.56	3.43					-3.13E+00	Neutral			-3.13E+00	Neutral
26	p.Glu26Asn		2618	1398	2.56	2.14					-1.41E+00	Neutral			-1.41E+00	Neutral
26	p.Glu26Lys		4162	2229	4.06	3.42					-4.39E-01	Neutral			-4.39E-01	Neutral
26	p.Glu26Thr		1175	706	1.15	1.08					-9.37E+00	Indeterminate			-9.37E+00	Indeterminate
26	p.Glu26Arg		8484	4992	8.28	7.65					-1.02E-01	Neutral			-1.02E-01	Neutral
26	p.Glu26Ser		903	705	0.88	1.08					-2.74E+01	Indeterminate			-2.74E+01	Indeterminate
26	p.Glu26Ile		6233	3893	6.09	5.97					-5.21E-01	Neutral			-5.21E-01	Neutral
26	p.Glu26Met		5772	3272	5.64	5.02					-2.70E-01	Neutral			-2.70E-01	Neutral
26	p.Glu26His		3306	2308	3.23	3.54					-4.15E+00	Neutral			-4.15E+00	Neutral
26	p.Glu26Gln		4189	2887	4.09	4.43					-2.58E+00	Neutral			-2.58E+00	Neutral
26	p.Glu26Pro		9408	6512	9.19	9.98					-3.86E-01	Neutral			-3.86E-01	Neutral
26	p.Glu26Leu		4257	2348	4.16	3.60					-5.32E-01	Neutral			-5.32E-01	Neutral
26	p.Glu26Asp		3303	2162	3.23	3.31					-3.00E+00	Neutral			-3.00E+00	Neutral
26	p.Glu26Glu	Synonymous	5487	3596	5.36	5.51					-1.06E+00	Neutral			-1.06E+00	Neutral
26	p.Glu26Ala		5080	3561	4.96	5.46					-1.96E+00	Neutral			-1.96E+00	Neutral
26	p.Glu26Gly		2672	1759	2.61	2.70					-4.39E+00	Neutral			-4.39E+00	Neutral
26	p.Glu26Val		9172	5895	8.96	9.04					-2.03E-01	Neutral			-2.03E-01	Neutral
26	p.Glu26Tyr		3224	2365	3.15	3.63					-5.42E+00	Neutral			-5.42E+00	Neutral
26	p.Glu26Cys		14018	8717	13.69	13.36					-2.39E-02	Neutral			-2.39E-02	Neutral
26	p.Glu26Trp		5619	3660	5.49	5.61					-9.58E-01	Neutral			-9.58E-01	Neutral
26	p.Glu26Phe		3324	2258	3.25	3.46					-3.59E+00	Neutral			-3.59E+00	Neutral
27	p.Glu27Asn		3049	116	1.35	0.54	2068	545	1.19	1.12	-1.15E-02	Neutral	-1.91E+01	Indeterminate	-1.53E+01	Indeterminate
27	p.Glu27Lys		23016	2287	10.17	10.63	16797	4397	9.63	9.04	-3.85E-01	Neutral	-6.79E-01	Neutral	-2.67E-01	Neutral
27	p.Glu27Thr		9777	1746	4.32	8.12	7364	2199	4.22	4.52	-2.54E+01	Indeterminate	-6.92E+00	Indeterminate	-2.78E+01	Indeterminate
27	p.Glu27Arg		9627	961	4.26	4.47	7549	2188	4.33	4.50	-3.03E+00	Neutral	-5.90E+00	Neutral	-6.08E+00	Indeterminate
27	p.Glu27Ser		7381	589	3.26	2.74	6110	1601	3.50	3.29	-1.34E+00	Neutral	-5.15E+00	Neutral	-4.04E+00	Neutral
27	p.Glu27Ile		12256	855	5.42	3.97	9316	2786	5.34	5.73	-8.34E-02	Neutral	-4.97E+00	Neutral	-2.89E+00	Neutral
27	p.Glu27Met		13701	1347	6.06	6.26	10283	2849	5.89	5.85	-1.37E+00	Neutral	-2.90E+00	Neutral	-2.28E+00	Neutral
27	p.Glu27His		11903	1119	5.26	5.20	8714	2582	5.00	5.31	-1.39E+00	Neutral	-5.27E+00	Neutral	-4.17E+00	Neutral
27	p.Glu27Gln		12459	1195	5.51	5.56	9245	2630	5.30	5.40	-1.44E+00	Neutral	-3.98E+00	Neutral	-3.17E+00	Neutral
27	p.Glu27Pro		14286	1751	6.31	8.14	11214	3736	6.43	7.68	-4.40E+00	Neutral	-6.10E+00	Neutral	-7.45E+00	Indeterminate
27	p.Glu27Leu		11139	661	4.92	3.07	8014	2183	4.59	4.49	-1.43E-02	Neutral	-4.06E+00	Neutral	-2.14E+00	Neutral
27	p.Glu27Asp		4309	353	1.90	1.64	3024	1006	1.73	2.07	-4.30E+00	Neutral	-2.57E+01	Indeterminate	-2.56E+01	Indeterminate
27	p.Glu27Glu	Synonymous	18925	1996	8.37	9.28	14627	3878	8.38	7.97	-1.06E+00	Neutral	-1.06E+00	Neutral	-8.18E-01	Neutral
27	p.Glu27Ala		10932	963	4.83	4.48	8227	2282	4.72	4.69	-1.08E+00	Neutral	-4.24E+00	Neutral	-3.09E+00	Neutral
27	p.Glu27Gly		3311	311	1.46	1.45	3177	813	1.82	1.67	-1.07E+01	Indeterminate	-1.11E+01	Indeterminate	-1.78E+01	Indeterminate
27	p.Glu27Val		18682	1607	8.26	7.47	14601	4170	8.37	8.57	-1.84E-01	Neutral	-1.76E+00	Neutral	-7.10E-01	Neutral
27	p.Glu27Tyr		4149	804	1.83	3.74	3759	949	2.15	1.95	-5.32E+01	Deleterious	-8.60E+00	Indeterminate	-5.32E+01	Deleterious
27	p.Glu27Cys		4188	379	1.85	1.76	3656	1088	2.10	2.24	-6.94E+00	Indeterminate	-1.56E+01	Indeterminate	-1.85E+01	Indeterminate
27	p.Glu27Trp		18527	1531	8.19	7.12	13839	3589	7.93	7.38	-1.22E-01	Neutral	-1.03E+00	Neutral	-3.07E-01	Neutral
27	p.Glu27Phe		14611	941	6.46	4.37	12869	3189	7.38	6.55	-1.26E-02	Neutral	-8.83E-01	Neutral	-1.99E-01	Neutral
28	p.Val28Asn		9727	11536	4.96	7.12					-5.32E+01	Deleterious			-5.32E+01	Deleterious
28	p.Val28Lys		9389	15308	4.79	9.45					-5.32E+01	Deleterious			-5.32E+01	Deleterious
28	p.Val28Thr		9288	1619	4.74	1.00					-1.58E-01	Neutral			-1.58E-01	Neutral
28	p.Val28Arg		8484	12431	4.33	7.67					-5.32E+01	Deleterious			-5.32E+01	Deleterious
28	p.Val28Ser		11984	2938	6.12	1.81					-1.43E+00	Neutral			-1.43E+00	Neutral
28	p.Val28Ile		10439	1906	5.33	1.18					-1.71E-01	Neutral			-1.71E-01	Neutral
28	p.Val28Met		7038	1186	3.59	0.73					-2.88E-01	Neutral			-2.88E-01	Neutral
28	p.Val28His		7337	11305	3.74	6.98					-5.32E+01	Deleterious			-5.32E+01	Deleterious
28	p.Val28Gln		9411	9712	4.80	5.99					-5.32E+01	Deleterious			-5.32E+01	Deleterious

28	p.Val28Pro			8623	12625	4.40	7.79					-5.32E+01	Deleterious			-5.32E+01	Deleterious
28	p.Val28Leu			11339	1580	5.79	0.98					-2.63E-03	Neutral			-2.63E-03	Neutral
28	p.Val28Asp			10547	15278	5.38	9.43					-5.32E+01	Deleterious			-5.32E+01	Deleterious
28	p.Val28Glu			12987	12241	6.63	7.56					-5.32E+01	Deleterious			-5.32E+01	Deleterious
28	p.Val28Ala			11044	1828	5.64	1.13					-4.21E-02	Neutral			-4.21E-02	Neutral
28	p.Val28Gly			8378	3399	4.28	2.10					-2.04E+01	Indeterminate			-2.04E+01	Indeterminate
28	p.Val28Val	Synonymous		11421	2640	5.83	1.63					-1.06E+00	Neutral			-1.06E+00	Neutral
28	p.Val28Tyr			8913	13513	4.55	8.34					-5.32E+01	Deleterious			-5.32E+01	Deleterious
28	p.Val28Cys			8264	1344	4.22	0.83					-1.12E-01	Neutral			-1.12E-01	Neutral
28	p.Val28Trp			11572	19076	5.91	11.77					-5.32E+01	Deleterious			-5.32E+01	Deleterious
28	p.Val28Phe			9745	10542	4.97	6.51					-5.32E+01	Deleterious			-5.32E+01	Deleterious
29	p.Arg29Asn			20602	4626	6.41	6.71					-4.64E+00	Neutral			-4.64E+00	Neutral
29	p.Arg29Lys			20053	2816	6.24	4.08					-1.73E-01	Neutral			-1.73E-01	Neutral
29	p.Arg29Thr			12052	2884	3.75	4.18					-1.21E+01	Indeterminate			-1.21E+01	Indeterminate
29	p.Arg29Arg	Synonymous		17229	2824	5.36	4.09					-1.06E+00	Neutral			-1.06E+00	Neutral
29	p.Arg29Ser			32631	6359	10.16	9.22					-7.93E-01	Neutral			-7.93E-01	Neutral
29	p.Arg29Ile			15556	4861	4.84	7.05					-2.12E+01	Indeterminate			-2.12E+01	Indeterminate
29	p.Arg29Met			16707	2888	5.20	4.19					-1.63E+00	Neutral			-1.63E+00	Neutral
29	p.Arg29His			7538	1143	2.35	1.66					-3.49E+00	Neutral			-3.49E+00	Neutral
29	p.Arg29Gln			19103	2763	5.95	4.01					-2.77E-01	Neutral			-2.77E-01	Neutral
29	p.Arg29Pro			4160	10588	1.30	15.35					-5.32E+01	Deleterious			-5.32E+01	Deleterious
29	p.Arg29Leu			19150	3168	5.96	4.59					-8.77E-01	Neutral			-8.77E-01	Neutral
29	p.Arg29Asp			17057	5258	5.31	7.62					-1.87E+01	Indeterminate			-1.87E+01	Indeterminate
29	p.Arg29Glu			13773	2739	4.29	3.97					-4.92E+00	Neutral			-4.92E+00	Neutral
29	p.Arg29Ala			17777	1898	5.53	2.75					-7.71E-03	Neutral			-7.71E-03	Neutral
29	p.Arg29Gly			19312	3544	6.01	5.14					-1.76E+00	Neutral			-1.76E+00	Neutral
29	p.Arg29Val			10545	1606	3.28	2.33					-1.92E+00	Neutral			-1.92E+00	Neutral
29	p.Arg29Tyr			18295	2604	5.70	3.77					-2.72E-01	Neutral			-2.72E-01	Neutral
29	p.Arg29Cys			22937	4095	7.14	5.94					-9.90E-01	Neutral			-9.90E-01	Neutral
29	p.Arg29Trp			9347	1224	2.91	1.77					-9.07E-01	Neutral			-9.07E-01	Neutral
29	p.Arg29Phe			7409	1098	2.31	1.59					-3.19E+00	Neutral			-3.19E+00	Neutral
30	p.Ala30Asn			21267	13488	5.27	11.35					-5.32E+01	Deleterious			-5.32E+01	Deleterious
30	p.Ala30Lys			17443	2724	4.32	2.29					-1.25E+00	Neutral			-1.25E+00	Neutral
30	p.Ala30Thr			24071	8424	5.96	7.09					-2.40E+01	Indeterminate			-2.40E+01	Indeterminate
30	p.Ala30Arg			26854	5725	6.65	4.82					-3.40E+00	Neutral			-3.40E+00	Neutral
30	p.Ala30Ser			20268	5206	5.02	4.38					-1.11E+01	Indeterminate			-1.11E+01	Indeterminate
30	p.Ala30Ile			18034	4333	4.47	3.64					-1.00E+01	Indeterminate			-1.00E+01	Indeterminate
30	p.Ala30Met			27133	6428	6.72	5.41					-5.52E+00	Neutral			-5.52E+00	Neutral
30	p.Ala30His			18096	3956	4.48	3.33					-6.94E+00	Indeterminate			-6.94E+00	Indeterminate
30	p.Ala30Gln			18462	4653	4.57	3.91					-1.15E+01	Indeterminate			-1.15E+01	Indeterminate
30	p.Ala30Pro			18318	9216	4.54	7.75					-5.32E+01	Deleterious			-5.32E+01	Deleterious
30	p.Ala30Leu			14439	3198	3.58	2.69					-9.74E+00	Indeterminate			-9.74E+00	Indeterminate
30	p.Ala30Asp			11706	4156	2.90	3.50					-4.46E+01	Indeterminate			-3.32E+01	Indeterminate
30	p.Ala30Glu			21369	3913	5.30	3.29					-2.24E+00	Neutral			-2.24E+00	Neutral
30	p.Ala30Ala	Synonymous		25468	4405	6.31	3.71					-1.06E+00	Neutral			-1.06E+00	Neutral
30	p.Ala30Gly			17570	10117	4.35	8.51					-5.32E+01	Deleterious			-5.32E+01	Deleterious
30	p.Ala30Val			33236	8979	8.24	7.55					-7.22E+00	Indeterminate			-7.22E+00	Indeterminate
30	p.Ala30Tyr			16045	5032	3.98	4.23					-2.56E+01	Indeterminate			-2.56E+01	Indeterminate
30	p.Ala30Cys			15100	4355	3.74	3.66					-2.15E+01	Indeterminate			-2.15E+01	Indeterminate
30	p.Ala30Trp			26402	7996	6.54	6.73					-1.43E+01	Indeterminate			-1.43E+01	Indeterminate
30	p.Ala30Phe			12276	2580	3.04	2.17					-9.80E+00	Indeterminate			-9.80E+00	Indeterminate
31	p.Leu31Asn			17658	3677	4.70	3.62					-1.58E+01	Indeterminate			-1.58E+01	Indeterminate
31	p.Leu31Lys			22252	3043	5.93	3.00					-1.84E+00	Neutral			-1.84E+00	Neutral
31	p.Leu31Thr			16130	2611	4.30	2.57					-7.23E+00	Indeterminate			-7.23E+00	Indeterminate
31	p.Leu31Arg			19399	2812	5.17	2.77					-3.31E+00	Neutral			-3.31E+00	Neutral
31	p.Leu31Ser			18159	3537	4.84	3.49					-1.24E+01	Indeterminate			-1.24E+01	Indeterminate
31	p.Leu31Ile			26461	3657	7.05	3.61					-1.36E+00	Neutral			-1.36E+00	Neutral
31	p.Leu31Met			25568	4775	6.81	4.71					-6.95E+00	Indeterminate			-6.95E+00	Indeterminate
31	p.Leu31His			14102	2056	3.76	2.03					-5.62E+00	Neutral			-5.62E+00	Neutral
31	p.Leu31Gln			20265	4695	5.40	4.63					-1.90E+01	Indeterminate			-1.90E+01	Indeterminate
31	p.Leu31Pro			18066	30967	4.81	30.53					-5.32E+01	Deleterious			-5.32E+01	Deleterious
31	p.Leu31Leu	Synonymous		29114	4009	7.76	3.95					-1.06E+00	Neutral			-1.06E+00	Neutral
31	p.Leu31Asp			17841	8877	4.75	8.75					-5.32E+01	Deleterious			-5.32E+01	Deleterious
31	p.Leu31Glu			12731	2060	3.39	2.03					-9.76E+00	Indeterminate			-9.76E+00	Indeterminate
31	p.Leu31Ala			17448	3125	4.65	3.08					-9.65E+00	Indeterminate			-9.65E+00	Indeterminate
31	p.Leu31Gly			16782	2693	4.47	2.65					-6.62E+00	Indeterminate			-6.62E+00	Indeterminate
31	p.Leu31Val			16042	3588	4.27	3.54					-2.14E+01	Indeterminate			-2.14E+01	Indeterminate
31	p.Leu31Tyr			29006	7869	7.73	7.76					-2.12E+01	Indeterminate			-2.12E+01	Indeterminate
31	p.Leu31Cys			10884	2718	2.90	2.68					-3.93E+01	Indeterminate			-3.32E+01	Indeterminate
31	p.Leu31Trp			13277	2652	3.54	2.61					-1.85E+01	Indeterminate			-1.85E+01	Indeterminate
31	p.Leu31Phe			14121	2017	3.76	1.99					-5.13E+00	Neutral			-5.13E+00	Neutral
32	p.Leu32Asn			2757	2086	5.77	6.42	1361	1177	5.80	7.89	-2.49E+01	Indeterminate	-2.98E+01	Indeterminate	-5.32E+01	Deleterious
32	p.Leu32Lys			4132	3792	8.65	11.66	1940	1823	8.26	12.23	-2.90E+01	Indeterminate	-2.73E+01	Indeterminate	-5.32E+01	Deleterious
32	p.Leu32Thr			1677	990	3.51	3.05	942	231	4.01	1.55	-1.98E+01	Indeterminate	-3.76E-02	Neutral	-1.59E+01	Indeterminate
32	p.Leu32Arg			1964	1636	4.11	5.03	944	914	4.02	6.13	-4.17E+01	Indeterminate	-5.14E+01	Indeterminate	-5.32E+01	Deleterious
32	p.Leu32Ser			2088	1649	4.37	5.07	1437	721	6.12	4.84	-3.51E+01	Indeterminate	-4.41E+00	Neutral	-3.25E+01	Indeterminate
32	p.Leu32Ile			2006	586	4.20	1.80	1122	207	4.78	1.39	-4.47E-01	Neutral	-1.01E-04	Neutral	-5.77E-02	Neutral
32	p.Leu32Met			3442	1252	7.21	3.85	1437	260	6.12	1.74	-6.29E-01	Neutral	-1.11E-05	Neutral	-1.07E-01	Neutral
32	p.Leu32His			1717	1516	3.60	4.66	795	651	3.39	4.37	-5.20E+01	Indeterminate	-4.02E+01	Indeterminate	-5.32E+01	Deleterious
32	p.Leu32Gln			2563	1371	5.37	4.22	1457	756	6.21	5.07	-9.05E+00	Indeterminate	-5.05E+00	Neutral	-1.07E+01	Indeterminate
32	p.Leu32Pro	Pathogenic		4912	4768	10.29	14.67	1841	2065	7.84	13.85	-2.89E+01	Indeterminate	-4.36E+01	Indeterminate	-5.32E+01	Deleterious
32	p.Leu32Leu	Synonymous		1981	642	4.15	1.97	851	281	3.63	1.88	-1.06E+00	Neutral	-1.06E+00	Neutral	-8.18E-01	Neutral
32	p.Leu32Asp			2011	1682	4.21	5.17	723	788	3.08	5.29	-4.13E+01	Indeterminate	-5.32E+01	Deleterious	-5.32E+01	Deleterious
32	p.Leu32Glu			2518	2363	5.27	7.27	1148	1069	4.89	7.17	-4.52E+01	Indeterminate	-4.08E+01	Indeterminate	-5.32E+01	Deleterious
32	p.Leu32Ala			876	322	1.83	0.99	256	98	1.09	0.66	-8.43E+00	Indeterminate	-1.42E+01	Indeterminate	-1.86E+01	Indeterminate
32	p.Leu32Gly			1595	1406	3.34	4.32	878	716	3.74	4.80	-5.32E+01	Deleterious	-3.68E+01	Indeterminate	-5.32E+01	Deleterious
32	p.Leu32Val			3564	1243	7.46	3.82	1582	300	6.74	2.01	-3.96E-01	Neutral	-1.56E-05	Neutral	-4.60E-02	Neutral
32	p.Leu32Tyr			1947	1414	4.08	4.35	1105	911	4.71	6.11	-3.02E+01	Indeterminate	-3.14E+01	Indeterminate	-5.32E+01	Deleterious
32	p.Leu32Cys			1419	550	2.97	1.69	793	133	3.38	0.89	-5.43E+00	Neutral	-1.53E-04	Neutral	-3.18E+00	Neutral
32	p.Leu32Trp			2952	2525	6.18	7.77	1653	1521	7.04	10.20	-3.22E+01	Indeterminate	-2.96E+01	Indeterminate	-5.32E+01	Deleterious
32	p.Leu32Phe			1622	716	3.40	2.20	1208	288	5.15	1.93	-7.76E+00	Indeterminate	-7.36E-03	Neutral	-5.09E+00	Neutral
33	p.Glu33Asn			2405	1987	6.29	6.03					-1.19E-01	Neutral			-1.19E-01	Neutral
33	p.Glu33Lys			2310	2125	6.04	6.45					-4.10E-01	Neutral			-4.10E-01	Neutral
33	p.Glu33Thr			2551	2244	6.67	6.81					-1.92E-01	Neutral			-1.92E-01	Neutral
33	p.Glu33Arg			323	265	0.84	0.80					-9.24E+00	Indeterminate			-9.24E+00	Indeterminate
33	p.Glu33Ser			894	826	2.34	2.51					-3.59E+00	Neutral			-3.59E+00	Neutral
33	p.Glu33Ile			3960	3269	10.35	9.92					-1.31E-02	Neutral			-1.31E-02	Neutral
33	p.Glu33Met			4009	3279	10.48	9.95					-1.07E-02	Neutral			-1.07E-02	Neutral
33	p.Glu33His			2694	2397	7.04	7.28					-1.79E-01	Neutral			-1.79E-01	Neutral
33	p.Glu33Gln			3450	3143	9.02	9.54					-9.23E-02	Neutral			-9.23E-02	Neutral
33	p.Glu33Pro			2489	1886	6.51	5.73					-3.51E-02	Neutral			-3.51E-02	Neutral
33	p.Glu33Leu			2006	1914	5.25	5.81					-8.22E-01	Neutral			-8.22E-01	Neutral
33	p.Glu33Asp			1895	1477	4.96	4.48					-1.51E-01	Neutral			-1.51E-01	Neutral
33	p.Glu33Glu	Synonymous		1646	1522	4.30	4.62					-1.06E+00	Neutral			-1.06E+00	Neutral
33	p.Glu33Ala			425	405	1.11	1.23					-1.14E+01	Indeterminate			-1.14E+01	Indeterminate
33	p.Glu33Gly			2167	1901	5.67	5.77					-3.21E-01	Neutral			-3.21E-01	Neutral
33	p.Glu33Val			1148	984	3.00	2.99					-1.44E+00	Neutral			-1.44E+00	Neutral
33	p.Glu33Tyr			374	322	0.98	0.98					-9.22E+00	Indeterminate			-9.22E+00	Indeterminate
33	p.Glu33Cys			784	708	2.05	2.15					-3.97E+00	Neutral			-3.97E+00	Neutral
33	p.Glu33Trp			1942	1609	5.08	4.88					-2.63E-01	Neutral			-2.63E-01	Neutral
33	p.Glu33Phe			771	678	2.02	2.06					-3.58E+00	Neutral			-3.58E+00	Neutral
34	p.Ala34Asn			1048	380	2.48	2.47	974	864	2.39	3.01	-5.06E+00	Neutral	-9.96E+00	Indeterminate	-1.15E+01	Indeterminate
34	p.Ala34Lys			1978	717	4.68	4.66	1977	1399	4.84	4.88	-1.63E+00	Neutral	-9.22E-01	Neutral	-1.08E+00	Neutral
34	p.Ala34Thr			1206	360	2.86	2.34	1040	862	2.55	3.01	-1.34E+00	Neutral	-7.07E+00	Indeterminate	-5.63E+00	Neutral
34	p.Ala34Arg			4462	1497	10.56	9.73	4243	2972	10.39	10.37	-6.89E-02	Neutral	-7.03E-02	Neutral	-6.31E-03	Neutral
34	p.Ala34Ser			1003	246	2.37	1.60	903	642	2.21	2.24	-4.70E-01	Neutral	-4.44E+00	Neutral	-2.77E+00	Neutral
34	p.Ala34Ile			1160	348	2.75	2.26	1259	760	3.08	2.65	-1.50E+00	Neutral	-8.61E-01	Neutral	-9.65E-01	Neutral
34	p.Ala34Met			2790	1090	6.61	7.08	2925	1910	7.16	6.66	-1.25E+00	Neutral	-1.32E-01	Neutral	-4.14E-01	Neutral
34	p.Ala34His			1987	510	4.70	3.31	1486	902	3.64	3.15	-6.96E-02	Neutral	-5.80E-01	Neutral	-1.13E-01	Neutral
34	p.Ala34Gln			2756	1108	6.53	7.20	2539	2187	6.22	7.63	-1.56E+00	Neutral	-1.98E+00	Neutral	-1.75E+00	Neutral
34	p.Ala34Pro			1736	907	4.11	5.89	1555	1319	3.81	4.60	-1.11E+01	Indeterminate	-4.36E+00	Neutral	-1.19E+01	Indeterminate
34	p.Ala34Leu			2439	838	5.77	5.45	2468	1808	6.05	6.31	-6.70E-01	Neutral	-6.81E-01	Neutral	-3.98E-01	Neutral
34	p.Ala34Asp			2130	868	5.04	5.64	2207	1477	5.41	5.15	-2.81E+00	Neutral	-4.33E-01	Neutral	-1.54E+00	Neutral
34	p.Ala34Glu			2272	972	5.38	6.32	2212	1619	5.42	5.65	-3.25E+00	Neutral	-8.98E-01	Neutral	-2.19E+00	Neutral
34	p.Ala34Ala	Synonymous		2329	837	5.51	5.44	2131	1576	5.22	5.50	-1.06E+00	Neutral	-1.06E+00	Neutral	-8.18E-01	Neutral
34	p.Ala34Gly			1829	780	4.33	5.07	1820	1092	4.46	3.81	-4.56E+00	Neutral	-2.84E-01	Neutral	-2.72E+00	Neutral
34	p.Ala34Val			1093	525	2.59	3.41	1289	878	3.16	3.06	-1.40E+01	Indeterminate	-1.87E+00	Neutral	-1.23E+01	Indeterminate
34	p.Ala34Tyr			2781	751	6.58	4.88	2256	1675	5.53	5.84	-3.14E-02	Neutral	-9.51E-01	Neutral	-2.33E-01	Neutral
34	p.Ala34Cys			2203	772	5.22	5.02	2252	1303	5.52	4.55	-1.01E+00	Neutral	-8.89E-02	Neutral	-2.82E-01	Neutral
34	p.Ala34Trp			1513	537	3.58	3.49	1712	1111	4.19	3.88	-2.45E+00	Neutral	-6.83E-01	Neutral	-1.47E+00	Neutral
34	p.Ala34Phe			3520	1345	8.33	8.74	3578	2302	8.76	8.03	-5.90E-01	Neutral	-4.90E-02	Neutral	-1.10E-01	Neutral
35	p.Gly35Asn			1949	799	3.50	1.78	10005	6273	4.43	2.61	-8.73E+00	Indeterminate	-1.65E+00	Neutral	-7.34E+00	Indeterminate
35	p.Gly35Lys			2552	1260	4.58	2.80	10948	8539	4.85	3.55	-1.23E+01	Indeterminate	-4.60E+00	Neutral	-1.32E+01	Indeterminate
35	p.Gly35Thr			2584	1647	4.63	3.66	10143	10610	4.49	4.41	-2.60E+01	Indeterminate	-1.54E+01	Indeterminate	-5.32E+01	Deleterious
35	p.Gly35Arg		Neutral	2733	924	4.90	2.06	11916	8419	5.28	3.50	-2.06E+00	Neutral	-2.39E+00	Neutral	-2.42E+00	Neutral
35	p.Gly35Ser			2126	992	3.81	2.21	8773	6513	3.89	2.71	-1.25E+01	Indeterminate	-5.14E+00	Neutral	-1.39E+01	Indeterminate
35	p.Gly35Ile			2090	4458	3.75	9.92	9346	19604	4.14	8.15	-5.32E+01	Deleterious	-5.32E+01	Deleterious	-5.32E+01	Deleterious
35	p.Gly35Met			3439	1998	6.17	4.44	12679	12683	5.62	5.27	-1.51E+01	Indeterminate	-1.04E+01	Indeterminate	-2.13E+01	Indeterminate
35	p.Gly35His			2988	1047	5.36	2.33	11525	8225	5.10	3.42	-2.14E+00	Neutral	-2.69E+00	Neutral	-2.71E+00	Neutral

35	p.Gly35Gln			4241	1991	7.61	4.43	14962	14359	6.63	5.97	-5.28E+00	Neutral	-7.21E+00	Indeterminate	-9.22E+00	Indeterminate
35	p.Gly35Pro			2624	9100	4.71	20.24	10587	31189	4.69	12.96	-5.32E+01	Deleterious	-5.32E+01	Deleterious	-5.32E+01	Deleterious
35	p.Gly35Leu			3145	2964	5.64	6.59	12395	15692	5.49	6.52	-5.32E+01	Deleterious	-2.23E+01	Indeterminate	-5.32E+01	Deleterious
35	p.Gly35Asp			2568	1419	4.61	3.16	10551	7765	4.67	3.23	-1.75E+01	Indeterminate	-3.69E+00	Neutral	-1.72E+01	Indeterminate
35	p.Gly35Glu		Neutral	2733	1146	4.90	2.55	10753	9407	4.76	3.91	-6.09E+00	Indeterminate	-7.72E+00	Indeterminate	-1.04E+01	Indeterminate
35	p.Gly35Ala			2285	903	4.10	2.01	9298	6841	4.12	2.84	-6.10E+00	Indeterminate	-4.52E+00	Neutral	-7.55E+00	Indeterminate
35	p.Gly35Gly	Synonymous		1358	329	2.44	0.73	5570	2694	2.47	1.12	-1.06E+00	Neutral	-1.06E+00	Neutral	-8.18E-01	Neutral
35	p.Gly35Val		Deleterious	3854	5902	6.91	13.13	15424	25699	6.83	10.68	-5.32E+01	Deleterious	-3.73E+01	Indeterminate	-5.32E+01	Deleterious
35	p.Gly35Tyr			2312	1427	4.15	3.17	10542	8067	4.67	3.35	-2.63E+01	Indeterminate	-4.47E+00	Neutral	-2.63E+01	Indeterminate
35	p.Gly35Cys			3042	1561	5.46	3.47	11585	9259	5.13	3.85	-1.15E+01	Indeterminate	-4.73E+00	Neutral	-1.26E+01	Indeterminate
35	p.Gly35Trp		Deleterious	4083	3648	7.32	8.11	15410	18194	6.83	7.56	-4.10E+01	Indeterminate	-1.46E+01	Indeterminate	-5.32E+01	Deleterious
35	p.Gly35Phe			3051	1446	5.47	3.22	13371	10574	5.92	4.39	-8.60E+00	Indeterminate	-3.57E+00	Neutral	-8.93E+00	Indeterminate
36	p.Ala36Asn			5581	3345	5.14	6.08					-2.13E+01	Indeterminate			-2.13E+01	Indeterminate
36	p.Ala36Lys			8028	18657	7.40	33.90					-5.32E+01	Deleterious			-5.32E+01	Deleterious
36	p.Ala36Thr			2783	1191	2.56	2.16					-1.56E+01	Indeterminate			-1.56E+01	Indeterminate
36	p.Ala36Arg			4397	1199	4.05	2.18					-8.65E-01	Neutral			-8.65E-01	Neutral
36	p.Ala36Ser			4348	1450	4.01	2.64					-3.16E+00	Neutral			-3.16E+00	Neutral
36	p.Ala36Ile			5237	1240	4.83	2.25					-1.36E-01	Neutral			-1.36E-01	Neutral
36	p.Ala36Met			6481	232	5.97	0.42					0.00E+00	Neutral			0.00E+00	Neutral
36	p.Ala36His			5535	1160	5.10	2.11					-2.28E-02	Neutral			-2.28E-02	Neutral
36	p.Ala36Gln			5409	1222	4.98	2.22					-6.88E-02	Neutral			-6.88E-02	Neutral
36	p.Ala36Pro			8410	9367	7.75	17.02					-5.32E+01	Deleterious			-5.32E+01	Deleterious
36	p.Ala36Leu			6369	2754	5.87	5.00					-5.94E+00	Indeterminate			-5.94E+00	Indeterminate
36	p.Ala36Asp			3629	1165	3.34	2.12					-3.54E+00	Neutral			-3.54E+00	Neutral
36	p.Ala36Glu			7209	3345	6.64	6.08					-6.72E+00	Indeterminate			-6.72E+00	Indeterminate
36	p.Ala36Ala	Synonymous		4309	1200	3.97	2.18					-1.06E+00	Neutral			-1.06E+00	Neutral
36	p.Ala36Gly			2425	903	2.23	1.64					-1.15E+01	Indeterminate			-1.15E+01	Indeterminate
36	p.Ala36Val			5770	1324	5.32	2.41					-6.40E-02	Neutral			-6.40E-02	Neutral
36	p.Ala36Tyr			4470	1637	4.12	2.97					-4.78E+00	Neutral			-4.78E+00	Neutral
36	p.Ala36Cys			4719	296	4.35	0.54					-1.04E-14	Neutral			0.00E+00	Neutral
36	p.Ala36Trp			7861	1433	7.24	2.60					-2.83E-04	Neutral			-2.83E-04	Neutral
36	p.Ala36Phe			5551	1908	5.12	3.47					-2.39E+00	Neutral			-2.39E+00	Neutral
37	p.Leu37Asn			15659	807	7.21	4.34					-7.48E-02	Neutral			-7.48E-02	Neutral
37	p.Leu37Lys			9469	805	4.36	4.33					-8.20E+00	Indeterminate			-8.20E+00	Indeterminate
37	p.Leu37Thr			7000	646	3.22	3.48					-1.54E+01	Indeterminate			-1.54E+01	Indeterminate
37	p.Leu37Arg			10261	287	4.73	1.54					-2.02E-05	Neutral			-2.02E-05	Neutral
37	p.Leu37Ser			7945	512	3.66	2.76					-3.05E+00	Neutral			-3.05E+00	Neutral
37	p.Leu37Ile			13094	1494	6.03	8.04					-1.56E+01	Indeterminate			-1.56E+01	Indeterminate
37	p.Leu37Met			11651	487	5.37	2.62					-1.66E-02	Neutral			-1.66E-02	Neutral
37	p.Leu37His			11818	1084	5.44	5.83					-8.27E+00	Indeterminate			-8.27E+00	Indeterminate
37	p.Leu37Gln			17769	1937	8.18	10.42					-9.51E+00	Indeterminate			-9.51E+00	Indeterminate
37	p.Leu37Pro			9322	1163	4.29	6.26					-2.77E+01	Indeterminate			-2.77E+01	Indeterminate
37	p.Leu37Leu	Synonymous		14149	931	6.52	5.01					-1.06E+00	Neutral			-1.06E+00	Neutral
37	p.Leu37Asp			10884	742	5.01	3.99					-2.34E+00	Neutral			-2.34E+00	Neutral
37	p.Leu37Glu			9551	634	4.40	3.41					-2.57E+00	Neutral			-2.57E+00	Neutral
37	p.Leu37Ala			11018	534	5.08	2.87					-1.43E-01	Neutral			-1.43E-01	Neutral
37	p.Leu37Gly			9306	588	4.29	3.16					-2.03E+00	Neutral			-2.03E+00	Neutral
37	p.Leu37Val			13136	1307	6.05	7.03					-9.83E+00	Indeterminate			-9.83E+00	Indeterminate
37	p.Leu37Tyr			8482	697	3.91	3.75					-8.28E+00	Indeterminate			-8.28E+00	Indeterminate
37	p.Leu37Cys			9322	518	4.29	2.79					-8.47E-01	Neutral			-8.47E-01	Neutral
37	p.Leu37Trp			6345	2856	2.92	15.37					-5.32E+01	Deleterious			-5.32E+01	Deleterious
37	p.Leu37Phe			10921	555	5.03	2.99					-2.43E-01	Neutral			-2.43E-01	Neutral
38	p.Pro38Asn			7663	2366	4.62	2.91					-5.32E+01	Deleterious			-5.32E+01	Deleterious
38	p.Pro38Lys			6677	2439	4.03	3.00					-5.32E+01	Deleterious			-5.32E+01	Deleterious
38	p.Pro38Thr			6647	881	4.01	1.08					-6.12E+00	Indeterminate			-6.12E+00	Indeterminate
38	p.Pro38Arg			6252	3126	3.77	3.84					-5.32E+01	Deleterious			-5.32E+01	Deleterious
38	p.Pro38Ser			6013	1033	3.63	1.27					-1.73E+01	Indeterminate			-1.73E+01	Indeterminate
38	p.Pro38Ile			6138	1077	3.70	1.32					-1.81E+01	Indeterminate			-1.81E+01	Indeterminate
38	p.Pro38Met			8518	1945	5.14	2.39					-2.77E+01	Indeterminate			-2.77E+01	Indeterminate
38	p.Pro38His			5342	2012	3.22	2.47					-5.32E+01	Deleterious			-5.32E+01	Deleterious
38	p.Pro38Gln			17977	7277	10.84	8.94					-5.32E+01	Deleterious			-5.32E+01	Deleterious
38	p.Pro38Pro	Synonymous		7226	717	4.36	0.88					-1.06E+00	Neutral			-1.06E+00	Neutral
38	p.Pro38Leu			10269	3279	6.19	4.03					-5.30E+01	Indeterminate			-3.32E+01	Indeterminate
38	p.Pro38Asp			7287	14782	4.39	18.15					-5.32E+01	Deleterious			-5.32E+01	Deleterious
38	p.Pro38Glu			14248	3748	8.59	4.60					-2.57E+01	Indeterminate			-2.57E+01	Indeterminate
38	p.Pro38Ala			6236	3456	3.76	4.24					-5.32E+01	Deleterious			-5.32E+01	Deleterious
38	p.Pro38Gly			8191	804	4.94	0.99					-7.14E-01	Neutral			-7.14E-01	Neutral
38	p.Pro38Val			10026	1016	6.04	1.25					-5.34E-01	Neutral			-5.34E-01	Neutral
38	p.Pro38Tyr			4282	1445	2.58	1.77					-5.32E+01	Deleterious			-5.32E+01	Deleterious
38	p.Pro38Cys			8165	611	4.92	0.75					-4.22E-02	Neutral			-4.22E-02	Neutral
38	p.Pro38Trp			7975	24617	4.81	30.23					-5.32E+01	Deleterious			-5.32E+01	Deleterious
38	p.Pro38Phe			10730	4800	6.47	5.89					-5.32E+01	Deleterious			-5.32E+01	Deleterious
39	p.Asn39Asn	Synonymous		11025	1321	4.88	1.95					-1.06E+00	Neutral			-1.06E+00	Neutral
39	p.Asn39Lys			12759	3256	5.65	4.81					-2.29E+01	Indeterminate			-2.29E+01	Indeterminate
39	p.Asn39Thr			9531	725	4.22	1.07					-1.29E-02	Neutral			-1.29E-02	Neutral
39	p.Asn39Arg			7919	1061	3.51	1.57					-3.95E+00	Neutral			-3.95E+00	Neutral
39	p.Asn39Ser			8858	1238	3.92	1.83					-4.05E+00	Neutral			-4.05E+00	Neutral
39	p.Asn39Ile			10639	1845	4.71	2.73					-7.95E+00	Indeterminate			-7.95E+00	Indeterminate
39	p.Asn39Met			12667	3339	5.61	4.93					-2.52E+01	Indeterminate			-2.52E+01	Indeterminate
39	p.Asn39His			11236	950	4.98	1.40					-2.72E-02	Neutral			-2.72E-02	Neutral
39	p.Asn39Gln			8347	932	3.70	1.38					-1.24E+00	Neutral			-1.24E+00	Neutral
39	p.Asn39Pro			9695	16800	4.30	24.82					-5.32E+01	Deleterious			-5.32E+01	Deleterious
39	p.Asn39Leu			13886	6932	6.15	10.24					-5.32E+01	Deleterious			-5.32E+01	Deleterious
39	p.Asn39Asp			15016	1873	6.65	2.77					-6.59E-01	Neutral			-6.59E-01	Neutral
39	p.Asn39Glu			15471	2195	6.85	3.24					-1.56E+00	Neutral			-1.56E+00	Neutral
39	p.Asn39Ala			8785	1365	3.89	2.02					-6.60E+00	Indeterminate			-6.60E+00	Indeterminate
39	p.Asn39Gly			15618	1831	6.92	2.70					-3.42E-01	Neutral			-3.42E-01	Neutral
39	p.Asn39Val			12208	1947	5.41	2.88					-4.58E+00	Neutral			-4.58E+00	Neutral
39	p.Asn39Tyr			8959	952	3.97	1.41					-7.21E-01	Neutral			-7.21E-01	Neutral
39	p.Asn39Cys			13589	7404	6.02	10.94					-5.32E+01	Deleterious			-5.32E+01	Deleterious
39	p.Asn39Trp			9837	1931	4.36	2.85					-1.36E+01	Indeterminate			-1.36E+01	Indeterminate
39	p.Asn39Phe			9654	9801	4.28	14.48					-5.32E+01	Deleterious			-5.32E+01	Deleterious
40	p.Ala40Asn			17799	1463	5.87	4.98					-6.02E+00	Indeterminate			-6.02E+00	Indeterminate
40	p.Ala40Lys			21626	1443	7.13	4.91					-1.40E+00	Neutral			-1.40E+00	Neutral
40	p.Ala40Thr			19138	8921	6.31	30.36					-5.32E+01	Deleterious			-5.32E+01	Deleterious
40	p.Ala40Arg			12717	763	4.19	2.60					-2.18E+00	Neutral			-2.18E+00	Neutral
40	p.Ala40Ser			7528	329	2.48	1.12					-8.34E-01	Neutral			-8.34E-01	Neutral
40	p.Ala40Ile			9646	648	3.18	2.21					-6.06E+00	Indeterminate			-6.06E+00	Indeterminate
40	p.Ala40Met			12826	464	4.23	1.58					-1.44E-02	Neutral			-1.44E-02	Neutral
40	p.Ala40His			10373	460	3.42	1.57					-3.86E-01	Neutral			-3.86E-01	Neutral
40	p.Ala40Gln			12433	454	4.10	1.54					-1.93E-02	Neutral			-1.93E-02	Neutral
40	p.Ala40Pro			13362	1203	4.41	4.09					-1.22E+01	Indeterminate			-1.22E+01	Indeterminate
40	p.Ala40Leu			14549	3440	4.80	11.71					-5.32E+01	Deleterious			-5.32E+01	Deleterious
40	p.Ala40Asp			12913	1289	4.26	4.39					-1.76E+01	Indeterminate			-1.76E+01	Indeterminate
40	p.Ala40Glu			22310	1055	7.36	3.59					-4.71E-02	Neutral			-4.71E-02	Neutral
40	p.Ala40Ala	Synonymous		17869	1075	5.89	3.66					-1.06E+00	Neutral			-1.06E+00	Neutral
40	p.Ala40Gly			20764	1292	6.85	4.40					-9.43E-01	Neutral			-9.43E-01	Neutral
40	p.Ala40Val			17468	1452	5.76	4.94					-6.48E+00	Indeterminate			-6.48E+00	Indeterminate
40	p.Ala40Tyr			14320	633	4.72	2.15					-1.25E-01	Neutral			-1.25E-01	Neutral
40	p.Ala40Cys			15434	936	5.09	3.19					-1.56E+00	Neutral			-1.56E+00	Neutral
40	p.Ala40Trp			12309	863	4.06	2.94					-5.12E+00	Neutral			-5.12E+00	Neutral
40	p.Ala40Phe			17875	1204	5.89	4.10					-2.21E+00	Neutral			-2.21E+00	Neutral
41	p.Pro41Asn			3018	760	4.29	3.16					-3.89E-01	Neutral			-3.89E-01	Neutral
41	p.Pro41Lys			2802	980	3.98	4.07					-4.11E+00	Neutral			-4.11E+00	Neutral
41	p.Pro41Thr			2775	782	3.95	3.25					-1.20E+00	Neutral			-1.20E+00	Neutral
41	p.Pro41Arg			2832	1777	4.03	7.39					-3.01E+01	Indeterminate			-2.99E+01	Indeterminate
41	p.Pro41Ser			4466	1181	6.35	4.91					-1.74E-01	Neutral			-1.74E-01	Neutral
41	p.Pro41Ile			2598	1141	3.69	4.74					-1.15E+01	Indeterminate			-1.15E+01	Indeterminate
41	p.Pro41Met			4122	874	5.86	3.63					-1.44E-02	Neutral			-1.44E-02	Neutral
41	p.Pro41His			2358	1155	3.35	4.80					-1.81E+01	Indeterminate			-1.81E+01	Indeterminate
41	p.Pro41Gln		Neutral	3840	1354	5.46	5.63					-2.48E+00	Neutral			-2.48E+00	Neutral
41	p.Pro41Pro	Synonymous		2669	730	3.79	3.03					-1.06E+00	Neutral			-1.06E+00	Neutral
41	p.Pro41Leu			3638	786	5.17	3.27					-3.41E-02	Neutral			-3.41E-02	Neutral
41	p.Pro41Asp			3619	1263	5.15	5.25					-2.62E+00	Neutral			-2.62E+00	Neutral
41	p.Pro41Glu			3875	956	5.51	3.97					-1.36E-01	Neutral			-1.36E-01	Neutral
41	p.Pro41Ala			4397	1208	6.25	5.02					-2.71E-01	Neutral			-2.71E-01	Neutral
41	p.Pro41Gly			2512	730	3.57	3.03					-1.82E+00	Neutral			-1.82E+00	Neutral
41	p.Pro41Val			5452	1628	7.75	6.77					-3.05E-01	Neutral			-3.05E-01	Neutral
41	p.Pro41Tyr			4042	2317	5.75	9.63					-1.69E+01	Indeterminate			-1.69E+01	Indeterminate
41	p.Pro41Cys			3683	1331	5.24	5.53					-3.07E+00	Neutral			-3.07E+00	Neutral
41	p.Pro41Trp			2884	1129	4.10	4.69					-6.53E+00	Indeterminate			-6.53E+00	Indeterminate
41	p.Pro41Phe			4753	1980	6.76	8.23					-4.10E+00	Neutral			-4.10E+00	Neutral
42	p.Asn42Asn	Synonymous		8645	341	4.30	0.55					-1.06E+00	Neutral			-1.06E+00	Neutral
42	p.Asn42Lys			9397	4647	4.67	7.54					-5.32E+01	Deleterious			-5.32E+01	Deleterious
42	p.Asn42Thr			10798	564	5.37	0.92					-3.68E+00	Neutral			-3.68E+00	Neutral
42	p.Asn42Arg			13173	6054	6.55	9.82					-5.32E+01	Deleterious			-5.32E+01	Deleterious
42	p.Asn42Ser			9213	444	4.58	0.72					-3.19E+00	Neutral			-3.19E+00	Neutral
42	p.Asn42Ile			10911	4981	5.42	8.08					-5.32E+01	Deleterious			-5.32E+01	Deleterious
42	p.Asn42Met			8586	3308	4.27	5.37					-5.32E+01	Deleterious			-5.32E+01	Deleterious

42	p.Asn42His		9638	2787	4.79	4.52					-5.32E+01	Deleterious			-5.32E+01	Deleterious
42	p.Asn42Gln		10388	2910	5.16	4.72					-5.32E+01	Deleterious			-5.32E+01	Deleterious
42	p.Asn42Pro		6267	2676	3.11	4.34					-5.32E+01	Deleterious			-5.32E+01	Deleterious
42	p.Asn42Leu		9292	4188	4.62	6.79					-5.32E+01	Deleterious			-5.32E+01	Deleterious
42	p.Asn42Asp		9064	576	4.50	0.93					-1.07E+01	Indeterminate			-1.07E+01	Indeterminate
42	p.Asn42Glu		12901	6353	6.41	10.31					-5.32E+01	Deleterious			-5.32E+01	Deleterious
42	p.Asn42Ala		7254	328	3.61	0.53					-3.46E+00	Neutral			-3.46E+00	Neutral
42	p.Asn42Gly		12142	451	6.03	0.73					-2.42E-01	Neutral			-2.42E-01	Neutral
42	p.Asn42Val		13261	5223	6.59	8.47					-5.32E+01	Deleterious			-5.32E+01	Deleterious
42	p.Asn42Tyr		9832	4604	4.89	7.47					-5.32E+01	Deleterious			-5.32E+01	Deleterious
42	p.Asn42Cys		10625	622	5.28	1.01					-6.36E+00	Indeterminate			-6.36E+00	Indeterminate
42	p.Asn42Trp		8933	5163	4.44	8.38					-5.32E+01	Deleterious			-5.32E+01	Deleterious
42	p.Asn42Phe		10895	5419	5.41	8.79					-5.32E+01	Deleterious			-5.32E+01	Deleterious
43	p.Ser43Asn		8865	6639	3.69	3.91					-3.02E+00	Neutral			-3.02E+00	Neutral
43	p.Ser43Lys		16747	11835	6.97	6.97					-4.78E-01	Neutral			-4.78E-01	Neutral
43	p.Ser43Thr		14488	9553	6.03	5.62					-3.96E-01	Neutral			-3.96E-01	Neutral
43	p.Ser43Arg		16871	11411	7.02	6.72					-3.13E-01	Neutral			-3.13E-01	Neutral
43	p.Ser43Ser	Synonymous	11014	7488	4.58	4.41					-1.06E+00	Neutral			-1.06E+00	Neutral
43	p.Ser43Ile		7943	6763	3.30	3.98					-6.55E+00	Indeterminate			-6.55E+00	Indeterminate
43	p.Ser43Met		15797	11730	6.57	6.90					-8.40E-01	Neutral			-8.40E-01	Neutral
43	p.Ser43His		16521	11177	6.87	6.58					-3.35E-01	Neutral			-3.35E-01	Neutral
43	p.Ser43Gln		17314	11767	7.20	6.93					-3.01E-01	Neutral			-3.01E-01	Neutral
43	p.Ser43Pro		12527	8469	5.21	4.98					-7.37E-01	Neutral			-7.37E-01	Neutral
43	p.Ser43Leu		10449	7515	4.35	4.42					-1.74E+00	Neutral			-1.74E+00	Neutral
43	p.Ser43Asp		11537	8545	4.80	5.03					-1.70E+00	Neutral			-1.70E+00	Neutral
43	p.Ser43Glu		14597	10128	6.07	5.96					-6.03E-01	Neutral			-6.03E-01	Neutral
43	p.Ser43Ala		11830	7793	4.92	4.59					-6.98E-01	Neutral			-6.98E-01	Neutral
43	p.Ser43Gly		4691	3009	1.95	1.77					-4.02E+00	Neutral			-4.02E+00	Neutral
43	p.Ser43Val		11779	8766	4.90	5.16					-1.68E+00	Neutral			-1.68E+00	Neutral
43	p.Ser43Tyr		9565	6955	3.98	4.09					-2.22E+00	Neutral			-2.22E+00	Neutral
43	p.Ser43Cys		5091	3662	2.12	2.16					-6.00E+00	Indeterminate			-6.00E+00	Indeterminate
43	p.Ser43Trp		10965	8262	4.56	4.86					-2.10E+00	Neutral			-2.10E+00	Neutral
43	p.Ser43Phe		11776	8442	4.90	4.97					-1.32E+00	Neutral			-1.32E+00	Neutral
44	p.Tyr44Asn		7443	4844	6.01	6.02					-3.69E+00	Neutral			-3.69E+00	Neutral
44	p.Tyr44Lys		4300	2790	3.47	3.47					-8.10E+00	Indeterminate			-8.10E+00	Indeterminate
44	p.Tyr44Thr		7466	3963	6.03	4.92					-1.08E+00	Neutral			-1.08E+00	Neutral
44	p.Tyr44Arg		4924	2723	3.98	3.38					-3.23E+00	Neutral			-3.23E+00	Neutral
44	p.Tyr44Ser		6073	3554	4.90	4.42					-3.02E+00	Neutral			-3.02E+00	Neutral
44	p.Tyr44Ile		5380	3030	4.34	3.76					-3.05E+00	Neutral			-3.05E+00	Neutral
44	p.Tyr44Met		5878	3444	4.75	4.28					-3.22E+00	Neutral			-3.22E+00	Neutral
44	p.Tyr44His		8889	5272	7.18	6.55					-1.55E+00	Neutral			-1.55E+00	Neutral
44	p.Tyr44Gln		2488	1711	2.01	2.13					-1.82E+01	Indeterminate			-1.82E+01	Indeterminate
44	p.Tyr44Pro		6918	8564	5.59	10.64					-3.52E+01	Indeterminate			-3.29E+01	Indeterminate
44	p.Tyr44Leu		6515	3822	5.26	4.75					-2.69E+00	Neutral			-2.69E+00	Neutral
44	p.Tyr44Asp		5508	3671	4.45	4.56					-6.48E+00	Indeterminate			-6.48E+00	Indeterminate
44	p.Tyr44Glu		4288	3217	3.46	4.00					-1.36E+01	Indeterminate			-1.36E+01	Indeterminate
44	p.Tyr44Ala		4861	4765	3.92	5.92					-2.63E+01	Indeterminate			-2.63E+01	Indeterminate
44	p.Tyr44Gly		4818	3568	3.89	4.43					-1.14E+01	Indeterminate			-1.14E+01	Indeterminate
44	p.Tyr44Val		11028	6477	8.90	8.05					-8.77E-01	Neutral			-8.77E-01	Neutral
44	p.Tyr44Tyr	Synonymous	7815	4201	6.31	5.22					-1.06E+00	Neutral			-1.06E+00	Neutral
44	p.Tyr44Cys		5174	2688	4.18	3.34					-2.08E+00	Neutral			-2.08E+00	Neutral
44	p.Tyr44Trp		7945	4712	6.41	5.85					-1.96E+00	Neutral			-1.96E+00	Neutral
44	p.Tyr44Phe		6154	3465	4.97	4.31					-2.38E+00	Neutral			-2.38E+00	Neutral
45	p.Gly45Asn		9066	190	4.25	1.00	4002	2167	4.05	3.80	-3.62E-02	Neutral	-3.51E+00	Neutral	-1.75E+00	Neutral
45	p.Gly45Lys		10852	426	5.08	2.25	4963	2650	5.02	4.65	-4.19E+00	Neutral	-2.20E+00	Neutral	-3.95E+00	Neutral
45	p.Gly45Thr		8428	159	3.95	0.84	3637	1917	3.68	3.36	-1.12E-02	Neutral	-3.59E+00	Neutral	-1.80E+00	Neutral
45	p.Gly45Arg		13226	375	6.19	1.98	5999	3130	6.07	5.49	-2.82E-01	Neutral	-1.27E+00	Neutral	-5.00E-01	Neutral
45	p.Gly45Ser		13865	787	6.49	4.15	6599	3360	6.68	5.89	-1.31E+01	Indeterminate	-8.49E-01	Neutral	-1.05E+01	Indeterminate
45	p.Gly45Ile		11823	669	5.54	3.53	6125	3452	6.20	6.05	-1.54E+01	Indeterminate	-2.00E+00	Neutral	-1.37E+01	Indeterminate
45	p.Gly45Met		11011	402	5.16	2.12	5143	2690	5.21	4.72	-2.82E+00	Neutral	-1.81E+00	Neutral	-2.56E+00	Neutral
45	p.Gly45His		9932	322	4.65	1.70	4752	2496	4.81	4.38	-1.72E+00	Neutral	-2.17E+00	Neutral	-2.01E+00	Neutral
45	p.Gly45Gln		10648	224	4.99	1.18	5023	2411	5.08	4.23	-1.80E-02	Neutral	-1.07E+00	Neutral	-2.78E-01	Neutral
45	p.Gly45Pro		8515	12301	3.99	64.90	3482	7178	3.52	12.59	-5.32E+01	Deleterious	-5.32E+01	Deleterious	-5.32E+01	Deleterious
45	p.Gly45Leu		13995	472	6.55	2.49	6319	3279	6.40	5.75	-1.04E+00	Neutral	-1.09E+00	Neutral	-8.21E-01	Neutral
45	p.Gly45Asp		11282	237	5.28	1.25	5537	2729	5.60	4.79	-1.32E-02	Neutral	-1.03E+00	Neutral	-2.57E-01	Neutral
45	p.Gly45Glu		10953	350	5.13	1.85	5234	2644	5.30	4.64	-1.26E+00	Neutral	-1.39E+00	Neutral	-1.15E+00	Neutral
45	p.Gly45Ala		8210	153	3.84	0.81	3412	1811	3.45	3.18	-1.06E-02	Neutral	-4.13E+00	Neutral	-2.19E+00	Neutral
45	p.Gly45Gly	Synonymous	9184	270	4.30	1.42	4688	2196	4.74	3.85	-1.06E+00	Neutral	-1.06E+00	Neutral	-8.18E-01	Neutral
45	p.Gly45Val		8564	481	4.01	2.54	4028	2387	4.08	4.19	-2.07E+01	Indeterminate	-5.33E+00	Neutral	-2.18E+01	Indeterminate
45	p.Gly45Tyr		8720	186	4.08	0.98	3827	2257	3.87	3.96	-5.42E-02	Neutral	-5.62E+00	Neutral	-3.37E+00	Neutral
45	p.Gly45Cys		11799	295	5.53	1.56	5280	2701	5.34	4.74	-1.11E-01	Neutral	-1.48E+00	Neutral	-5.20E-01	Neutral
45	p.Gly45Trp		9020	275	4.22	1.45	3974	2313	4.02	4.06	-1.42E+00	Neutral	-5.01E+00	Neutral	-3.99E+00	Neutral
45	p.Gly45Phe		14446	381	6.77	2.01	6777	3246	6.86	5.69	-9.41E-02	Neutral	-4.79E-01	Neutral	-9.06E-02	Neutral
46	p.Arg46Asn		12380	531	5.08	1.57					-7.10E-01	Neutral			-7.10E-01	Neutral
46	p.Arg46Lys		9730	361	3.99	1.07					-4.08E-01	Neutral			-4.08E-01	Neutral
46	p.Arg46Thr		16955	2071	6.96	6.12					-3.64E+01	Indeterminate			-3.31E+01	Indeterminate
46	p.Arg46Arg	Synonymous	13782	646	5.65	1.91					-1.06E+00	Neutral			-1.06E+00	Neutral
46	p.Arg46Ser		13112	523	5.38	1.54					-3.19E-01	Neutral			-3.19E-01	Neutral
46	p.Arg46Ile		17693	2934	7.26	8.67					-5.32E+01	Deleterious			-5.32E+01	Deleterious
46	p.Arg46Met		7746	476	3.18	1.41					-1.07E+01	Indeterminate			-1.07E+01	Indeterminate
46	p.Arg46His		8963	333	3.68	0.98					-5.25E-01	Neutral			-5.25E-01	Neutral
46	p.Arg46Gln		9746	704	4.00	2.08					-1.43E+01	Indeterminate			-1.43E+01	Indeterminate
46	p.Arg46Pro		18485	15419	7.58	45.54					-5.32E+01	Deleterious			-5.32E+01	Deleterious
46	p.Arg46Leu		16241	1098	6.66	3.24					-5.97E+00	Indeterminate			-5.97E+00	Indeterminate
46	p.Arg46Asp		8698	2412	3.57	7.12					-5.32E+01	Deleterious			-5.32E+01	Deleterious
46	p.Arg46Glu		7379	1044	3.03	3.08					-5.32E+01	Deleterious			-5.32E+01	Deleterious
46	p.Arg46Ala		9496	620	3.90	1.83					-1.04E+01	Indeterminate			-1.04E+01	Indeterminate
46	p.Arg46Gly		12625	802	5.18	2.37					-6.54E+00	Indeterminate			-6.54E+00	Indeterminate
46	p.Arg46Val		8953	791	3.67	2.34					-2.76E+01	Indeterminate			-2.76E+01	Indeterminate
46	p.Arg46Tyr		14312	1176	5.87	3.47					-1.43E+01	Indeterminate			-1.43E+01	Indeterminate
46	p.Arg46Cys		14267	907	5.85	2.68					-5.51E+00	Neutral			-5.51E+00	Neutral
46	p.Arg46Trp		11216	426	4.60	1.26					-3.27E-01	Neutral			-3.27E-01	Neutral
46	p.Arg46Phe		11963	581	4.91	1.72					-1.84E+00	Neutral			-1.84E+00	Neutral
47	p.Arg47Asn		4828	1429	3.10	2.33					-2.20E+00	Neutral			-2.20E+00	Neutral
47	p.Arg47Lys		6158	1982	3.96	3.24					-2.24E+00	Neutral			-2.24E+00	Neutral
47	p.Arg47Thr		7976	2557	5.12	4.18					-1.27E+00	Neutral			-1.27E+00	Neutral
47	p.Arg47Arg	Synonymous	8947	2906	5.75	4.75					-1.06E+00	Neutral			-1.06E+00	Neutral
47	p.Arg47Ser		6416	2760	4.12	4.51					-8.25E+00	Indeterminate			-8.25E+00	Indeterminate
47	p.Arg47Ile		6040	2038	3.88	3.33					-3.02E+00	Neutral			-3.02E+00	Neutral
47	p.Arg47Met		8024	2754	5.15	4.50					-1.94E+00	Neutral			-1.94E+00	Neutral
47	p.Arg47His		7289	2516	4.68	4.11					-2.42E+00	Neutral			-2.42E+00	Neutral
47	p.Arg47Gln		7598	3704	4.88	6.05					-1.06E+01	Indeterminate			-1.06E+01	Indeterminate
47	p.Arg47Pro		8480	4396	5.45	7.18					-1.16E+01	Indeterminate			-1.16E+01	Indeterminate
47	p.Arg47Leu		8455	2369	5.43	3.87					-3.70E-01	Neutral			-3.70E-01	Neutral
47	p.Arg47Asp		6458	7409	4.15	12.10					-5.32E+01	Deleterious			-5.32E+01	Deleterious
47	p.Arg47Glu		9515	3930	6.11	6.42					-3.92E+00	Neutral			-3.92E+00	Neutral
47	p.Arg47Ala		5555	1795	3.57	2.93					-2.77E+00	Neutral			-2.77E+00	Neutral
47	p.Arg47Gly		7345	3399	4.72	5.55					-9.18E+00	Indeterminate			-9.18E+00	Indeterminate
47	p.Arg47Val		7749	2449	4.98	4.00					-1.23E+00	Neutral			-1.23E+00	Neutral
47	p.Arg47Tyr		9908	3661	6.36	5.98					-1.99E+00	Neutral			-1.99E+00	Neutral
47	p.Arg47Cys		9687	2786	6.22	4.55					-3.09E-01	Neutral			-3.09E-01	Neutral
47	p.Arg47Trp		8071	3147	5.18	5.14					-3.86E+00	Neutral			-3.86E+00	Neutral
47	p.Arg47Phe		11181	3229	7.18	5.27					-2.00E-01	Neutral			-2.00E-01	Neutral
48	p.Pro48Asn		2904	1850	5.11	6.04					-5.32E+01	Deleterious			-5.32E+01	Deleterious
48	p.Pro48Lys		3193	2371	5.62	7.75					-5.32E+01	Deleterious			-5.32E+01	Deleterious
48	p.Pro48Thr	Pathogenic	2253	231	3.97	0.75					-1.93E+01	Indeterminate			-1.93E+01	Indeterminate
48	p.Pro48Arg		2576	1662	4.53	5.43					-5.32E+01	Deleterious			-5.32E+01	Deleterious
48	p.Pro48Ser		2673	193	4.70	0.63					-4.30E+00	Neutral			-4.30E+00	Neutral
48	p.Pro48Ile		3400	1282	5.98	4.19					-5.32E+01	Deleterious			-5.32E+01	Deleterious
48	p.Pro48Met		3721	2133	6.55	6.97					-5.32E+01	Deleterious			-5.32E+01	Deleterious
48	p.Pro48His		2798	1554	4.92	5.08					-5.32E+01	Deleterious			-5.32E+01	Deleterious
48	p.Pro48Gln		2850	2049	5.02	6.69					-5.32E+01	Deleterious			-5.32E+01	Deleterious
48	p.Pro48Pro	Synonymous	1620	78	2.85	0.25					-1.06E+00	Neutral			-1.06E+00	Neutral
48	p.Pro48Leu		4208	3546	7.41	11.59					-5.32E+01	Deleterious			-5.32E+01	Deleterious
48	p.Pro48Asp		3106	2678	5.47	8.75					-5.32E+01	Deleterious			-5.32E+01	Deleterious
48	p.Pro48Glu		2942	2356	5.18	7.70					-5.32E+01	Deleterious			-5.32E+01	Deleterious
48	p.Pro48Ala		1831	66	3.22	0.22					-3.82E-02	Neutral			-3.82E-02	Neutral
48	p.Pro48Gly		2682	268	4.72	0.88					-1.50E+01	Indeterminate			-1.50E+01	Indeterminate
48	p.Pro48Val		2679	279	4.72	0.91					-1.70E+01	Indeterminate			-1.70E+01	Indeterminate
48	p.Pro48Tyr		2687	2346	4.73	7.66					-5.32E+01	Deleterious			-5.32E+01	Deleterious
48	p.Pro48Cys		1917	219	3.37	0.72					-2.99E+01	Indeterminate			-2.98E+01	Indeterminate
48	p.Pro48Trp		3810	3295	6.71	10.77					-5.32E+01	Deleterious			-5.32E+01	Deleterious
48	p.Pro48Phe		2967	2151	5.22	7.03					-5.32E+01	Deleterious			-5.32E+01	Deleterious
49	p.Ile49Asn		2237	1425	4.04	5.04					-5.32E+01	Deleterious			-5.32E+01	Deleterious
49	p.Ile49Lys		2695	2221	4.87	7.85					-5.32E+01	Deleterious			-5.32E+01	Deleterious
49	p.Ile49Thr	Likely pathogenic	2164	487	3.91	1.72					-3.01E+01	Indeterminate			-3.00E+01	Indeterminate
49	p.Ile49Arg		3841	2810	6.94	9.94					-5.32E+01	Deleterious			-5.32E+01	Deleterious
49	p.Ile49Ser	Likely pathogenic	3236	1418	5.85	5.01					-5.32E+01	Deleterious			-5.32E+01	Deleterious
49	p.Ile49Ile	Synonymous	1978	192	3.57	0.68					-1.06E+00	Neutral			-1.06E+00	Neutral

49	p.Ile49Met			2492	263	4.50	0.93	-1.12E+00	Neutral	-1.12E+00	Neutral
49	p.Ile49His			2474	1184	4.47	4.19	-5.32E+01	Deleterious	-5.32E+01	Deleterious
49	p.Ile49Gln			3367	1300	6.08	4.60	-5.32E+01	Deleterious	-5.32E+01	Deleterious
49	p.Ile49Pro			3123	2176	5.64	7.69	-5.32E+01	Deleterious	-5.32E+01	Deleterious
49	p.Ile49Leu			2625	246	4.74	0.87	-3.69E-01	Neutral	-3.69E-01	Neutral
49	p.Ile49Asp			3604	3212	6.51	11.36	-5.32E+01	Deleterious	-5.32E+01	Deleterious
49	p.Ile49Glu			3855	3143	6.96	11.11	-5.32E+01	Deleterious	-5.32E+01	Deleterious
49	p.Ile49Ala			2079	459	3.76	1.62	-2.97E+01	Indeterminate	-2.96E+01	Indeterminate
49	p.Ile49Gly			3080	2656	5.56	9.39	-5.32E+01	Deleterious	-5.32E+01	Deleterious
49	p.Ile49Val			2891	306	5.22	1.08	-7.90E-01	Neutral	-7.90E-01	Neutral
49	p.Ile49Tyr			1968	1294	3.56	4.58	-5.32E+01	Deleterious	-5.32E+01	Deleterious
49	p.Ile49Cys			1784	321	3.22	1.14	-1.96E+01	Indeterminate	-1.96E+01	Indeterminate
49	p.Ile49Trp			3459	2624	6.25	9.28	-5.32E+01	Deleterious	-5.32E+01	Deleterious
49	p.Ile49Phe			2399	543	4.33	1.92	-2.80E+01	Indeterminate	-2.80E+01	Indeterminate
50	p.Gln50Asn			6179	763	3.25	0.67	-1.25E+01	Indeterminate	-1.25E+01	Indeterminate
50	p.Gln50Lys			7027	8327	3.70	7.36	-5.32E+01	Deleterious	-5.32E+01	Deleterious
50	p.Gln50Thr			5937	695	3.12	0.61	-1.09E+01	Indeterminate	-1.09E+01	Indeterminate
50	p.Gln50Arg		Deleterious	7334	11605	3.86	10.26	-5.32E+01	Deleterious	-5.32E+01	Deleterious
50	p.Gln50Ser			8421	926	4.43	0.82	-5.39E+00	Neutral	-5.39E+00	Neutral
50	p.Gln50Ile			8078	3493	4.25	3.09	-5.32E+01	Deleterious	-5.32E+01	Deleterious
50	p.Gln50Met			9962	960	5.24	0.85	-2.05E+00	Neutral	-2.05E+00	Neutral
50	p.Gln50His			10223	4095	5.38	3.62	-5.32E+01	Deleterious	-5.32E+01	Deleterious
50	p.Gln50Gln	Synonymous		12106	1126	6.37	1.00	-1.06E+00	Neutral	-1.06E+00	Neutral
50	p.Gln50Pro			9549	16066	5.03	14.20	-5.32E+01	Deleterious	-5.32E+01	Deleterious
50	p.Gln50Leu			11007	1915	5.79	1.69	-2.00E+01	Indeterminate	-2.00E+01	Indeterminate
50	p.Gln50Asp			10090	6468	5.31	5.72	-5.32E+01	Deleterious	-5.32E+01	Deleterious
50	p.Gln50Glu			12681	1243	6.67	1.10	-1.37E+00	Neutral	-1.37E+00	Neutral
50	p.Gln50Ala			7885	1001	4.15	0.88	-1.04E+01	Indeterminate	-1.04E+01	Indeterminate
50	p.Gln50Gly			7736	1057	4.07	0.93	-1.36E+01	Indeterminate	-1.36E+01	Indeterminate
50	p.Gln50Val			14010	5143	7.37	4.55	-5.32E+01	Deleterious	-5.32E+01	Deleterious
50	p.Gln50Tyr			13564	21314	7.14	18.84	-5.32E+01	Deleterious	-5.32E+01	Deleterious
50	p.Gln50Cys			9439	1625	4.97	1.44	-2.24E+01	Indeterminate	-2.24E+01	Indeterminate
50	p.Gln50Trp			10854	15054	5.71	13.30	-5.32E+01	Deleterious	-5.32E+01	Deleterious
50	p.Gln50Phe			7924	10276	4.17	9.08	-5.32E+01	Deleterious	-5.32E+01	Deleterious
51	p.Val51Asn			2467	455	1.70	0.40	-5.32E+01	Deleterious	-5.32E+01	Deleterious
51	p.Val51Lys			3393	9087	2.34	8.00	-5.32E+01	Deleterious	-5.32E+01	Deleterious
51	p.Val51Thr			3961	298	2.73	0.26	-4.90E+00	Neutral	-4.90E+00	Neutral
51	p.Val51Arg			3536	10023	2.44	8.83	-5.32E+01	Deleterious	-5.32E+01	Deleterious
51	p.Val51Ser			7469	443	5.14	0.39	-2.22E-01	Neutral	-2.22E-01	Neutral
51	p.Val51Ile			8945	908	6.16	0.80	-5.58E+00	Neutral	-5.58E+00	Neutral
51	p.Val51Met			6116	1205	4.21	1.06	-5.32E+01	Deleterious	-5.32E+01	Deleterious
51	p.Val51His			6561	10183	4.52	8.97	-5.32E+01	Deleterious	-5.32E+01	Deleterious
51	p.Val51Gln			10283	2904	7.08	2.56	-5.32E+01	Deleterious	-5.32E+01	Deleterious
51	p.Val51Pro			12358	22046	8.51	19.41	-5.32E+01	Deleterious	-5.32E+01	Deleterious
51	p.Val51Leu			9977	848	6.87	0.75	-1.84E+00	Neutral	-1.84E+00	Neutral
51	p.Val51Asp			11158	18353	7.68	16.16	-5.32E+01	Deleterious	-5.32E+01	Deleterious
51	p.Val51Glu			7184	13435	4.95	11.83	-5.32E+01	Deleterious	-5.32E+01	Deleterious
51	p.Val51Ala			6834	308	4.71	0.27	-9.40E-03	Neutral	-9.40E-03	Neutral
51	p.Val51Gly			5966	473	4.11	0.42	-3.26E+00	Neutral	-3.26E+00	Neutral
51	p.Val51Val	Synonymous		10471	833	7.21	0.73	-1.06E+00	Neutral	-1.06E+00	Neutral
51	p.Val51Tyr			4583	4152	3.16	3.66	-5.32E+01	Deleterious	-5.32E+01	Deleterious
51	p.Val51Cys			13104	988	9.02	0.87	-3.73E-01	Neutral	-3.73E-01	Neutral
51	p.Val51Trp			7433	14300	5.12	12.59	-5.32E+01	Deleterious	-5.32E+01	Deleterious
51	p.Val51Phe			3400	2313	2.34	2.04	-5.32E+01	Deleterious	-5.32E+01	Deleterious
52	p.Met52Asn			4478	6062	2.15	2.76	-1.64E+01	Indeterminate	-1.64E+01	Indeterminate
52	p.Met52Lys			3389	6716	1.63	3.06	-5.32E+01	Deleterious	-5.32E+01	Deleterious
52	p.Met52Thr			7325	6547	3.52	2.98	-1.17E+00	Neutral	-1.17E+00	Neutral
52	p.Met52Arg			3644	7476	1.75	3.41	-5.32E+01	Deleterious	-5.32E+01	Deleterious
52	p.Met52Ser			12692	10076	6.09	4.59	-6.65E-02	Neutral	-6.65E-02	Neutral
52	p.Met52Ile			4566	5019	2.19	2.29	-7.82E+00	Indeterminate	-7.82E+00	Indeterminate
52	p.Met52Met	Synonymous		4922	3803	2.36	1.73	-1.06E+00	Neutral	-1.06E+00	Neutral
52	p.Met52His			8903	10578	4.27	4.82	-4.19E+00	Neutral	-4.19E+00	Neutral
52	p.Met52Gln			8262	7816	3.97	3.56	-1.31E+00	Neutral	-1.31E+00	Neutral
52	p.Met52Pro			11304	19362	5.43	8.83	-1.31E+01	Indeterminate	-1.31E+01	Indeterminate
52	p.Met52Leu			19024	15490	9.13	7.06	-1.49E-02	Neutral	-1.49E-02	Neutral
52	p.Met52Asp			7647	15009	3.67	6.84	-2.82E+01	Indeterminate	-2.82E+01	Indeterminate
52	p.Met52Glu			10223	16152	4.91	7.36	-1.11E+01	Indeterminate	-1.11E+01	Indeterminate
52	p.Met52Ala			20519	16257	9.85	7.41	-6.58E-03	Neutral	-6.58E-03	Neutral
52	p.Met52Gly			28403	21442	13.64	9.77	-4.04E-04	Neutral	-4.04E-04	Neutral
52	p.Met52Val			16148	12224	7.75	5.57	-1.12E-02	Neutral	-1.12E-02	Neutral
52	p.Met52Tyr			5210	6051	2.50	2.76	-8.17E+00	Indeterminate	-8.17E+00	Indeterminate
52	p.Met52Cys			15883	11778	7.63	5.37	-8.88E-03	Neutral	-8.88E-03	Neutral
52	p.Met52Trp			10918	16716	5.24	7.62	-9.17E+00	Indeterminate	-9.17E+00	Indeterminate
52	p.Met52Phe			4809	4797	2.31	2.19	-4.81E+00	Neutral	-4.81E+00	Neutral
53	p.Met53Asn			5148	1876	4.45	2.67	-3.58E+01	Indeterminate	-3.30E+01	Indeterminate
53	p.Met53Lys			6247	1516	5.40	2.16	-8.88E+00	Indeterminate	-8.88E+00	Indeterminate
53	p.Met53Thr			5939	2515	5.13	3.58	-4.53E+01	Indeterminate	-3.32E+01	Indeterminate
53	p.Met53Arg			5012	915	4.33	1.30	-3.54E+00	Neutral	-3.54E+00	Neutral
53	p.Met53Ser			5763	1238	4.98	1.76	-6.07E+00	Indeterminate	-6.07E+00	Indeterminate
53	p.Met53Ile	Pathogenic		5291	4631	4.57	6.59	-5.32E+01	Deleterious	-5.32E+01	Deleterious
53	p.Met53Met	Synonymous		3139	402	2.71	0.57	-1.06E+00	Neutral	-1.06E+00	Neutral
53	p.Met53His			7214	3027	6.23	4.31	-3.83E+01	Indeterminate	-3.32E+01	Indeterminate
53	p.Met53Gln			8241	1527	7.12	2.17	-1.49E+00	Neutral	-1.49E+00	Neutral
53	p.Met53Pro			5318	6610	4.59	9.40	-5.32E+01	Deleterious	-5.32E+01	Deleterious
53	p.Met53Leu			6554	1519	5.66	2.16	-6.97E+00	Indeterminate	-6.97E+00	Indeterminate
53	p.Met53Asp			5058	9025	4.37	12.84	-5.32E+01	Deleterious	-5.32E+01	Deleterious
53	p.Met53Glu			7104	3670	6.14	5.22	-5.32E+01	Deleterious	-5.32E+01	Deleterious
53	p.Met53Ala			6055	1223	5.23	1.74	-4.27E+00	Neutral	-4.27E+00	Neutral
53	p.Met53Gly			5478	4287	4.73	6.10	-5.32E+01	Deleterious	-5.32E+01	Deleterious
53	p.Met53Val	Likely pathogenic		5837	5714	5.04	8.13	-5.32E+01	Deleterious	-5.32E+01	Deleterious
53	p.Met53Tyr			5550	4771	4.80	6.79	-5.32E+01	Deleterious	-5.32E+01	Deleterious
53	p.Met53Cys			4558	1062	3.94	1.51	-1.12E+01	Indeterminate	-1.12E+01	Indeterminate
53	p.Met53Trp			7931	11517	6.85	16.38	-5.32E+01	Deleterious	-5.32E+01	Deleterious
53	p.Met53Phe			4299	3259	3.71	4.64	-5.32E+01	Deleterious	-5.32E+01	Deleterious
54	p.Met54Asn			4729	6229	4.82	4.68	-1.00E+00	Neutral	-1.00E+00	Neutral
54	p.Met54Lys			1457	2678	1.48	2.01	-2.35E+01	Indeterminate	-2.35E+01	Indeterminate
54	p.Met54Thr			9921	9389	10.11	7.06	-6.02E-04	Neutral	-6.02E-04	Neutral
54	p.Met54Arg			2692	6197	2.74	4.66	-2.49E+01	Indeterminate	-2.49E+01	Indeterminate
54	p.Met54Ser			7785	8580	7.93	6.45	-2.84E-02	Neutral	-2.84E-02	Neutral
54	p.Met54Ile			4949	5748	5.04	4.32	-3.06E-01	Neutral	-3.06E-01	Neutral
54	p.Met54Met	Synonymous		1901	1865	1.94	1.40	-1.06E+00	Neutral	-1.06E+00	Neutral
54	p.Met54His			1990	2453	2.03	1.84	-3.79E+00	Neutral	-3.79E+00	Neutral
54	p.Met54Gln			3851	6289	3.92	4.73	-5.09E+00	Neutral	-5.09E+00	Neutral
54	p.Met54Pro			7720	20805	7.86	15.63	-1.42E+01	Indeterminate	-1.42E+01	Indeterminate
54	p.Met54Leu			5644	6063	5.75	4.56	-8.18E-02	Neutral	-8.18E-02	Neutral
54	p.Met54Asp			5244	12085	5.34	9.08	-1.29E+01	Indeterminate	-1.29E+01	Indeterminate
54	p.Met54Glu			3404	6990	3.47	5.25	-1.42E+01	Indeterminate	-1.42E+01	Indeterminate
54	p.Met54Ala			4236	4218	4.32	3.17	-1.03E-01	Neutral	-1.03E-01	Neutral
54	p.Met54Gly			6165	6242	6.28	4.69	-2.56E-02	Neutral	-2.56E-02	Neutral
54	p.Met54Val			5273	5438	5.37	4.09	-6.54E-02	Neutral	-6.54E-02	Neutral
54	p.Met54Tyr			2374	2422	2.42	1.82	-8.20E-01	Neutral	-8.20E-01	Neutral
54	p.Met54Cys			7763	6885	7.91	5.17	-8.22E-04	Neutral	-8.22E-04	Neutral
54	p.Met54Trp			4101	4916	4.18	3.69	-7.00E-01	Neutral	-7.00E-01	Neutral
54	p.Met54Phe			6961	7588	7.09	5.70	-4.06E-02	Neutral	-4.06E-02	Neutral
55	p.Gly55Asn			7992	2142	3.29	3.35	-5.32E+01	Deleterious	-5.32E+01	Deleterious
55	p.Gly55Lys			10951	2798	4.51	4.37	-5.32E+01	Deleterious	-5.32E+01	Deleterious
55	p.Gly55Thr			17033	3602	7.01	5.63	-5.32E+01	Deleterious	-5.32E+01	Deleterious
55	p.Gly55Arg			11124	2826	4.58	4.42	-5.32E+01	Deleterious	-5.32E+01	Deleterious
55	p.Gly55Ser			12413	706	5.11	1.10	-3.27E+01	Indeterminate	-3.19E+01	Indeterminate
55	p.Gly55Ile			11813	2953	4.86	4.62	-5.32E+01	Deleterious	-5.32E+01	Deleterious
55	p.Gly55Met			10258	3064	4.22	4.79	-5.32E+01	Deleterious	-5.32E+01	Deleterious
55	p.Gly55His			14055	4676	5.78	7.31	-5.32E+01	Deleterious	-5.32E+01	Deleterious
55	p.Gly55Gln			14040	3922	5.78	6.13	-5.32E+01	Deleterious	-5.32E+01	Deleterious
55	p.Gly55Pro			24068	10469	9.90	16.36	-5.32E+01	Deleterious	-5.32E+01	Deleterious
55	p.Gly55Leu			21350	7759	8.79	12.13	-5.32E+01	Deleterious	-5.32E+01	Deleterious
55	p.Gly55Asp			12218	4275	5.03	6.68	-5.32E+01	Deleterious	-5.32E+01	Deleterious
55	p.Gly55Glu			16079	4459	6.62	6.97	-5.32E+01	Deleterious	-5.32E+01	Deleterious
55	p.Gly55Ala			8380	288	3.45	0.45	-1.10E+01	Indeterminate	-1.10E+01	Indeterminate
55	p.Gly55Gly	Synonymous		8104	172	3.34	0.27	-1.06E+00	Neutral	-1.06E+00	Neutral
55	p.Gly55Val			7187	2149	2.96	3.36	-5.32E+01	Deleterious	-5.32E+01	Deleterious
55	p.Gly55Tyr			10835	2494	4.46	3.90	-5.32E+01	Deleterious	-5.32E+01	Deleterious
55	p.Gly55Cys			9250	737	3.81	1.15	-5.32E+01	Deleterious	-5.32E+01	Deleterious
55	p.Gly55Trp			8904	2798	3.66	4.37	-5.32E+01	Deleterious	-5.32E+01	Deleterious
55	p.Gly55Phe			6940	1690	2.86	2.64	-5.32E+01	Deleterious	-5.32E+01	Deleterious
56	p.Ser56Asn			15782	229	5.04	0.81	-8.15E-10	Neutral	-6.71E-10	Neutral
56	p.Ser56Lys			21348	835	6.82	2.94	-9.71E-02	Neutral	-9.71E-02	Neutral
56	p.Ser56Thr			12135	459	3.88	1.62	-4.71E-01	Neutral	-4.71E-01	Neutral
56	p.Ser56Arg			12834	231	4.10	0.81	-1.41E-06	Neutral	-1.41E-06	Neutral
56	p.Ser56Ser	Synonymous		18053	863	5.77	3.04	-1.06E+00	Neutral	-1.06E+00	Neutral

56	p.Ser56Ile	Pathogenic		15771	4860	5.04	17.13					-5.32E+01	Deleterious			-5.32E+01	Deleterious
56	p.Ser56Met			9103	181	2.91	0.64					-1.66E-04	Neutral			-1.66E-04	Neutral
56	p.Ser56His			16781	342	5.36	1.21					-3.27E-06	Neutral			-3.27E-06	Neutral
56	p.Ser56Gln			13828	378	4.42	1.33					-4.63E-03	Neutral			-4.63E-03	Neutral
56	p.Ser56Pro			15799	9165	5.05	32.30					-5.32E+01	Deleterious			-5.32E+01	Deleterious
56	p.Ser56Leu			18803	1683	6.01	5.93					-1.75E+01	Indeterminate			-1.75E+01	Indeterminate
56	p.Ser56Asp			15719	594	5.02	2.09					-2.05E-01	Neutral			-2.05E-01	Neutral
56	p.Ser56Glu			16419	941	5.25	3.32					-3.79E+00	Neutral			-3.79E+00	Neutral
56	p.Ser56Ala			17828	655	5.70	2.31					-9.40E-02	Neutral			-9.40E-02	Neutral
56	p.Ser56Gly			20474	1154	6.54	4.07					-2.34E+00	Neutral			-2.34E+00	Neutral
56	p.Ser56Val			17532	973	5.60	3.43					-2.86E+00	Neutral			-2.86E+00	Neutral
56	p.Ser56Tyr			7771	187	2.48	0.66					-1.28E-02	Neutral			-1.28E-02	Neutral
56	p.Ser56Cys			14573	299	4.66	1.05					-1.13E-05	Neutral			-1.13E-05	Neutral
56	p.Ser56Trp			17186	3817	5.49	13.45					-5.32E+01	Deleterious			-5.32E+01	Deleterious
56	p.Ser56Phe			15288	528	4.88	1.86					-8.26E-02	Neutral			-8.26E-02	Neutral
57	p.Ala57Asn			4228	707	5.51	4.84	12028	2228	5.44	4.68	-8.42E-07	Neutral	-1.69E+00	Neutral	-5.69E-01	Neutral
57	p.Ala57Lys			3617	548	4.71	3.75	10968	2317	4.96	4.86	-2.63E-07	Neutral	-4.13E+00	Neutral	-2.18E+00	Neutral
57	p.Ala57Thr			2864	570	3.73	3.90	8869	1879	4.01	3.95	-6.42E-04	Neutral	-5.78E+00	Neutral	-3.45E+00	Neutral
57	p.Ala57Arg			3189	550	4.16	3.77	11295	1899	5.10	3.99	-1.57E-05	Neutral	-1.01E+00	Neutral	-2.42E-01	Neutral
57	p.Ala57Ser			4843	991	6.31	6.79	14163	3613	6.40	7.59	-3.20E-05	Neutral	-6.57E+00	Neutral	-4.10E+00	Neutral
57	p.Ala57Ile			2093	525	2.73	3.60	10521	1993	4.75	4.18	-1.10E-01	Neutral	-2.51E+00	Neutral	-1.13E+00	Neutral
57	p.Ala57Met			3187	502	4.15	3.44	8084	1645	3.65	3.45	-1.92E-06	Neutral	-5.54E+00	Neutral	-3.27E+00	Neutral
57	p.Ala57His			4842	956	6.31	6.55	13594	1983	6.14	4.16	-1.45E-05	Neutral	-1.62E-01	Neutral	-8.49E-03	Neutral
57	p.Ala57Gln			4779	911	6.23	6.24	11318	2326	5.11	4.88	-7.21E-06	Neutral	-3.42E+00	Neutral	-1.67E+00	Neutral
57	p.Ala57Pro			4088	887	5.33	6.08	10929	2644	4.94	5.55	-3.47E-04	Neutral	-7.51E+00	Indeterminate	-4.88E+00	Neutral
57	p.Ala57Leu			3855	603	5.02	4.13	9126	2396	4.12	5.03	-3.45E-07	Neutral	-1.26E+01	Indeterminate	-9.30E+00	Indeterminate
57	p.Ala57Asp			4487	1216	5.85	8.33	13571	2300	6.13	4.83	-1.01E-02	Neutral	-6.69E-01	Neutral	-1.23E-01	Neutral
57	p.Ala57Glu			3993	794	5.20	5.44	13025	3886	5.89	8.16	-6.86E-05	Neutral	-1.30E+01	Indeterminate	-9.67E+00	Indeterminate
57	p.Ala57Ala	Synonymous		2787	983	3.63	6.73	9862	1597	4.46	3.35	-1.06E+00	Neutral	-1.06E+00	Neutral	-8.18E-01	Neutral
57	p.Ala57Gly			5677	999	7.40	6.84	13161	2709	5.95	5.69	-2.63E-07	Neutral	-2.64E+00	Neutral	-1.14E+00	Neutral
57	p.Ala57Val	Likely benign		4184	783	5.45	5.36	14947	2673	6.75	5.61	-1.29E-05	Neutral	-7.94E-01	Neutral	-1.61E-01	Neutral
57	p.Ala57Tyr			3130	578	4.08	3.96	8794	2642	3.97	5.55	-7.93E-05	Neutral	-1.99E+01	Indeterminate	-1.60E+01	Indeterminate
57	p.Ala57Cys			4967	664	6.47	4.55	11591	2870	5.24	6.03	-5.42E-10	Neutral	-7.62E+00	Indeterminate	-4.97E+00	Neutral
57	p.Ala57Trp			3220	520	4.20	3.56	8448	2110	3.82	4.43	-3.21E-06	Neutral	-1.16E+01	Indeterminate	-8.41E+00	Indeterminate
57	p.Ala57Phe			2695	310	3.51	2.12	7000	1919	3.16	4.03	-2.37E-09	Neutral	-1.90E+01	Indeterminate	-1.51E+01	Indeterminate
58	p.Arg58Asn			11388	6167	5.68	5.29					-3.71E-04	Neutral			-3.71E-04	Neutral
58	p.Arg58Lys			12667	6509	6.32	5.59					-6.17E-05	Neutral			-6.17E-05	Neutral
58	p.Arg58Thr			4945	2887	2.47	2.48					-9.58E-02	Neutral			-9.58E-02	Neutral
58	p.Arg58Arg	Synonymous		4309	3109	2.15	2.67					-1.06E+00	Neutral			-1.06E+00	Neutral
58	p.Arg58Ser			8923	4891	4.45	4.20					-2.10E-03	Neutral			-2.10E-03	Neutral
58	p.Arg58Ile			14106	8230	7.04	7.06					-3.97E-04	Neutral			-3.97E-04	Neutral
58	p.Arg58Met			8462	4305	4.22	3.70					-7.43E-04	Neutral			-7.43E-04	Neutral
58	p.Arg58His			10078	4647	5.03	3.99					-3.07E-05	Neutral			-3.07E-05	Neutral
58	p.Arg58Gln			8727	5863	4.35	5.03					-5.28E-02	Neutral			-5.28E-02	Neutral
58	p.Arg58Pro			9411	7328	4.70	6.29					-2.18E-01	Neutral			-2.18E-01	Neutral
58	p.Arg58Leu			10691	6368	5.33	5.47					-3.14E-03	Neutral			-3.14E-03	Neutral
58	p.Arg58Asp			10734	5563	5.36	4.78					-2.29E-04	Neutral			-2.29E-04	Neutral
58	p.Arg58Glu			12402	7460	6.19	6.40					-1.57E-03	Neutral			-1.57E-03	Neutral
58	p.Arg58Ala			8563	5464	4.27	4.69					-2.88E-02	Neutral			-2.88E-02	Neutral
58	p.Arg58Gly			9407	6284	4.69	5.39					-3.52E-02	Neutral			-3.52E-02	Neutral
58	p.Arg58Val			13045	8449	6.51	7.25					-4.23E-03	Neutral			-4.23E-03	Neutral
58	p.Arg58Tyr			11421	5102	5.70	4.38					-5.90E-06	Neutral			-5.90E-06	Neutral
58	p.Arg58Cys			10605	5418	5.29	4.65					-1.86E-04	Neutral			-1.86E-04	Neutral
58	p.Arg58Trp			8533	4672	4.26	4.01					-2.67E-03	Neutral			-2.67E-03	Neutral
58	p.Arg58Phe			12024	7781	6.00	6.68					-6.51E-03	Neutral			-6.51E-03	Neutral
59	p.Val59Asn			2415	585	4.15	1.45					-5.32E+01	Deleterious			-5.32E+01	Deleterious
59	p.Val59Lys			3708	3626	6.38	8.99					-5.32E+01	Deleterious			-5.32E+01	Deleterious
59	p.Val59Thr			3352	218	5.77	0.54					-2.12E-01	Neutral			-2.12E-01	Neutral
59	p.Val59Arg			2890	4202	4.97	10.42					-5.32E+01	Deleterious			-5.32E+01	Deleterious
59	p.Val59Ser			2742	260	4.72	0.64					-4.70E+00	Neutral			-4.70E+00	Neutral
59	p.Val59Ile			2316	130	3.98	0.32					-1.64E-01	Neutral			-1.64E-01	Neutral
59	p.Val59Met			2652	203	4.56	0.50					-1.55E+00	Neutral			-1.55E+00	Neutral
59	p.Val59His			2849	3587	4.90	8.90					-5.32E+01	Deleterious			-5.32E+01	Deleterious
59	p.Val59Gln			2872	790	4.94	1.96					-5.32E+01	Deleterious			-5.32E+01	Deleterious
59	p.Val59Pro			3188	3072	5.48	7.62					-5.32E+01	Deleterious			-5.32E+01	Deleterious
59	p.Val59Leu			3295	327	5.67	0.81					-4.37E+00	Neutral			-4.37E+00	Neutral
59	p.Val59Asp			2993	4745	5.15	11.77					-5.32E+01	Deleterious			-5.32E+01	Deleterious
59	p.Val59Glu			3323	3022	5.72	7.50					-5.32E+01	Deleterious			-5.32E+01	Deleterious
59	p.Val59Ala			2057	183	3.54	0.45					-5.43E+00	Neutral			-5.43E+00	Neutral
59	p.Val59Gly	Pathogenic		1783	295	3.07	0.73					-4.27E+01	Indeterminate			-3.32E+01	Indeterminate
59	p.Val59Val	Synonymous		3055	232	5.26	0.58					-1.06E+00	Neutral			-1.06E+00	Neutral
59	p.Val59Tyr			3471	4948	5.97	12.27					-5.32E+01	Deleterious			-5.32E+01	Deleterious
59	p.Val59Cys			2693	193	4.63	0.48					-9.44E-01	Neutral			-9.44E-01	Neutral
59	p.Val59Trp			3706	6000	6.38	14.88					-5.32E+01	Deleterious			-5.32E+01	Deleterious
59	p.Val59Phe			2767	3698	4.76	9.17					-5.32E+01	Deleterious			-5.32E+01	Deleterious
60	p.Ala60Asn			8575	4388	3.78	4.49					-5.32E+01	Deleterious			-5.32E+01	Deleterious
60	p.Ala60Lys			13108	8103	5.78	8.29					-5.32E+01	Deleterious			-5.32E+01	Deleterious
60	p.Ala60Thr			14496	2990	6.39	3.06					-1.18E+00	Neutral			-1.18E+00	Neutral
60	p.Ala60Arg		Deleterious	11246	10734	4.96	10.98					-5.32E+01	Deleterious			-5.32E+01	Deleterious
60	p.Ala60Ser			13309	4554	5.86	4.66					-1.44E+01	Indeterminate			-1.44E+01	Indeterminate
60	p.Ala60Ile			11680	4451	5.15	4.55					-2.25E+01	Indeterminate			-2.25E+01	Indeterminate
60	p.Ala60Met			13714	3222	6.04	3.30					-2.94E+00	Neutral			-2.94E+00	Neutral
60	p.Ala60His			14244	7153	6.28	7.32					-3.83E+01	Indeterminate			-3.32E+01	Indeterminate
60	p.Ala60Gln			13494	8204	5.95	8.40					-5.32E+01	Deleterious			-5.32E+01	Deleterious
60	p.Ala60Pro			8977	2945	3.96	3.01					-1.89E+01	Indeterminate			-1.89E+01	Indeterminate
60	p.Ala60Leu			9673	1966	4.26	2.01					-2.51E+00	Neutral			-2.51E+00	Neutral
60	p.Ala60Asp			10802	5546	4.76	5.68					-4.95E+01	Indeterminate			-3.32E+01	Indeterminate
60	p.Ala60Glu	Likely pathogenic		12346	6697	5.44	6.85					-5.07E+01	Indeterminate			-3.32E+01	Indeterminate
60	p.Ala60Ala	Synonymous		12155	2329	5.36	2.38					-1.06E+00	Neutral			-1.06E+00	Neutral
60	p.Ala60Gly			10466	2098	4.61	2.15					-1.98E+00	Neutral			-1.98E+00	Neutral
60	p.Ala60Val			9945	2357	4.38	2.41					-5.18E+00	Neutral			-5.18E+00	Neutral
60	p.Ala60Tyr			9740	5258	4.29	5.38					-5.32E+01	Deleterious			-5.32E+01	Deleterious
60	p.Ala60Cys			9566	2423	4.21	2.48					-7.26E+00	Indeterminate			-7.26E+00	Indeterminate
60	p.Ala60Trp			8828	5434	3.89	5.56					-5.32E+01	Deleterious			-5.32E+01	Deleterious
60	p.Ala60Phe			10592	6867	4.67	7.03					-5.32E+01	Deleterious			-5.32E+01	Deleterious
61	p.Glu61Asn			14015	2650	5.27	4.43					-1.02E+00	Neutral			-1.02E+00	Neutral
61	p.Glu61Lys			12182	1826	4.58	3.05					-2.01E-01	Neutral			-2.01E-01	Neutral
61	p.Glu61Thr			15061	2530	5.66	4.23					-3.11E-01	Neutral			-3.11E-01	Neutral
61	p.Glu61Arg			12012	2820	4.52	4.71					-4.80E+00	Neutral			-4.80E+00	Neutral
61	p.Glu61Ser			14189	2881	5.34	4.82					-1.62E+00	Neutral			-1.62E+00	Neutral
61	p.Glu61Ile			12732	2674	4.79	4.47					-2.46E+00	Neutral			-2.46E+00	Neutral
61	p.Glu61Met			14829	3250	5.58	5.43					-2.35E+00	Neutral			-2.35E+00	Neutral
61	p.Glu61His			13568	2686	5.10	4.49					-1.51E+00	Neutral			-1.51E+00	Neutral
61	p.Glu61Gln			13805	2206	5.19	3.69					-2.53E-01	Neutral			-2.53E-01	Neutral
61	p.Glu61Pro			10268	11949	3.86	19.97					-5.32E+01	Deleterious			-5.32E+01	Deleterious
61	p.Glu61Leu			17939	3142	6.75	5.25					-2.65E-01	Neutral			-2.65E-01	Neutral
61	p.Glu61Asp			12883	2304	4.85	3.85					-8.32E-01	Neutral			-8.32E-01	Neutral
61	p.Glu61Glu	Synonymous		14218	2715	5.35	4.54					-1.06E+00	Neutral			-1.06E+00	Neutral
61	p.Glu61Ala			13232	2302	4.98	3.85					-6.23E-01	Neutral			-6.23E-01	Neutral
61	p.Glu61Gly			11310	1790	4.25	2.99					-4.32E-01	Neutral			-4.32E-01	Neutral
61	p.Glu61Val			14302	2388	5.38	3.99					-3.46E-01	Neutral			-3.46E-01	Neutral
61	p.Glu61Tyr			11817	2739	4.44	4.58					-4.64E+00	Neutral			-4.64E+00	Neutral
61	p.Glu61Cys			13774	2388	5.18	3.99					-5.41E-01	Neutral			-5.41E-01	Neutral
61	p.Glu61Trp			14023	2781	5.27	4.65					-1.42E+00	Neutral			-1.42E+00	Neutral
61	p.Glu61Phe			9711	1799	3.65	3.01					-2.00E+00	Neutral			-2.00E+00	Neutral
62	p.Leu62Asn			5284	1310	4.68	2.44	3577	4531	4.52	4.40	-2.92E-03	Neutral	-2.05E-02	Neutral	-1.88E-04	Neutral
62	p.Leu62Lys			5818	2856	5.15	5.32	4271	5506	5.40	5.34	-2.78E+00	Neutral	-1.10E-02	Neutral	-1.24E+00	Neutral
62	p.Leu62Thr			4915	1901	4.35	3.54	4822	6010	6.10	5.83	-8.57E-01	Neutral	-3.32E-03	Neutral	-1.86E-01	Neutral
62	p.Leu62Arg			3035	2548	2.69	4.74	2222	1857	2.81	1.80	-3.79E+01	Indeterminate	-2.36E-04	Neutral	-2.86E+01	Indeterminate
62	p.Leu62Ser			3653	1159	3.23	2.16	2675	3190	3.38	3.10	-3.71E-01	Neutral	-3.40E-02	Neutral	-4.81E-02	Neutral
62	p.Leu62Ile			1275	513	1.13	0.96	1037	743	1.31	0.72	-1.06E+01	Indeterminate	-1.55E-03	Neutral	-7.52E+00	Indeterminate
62	p.Leu62Met			7821	2419	6.92	4.50	5702	4735	7.21	4.59	-1.28E-02	Neutral	-1.57E-07	Neutral	-5.61E-05	Neutral
62	p.Leu62His			14346	3742	12.70	6.97	10997	15496	13.91	15.04	-1.03E-05	Neutral	-1.98E-04	Neutral	-1.50E-08	Neutral
62	p.Leu62Gln			10306	14558	9.12	27.11	6533	14265	8.26	13.84	-4.91E+01	Indeterminate	-8.48E-01	Neutral	-2.94E+01	Indeterminate
62	p.Leu62Pro			56	9	0.05	0.02	31	12	0.04	0.01	-1.58E+01	Indeterminate	-1.19E+00	Neutral	-1.33E+01	Indeterminate
62	p.Leu62Leu	Synonymous		3953	1464	3.50	2.73	2244	3542	2.84	3.44	-1.06E+00	Neutral	-1.06E+00	Neutral	-8.18E-01	Neutral
62	p.Leu62Asp			8215	3191	7.27	5.94	5245	9043	6.63	8.78	-1.92E-01	Neutral	-1.95E-01	Neutral	-4.43E-02	Neutral
62	p.Leu62Glu			8076	3464	7.15	6.45	5397	3735	6.83	3.62	-5.21E-01	Neutral	-1.98E-09	Neutral	-7.63E-02	Neutral
62	p.Leu62Ala			5822	2021	5.15	3.76	3711	2780	4.69	2.70	-1.96E-01	Neutral	-4.26E-07	Neutral	-1.22E-02	Neutral
62	p.Leu62Gly			5101	2717	4.52	5.06	3194	5893	4.04	5.72	-5.17E+00	Neutral	-1.39E+00	Neutral	-4.09E+00	Neutral
62	p.Leu62Val			5797	2364	5.13	4.40	4147	3770	5.25	3.66	-8.45E-01	Neutral	-1.73E-05	Neutral	-1.80E-01	Neutral
62	p.Leu62Tyr			5132	3349	4.54	6.24	3645	5500	4.61	5.34	-1.14E+01	Indeterminate	-1.71E-01	Neutral	-8.39E+00	Indeterminate
62	p.Leu62Cys			1257	423	1.11	0.79	849	1112	1.07	1.08	-5.31E+00	Neutral	-2.53E+00	Neutral	-5.15E+00	Neutral
62	p.Leu62Trp			7748	2419	6.86	4.50	5120	7229	6.48	7.01	-1.54E-02	Neutral	-1.75E-02	Neutral	-3.70E-04	Neutral
62	p.Leu62Phe			5365	1278	4.75	2.38	3646	4104	4.61	3.98	-1.31E-03	Neutral	-2.84E-03	Neutral	-5.97E-06	Neutral
63	p.Leu63Asn			13544	5590	7.01	6.89					-5.32E+01	Deleterious			-5.32E+01	Deleterious
63	p.Leu63Lys			6835	4935	3.54	6.09					-5.32E+01	Deleterious			-5.32E+01	Deleterious
63	p.Leu63Thr			10869	4259	5.63	5.25					-5.32E+01	Deleterious			-5.32E+01	Deleterious
63	p.Leu63Arg			9152	4924	4.74	6.07					-5.32E+01	Deleterious			-5.32E+01	Deleterious

63	p.Leu63Ser			8714	4308	4.51	5.31	-5.32E+01	Deleterious	-5.32E+01	Deleterious
63	p.Leu63Ile			10567	462	5.47	0.57	-5.21E+00	Neutral	-5.21E+00	Neutral
63	p.Leu63Met			8538	226	4.42	0.28	-3.02E-01	Neutral	-3.02E-01	Neutral
63	p.Leu63His			11608	6109	6.01	7.53	-5.32E+01	Deleterious	-5.32E+01	Deleterious
63	p.Leu63Gln			7276	4368	3.77	5.39	-5.32E+01	Deleterious	-5.32E+01	Deleterious
63	p.Leu63Pro			10214	6547	5.29	8.07	-5.32E+01	Deleterious	-5.32E+01	Deleterious
63	p.Leu63Leu	Synonymous		8789	273	4.55	0.34	-1.06E+00	Neutral	-1.06E+00	Neutral
63	p.Leu63Asp			6798	6741	3.52	8.31	-5.32E+01	Deleterious	-5.32E+01	Deleterious
63	p.Leu63Glu			13660	6285	7.07	7.75	-5.32E+01	Deleterious	-5.32E+01	Deleterious
63	p.Leu63Ala			9691	4744	5.02	5.85	-5.32E+01	Deleterious	-5.32E+01	Deleterious
63	p.Leu63Gly			8561	5277	4.43	6.51	-5.32E+01	Deleterious	-5.32E+01	Deleterious
63	p.Leu63Val			6705	689	3.47	0.85	-5.32E+01	Deleterious	-5.32E+01	Deleterious
63	p.Leu63Tyr			15623	7883	8.09	9.72	-5.32E+01	Deleterious	-5.32E+01	Deleterious
63	p.Leu63Cys			7726	791	4.00	0.98	-5.32E+01	Deleterious	-5.32E+01	Deleterious
63	p.Leu63Trp			11790	6222	6.11	7.67	-5.32E+01	Deleterious	-5.32E+01	Deleterious
63	p.Leu63Phe			6454	448	3.34	0.55	-3.80E+01	Indeterminate	-3.32E+01	Indeterminate
64	p.Leu64Asn			12833	6821	4.39	5.64	-1.11E+01	Indeterminate	-1.11E+01	Indeterminate
64	p.Leu64Lys			16339	6603	5.59	5.46	-2.27E+00	Neutral	-2.27E+00	Neutral
64	p.Leu64Thr			14425	4621	4.94	3.82	-6.00E-01	Neutral	-6.00E-01	Neutral
64	p.Leu64Arg			20825	9191	7.13	7.60	-2.37E+00	Neutral	-2.37E+00	Neutral
64	p.Leu64Ser			13437	4243	4.60	3.51	-6.47E-01	Neutral	-6.47E-01	Neutral
64	p.Leu64Ile			6398	2634	2.19	2.18	-1.02E+01	Indeterminate	-1.02E+01	Indeterminate
64	p.Leu64Met			11781	4017	4.03	3.32	-1.56E+00	Neutral	-1.56E+00	Neutral
64	p.Leu64His			21371	9431	7.31	7.80	-2.25E+00	Neutral	-2.25E+00	Neutral
64	p.Leu64Gln			15398	7296	5.27	6.03	-5.58E+00	Neutral	-5.58E+00	Neutral
64	p.Leu64Pro			1303	1822	0.45	1.51	-5.32E+01	Deleterious	-5.32E+01	Deleterious
64	p.Leu64Leu	Synonymous		15457	5454	5.29	4.51	-1.06E+00	Neutral	-1.06E+00	Neutral
64	p.Leu64Asp			14256	9066	4.88	7.50	-1.76E+01	Indeterminate	-1.76E+01	Indeterminate
64	p.Leu64Glu			14674	7300	5.02	6.04	-7.32E+00	Indeterminate	-7.32E+00	Indeterminate
64	p.Leu64Ala			18044	7247	6.17	5.99	-1.79E+00	Neutral	-1.79E+00	Neutral
64	p.Leu64Gly			19651	9506	6.72	7.86	-4.23E+00	Neutral	-4.23E+00	Neutral
64	p.Leu64Val			10172	2912	3.48	2.41	-6.18E-01	Neutral	-6.18E-01	Neutral
64	p.Leu64Tyr			14135	5042	4.84	4.17	-1.41E+00	Neutral	-1.41E+00	Neutral
64	p.Leu64Cys			15527	5308	5.31	4.39	-8.30E-01	Neutral	-8.30E-01	Neutral
64	p.Leu64Trp			20389	7820	6.98	6.47	-9.98E-01	Neutral	-9.98E-01	Neutral
64	p.Leu64Phe			15813	4622	5.41	3.82	-1.91E-01	Neutral	-1.91E-01	Neutral
65	p.Leu65Asn			1677	543	3.60	3.38	-1.20E+01	Indeterminate	-1.20E+01	Indeterminate
65	p.Leu65Lys			2335	440	5.01	2.74	-2.88E-01	Neutral	-2.88E-01	Neutral
65	p.Leu65Thr			2434	656	5.22	4.09	-3.35E+00	Neutral	-3.35E+00	Neutral
65	p.Leu65Arg			2730	595	5.85	3.71	-6.63E-01	Neutral	-6.63E-01	Neutral
65	p.Leu65Ser			2983	833	6.40	5.19	-2.83E+00	Neutral	-2.83E+00	Neutral
65	p.Leu65Ile			1967	438	4.22	2.73	-1.69E+00	Neutral	-1.69E+00	Neutral
65	p.Leu65Met			2487	463	5.33	2.89	-2.06E-01	Neutral	-2.06E-01	Neutral
65	p.Leu65His			1410	419	3.02	2.61	-1.09E+01	Indeterminate	-1.09E+01	Indeterminate
65	p.Leu65Gln			2753	794	5.90	4.95	-3.84E+00	Neutral	-3.84E+00	Neutral
65	p.Leu65Pro	Likely pathogenic		1740	4497	3.73	28.03	-5.32E+01	Deleterious	-5.32E+01	Deleterious
65	p.Leu65Leu	Synonymous		2636	604	5.65	3.76	-1.06E+00	Neutral	-1.06E+00	Neutral
65	p.Leu65Asp			2598	572	5.57	3.57	-8.17E-01	Neutral	-8.17E-01	Neutral
65	p.Leu65Glu			2759	572	5.92	3.57	-4.19E-01	Neutral	-4.19E-01	Neutral
65	p.Leu65Ala			2039	549	4.37	3.42	-4.46E+00	Neutral	-4.46E+00	Neutral
65	p.Leu65Gly			2677	911	5.74	5.68	-8.22E+00	Indeterminate	-8.22E+00	Indeterminate
65	p.Leu65Val			2667	680	5.72	4.24	-2.08E+00	Neutral	-2.08E+00	Neutral
65	p.Leu65Tyr			2428	553	5.21	3.45	-1.23E+00	Neutral	-1.23E+00	Neutral
65	p.Leu65Cys			2119	761	4.54	4.74	-1.31E+01	Indeterminate	-1.31E+01	Indeterminate
65	p.Leu65Trp			2348	753	5.03	4.69	-7.73E+00	Indeterminate	-7.73E+00	Indeterminate
65	p.Leu65Phe			1851	410	3.97	2.56	-1.85E+00	Neutral	-1.85E+00	Neutral
66	p.His66Asn			11379	793	4.83	1.96	-1.65E-01	Neutral	-1.65E-01	Neutral
66	p.His66Lys			12099	869	5.14	2.15	-1.83E-01	Neutral	-1.83E-01	Neutral
66	p.His66Thr			9820	910	4.17	2.25	-2.36E+00	Neutral	-2.36E+00	Neutral
66	p.His66Arg		Neutral	12631	1230	5.36	3.05	-1.93E+00	Neutral	-1.93E+00	Neutral
66	p.His66Ser			10876	904	4.62	2.24	-9.28E-01	Neutral	-9.28E-01	Neutral
66	p.His66Ile			11804	1490	5.01	3.69	-7.66E+00	Indeterminate	-7.66E+00	Indeterminate
66	p.His66Met			11778	860	5.00	2.13	-2.38E-01	Neutral	-2.38E-01	Neutral
66	p.His66His	Synonymous		9315	749	3.96	1.86	-1.06E+00	Neutral	-1.06E+00	Neutral
66	p.His66Gln			13561	1242	5.76	3.08	-1.10E+00	Neutral	-1.10E+00	Neutral
66	p.His66Pro		Deleterious	14437	20924	6.13	51.84	-5.32E+01	Deleterious	-5.32E+01	Deleterious
66	p.His66Leu			13015	1236	5.53	3.06	-1.55E+00	Neutral	-1.55E+00	Neutral
66	p.His66Asp			6613	509	2.81	1.26	-1.70E+00	Neutral	-1.70E+00	Neutral
66	p.His66Glu			13336	1439	5.66	3.56	-3.12E+00	Neutral	-3.12E+00	Neutral
66	p.His66Ala			12769	1270	5.42	3.15	-2.15E+00	Neutral	-2.15E+00	Neutral
66	p.His66Gly			13669	1191	5.81	2.95	-7.45E-01	Neutral	-7.45E-01	Neutral
66	p.His66Val			10655	1203	4.53	2.98	-5.55E+00	Neutral	-5.55E+00	Neutral
66	p.His66Tyr			10205	865	4.33	2.14	-1.24E+00	Neutral	-1.24E+00	Neutral
66	p.His66Cys			12241	683	5.20	1.69	-5.86E-03	Neutral	-5.86E-03	Neutral
66	p.His66Trp			14222	1111	6.04	2.75	-2.50E-01	Neutral	-2.50E-01	Neutral
66	p.His66Phe			11036	888	4.69	2.20	-6.96E-01	Neutral	-6.96E-01	Neutral
67	p.Gly67Asn			1613	199	4.19	1.41	-1.34E-01	Neutral	-1.34E-01	Neutral
67	p.Gly67Lys			1931	310	5.01	2.20	-8.83E-01	Neutral	-8.83E-01	Neutral
67	p.Gly67Thr			1987	510	5.16	3.62	-9.71E+00	Indeterminate	-9.71E+00	Indeterminate
67	p.Gly67Arg		Neutral	2225	231	5.77	1.64	-2.44E-03	Neutral	-2.44E-03	Neutral
67	p.Gly67Ser			1578	182	4.09	1.29	-6.56E-02	Neutral	-6.56E-02	Neutral
67	p.Gly67Ile			1553	1876	4.03	13.31	-5.32E+01	Deleterious	-5.32E+01	Deleterious
67	p.Gly67Met			1703	310	4.42	2.20	-2.62E+00	Neutral	-2.62E+00	Neutral
67	p.Gly67His			2343	275	6.08	1.95	-1.36E-02	Neutral	-1.36E-02	Neutral
67	p.Gly67Gln			1984	187	5.15	1.33	-8.48E-04	Neutral	-8.48E-04	Neutral
67	p.Gly67Pro			2448	5867	6.35	41.61	-5.32E+01	Deleterious	-5.32E+01	Deleterious
67	p.Gly67Leu			2000	298	5.19	2.11	-4.35E-01	Neutral	-4.35E-01	Neutral
67	p.Gly67Asp			1535	195	3.98	1.38	-2.19E-01	Neutral	-2.19E-01	Neutral
67	p.Gly67Glu			1601	201	4.15	1.43	-1.67E-01	Neutral	-1.67E-01	Neutral
67	p.Gly67Ala			2068	206	5.37	1.46	-1.81E-03	Neutral	-1.81E-03	Neutral
67	p.Gly67Gly	Synonymous		1598	247	4.15	1.75	-1.06E+00	Neutral	-1.06E+00	Neutral
67	p.Gly67Val			2037	1509	5.29	10.70	-5.32E+01	Deleterious	-5.32E+01	Deleterious
67	p.Gly67Tyr			2243	315	5.82	2.23	-1.67E-01	Neutral	-1.67E-01	Neutral
67	p.Gly67Cys			1798	317	4.67	2.25	-1.96E+00	Neutral	-1.96E+00	Neutral
67	p.Gly67Trp			1957	441	5.08	3.13	-5.92E+00	Indeterminate	-5.92E+00	Indeterminate
67	p.Gly67Phe			2337	423	6.06	3.00	-1.32E+00	Neutral	-1.32E+00	Neutral
68	p.Ala68Asn			10578	6466	3.32	4.05	-5.32E+01	Deleterious	-5.32E+01	Deleterious
68	p.Ala68Lys			16843	13015	5.29	8.16	-5.32E+01	Deleterious	-5.32E+01	Deleterious
68	p.Ala68Thr			20629	5342	6.48	3.35	-2.88E+00	Neutral	-2.88E+00	Neutral
68	p.Ala68Arg			9508	7401	2.99	4.64	-5.32E+01	Deleterious	-5.32E+01	Deleterious
68	p.Ala68Ser			15363	2849	4.83	1.79	-5.90E-01	Neutral	-5.90E-01	Neutral
68	p.Ala68Ile			11369	7162	3.57	4.49	-5.32E+01	Deleterious	-5.32E+01	Deleterious
68	p.Ala68Met			20851	11459	6.55	7.18	-3.82E+01	Indeterminate	-3.32E+01	Indeterminate
68	p.Ala68His			14597	6003	4.59	3.76	-2.49E+01	Indeterminate	-2.49E+01	Indeterminate
68	p.Ala68Gln			14112	8817	4.43	5.53	-5.32E+01	Deleterious	-5.32E+01	Deleterious
68	p.Ala68Pro			19019	12912	5.98	8.09	-5.32E+01	Deleterious	-5.32E+01	Deleterious
68	p.Ala68Leu	Likely pathogenic		17116	12054	5.38	7.56	-5.32E+01	Deleterious	-5.32E+01	Deleterious
68	p.Ala68Asp			14679	6960	4.61	4.36	-3.55E+01	Indeterminate	-3.29E+01	Indeterminate
68	p.Ala68Glu			19547	9872	6.14	6.19	-3.28E+01	Indeterminate	-3.20E+01	Indeterminate
68	p.Ala68Ala	Synonymous		13663	2629	4.29	1.65	-1.06E+00	Neutral	-1.06E+00	Neutral
68	p.Ala68Gly			15153	3134	4.76	1.96	-1.38E+00	Neutral	-1.38E+00	Neutral
68	p.Ala68Val			20388	6977	6.41	4.37	-1.00E+01	Indeterminate	-1.00E+01	Indeterminate
68	p.Ala68Tyr			10108	7330	3.18	4.60	-5.32E+01	Deleterious	-5.32E+01	Deleterious
68	p.Ala68Cys			21550	4671	6.77	2.93	-8.36E-01	Neutral	-8.36E-01	Neutral
68	p.Ala68Trp			16659	12867	5.23	8.07	-5.32E+01	Deleterious	-5.32E+01	Deleterious
68	p.Ala68Phe			16504	11587	5.19	7.26	-5.32E+01	Deleterious	-5.32E+01	Deleterious
69	p.Glu69Asn			1335	244	3.53	5.26	-5.32E+01	Deleterious	-5.32E+01	Deleterious
69	p.Glu69Lys			1741	274	4.60	5.91	-3.58E+01	Indeterminate	-3.30E+01	Indeterminate
69	p.Glu69Thr			1856	192	4.91	4.14	-9.93E+00	Indeterminate	-9.93E+00	Indeterminate
69	p.Glu69Arg			1897	137	5.02	2.95	-1.75E+00	Neutral	-1.75E+00	Neutral
69	p.Glu69Ser			2201	266	5.82	5.73	-1.39E+01	Indeterminate	-1.39E+01	Indeterminate
69	p.Glu69Ile			2528	228	6.68	4.91	-3.57E+00	Neutral	-3.57E+00	Neutral
69	p.Glu69Met			2022	315	5.35	6.79	-3.09E+01	Indeterminate	-3.07E+01	Indeterminate
69	p.Glu69His			1853	178	4.90	3.84	-7.51E+00	Indeterminate	-7.51E+00	Indeterminate
69	p.Glu69Gln			1715	155	4.53	3.34	-6.51E+00	Indeterminate	-6.51E+00	Indeterminate
69	p.Glu69Pro			1958	639	5.18	13.77	-5.32E+01	Deleterious	-5.32E+01	Deleterious
69	p.Glu69Leu			1850	329	4.89	7.09	-4.53E+01	Indeterminate	-3.32E+01	Indeterminate
69	p.Glu69Asp			1695	145	4.48	3.13	-5.22E+00	Neutral	-5.22E+00	Neutral
69	p.Glu69Glu	Synonymous		1906	128	5.04	2.76	-1.06E+00	Neutral	-1.06E+00	Neutral
69	p.Glu69Ala			1875	102	4.96	2.20	-1.73E-01	Neutral	-1.73E-01	Neutral
69	p.Glu69Gly			1878	211	4.96	4.55	-1.30E+01	Indeterminate	-1.30E+01	Indeterminate
69	p.Glu69Val			2070	301	5.47	6.49	-2.54E+01	Indeterminate	-2.54E+01	Indeterminate
69	p.Glu69Tyr			1544	146	4.08	3.15	-8.93E+00	Indeterminate	-8.93E+00	Indeterminate
69	p.Glu69Cys			2103	199	5.56	4.29	-5.94E+00	Indeterminate	-5.94E+00	Indeterminate
69	p.Glu69Trp			1943	251	5.14	5.41	-1.94E+01	Indeterminate	-1.94E+01	Indeterminate
69	p.Glu69Phe			1855	199	4.90	4.29	-1.13E+01	Indeterminate	-1.13E+01	Indeterminate
70	p.Pro70Asn			1067	1056	7.80	6.39	-3.10E+00	Neutral	-3.10E+00	Neutral
70	p.Pro70Lys			292	407	2.13	2.46	-4.16E+01	Indeterminate	-3.32E+01	Indeterminate
70	p.Pro70Thr			534	451	3.90	2.73	-4.42E+00	Neutral	-4.42E+00	Neutral

70	p.Pro70Arg			1225	962	8.95	5.82					-4.65E-01	Neutral			-4.65E-01	Neutral
70	p.Pro70Ser			611	1001	4.46	6.06					-3.42E+01	Indeterminate			-3.26E+01	Indeterminate
70	p.Pro70Ile			868	595	6.34	3.60					-3.83E-01	Neutral			-3.83E-01	Neutral
70	p.Pro70Met			1191	437	8.70	2.65					-1.73E-06	Neutral			-1.73E-06	Neutral
70	p.Pro70His			344	116	2.51	0.70					-1.74E-03	Neutral			-1.74E-03	Neutral
70	p.Pro70Gln			453	139	3.31	0.84					-4.43E-05	Neutral			-4.43E-05	Neutral
70	p.Pro70Pro	Synonymous		683	490	4.99	2.97					-1.06E+00	Neutral			-1.06E+00	Neutral
70	p.Pro70Leu			725	642	5.30	3.89					-3.39E+00	Neutral			-3.39E+00	Neutral
70	p.Pro70Asp			669	5207	4.89	31.52					-5.32E+01	Deleterious			-5.32E+01	Deleterious
70	p.Pro70Glu			405	1516	2.96	9.18					-5.32E+01	Deleterious			-5.32E+01	Deleterious
70	p.Pro70Ala			506	362	3.70	2.19					-2.01E+00	Neutral			-2.01E+00	Neutral
70	p.Pro70Gly			500	371	3.65	2.25					-2.54E+00	Neutral			-2.54E+00	Neutral
70	p.Pro70Val			703	347	5.14	2.10					-1.80E-02	Neutral			-1.80E-02	Neutral
70	p.Pro70Tyr			458	314	3.35	1.90					-1.90E+00	Neutral			-1.90E+00	Neutral
70	p.Pro70Cys			1010	291	7.38	1.76					-1.48E-08	Neutral			-1.46E-08	Neutral
70	p.Pro70Trp			557	1275	4.07	7.72					-5.32E+01	Deleterious			-5.32E+01	Deleterious
70	p.Pro70Phe			887	542	6.48	3.28					-1.08E-01	Neutral			-1.08E-01	Neutral
71	p.Asn71Asn	Synonymous		7181	171	4.62	0.30					-1.06E+00	Neutral			-1.06E+00	Neutral
71	p.Asn71Lys		Deleterious	8578	3658	5.51	6.33					-5.32E+01	Deleterious			-5.32E+01	Deleterious
71	p.Asn71Thr			7155	580	4.60	1.00					-5.32E+01	Deleterious			-5.32E+01	Deleterious
71	p.Asn71Arg			7444	2468	4.78	4.27					-5.32E+01	Deleterious			-5.32E+01	Deleterious
71	p.Asn71Ser	Likely pathogenic		5704	4086	3.67	7.07					-5.32E+01	Deleterious			-5.32E+01	Deleterious
71	p.Asn71Ile		Deleterious	7003	6534	4.50	11.31					-5.32E+01	Deleterious			-5.32E+01	Deleterious
71	p.Asn71Met			4089	487	2.63	0.84					-5.32E+01	Deleterious			-5.32E+01	Deleterious
71	p.Asn71His			7484	353	4.81	0.61					-2.06E+01	Indeterminate			-2.06E+01	Indeterminate
71	p.Asn71Gln			6723	266	4.32	0.46					-1.35E+01	Indeterminate			-1.35E+01	Indeterminate
71	p.Asn71Pro			8614	17352	5.54	30.02					-5.32E+01	Deleterious			-5.32E+01	Deleterious
71	p.Asn71Leu			7663	5928	4.93	10.26					-5.32E+01	Deleterious			-5.32E+01	Deleterious
71	p.Asn71Asp			9069	498	5.83	0.86					-2.63E+01	Indeterminate			-2.63E+01	Indeterminate
71	p.Asn71Glu			10749	485	6.91	0.84					-1.24E+01	Indeterminate			-1.24E+01	Indeterminate
71	p.Asn71Ala			11275	608	7.25	1.05					-2.05E+01	Indeterminate			-2.05E+01	Indeterminate
71	p.Asn71Gly			6444	162	4.14	0.28					-1.93E+00	Neutral			-1.93E+00	Neutral
71	p.Asn71Val			7148	4897	4.59	8.47					-5.32E+01	Deleterious			-5.32E+01	Deleterious
71	p.Asn71Tyr			8986	3770	5.78	6.52					-5.32E+01	Deleterious			-5.32E+01	Deleterious
71	p.Asn71Cys			6712	283	4.31	0.49					-1.65E+01	Indeterminate			-1.65E+01	Indeterminate
71	p.Asn71Trp			8041	1238	5.17	2.14					-5.32E+01	Deleterious			-5.32E+01	Deleterious
71	p.Asn71Phe			9509	3973	6.11	6.87					-5.32E+01	Deleterious			-5.32E+01	Deleterious
72	p.Cys72Asn			8802	378	5.55	6.25	3988	1635	5.17	4.66	-7.98E+00	Indeterminate	-3.88E-01	Neutral	-5.60E+00	Neutral
72	p.Cys72Lys			6740	268	4.25	4.43	3506	1606	4.54	4.57	-8.34E+00	Indeterminate	-1.31E+00	Neutral	-6.71E+00	Indeterminate
72	p.Cys72Thr			10096	417	6.36	6.90	4817	1804	6.24	5.14	-5.63E+00	Neutral	-7.58E-02	Neutral	-3.40E+00	Neutral
72	p.Cys72Arg			5486	148	3.46	2.45	4246	1315	5.50	3.74	-1.75E+00	Neutral	-9.63E-03	Neutral	-6.11E-01	Neutral
72	p.Cys72Ser			7807	304	4.92	5.03	2617	1397	3.39	3.98	-6.31E+00	Indeterminate	-5.20E+00	Neutral	-8.34E+00	Indeterminate
72	p.Cys72Ile			7381	305	4.65	5.05	3965	1960	5.14	5.58	-8.61E+00	Indeterminate	-1.65E+00	Neutral	-7.24E+00	Indeterminate
72	p.Cys72Met			6176	187	3.89	3.09	2324	1050	3.01	2.99	-2.74E+00	Neutral	-2.77E+00	Neutral	-3.24E+00	Neutral
72	p.Cys72His			4117	134	2.59	2.22	1768	865	2.29	2.46	-7.23E+00	Indeterminate	-6.27E+00	Neutral	-1.01E+01	Indeterminate
72	p.Cys72Gln			6432	304	4.05	5.03	3140	1134	4.07	3.23	-1.59E+01	Indeterminate	-2.46E-01	Neutral	-1.26E+01	Indeterminate
72	p.Cys72Pro			9516	455	6.00	7.53	6995	2515	9.07	7.16	-1.08E+01	Indeterminate	-7.42E-03	Neutral	-7.71E+00	Indeterminate
72	p.Cys72Leu			4547	198	2.87	3.28	1856	930	2.41	2.65	-1.76E+01	Indeterminate	-6.48E+00	Neutral	-2.00E+01	Indeterminate
72	p.Cys72Asp			9589	296	6.04	4.90	4756	2676	6.16	7.62	-1.26E+00	Neutral	-2.50E+00	Neutral	-1.91E+00	Neutral
72	p.Cys72Glu			10351	375	6.52	6.20	4290	2323	5.56	6.61	-2.86E+00	Neutral	-2.44E+00	Neutral	-3.08E+00	Neutral
72	p.Cys72Ala			8098	266	5.10	4.40	2928	1595	3.79	4.54	-2.61E+00	Neutral	-4.81E+00	Neutral	-4.81E+00	Neutral
72	p.Cys72Gly			4850	154	3.06	2.55	3719	984	4.82	2.80	-5.16E+00	Neutral	-1.42E-03	Neutral	-2.97E+00	Neutral
72	p.Cys72Val			9374	377	5.91	6.24	5039	2858	6.53	8.14	-5.58E+00	Neutral	-2.35E+00	Neutral	-5.23E+00	Neutral
72	p.Cys72Tyr			11042	374	6.96	6.19	5452	2304	7.07	6.56	-1.70E+00	Neutral	-1.88E-01	Neutral	-6.82E-01	Neutral
72	p.Cys72Cys	Synonymous		9589	289	6.04	4.78	3417	1506	4.43	4.29	-1.06E+00	Neutral	-1.06E+00	Neutral	-8.18E-01	Neutral
72	p.Cys72Trp			10835	582	6.83	9.63	4896	2290	6.35	6.52	-1.38E+01	Indeterminate	-6.72E-01	Neutral	-1.10E+01	Indeterminate
72	p.Cys72Phe			7835	234	4.94	3.87	3442	2376	4.46	6.76	-1.58E+00	Neutral	-1.01E+01	Indeterminate	-8.51E+00	Indeterminate
73	p.Ala73Asn			6226	776	4.30	5.20	11983	6680	4.44	4.78	-7.10E+00	Indeterminate	-3.83E+00	Neutral	-7.83E+00	Indeterminate
73	p.Ala73Lys			5464	431	3.77	2.89	9537	5074	3.54	3.63	-6.66E-01	Neutral	-4.41E+00	Neutral	-2.90E+00	Neutral
73	p.Ala73Thr			11273	1232	7.78	8.26	21395	10364	7.93	7.42	-1.26E+00	Neutral	-4.10E-01	Neutral	-5.62E-01	Neutral
73	p.Ala73Arg			6861	819	4.74	5.49	13885	6784	5.15	4.86	-5.15E+00	Neutral	-1.35E+00	Neutral	-4.04E+00	Neutral
73	p.Ala73Ser			7157	807	4.94	5.41	13597	7093	5.04	5.08	-3.67E+00	Neutral	-2.13E+00	Neutral	-3.48E+00	Neutral
73	p.Ala73Ile			6816	562	4.71	3.77	11736	5986	4.35	4.29	-5.23E-01	Neutral	-2.49E+00	Neutral	-1.38E+00	Neutral
73	p.Ala73Met			4904	408	3.39	2.73	9903	5188	3.67	3.71	-1.29E+00	Neutral	-3.86E+00	Neutral	-2.96E+00	Neutral
73	p.Ala73His			12140	1111	8.38	7.45	19805	9884	7.34	7.08	-2.31E-01	Neutral	-6.60E-01	Neutral	-1.97E-01	Neutral
73	p.Ala73Gln			4919	518	3.40	3.47	8952	4454	3.32	3.19	-4.81E+00	Neutral	-3.52E+00	Neutral	-5.57E+00	Neutral
73	p.Ala73Pro			8448	860	5.83	5.76	14367	8124	5.33	5.82	-1.49E+00	Neutral	-3.03E+00	Neutral	-2.47E+00	Neutral
73	p.Ala73Leu			6766	587	4.67	3.93	12546	6520	4.65	4.67	-8.07E-01	Neutral	-2.44E+00	Neutral	-1.54E+00	Neutral
73	p.Ala73Asp			4742	488	3.27	3.27	7482	3511	2.77	2.51	-4.57E+00	Neutral	-3.54E+00	Neutral	-5.39E+00	Neutral
73	p.Ala73Glu			7289	687	5.03	4.60	13173	7029	4.88	5.03	-1.23E+00	Neutral	-2.58E+00	Neutral	-1.95E+00	Neutral
73	p.Ala73Ala	Synonymous		9811	999	6.77	6.70	18989	9945	7.04	7.12	-1.06E+00	Neutral	-1.06E+00	Neutral	-8.18E-01	Neutral
73	p.Ala73Gly			7551	854	5.21	5.72	15359	8099	5.70	5.80	-3.41E+00	Neutral	-1.78E+00	Neutral	-2.99E+00	Neutral
73	p.Ala73Val			7585	867	5.24	5.81	16194	7473	6.01	5.35	-3.57E+00	Neutral	-6.00E-01	Neutral	-2.21E+00	Neutral
73	p.Ala73Tyr			8714	958	6.02	6.42	14375	8705	5.33	6.23	-2.25E+00	Neutral	-4.27E+00	Neutral	-4.06E+00	Neutral
73	p.Ala73Cys			7123	790	4.92	5.30	13784	7017	5.11	5.02	-3.40E+00	Neutral	-1.79E+00	Neutral	-2.99E+00	Neutral
73	p.Ala73Trp			5446	663	3.76	4.44	11835	6004	4.39	4.30	-7.72E+00	Indeterminate	-2.37E+00	Neutral	-7.09E+00	Indeterminate
73	p.Ala73Phe			5632	502	3.89	3.36	10777	5759	4.00	4.12	-1.51E+00	Neutral	-3.70E+00	Neutral	-3.00E+00	Neutral
74	p.Asp74Asn			1709	639	4.77	1.32	1637	440	4.33	2.22	-1.61E+01	Indeterminate	-2.63E+01	Indeterminate	-5.32E+01	Deleterious
74	p.Asp74Lys			2080	5773	5.81	11.95	2112	2212	5.59	11.17	-5.32E+01	Deleterious	-5.32E+01	Deleterious	-5.32E+01	Deleterious
74	p.Asp74Thr			1807	2120	5.05	4.39	2075	1153	5.49	5.82	-5.32E+01	Deleterious	-5.32E+01	Deleterious	-5.32E+01	Deleterious
74	p.Asp74Arg			1651	4359	4.61	9.02	1825	1757	4.83	8.87	-5.32E+01	Deleterious	-5.32E+01	Deleterious	-5.32E+01	Deleterious
74	p.Asp74Ser			1832	475	5.12	0.98	1970	439	5.21	2.22	-3.50E+00	Neutral	-1.26E+01	Indeterminate	-1.25E+01	Indeterminate
74	p.Asp74Ile			1665	5131	4.65	10.62	1904	1506	5.04	7.60	-5.32E+01	Deleterious	-5.32E+01	Deleterious	-5.32E+01	Deleterious
74	p.Asp74Met			2121	2922	5.92	6.05	2291	1390	6.06	7.02	-5.32E+01	Deleterious	-5.32E+01	Deleterious	-5.32E+01	Deleterious
74	p.Asp74His		Neutral	1436	376	4.01	0.78	1554	225	4.11	1.14	-5.38E+00	Neutral	-2.84E+00	Neutral	-5.47E+00	Neutral
74	p.Asp74Gln			1572	2339	4.39	4.84	1722	962	4.56	4.86	-5.32E+01	Deleterious	-5.32E+01	Deleterious	-5.32E+01	Deleterious
74	p.Asp74Pro			1306	3867	3.65	8.00	1439	1350	3.81	6.82	-5.32E+01	Deleterious	-5.32E+01	Deleterious	-5.32E+01	Deleterious
74	p.Asp74Leu			2179	3931	6.09	8.14	2204	1598	5.83	8.07	-5.32E+01	Deleterious	-5.32E+01	Deleterious	-5.32E+01	Deleterious
74	p.Asp74Asp	Synonymous		2293	526	6.40	1.09	2368	337	6.26	1.70	-1.06E+00	Neutral	-1.06E+00	Neutral	-8.18E-01	Neutral
74	p.Asp74Glu			1910	219	5.33	0.45	1855	108	4.91	0.55	-2.35E-04	Neutral	-4.72E-06	Neutral	-1.99E-08	Neutral
74	p.Asp74Ala		Deleterious	1340	933	3.74	1.93	1438	505	3.80	2.55	-5.32E+01	Deleterious	-5.32E+01	Deleterious	-5.32E+01	Deleterious
74	p.Asp74Gly			1450	722	4.05	1.49	1509	400	3.99	2.02	-3.98E+01	Indeterminate	-2.72E+01	Indeterminate	-5.32E+01	Deleterious
74	p.Asp74Val			2034	4350	5.68	9.00	2153	1594	5.70	8.05	-5.32E+01	Deleterious	-5.32E+01	Deleterious	-5.32E+01	Deleterious
74	p.Asp74Tyr		Deleterious	1872	2840	5.23	5.88	1987	1081	5.26	5.46	-5.32E+01	Deleterious	-5.32E+01	Deleterious	-5.32E+01	Deleterious
74	p.Asp74Cys			1766	266	4.93	0.55	1743	175	4.61	0.88	-3.63E-02	Neutral	-1.08E-01	Neutral	-6.79E-03	Neutral
74	p.Asp74Trp			2243	3647	6.26	7.55	2314	1334	6.12	6.73	-5.32E+01	Deleterious	-5.32E+01	Deleterious	-5.32E+01	Deleterious
74	p.Asp74Phe			1538	2881	4.30	5.96	1700	1243	4.50	6.27	-5.32E+01	Deleterious	-5.32E+01	Deleterious	-5.32E+01	Deleterious
75	p.Pro75Asn			9670	524	4.87	4.68	11723	3674	4.98	5.59	-1.84E+00	Neutral	-2.33E+00	Neutral	-2.21E+00	Neutral
75	p.Pro75Lys			9337	609	4.70	5.43	11391	3490	4.84	5.31	-5.18E+00	Neutral	-2.16E+00	Neutral	-4.73E+00	Neutral
75	p.Pro75Thr			11192	685	5.64	6.11	12660	3467	5.38	5.27	-2.81E+00	Neutral	-7.98E-01	Neutral	-1.80E+00	Neutral
75	p.Pro75Arg			9880	459	4.98	4.10	10942	3084	4.65	4.69	-5.75E-01	Neutral	-1.39E+00	Neutral	-7.26E-01	Neutral
75	p.Pro75Ser			10276	621	5.18	5.54	11437	2762	4.86	4.20	-3.06E+00	Neutral	-3.66E-01	Neutral	-1.67E+00	Neutral
75	p.Pro75Ile			9905	491	4.99	4.38	11194	3667	4.76	5.57	-9.52E-01	Neutral	-3.23E+00	Neutral	-2.22E+00	Neutral
75	p.Pro75Met			9184	602	4.63	5.37	11731	3461	4.99	5.26	-5.43E+00	Neutral	-1.61E+00	Neutral	-4.48E+00	Neutral
75	p.Pro75His			9528	472	4.80	4.21	10826	2310	4.60	3.51	-1.04E+00	Neutral	-1.23E-01	Neutral	-3.11E-01	Neutral
75	p.Pro75Gln			8492	532	4.28	4.75	11101	3043	4.72	4.63	-4.98E+00	Neutral	-1.11E+00	Neutral	-3.70E+00	Neutral
75	p.Pro75Pro	Synonymous		11272	592	5.68	5.28	11825	3289	5.03	5.00	-1.06E+00	Neutral	-1.06E+00	Neutral	-8.18E-01	Neutral
75	p.Pro75Leu			8173	440	4.12	3.93	10218	2734	4.34	4.16	-2.46E+00	Neutral	-1.14E+00	Neutral	-1.79E+00	Neutral
75	p.Pro75Asp			7922	428	3.99	3.82	9264	2396	3.94	3.64	-2.66E+00	Neutral	-1.13E+00	Neutral	-1.93E+00	Neutral
75	p.Pro75Glu			10198	576	5.14	5.14	12698	3162	5.40	4.81	-2.12E+00	Neutral	-3.52E-01	Neutral	-1.03E+00	Neutral
75	p.Pro75Ala			10907	615	5.49	5.49	13939	3795	5.93	5.77	-1.83E+00	Neutral	-5.89E-01	Neutral	-1.00E+00	Neutral
75	p.Pro75Gly			8363	444	4.21	3.96	10388	2646	4.42	4.02	-2.17E+00	Neutral	-7.57E-01	Neutral	-1.33E+00	Neutral
75	p.Pro75Val			11370	636	5.73	5.67	14557	4466	6.19	6.79	-1.60E+00	Neutral	-1.30E+00	Neutral	-1.31E+00	Neutral
75	p.Pro75Tyr			11247	621	5.66	5.54	12810	3790	5.45	5.76	-1.50E+00	Neutral	-1.36E+00	Neutral	-1.28E+00	Neutral
75	p.Pro75Cys			9823	570	4.95	5.09	11358	2913	4.83	4.43	-2.66E+00	Neutral	-6.31E-01	Neutral	-1.58E+00	Neutral
75	p.Pro75Trp			11015	691	5.55	6.17	13000	4054	5.53	6.16	-3.29E+00	Neutral	-1.85E+00	Neutral	-2.95E+00	Neutral
75	p.Pro75Phe			10783	600	5.43	5.35	12116	3578	5.15	5.44	-1.73E+00	Neutral	-1.51E+00	Neutral	-1.54E+00	Neutral
76	p.Ala76Asn			1971	8478	4.41	5.28					-1.34E+01	Indeterminate			-1.34E+01	Indeterminate
76	p.Ala76Lys			1935	7710	4.33	4.80					-1.05E+01	Indeterminate			-1.05E+01	Indeterminate
76	p.Ala76Thr			2338	7340	5.23	4.57					-2.83E+00	Neutral			-2.83E+00	Neutral
76	p.Ala76Arg			1828	7490	4.09	4.66					-1.24E+01	Indeterminate			-1.24E+01	Indeterminate
76	p.Ala76Ser			2564	10119	5.73	6.30					-7.14E+00	Indeterminate			-7.14E+00	Indeterminate
76	p.Ala76Ile			1198	4992	2.68	3.11					-2.02E+01	Indeterminate			-2.02E+01	Indeterminate
76	p.Ala76Met			2897	9059	6.48	5.64					-1.83E+00	Neutral			-1.83E+00	Neutral
76	p.Ala76His			2955	11104	6.61	6.91					-4.71E+00	Neutral			-4.71E+00	Neutral
76	p.Ala76Gln			1360	5492	3.04	3.42					-1.62E+01	Indeterminate			-1.62E+01	Indeterminate
76	p.Ala76Pro			2850	9900	6.37	6.16					-3.41E+00	Neutral			-3.41E+00	Neutral
76	p.Ala76Leu			1821	6818	4.07	4.24					-9.05E+00	Indeterminate			-9.05E+00	Indeterminate
76	p.Ala76Asp			2380	8354	5.32	5.20					-4.79E+00	Neutral			-4.79E+00	Neutral
76	p.Ala76Glu			2284	9100	5.11	5.66					-8.61E+00	Indeterminate			-8.61E+00	Indeterminate
76	p.Ala76Ala	Synonymous		1395	3155	3.12	1.96					-1.06E+00	Neutral			-1.06E+00	Neutral
76	p.Ala76Gly			2810	9192	6.28	5.72					-2.53E+00	Neutral			-2.53E+00	Neutral
76	p.Ala76Val			1949	6091	4.36	3.79					-3.78E+00	Neutral			-3.78E+00	Neutral
76	p.Ala76Tyr			2465	8009	5.51	4.99					-3.09E+00	Neutral			-3.09E+00	Neutral
76	p.Ala76Cys			2123	9589	4.75	5.97					-1.45E+01	Indeterminate			-1.45E+01	Indeterminate
76	p.Ala76Trp			3462	10823	7.74	6.74					-1.24E+00	Neutral			-1.24E+00	Neutral
76	p.Ala76Phe			2136	7846	4.78	4.88					-6.82E+00	Indeterminate			-6.82E+00	Indeterminate
77	p.Thr77Asn			10090	1639	5.69	5.55					-4.52E+00	Neutral			-4.52E+00	Neutral
77	p.Thr77Lys			12148	2087	6.85	7.06					-4.40E+00	Neutral			-4.40E+00	Neutral
77	p.Thr77Thr	Synonymous		6814	757	3.84	2.56					-1.06E+00	Neutral			-1.06E+00	Neutral

77	p.Thr77Arg			7484	1416	4.22	4.79	-1.23E+01	Indeterminate	-1.23E+01	Indeterminate
77	p.Thr77Ser			8715	1463	4.91	4.95	-6.48E+00	Indeterminate	-6.48E+00	Indeterminate
77	p.Thr77Ile			9404	1326	5.30	4.49	-2.46E+00	Neutral	-2.46E+00	Neutral
77	p.Thr77Met			5988	879	3.37	2.97	-6.23E+00	Indeterminate	-6.23E+00	Indeterminate
77	p.Thr77His			7330	1092	4.13	3.69	-4.95E+00	Neutral	-4.95E+00	Neutral
77	p.Thr77Gln			8278	1295	4.66	4.38	-5.14E+00	Neutral	-5.14E+00	Neutral
77	p.Thr77Pro		Deleterious	7110	2118	4.01	7.17	-4.41E+01	Indeterminate	-3.32E+01	Indeterminate
77	p.Thr77Leu			11158	1918	6.29	6.49	-5.03E+00	Neutral	-5.03E+00	Neutral
77	p.Thr77Asp			4915	701	2.77	2.37	-7.25E+00	Indeterminate	-7.25E+00	Indeterminate
77	p.Thr77Glu			9748	2211	5.49	7.48	-1.66E+01	Indeterminate	-1.66E+01	Indeterminate
77	p.Thr77Ala			9298	1602	5.24	5.42	-6.61E+00	Indeterminate	-6.61E+00	Indeterminate
77	p.Thr77Gly			12368	1745	6.97	5.90	-1.41E+00	Neutral	-1.41E+00	Neutral
77	p.Thr77Val			11478	2244	6.47	7.59	-8.27E+00	Indeterminate	-8.27E+00	Indeterminate
77	p.Thr77Tyr			9931	1465	5.60	4.96	-2.86E+00	Neutral	-2.86E+00	Neutral
77	p.Thr77Cys			13304	2046	7.50	6.92	-2.08E+00	Neutral	-2.08E+00	Neutral
77	p.Thr77Trp			6374	853	3.59	2.89	-3.70E+00	Neutral	-3.70E+00	Neutral
77	p.Thr77Phe			5533	701	3.12	2.37	-3.56E+00	Neutral	-3.56E+00	Neutral
78	p.Leu78Asn			2675	1771	7.71	6.34	-4.47E-03	Neutral	-4.47E-03	Neutral
78	p.Leu78Lys			1995	1493	5.75	5.34	-1.01E-01	Neutral	-1.01E-01	Neutral
78	p.Leu78Thr			1668	1264	4.81	4.52	-2.23E-01	Neutral	-2.23E-01	Neutral
78	p.Leu78Arg			943	1294	2.72	4.63	-1.69E+01	Indeterminate	-1.69E+01	Indeterminate
78	p.Leu78Ser			1338	660	3.86	2.36	-1.46E-03	Neutral	-1.46E-03	Neutral
78	p.Leu78Ile			2099	1782	6.05	6.38	-3.23E-01	Neutral	-3.23E-01	Neutral
78	p.Leu78Met			1020	1289	2.94	4.61	-1.20E+01	Indeterminate	-1.20E+01	Indeterminate
78	p.Leu78His			1588	1438	4.58	5.15	-1.17E+00	Neutral	-1.17E+00	Neutral
78	p.Leu78Gln			1438	1676	4.14	6.00	-5.66E+00	Neutral	-5.66E+00	Neutral
78	p.Leu78Pro			1788	2655	5.15	9.50	-1.10E+01	Indeterminate	-1.10E+01	Indeterminate
78	p.Leu78Leu	Synonymous		1779	1650	5.13	5.90	-1.06E+00	Neutral	-1.06E+00	Neutral
78	p.Leu78Asp			5455	2299	15.72	8.23	-5.61E-10	Neutral	-4.16E-10	Neutral
78	p.Leu78Glu			1427	1642	4.11	5.88	-5.41E+00	Neutral	-5.41E+00	Neutral
78	p.Leu78Ala			637	727	1.84	2.60	-1.43E+01	Indeterminate	-1.43E+01	Indeterminate
78	p.Leu78Gly			1290	219	3.72	0.78	-1.12E-15	Neutral	0.00E+00	Neutral
78	p.Leu78Val			2096	1560	6.04	5.58	-7.80E-02	Neutral	-7.80E-02	Neutral
78	p.Leu78Tyr			1478	1553	4.26	5.56	-3.31E+00	Neutral	-3.31E+00	Neutral
78	p.Leu78Cys			1343	1282	3.87	4.59	-2.33E+00	Neutral	-2.33E+00	Neutral
78	p.Leu78Trp			1194	791	3.44	2.83	-1.75E-01	Neutral	-1.75E-01	Neutral
78	p.Leu78Phe			1456	900	4.20	3.22	-3.38E-02	Neutral	-3.38E-02	Neutral
79	p.Thr79Asn			6357	2367	6.19	4.94	-3.23E-01	Neutral	-3.23E-01	Neutral
79	p.Thr79Lys			4790	2080	4.67	4.34	-2.10E+00	Neutral	-2.10E+00	Neutral
79	p.Thr79Thr	Synonymous		7615	3475	7.42	7.25	-1.06E+00	Neutral	-1.06E+00	Neutral
79	p.Thr79Arg			3106	1753	3.03	3.66	-1.26E+01	Indeterminate	-1.26E+01	Indeterminate
79	p.Thr79Ser			5296	2013	5.16	4.20	-6.60E-01	Neutral	-6.60E-01	Neutral
79	p.Thr79Ile			5523	1971	5.38	4.11	-3.36E-01	Neutral	-3.36E-01	Neutral
79	p.Thr79Met			6304	2761	6.14	5.76	-1.24E+00	Neutral	-1.24E+00	Neutral
79	p.Thr79His			5309	2284	5.17	4.77	-1.61E+00	Neutral	-1.61E+00	Neutral
79	p.Thr79Gln			4412	1628	4.30	3.40	-8.42E-01	Neutral	-8.42E-01	Neutral
79	p.Thr79Pro			3460	4139	3.37	8.64	-5.32E+01	Deleterious	-5.32E+01	Deleterious
79	p.Thr79Leu			4103	1982	4.00	4.14	-4.80E+00	Neutral	-4.80E+00	Neutral
79	p.Thr79Asp			3999	1769	3.90	3.69	-3.25E+00	Neutral	-3.25E+00	Neutral
79	p.Thr79Glu			3681	1420	3.59	2.96	-1.74E+00	Neutral	-1.74E+00	Neutral
79	p.Thr79Ala			5581	2518	5.44	5.25	-1.95E+00	Neutral	-1.95E+00	Neutral
79	p.Thr79Gly			5518	2596	5.38	5.42	-2.54E+00	Neutral	-2.54E+00	Neutral
79	p.Thr79Val			8644	4107	8.42	8.57	-1.05E+00	Neutral	-1.05E+00	Neutral
79	p.Thr79Tyr			3452	1556	3.36	3.25	-4.54E+00	Neutral	-4.54E+00	Neutral
79	p.Thr79Cys			5814	2910	5.66	6.07	-3.24E+00	Neutral	-3.24E+00	Neutral
79	p.Thr79Trp			4719	2447	4.60	5.11	-5.36E+00	Neutral	-5.36E+00	Neutral
79	p.Thr79Phe			4968	2150	4.84	4.49	-1.91E+00	Neutral	-1.91E+00	Neutral
80	p.Arg80Asn			9628	1551	4.97	5.34	-1.78E+01	Indeterminate	-1.78E+01	Indeterminate
80	p.Arg80Lys			11473	1355	5.92	4.67	-4.55E+00	Neutral	-4.55E+00	Neutral
80	p.Arg80Thr			5402	616	2.79	2.12	-1.09E+01	Indeterminate	-1.09E+01	Indeterminate
80	p.Arg80Arg	Synonymous		13295	1283	6.86	4.42	-1.06E+00	Neutral	-1.06E+00	Neutral
80	p.Arg80Ser			12287	1392	6.34	4.80	-3.31E+00	Neutral	-3.31E+00	Neutral
80	p.Arg80Ile			7889	1862	4.07	6.41	-5.32E+01	Deleterious	-5.32E+01	Deleterious
80	p.Arg80Met			8839	1240	4.56	4.27	-1.26E+01	Indeterminate	-1.26E+01	Indeterminate
80	p.Arg80His			9521	1203	4.92	4.14	-7.91E+00	Indeterminate	-7.91E+00	Indeterminate
80	p.Arg80Gln			7688	1142	3.97	3.93	-1.75E+01	Indeterminate	-1.75E+01	Indeterminate
80	p.Arg80Pro			8976	4035	4.63	13.90	-5.32E+01	Deleterious	-5.32E+01	Deleterious
80	p.Arg80Leu			10680	1517	5.51	5.23	-1.06E+01	Indeterminate	-1.06E+01	Indeterminate
80	p.Arg80Asp			10178	1582	5.26	5.45	-1.51E+01	Indeterminate	-1.51E+01	Indeterminate
80	p.Arg80Glu			9465	1058	4.89	3.64	-4.74E+00	Neutral	-4.74E+00	Neutral
80	p.Arg80Ala			11000	1400	5.68	4.82	-6.72E+00	Indeterminate	-6.72E+00	Indeterminate
80	p.Arg80Gly			10632	1370	5.49	4.72	-7.39E+00	Indeterminate	-7.39E+00	Indeterminate
80	p.Arg80Val			8051	1272	4.16	4.38	-2.01E+01	Indeterminate	-2.01E+01	Indeterminate
80	p.Arg80Tyr			11274	1590	5.82	5.48	-9.69E+00	Indeterminate	-9.69E+00	Indeterminate
80	p.Arg80Cys			6897	589	3.56	2.03	-2.00E+00	Neutral	-2.00E+00	Neutral
80	p.Arg80Trp			11399	1615	5.89	5.56	-9.72E+00	Indeterminate	-9.72E+00	Indeterminate
80	p.Arg80Phe			9095	1358	4.70	4.68	-1.50E+01	Indeterminate	-1.50E+01	Indeterminate
81	p.Pro81Asn			3270	2138	4.49	5.03	-5.32E+01	Deleterious	-5.32E+01	Deleterious
81	p.Pro81Lys			4056	3564	5.57	8.39	-5.32E+01	Deleterious	-5.32E+01	Deleterious
81	p.Pro81Thr		Deleterious	3644	1370	5.01	3.23	-4.95E+01	Indeterminate	-3.32E+01	Indeterminate
81	p.Pro81Arg			4210	2742	5.79	6.46	-5.32E+01	Deleterious	-5.32E+01	Deleterious
81	p.Pro81Ser			2453	1122	3.37	2.64	-5.32E+01	Deleterious	-5.32E+01	Deleterious
81	p.Pro81Ile			2461	1198	3.38	2.82	-5.32E+01	Deleterious	-5.32E+01	Deleterious
81	p.Pro81Met			4378	2646	6.02	6.23	-5.32E+01	Deleterious	-5.32E+01	Deleterious
81	p.Pro81His			3889	2773	5.35	6.53	-5.32E+01	Deleterious	-5.32E+01	Deleterious
81	p.Pro81Gln			4314	4646	5.93	10.94	-5.32E+01	Deleterious	-5.32E+01	Deleterious
81	p.Pro81Pro	Synonymous		3937	545	5.41	1.28	-1.06E+00	Neutral	-1.06E+00	Neutral
81	p.Pro81Leu		Deleterious	2688	1382	3.69	3.25	-5.32E+01	Deleterious	-5.32E+01	Deleterious
81	p.Pro81Asp			3627	2528	4.99	5.95	-5.32E+01	Deleterious	-5.32E+01	Deleterious
81	p.Pro81Glu			3955	3393	5.44	7.99	-5.32E+01	Deleterious	-5.32E+01	Deleterious
81	p.Pro81Ala			2546	590	3.50	1.39	-2.01E+01	Indeterminate	-2.01E+01	Indeterminate
81	p.Pro81Gly			3018	1629	4.15	3.84	-5.32E+01	Deleterious	-5.32E+01	Deleterious
81	p.Pro81Val			3803	1377	5.23	3.24	-4.42E+01	Indeterminate	-3.32E+01	Indeterminate
81	p.Pro81Tyr			3466	2061	4.76	4.85	-5.32E+01	Deleterious	-5.32E+01	Deleterious
81	p.Pro81Cys			5033	1452	6.92	3.42	-1.97E+01	Indeterminate	-1.97E+01	Indeterminate
81	p.Pro81Trp			4048	2847	5.56	6.70	-5.32E+01	Deleterious	-5.32E+01	Deleterious
81	p.Pro81Phe			3961	2474	5.44	5.82	-5.32E+01	Deleterious	-5.32E+01	Deleterious
82	p.Val82Asn			5118	927	3.84	4.94	-5.32E+01	Deleterious	-5.32E+01	Deleterious
82	p.Val82Lys			4194	977	3.15	5.20	-5.32E+01	Deleterious	-5.32E+01	Deleterious
82	p.Val82Thr			11017	1270	8.28	6.76	-8.29E+00	Indeterminate	-8.29E+00	Indeterminate
82	p.Val82Arg			8316	1978	6.25	10.53	-5.32E+01	Deleterious	-5.32E+01	Deleterious
82	p.Val82Ser			8583	897	6.45	4.78	-7.82E+00	Indeterminate	-7.82E+00	Indeterminate
82	p.Val82Ile			7271	717	5.46	3.82	-7.73E+00	Indeterminate	-7.73E+00	Indeterminate
82	p.Val82Met			5075	324	3.81	1.73	-1.59E+00	Neutral	-1.59E+00	Neutral
82	p.Val82His			8957	1689	6.73	9.00	-4.23E+01	Indeterminate	-3.32E+01	Indeterminate
82	p.Val82Gln			6300	686	4.73	3.65	-1.31E+01	Indeterminate	-1.31E+01	Indeterminate
82	p.Val82Pro			7701	1207	5.78	6.43	-3.07E+01	Indeterminate	-3.05E+01	Indeterminate
82	p.Val82Leu			6686	586	5.02	3.12	-5.30E+00	Neutral	-5.30E+00	Neutral
82	p.Val82Asp			3241	694	2.43	3.70	-5.32E+01	Deleterious	-5.32E+01	Deleterious
82	p.Val82Glu			6491	1072	4.88	5.71	-3.99E+01	Indeterminate	-3.32E+01	Indeterminate
82	p.Val82Ala			8058	625	6.05	3.33	-2.05E+00	Neutral	-2.05E+00	Neutral
82	p.Val82Gly			4572	717	3.43	3.82	-4.61E+01	Indeterminate	-3.32E+01	Indeterminate
82	p.Val82Val	Synonymous		10662	821	8.01	4.37	-1.06E+00	Neutral	-1.06E+00	Neutral
82	p.Val82Tyr			3733	830	2.80	4.42	-5.32E+01	Deleterious	-5.32E+01	Deleterious
82	p.Val82Cys			4968	509	3.73	2.71	-1.39E+01	Indeterminate	-1.39E+01	Indeterminate
82	p.Val82Trp			4187	1143	3.14	6.09	-5.32E+01	Deleterious	-5.32E+01	Deleterious
82	p.Val82Phe			8005	1107	6.01	5.90	-2.13E+01	Indeterminate	-2.13E+01	Indeterminate
83	p.His83Asn			2209	1716	5.20	5.30	-5.32E+01	Deleterious	-5.32E+01	Deleterious
83	p.His83Lys			2282	2477	5.37	7.65	-5.32E+01	Deleterious	-5.32E+01	Deleterious
83	p.His83Thr			1704	1097	4.01	3.39	-5.32E+01	Deleterious	-5.32E+01	Deleterious
83	p.His83Arg	Pathogenic		2229	2555	5.25	7.89	-5.32E+01	Deleterious	-5.32E+01	Deleterious
83	p.His83Ser			2121	1323	4.99	4.09	-5.32E+01	Deleterious	-5.32E+01	Deleterious
83	p.His83Ile			2239	1501	5.27	4.64	-5.32E+01	Deleterious	-5.32E+01	Deleterious
83	p.His83Met			1978	448	4.66	1.38	-3.07E+01	Indeterminate	-3.05E+01	Indeterminate
83	p.His83His	Synonymous		2145	220	5.05	0.68	-1.06E+00	Neutral	-1.06E+00	Neutral
83	p.His83Gln			2092	858	4.92	2.65	-5.32E+01	Deleterious	-5.32E+01	Deleterious
83	p.His83Pro			2039	2411	4.80	7.45	-5.32E+01	Deleterious	-5.32E+01	Deleterious
83	p.His83Leu			2061	1244	4.85	3.84	-5.32E+01	Deleterious	-5.32E+01	Deleterious
83	p.His83Asp	Likely pathogenic		1956	2148	4.60	6.63	-5.32E+01	Deleterious	-5.32E+01	Deleterious
83	p.His83Glu			2438	2516	5.74	7.77	-5.32E+01	Deleterious	-5.32E+01	Deleterious
83	p.His83Ala			2190	589	5.15	1.82	-4.26E+01	Indeterminate	-3.32E+01	Indeterminate
83	p.His83Gly			2738	2060	6.44	6.36	-5.32E+01	Deleterious	-5.32E+01	Deleterious
83	p.His83Val			1653	1644	3.89	5.08	-5.32E+01	Deleterious	-5.32E+01	Deleterious
83	p.His83Tyr	Likely pathogenic		1726	1905	4.06	5.88	-5.32E+01	Deleterious	-5.32E+01	Deleterious
83	p.His83Cys			1753	1232	4.13	3.80	-5.32E+01	Deleterious	-5.32E+01	Deleterious
83	p.His83Trp			2279	2636	5.36	8.14	-5.32E+01	Deleterious	-5.32E+01	Deleterious
83	p.His83Phe			2658	1802	6.26	5.56	-5.32E+01	Deleterious	-5.32E+01	Deleterious
84	p.Asp84Asn	Likely pathogenic		2322	945	3.70	3.51	-5.32E+01	Deleterious	-5.32E+01	Deleterious
84	p.Asp84Lys			3482	2031	5.55	7.54	-5.32E+01	Deleterious	-5.32E+01	Deleterious

84	p.Asp84Thr			2464	1033	3.92	3.83	-5.32E+01	Deleterious	-5.32E+01	Deleterious
84	p.Asp84Arg			3099	1815	4.94	6.73	-5.32E+01	Deleterious	-5.32E+01	Deleterious
84	p.Asp84Ser			2805	443	4.47	1.64	-5.32E+01	Deleterious	-5.32E+01	Deleterious
84	p.Asp84Ile			3566	2122	5.68	7.87	-5.32E+01	Deleterious	-5.32E+01	Deleterious
84	p.Asp84Met			2901	1475	4.62	5.47	-5.32E+01	Deleterious	-5.32E+01	Deleterious
84	p.Asp84His		Deleterious	3544	821	5.64	3.05	-5.32E+01	Deleterious	-5.32E+01	Deleterious
84	p.Asp84Gln			2535	744	4.04	2.76	-5.32E+01	Deleterious	-5.32E+01	Deleterious
84	p.Asp84Pro			3263	1893	5.20	7.02	-5.32E+01	Deleterious	-5.32E+01	Deleterious
84	p.Asp84Leu			3617	2184	5.76	8.10	-5.32E+01	Deleterious	-5.32E+01	Deleterious
84	p.Asp84Asp	Synonymous		3069	79	4.89	0.29	-1.06E+00	Neutral	-1.06E+00	Neutral
84	p.Asp84Glu			2431	94	3.87	0.35	-1.16E+01	Indeterminate	-1.16E+01	Indeterminate
84	p.Asp84Ala		Deleterious	4167	1843	6.64	6.84	-5.32E+01	Deleterious	-5.32E+01	Deleterious
84	p.Asp84Gly		Deleterious	2603	1289	4.15	4.78	-5.32E+01	Deleterious	-5.32E+01	Deleterious
84	p.Asp84Val		Deleterious	2863	1540	4.56	5.71	-5.32E+01	Deleterious	-5.32E+01	Deleterious
84	p.Asp84Tyr		Deleterious	3406	1751	5.42	6.50	-5.32E+01	Deleterious	-5.32E+01	Deleterious
84	p.Asp84Cys			3373	512	5.37	1.90	-5.32E+01	Deleterious	-5.32E+01	Deleterious
84	p.Asp84Trp			3522	2243	5.61	8.32	-5.32E+01	Deleterious	-5.32E+01	Deleterious
84	p.Asp84Phe			3758	2096	5.99	7.78	-5.32E+01	Deleterious	-5.32E+01	Deleterious
85	p.Ala85Asn			5212	1526	2.82	2.47	-1.83E+01	Indeterminate	-1.83E+01	Indeterminate
85	p.Ala85Lys			9383	4778	5.07	7.73	-4.54E+01	Indeterminate	-3.32E+01	Indeterminate
85	p.Ala85Thr			7427	2426	4.01	3.92	-1.78E+01	Indeterminate	-1.78E+01	Indeterminate
85	p.Ala85Arg			8838	4487	4.78	7.26	-4.72E+01	Indeterminate	-3.32E+01	Indeterminate
85	p.Ala85Ser			10574	2582	5.72	4.18	-3.75E+00	Neutral	-3.75E+00	Neutral
85	p.Ala85Ile			11678	4130	6.31	6.68	-1.43E+01	Indeterminate	-1.43E+01	Indeterminate
85	p.Ala85Met			6476	1133	3.50	1.83	-1.40E+00	Neutral	-1.40E+00	Neutral
85	p.Ala85His			7202	2718	3.89	4.40	-2.74E+01	Indeterminate	-2.74E+01	Indeterminate
85	p.Ala85Gln			8493	2892	4.59	4.68	-1.77E+01	Indeterminate	-1.77E+01	Indeterminate
85	p.Ala85Pro			10997	3768	5.94	6.09	-1.38E+01	Indeterminate	-1.38E+01	Indeterminate
85	p.Ala85Leu			10857	2524	5.87	4.08	-2.77E+00	Neutral	-2.77E+00	Neutral
85	p.Ala85Asp			7167	3294	3.87	5.33	-4.41E+01	Indeterminate	-3.32E+01	Indeterminate
85	p.Ala85Glu			7645	3405	4.13	5.51	-3.91E+01	Indeterminate	-3.32E+01	Indeterminate
85	p.Ala85Ala	Synonymous		15969	3621	8.63	5.86	-1.06E+00	Neutral	-1.06E+00	Neutral
85	p.Ala85Gly			13936	2718	7.53	4.40	-4.57E-01	Neutral	-4.57E-01	Neutral
85	p.Ala85Val			10776	3252	5.82	5.26	-9.08E+00	Indeterminate	-9.08E+00	Indeterminate
85	p.Ala85Tyr			6635	2743	3.59	4.44	-3.67E+01	Indeterminate	-3.31E+01	Indeterminate
85	p.Ala85Cys			9406	1933	5.08	3.13	-1.78E+00	Neutral	-1.78E+00	Neutral
85	p.Ala85Trp			6098	3377	3.30	5.46	-5.32E+01	Deleterious	-5.32E+01	Deleterious
85	p.Ala85Phe			10242	4529	5.54	7.32	-3.04E+01	Indeterminate	-3.02E+01	Indeterminate
86	p.Ala86Asn			2738	7263	5.42	6.88	-5.32E+01	Deleterious	-5.32E+01	Deleterious
86	p.Ala86Lys			1712	5339	3.39	5.06	-5.32E+01	Deleterious	-5.32E+01	Deleterious
86	p.Ala86Thr			1173	286	2.32	0.27	-6.25E-02	Neutral	-6.25E-02	Neutral
86	p.Ala86Arg			2701	8049	5.35	7.62	-5.32E+01	Deleterious	-5.32E+01	Deleterious
86	p.Ala86Ser			1742	753	3.45	0.71	-2.71E+00	Neutral	-2.71E+00	Neutral
86	p.Ala86Ile			2571	1478	5.09	1.40	-5.80E+00	Neutral	-5.80E+00	Indeterminate
86	p.Ala86Met			2759	6416	5.46	6.08	-5.32E+01	Deleterious	-5.32E+01	Deleterious
86	p.Ala86His			3907	10251	7.74	9.71	-5.32E+01	Deleterious	-5.32E+01	Deleterious
86	p.Ala86Gln			3641	10410	7.21	9.86	-5.32E+01	Deleterious	-5.32E+01	Deleterious
86	p.Ala86Pro			3199	8498	6.34	8.05	-5.32E+01	Deleterious	-5.32E+01	Deleterious
86	p.Ala86Leu			3102	7242	6.14	6.86	-5.32E+01	Deleterious	-5.32E+01	Deleterious
86	p.Ala86Asp			2882	7986	5.71	7.56	-5.32E+01	Deleterious	-5.32E+01	Deleterious
86	p.Ala86Glu			3145	8838	6.23	8.37	-5.32E+01	Deleterious	-5.32E+01	Deleterious
86	p.Ala86Ala	Synonymous		986	304	1.95	0.29	-1.06E+00	Neutral	-1.06E+00	Neutral
86	p.Ala86Gly			1964	522	3.89	0.49	-1.98E-02	Neutral	-1.98E-02	Neutral
86	p.Ala86Val			2113	649	4.18	0.61	-9.64E-02	Neutral	-9.64E-02	Neutral
86	p.Ala86Tyr			2032	5898	4.02	5.59	-5.32E+01	Deleterious	-5.32E+01	Deleterious
86	p.Ala86Cys			2481	507	4.91	0.48	-4.00E-05	Neutral	-4.00E-05	Neutral
86	p.Ala86Trp			2661	6920	5.27	6.55	-5.32E+01	Deleterious	-5.32E+01	Deleterious
86	p.Ala86Phe			2986	7970	5.91	7.55	-5.32E+01	Deleterious	-5.32E+01	Deleterious
87	p.Arg87Asn			7612	236	5.72	1.26	-3.51E+00	Neutral	-3.51E+00	Neutral
87	p.Arg87Lys			3377	37	2.54	0.20	-7.53E-04	Neutral	-7.53E-04	Neutral
87	p.Arg87Thr			4254	127	3.20	0.68	-7.09E+00	Indeterminate	-7.09E+00	Indeterminate
87	p.Arg87Arg	Synonymous		5869	137	5.16	1.03	-1.06E+00	Neutral	-1.06E+00	Neutral
87	p.Arg87Ser			3450	208	2.59	1.11	-5.32E+01	Deleterious	-5.32E+01	Deleterious
87	p.Arg87Ile			4046	129	3.04	0.69	-9.73E+00	Indeterminate	-9.73E+00	Indeterminate
87	p.Arg87Met			7168	345	5.38	1.84	-1.99E+01	Indeterminate	-1.99E+01	Indeterminate
87	p.Arg87His			4551	247	3.42	1.32	-3.99E+01	Indeterminate	-3.32E+01	Indeterminate
87	p.Arg87Gln			7646	452	5.74	2.41	-3.25E+01	Indeterminate	-3.18E+01	Indeterminate
87	p.Arg87Pro	Pathogenic		3416	3365	2.57	17.92	-5.32E+01	Deleterious	-5.32E+01	Deleterious
87	p.Arg87Leu			4276	160	3.21	0.85	-1.57E+01	Indeterminate	-1.57E+01	Indeterminate
87	p.Arg87Asp			4569	777	3.43	4.14	-5.32E+01	Deleterious	-5.32E+01	Deleterious
87	p.Arg87Glu			6786	626	5.10	3.33	-5.32E+01	Deleterious	-5.32E+01	Deleterious
87	p.Arg87Ala			7639	562	5.74	2.99	-5.30E+01	Indeterminate	-3.32E+01	Indeterminate
87	p.Arg87Gly			5541	127	4.16	0.68	-1.07E+00	Neutral	-1.07E+00	Neutral
87	p.Arg87Val			6100	198	4.58	1.05	-6.11E+00	Indeterminate	-6.11E+00	Indeterminate
87	p.Arg87Tyr			2406	123	1.81	0.66	-5.32E+01	Deleterious	-5.32E+01	Deleterious
87	p.Arg87Cys			8971	188	6.74	1.00	-1.06E-01	Neutral	-1.06E-01	Neutral
87	p.Arg87Trp	Likely pathogenic		8616	4952	6.47	26.37	-5.32E+01	Deleterious	-5.32E+01	Deleterious
87	p.Arg87Phe			7490	267	5.63	1.42	-6.79E+00	Indeterminate	-6.79E+00	Indeterminate
88	p.Glu88Asn			7680	5284	4.42	4.15	-8.96E+00	Indeterminate	-8.96E+00	Indeterminate
88	p.Glu88Lys		Deleterious	9106	9400	5.25	7.39	-2.66E+01	Indeterminate	-2.65E+01	Indeterminate
88	p.Glu88Thr			5283	3622	3.04	2.85	-1.37E+01	Indeterminate	-1.37E+01	Indeterminate
88	p.Glu88Arg			9435	9436	5.44	7.42	-2.37E+01	Indeterminate	-2.37E+01	Indeterminate
88	p.Glu88Ser			6793	4359	3.91	3.43	-8.02E+00	Indeterminate	-8.02E+00	Indeterminate
88	p.Glu88Ile			7738	4822	4.46	3.79	-5.97E+00	Indeterminate	-5.97E+00	Indeterminate
88	p.Glu88Met			9862	6110	5.68	4.80	-4.02E+00	Neutral	-4.02E+00	Neutral
88	p.Glu88His			6938	5151	4.00	4.05	-1.32E+01	Indeterminate	-1.32E+01	Indeterminate
88	p.Glu88Gln			7089	4779	4.08	3.76	-9.17E+00	Indeterminate	-9.17E+00	Indeterminate
88	p.Glu88Pro			6230	10321	3.59	8.11	-5.32E+01	Deleterious	-5.32E+01	Deleterious
88	p.Glu88Leu			7368	5329	4.24	4.19	-1.13E+01	Indeterminate	-1.13E+01	Indeterminate
88	p.Glu88Asp			8787	6308	5.06	4.96	-8.89E+00	Indeterminate	-8.89E+00	Indeterminate
88	p.Glu88Glu	Synonymous		12109	6412	6.98	5.04	-1.06E+00	Neutral	-1.06E+00	Neutral
88	p.Glu88Ala			6884	3920	3.97	3.08	-4.75E+00	Neutral	-4.75E+00	Neutral
88	p.Glu88Gly			8821	5602	5.08	4.40	-5.36E+00	Neutral	-5.36E+00	Neutral
88	p.Glu88Val			16505	11466	9.51	9.01	-3.02E+00	Neutral	-3.02E+00	Neutral
88	p.Glu88Tyr			9528	6744	5.49	5.30	-7.59E+00	Indeterminate	-7.59E+00	Indeterminate
88	p.Glu88Cys			8363	5109	4.82	4.02	-4.88E+00	Neutral	-4.88E+00	Neutral
88	p.Glu88Trp			10256	6510	5.91	5.12	-4.25E+00	Neutral	-4.25E+00	Neutral
88	p.Glu88Phe			8799	6563	5.07	5.16	-1.02E+01	Indeterminate	-1.02E+01	Indeterminate
89	p.Gly89Asn			2627	478	3.60	0.67	-1.70E+01	Indeterminate	-1.70E+01	Indeterminate
89	p.Gly89Lys			4247	3480	5.82	4.87	-5.32E+01	Deleterious	-5.32E+01	Deleterious
89	p.Gly89Thr			3807	5129	5.22	7.18	-5.32E+01	Deleterious	-5.32E+01	Deleterious
89	p.Gly89Arg			3355	3087	4.60	4.32	-5.32E+01	Deleterious	-5.32E+01	Deleterious
89	p.Gly89Ser			3824	1590	5.24	2.23	-5.32E+01	Deleterious	-5.32E+01	Deleterious
89	p.Gly89Ile			3356	5608	4.60	7.85	-5.32E+01	Deleterious	-5.32E+01	Deleterious
89	p.Gly89Met			3081	3692	4.22	5.17	-5.32E+01	Deleterious	-5.32E+01	Deleterious
89	p.Gly89His			3103	3096	4.25	4.33	-5.32E+01	Deleterious	-5.32E+01	Deleterious
89	p.Gly89Gln			3330	2815	4.56	3.94	-5.32E+01	Deleterious	-5.32E+01	Deleterious
89	p.Gly89Pro			4288	4970	5.87	6.96	-5.32E+01	Deleterious	-5.32E+01	Deleterious
89	p.Gly89Leu			3791	5275	5.19	7.38	-5.32E+01	Deleterious	-5.32E+01	Deleterious
89	p.Gly89Asp		Deleterious	3504	1266	4.80	1.77	-5.32E+01	Deleterious	-5.32E+01	Deleterious
89	p.Gly89Glu			4701	4959	6.44	6.94	-5.32E+01	Deleterious	-5.32E+01	Deleterious
89	p.Gly89Ala			2968	631	4.07	0.88	-2.36E+01	Indeterminate	-2.36E+01	Indeterminate
89	p.Gly89Gly	Synonymous		2868	294	3.93	0.41	-1.06E+00	Neutral	-1.06E+00	Neutral
89	p.Gly89Val			3667	5622	5.02	7.87	-5.32E+01	Deleterious	-5.32E+01	Deleterious
89	p.Gly89Tyr			3624	5208	4.96	7.29	-5.32E+01	Deleterious	-5.32E+01	Deleterious
89	p.Gly89Cys			4087	1699	5.60	2.38	-5.32E+01	Deleterious	-5.32E+01	Deleterious
89	p.Gly89Trp			4212	6145	5.77	8.60	-5.32E+01	Deleterious	-5.32E+01	Deleterious
89	p.Gly89Phe			4558	6406	6.24	8.97	-5.32E+01	Deleterious	-5.32E+01	Deleterious
90	p.Phe90Asn			17215	3468	7.19	6.33	-7.65E-02	Neutral	-7.65E-02	Neutral
90	p.Phe90Lys			12298	3370	5.14	6.15	-2.63E+00	Neutral	-2.63E+00	Neutral
90	p.Phe90Thr			8757	2286	3.66	4.17	-3.69E+00	Neutral	-3.69E+00	Neutral
90	p.Phe90Arg			14675	3186	6.13	5.81	-3.08E-01	Neutral	-3.08E-01	Neutral
90	p.Phe90Ser			12195	2607	5.09	4.76	-4.70E-01	Neutral	-4.70E-01	Neutral
90	p.Phe90Ile			9858	1941	4.12	3.54	-4.24E-01	Neutral	-4.24E-01	Neutral
90	p.Phe90Met			9884	1676	4.13	3.06	-8.85E-02	Neutral	-8.85E-02	Neutral
90	p.Phe90His			9400	1661	3.93	3.03	-1.71E-01	Neutral	-1.71E-01	Neutral
90	p.Phe90Gln			11418	1955	4.77	3.57	-5.51E-02	Neutral	-5.51E-02	Neutral
90	p.Phe90Pro			13543	4974	5.66	9.08	-8.66E+00	Indeterminate	-8.66E+00	Indeterminate
90	p.Phe90Leu			12336	2179	5.15	3.98	-5.91E-02	Neutral	-5.91E-02	Neutral
90	p.Phe90Asp			8808	3457	3.68	6.31	-1.75E+01	Indeterminate	-1.75E+01	Indeterminate
90	p.Phe90Glu			12532	3273	5.23	5.97	-1.91E+00	Neutral	-1.91E+00	Neutral
90	p.Phe90Ala			9217	1762	3.85	3.22	-3.98E-01	Neutral	-3.98E-01	Neutral
90	p.Phe90Gly			12639	2896	5.28	5.28	-7.58E-01	Neutral	-7.58E-01	Neutral
90	p.Phe90Val			12878	3230	5.38	5.89	-1.40E+00	Neutral	-1.40E+00	Neutral
90	p.Phe90Tyr			13813	2502	5.77	4.57	-5.03E-02	Neutral	-5.03E-02	Neutral
90	p.Phe90Cys			14267	3205	5.96	5.85	-4.60E-01	Neutral	-4.60E-01	Neutral
90	p.Phe90Trp			13085	2766	5.46	5.05	-3.43E-01	Neutral	-3.43E-01	Neutral
90	p.Phe90Phe	Synonymous		10647	2410	4.45	4.40	-1.06E+00	Neutral	-1.06E+00	Neutral
91	p.Leu91Asn			8432	493	5.01	2.36	-2.02E-02	Neutral	-2.02E-02	Neutral

91	p.Leu91Lys			7857	938	4.67	4.49					-7.34E+00	Indeterminate			-7.34E+00	Indeterminate
91	p.Leu91Thr			7096	862	4.22	4.13					-8.95E+00	Indeterminate			-8.95E+00	Indeterminate
91	p.Leu91Arg			10604	1024	6.30	4.91					-1.49E+00	Neutral			-1.49E+00	Neutral
91	p.Leu91Ser			8848	1433	5.26	6.86					-1.82E+01	Indeterminate			-1.82E+01	Indeterminate
91	p.Leu91Ile			5518	782	3.28	3.75					-1.95E+01	Indeterminate			-1.95E+01	Indeterminate
91	p.Leu91Met			9789	2126	5.82	10.18					-3.62E+01	Indeterminate			-3.31E+01	Indeterminate
91	p.Leu91His			6249	874	3.71	4.19					-1.66E+01	Indeterminate			-1.66E+01	Indeterminate
91	p.Leu91Gln			7540	500	4.48	2.40					-1.59E-01	Neutral			-1.59E-01	Neutral
91	p.Leu91Pro			9411	1199	5.59	5.74					-7.49E+00	Indeterminate			-7.49E+00	Indeterminate
91	p.Leu91Leu	Synonymous		7934	662	4.72	3.17					-1.06E+00	Neutral			-1.06E+00	Neutral
91	p.Leu91Asp			6698	730	3.98	3.50					-6.28E+00	Indeterminate			-6.28E+00	Indeterminate
91	p.Leu91Glu			8804	987	5.23	4.73					-4.76E+00	Neutral			-4.76E+00	Neutral
91	p.Leu91Ala			8686	744	5.16	3.56					-1.03E+00	Neutral			-1.03E+00	Neutral
91	p.Leu91Gly			9719	1125	5.78	5.39					-4.74E+00	Neutral			-4.74E+00	Neutral
91	p.Leu91Val			11061	1191	6.58	5.71					-2.65E+00	Neutral			-2.65E+00	Neutral
91	p.Leu91Tyr			5704	1472	3.39	7.05					-5.32E+01	Deleterious			-5.32E+01	Deleterious
91	p.Leu91Cys			8443	679	5.02	3.25					-6.85E-01	Neutral			-6.85E-01	Neutral
91	p.Leu91Trp			10058	1333	5.98	6.39					-8.02E+00	Indeterminate			-8.02E+00	Indeterminate
91	p.Leu91Phe			9772	1720	5.81	8.24					-2.11E+01	Indeterminate			-2.11E+01	Indeterminate
92	p.Asp92Asn			3059	1748	3.61	3.58					-3.34E+00	Neutral			-3.34E+00	Neutral
92	p.Asp92Lys			2980	1988	3.51	4.07					-7.05E+00	Indeterminate			-7.05E+00	Indeterminate
92	p.Asp92Thr			3372	1636	3.97	3.35					-1.00E+00	Neutral			-1.00E+00	Neutral
92	p.Asp92Arg			5018	2780	5.92	5.69					-1.01E+00	Neutral			-1.01E+00	Neutral
92	p.Asp92Ser			2101	1098	2.48	2.25					-3.99E+00	Neutral			-3.99E+00	Neutral
92	p.Asp92Ile			5343	3437	6.30	7.04					-2.32E+00	Neutral			-2.32E+00	Neutral
92	p.Asp92Met			5318	2816	6.27	5.77					-6.09E-01	Neutral			-6.09E-01	Neutral
92	p.Asp92His			2930	1735	3.45	3.55					-4.28E+00	Neutral			-4.28E+00	Neutral
92	p.Asp92Gln			5536	3118	6.53	6.39					-8.92E-01	Neutral			-8.92E-01	Neutral
92	p.Asp92Pro			5268	3172	6.21	6.50					-1.59E+00	Neutral			-1.59E+00	Neutral
92	p.Asp92Leu			6269	3445	7.39	7.06					-5.25E-01	Neutral			-5.25E-01	Neutral
92	p.Asp92Asp	Synonymous		4573	2475	5.39	5.07					-1.06E+00	Neutral			-1.06E+00	Neutral
92	p.Asp92Glu			3137	1867	3.70	3.82					-3.93E+00	Neutral			-3.93E+00	Neutral
92	p.Asp92Ala			6689	4069	7.88	8.33					-9.91E-01	Neutral			-9.91E-01	Neutral
92	p.Asp92Gly			3137	1980	3.70	4.05					-5.17E+00	Neutral			-5.17E+00	Neutral
92	p.Asp92Val			5507	3063	6.49	6.27					-8.22E-01	Neutral			-8.22E-01	Neutral
92	p.Asp92Tyr			2568	1440	3.03	2.95					-4.06E+00	Neutral			-4.06E+00	Neutral
92	p.Asp92Cys			5479	2909	6.46	5.96					-5.73E-01	Neutral			-5.73E-01	Neutral
92	p.Asp92Trp			2854	1628	3.36	3.33					-3.72E+00	Neutral			-3.72E+00	Neutral
92	p.Asp92Phe			3697	2426	4.36	4.97					-4.82E+00	Neutral			-4.82E+00	Neutral
93	p.Thr93Asn			12872	1136	4.39	3.41					-1.65E+01	Indeterminate			-1.65E+01	Indeterminate
93	p.Thr93Lys			10656	1950	3.64	5.86					-5.32E+01	Deleterious			-5.32E+01	Deleterious
93	p.Thr93Thr	Synonymous		12435	599	4.24	1.80					-1.06E+00	Neutral			-1.06E+00	Neutral
93	p.Thr93Arg			12522	1486	4.27	4.47					-3.73E+01	Indeterminate			-3.31E+01	Indeterminate
93	p.Thr93Ser			11324	798	3.86	2.40					-8.91E+00	Indeterminate			-8.91E+00	Indeterminate
93	p.Thr93Ile			13017	614	4.44	1.85					-8.11E-01	Neutral			-8.11E-01	Neutral
93	p.Thr93Met			14173	1206	4.84	3.62					-1.33E+01	Indeterminate			-1.33E+01	Indeterminate
93	p.Thr93His			19146	2259	6.53	6.79					-2.59E+01	Indeterminate			-2.59E+01	Indeterminate
93	p.Thr93Gln			17574	1861	6.00	5.59					-2.08E+01	Indeterminate			-2.08E+01	Indeterminate
93	p.Thr93Pro			18332	5525	6.26	16.61					-5.32E+01	Deleterious			-5.32E+01	Deleterious
93	p.Thr93Leu			15110	1179	5.16	3.54					-9.12E+00	Indeterminate			-9.12E+00	Indeterminate
93	p.Thr93Asp			11110	1576	3.79	4.74					-5.32E+01	Deleterious			-5.32E+01	Deleterious
93	p.Thr93Glu			5928	693	2.02	2.08					-5.32E+01	Deleterious			-5.32E+01	Deleterious
93	p.Thr93Ala			18371	2050	6.27	6.16					-2.31E+01	Indeterminate			-2.31E+01	Indeterminate
93	p.Thr93Gly			21093	2490	7.20	7.48					-2.38E+01	Indeterminate			-2.38E+01	Indeterminate
93	p.Thr93Val			14806	1033	5.05	3.10					-5.99E+00	Indeterminate			-5.99E+00	Indeterminate
93	p.Thr93Tyr			15176	2095	5.18	6.30					-4.55E+01	Indeterminate			-3.32E+01	Indeterminate
93	p.Thr93Cys			14861	1164	5.07	3.50					-9.44E+00	Indeterminate			-9.44E+00	Indeterminate
93	p.Thr93Trp			16976	1890	5.79	5.68					-2.47E+01	Indeterminate			-2.47E+01	Indeterminate
93	p.Thr93Phe			17510	1668	5.98	5.01					-1.52E+01	Indeterminate			-1.52E+01	Indeterminate
94	p.Leu94Asn			1461	780	4.07	2.20					-3.44E+01	Indeterminate			-3.27E+01	Indeterminate
94	p.Leu94Lys			2375	3419	6.61	9.64					-5.32E+01	Deleterious			-5.32E+01	Deleterious
94	p.Leu94Thr			1880	1076	5.23	3.03					-3.32E+01	Indeterminate			-3.22E+01	Indeterminate
94	p.Leu94Arg			2092	3934	5.82	11.09					-5.32E+01	Deleterious			-5.32E+01	Deleterious
94	p.Leu94Ser			1901	421	5.29	1.19					-4.70E-01	Neutral			-4.70E-01	Neutral
94	p.Leu94Ile			1867	295	5.20	0.83					-8.36E-03	Neutral			-8.36E-03	Neutral
94	p.Leu94Met			432	42	1.20	0.12					-8.41E-03	Neutral			-8.41E-03	Neutral
94	p.Leu94His			1805	3659	5.02	10.31					-5.32E+01	Deleterious			-5.32E+01	Deleterious
94	p.Leu94Gln	Likely pathogenic		1288	1435	3.58	4.04					-5.32E+01	Deleterious			-5.32E+01	Deleterious
94	p.Leu94Pro	Likely pathogenic		2142	2900	5.96	8.17					-5.32E+01	Deleterious			-5.32E+01	Deleterious
94	p.Leu94Leu	Synonymous		1575	363	4.38	1.02					-1.06E+00	Neutral			-1.06E+00	Neutral
94	p.Leu94Asp			2051	3654	5.71	10.30					-5.32E+01	Deleterious			-5.32E+01	Deleterious
94	p.Leu94Glu			2084	2145	5.80	6.04					-5.32E+01	Deleterious			-5.32E+01	Deleterious
94	p.Leu94Ala			946	501	2.63	1.41					-4.70E+01	Indeterminate			-3.32E+01	Indeterminate
94	p.Leu94Gly			1187	1220	3.30	3.44					-5.32E+01	Deleterious			-5.32E+01	Deleterious
94	p.Leu94Val			1555	436	4.33	1.23					-3.56E+00	Neutral			-3.56E+00	Neutral
94	p.Leu94Tyr			1270	2783	3.53	7.84					-5.32E+01	Deleterious			-5.32E+01	Deleterious
94	p.Leu94Cys			2850	1202	7.93	3.39					-8.77E+00	Indeterminate			-8.77E+00	Indeterminate
94	p.Leu94Trp			2406	4027	6.70	11.35					-5.32E+01	Deleterious			-5.32E+01	Deleterious
94	p.Leu94Phe			2763	1193	7.69	3.36					-9.94E+00	Indeterminate			-9.94E+00	Indeterminate
95	p.Val95Asn			7232	2182	3.02	3.98					-4.64E+00	Neutral			-4.64E+00	Neutral
95	p.Val95Lys			10963	2844	4.58	5.19					-8.25E-01	Neutral			-8.25E-01	Neutral
95	p.Val95Thr			5808	1609	2.43	2.94					-4.38E+00	Neutral			-4.38E+00	Neutral
95	p.Val95Arg			9203	2809	3.84	5.13					-3.32E+00	Neutral			-3.32E+00	Neutral
95	p.Val95Ser			6861	2066	2.87	3.77					-4.99E+00	Neutral			-4.99E+00	Neutral
95	p.Val95Ile			8681	2394	3.63	4.37					-2.11E+00	Neutral			-2.11E+00	Neutral
95	p.Val95Met			4894	1367	2.04	2.49					-5.88E+00	Indeterminate			-5.88E+00	Indeterminate
95	p.Val95His			7829	1699	3.27	3.10					-4.78E-01	Neutral			-4.78E-01	Neutral
95	p.Val95Gln			2718	740	1.14	1.35					-1.13E+01	Indeterminate			-1.13E+01	Indeterminate
95	p.Val95Pro			5472	7300	2.29	13.32					-5.32E+01	Deleterious			-5.32E+01	Deleterious
95	p.Val95Leu			5183	1187	2.16	2.17					-1.93E+00	Neutral			-1.93E+00	Neutral
95	p.Val95Asp			7731	2073	3.23	3.78					-2.24E+00	Neutral			-2.24E+00	Neutral
95	p.Val95Glu			7407	2177	3.09	3.97					-3.95E+00	Neutral			-3.95E+00	Neutral
95	p.Val95Ala			6406	1317	2.68	2.40					-5.32E-01	Neutral			-5.32E-01	Neutral
95	p.Val95Gly			9626	2766	4.02	5.05					-2.19E+00	Neutral			-2.19E+00	Neutral
95	p.Val95Val	Synonymous		12313	3434	5.14	6.27					-1.06E+00	Neutral			-1.06E+00	Neutral
95	p.Val95Tyr			5221	1479	2.18	2.70					-5.69E+00	Neutral			-5.69E+00	Neutral
95	p.Val95Cys			6989	1603	2.92	2.92					-1.01E+00	Neutral			-1.01E+00	Neutral
95	p.Val95Trp			6758	2279	2.82	4.16					-8.18E+00	Indeterminate			-8.18E+00	Indeterminate
95	p.Val95Phe			5986	1846	2.50	3.37					-6.73E+00	Indeterminate			-6.73E+00	Indeterminate
96	p.Val96Asn			3048	665	5.44	4.93	5216	4257	5.13	6.03	-2.60E-01	Neutral	-7.47E+00	Indeterminate	-5.06E+00	Neutral
96	p.Val96Lys			4337	1119	7.74	8.30	7262	4566	7.14	6.47	-4.20E-01	Neutral	-1.03E+00	Neutral	-4.44E-01	Neutral
96	p.Val96Thr			3524	706	6.29	5.23	5839	3601	5.74	5.10	-5.74E-02	Neutral	-1.48E+00	Neutral	-4.91E-01	Neutral
96	p.Val96Arg			4400	1080	7.85	8.01	8595	5833	8.45	8.26	-2.49E-01	Neutral	-1.19E+00	Neutral	-4.39E-01	Neutral
96	p.Val96Ser			3519	765	6.28	5.67	6849	3584	6.73	5.08	-1.52E-01	Neutral	-2.52E-01	Neutral	-4.78E-02	Neutral
96	p.Val96Ile			1854	453	3.31	3.36	3334	2258	3.28	3.20	-2.21E+00	Neutral	-6.40E+00	Neutral	-5.81E+00	Indeterminate
96	p.Val96Met			2021	408	3.61	3.02	4039	2200	3.97	3.12	-4.52E-01	Neutral	-1.46E+00	Neutral	-6.96E-01	Neutral
96	p.Val96His			1796	486	3.21	3.60	3759	2299	3.70	3.26	-4.03E+00	Neutral	-3.31E+00	Neutral	-4.73E+00	Neutral
96	p.Val96Gln			2870	711	5.12	5.27	5857	4319	5.76	6.12	-9.26E-01	Neutral	-4.04E+00	Neutral	-2.82E+00	Neutral
96	p.Val96Pro			2688	727	4.80	5.39	6159	3568	6.06	5.05	-1.94E+00	Neutral	-8.38E-01	Neutral	-1.23E+00	Neutral
96	p.Val96Leu			1684	426	3.01	3.16	3163	2563	3.11	3.63	-3.19E+00	Neutral	-1.33E+01	Indeterminate	-1.28E+01	Indeterminate
96	p.Val96Asp			2748	651	4.90	4.83	3473	2913	3.41	4.13	-7.34E-01	Neutral	-1.34E+01	Indeterminate	-1.07E+01	Indeterminate
96	p.Val96Glu			3956	740	7.06	5.49	6847	4820	6.73	6.83	-1.29E-02	Neutral	-2.41E+00	Neutral	-1.00E+00	Neutral
96	p.Val96Ala			1027	328	1.83	2.43	1739	1778	1.71	2.52	-1.57E+01	Indeterminate	-4.25E+01	Indeterminate	-5.32E+01	Deleterious
96	p.Val96Gly			2331	764	4.16	5.66	4461	2549	4.39	3.61	-6.46E+00	Indeterminate	-1.61E+00	Neutral	-5.35E+00	Neutral
96	p.Val96Val	Synonymous		4346	1259	7.76	9.33	8569	5719	8.43	8.10	-1.06E+00	Neutral	-1.06E+00	Neutral	-8.18E-01	Neutral
96	p.Val96Tyr			1651	394	2.95	2.92	3353	2410	3.30	3.41	-2.41E+00	Neutral	-8.07E+00	Indeterminate	-7.43E+00	Indeterminate
96	p.Val96Cys			3437	820	6.13	6.08	5233	4773	5.15	6.76	-4.16E-01	Neutral	-1.13E+01	Indeterminate	-8.48E+00	Indeterminate
96	p.Val96Trp			2216	450	3.96	3.34	3357	3261	3.30	4.62	-3.61E-01	Neutral	-2.16E+01	Indeterminate	-1.80E+01	Indeterminate
96	p.Val96Phe			2574	536	4.59	3.97	4599	3318	4.52	4.70	-2.85E-01	Neutral	-5.30E+00	Neutral	-3.30E+00	Neutral
97	p.Leu97Asn			2906	1928	3.97	5.53					-5.32E+01	Deleterious			-5.32E+01	Deleterious
97	p.Leu97Lys			5234	3867	7.14	11.09					-5.32E+01	Deleterious			-5.32E+01	Deleterious
97	p.Leu97Thr			2517	1324	3.43	3.80					-5.32E+01	Deleterious			-5.32E+01	Deleterious
97	p.Leu97Arg		Deleterious	3861	2940	5.27	8.43					-5.32E+01	Deleterious			-5.32E+01	Deleterious
97	p.Leu97Ser			5025	3430	6.86	9.84					-5.32E+01	Deleterious			-5.32E+01	Deleterious
97	p.Leu97Ile			3028	229	4.13	0.66					-2.64E-01	Neutral			-2.64E-01	Neutral
97	p.Leu97Met			2875	178	3.92	0.51					-3.03E-02	Neutral			-3.03E-02	Neutral
97	p.Leu97His			2448	1356	3.34	3.89					-5.32E+01	Deleterious			-5.32E+01	Deleterious
97	p.Leu97Gln			3798	1421	5.18	4.08					-5.32E+01	Deleterious			-5.32E+01	Deleterious
97	p.Leu97Pro			2652	2066	3.62	5.93					-5.32E+01	Deleterious			-5.32E+01	Deleterious
97	p.Leu97Leu	Synonymous		3046	272	4.16	0.78					-1.06E+00	Neutral			-1.06E+00	Neutral
97	p.Leu97Asp			4997	3900	6.82	11.19					-5.32E+01	Deleterious			-5.32E+01	Deleterious
97	p.Leu97Glu			4583	2785	6.25	7.99					-5.32E+01	Deleterious			-5.32E+01	Deleterious
97	p.Leu97Ala			3439	1472	4.69	4.22					-5.32E+01	Deleterious			-5.32E+01	Deleterious
97	p.Leu97Gly			4508	3527	6.15	10.12					-5.32E+01	Deleterious			-5.32E+01	Deleterious
97	p.Leu97Val			3310	497	4.52	1.43					-1.19E+01	Indeterminate			-1.19E+01	Indeterminate
97	p.Leu97Tyr			3469	313	4.73	0.90					-8.32E-01	Neutral			-8.32E-01	Neutral
97	p.Leu97Cys			3690	339	5.04	0.97					-8.16E-01	Neutral			-8.16E-01	Neutral
97	p.Leu97Trp			4586	2750	6.26	7.89					-5.32E+01	Deleterious			-5.32E+01	Deleterious
97	p.Leu97Phe			3313	273	4.52	0.78					-4.46E-01	Neutral			-4.46E-01	Neutral

98	p.His98Asn			6962	3204	5.23	4.40	-6.69E-01	Neutral	-6.69E-01	Neutral
98	p.His98Lys			3696	1535	2.77	2.11	-1.47E+00	Neutral	-1.47E+00	Neutral
98	p.His98Thr			4272	2069	3.21	2.84	-2.77E+00	Neutral	-2.77E+00	Neutral
98	p.His98Arg			7635	3456	5.73	4.75	-4.48E-01	Neutral	-4.48E-01	Neutral
98	p.His98Ser			6500	3794	4.88	5.21	-3.55E+00	Neutral	-3.55E+00	Neutral
98	p.His98Ile			6867	3037	5.15	4.17	-4.98E-01	Neutral	-4.98E-01	Neutral
98	p.His98Met			5455	2395	4.09	3.29	-8.72E-01	Neutral	-8.72E-01	Neutral
98	p.His98His	Synonymous		5527	2503	4.15	3.44	-1.06E+00	Neutral	-1.06E+00	Neutral
98	p.His98Gln			4315	2331	3.24	3.20	-4.71E+00	Neutral	-4.71E+00	Neutral
98	p.His98Pro		Deleterious	4682	11420	3.51	15.68	-5.32E+01	Deleterious	-5.32E+01	Deleterious
98	p.His98Leu			5788	3026	4.34	4.16	-2.42E+00	Neutral	-2.42E+00	Neutral
98	p.His98Asp			5699	3256	4.28	4.47	-3.97E+00	Neutral	-3.97E+00	Neutral
98	p.His98Glu			8665	4094	6.50	5.62	-4.51E-01	Neutral	-4.51E-01	Neutral
98	p.His98Ala			8015	3389	6.02	4.65	-1.99E-01	Neutral	-1.99E-01	Neutral
98	p.His98Gly			5917	3101	4.44	4.26	-2.35E+00	Neutral	-2.35E+00	Neutral
98	p.His98Val			12966	6294	9.73	8.64	-1.58E-01	Neutral	-1.58E-01	Neutral
98	p.His98Tyr			8668	4200	6.51	5.77	-5.61E-01	Neutral	-5.61E-01	Neutral
98	p.His98Cys			7543	3723	5.66	5.11	-9.36E-01	Neutral	-9.36E-01	Neutral
98	p.His98Trp			7222	3059	5.42	4.20	-2.87E-01	Neutral	-2.87E-01	Neutral
98	p.His98Phe			6849	2927	5.14	4.02	-3.69E-01	Neutral	-3.69E-01	Neutral
99	p.Arg99Asn			2603	447	5.58	2.32	-8.76E+00	Indeterminate	-8.76E+00	Indeterminate
99	p.Arg99Lys			2018	299	4.32	1.55	-6.84E+00	Indeterminate	-6.84E+00	Indeterminate
99	p.Arg99Thr			1591	288	3.41	1.49	-1.80E+01	Indeterminate	-1.80E+01	Indeterminate
99	p.Arg99Arg	Synonymous		3293	407	7.05	2.11	-1.06E+00	Neutral	-1.06E+00	Neutral
99	p.Arg99Ser			2572	463	5.51	2.40	-1.06E+01	Indeterminate	-1.06E+01	Indeterminate
99	p.Arg99Ile			2237	347	4.79	1.80	-7.17E+00	Indeterminate	-7.17E+00	Indeterminate
99	p.Arg99Met			2430	480	5.20	2.49	-1.53E+01	Indeterminate	-1.53E+01	Indeterminate
99	p.Arg99His			2305	267	4.94	1.38	-1.53E+00	Neutral	-1.53E+00	Neutral
99	p.Arg99Gln			1726	197	3.70	1.02	-2.51E+00	Neutral	-2.51E+00	Neutral
99	p.Arg99Pro	Likely pathogenic		2397	11387	5.13	59.05	-5.32E+01	Deleterious	-5.32E+01	Deleterious
99	p.Arg99Leu			1736	369	3.72	1.91	-2.60E+01	Indeterminate	-2.60E+01	Indeterminate
99	p.Arg99Asp			1995	474	4.27	2.46	-3.08E+01	Indeterminate	-3.05E+01	Indeterminate
99	p.Arg99Glu			2272	392	4.87	2.03	-1.05E+01	Indeterminate	-1.05E+01	Indeterminate
99	p.Arg99Ala			2818	806	6.04	4.18	-3.66E+01	Indeterminate	-3.31E+01	Indeterminate
99	p.Arg99Gly		Neutral	3322	420	7.12	2.18	-1.22E+00	Neutral	-1.22E+00	Neutral
99	p.Arg99Val			1847	518	3.96	2.69	-4.81E+01	Indeterminate	-3.32E+01	Indeterminate
99	p.Arg99Tyr			2348	308	5.03	1.60	-3.07E+00	Neutral	-3.07E+00	Neutral
99	p.Arg99Cys			2310	505	4.95	2.62	-2.17E+01	Indeterminate	-2.17E+01	Indeterminate
99	p.Arg99Trp			2612	591	5.59	3.06	-2.13E+01	Indeterminate	-2.13E+01	Indeterminate
99	p.Arg99Phe			2255	318	4.83	1.65	-4.69E+00	Neutral	-4.69E+00	Neutral
100	p.Ala100Asn			3418	332	2.33	1.28	-1.08E+00	Neutral	-1.08E+00	Neutral
100	p.Ala100Lys			13759	1148	9.36	4.42	-3.54E-04	Neutral	-3.54E-04	Neutral
100	p.Ala100Thr			5473	648	3.72	2.49	-1.44E+00	Neutral	-1.44E+00	Neutral
100	p.Ala100Arg			14131	1152	9.61	4.43	-1.87E-04	Neutral	-1.87E-04	Neutral
100	p.Ala100Ser	Likely benign		3813	249	2.59	0.96	-8.77E-03	Neutral	-8.77E-03	Neutral
100	p.Ala100Ile			6693	620	4.55	2.38	-8.43E-02	Neutral	-8.43E-02	Neutral
100	p.Ala100Met			14631	1686	9.95	6.49	-5.02E-02	Neutral	-5.02E-02	Neutral
100	p.Ala100His			7412	516	5.04	1.98	-5.68E-04	Neutral	-5.68E-04	Neutral
100	p.Ala100Gln			6694	604	4.55	2.32	-6.09E-02	Neutral	-6.09E-02	Neutral
100	p.Ala100Pro		Deleterious	10531	12616	7.16	48.53	-5.32E+01	Deleterious	-5.32E+01	Deleterious
100	p.Ala100Leu			6791	525	4.62	2.02	-6.08E-03	Neutral	-6.08E-03	Neutral
100	p.Ala100Asp			1017	66	0.69	0.25	-1.07E+00	Neutral	-1.07E+00	Neutral
100	p.Ala100Glu			7528	1165	5.12	4.48	-3.66E+00	Neutral	-3.66E+00	Neutral
100	p.Ala100Ala	Synonymous		2608	231	1.77	0.89	-1.06E+00	Neutral	-1.06E+00	Neutral
100	p.Ala100Gly			5224	1793	3.55	6.90	-5.32E+01	Deleterious	-5.32E+01	Deleterious
100	p.Ala100Val			9737	705	6.62	2.71	-2.07E-04	Neutral	-2.07E-04	Neutral
100	p.Ala100Tyr			3636	375	2.47	1.44	-1.42E+00	Neutral	-1.42E+00	Neutral
100	p.Ala100Cys			4099	233	2.79	0.90	-5.06E-04	Neutral	-5.06E-04	Neutral
100	p.Ala100Trp			15058	1024	10.24	3.94	-2.12E-06	Neutral	-2.12E-06	Neutral
100	p.Ala100Phe			4733	309	3.22	1.19	-2.66E-03	Neutral	-2.66E-03	Neutral
101	p.Gly101Asn			16195	7975	4.69	2.29	-6.43E+00	Indeterminate	-6.43E+00	Indeterminate
101	p.Gly101Lys			18728	11869	5.42	3.41	-1.36E+01	Indeterminate	-1.36E+01	Indeterminate
101	p.Gly101Thr			14607	10765	4.23	3.09	-2.69E+01	Indeterminate	-2.68E+01	Indeterminate
101	p.Gly101Arg		Neutral	17888	10294	5.18	2.95	-1.03E+01	Indeterminate	-1.03E+01	Indeterminate
101	p.Gly101Ser			20113	10885	5.82	3.12	-7.05E+00	Indeterminate	-7.05E+00	Indeterminate
101	p.Gly101Ile			17140	56282	4.96	16.15	-5.32E+01	Deleterious	-5.32E+01	Deleterious
101	p.Gly101Met			24756	17567	7.16	5.04	-1.45E+01	Indeterminate	-1.45E+01	Indeterminate
101	p.Gly101His			12394	6745	3.59	1.94	-1.30E+01	Indeterminate	-1.30E+01	Indeterminate
101	p.Gly101Gln			19989	10844	5.78	3.11	-7.18E+00	Indeterminate	-7.18E+00	Indeterminate
101	p.Gly101Pro			13085	38084	3.79	10.93	-5.32E+01	Deleterious	-5.32E+01	Deleterious
101	p.Gly101Leu			17138	20603	4.96	5.91	-5.32E+01	Deleterious	-5.32E+01	Deleterious
101	p.Gly101Asp			16905	7563	4.89	2.17	-3.90E+00	Neutral	-3.90E+00	Neutral
101	p.Gly101Glu			16990	10748	4.92	3.08	-1.50E+01	Indeterminate	-1.50E+01	Indeterminate
101	p.Gly101Ala			14073	7741	4.07	2.22	-1.17E+01	Indeterminate	-1.17E+01	Indeterminate
101	p.Gly101Gly	Synonymous		14598	4998	4.22	1.43	-1.06E+00	Neutral	-1.06E+00	Neutral
101	p.Gly101Val			14907	44442	4.31	12.75	-5.32E+01	Deleterious	-5.32E+01	Deleterious
101	p.Gly101Tyr			15482	13387	4.48	3.84	-3.80E+01	Indeterminate	-3.32E+01	Indeterminate
101	p.Gly101Cys			26741	15085	7.74	4.33	-5.64E+00	Neutral	-5.64E+00	Neutral
101	p.Gly101Trp	Pathogenic		21842	33554	6.32	9.63	-5.32E+01	Deleterious	-5.32E+01	Deleterious
101	p.Gly101Phe			12038	9008	3.48	2.59	-3.28E+01	Indeterminate	-3.20E+01	Indeterminate
102	p.Ala102Asn			1416	161	0.51	0.23	-5.32E+01	Deleterious	-5.32E+01	Deleterious
102	p.Ala102Lys			25422	7632	9.21	10.79	-5.32E+01	Deleterious	-5.32E+01	Deleterious
102	p.Ala102Thr			90	11	0.03	0.02	-5.32E+01	Deleterious	-5.32E+01	Deleterious
102	p.Ala102Arg			16482	8234	5.97	11.65	-5.32E+01	Deleterious	-5.32E+01	Deleterious
102	p.Ala102Ser			6001	154	2.17	0.22	-5.32E+01	Deleterious	-5.32E+01	Deleterious
102	p.Ala102Ile			14717	2928	5.33	4.14	-5.32E+01	Deleterious	-5.32E+01	Deleterious
102	p.Ala102Met			12041	3575	4.36	5.06	-5.32E+01	Deleterious	-5.32E+01	Deleterious
102	p.Ala102His			10705	2037	3.88	2.88	-5.32E+01	Deleterious	-5.32E+01	Deleterious
102	p.Ala102Gln			20898	5855	7.57	8.28	-5.32E+01	Deleterious	-5.32E+01	Deleterious
102	p.Ala102Pro			1015	335	0.37	0.47	-5.32E+01	Deleterious	-5.32E+01	Deleterious
102	p.Ala102Leu			19464	8187	7.05	11.58	-5.32E+01	Deleterious	-5.32E+01	Deleterious
102	p.Ala102Asp			15694	4695	5.69	6.64	-5.32E+01	Deleterious	-5.32E+01	Deleterious
102	p.Ala102Glu			22596	8973	8.19	12.69	-5.32E+01	Deleterious	-5.32E+01	Deleterious
102	p.Ala102Ala	Synonymous		17069	184	6.18	0.26	-1.06E+00	Neutral	-1.06E+00	Neutral
102	p.Ala102Gly			8592	52	3.11	0.07	-2.94E-02	Neutral	-2.94E-02	Neutral
102	p.Ala102Val			31839	787	11.54	1.11	-1.80E+01	Indeterminate	-1.80E+01	Indeterminate
102	p.Ala102Tyr			7303	3519	2.65	4.98	-5.32E+01	Deleterious	-5.32E+01	Deleterious
102	p.Ala102Cys			9054	131	3.28	0.19	-1.24E+01	Indeterminate	-1.24E+01	Indeterminate
102	p.Ala102Trp			27880	10063	10.10	14.23	-5.32E+01	Deleterious	-5.32E+01	Deleterious
102	p.Ala102Phe			7718	3189	2.80	4.51	-5.32E+01	Deleterious	-5.32E+01	Deleterious
103	p.Arg103Asn			6943	7127	8.93	8.95	-1.36E-02	Neutral	-1.36E-02	Neutral
103	p.Arg103Lys			3330	3626	4.28	4.55	-4.82E-01	Neutral	-4.82E-01	Neutral
103	p.Arg103Thr			3053	4907	3.93	6.16	-6.16E+00	Indeterminate	-6.16E+00	Indeterminate
103	p.Arg103Arg	Synonymous		2349	2516	3.02	3.16	-1.06E+00	Neutral	-1.06E+00	Neutral
103	p.Arg103Ser			2005	1352	2.58	1.70	-1.21E-02	Neutral	-1.21E-02	Neutral
103	p.Arg103Ile			3142	4421	4.04	5.55	-3.14E+00	Neutral	-3.14E+00	Neutral
103	p.Arg103Met			1311	1440	1.69	1.81	-3.90E+00	Neutral	-3.90E+00	Neutral
103	p.Arg103His			1356	1198	1.74	1.50	-9.86E-01	Neutral	-9.86E-01	Neutral
103	p.Arg103Gln			2359	2500	3.03	3.14	-9.72E-01	Neutral	-9.72E-01	Neutral
103	p.Arg103Pro			1548	1348	1.99	1.69	-6.26E-01	Neutral	-6.26E-01	Neutral
103	p.Arg103Leu			2506	1742	3.22	2.19	-6.08E-03	Neutral	-6.08E-03	Neutral
103	p.Arg103Asp			5113	4011	6.58	5.03	-7.98E-04	Neutral	-7.98E-04	Neutral
103	p.Arg103Glu			753	642	0.97	0.81	-2.72E+00	Neutral	-2.72E+00	Neutral
103	p.Arg103Ala			6092	5272	7.83	6.62	-1.66E-03	Neutral	-1.66E-03	Neutral
103	p.Arg103Gly			7131	7369	9.17	9.25	-1.32E-02	Neutral	-1.32E-02	Neutral
103	p.Arg103Val			2730	2488	3.51	3.12	-1.62E-01	Neutral	-1.62E-01	Neutral
103	p.Arg103Tyr			3731	4354	4.80	5.47	-6.30E-01	Neutral	-6.30E-01	Neutral
103	p.Arg103Cys			10170	11475	13.08	14.40	-8.28E-03	Neutral	-8.28E-03	Neutral
103	p.Arg103Trp			2854	2738	3.67	3.44	-2.37E-01	Neutral	-2.37E-01	Neutral
103	p.Arg103Phe			9286	9137	11.94	11.47	-1.35E-03	Neutral	-1.35E-03	Neutral
104	p.Leu104Asn			5163	1602	5.50	3.07	-6.56E-09	Neutral	-6.41E-09	Neutral
104	p.Leu104Lys			4859	6969	5.17	13.35	-1.14E+01	Indeterminate	-1.14E+01	Indeterminate
104	p.Leu104Thr			2937	952	3.13	1.82	-2.40E-06	Neutral	-2.40E-06	Neutral
104	p.Leu104Arg			5550	15703	5.91	30.09	-5.32E+01	Deleterious	-5.32E+01	Deleterious
104	p.Leu104Ser			2630	681	2.80	1.31	-2.09E-08	Neutral	-2.08E-08	Neutral
104	p.Leu104Ile			4061	634	4.32	1.21	0.00E+00	Neutral	0.00E+00	Neutral
104	p.Leu104Met			6322	1029	6.73	1.97	0.00E+00	Neutral	0.00E+00	Neutral
104	p.Leu104His			3101	650	3.30	1.25	-1.12E-11	Neutral	0.00E+00	Neutral
104	p.Leu104Gln			4063	2097	4.32	4.02	-3.69E-03	Neutral	-3.69E-03	Neutral
104	p.Leu104Pro			1101	388	1.17	0.74	-8.64E-03	Neutral	-8.64E-03	Neutral
104	p.Leu104Leu	Synonymous		2345	1636	2.50	3.14	-1.06E+00	Neutral	-1.06E+00	Neutral
104	p.Leu104Asp			5290	1684	5.63	3.23	-1.05E-08	Neutral	-1.04E-08	Neutral
104	p.Leu104Glu			6303	3154	6.71	6.04	-1.33E-04	Neutral	-1.33E-04	Neutral
104	p.Leu104Ala			3493	486	3.72	0.93	0.00E+00	Neutral	0.00E+00	Neutral
104	p.Leu104Gly			3859	11170	4.11	21.41	-5.32E+01	Deleterious	-5.32E+01	Deleterious
104	p.Leu104Val			10072	959	10.72	1.84	0.00E+00	Neutral	0.00E+00	Neutral
104	p.Leu104Tyr			5704	811	6.07	1.55	0.00E+00	Neutral	0.00E+00	Neutral
104	p.Leu104Cys			3887	660	4.14	1.26	-1.44E-15	Neutral	0.00E+00	Neutral
104	p.Leu104Trp			7298	511	7.77	0.98	0.00E+00	Neutral	0.00E+00	Neutral

104	p.Leu104Phe			5907	407	6.29	0.78					0.00E+00	Neutral			0.00E+00	Neutral
105	p.Asp105Asn			2615	618	3.97	2.03					-2.86E+00	Neutral			-2.86E+00	Neutral
105	p.Asp105Lys			4759	2093	7.22	6.86					-1.59E+01	Indeterminate			-1.59E+01	Indeterminate
105	p.Asp105Thr			2587	857	3.92	2.81					-1.22E+01	Indeterminate			-1.22E+01	Indeterminate
105	p.Asp105Arg			4066	2281	6.17	7.48					-3.54E+01	Indeterminate			-3.29E+01	Indeterminate
105	p.Asp105Ser			985	469	1.49	1.54					-5.32E+01	Deleterious			-5.32E+01	Deleterious
105	p.Asp105Ile			2485	1504	3.77	4.93					-5.32E+01	Deleterious			-5.32E+01	Deleterious
105	p.Asp105Met			2222	756	3.37	2.48					-1.57E+01	Indeterminate			-1.57E+01	Indeterminate
105	p.Asp105His			3979	1502	6.04	4.92					-1.17E+01	Indeterminate			-1.17E+01	Indeterminate
105	p.Asp105Gln			3702	1586	5.62	5.20					-1.89E+01	Indeterminate			-1.89E+01	Indeterminate
105	p.Asp105Pro			2531	5497	3.84	18.01					-5.32E+01	Deleterious			-5.32E+01	Deleterious
105	p.Asp105Leu			3705	1568	5.62	5.14					-1.82E+01	Indeterminate			-1.82E+01	Indeterminate
105	p.Asp105Asp	Synonymous		3838	879	5.82	2.88					-1.06E+00	Neutral			-1.06E+00	Neutral
105	p.Asp105Glu			5734	2764	8.70	9.06					-1.74E+01	Indeterminate			-1.74E+01	Indeterminate
105	p.Asp105Ala			4005	1276	6.08	4.18					-6.08E+00	Indeterminate			-6.08E+00	Indeterminate
105	p.Asp105Gly			1562	963	2.37	3.16					-5.32E+01	Deleterious			-5.32E+01	Deleterious
105	p.Asp105Val			4690	2554	7.11	8.37					-2.92E+01	Indeterminate			-2.92E+01	Indeterminate
105	p.Asp105Tyr			3757	461	5.70	1.51					-3.22E-04	Neutral			-3.22E-04	Neutral
105	p.Asp105Cys			1256	166	1.91	0.54					-2.28E-01	Neutral			-2.28E-01	Neutral
105	p.Asp105Trp			3585	1128	5.44	3.70					-6.73E+00	Indeterminate			-6.73E+00	Indeterminate
105	p.Asp105Phe			3861	1593	5.86	5.22					-1.61E+01	Indeterminate			-1.61E+01	Indeterminate
106	p.Val106Asn			1125	3424	4.79	5.65	5445	1988	4.36	4.81	-7.07E+00	Indeterminate	-4.25E+00	Neutral	-8.17E+00	Indeterminate
106	p.Val106Lys			1023	4025	4.36	6.64	7514	2575	6.02	6.23	-1.90E+01	Indeterminate	-1.66E+00	Neutral	-1.67E+01	Indeterminate
106	p.Val106Thr			979	2138	4.17	3.53	5546	1736	4.44	4.20	-1.66E+00	Neutral	-1.77E+00	Neutral	-1.68E+00	Neutral
106	p.Val106Arg			641	2444	2.73	4.03	3991	1125	3.20	2.72	-2.67E+01	Indeterminate	-1.82E+00	Neutral	-2.41E+01	Indeterminate
106	p.Val106Ser			1510	3703	6.44	6.11	7597	2262	6.08	5.47	-1.39E+00	Neutral	-5.78E-01	Neutral	-7.25E-01	Neutral
106	p.Val106Ile			1265	1935	5.39	3.19	6656	2352	5.33	5.69	-2.05E-02	Neutral	-2.54E+00	Neutral	-1.09E+00	Neutral
106	p.Val106Met			1360	3085	5.80	5.09	7476	2399	5.99	5.80	-1.03E+00	Neutral	-1.08E+00	Neutral	-8.05E-01	Neutral
106	p.Val106His			1415	3084	6.03	5.09	7083	2284	5.67	5.52	-6.83E-01	Neutral	-1.26E+00	Neutral	-7.14E-01	Neutral
106	p.Val106Gln			1752	2941	7.47	4.85	6941	2628	5.56	6.35	-1.64E-02	Neutral	-3.41E+00	Neutral	-1.67E+00	Neutral
106	p.Val106Pro			827	3185	3.52	5.26	4716	1641	3.78	3.97	-2.18E+01	Indeterminate	-4.24E+00	Neutral	-2.18E+01	Indeterminate
106	p.Val106Leu			633	1732	2.70	2.86	4193	1337	3.36	3.23	-9.78E+00	Indeterminate	-3.33E+00	Neutral	-9.78E+00	Indeterminate
106	p.Val106Asp			1686	3754	7.19	6.19	7863	2900	6.30	7.01	-5.03E-01	Neutral	-2.36E+00	Neutral	-1.29E+00	Neutral
106	p.Val106Glu			517	1854	2.20	3.06	2857	932	2.29	2.25	-2.74E+01	Indeterminate	-6.67E+00	Indeterminate	-2.94E+01	Indeterminate
106	p.Val106Ala			1537	5106	6.55	8.43	7541	2613	6.04	6.32	-6.62E+00	Indeterminate	-1.77E+00	Neutral	-5.62E+00	Neutral
106	p.Val106Gly			1091	3828	4.65	6.32	7234	2084	5.79	5.04	-1.24E+01	Indeterminate	-5.01E-01	Neutral	-9.59E+00	Indeterminate
106	p.Val106Val	Synonymous		1338	3033	5.70	5.00	6396	1945	5.12	4.70	-1.06E+00	Neutral	-1.06E+00	Neutral	-8.18E-01	Neutral
106	p.Val106Tyr			1292	2558	5.51	4.22	5942	2069	4.76	5.00	-3.88E-01	Neutral	-2.88E+00	Neutral	-1.56E+00	Neutral
106	p.Val106Cys			1632	4660	6.96	7.69	9313	2868	7.46	6.93	-2.96E+00	Neutral	-4.31E-01	Neutral	-1.64E+00	Neutral
106	p.Val106Trp			338	818	1.44	1.35	2610	734	2.09	1.77	-1.32E+01	Indeterminate	-3.91E+00	Neutral	-1.34E+01	Indeterminate
106	p.Val106Phe			1504	3293	6.41	5.43	7973	2886	6.38	6.98	-6.01E-01	Neutral	-2.06E+00	Neutral	-1.15E+00	Neutral
107	p.Arg107Asn			4635	525	4.04	4.09	1667	4330	4.10	4.93	-1.50E+00	Neutral	-3.32E+00	Neutral	-2.70E+00	Neutral
107	p.Arg107Lys			4356	469	3.80	3.65	1642	3493	4.04	3.98	-1.23E+00	Neutral	-1.00E+00	Neutral	-8.82E-01	Neutral
107	p.Arg107Thr			7105	785	6.19	6.11	2387	4172	5.87	4.75	-4.16E-01	Neutral	-3.90E-02	Neutral	-5.96E-02	Neutral
107	p.Arg107Arg	Synonymous		6674	811	5.82	6.31	2006	4597	4.94	5.24	-1.06E+00	Neutral	-1.06E+00	Neutral	-8.18E-01	Neutral
107	p.Arg107Ser			5046	684	4.40	5.32	1875	4722	4.61	5.38	-3.59E+00	Neutral	-2.26E+00	Neutral	-3.51E+00	Neutral
107	p.Arg107Ile			3528	390	3.08	3.04	1126	1863	2.77	2.12	-2.26E+00	Neutral	-3.53E-01	Neutral	-1.12E+00	Neutral
107	p.Arg107Met			7376	804	6.43	6.26	2724	5759	6.70	6.56	-3.28E-01	Neutral	-2.20E-01	Neutral	-8.34E-02	Neutral
107	p.Arg107His			4098	438	3.57	3.41	1418	3207	3.49	3.65	-1.34E+00	Neutral	-2.07E+00	Neutral	-1.66E+00	Neutral
107	p.Arg107Gln			10107	1081	8.81	8.41	4069	7524	10.01	8.57	-8.71E-02	Neutral	-6.53E-03	Neutral	-2.91E-03	Neutral
107	p.Arg107Pro			6819	747	5.94	5.81	2662	5187	6.55	5.91	-4.36E-01	Neutral	-9.73E-02	Neutral	-7.95E-02	Neutral
107	p.Arg107Leu			6193	712	5.40	5.54	2041	5611	5.02	6.39	-8.46E-01	Neutral	-3.14E+00	Neutral	-2.08E+00	Neutral
107	p.Arg107Asp			3847	431	3.35	3.35	1546	3341	3.80	3.81	-2.07E+00	Neutral	-1.29E+00	Neutral	-1.62E+00	Neutral
107	p.Arg107Glu			3892	481	3.39	3.74	1098	2698	2.70	3.07	-3.48E+00	Neutral	-4.97E+00	Neutral	-5.67E+00	Neutral
107	p.Arg107Ala			8763	943	7.64	7.34	3149	6188	7.75	7.05	-1.61E-01	Neutral	-5.43E-02	Neutral	-1.46E-02	Neutral
107	p.Arg107Gly			5027	563	4.38	4.38	1622	3862	3.99	4.40	-1.16E+00	Neutral	-2.16E+00	Neutral	-1.60E+00	Neutral
107	p.Arg107Val			7473	764	6.51	5.95	2752	6282	6.77	7.16	-1.64E-01	Neutral	-4.44E-01	Neutral	-1.00E-01	Neutral
107	p.Arg107Tyr			5383	660	4.69	5.14	1758	3648	4.33	4.16	-1.82E+00	Neutral	-6.97E-01	Neutral	-1.06E+00	Neutral
107	p.Arg107Cys			2751	264	2.40	2.05	1066	3094	2.62	3.52	-1.58E+00	Neutral	-1.01E+01	Indeterminate	-8.51E+00	Indeterminate
107	p.Arg107Trp			6378	683	5.56	5.32	2287	4275	5.63	4.87	-4.34E-01	Neutral	-1.08E-01	Neutral	-8.19E-02	Neutral
107	p.Arg107Phe			5273	613	4.60	4.77	1737	3940	4.27	4.49	-1.35E+00	Neutral	-1.38E+00	Neutral	-1.19E+00	Neutral
108	p.Asp108Asn		Neutral	2885	264	5.26	1.09					-7.77E-01	Neutral			-7.77E-01	Neutral
108	p.Asp108Lys			2742	1549	5.00	6.37					-5.32E+01	Deleterious			-5.32E+01	Deleterious
108	p.Asp108Thr			3646	860	6.65	3.54					-3.14E+01	Indeterminate			-3.10E+01	Indeterminate
108	p.Asp108Arg			3490	2166	6.36	8.91					-5.32E+01	Deleterious			-5.32E+01	Deleterious
108	p.Asp108Ser			4074	1584	7.43	6.52					-5.32E+01	Deleterious			-5.32E+01	Deleterious
108	p.Asp108Ile			3055	1607	5.57	6.61					-5.32E+01	Deleterious			-5.32E+01	Deleterious
108	p.Asp108Met			3341	1731	6.09	7.12					-5.32E+01	Deleterious			-5.32E+01	Deleterious
108	p.Asp108His		Deleterious	1882	1059	3.43	4.36					-5.32E+01	Deleterious			-5.32E+01	Deleterious
108	p.Asp108Gln			2051	1059	3.74	4.36					-5.32E+01	Deleterious			-5.32E+01	Deleterious
108	p.Asp108Pro			2292	1346	4.18	5.54					-5.32E+01	Deleterious			-5.32E+01	Deleterious
108	p.Asp108Leu			1845	1018	3.36	4.19					-5.32E+01	Deleterious			-5.32E+01	Deleterious
108	p.Asp108Asp	Synonymous		2828	268	5.16	1.10					-1.06E+00	Neutral			-1.06E+00	Neutral
108	p.Asp108Glu			1895	256	3.46	1.05					-1.17E+01	Indeterminate			-1.17E+01	Indeterminate
108	p.Asp108Ala			1228	713	2.24	2.93					-5.32E+01	Deleterious			-5.32E+01	Deleterious
108	p.Asp108Gly			2077	1202	3.79	4.95					-5.32E+01	Deleterious			-5.32E+01	Deleterious
108	p.Asp108Val			2618	1408	4.77	5.79					-5.32E+01	Deleterious			-5.32E+01	Deleterious
108	p.Asp108Tyr		Deleterious	3107	1750	5.67	7.20					-5.32E+01	Deleterious			-5.32E+01	Deleterious
108	p.Asp108Cys			3596	1073	6.56	4.42					-5.32E+01	Deleterious			-5.32E+01	Deleterious
108	p.Asp108Trp			2630	1506	4.80	6.20					-5.32E+01	Deleterious			-5.32E+01	Deleterious
108	p.Asp108Phe			3562	1880	6.49	7.74					-5.32E+01	Deleterious			-5.32E+01	Deleterious
109	p.Ala109Asn			5087	2168	5.92	3.29	2356	497	5.70	3.39	-5.43E-02	Neutral	-6.55E-05	Neutral	-1.00E-03	Neutral
109	p.Ala109Lys			4243	2332	4.94	3.54	2242	926	5.42	6.32	-1.15E+00	Neutral	-1.01E+00	Neutral	-8.41E-01	Neutral
109	p.Ala109Thr			3577	1765	4.16	2.68	1732	348	4.19	2.38	-7.87E-01	Neutral	-1.98E-04	Neutral	-1.59E-01	Neutral
109	p.Ala109Arg			4277	1773	4.98	2.69	2178	929	5.27	6.35	-7.90E-02	Neutral	-1.36E+00	Neutral	-4.40E-01	Neutral
109	p.Ala109Ser			5242	1956	6.10	2.97	2496	754	6.03	5.15	-6.86E-03	Neutral	-2.73E-02	Neutral	-3.97E-04	Neutral
109	p.Ala109Ile			2094	593	2.44	0.90	1056	155	2.55	1.06	-1.08E-02	Neutral	-7.81E-06	Neutral	-4.05E-05	Neutral
109	p.Ala109Met			4264	1398	4.96	2.12	2308	561	5.58	3.83	-2.48E-03	Neutral	-1.24E-03	Neutral	-4.77E-06	Neutral
109	p.Ala109His			4833	1775	5.62	2.70	2328	605	5.63	4.13	-8.20E-03	Neutral	-3.81E-03	Neutral	-4.97E-05	Neutral
109	p.Ala109Gln			3927	1804	4.57	2.74	1861	497	4.50	3.39	-3.26E-01	Neutral	-1.89E-02	Neutral	-3.56E-02	Neutral
109	p.Ala109Pro		Deleterious	4529	30146	5.27	45.77	2031	3032	4.91	20.71	-5.32E+01	Deleterious	-5.32E+01	Deleterious	-5.32E+01	Deleterious
109	p.Ala109Leu			4138	1033	4.81	1.57	2106	955	5.09	6.52	-1.26E-05	Neutral	-2.15E+00	Neutral	-8.31E-01	Neutral
109	p.Ala109Asp			5728	2678	6.66	4.07	2458	789	5.94	5.39	-1.04E-01	Neutral	-6.53E-02	Neutral	-9.24E-03	Neutral
109	p.Ala109Glu			5017	3315	5.84	5.03	2333	784	5.64	5.35	-2.58E+00	Neutral	-1.38E-01	Neutral	-1.19E+00	Neutral
109	p.Ala109Ala	Synonymous		2870	1370	3.34	2.08	1272	438	3.08	2.99	-1.06E+00	Neutral	-1.06E+00	Neutral	-8.18E-01	Neutral
109	p.Ala109Gly			3264	1198	3.80	1.82	1630	426	3.94	2.91	-5.29E-02	Neutral	-2.60E-02	Neutral	-2.08E-03	Neutral
109	p.Ala109Val			3862	1619	4.49	2.46	1980	602	4.79	4.11	-1.34E-01	Neutral	-8.11E-02	Neutral	-1.46E-02	Neutral
109	p.Ala109Tyr			3063	1160	3.56	1.76	1340	362	3.24	2.47	-1.01E-01	Neutral	-9.30E-02	Neutral	-1.20E-02	Neutral
109	p.Ala109Cys			5733	2308	6.67	3.50	2540	570	6.14	3.89	-1.41E-02	Neutral	-1.41E-04	Neutral	-6.98E-05	Neutral
109	p.Ala109Trp			5488	2705	6.38	4.11	2690	766	6.50	5.23	-2.18E-01	Neutral	-7.76E-03	Neutral	-1.60E-02	Neutral
109	p.Ala109Phe			4731	2761	5.50	4.19	2428	645	5.87	4.41	-1.35E+00	Neutral	-4.35E-03	Neutral	-4.01E-01	Neutral
110	p.Trp110Asn			4679	4750	5.07	5.08					-4.11E-01	Neutral			-4.11E-01	Neutral
110	p.Trp110Lys			4116	4922	4.46	5.27					-1.92E+00	Neutral			-1.92E+00	Neutral
110	p.Trp110Thr			4345	3844	4.71	4.11					-1.31E-01	Neutral			-1.31E-01	Neutral
110	p.Trp110Arg			5145	4792	5.58	5.13					-1.24E-01	Neutral			-1.24E-01	Neutral
110	p.Trp110Ser			4655	5660	5.05	6.06					-1.65E+00	Neutral			-1.65E+00	Neutral
110	p.Trp110Ile			6799	7011	7.37	7.50					-1.38E-01	Neutral			-1.38E-01	Neutral
110	p.Trp110Met			3650	3895	3.96	4.17					-1.18E+00	Neutral			-1.18E+00	Neutral
110	p.Trp110His			2242	2520	2.43	2.70					-4.10E+00	Neutral			-4.10E+00	Neutral
110	p.Trp110Gln			4280	4927	4.64	5.27					-1.38E+00	Neutral			-1.38E+00	Neutral
110	p.Trp110Pro			2564	3394	2.78	3.63					-6.95E+00	Indeterminate			-6.95E+00	Indeterminate
110	p.Trp110Leu			5206	4558	5.64	4.88					-5.62E-02	Neutral			-5.62E-02	Neutral
110	p.Trp110Asp			5506	4245	5.97	4.54					-6.85E-03	Neutral			-6.85E-03	Neutral
110	p.Trp110Glu			4669	5741	5.06	6.14					-1.76E+00	Neutral			-1.76E+00	Neutral
110	p.Trp110Ala			2347	3010	2.54	3.22					-6.88E+00	Indeterminate			-6.88E+00	Indeterminate
110	p.Trp110Gly			4650	4291	5.04	4.59					-1.63E-01	Neutral			-1.63E-01	Neutral
110	p.Trp110Val			8901	8507	9.65	9.10					-1.61E-02	Neutral			-1.61E-02	Neutral
110	p.Trp110Tyr			5931	5740	6.43	6.14					-1.12E-01	Neutral			-1.12E-01	Neutral
110	p.Trp110Cys			4011	3729	4.35	3.99					-2.91E-01	Neutral			-2.91E-01	Neutral
110	p.Trp110Trp	Synonymous		3742	3966	4.06	4.24					-1.06E+00	Neutral			-1.06E+00	Neutral
110	p.Trp110Phe			4803	3940	5.21	4.22					-3.39E-02	Neutral			-3.39E-02	Neutral
111	p.Gly111Asn			6372	1964	5.15	2.01					-1.98E-01	Neutral			-1.98E-01	Neutral
111	p.Gly111Lys			6688	3205	5.41	3.28					-4.17E+00	Neutral			-4.17E+00	Neutral
111	p.Gly111Thr			3788	3224	3.06	3.30					-4.65E+01	Indeterminate			-3.32E+01	Indeterminate
111	p.Gly111Arg			6851	2649	5.54	2.71					-1.14E+00	Neutral			-1.14E+00	Neutral
111	p.Gly111Ser		Neutral	6085	1922	4.92	1.97					-2.95E-01	Neutral			-2.95E-01	Neutral
111	p.Gly111Ile			6288	9894	5.08	10.12					-5.32E+01	Deleterious			-5.32E+01	Deleterious
111	p.Gly111Met			5548	1895	4.49	1.94					-7.64E-01	Neutral			-7.64E-01	Neutral
111	p.Gly111His			7031	3824	5.68	3.91					-6.80E+00	Indeterminate			-6.80E+00	Indeterminate
111	p.Gly111Gln			6371	1696	5.15	1.73					-3.39E-02	Neutral			-3.39E-02	Neutral
111	p.Gly111Pro			3949	35782	3.19	36.59					-5.32E+01	Deleterious			-5.32E+01	Deleterious
111	p.Gly111Leu			4980	1649	4.03	1.69					-7.94E-01	Neutral			-7.94E-01	Neutral
111	p.Gly111Asp			5458	1653	4.41	1.69					-2.79E-01	Neutral			-2.79E-01	Neutral
111	p.Gly111Glu			8722	3773	7.05	3.86					-1.42E+00	Neutral			-1.42E+00	Neutral
111	p.Gly111Ala			4049	1655	3.27	1.69					-4.33E+00	Neutral			-4.33E+00	Neutral
111	p.Gly111Gly	Synonymous		5356	1889	4.33	1.93					-1.06E+00	Neutral			-1.06E+00	Neutral
111	p.Gly111Val			8116	10825	6.56	11.07					-5.32E+01	Deleterious			-5.32E+01	Deleterious
111	p.Gly111Tyr			5450	1959	4.41	2.00					-1.17E+00	Neutral			-1.17E+00	Neutral
111	p.Gly111Cys			8487	3356	6.86	3.43					-7.96E-01	Neutral			-7.96E-01	Neutral

111	p.Gly111Trp			7849	2880	6.35	2.95					-5.35E-01	Neutral			-5.35E-01	Neutral
111	p.Gly111Phe			6259	2098	5.06	2.15					-4.69E-01	Neutral			-4.69E-01	Neutral
112	p.Arg112Asn			1991	616	5.55	1.56					-2.08E-01	Neutral			-2.08E-01	Neutral
112	p.Arg112Lys			1846	746	5.14	1.89					-2.10E+00	Neutral			-2.10E+00	Neutral
112	p.Arg112Thr			1776	536	4.95	1.36					-2.38E-01	Neutral			-2.38E-01	Neutral
112	p.Arg112Arg	Synonymous		1452	488	4.05	1.24					-1.06E+00	Neutral			-1.06E+00	Neutral
112	p.Arg112Ser			1978	644	5.51	1.63					-3.51E-01	Neutral			-3.51E-01	Neutral
112	p.Arg112Ile			2053	798	5.72	2.02					-1.31E+00	Neutral			-1.31E+00	Neutral
112	p.Arg112Met			1638	361	4.56	0.91					-5.32E-03	Neutral			-5.32E-03	Neutral
112	p.Arg112His			2167	850	6.04	2.15					-1.24E+00	Neutral			-1.24E+00	Neutral
112	p.Arg112Gln			2010	873	5.60	2.21					-2.70E+00	Neutral			-2.70E+00	Neutral
112	p.Arg112Pro			1820	24747	5.07	62.68					-5.32E+01	Deleterious			-5.32E+01	Deleterious
112	p.Arg112Leu			1796	1126	5.00	2.85					-1.45E+01	Indeterminate			-1.45E+01	Indeterminate
112	p.Arg112Asp			1639	814	4.57	2.06					-7.02E+00	Indeterminate			-7.02E+00	Indeterminate
112	p.Arg112Glu			1519	919	4.23	2.33					-1.55E+01	Indeterminate			-1.55E+01	Indeterminate
112	p.Arg112Ala			1691	765	4.71	1.94					-4.46E+00	Neutral			-4.46E+00	Neutral
112	p.Arg112Gly	Pathogenic		1652	1294	4.60	3.28					-2.95E+01	Indeterminate			-2.94E+01	Indeterminate
112	p.Arg112Val			1610	761	4.49	1.93					-5.85E+00	Indeterminate			-5.85E+00	Indeterminate
112	p.Arg112Tyr			2411	944	6.72	2.39					-9.47E-01	Neutral			-9.47E-01	Neutral
112	p.Arg112Cys			1023	549	2.85	1.39					-1.60E+01	Indeterminate			-1.60E+01	Indeterminate
112	p.Arg112Trp			1893	729	5.27	1.85					-1.48E+00	Neutral			-1.48E+00	Neutral
112	p.Arg112Phe			1931	923	5.38	2.34					-4.69E+00	Neutral			-4.69E+00	Neutral
113	p.Leu113Asn			5379	2633	3.76	3.88	4836	1048	3.79	4.54	-5.05E+00	Neutral	-6.01E+00	Neutral	-7.95E+00	Indeterminate
113	p.Leu113Lys			10008	4687	7.00	6.90	9167	1337	7.19	5.79	-1.25E+00	Neutral	-6.04E-02	Neutral	-3.79E-01	Neutral
113	p.Leu113Thr			7489	4108	5.24	6.05	7289	1294	5.72	5.61	-5.15E+00	Neutral	-9.00E-01	Neutral	-3.67E+00	Neutral
113	p.Leu113Arg			5302	2864	3.71	4.22	5345	800	4.19	3.47	-7.81E+00	Indeterminate	-5.23E-01	Neutral	-5.57E+00	Neutral
113	p.Leu113Ser			9675	4603	6.77	6.78	9003	1272	7.06	5.51	-1.50E+00	Neutral	-4.32E-02	Neutral	-4.94E-01	Neutral
113	p.Leu113Ile			7432	3761	5.20	5.54	6762	832	5.31	3.61	-3.54E+00	Neutral	-2.39E-02	Neutral	-1.77E+00	Neutral
113	p.Leu113Met			7071	2518	4.95	3.71	5559	1368	4.36	5.93	-3.49E-01	Neutral	-8.34E+00	Indeterminate	-5.88E+00	Indeterminate
113	p.Leu113His			5034	2915	3.52	4.29	4779	1071	3.75	4.64	-1.08E+01	Indeterminate	-7.03E+00	Indeterminate	-1.41E+01	Indeterminate
113	p.Leu113Gln			6683	3104	4.67	4.57	6294	360	4.94	1.56	-2.72E+00	Neutral	-2.58E-09	Neutral	-1.19E+00	Neutral
113	p.Leu113Pro			2367	1619	1.66	2.38	2507	642	1.97	2.78	-3.57E+01	Indeterminate	-2.24E+01	Indeterminate	-5.32E+01	Deleterious
113	p.Leu113Leu	Synonymous		8768	3839	6.13	5.65	7778	1443	6.10	6.25	-1.06E+00	Neutral	-1.06E+00	Neutral	-8.18E-01	Neutral
113	p.Leu113Asp			8948	4600	6.26	6.78	8096	1453	6.35	6.30	-2.78E+00	Neutral	-7.48E-01	Neutral	-1.75E+00	Neutral
113	p.Leu113Glu			9912	4090	6.93	6.02	7870	1492	6.18	6.46	-4.80E-01	Neutral	-1.20E+00	Neutral	-5.68E-01	Neutral
113	p.Leu113Ala			4998	2415	3.50	3.56	4479	1363	3.51	5.91	-5.32E+00	Neutral	-2.11E+01	Indeterminate	-2.22E+01	Indeterminate
113	p.Leu113Gly			8660	4353	6.06	6.41	7215	1802	5.66	7.81	-2.61E+00	Neutral	-6.25E+00	Neutral	-6.02E+00	Indeterminate
113	p.Leu113Val			10162	4313	7.11	6.35	9204	1432	7.22	6.20	-5.69E-01	Neutral	-1.29E-01	Neutral	-1.29E-01	Neutral
113	p.Leu113Tyr			4520	2034	3.16	3.00	3746	470	2.94	2.04	-4.48E+00	Neutral	-3.07E-01	Neutral	-2.67E+00	Neutral
113	p.Leu113Cys			7692	3300	5.38	4.86	6668	1154	5.23	5.00	-1.25E+00	Neutral	-9.28E-01	Neutral	-8.50E-01	Neutral
113	p.Leu113Trp			8114	3571	5.67	5.26	6388	1407	5.01	6.10	-1.32E+00	Neutral	-4.26E+00	Neutral	-3.29E+00	Neutral
113	p.Leu113Phe			4770	2565	3.34	3.78	4461	1039	3.50	4.50	-8.78E+00	Indeterminate	-8.93E+00	Indeterminate	-1.40E+01	Indeterminate
114	p.Pro114Asn			1817	3514	6.12	6.50					-5.32E+01	Deleterious			-5.32E+01	Deleterious
114	p.Pro114Lys			995	2390	3.35	4.42					-5.32E+01	Deleterious			-5.32E+01	Deleterious
114	p.Pro114Thr	Likely pathogenic		936	1298	3.15	2.40					-5.32E+01	Deleterious			-5.32E+01	Deleterious
114	p.Pro114Arg			1780	3499	6.00	6.47					-5.32E+01	Deleterious			-5.32E+01	Deleterious
114	p.Pro114Ser			1360	987	4.58	1.83					-9.07E+00	Indeterminate			-9.07E+00	Indeterminate
114	p.Pro114Ile			1197	2618	4.03	4.84					-5.32E+01	Deleterious			-5.32E+01	Deleterious
114	p.Pro114Met			1129	2500	3.81	4.62					-5.32E+01	Deleterious			-5.32E+01	Deleterious
114	p.Pro114His	Likely pathogenic		1368	3436	4.61	6.35					-5.32E+01	Deleterious			-5.32E+01	Deleterious
114	p.Pro114Gln			1806	4048	6.09	7.49					-5.32E+01	Deleterious			-5.32E+01	Deleterious
114	p.Pro114Pro	Synonymous		970	415	3.27	0.77					-1.06E+00	Neutral			-1.06E+00	Neutral
114	p.Pro114Leu		Deleterious	1586	3408	5.35	6.30					-5.32E+01	Deleterious			-5.32E+01	Deleterious
114	p.Pro114Asp			1846	3955	6.22	7.31					-5.32E+01	Deleterious			-5.32E+01	Deleterious
114	p.Pro114Glu			1828	3725	6.16	6.89					-5.32E+01	Deleterious			-5.32E+01	Deleterious
114	p.Pro114Ala			394	187	1.33	0.35					-8.63E+00	Indeterminate			-8.63E+00	Indeterminate
114	p.Pro114Gly			1358	1887	4.58	3.49					-5.32E+01	Deleterious			-5.32E+01	Deleterious
114	p.Pro114Val			1936	2488	6.53	4.60					-3.48E+01	Indeterminate			-3.28E+01	Indeterminate
114	p.Pro114Tyr			1770	4188	5.97	7.74					-5.32E+01	Deleterious			-5.32E+01	Deleterious
114	p.Pro114Cys			1321	903	4.45	1.67					-7.47E+00	Indeterminate			-7.47E+00	Indeterminate
114	p.Pro114Trp			2579	5476	8.69	10.13					-5.32E+01	Deleterious			-5.32E+01	Deleterious
114	p.Pro114Phe			1693	3153	5.71	5.83					-5.32E+01	Deleterious			-5.32E+01	Deleterious
115	p.Val115Asn			6328	3214	5.11	4.25					-2.41E+00	Neutral			-2.41E+00	Neutral
115	p.Val115Lys			5786	2973	4.67	3.93					-3.03E+00	Neutral			-3.03E+00	Neutral
115	p.Val115Thr			6751	3265	5.45	4.31					-1.57E+00	Neutral			-1.57E+00	Neutral
115	p.Val115Arg			3621	2684	2.92	3.55					-2.19E+01	Indeterminate			-2.19E+01	Indeterminate
115	p.Val115Ser			5887	3318	4.75	4.38					-4.66E+00	Neutral			-4.66E+00	Neutral
115	p.Val115Ile			4855	2279	3.92	3.01					-2.54E+00	Neutral			-2.54E+00	Neutral
115	p.Val115Met			7340	3600	5.92	4.76					-1.44E+00	Neutral			-1.44E+00	Neutral
115	p.Val115His			4929	2890	3.98	3.82					-7.13E+00	Indeterminate			-7.13E+00	Indeterminate
115	p.Val115Gln			4345	3257	3.51	4.30					-1.91E+01	Indeterminate			-1.91E+01	Indeterminate
115	p.Val115Pro			9037	11154	7.29	14.74					-3.72E+01	Indeterminate			-3.31E+01	Indeterminate
115	p.Val115Leu			9713	5878	7.84	7.77					-2.89E+00	Neutral			-2.89E+00	Neutral
115	p.Val115Asp			5570	3650	4.50	4.82					-9.37E+00	Indeterminate			-9.37E+00	Indeterminate
115	p.Val115Glu			4451	2694	3.59	3.56					-9.20E+00	Indeterminate			-9.20E+00	Indeterminate
115	p.Val115Ala			3262	2371	2.63	3.13					-2.29E+01	Indeterminate			-2.29E+01	Indeterminate
115	p.Val115Gly			9239	5617	7.46	7.42					-3.24E+00	Neutral			-3.24E+00	Neutral
115	p.Val115Val	Synonymous		8999	4521	7.26	5.97					-1.06E+00	Neutral			-1.06E+00	Neutral
115	p.Val115Tyr			5619	2709	4.54	3.58					-2.24E+00	Neutral			-2.24E+00	Neutral
115	p.Val115Cys			4727	2228	3.82	2.94					-2.73E+00	Neutral			-2.73E+00	Neutral
115	p.Val115Trp			6028	3240	4.87	4.28					-3.56E+00	Neutral			-3.56E+00	Neutral
115	p.Val115Phe			7402	4130	5.97	5.46					-3.04E+00	Neutral			-3.04E+00	Neutral
116	p.Asp116Asn			4943	1914	2.89	2.39					-3.49E+00	Neutral			-3.49E+00	Neutral
116	p.Asp116Lys			12435	4890	7.27	6.11					-5.04E-01	Neutral			-5.04E-01	Neutral
116	p.Asp116Thr			6247	2920	3.65	3.65					-5.85E+00	Indeterminate			-5.85E+00	Indeterminate
116	p.Asp116Arg			7079	3300	4.14	4.12					-4.81E+00	Neutral			-4.81E+00	Neutral
116	p.Asp116Ser			10328	4273	6.04	5.34					-1.21E+00	Neutral			-1.21E+00	Neutral
116	p.Asp116Ile			8211	3857	4.80	4.82					-3.94E+00	Neutral			-3.94E+00	Neutral
116	p.Asp116Met			7955	2576	4.65	3.22					-3.32E-01	Neutral			-3.32E-01	Neutral
116	p.Asp116His			10717	4466	6.26	5.58					-1.16E+00	Neutral			-1.16E+00	Neutral
116	p.Asp116Gln			8325	3445	4.87	4.30					-1.92E+00	Neutral			-1.92E+00	Neutral
116	p.Asp116Pro			4065	7636	2.38	9.54					-5.32E+01	Deleterious			-5.32E+01	Deleterious
116	p.Asp116Leu			7761	3355	4.54	4.19					-2.83E+00	Neutral			-2.83E+00	Neutral
116	p.Asp116Asp	Synonymous		9567	3790	5.59	4.73					-1.06E+00	Neutral			-1.06E+00	Neutral
116	p.Asp116Glu			10329	4515	6.04	5.64					-1.73E+00	Neutral			-1.73E+00	Neutral
116	p.Asp116Ala			6842	2732	4.00	3.41					-2.29E+00	Neutral			-2.29E+00	Neutral
116	p.Asp116Gly			10318	6029	6.03	7.53					-7.40E+00	Indeterminate			-7.40E+00	Indeterminate
116	p.Asp116Val			8773	3898	5.13	4.87					-2.63E+00	Neutral			-2.63E+00	Neutral
116	p.Asp116Tyr			7958	2869	4.65	3.58					-8.30E-01	Neutral			-8.30E-01	Neutral
116	p.Asp116Cys			12620	5342	7.38	6.67					-8.78E-01	Neutral			-8.78E-01	Neutral
116	p.Asp116Trp			7328	3954	4.28	4.94					-8.45E+00	Indeterminate			-8.45E+00	Indeterminate
116	p.Asp116Phe			9303	4319	5.44	5.39					-3.01E+00	Neutral			-3.01E+00	Neutral
117	p.Leu117Asn			7070	2516	4.56	4.40					-1.87E+00	Neutral			-1.87E+00	Neutral
117	p.Leu117Lys			9877	3587	6.37	6.28					-1.02E+00	Neutral			-1.02E+00	Neutral
117	p.Leu117Thr			7864	2594	5.07	4.54					-8.74E-01	Neutral			-8.74E-01	Neutral
117	p.Leu117Arg			8872	3342	5.72	5.85					-1.66E+00	Neutral			-1.66E+00	Neutral
117	p.Leu117Ser			8065	2946	5.20	5.15					-1.67E+00	Neutral			-1.67E+00	Neutral
117	p.Leu117Ile			8208	2847	5.30	4.98					-1.14E+00	Neutral			-1.14E+00	Neutral
117	p.Leu117Met			6340	2230	4.09	3.90					-2.16E+00	Neutral			-2.16E+00	Neutral
117	p.Leu117His			6546	1898	4.22	3.32					-5.04E-01	Neutral			-5.04E-01	Neutral
117	p.Leu117Gln			7223	2954	4.66	5.17					-3.86E+00	Neutral			-3.86E+00	Neutral
117	p.Leu117Pro			5349	4175	3.45	7.30					-4.32E+01	Indeterminate			-3.32E+01	Indeterminate
117	p.Leu117Leu	Synonymous		8344	2882	5.38	5.04					-1.06E+00	Neutral			-1.06E+00	Neutral
117	p.Leu117Asp			8914	3001	5.75	5.25					-7.42E-01	Neutral			-7.42E-01	Neutral
117	p.Leu117Glu			9669	3670	6.24	6.42					-1.45E+00	Neutral			-1.45E+00	Neutral
117	p.Leu117Ala			4021	1315	2.59	2.30					-3.34E+00	Neutral			-3.34E+00	Neutral
117	p.Leu117Gly			8262	3050	5.33	5.34					-1.70E+00	Neutral			-1.70E+00	Neutral
117	p.Leu117Val			9084	3239	5.86	5.67					-1.09E+00	Neutral			-1.09E+00	Neutral
117	p.Leu117Tyr			9820	3189	6.34	5.58					-4.17E-01	Neutral			-4.17E-01	Neutral
117	p.Leu117Cys			8025	3045	5.18	5.33					-2.13E+00	Neutral			-2.13E+00	Neutral
117	p.Leu117Trp			5931	1964	3.83	3.44					-1.72E+00	Neutral			-1.72E+00	Neutral
117	p.Leu117Phe			7491	2716	4.83	4.75					-1.86E+00	Neutral			-1.86E+00	Neutral
118	p.Ala118Asn			5316	3171	4.50	5.33					-3.28E+01	Indeterminate			-3.20E+01	Indeterminate
118	p.Ala118Lys			5355	3669	4.53	6.16					-4.51E+01	Indeterminate			-3.32E+01	Indeterminate
118	p.Ala118Thr			7459	2789	6.31	4.69					-4.89E+00	Neutral			-4.89E+00	Neutral
118	p.Ala118Arg			8551	5560	7.24	9.34					-2.74E+01	Indeterminate			-2.74E+01	Indeterminate
118	p.Ala118Ser			4903	1694	4.15	2.85					-6.37E+00	Indeterminate			-6.37E+00	Indeterminate
118	p.Ala118Ile			7141	3330	6.05	5.59					-1.23E+01	Indeterminate			-1.23E+01	Indeterminate
118	p.Ala118Met			4319	2202	3.66	3.70					-2.63E+01	Indeterminate			-2.63E+01	Indeterminate
118	p.Ala118His			4487	2865	3.80	4.81					-4.39E+01	Indeterminate			-3.32E+01	Indeterminate
118	p.Ala118Gln			5718	3355	4.84	5.64					-2.97E+01	Indeterminate			-2.96E+01	Indeterminate
118	p.Ala118Pro			2401	1145	2.03	1.92					-3.62E+01	Indeterminate			-3.30E+01	Indeterminate
118	p.Ala118Leu			6468	2765	5.48	4.64					-1.02E+01	Indeterminate			-1.02E+01	Indeterminate
118	p.Ala118Asp			7364	4617	6.23	7.76					-2.84E+01	Indeterminate			-2.83E+01	Indeterminate
118	p.Ala118Glu			6132	3782	5.19	6.35					-3.17E+01	Indeterminate			-3.13E+01	Indeterminate
118	p.Ala118Ala	Synonymous		7922	2315	6.71	3.89					-1.06E+00	Neutral			-1.06E+00	Neutral
118	p.Ala118Gly			4629	1330	3.92	2.23					-2.90E+00	Neutral			-2.90E+00	Neutral
118	p.Ala118Val			6165	2323	5.22	3.90					-6.66E+00	Indeterminate			-6.66E+00	Indeterminate
118	p.Ala118Tyr			4780	2825	4.05	4.75					-3.50E+01	Indeterminate			-3.28E+01	Indeterminate

118	p.Ala118Cys			7840	2535	6.64	4.26					-2.10E+00	Neutral			-2.10E+00	Neutral
118	p.Ala118Trp			5510	3866	4.66	6.49					-4.65E+01	Indeterminate			-3.32E+01	Indeterminate
118	p.Ala118Phe			5657	3391	4.79	5.70					-3.16E+01	Indeterminate			-3.12E+01	Indeterminate
119	p.Glu119Asn			4365	494	4.22	3.26					-1.46E+00	Neutral			-1.46E+00	Neutral
119	p.Glu119Lys			4474	419	4.33	2.77					-3.00E-01	Neutral			-3.00E-01	Neutral
119	p.Glu119Thr			6227	566	6.02	3.74					-6.40E-02	Neutral			-6.40E-02	Neutral
119	p.Glu119Arg			3977	649	3.85	4.29					-1.00E+01	Indeterminate			-1.00E+01	Indeterminate
119	p.Glu119Ser			5648	290	5.46	1.92					-3.20E-06	Neutral			-3.20E-06	Neutral
119	p.Glu119Ile			4576	367	4.42	2.42					-4.77E-02	Neutral			-4.77E-02	Neutral
119	p.Glu119Met			6932	1140	6.70	7.53					-4.89E+00	Neutral			-4.89E+00	Neutral
119	p.Glu119His			5341	1407	5.16	9.29					-3.11E+01	Indeterminate			-3.08E+01	Indeterminate
119	p.Glu119Gln			4684	1065	4.53	7.03					-2.38E+01	Indeterminate			-2.38E+01	Indeterminate
119	p.Glu119Pro			4894	1402	4.73	9.26					-4.08E+01	Indeterminate			-3.32E+01	Indeterminate
119	p.Glu119Leu			3932	414	3.80	2.73					-1.13E+00	Neutral			-1.13E+00	Neutral
119	p.Glu119Asp			4939	865	4.78	5.71					-9.98E+00	Indeterminate			-9.98E+00	Indeterminate
119	p.Glu119Glu	Synonymous		7139	908	6.90	6.00					-1.06E+00	Neutral			-1.06E+00	Neutral
119	p.Glu119Ala			3939	268	3.81	1.77					-9.16E-03	Neutral			-9.16E-03	Neutral
119	p.Glu119Gly			6152	699	5.95	4.62					-6.53E-01	Neutral			-6.53E-01	Neutral
119	p.Glu119Val			5805	939	5.61	6.20					-5.91E+00	Indeterminate			-5.91E+00	Indeterminate
119	p.Glu119Tyr			4931	262	4.77	1.73					-1.97E-05	Neutral			-1.97E-05	Neutral
119	p.Glu119Cys			5729	354	5.54	2.34					-1.70E-04	Neutral			-1.70E-04	Neutral
119	p.Glu119Trp			5340	1418	5.16	9.37					-3.18E+01	Indeterminate			-3.13E+01	Indeterminate
119	p.Glu119Phe			4406	1213	4.26	8.01					-4.03E+01	Indeterminate			-3.32E+01	Indeterminate
120	p.Glu120Asn			6668	1715	6.69	7.09	5879	924	6.09	6.77	-2.64E+00	Neutral	-9.94E-01	Neutral	-1.82E+00	Neutral
120	p.Glu120Lys			5140	1178	5.16	4.87	4764	741	4.94	5.43	-2.24E+00	Neutral	-1.50E+00	Neutral	-1.90E+00	Neutral
120	p.Glu120Thr			4944	1177	4.96	4.87	5718	665	5.93	4.87	-2.98E+00	Neutral	-5.61E-02	Neutral	-1.40E+00	Neutral
120	p.Glu120Arg			3381	806	3.39	3.33	3318	513	3.44	3.76	-5.53E+00	Neutral	-2.95E+00	Neutral	-5.70E+00	Neutral
120	p.Glu120Ser			4204	949	4.22	3.93	3955	492	4.10	3.60	-2.99E+00	Neutral	-4.35E-01	Neutral	-1.67E+00	Neutral
120	p.Glu120Ile			5170	1508	5.19	6.24	5138	717	5.32	5.25	-7.14E+00	Indeterminate	-5.55E-01	Neutral	-5.03E+00	Neutral
120	p.Glu120Met			3518	1081	3.53	4.47	3493	547	3.62	4.01	-1.37E+01	Indeterminate	-2.88E+00	Neutral	-1.29E+01	Indeterminate
120	p.Glu120His			4893	1157	4.91	4.79	4467	725	4.63	5.31	-2.93E+00	Neutral	-2.23E+00	Neutral	-2.96E+00	Neutral
120	p.Glu120Gln			5102	1362	5.12	5.63	4390	675	4.55	4.95	-4.98E+00	Neutral	-1.66E+00	Neutral	-4.15E+00	Neutral
120	p.Glu120Pro			3878	1004	3.89	4.15	4187	558	4.34	4.09	-6.48E+00	Indeterminate	-6.65E-01	Neutral	-4.57E+00	Neutral
120	p.Glu120Leu			4277	850	4.29	3.52	3887	565	4.03	4.14	-1.35E+00	Neutral	-1.50E+00	Neutral	-1.27E+00	Neutral
120	p.Glu120Asp			5594	1357	5.61	5.61	5098	821	5.28	6.02	-2.64E+00	Neutral	-1.62E+00	Neutral	-2.28E+00	Neutral
120	p.Glu120Glu	Synonymous		5994	1288	6.01	5.33	5329	818	5.52	5.99	-1.06E+00	Neutral	-1.06E+00	Neutral	-8.18E-01	Neutral
120	p.Glu120Ala			4804	1061	4.82	4.39	5170	515	5.36	3.77	-2.06E+00	Neutral	-1.00E-02	Neutral	-7.85E-01	Neutral
120	p.Glu120Gly			4006	926	4.02	3.83	4433	514	4.59	3.77	-3.67E+00	Neutral	-1.48E-01	Neutral	-1.95E+00	Neutral
120	p.Glu120Val			5499	1316	5.52	5.44	5200	764	5.39	5.60	-2.53E+00	Neutral	-8.14E-01	Neutral	-1.61E+00	Neutral
120	p.Glu120Tyr			2339	529	2.35	2.19	2119	350	2.20	2.56	-7.40E+00	Indeterminate	-7.88E+00	Indeterminate	-1.18E+01	Indeterminate
120	p.Glu120Cys			6757	1679	6.78	6.94	6708	1017	6.95	7.45	-2.12E+00	Neutral	-5.29E-01	Neutral	-1.14E+00	Neutral
120	p.Glu120Trp			5516	1474	5.53	6.10	5475	719	5.67	5.27	-4.44E+00	Neutral	-2.62E-01	Neutral	-2.61E+00	Neutral
120	p.Glu120Phe			7976	1759	8.00	7.28	7777	1009	8.06	7.39	-6.25E-01	Neutral	-6.15E-02	Neutral	-1.25E-01	Neutral
121	p.Leu121Asn			5557	2222	3.85	4.08					-4.57E+00	Neutral			-4.57E+00	Neutral
121	p.Leu121Lys			5913	2024	4.10	3.72					-1.77E+00	Neutral			-1.77E+00	Neutral
121	p.Leu121Thr			7287	2639	5.05	4.85					-1.63E+00	Neutral			-1.63E+00	Neutral
121	p.Leu121Arg			6565	3538	4.55	6.50					-1.20E+01	Indeterminate			-1.20E+01	Indeterminate
121	p.Leu121Ser			4991	1747	3.46	3.21					-2.80E+00	Neutral			-2.80E+00	Neutral
121	p.Leu121Ile			6708	2326	4.65	4.27					-1.47E+00	Neutral			-1.47E+00	Neutral
121	p.Leu121Met			7054	9643	4.89	17.72					-5.32E+01	Deleterious			-5.32E+01	Deleterious
121	p.Leu121His			6679	1883	4.63	3.46					-2.79E-01	Neutral			-2.79E-01	Neutral
121	p.Leu121Gln			6870	2290	4.76	4.21					-1.06E+00	Neutral			-1.06E+00	Neutral
121	p.Leu121Pro			6118	1809	4.24	3.32					-5.61E-01	Neutral			-5.61E-01	Neutral
121	p.Leu121Leu	Synonymous		5345	1640	3.71	3.01					-1.06E+00	Neutral			-1.06E+00	Neutral
121	p.Leu121Asp			20642	4114	14.32	7.56					-9.42E-07	Neutral			-9.42E-07	Neutral
121	p.Leu121Glu			8089	3520	5.61	6.47					-3.77E+00	Neutral			-3.77E+00	Neutral
121	p.Leu121Ala			5113	1790	3.55	3.29					-2.68E+00	Neutral			-2.68E+00	Neutral
121	p.Leu121Gly			7464	3126	5.18	5.74					-3.55E+00	Neutral			-3.55E+00	Neutral
121	p.Leu121Val			8431	2171	5.85	3.99					-4.01E-02	Neutral			-4.01E-02	Neutral
121	p.Leu121Tyr			4665	1066	3.24	1.96					-1.05E-01	Neutral			-1.05E-01	Neutral
121	p.Leu121Cys			6774	1898	4.70	3.49					-2.50E-01	Neutral			-2.50E-01	Neutral
121	p.Leu121Trp			7344	2981	5.09	5.48					-3.11E+00	Neutral			-3.11E+00	Neutral
121	p.Leu121Phe			6583	1996	4.57	3.67					-5.65E-01	Neutral			-5.65E-01	Neutral
122	p.Gly122Asn			7569	3399	4.56	3.94	11922	382	4.65	2.73	-4.61E-01	Neutral	-1.76E-05	Neutral	-6.09E-02	Neutral
122	p.Gly122Lys			8834	4858	5.33	5.63	14027	527	5.47	3.77	-1.46E+00	Neutral	-1.66E-04	Neutral	-4.53E-01	Neutral
122	p.Gly122Thr			9750	4942	5.88	5.73	14935	744	5.83	5.32	-6.31E-01	Neutral	-1.67E-02	Neutral	-1.13E-01	Neutral
122	p.Gly122Arg			9626	6094	5.80	7.07	13461	1005	5.25	7.18	-2.86E+00	Neutral	-1.50E+00	Neutral	-2.36E+00	Neutral
122	p.Gly122Ser			10109	4589	6.09	5.32	13433	666	5.24	4.76	-2.03E-01	Neutral	-2.60E-02	Neutral	-1.65E-02	Neutral
122	p.Gly122Ile			5859	3683	3.53	4.27	11299	488	4.41	3.49	-6.10E+00	Indeterminate	-7.76E-03	Neutral	-3.72E+00	Neutral
122	p.Gly122Met			8522	3997	5.14	4.63	12277	772	4.79	5.52	-4.78E-01	Neutral	-5.21E-01	Neutral	-2.40E-01	Neutral
122	p.Gly122His			8831	4052	5.32	4.70	13991	782	5.46	5.59	-3.52E-01	Neutral	-1.02E-01	Neutral	-5.93E-02	Neutral
122	p.Gly122Gln			9295	4626	5.60	5.36	13146	527	5.13	3.77	-6.19E-01	Neutral	-8.97E-04	Neutral	-1.04E-01	Neutral
122	p.Gly122Pro			7279	3497	4.39	4.05	11943	942	4.66	6.73	-8.88E-01	Neutral	-2.66E+00	Neutral	-1.75E+00	Neutral
122	p.Gly122Leu			6446	3447	3.89	4.00	10134	422	3.95	3.02	-2.35E+00	Neutral	-7.71E-03	Neutral	-9.59E-01	Neutral
122	p.Gly122Asp			8712	4580	5.25	5.31	14156	564	5.52	4.03	-1.11E+00	Neutral	-5.01E-04	Neutral	-2.87E-01	Neutral
122	p.Gly122Glu			9974	5662	6.01	6.56	15722	654	6.13	4.67	-1.38E+00	Neutral	-5.87E-04	Neutral	-4.14E-01	Neutral
122	p.Gly122Ala			8715	4648	5.25	5.39	14349	673	5.60	4.81	-1.23E+00	Neutral	-8.15E-03	Neutral	-3.43E-01	Neutral
122	p.Gly122Gly	Synonymous		6971	3382	4.20	3.92	9670	615	3.77	4.40	-1.06E+00	Neutral	-1.06E+00	Neutral	-8.18E-01	Neutral
122	p.Gly122Val		Neutral	7691	3268	4.64	3.79	12215	711	4.77	5.08	-2.61E-01	Neutral	-2.59E-01	Neutral	-7.59E-02	Neutral
122	p.Gly122Tyr			9213	4716	5.55	5.47	14745	1063	5.75	7.60	-7.93E-01	Neutral	-9.53E-01	Neutral	-6.02E-01	Neutral
122	p.Gly122Cys			6227	4023	3.75	4.66	9379	574	3.66	4.10	-6.29E+00	Indeterminate	-8.61E-01	Neutral	-4.58E+00	Neutral
122	p.Gly122Trp			6552	2705	3.95	3.14	8809	789	3.44	5.64	-3.31E-01	Neutral	-7.69E+00	Indeterminate	-5.31E+00	Neutral
122	p.Gly122Phe			9687	6084	5.84	7.05	16655	1092	6.50	7.80	-2.71E+00	Neutral	-3.03E-01	Neutral	-1.39E+00	Neutral
123	p.His123Asn			7749	1318	5.54	5.67	4136	391	5.18	4.24	-6.16E+00	Indeterminate	-2.67E+00	Neutral	-6.00E+00	Indeterminate
123	p.His123Lys			8216	869	5.87	3.74	4134	343	5.17	3.72	-2.36E-01	Neutral	-1.19E+00	Neutral	-4.32E-01	Neutral
123	p.His123Thr			5633	1158	4.03	4.98	3132	397	3.92	4.31	-1.76E+01	Indeterminate	-1.36E+01	Indeterminate	-2.66E+01	Indeterminate
123	p.His123Arg			7158	1021	5.12	4.39	3689	331	4.62	3.59	-3.03E+00	Neutral	-2.46E+00	Neutral	-3.23E+00	Neutral
123	p.His123Ser			6242	951	4.46	4.09	3377	270	4.23	2.93	-5.21E+00	Neutral	-1.45E+00	Neutral	-4.18E+00	Neutral
123	p.His123Ile			6667	1082	4.76	4.65	3468	304	4.34	3.30	-6.24E+00	Indeterminate	-2.42E+00	Neutral	-5.85E+00	Indeterminate
123	p.His123Met			7540	1467	5.39	6.31	4059	452	5.08	4.91	-1.07E+01	Indeterminate	-6.08E+00	Neutral	-1.31E+01	Indeterminate
123	p.His123His	Synonymous		8307	1051	5.94	4.52	4588	388	5.74	4.21	-1.06E+00	Neutral	-1.06E+00	Neutral	-8.18E-01	Neutral
123	p.His123Gln	Likely benign		7177	959	5.13	4.12	4451	353	5.57	3.83	-2.09E+00	Neutral	-7.04E-01	Neutral	-1.24E+00	Neutral
123	p.His123Pro			4158	1844	2.97	7.93	2773	1007	3.47	10.93	-5.32E+01	Deleterious	-5.32E+01	Deleterious	-5.32E+01	Deleterious
123	p.His123Leu			6904	1120	4.93	4.82	4153	458	5.20	4.97	-5.93E+00	Indeterminate	-5.64E+00	Neutral	-8.40E+00	Indeterminate
123	p.His123Asp			7108	1408	5.08	6.05	4396	588	5.50	6.38	-1.22E+01	Indeterminate	-1.11E+01	Indeterminate	-1.92E+01	Indeterminate
123	p.His123Glu			8263	1312	5.91	5.64	4672	718	5.85	7.79	-4.10E+00	Neutral	-1.64E+01	Indeterminate	-1.65E+01	Indeterminate
123	p.His123Ala			5540	763	3.96	3.28	3137	305	3.93	3.31	-3.91E+00	Neutral	-4.87E+00	Neutral	-5.96E+00	Indeterminate
123	p.His123Gly			4257	992	3.04	4.27	2165	292	2.71	3.17	-3.18E+01	Indeterminate	-2.35E+01	Indeterminate	-5.32E+01	Deleterious
123	p.His123Val			7367	1424	5.26	6.12	4585	769	5.74	8.35	-1.08E+01	Indeterminate	-2.15E+01	Indeterminate	-2.78E+01	Indeterminate
123	p.His123Tyr			6410	817	4.58	3.51	3853	265	4.82	2.88	-1.97E+00	Neutral	-3.07E-01	Neutral	-9.09E-01	Neutral
123	p.His123Cys			10430	1689	7.45	7.26	5920	478	7.41	5.19	-3.06E+00	Neutral	-3.63E-01	Neutral	-1.67E+00	Neutral
123	p.His123Trp			9243	1217	6.61	5.23	5457	491	6.83	5.33	-1.10E+00	Neutral	-1.10E+00	Neutral	-8.58E-01	Neutral
123	p.His123Phe			5563	793	3.98	3.41	3757	612	4.70	6.64	-4.58E+00	Neutral	-2.39E+01	Indeterminate	-2.41E+01	Indeterminate
124	p.Arg124Asn			13117	2444	5.17	4.54	3663	474	4.87	3.58	-3.29E-01	Neutral	-2.58E-02	Neutral	-3.76E-02	Neutral
124	p.Arg124Lys			12813	2748	5.05	5.10	3694	668	4.91	5.04	-1.12E+00	Neutral	-8.71E-01	Neutral	-7.38E-01	Neutral
124	p.Arg124Thr			9192	1893	3.62	3.52	2863	759	3.80	5.73	-1.78E+00	Neutral	-9.91E+00	Indeterminate	-8.50E+00	Indeterminate
124	p.Arg124Arg	Synonymous		17487	4129	6.90	7.67	4768	964	6.34	7.27	-1.06E+00	Neutral	-1.06E+00	Neutral	-8.18E-01	Neutral
124	p.Arg124Ser			13488	2606	5.32	4.84	4071	483	5.41	3.65	-4.23E-01	Neutral	-3.91E-03	Neutral	-5.28E-02	Neutral
124	p.Arg124Ile			12169	3269	4.80	6.07	3406	1712	4.53	12.92	-4.52E+00	Neutral	-4.93E+01	Indeterminate	-3.30E+01	Indeterminate
124	p.Arg124Met			12928	2709	5.10	5.03	3850	244	5.12	1.84	-9.23E-01	Neutral	-4.15E-09	Neutral	-2.09E-01	Neutral
124	p.Arg124His			11854	2494	4.67	4.63	3574	303	4.75	2.29	-1.17E+00	Neutral	-1.16E-05	Neutral	-3.13E-01	Neutral
124	p.Arg124Gln			11493	2278	4.53	4.23	3369	459	4.48	3.46	-8.14E-01	Neutral	-7.35E-02	Neutral	-1.96E-01	Neutral
124	p.Arg124Pro			11653	2437	4.60	4.53	4001	473	5.32	3.57	-1.17E+00	Neutral	-4.05E-03	Neutral	-3.14E-01	Neutral
124	p.Arg124Leu			12505	2677	4.93	4.97	4088	679	5.43	5.12	-1.17E+00	Neutral	-3.17E-01	Neutral	-4.63E-01	Neutral
124	p.Arg124Asp			12156	2226	4.79	4.13	3222	321	4.28	2.42	-3.55E-01	Neutral	-6.88E-04	Neutral	-3.77E-02	Neutral
124	p.Arg124Glu			14476	2761	5.71	5.13	4456	841	5.92	6.35	-3.01E-01	Neutral	-7.49E-01	Neutral	-2.61E-01	Neutral
124	p.Arg124Ala			11408	2306	4.50	4.28	3663	419	4.87	3.16	-9.62E-01	Neutral	-3.81E-03	Neutral	-2.26E-01	Neutral
124	p.Arg124Gly			9347	2760	3.69	5.13	2629	570	3.49	4.30	-9.60E+00	Indeterminate	-4.91E+00	Neutral	-1.10E+01	Indeterminate
124	p.Arg124Val			14183	3045	5.59	5.66	4315	946	5.73	7.14	-8.79E-01	Neutral	-2.23E+00	Neutral	-1.45E+00	Neutral
124	p.Arg124Tyr			14546	2873	5.74	5.34	3803	1355	5.05	10.23	-4.11E-01	Neutral	-1.90E+01	Indeterminate	-1.56E+01	Indeterminate
124	p.Arg124Cys		Neutral	10657	2523	4.20	4.69	3124	287	4.15	2.17	-2.99E+00	Neutral	-1.71E-04	Neutral	-1.37E+00	Neutral
124	p.Arg124Trp			14282	3094	5.63	5.75	4499	564	5.98	4.26	-9.24E-01	Neutral	-5.62E-03	Neutral	-2.12E-01	Neutral
124	p.Arg124Phe			13826	2570	5.45	4.77	4199	730	5.58	5.51	-2.72E-01	Neutral	-4.44E-01	Neutral	-1.35E-01	Neutral
125	p.Asp125Asn			2848	2460	5.20	6.10	3408	1349	5.14	4.32	-1.78E+00	Neutral	-1.18E-01	Neutral	-6.88E-01	Neutral
125	p.Asp125Lys			3101	2194	5.66	5.44	4037	1492	6.09	4.78	-3.02E-01	Neutral	-2.37E-02	Neutral	-3.20E-02	Neutral
125	p.Asp125Thr			3289	1858	6.00	4.61	3748	1509	5.65	4.84	-1.52E-02	Neutral	-9.95E-02	Neutral	-4.33E-03	Neutral
125	p.Asp125Arg			3171	2491	5.79	6.18	3969	1800	5.99	5.77	-7.10E-01	Neutral	-2.91E-01	Neutral	-2.40E-01	Neutral
125	p.Asp125Ser			2543	1437	4.64	3.57	2832	1341	4.27	4.30	-5.04E-02	Neutral	-1.07E+00	Neutral	-2.91E-01	Neutral
125	p.Asp125Ile			2818	1560	5.14	3.87	3472	1190	5.24	3.81	-2.39E-02	Neutral	-1.64E-02	Neutral	-5.54E-04	Neutral
125	p.Asp125Met			2373	1268	4.33	3.15	2705	1017	4.08	3.26	-3.24E-02	Neutral	-1.56E-01	Neutral	-1.14E-02	Neutral
125	p.Asp125His		Neutral	2521	1745	4.60	4.33	3237	1195	4.88	3.83	-4.65E-01	Neutral	-6.25E-02	Neutral	-7.80E-02	Neutral
125	p.Asp125Gln			2478	1355	4.52	3.36	3307	1133	4.99	3.63	-3.65E-02	Neutral	-2.07E-02	Neutral	-1.10E-03	Neutral
125	p.Asp125Pro			1616	908	2.95	2.25	1907	835	2.88	2.68	-2.63E-01	Neutral	-1.54E+00	Neutral	-6.31E-01	Neutral
125	p.Asp125Leu			2325	1463	4.24	3.63	2786	1357	4.20	4.35	-2.44E-01	Neutral	-1.35E+00	Neutral	-5.21E-01	Neutral
125	p.Asp125Asp	Synonymous		2689	2109	4.91	5.23	3254	1612	4.91	5.17	-1.06E+00	Neutral	-1.06E+00	Neutral	-8.18E-01	Neutral
125	p.Asp125Glu			2333	1284	4.26	3.19	2599	1204	3.92	3.86	-5.18E-02	Neutral	-1.12E+00	Neutral	-3.14E-01	Neutral
125	p.Asp125Ala			2163	1221	3.95	3.03	2290	1343	3.46	4.31	-9.68E-02	Neutral	-5.33E+00	Neutral	-3.17E+00	Neutral
125	p.Asp125Gly			2889	2643	5.27	6.56	3529	1120	5.32	3.59	-2.44E+00	Neutral	-4.49E-03	Neutral	-1.02E+00	Neutral
125	p.Asp125Val			2562	1031	4.68	2.56	2832	1017	4.27	3.26	-1.62E-04	Neutral	-7.70E-02	Neutral	-1.99E-03	Neutral

125	p.Asp125Tyr			3302	6200	6.03	15.38	4205	5560	6.34	17.82	-2.96E+01	Indeterminate	-3.71E+01	Indeterminate	-5.32E+01	Deleterious
125	p.Asp125Cys			3684	3165	6.72	7.85	4837	1450	7.30	4.65	-9.62E-01	Neutral	-2.29E-04	Neutral	-2.25E-01	Neutral
125	p.Asp125Trp			3184	2210	5.81	5.48	3864	1751	5.83	5.61	-2.29E-01	Neutral	-3.15E-01	Neutral	-8.23E-02	Neutral
125	p.Asp125Phe			2896	1702	5.29	4.22	3460	1919	5.22	6.15	-4.79E-02	Neutral	-1.96E+00	Neutral	-7.48E-01	Neutral
126	p.Val126Asn			13592	1396	4.72	1.65					-4.72E-01	Neutral			-4.72E-01	Neutral
126	p.Val126Lys			15516	11295	5.38	13.32					-5.32E+01	Deleterious			-5.32E+01	Deleterious
126	p.Val126Thr			13332	577	4.63	0.68					-1.29E-07	Neutral			-1.28E-07	Neutral
126	p.Val126Arg			18926	19643	6.57	23.16					-5.32E+01	Deleterious			-5.32E+01	Deleterious
126	p.Val126Ser			13725	918	4.76	1.08					-1.54E-03	Neutral			-1.54E-03	Neutral
126	p.Val126Ile			16166	1121	5.61	1.32					-1.10E-03	Neutral			-1.10E-03	Neutral
126	p.Val126Met			19562	1317	6.79	1.55					-1.83E-04	Neutral			-1.83E-04	Neutral
126	p.Val126His			14043	4631	4.87	5.46					-5.30E+01	Indeterminate			-3.32E+01	Indeterminate
126	p.Val126Gln			16828	2136	5.84	2.52					-1.39E+00	Neutral			-1.39E+00	Neutral
126	p.Val126Pro			14969	885	5.19	1.04					-7.83E-05	Neutral			-7.83E-05	Neutral
126	p.Val126Leu			12287	717	4.26	0.85					-2.30E-04	Neutral			-2.30E-04	Neutral
126	p.Val126Asp	Pathogenic		10860	7618	3.77	8.98					-5.32E+01	Deleterious			-5.32E+01	Deleterious
126	p.Val126Glu			15495	1254	5.38	1.48					-1.80E-02	Neutral			-1.80E-02	Neutral
126	p.Val126Ala			13910	1058	4.83	1.25					-1.21E-02	Neutral			-1.21E-02	Neutral
126	p.Val126Gly			13329	1339	4.62	1.58					-4.10E-01	Neutral			-4.10E-01	Neutral
126	p.Val126Val	Synonymous		10679	1121	3.70	1.32					-1.06E+00	Neutral			-1.06E+00	Neutral
126	p.Val126Tyr			14358	11033	4.98	13.01					-5.32E+01	Deleterious			-5.32E+01	Deleterious
126	p.Val126Cys			10597	503	3.68	0.59					-7.77E-06	Neutral			-7.77E-06	Neutral
126	p.Val126Trp			16355	14867	5.67	17.53					-5.32E+01	Deleterious			-5.32E+01	Deleterious
126	p.Val126Phe			13728	1397	4.76	1.65					-4.22E-01	Neutral			-4.22E-01	Neutral
127	p.Ala127Asn			7524	1490	4.20	2.73					-1.01E+00	Neutral			-1.01E+00	Neutral
127	p.Ala127Lys			7364	2041	4.11	3.74					-6.63E+00	Indeterminate			-6.63E+00	Indeterminate
127	p.Ala127Thr			7875	3331	4.40	6.11					-2.48E+01	Indeterminate			-2.48E+01	Indeterminate
127	p.Ala127Arg			10173	4327	5.68	7.93					-1.99E+01	Indeterminate			-1.99E+01	Indeterminate
127	p.Ala127Ser	Benign		9439	2253	5.27	4.13					-2.10E+00	Neutral			-2.10E+00	Neutral
127	p.Ala127Ile			8459	3367	4.72	6.17					-1.96E+01	Indeterminate			-1.96E+01	Indeterminate
127	p.Ala127Met			10187	2188	5.69	4.01					-8.74E-01	Neutral			-8.74E-01	Neutral
127	p.Ala127His			7847	3375	4.38	6.19					-2.60E+01	Indeterminate			-2.60E+01	Indeterminate
127	p.Ala127Gln			9425	2342	5.26	4.29					-2.66E+00	Neutral			-2.66E+00	Neutral
127	p.Ala127Pro		Deleterious	5169	5329	2.89	9.77					-5.32E+01	Deleterious			-5.32E+01	Deleterious
127	p.Ala127Leu			9185	2356	5.13	4.32					-3.30E+00	Neutral			-3.30E+00	Neutral
127	p.Ala127Asp			10308	3893	5.76	7.14					-1.37E+01	Indeterminate			-1.37E+01	Indeterminate
127	p.Ala127Glu			11153	2432	6.23	4.46					-7.79E-01	Neutral			-7.79E-01	Neutral
127	p.Ala127Ala	Synonymous		7852	1588	4.38	2.91					-1.06E+00	Neutral			-1.06E+00	Neutral
127	p.Ala127Gly			8262	2466	4.61	4.52					-7.66E+00	Indeterminate			-7.66E+00	Indeterminate
127	p.Ala127Val			11122	2140	6.21	3.92					-2.61E-01	Neutral			-2.61E-01	Neutral
127	p.Ala127Tyr			7626	2133	4.26	3.91					-6.56E+00	Indeterminate			-6.56E+00	Indeterminate
127	p.Ala127Cys			8750	1407	4.89	2.58					-8.73E-02	Neutral			-8.73E-02	Neutral
127	p.Ala127Trp			10780	3177	6.02	5.82					-5.00E+00	Neutral			-5.00E+00	Neutral
127	p.Ala127Phe			10582	2912	5.91	5.34					-3.71E+00	Neutral			-3.71E+00	Neutral
128	p.Arg128Asn			2284	409	3.90	1.59					-5.62E-05	Neutral			-5.62E-05	Neutral
128	p.Arg128Lys			3039	1401	5.19	5.46					-2.71E+00	Neutral			-2.71E+00	Neutral
128	p.Arg128Thr			3022	1036	5.16	4.04					-3.17E-01	Neutral			-3.17E-01	Neutral
128	p.Arg128Arg	Synonymous		5105	2409	8.72	9.39					-1.06E+00	Neutral			-1.06E+00	Neutral
128	p.Arg128Ser			2145	1444	3.66	5.63					-1.91E+01	Indeterminate			-1.91E+01	Indeterminate
128	p.Arg128Ile			3252	1959	5.55	7.63					-8.33E+00	Indeterminate			-8.33E+00	Indeterminate
128	p.Arg128Met			2925	1150	4.99	4.48					-1.08E+00	Neutral			-1.08E+00	Neutral
128	p.Arg128His			1803	728	3.08	2.84					-3.37E+00	Neutral			-3.37E+00	Neutral
128	p.Arg128Gln			2303	651	3.93	2.54					-1.07E-01	Neutral			-1.07E-01	Neutral
128	p.Arg128Pro		Deleterious	2278	2859	3.89	11.14					-5.32E+01	Deleterious			-5.32E+01	Deleterious
128	p.Arg128Leu			1945	682	3.32	2.66					-1.25E+00	Neutral			-1.25E+00	Neutral
128	p.Arg128Asp			2937	906	5.02	3.53					-1.15E-01	Neutral			-1.15E-01	Neutral
128	p.Arg128Glu			4092	1701	6.99	6.63					-7.08E-01	Neutral			-7.08E-01	Neutral
128	p.Arg128Ala			2727	946	4.66	3.69					-4.82E-01	Neutral			-4.82E-01	Neutral
128	p.Arg128Gly			3260	1838	5.57	7.16					-6.37E+00	Indeterminate			-6.37E+00	Indeterminate
128	p.Arg128Val			3216	980	5.49	3.82					-6.95E-02	Neutral			-6.95E-02	Neutral
128	p.Arg128Tyr			2778	1025	4.74	3.99					-7.63E-01	Neutral			-7.63E-01	Neutral
128	p.Arg128Cys			2996	723	5.12	2.82					-3.13E-03	Neutral			-3.13E-03	Neutral
128	p.Arg128Trp			3307	1044	5.65	4.07					-9.54E-02	Neutral			-9.54E-02	Neutral
128	p.Arg128Phe			3149	1776	5.38	6.92					-6.69E+00	Indeterminate			-6.69E+00	Indeterminate
129	p.Tyr129Asn			3172	1902	2.84	4.42					-4.63E+01	Indeterminate			-3.32E+01	Indeterminate
129	p.Tyr129Lys			5186	1692	4.64	3.93					-3.97E+00	Neutral			-3.97E+00	Neutral
129	p.Tyr129Thr			3503	1741	3.14	4.05					-2.74E+01	Indeterminate			-2.74E+01	Indeterminate
129	p.Tyr129Arg			6578	1764	5.89	4.10					-7.14E-01	Neutral			-7.14E-01	Neutral
129	p.Tyr129Ser			3563	958	3.19	2.23					-2.79E+00	Neutral			-2.79E+00	Neutral
129	p.Tyr129Ile			5889	2057	5.27	4.78					-4.50E+00	Neutral			-4.50E+00	Neutral
129	p.Tyr129Met			4837	1185	4.33	2.76					-7.83E-01	Neutral			-7.83E-01	Neutral
129	p.Tyr129His			5494	1466	4.92	3.41					-1.08E+00	Neutral			-1.08E+00	Neutral
129	p.Tyr129Gln			6782	1841	6.07	4.28					-7.26E-01	Neutral			-7.26E-01	Neutral
129	p.Tyr129Pro			4492	9559	4.02	22.23					-5.32E+01	Deleterious			-5.32E+01	Deleterious
129	p.Tyr129Leu			4705	1043	4.21	2.43					-3.61E-01	Neutral			-3.61E-01	Neutral
129	p.Tyr129Asp			7326	3885	6.56	9.03					-1.65E+01	Indeterminate			-1.65E+01	Indeterminate
129	p.Tyr129Glu			5802	1453	5.19	3.38					-5.74E-01	Neutral			-5.74E-01	Neutral
129	p.Tyr129Ala			8100	2262	7.25	5.26					-5.65E-01	Neutral			-5.65E-01	Neutral
129	p.Tyr129Gly			6216	2149	5.56	5.00					-3.93E+00	Neutral			-3.93E+00	Neutral
129	p.Tyr129Val			7809	2285	6.99	5.31					-9.02E-01	Neutral			-9.02E-01	Neutral
129	p.Tyr129Tyr	Synonymous		4524	1129	4.05	2.63					-1.06E+00	Neutral			-1.06E+00	Neutral
129	p.Tyr129Cys			5819	1556	5.21	3.62					-9.55E-01	Neutral			-9.55E-01	Neutral
129	p.Tyr129Trp			6000	1338	5.37	3.11					-1.71E-01	Neutral			-1.71E-01	Neutral
129	p.Tyr129Phe			5935	1736	5.31	4.04					-1.69E+00	Neutral			-1.69E+00	Neutral
130	p.Leu130Asn			6622	5730	3.98	4.80					-2.91E+01	Indeterminate			-2.90E+01	Indeterminate
130	p.Leu130Lys			6586	10703	3.96	8.96					-5.32E+01	Deleterious			-5.32E+01	Deleterious
130	p.Leu130Thr			8414	4005	5.06	3.35					-2.31E+00	Neutral			-2.31E+00	Neutral
130	p.Leu130Arg			6954	11309	4.18	9.47					-5.32E+01	Deleterious			-5.32E+01	Deleterious
130	p.Leu130Ser			10649	5793	6.40	4.85					-3.13E+00	Neutral			-3.13E+00	Neutral
130	p.Leu130Ile			11747	4756	7.06	3.98					-2.97E-01	Neutral			-2.97E-01	Neutral
130	p.Leu130Met			8403	3454	5.05	2.89					-8.77E-01	Neutral			-8.77E-01	Neutral
130	p.Leu130His			5760	4572	3.46	3.83					-2.62E+01	Indeterminate			-2.62E+01	Indeterminate
130	p.Leu130Gln			6416	4936	3.86	4.13					-2.19E+01	Indeterminate			-2.19E+01	Indeterminate
130	p.Leu130Pro			5787	9545	3.48	7.99					-5.32E+01	Deleterious			-5.32E+01	Deleterious
130	p.Leu130Leu	Synonymous		9828	4367	5.91	3.66					-1.06E+00	Neutral			-1.06E+00	Neutral
130	p.Leu130Asp			7930	10361	4.77	8.67					-5.32E+01	Deleterious			-5.32E+01	Deleterious
130	p.Leu130Glu			9503	11579	5.71	9.69					-4.90E+01	Indeterminate			-3.32E+01	Indeterminate
130	p.Leu130Ala			6719	3124	4.04	2.62					-3.07E+00	Neutral			-3.07E+00	Neutral
130	p.Leu130Gly			9249	4455	5.56	3.73					-2.06E+00	Neutral			-2.06E+00	Neutral
130	p.Leu130Val			9958	3841	5.99	3.22					-3.15E-01	Neutral			-3.15E-01	Neutral
130	p.Leu130Tyr			7425	3577	4.46	2.99					-3.10E+00	Neutral			-3.10E+00	Neutral
130	p.Leu130Cys			10316	4878	6.20	4.08					-1.46E+00	Neutral			-1.46E+00	Neutral
130	p.Leu130Trp			4919	2621	2.96	2.19					-8.91E+00	Indeterminate			-8.91E+00	Indeterminate
130	p.Leu130Phe			13178	5836	7.92	4.89					-4.77E-01	Neutral			-4.77E-01	Neutral
131	p.Arg131Asn			5042	2882	3.58	2.81					-6.79E-01	Neutral			-6.79E-01	Neutral
131	p.Arg131Lys			9318	4561	6.62	4.44					-1.08E-02	Neutral			-1.08E-02	Neutral
131	p.Arg131Thr			7380	7597	5.25	7.40					-8.63E+00	Indeterminate			-8.63E+00	Indeterminate
131	p.Arg131Arg	Synonymous		4654	2747	3.31	2.68					-1.06E+00	Neutral			-1.06E+00	Neutral
131	p.Arg131Ser			6240	4107	4.44	4.00					-1.15E+00	Neutral			-1.15E+00	Neutral
131	p.Arg131Ile			7882	4014	5.60	3.91					-4.23E-02	Neutral			-4.23E-02	Neutral
131	p.Arg131Met			6830	3996	4.86	3.89					-3.46E-01	Neutral			-3.46E-01	Neutral
131	p.Arg131His			8168	5443	5.81	5.30					-6.42E-01	Neutral			-6.42E-01	Neutral
131	p.Arg131Gln			8309	11946	5.91	11.63					-2.23E+01	Indeterminate			-2.23E+01	Indeterminate
131	p.Arg131Pro			6340	7930	4.51	7.72					-1.94E+01	Indeterminate			-1.94E+01	Indeterminate
131	p.Arg131Leu			6498	2904	4.62	2.83					-1.66E-02	Neutral			-1.66E-02	Neutral
131	p.Arg131Asp			9630	4408	6.85	4.29					-3.02E-03	Neutral			-3.02E-03	Neutral
131	p.Arg131Glu			8105	4164	5.76	4.06					-4.21E-02	Neutral			-4.21E-02	Neutral
131	p.Arg131Ala			8275	5635	5.88	5.49					-7.38E-01	Neutral			-7.38E-01	Neutral
131	p.Arg131Gly			4870	2924	3.46	2.85					-1.08E+00	Neutral			-1.08E+00	Neutral
131	p.Arg131Val			4142	2191	2.94	2.13					-6.14E-01	Neutral			-6.14E-01	Neutral
131	p.Arg131Tyr			4020	2220	2.86	2.16					-9.31E-01	Neutral			-9.31E-01	Neutral
131	p.Arg131Cys			5494	12172	3.91	11.85					-5.32E+01	Deleterious			-5.32E+01	Deleterious
131	p.Arg131Trp			9383	6720	6.67	6.54					-7.90E-01	Neutral			-7.90E-01	Neutral
131	p.Arg131Phe			10081	4121	7.17	4.01					-2.80E-04	Neutral			-2.80E-04	Neutral
132	p.Ala132Asn			3857	2920	3.55	4.75					-3.99E+01	Indeterminate			-3.32E+01	Indeterminate
132	p.Ala132Lys			6605	2921	6.08	4.75					-3.78E+00	Neutral			-3.78E+00	Neutral
132	p.Ala132Thr			1388	575	1.28	0.94					-2.04E+01	Indeterminate			-2.04E+01	Indeterminate
132	p.Ala132Arg			8297	3403	7.63	5.54					-1.61E+00	Neutral			-1.61E+00	Neutral
132	p.Ala132Ser			4216	3329	3.88	5.42					-4.11E+01	Indeterminate			-3.32E+01	Indeterminate
132	p.Ala132Ile			4164	2141	3.83	3.48					-1.29E+01	Indeterminate			-1.29E+01	Indeterminate
132	p.Ala132Met			4378	2043	4.03	3.33					-8.62E+00	Indeterminate			-8.62E+00	Indeterminate
132	p.Ala132His			3831	2206	3.52	3.59					-1.99E+01	Indeterminate			-1.99E+01	Indeterminate
132	p.Ala132Gln			8852	3713	8.14	6.04					-1.62E+00	Neutral			-1.62E+00	Neutral
132	p.Ala132Pro			5579	2302	5.13	3.75					-3.53E+00	Neutral			-3.53E+00	Neutral
132	p.Ala132Leu			5922	3129	5.45	5.09					-9.42E+00	Indeterminate			-9.42E+00	Indeterminate
132	p.Ala132Asp			4119	2805	3.79	4.57					-2.93E+01	Indeterminate			-2.92E+01	Indeterminate
132	p.Ala132Glu			5719	3008	5.26	4.90					-9.67E+00	Indeterminate			-9.67E+00	Indeterminate
132	p.Ala132Ala	Synonymous		6066	2109	5.58	3.43					-1.06E+00	Neutral			-1.06E+00	Neutral
132	p.Ala132Gly			4391	8761	4.04	14.26					-5.32E+01	Deleterious			-5.32E+01	Deleterious

132	p.Ala132Val			7251	3409	6.67	5.55					-4.37E+00	Neutral			-4.37E+00	Neutral
132	p.Ala132Tyr			5165	2556	4.75	4.16					-8.74E+00	Indeterminate			-8.74E+00	Indeterminate
132	p.Ala132Cys			8345	5592	7.68	9.10					-1.44E+01	Indeterminate			-1.44E+01	Indeterminate
132	p.Ala132Trp			5564	2457	5.12	4.00					-4.92E+00	Neutral			-4.92E+00	Neutral
132	p.Ala132Phe			4977	2057	4.58	3.35					-4.29E+00	Neutral			-4.29E+00	Neutral
133	p.Ala133Asn			4372	938	4.80	3.57					-2.23E-02	Neutral			-2.23E-02	Neutral
133	p.Ala133Lys			4380	1041	4.81	3.96					-8.53E-02	Neutral			-8.53E-02	Neutral
133	p.Ala133Thr			4331	1060	4.76	4.03					-1.26E-01	Neutral			-1.26E-01	Neutral
133	p.Ala133Arg			3954	1335	4.34	5.08					-2.27E+00	Neutral			-2.27E+00	Neutral
133	p.Ala133Ser			6658	1862	7.31	7.08					-1.13E-01	Neutral			-1.13E-01	Neutral
133	p.Ala133Ile			4627	1388	5.08	5.28					-7.13E-01	Neutral			-7.13E-01	Neutral
133	p.Ala133Met			6154	1438	6.76	5.47					-1.48E-02	Neutral			-1.48E-02	Neutral
133	p.Ala133His			2367	508	2.60	1.93					-2.62E-01	Neutral			-2.62E-01	Neutral
133	p.Ala133Gln			4448	1056	4.89	4.02					-7.91E-02	Neutral			-7.91E-02	Neutral
133	p.Ala133Pro			6362	1967	6.99	7.48					-3.73E-01	Neutral			-3.73E-01	Neutral
133	p.Ala133Leu			5900	1653	6.48	6.29					-1.80E-01	Neutral			-1.80E-01	Neutral
133	p.Ala133Asp			2399	878	2.63	3.34					-7.43E+00	Indeterminate			-7.43E+00	Indeterminate
133	p.Ala133Glu			4841	1739	5.32	6.62					-2.21E+00	Neutral			-2.21E+00	Neutral
133	p.Ala133Ala	Synonymous		4332	1340	4.76	5.10					-1.06E+00	Neutral			-1.06E+00	Neutral
133	p.Ala133Gly			4928	1597	5.41	6.08					-1.09E+00	Neutral			-1.09E+00	Neutral
133	p.Ala133Val			5234	1964	5.75	7.47					-2.44E+00	Neutral			-2.44E+00	Neutral
133	p.Ala133Tyr			5290	1737	5.81	6.61					-1.01E+00	Neutral			-1.01E+00	Neutral
133	p.Ala133Cys			2580	572	2.83	2.18					-2.72E-01	Neutral			-2.72E-01	Neutral
133	p.Ala133Trp			4232	1160	4.65	4.41					-4.27E-01	Neutral			-4.27E-01	Neutral
133	p.Ala133Phe			3655	1054	4.01	4.01					-9.60E-01	Neutral			-9.60E-01	Neutral
134	p.Ala134Asn			8503	16401	4.84	4.71	3843	656	4.51	4.15	-5.88E-01	Neutral	-3.23E-02	Neutral	-1.04E-01	Neutral
134	p.Ala134Lys			6884	14985	3.92	4.30	3773	681	4.43	4.31	-2.24E+00	Neutral	-7.23E-02	Neutral	-9.30E-01	Neutral
134	p.Ala134Thr		Neutral	7442	13995	4.24	4.02	2573	670	3.02	4.24	-6.87E-01	Neutral	-3.79E+00	Neutral	-2.44E+00	Neutral
134	p.Ala134Arg			5325	12020	3.03	3.45	6217	601	7.30	3.80	-4.25E+00	Neutral	-6.81E-09	Neutral	-2.27E+00	Neutral
134	p.Ala134Ser			9157	17350	5.21	4.98	3396	901	3.99	5.70	-4.07E-01	Neutral	-2.57E+00	Neutral	-1.36E+00	Neutral
134	p.Ala134Ile			8079	14846	4.60	4.26	3017	641	3.54	4.06	-4.49E-01	Neutral	-7.67E-01	Neutral	-3.34E-01	Neutral
134	p.Ala134Met			7883	15439	4.49	4.43	3536	666	4.15	4.22	-8.14E-01	Neutral	-1.53E-01	Neutral	-2.27E-01	Neutral
134	p.Ala134His			9304	18154	5.29	5.21	4549	925	5.34	5.86	-5.03E-01	Neutral	-1.41E-01	Neutral	-1.12E-01	Neutral
134	p.Ala134Gln			8265	16273	4.70	4.67	3771	773	4.43	4.89	-7.50E-01	Neutral	-2.94E-01	Neutral	-2.58E-01	Neutral
134	p.Ala134Pro			5493	10521	3.13	3.02	3057	490	3.59	3.10	-1.62E+00	Neutral	-3.85E-02	Neutral	-5.53E-01	Neutral
134	p.Ala134Leu			9637	18558	5.48	5.33	4933	845	5.79	5.35	-4.04E-01	Neutral	-1.01E-02	Neutral	-5.00E-02	Neutral
134	p.Ala134Asp			10375	19782	5.90	5.68	4062	849	4.77	5.37	-2.92E-01	Neutral	-2.79E-01	Neutral	-9.00E-02	Neutral
134	p.Ala134Glu			11179	23452	6.36	6.74	7244	1068	8.51	6.76	-5.55E-01	Neutral	-5.89E-05	Neutral	-8.56E-02	Neutral
134	p.Ala134Ala	Synonymous		6807	13159	3.87	3.78	2772	598	3.26	3.79	-1.06E+00	Neutral	-1.06E+00	Neutral	-8.18E-01	Neutral
134	p.Ala134Gly			7599	15473	4.32	4.44	3125	574	3.67	3.63	-1.18E+00	Neutral	-1.81E-01	Neutral	-4.05E-01	Neutral
134	p.Ala134Val			11867	23859	6.75	6.85	5268	1204	6.19	7.62	-3.17E-01	Neutral	-2.87E-01	Neutral	-9.96E-02	Neutral
134	p.Ala134Tyr			11715	22207	6.67	6.38	4204	1130	4.94	7.15	-1.83E-01	Neutral	-1.83E+00	Neutral	-7.53E-01	Neutral
134	p.Ala134Cys			11760	21834	6.69	6.27	3466	958	4.07	6.06	-1.44E-01	Neutral	-3.10E+00	Neutral	-1.54E+00	Neutral
134	p.Ala134Trp			9783	23774	5.57	6.83	8758	932	10.28	5.90	-2.15E+00	Neutral	-4.19E-09	Neutral	-8.35E-01	Neutral
134	p.Ala134Phe			8664	16053	4.93	4.61	3592	634	4.22	4.01	-3.92E-01	Neutral	-6.71E-02	Neutral	-6.06E-02	Neutral
135	p.Gly135Asn			7098	1823	4.57	3.10					-1.29E-02	Neutral			-1.29E-02	Neutral
135	p.Gly135Lys			5037	2084	3.24	3.55					-3.29E+00	Neutral			-3.29E+00	Neutral
135	p.Gly135Thr			5864	2062	3.77	3.51					-8.61E-01	Neutral			-8.61E-01	Neutral
135	p.Gly135Arg			13028	3421	8.38	5.82					-5.94E-04	Neutral			-5.94E-04	Neutral
135	p.Gly135Ser			4979	1773	3.20	3.02					-1.39E+00	Neutral			-1.39E+00	Neutral
135	p.Gly135Ile			8051	3609	5.18	6.14					-2.16E+00	Neutral			-2.16E+00	Neutral
135	p.Gly135Met			12233	4835	7.87	8.23					-2.87E-01	Neutral			-2.87E-01	Neutral
135	p.Gly135His			11507	4705	7.40	8.01					-4.74E-01	Neutral			-4.74E-01	Neutral
135	p.Gly135Gln			6056	2112	3.90	3.59					-7.44E-01	Neutral			-7.44E-01	Neutral
135	p.Gly135Pro			8775	3358	5.65	5.71					-5.81E-01	Neutral			-5.81E-01	Neutral
135	p.Gly135Leu			8259	3502	5.31	5.96					-1.45E+00	Neutral			-1.45E+00	Neutral
135	p.Gly135Asp			5506	1975	3.54	3.36					-1.16E+00	Neutral			-1.16E+00	Neutral
135	p.Gly135Glu			4893	3812	3.15	6.49					-3.14E+01	Indeterminate			-3.10E+01	Indeterminate
135	p.Gly135Ala			6693	2173	4.31	3.70					-2.97E-01	Neutral			-2.97E-01	Neutral
135	p.Gly135Gly	Synonymous		8277	3357	5.33	5.71					-1.06E+00	Neutral			-1.06E+00	Neutral
135	p.Gly135Val			5528	2052	3.56	3.49					-1.45E+00	Neutral			-1.45E+00	Neutral
135	p.Gly135Tyr			7744	2852	4.98	4.85					-5.97E-01	Neutral			-5.97E-01	Neutral
135	p.Gly135Cys			8671	2609	5.58	4.44					-4.73E-02	Neutral			-4.73E-02	Neutral
135	p.Gly135Trp			9281	3513	5.97	5.98					-4.51E-01	Neutral			-4.51E-01	Neutral
135	p.Gly135Phe			7947	3144	5.11	5.35					-9.77E-01	Neutral			-9.77E-01	Neutral
136	p.Gly136Asn			10420	5992	5.71	5.62	12915	6828	5.88	9.49	-5.84E-01	Neutral	-9.84E+00	Indeterminate	-7.38E+00	Indeterminate
136	p.Gly136Lys			9133	4870	5.00	4.57	10618	2631	4.83	3.66	-4.44E-01	Neutral	-9.93E-02	Neutral	-8.23E-02	Neutral
136	p.Gly136Thr			11568	6636	6.33	6.22	13564	1561	6.18	2.17	-4.23E-01	Neutral	-2.85E-09	Neutral	-5.21E-02	Neutral
136	p.Gly136Arg			13087	7645	7.17	7.17	15369	3820	7.00	5.31	-3.43E-01	Neutral	-2.07E-02	Neutral	-3.94E-02	Neutral
136	p.Gly136Ser			9479	5468	5.19	5.13	11411	5318	5.20	7.39	-7.72E-01	Neutral	-7.10E+00	Indeterminate	-5.18E+00	Neutral
136	p.Gly136Ile			7498	4411	4.11	4.14	9506	1638	4.33	2.28	-1.55E+00	Neutral	-6.45E-04	Neutral	-4.96E-01	Neutral
136	p.Gly136Met			10463	6084	5.73	5.70	13373	2252	6.09	3.13	-6.33E-01	Neutral	-4.03E-05	Neutral	-1.08E-01	Neutral
136	p.Gly136His			10513	5586	5.76	5.24	11604	6173	5.28	8.58	-2.78E-01	Neutral	-1.14E+01	Indeterminate	-8.50E+00	Indeterminate
136	p.Gly136Gln			8073	4302	4.42	4.03	9656	3063	4.40	4.26	-6.27E-01	Neutral	-1.26E+00	Neutral	-6.79E-01	Neutral
136	p.Gly136Pro			6566	3880	3.59	3.64	8140	4546	3.71	6.32	-2.09E+00	Neutral	-1.93E+01	Indeterminate	-1.74E+01	Indeterminate
136	p.Gly136Leu			9190	5242	5.03	4.92	11122	5391	5.06	7.49	-7.66E-01	Neutral	-8.57E+00	Indeterminate	-6.43E+00	Indeterminate
136	p.Gly136Asp			9243	5278	5.06	4.95	10964	7479	4.99	10.40	-7.61E-01	Neutral	-2.56E+01	Indeterminate	-2.20E+01	Indeterminate
136	p.Gly136Glu			6896	3746	3.78	3.51	8983	1778	4.09	2.47	-1.09E+00	Neutral	-9.84E-03	Neutral	-2.80E-01	Neutral
136	p.Gly136Ala			8356	5198	4.58	4.87	9671	1125	4.40	1.56	-1.76E+00	Neutral	-7.70E-08	Neutral	-6.07E-01	Neutral
136	p.Gly136Gly	Synonymous		11614	7478	6.36	7.01	14031	4919	6.39	6.84	-1.06E+00	Neutral	-1.06E+00	Neutral	-8.18E-01	Neutral
136	p.Gly136Val			12033	7432	6.59	6.97	14744	2105	6.71	2.93	-7.08E-01	Neutral	-4.00E-07	Neutral	-1.32E-01	Neutral
136	p.Gly136Tyr			7640	5300	4.18	4.97	9352	2412	4.26	3.35	-3.80E+00	Neutral	-2.43E-01	Neutral	-2.12E+00	Neutral
136	p.Gly136Cys			7914	4580	4.33	4.29	9143	4462	4.16	6.20	-1.23E+00	Neutral	-1.11E+01	Indeterminate	-9.10E+00	Indeterminate
136	p.Gly136Trp			4677	2955	2.56	2.77	5657	474	2.58	0.66	-5.27E+00	Neutral	-1.35E-09	Neutral	-3.05E+00	Neutral
136	p.Gly136Phe			8281	4565	4.53	4.28	9815	3962	4.47	5.51	-7.69E-01	Neutral	-4.74E+00	Neutral	-3.24E+00	Neutral
137	p.Thr137Asn			12637	7950	6.42	6.37					-5.48E-01	Neutral			-5.48E-01	Neutral
137	p.Thr137Lys			10056	5827	5.11	4.67					-5.19E-01	Neutral			-5.19E-01	Neutral
137	p.Thr137Thr	Synonymous		9153	5630	4.65	4.51					-1.06E+00	Neutral			-1.06E+00	Neutral
137	p.Thr137Arg			10802	7256	5.48	5.81					-1.34E+00	Neutral			-1.34E+00	Neutral
137	p.Thr137Ser			15695	10181	7.97	8.15					-3.80E-01	Neutral			-3.80E-01	Neutral
137	p.Thr137Ile			5926	3761	3.01	3.01					-3.13E+00	Neutral			-3.13E+00	Neutral
137	p.Thr137Met			15098	10222	7.67	8.18					-6.18E-01	Neutral			-6.18E-01	Neutral
137	p.Thr137His			7921	5006	4.02	4.01					-1.76E+00	Neutral			-1.76E+00	Neutral
137	p.Thr137Gln			2342	1107	1.19	0.89					-3.36E+00	Neutral			-3.36E+00	Neutral
137	p.Thr137Pro			6861	3500	3.48	2.80					-5.17E-01	Neutral			-5.17E-01	Neutral
137	p.Thr137Leu			11186	7011	5.68	5.61					-7.40E-01	Neutral			-7.40E-01	Neutral
137	p.Thr137Asp			6374	3772	3.24	3.02					-1.83E+00	Neutral			-1.83E+00	Neutral
137	p.Thr137Glu			9691	6160	4.92	4.93					-1.17E+00	Neutral			-1.17E+00	Neutral
137	p.Thr137Ala			8690	6226	4.41	4.98					-3.04E+00	Neutral			-3.04E+00	Neutral
137	p.Thr137Gly			14994	10033	7.61	8.03					-5.71E-01	Neutral			-5.71E-01	Neutral
137	p.Thr137Val			17827	12301	9.05	9.85					-4.53E-01	Neutral			-4.53E-01	Neutral
137	p.Thr137Tyr			7543	4012	3.83	3.21					-5.64E-01	Neutral			-5.64E-01	Neutral
137	p.Thr137Cys			4535	3046	2.30	2.44					-6.22E+00	Indeterminate			-6.22E+00	Indeterminate
137	p.Thr137Trp			9317	5548	4.73	4.44					-7.97E-01	Neutral			-7.97E-01	Neutral
137	p.Thr137Phe			10305	6346	5.23	5.08					-7.98E-01	Neutral			-7.98E-01	Neutral
138	p.Arg138Asn			9124	4360	3.23	3.99					-1.57E+00	Neutral			-1.57E+00	Neutral
138	p.Arg138Lys			16369	6363	5.80	5.82					-3.22E-02	Neutral			-3.22E-02	Neutral
138	p.Arg138Thr			14620	6499	5.18	5.94					-2.46E-01	Neutral			-2.46E-01	Neutral
138	p.Arg138Arg	Synonymous		7495	3167	2.65	2.90					-1.06E+00	Neutral			-1.06E+00	Neutral
138	p.Arg138Ser			15989	5361	5.66	4.90					-3.76E-03	Neutral			-3.76E-03	Neutral
138	p.Arg138Ile			21820	8258	7.73	7.55					-5.06E-03	Neutral			-5.06E-03	Neutral
138	p.Arg138Met			27553	10505	9.76	9.61					-1.51E-03	Neutral			-1.51E-03	Neutral
138	p.Arg138His			7027	2951	2.49	2.70					-1.18E+00	Neutral			-1.18E+00	Neutral
138	p.Arg138Gln			15327	6033	5.43	5.52					-5.09E-02	Neutral			-5.09E-02	Neutral
138	p.Arg138Pro			6749	3535	2.39	3.23					-4.52E+00	Neutral			-4.52E+00	Neutral
138	p.Arg138Leu			33430	13311	11.84	12.17					-1.03E-03	Neutral			-1.03E-03	Neutral
138	p.Arg138Asp			16062	6554	5.69	5.99					-6.60E-02	Neutral			-6.60E-02	Neutral
138	p.Arg138Glu			6604	2634	2.34	2.41					-9.47E-01	Neutral			-9.47E-01	Neutral
138	p.Arg138Ala			1275	310	0.45	0.28					-1.36E+00	Neutral			-1.36E+00	Neutral
138	p.Arg138Gly			9750	3959	3.45	3.62					-3.76E-01	Neutral			-3.76E-01	Neutral
138	p.Arg138Val			10559	4030	3.74	3.69					-1.54E-01	Neutral			-1.54E-01	Neutral
138	p.Arg138Tyr			15818	4829	5.60	4.42					-7.49E-04	Neutral			-7.49E-04	Neutral
138	p.Arg138Cys			18784	6819	6.65	6.24					-5.78E-03	Neutral			-5.78E-03	Neutral
138	p.Arg138Trp			17148	5835	6.07	5.34					-3.25E-03	Neutral			-3.25E-03	Neutral
138	p.Arg138Phe			10816	4048	3.83	3.70					-1.13E-01	Neutral			-1.13E-01	Neutral
139	p.Gly139Asn			8496	2550	3.53	3.45	3712	1812	3.95	3.41	-2.29E+00	Neutral	-2.33E+00	Neutral	-2.55E+00	Neutral
139	p.Gly139Lys			12403	4023	5.15	5.44	4555	2814	4.84	5.29	-1.71E+00	Neutral	-5.43E+00	Neutral	-4.57E+00	Neutral
139	p.Gly139Thr			8144	2197	3.38	2.97	2926	1791	3.11	3.37	-1.28E+00	Neutral	-9.45E+00	Indeterminate	-7.65E+00	Indeterminate
139	p.Gly139Arg		Neutral	12860	3446	5.34	4.66	4701	2594	5.00	4.88	-3.60E-01	Neutral	-2.98E+00	Neutral	-1.61E+00	Neutral
139	p.Gly139Ser			13019	4819	5.41	6.51	5676	3432	6.04	6.45	-3.32E+00	Neutral	-3.46E+00	Neutral	-4.27E+00	Neutral
139	p.Gly139Ile			15538	4725	6.46	6.39	5905	3339	6.28	6.28	-6.28E-01	Neutral	-2.24E+00	Neutral	-1.29E+00	Neutral
139	p.Gly139Met			11545	4056	4.80	5.48	4565	2731	4.85	5.14	-3.11E+00	Neutral	-4.68E+00	Neutral	-5.11E+00	Neutral
139	p.Gly139His			11470	3114	4.77	4.21	4453	2398	4.74	4.51	-5.65E-01	Neutral	-2.88E+00	Neutral	-1.68E+00	Neutral
139	p.Gly139Gln			10561	3014	4.39	4.07	4842	2382	5.15	4.48	-1.03E+00	Neutral	-1.42E+00	Neutral	-1.02E+00	Neutral
139	p.Gly139Pro			8278	3312	3.44	4.48	4736	2102	5.04	3.95	-9.03E+00	Indeterminate	-7.05E-01	Neutral	-6.78E+00	Indeterminate
139	p.Gly139Leu			13458	3515	5.59	4.75	4746	3057	5.05	5.75	-2.42E-01	Neutral	-6.13E+00	Neutral	-3.93E+00	Neutral
139	p.Gly139Asp			12264	3368	5.10	4.55	3626	2391	3.86	4.50	-5.16E-01	Neutral	-9.57E+00	Indeterminate	-7.08E+00	Indeterminate
139	p.Gly139Glu			14048	4837	5.84	6.54	5228	3110	5.56	5.85	-1.92E+00	Neutral	-3.66E+00	Neutral	-3.30E+00	Neutral
139	p.Gly139Ala			10712	2633	4.45	3.56	3613	2393	3.84	4.50	-2.81E-01	Neutral	-9.76E+00	Indeterminate	-7.05E+00	Indeterminate
139	p.Gly139Gly	Synonymous		13109	4034	5.45	5.45	5129	2467	5.45	4.64	-1.06E+00	Neutral	-1.06E+00	Neutral	-8.18E-01	Neutral

139	p.Gly139Val			15407	4203	6.40	5.68	6369	3174	6.77	5.97	-2.39E-01	Neutral	-8.09E-01	Neutral	-2.60E-01	Neutral
139	p.Gly139Tyr			10886	4620	4.52	6.24	4241	2748	4.51	5.17	-8.00E+00	Indeterminate	-7.33E+00	Indeterminate	-1.18E+01	Indeterminate
139	p.Gly139Cys			12796	3369	5.32	4.55	5017	2479	5.34	4.66	-3.09E-01	Neutral	-1.35E+00	Neutral	-5.54E-01	Neutral
139	p.Gly139Trp			12302	3446	5.11	4.66	4799	2716	5.10	5.11	-6.04E-01	Neutral	-3.28E+00	Neutral	-2.00E+00	Neutral
139	p.Gly139Phe			13311	4715	5.53	6.37	5200	3243	5.53	6.10	-2.52E+00	Neutral	-4.64E+00	Neutral	-4.58E+00	Neutral
140	p.Ser140Asn			10552	7922	5.17	5.24					-7.53E-01	Neutral			-7.53E-01	Neutral
140	p.Ser140Lys			11164	7762	5.47	5.14					-3.30E-01	Neutral			-3.30E-01	Neutral
140	p.Ser140Thr			12855	9304	6.30	6.16					-3.09E-01	Neutral			-3.09E-01	Neutral
140	p.Ser140Arg			7945	6865	3.89	4.54					-3.36E+00	Neutral			-3.36E+00	Neutral
140	p.Ser140Ser	Synonymous		8620	6334	4.23	4.19					-1.06E+00	Neutral			-1.06E+00	Neutral
140	p.Ser140Ile			15602	10874	7.65	7.20					-1.05E-01	Neutral			-1.05E-01	Neutral
140	p.Ser140Met			7484	5193	3.67	3.44					-9.84E-01	Neutral			-9.84E-01	Neutral
140	p.Ser140His			9812	7506	4.81	4.97					-1.04E+00	Neutral			-1.04E+00	Neutral
140	p.Ser140Gln			11521	8353	5.65	5.53					-4.40E-01	Neutral			-4.40E-01	Neutral
140	p.Ser140Pro			5355	4736	2.62	3.13					-6.82E+00	Indeterminate			-6.82E+00	Indeterminate
140	p.Ser140Leu			9344	6183	4.58	4.09					-3.61E-01	Neutral			-3.61E-01	Neutral
140	p.Ser140Asp			15150	10921	7.43	7.23					-1.71E-01	Neutral			-1.71E-01	Neutral
140	p.Ser140Glu			9632	6353	4.72	4.20					-3.18E-01	Neutral			-3.18E-01	Neutral
140	p.Ser140Ala			4610	3616	2.26	2.39					-5.04E+00	Neutral			-5.04E+00	Neutral
140	p.Ser140Gly			6200	5842	3.04	3.87					-7.24E+00	Indeterminate			-7.24E+00	Indeterminate
140	p.Ser140Val			17043	12142	8.35	8.04					-9.60E-02	Neutral			-9.60E-02	Neutral
140	p.Ser140Tyr			15829	12798	7.76	8.47					-4.54E-01	Neutral			-4.54E-01	Neutral
140	p.Ser140Cys			11151	8478	5.47	5.61					-7.20E-01	Neutral			-7.20E-01	Neutral
140	p.Ser140Trp			7713	5562	3.78	3.68					-1.21E+00	Neutral			-1.21E+00	Neutral
140	p.Ser140Phe			6438	4352	3.16	2.88					-1.17E+00	Neutral			-1.17E+00	Neutral
141	p.Asn141Asn	Synonymous		9534	7176	4.94	5.44					-1.06E+00	Neutral			-1.06E+00	Neutral
141	p.Asn141Lys			6445	3767	3.34	2.86					-3.91E-01	Neutral			-3.91E-01	Neutral
141	p.Asn141Thr			5628	4142	2.91	3.14					-2.77E+00	Neutral			-2.77E+00	Neutral
141	p.Asn141Arg			9129	6690	4.73	5.07					-9.71E-01	Neutral			-9.71E-01	Neutral
141	p.Asn141Ser			9765	6185	5.06	4.69					-2.24E-01	Neutral			-2.24E-01	Neutral
141	p.Asn141Ile			11867	8445	6.14	6.41					-3.71E-01	Neutral			-3.71E-01	Neutral
141	p.Asn141Met			11856	8168	6.14	6.20					-2.73E-01	Neutral			-2.73E-01	Neutral
141	p.Asn141His			4415	2868	2.29	2.18					-2.15E+00	Neutral			-2.15E+00	Neutral
141	p.Asn141Gln			10967	8321	5.68	6.31					-7.96E-01	Neutral			-7.96E-01	Neutral
141	p.Asn141Pro			6338	4134	3.28	3.14					-1.00E+00	Neutral			-1.00E+00	Neutral
141	p.Asn141Leu			11622	7486	6.02	5.68					-1.45E-01	Neutral			-1.45E-01	Neutral
141	p.Asn141Asp			13047	9029	6.76	6.85					-2.07E-01	Neutral			-2.07E-01	Neutral
141	p.Asn141Glu			7938	5777	4.11	4.38					-1.28E+00	Neutral			-1.28E+00	Neutral
141	p.Asn141Ala			12198	8306	6.32	6.30					-2.20E-01	Neutral			-2.20E-01	Neutral
141	p.Asn141Gly			9905	6558	5.13	4.97					-3.31E-01	Neutral			-3.31E-01	Neutral
141	p.Asn141Val			15454	9860	8.00	7.48					-4.06E-02	Neutral			-4.06E-02	Neutral
141	p.Asn141Tyr			6746	4426	3.49	3.36					-9.00E-01	Neutral			-9.00E-01	Neutral
141	p.Asn141Cys			4124	2702	2.14	2.05					-2.57E+00	Neutral			-2.57E+00	Neutral
141	p.Asn141Trp			15020	9827	7.78	7.45					-6.36E-02	Neutral			-6.36E-02	Neutral
141	p.Asn141Phe			11136	7974	5.77	6.05					-4.74E-01	Neutral			-4.74E-01	Neutral
142	p.His142Asn			8383	1168	4.70	4.66					-1.78E-04	Neutral			-1.78E-04	Neutral
142	p.His142Lys			10971	1665	6.15	6.65					-1.65E-04	Neutral			-1.65E-04	Neutral
142	p.His142Thr			8686	1345	4.87	5.37					-1.10E-03	Neutral			-1.10E-03	Neutral
142	p.His142Arg			10575	1237	5.93	4.94					-6.01E-07	Neutral			-6.01E-07	Neutral
142	p.His142Ser			7833	1082	4.39	4.32					-2.36E-04	Neutral			-2.36E-04	Neutral
142	p.His142Ile			11025	1519	6.18	6.06					-2.03E-05	Neutral			-2.03E-05	Neutral
142	p.His142Met			7777	1104	4.36	4.41					-4.24E-04	Neutral			-4.24E-04	Neutral
142	p.His142His	Synonymous		1320	190	0.74	0.76					-1.06E+00	Neutral			-1.06E+00	Neutral
142	p.His142Gln			6699	1050	3.76	4.19					-6.05E-03	Neutral			-6.05E-03	Neutral
142	p.His142Pro			5548	762	3.11	3.04					-1.85E-03	Neutral			-1.85E-03	Neutral
142	p.His142Leu			15858	2076	8.89	8.29					-3.48E-07	Neutral			-3.48E-07	Neutral
142	p.His142Asp			8253	1295	4.63	5.17					-1.89E-03	Neutral			-1.89E-03	Neutral
142	p.His142Glu			10412	1458	5.84	5.82					-4.39E-05	Neutral			-4.39E-05	Neutral
142	p.His142Ala			12203	1693	6.84	6.76					-1.11E-05	Neutral			-1.11E-05	Neutral
142	p.His142Gly			7160	966	4.01	3.86					-2.68E-04	Neutral			-2.68E-04	Neutral
142	p.His142Val			11560	1684	6.48	6.72					-4.87E-05	Neutral			-4.87E-05	Neutral
142	p.His142Tyr			8206	1063	4.60	4.24					-4.50E-05	Neutral			-4.50E-05	Neutral
142	p.His142Cys			9000	1249	5.05	4.99					-1.01E-04	Neutral			-1.01E-04	Neutral
142	p.His142Trp			9332	1224	5.23	4.89					-2.33E-05	Neutral			-2.33E-05	Neutral
142	p.His142Phe			7571	1225	4.24	4.89					-5.23E-03	Neutral			-5.23E-03	Neutral
143	p.Ala143Asn			10595	21434	4.99	5.07					-1.04E+00	Neutral			-1.04E+00	Neutral
143	p.Ala143Lys			11917	23232	5.61	5.49					-5.77E-01	Neutral			-5.77E-01	Neutral
143	p.Ala143Thr		Neutral	12320	23747	5.80	5.61					-4.77E-01	Neutral			-4.77E-01	Neutral
143	p.Ala143Arg			10547	20563	4.96	4.86					-8.01E-01	Neutral			-8.01E-01	Neutral
143	p.Ala143Ser			9145	17169	4.30	4.06					-8.62E-01	Neutral			-8.62E-01	Neutral
143	p.Ala143Ile			9506	17634	4.47	4.17					-7.11E-01	Neutral			-7.11E-01	Neutral
143	p.Ala143Met			10605	21003	4.99	4.96					-8.91E-01	Neutral			-8.91E-01	Neutral
143	p.Ala143His			9550	18973	4.49	4.48					-1.17E+00	Neutral			-1.17E+00	Neutral
143	p.Ala143Gln			11085	22354	5.22	5.28					-9.13E-01	Neutral			-9.13E-01	Neutral
143	p.Ala143Pro			8704	17930	4.10	4.24					-1.82E+00	Neutral			-1.82E+00	Neutral
143	p.Ala143Leu			9306	17759	4.38	4.20					-9.33E-01	Neutral			-9.33E-01	Neutral
143	p.Ala143Asp			8557	15731	4.03	3.72					-8.69E-01	Neutral			-8.69E-01	Neutral
143	p.Ala143Glu			11690	23121	5.50	5.47					-6.85E-01	Neutral			-6.85E-01	Neutral
143	p.Ala143Ala	Synonymous		10390	20931	4.89	4.95					-1.06E+00	Neutral			-1.06E+00	Neutral
143	p.Ala143Gly			12401	25612	5.84	6.05					-8.25E-01	Neutral			-8.25E-01	Neutral
143	p.Ala143Val			11134	21640	5.24	5.12					-6.78E-01	Neutral			-6.78E-01	Neutral
143	p.Ala143Tyr			10660	21796	5.02	5.15					-1.11E+00	Neutral			-1.11E+00	Neutral
143	p.Ala143Cys			10965	22961	5.16	5.43					-1.23E+00	Neutral			-1.23E+00	Neutral
143	p.Ala143Trp			11086	24426	5.22	5.77					-1.68E+00	Neutral			-1.68E+00	Neutral
143	p.Ala143Phe			12322	25053	5.80	5.92					-7.43E-01	Neutral			-7.43E-01	Neutral
144	p.Arg144Asn			19759	3661	6.06	5.83					-1.14E-01	Neutral			-1.14E-01	Neutral
144	p.Arg144Lys			11748	2225	3.60	3.54					-7.41E-01	Neutral			-7.41E-01	Neutral
144	p.Arg144Thr			15043	2830	4.61	4.50					-3.45E-01	Neutral			-3.45E-01	Neutral
144	p.Arg144Arg	Synonymous		13943	2932	4.28	4.67					-1.06E+00	Neutral			-1.06E+00	Neutral
144	p.Arg144Ser			13559	2514	4.16	4.00					-4.14E-01	Neutral			-4.14E-01	Neutral
144	p.Arg144Ile			13225	2572	4.06	4.09					-6.69E-01	Neutral			-6.69E-01	Neutral
144	p.Arg144Met			14812	2776	4.54	4.42					-3.49E-01	Neutral			-3.49E-01	Neutral
144	p.Arg144His			13721	2644	4.21	4.21					-5.60E-01	Neutral			-5.60E-01	Neutral
144	p.Arg144Gln			16857	3371	5.17	5.37					-4.27E-01	Neutral			-4.27E-01	Neutral
144	p.Arg144Pro			9345	1766	2.87	2.81					-1.27E+00	Neutral			-1.27E+00	Neutral
144	p.Arg144Leu			23232	4702	7.13	7.48					-1.68E-01	Neutral			-1.68E-01	Neutral
144	p.Arg144Asp			13837	2604	4.24	4.15					-4.45E-01	Neutral			-4.45E-01	Neutral
144	p.Arg144Glu			16608	3337	5.09	5.31					-4.65E-01	Neutral			-4.65E-01	Neutral
144	p.Arg144Ala			17489	3491	5.36	5.56					-3.75E-01	Neutral			-3.75E-01	Neutral
144	p.Arg144Gly			13921	2836	4.27	4.51					-8.42E-01	Neutral			-8.42E-01	Neutral
144	p.Arg144Val			19701	3764	6.04	5.99					-1.63E-01	Neutral			-1.63E-01	Neutral
144	p.Arg144Tyr			16291	3119	5.00	4.96					-3.17E-01	Neutral			-3.17E-01	Neutral
144	p.Arg144Cys	Benign		24189	4270	7.42	6.80					-2.61E-02	Neutral			-2.61E-02	Neutral
144	p.Arg144Trp			17236	3316	5.29	5.28					-2.77E-01	Neutral			-2.77E-01	Neutral
144	p.Arg144Phe			21521	4091	6.60	6.51					-1.11E-01	Neutral			-1.11E-01	Neutral
145	p.Ile145Asn			7612	1838	4.67	4.38					-6.79E-01	Neutral			-6.79E-01	Neutral
145	p.Ile145Lys			11244	3123	6.90	7.45					-7.38E-01	Neutral			-7.38E-01	Neutral
145	p.Ile145Thr			7751	1989	4.76	4.74					-1.03E+00	Neutral			-1.03E+00	Neutral
145	p.Ile145Arg			8952	2589	5.50	6.17					-1.68E+00	Neutral			-1.68E+00	Neutral
145	p.Ile145Ser			10994	2980	6.75	7.11					-6.44E-01	Neutral			-6.44E-01	Neutral
145	p.Ile145Ile	Synonymous		6650	1628	4.08	3.88					-1.06E+00	Neutral			-1.06E+00	Neutral
145	p.Ile145Met			7048	2095	4.33	5.00					-3.12E+00	Neutral			-3.12E+00	Neutral
145	p.Ile145His			7200	1665	4.42	3.97					-5.54E-01	Neutral			-5.54E-01	Neutral
145	p.Ile145Gln			8916	2404	5.47	5.73					-1.06E+00	Neutral			-1.06E+00	Neutral
145	p.Ile145Pro			8974	2142	5.51	5.11					-3.84E-01	Neutral			-3.84E-01	Neutral
145	p.Ile145Leu			8437	2140	5.18	5.10					-7.65E-01	Neutral			-7.65E-01	Neutral
145	p.Ile145Asp			7325	1859	4.50	4.43					-1.09E+00	Neutral			-1.09E+00	Neutral
145	p.Ile145Glu			9464	2422	5.81	5.78					-6.05E-01	Neutral			-6.05E-01	Neutral
145	p.Ile145Ala			5278	1486	3.24	3.54					-3.86E+00	Neutral			-3.86E+00	Neutral
145	p.Ile145Gly			9634	2314	5.92	5.52					-3.27E-01	Neutral			-3.27E-01	Neutral
145	p.Ile145Val			6709	1848	4.12	4.41					-2.24E+00	Neutral			-2.24E+00	Neutral
145	p.Ile145Tyr			7802	1946	4.79	4.64					-8.22E-01	Neutral			-8.22E-01	Neutral
145	p.Ile145Cys			8714	1957	5.35	4.67					-2.34E-01	Neutral			-2.34E-01	Neutral
145	p.Ile145Trp			7610	1935	4.67	4.61					-1.01E+00	Neutral			-1.01E+00	Neutral
145	p.Ile145Phe			6546	1571	4.02	3.75					-9.54E-01	Neutral			-9.54E-01	Neutral
146	p.Asp146Asn			10106	2780	4.71	5.30					-3.08E+00	Neutral			-3.08E+00	Neutral
146	p.Asp146Lys			9313	1900	4.34	3.62					-4.83E-01	Neutral			-4.83E-01	Neutral
146	p.Asp146Thr			8674	2156	4.04	4.11					-2.33E+00	Neutral			-2.33E+00	Neutral
146	p.Asp146Arg			15511	3860	7.23	7.35					-6.19E-01	Neutral			-6.19E-01	Neutral
146	p.Asp146Ser			11188	2585	5.21	4.93					-8.14E-01	Neutral			-8.14E-01	Neutral
146	p.Asp146Ile			9107	1893	4.24	3.61					-6.03E-01	Neutral			-6.03E-01	Neutral
146	p.Asp146Met			12934	3264	6.03	6.22					-1.10E+00	Neutral			-1.10E+00	Neutral
146	p.Asp146His			10636	2801	4.96	5.34					-2.20E+00	Neutral			-2.20E+00	Neutral
146	p.Asp146Gln			10024	2577	4.67	4.91					-2.14E+00	Neutral			-2.14E+00	Neutral
146	p.Asp146Pro			11243	2945	5.24	5.61					-1.91E+00	Neutral			-1.91E+00	Neutral
146	p.Asp146Leu			8863	2148	4.13	4.09					-1.92E+00	Neutral			-1.92E+00	Neutral
146	p.Asp146Asp	Synonymous		14629	3829	6.82	7.30					-1.06E+00	Neutral			-1.06E+00	Neutral
146	p.Asp146Glu			11304	2581	5.27	4.92					-7.22E-01	Neutral			-7.22E-01	Neutral
146	p.Asp146Ala			9378	2111	4.37	4.02					-1.04E+00	Neutral			-1.04E+00	Neutral

146	p.Asp146Gly		10422	2344	4.86	4.47					-7.93E-01	Neutral			-7.93E-01	Neutral
146	p.Asp146Val		10670	2408	4.97	4.59					-7.66E-01	Neutral			-7.66E-01	Neutral
146	p.Asp146Tyr		8216	2149	3.83	4.09					-3.39E+00	Neutral			-3.39E+00	Neutral
146	p.Asp146Cys		8017	2125	3.74	4.05					-3.78E+00	Neutral			-3.78E+00	Neutral
146	p.Asp146Trp		11676	2611	5.44	4.97					-5.58E-01	Neutral			-5.58E-01	Neutral
146	p.Asp146Phe		12657	3420	5.90	6.52					-1.81E+00	Neutral			-1.81E+00	Neutral
147	p.Ala147Asn		9204	2354	5.80	6.05					-9.33E-01	Neutral			-9.33E-01	Neutral
147	p.Ala147Lys		5763	1279	3.63	3.29					-1.03E+00	Neutral			-1.03E+00	Neutral
147	p.Ala147Thr		8653	2165	5.45	5.56					-9.22E-01	Neutral			-9.22E-01	Neutral
147	p.Ala147Arg		9200	2032	5.79	5.22					-2.64E-01	Neutral			-2.64E-01	Neutral
147	p.Ala147Ser		4918	1083	3.10	2.78					-1.41E+00	Neutral			-1.41E+00	Neutral
147	p.Ala147Ile		12599	3435	7.93	8.82					-6.82E-01	Neutral			-6.82E-01	Neutral
147	p.Ala147Met		8368	2192	5.27	5.63					-1.38E+00	Neutral			-1.38E+00	Neutral
147	p.Ala147His		8991	1948	5.66	5.00					-2.35E-01	Neutral			-2.35E-01	Neutral
147	p.Ala147Gln		8751	3013	5.51	7.74					-5.59E+00	Neutral			-5.59E+00	Neutral
147	p.Ala147Pro		4007	861	2.52	2.21					-1.86E+00	Neutral			-1.86E+00	Neutral
147	p.Ala147Leu		7838	2032	4.94	5.22					-1.49E+00	Neutral			-1.49E+00	Neutral
147	p.Ala147Asp		7218	1972	4.55	5.07					-2.43E+00	Neutral			-2.43E+00	Neutral
147	p.Ala147Glu		10401	2371	6.55	6.09					-2.41E-01	Neutral			-2.41E-01	Neutral
147	p.Ala147Ala	Synonymous	7509	1827	4.73	4.69					-1.06E+00	Neutral			-1.06E+00	Neutral
147	p.Ala147Gly		7263	1725	4.57	4.43					-9.65E-01	Neutral			-9.65E-01	Neutral
147	p.Ala147Val		6913	1751	4.35	4.50					-1.68E+00	Neutral			-1.68E+00	Neutral
147	p.Ala147Tyr		7642	1659	4.81	4.26					-4.02E-01	Neutral			-4.02E-01	Neutral
147	p.Ala147Cys		6487	1451	4.08	3.73					-8.15E-01	Neutral			-8.15E-01	Neutral
147	p.Ala147Trp		7819	1773	4.92	4.55					-5.51E-01	Neutral			-5.51E-01	Neutral
147	p.Ala147Phe		9259	2010	5.83	5.16					-2.17E-01	Neutral			-2.17E-01	Neutral
148	p.Ala148Asn		5500	1127	5.24	5.45					-6.43E+00	Indeterminate			-6.43E+00	Indeterminate
148	p.Ala148Lys		5191	1606	4.94	7.77					-2.64E+01	Indeterminate			-2.64E+01	Indeterminate
148	p.Ala148Thr	Benign	4478	636	4.27	3.08					-1.32E+00	Neutral			-1.32E+00	Neutral
148	p.Ala148Arg		5505	1227	5.24	5.94					-8.95E+00	Indeterminate			-8.95E+00	Indeterminate
148	p.Ala148Ser		5604	937	5.34	4.53					-2.30E+00	Neutral			-2.30E+00	Neutral
148	p.Ala148Ile		5331	951	5.08	4.60					-3.57E+00	Neutral			-3.57E+00	Neutral
148	p.Ala148Met		5054	1202	4.81	5.81					-1.25E+01	Indeterminate			-1.25E+01	Indeterminate
148	p.Ala148His		4976	975	4.74	4.72					-6.12E+00	Indeterminate			-6.12E+00	Indeterminate
148	p.Ala148Gln		4007	815	3.82	3.94					-9.43E+00	Indeterminate			-9.43E+00	Indeterminate
148	p.Ala148Pro		5297	975	5.05	4.72					-4.20E+00	Neutral			-4.20E+00	Neutral
148	p.Ala148Leu		5329	998	5.08	4.83					-4.52E+00	Neutral			-4.52E+00	Neutral
148	p.Ala148Asp		7534	1486	7.18	7.19					-3.31E+00	Neutral			-3.31E+00	Neutral
148	p.Ala148Glu		5527	1188	5.26	5.75					-7.75E+00	Indeterminate			-7.75E+00	Indeterminate
148	p.Ala148Ala	Synonymous	6364	985	6.06	4.76					-1.06E+00	Neutral			-1.06E+00	Neutral
148	p.Ala148Gly		5784	901	5.51	4.36					-1.38E+00	Neutral			-1.38E+00	Neutral
148	p.Ala148Val		5833	1397	5.56	6.76					-1.08E+01	Indeterminate			-1.08E+01	Indeterminate
148	p.Ala148Tyr		5919	862	5.64	4.17					-8.07E-01	Neutral			-8.07E-01	Neutral
148	p.Ala148Cys		4878	497	4.65	2.40					-3.84E-02	Neutral			-3.84E-02	Neutral
148	p.Ala148Trp		1553	307	1.48	1.49					-2.33E+01	Indeterminate			-2.33E+01	Indeterminate
148	p.Ala148Phe		5316	1600	5.06	7.74					-2.40E+01	Indeterminate			-2.40E+01	Indeterminate
149	p.Glu149Asn		11526	2539	5.21	4.39					-2.13E-01	Neutral			-2.13E-01	Neutral
149	p.Glu149Lys		12918	3636	5.84	6.28					-1.24E+00	Neutral			-1.24E+00	Neutral
149	p.Glu149Thr		8983	2667	4.06	4.61					-3.47E+00	Neutral			-3.47E+00	Neutral
149	p.Glu149Arg		11953	2775	5.40	4.79					-3.18E-01	Neutral			-3.18E-01	Neutral
149	p.Glu149Ser		7632	2377	3.45	4.11					-5.62E+00	Neutral			-5.62E+00	Neutral
149	p.Glu149Ile		9830	2957	4.44	5.11					-3.18E+00	Neutral			-3.18E+00	Neutral
149	p.Glu149Met		8397	1815	3.79	3.14					-4.83E-01	Neutral			-4.83E-01	Neutral
149	p.Glu149His		9745	2478	4.40	4.28					-1.17E+00	Neutral			-1.17E+00	Neutral
149	p.Glu149Gln		14693	3524	6.64	6.09					-2.22E-01	Neutral			-2.22E-01	Neutral
149	p.Glu149Pro		18087	4495	8.17	7.77					-1.56E-01	Neutral			-1.56E-01	Neutral
149	p.Glu149Leu		19825	4976	8.96	8.60					-1.24E-01	Neutral			-1.24E-01	Neutral
149	p.Glu149Asp		6538	1538	2.95	2.66					-1.68E+00	Neutral			-1.68E+00	Neutral
149	p.Glu149Glu	Synonymous	12702	3479	5.74	6.01					-1.06E+00	Neutral			-1.06E+00	Neutral
149	p.Glu149Ala		9398	2578	4.25	4.45					-2.07E+00	Neutral			-2.07E+00	Neutral
149	p.Glu149Gly		10131	2730	4.58	4.72					-1.58E+00	Neutral			-1.58E+00	Neutral
149	p.Glu149Val		11244	3057	5.08	5.28					-1.34E+00	Neutral			-1.34E+00	Neutral
149	p.Glu149Tyr		13264	3705	5.99	6.40					-1.10E+00	Neutral			-1.10E+00	Neutral
149	p.Glu149Cys		7128	1678	3.22	2.90					-1.40E+00	Neutral			-1.40E+00	Neutral
149	p.Glu149Trp		10289	2823	4.65	4.88					-1.72E+00	Neutral			-1.72E+00	Neutral
149	p.Glu149Phe		7043	2050	3.18	3.54					-4.67E+00	Neutral			-4.67E+00	Neutral
150	p.Gly150Asn		7889	2318	5.48	5.15					-2.17E-01	Neutral			-2.17E-01	Neutral
150	p.Gly150Lys		5103	1727	3.55	3.84					-2.08E+00	Neutral			-2.08E+00	Neutral
150	p.Gly150Thr		5460	1724	3.79	3.83					-1.15E+00	Neutral			-1.15E+00	Neutral
150	p.Gly150Arg		10512	3556	7.31	7.91					-3.40E-01	Neutral			-3.40E-01	Neutral
150	p.Gly150Ser		5701	1862	3.96	4.14					-1.31E+00	Neutral			-1.31E+00	Neutral
150	p.Gly150Ile		9376	2842	6.52	6.32					-1.65E-01	Neutral			-1.65E-01	Neutral
150	p.Gly150Met		8873	3128	6.17	6.95					-7.85E-01	Neutral			-7.85E-01	Neutral
150	p.Gly150His		6463	2210	4.49	4.91					-1.35E+00	Neutral			-1.35E+00	Neutral
150	p.Gly150Gln		4578	1412	3.18	3.14					-1.46E+00	Neutral			-1.46E+00	Neutral
150	p.Gly150Pro		7208	2368	5.01	5.26					-7.75E-01	Neutral			-7.75E-01	Neutral
150	p.Gly150Leu		6458	1932	4.49	4.30					-4.84E-01	Neutral			-4.84E-01	Neutral
150	p.Gly150Asp		4629	1476	3.22	3.28					-1.76E+00	Neutral			-1.76E+00	Neutral
150	p.Gly150Glu		7971	2314	5.54	5.14					-1.85E-01	Neutral			-1.85E-01	Neutral
150	p.Gly150Ala		8197	2338	5.70	5.20					-1.38E-01	Neutral			-1.38E-01	Neutral
150	p.Gly150Gly	Synonymous	6435	2123	4.47	4.72					-1.06E+00	Neutral			-1.06E+00	Neutral
150	p.Gly150Val		6648	2121	4.62	4.72					-7.61E-01	Neutral			-7.61E-01	Neutral
150	p.Gly150Tyr		9875	2976	6.86	6.62					-1.28E-01	Neutral			-1.28E-01	Neutral
150	p.Gly150Cys		9027	2296	6.27	5.10					-2.12E-02	Neutral			-2.12E-02	Neutral
150	p.Gly150Trp		6653	2013	4.62	4.48					-4.90E-01	Neutral			-4.90E-01	Neutral
150	p.Gly150Phe		6845	2245	4.76	4.99					-8.74E-01	Neutral			-8.74E-01	Neutral
151	p.Pro151Asn		7141	5866	5.72	6.09					-7.22E-01	Neutral			-7.22E-01	Neutral
151	p.Pro151Lys		10023	7533	8.03	7.83					-1.02E-01	Neutral			-1.02E-01	Neutral
151	p.Pro151Thr		4705	3885	3.77	4.04					-1.96E+00	Neutral			-1.96E+00	Neutral
151	p.Pro151Arg		4020	2684	3.22	2.79					-6.32E-01	Neutral			-6.32E-01	Neutral
151	p.Pro151Ser		1092	898	0.87	0.93					-1.55E+01	Indeterminate			-1.55E+01	Indeterminate
151	p.Pro151Ile		7374	5843	5.91	6.07					-4.90E-01	Neutral			-4.90E-01	Neutral
151	p.Pro151Met		10441	7670	8.36	7.97					-6.54E-02	Neutral			-6.54E-02	Neutral
151	p.Pro151His		7271	6167	5.82	6.41					-8.83E-01	Neutral			-8.83E-01	Neutral
151	p.Pro151Gln		7412	5338	5.94	5.55					-1.93E-01	Neutral			-1.93E-01	Neutral
151	p.Pro151Pro	Synonymous	5643	4511	4.52	4.69					-1.06E+00	Neutral			-1.06E+00	Neutral
151	p.Pro151Leu		3192	2581	2.56	2.68					-3.56E+00	Neutral			-3.56E+00	Neutral
151	p.Pro151Asp		3871	3149	3.10	3.27					-2.62E+00	Neutral			-2.62E+00	Neutral
151	p.Pro151Glu		7344	6204	5.88	6.45					-8.35E-01	Neutral			-8.35E-01	Neutral
151	p.Pro151Ala		5061	4390	4.05	4.56					-2.27E+00	Neutral			-2.27E+00	Neutral
151	p.Pro151Gly		2399	2158	1.92	2.24					-8.60E+00	Indeterminate			-8.60E+00	Indeterminate
151	p.Pro151Val		8770	6632	7.02	6.89					-1.79E-01	Neutral			-1.79E-01	Neutral
151	p.Pro151Tyr		10716	7570	8.58	7.87					-3.50E-02	Neutral			-3.50E-02	Neutral
151	p.Pro151Cys		5307	3759	4.25	3.91					-4.77E-01	Neutral			-4.77E-01	Neutral
151	p.Pro151Trp		6639	4694	5.32	4.88					-2.33E-01	Neutral			-2.33E-01	Neutral
151	p.Pro151Phe		6424	4716	5.15	4.90					-3.72E-01	Neutral			-3.72E-01	Neutral
152	p.Ser152Asn		997	2607	2.80	2.66					-3.22E+00	Neutral			-3.22E+00	Neutral
152	p.Ser152Lys		1636	4727	4.59	4.82					-2.25E+00	Neutral			-2.25E+00	Neutral
152	p.Ser152Thr		1526	4399	4.28	4.48					-2.54E+00	Neutral			-2.54E+00	Neutral
152	p.Ser152Arg		1486	3962	4.17	4.04					-1.68E+00	Neutral			-1.68E+00	Neutral
152	p.Ser152Ser	Synonymous	2605	7820	7.31	7.97					-1.06E+00	Neutral			-1.06E+00	Neutral
152	p.Ser152Ile		1530	3475	4.29	3.54					-4.71E-01	Neutral			-4.71E-01	Neutral
152	p.Ser152Met		1280	3711	3.59	3.78					-3.57E+00	Neutral			-3.57E+00	Neutral
152	p.Ser152His		2226	5927	6.25	6.04					-6.31E-01	Neutral			-6.31E-01	Neutral
152	p.Ser152Gln		2013	4802	5.65	4.90					-3.20E-01	Neutral			-3.20E-01	Neutral
152	p.Ser152Pro		1571	4606	4.41	4.70					-2.64E+00	Neutral			-2.64E+00	Neutral
152	p.Ser152Leu		1380	3970	3.87	4.05					-3.01E+00	Neutral			-3.01E+00	Neutral
152	p.Ser152Asp		2675	7145	7.51	7.28					-3.81E-01	Neutral			-3.81E-01	Neutral
152	p.Ser152Glu		1732	4745	4.86	4.84					-1.44E+00	Neutral			-1.44E+00	Neutral
152	p.Ser152Ala		1609	5022	4.52	5.12					-3.53E+00	Neutral			-3.53E+00	Neutral
152	p.Ser152Gly		1064	3104	2.99	3.16					-4.94E+00	Neutral			-4.94E+00	Neutral
152	p.Ser152Val		2808	8240	7.88	8.40					-7.41E-01	Neutral			-7.41E-01	Neutral
152	p.Ser152Tyr		1351	4459	3.79	4.55					-6.00E+00	Indeterminate			-6.00E+00	Indeterminate
152	p.Ser152Cys		2129	4765	5.98	4.86					-1.37E-01	Neutral			-1.37E-01	Neutral
152	p.Ser152Trp		2066	5505	5.80	5.61					-7.73E-01	Neutral			-7.73E-01	Neutral
152	p.Ser152Phe		1942	5105	5.45	5.20					-8.17E-01	Neutral			-8.17E-01	Neutral
153	p.Asp153Asn		6218	2873	4.13	4.91	8856	2743	4.08	3.33	-5.02E+00	Neutral	-2.25E-01	Neutral	-3.03E+00	Neutral
153	p.Asp153Lys		8629	2960	5.73	5.06	12644	6300	5.83	7.65	-3.51E-01	Neutral	-3.16E+00	Neutral	-1.73E+00	Neutral
153	p.Asp153Thr		7168	3671	4.76	6.28	10937	4515	5.04	5.48	-6.40E+00	Indeterminate	-1.38E+00	Neutral	-5.10E+00	Neutral
153	p.Asp153Arg		7408	3213	4.92	5.49	11470	5904	5.29	7.17	-2.74E+00	Neutral	-4.37E+00	Neutral	-4.54E+00	Neutral
153	p.Asp153Ser		8767	2503	5.82	4.28	12287	3331	5.67	4.05	-4.19E-02	Neutral	-1.01E-02	Neutral	-9.15E-04	Neutral
153	p.Asp153Ile		7764	2451	5.16	4.19	11706	5110	5.40	6.21	-2.19E-01	Neutral	-1.71E+00	Neutral	-7.04E-01	Neutral
153	p.Asp153Met		8272	3776	5.49	6.46	12502	4304	5.77	5.23	-2.96E+00	Neutral	-2.06E-01	Neutral	-1.49E+00	Neutral
153	p.Asp153His		7310	3413	4.86	5.84	10786	3711	4.97	4.51	-4.10E+00	Neutral	-3.33E-01	Neutral	-2.40E+00	Neutral
153	p.Asp153Gln		8065	3909	5.36	6.68	11217	3993	5.17	4.85	-4.19E+00	Neutral	-4.04E-01	Neutral	-2.53E+00	Neutral
153	p.Asp153Pro		5129	1214	3.41	2.08	7233	1428	3.34	1.73	-3.69E-02	Neutral	-9.05E-04	Neutral	-4.86E-04	Neutral
153	p.Asp153Leu		8094	3783	5.38	6.47	11872	4179	5.48	5.08	-3.48E+00	Neutral	-3.05E-01	Neutral	-1.93E+00	Neutral
153	p.Asp153Asp	Synonymous	6009	2091	3.99	3.58	9161	3435	4.23	4.17	-1.06E+00	Neutral	-1.06E+00	Neutral	-8.18E-01	Neutral
153	p.Asp153Glu		7756	3190	5.15	5.46	10546	3242	4.86	3.94	-1.84E+00	Neutral	-1.10E-01	Neutral	-7.16E-01	Neutral

153	p.Asp153Ala		8429	1977	5.60	3.38	11857	4363	5.47	5.30	-2.49E-03	Neutral	-4.61E-01	Neutral	-6.16E-02	Neutral
153	p.Asp153Gly		7582	1910	5.04	3.27	11395	2748	5.26	3.34	-1.41E-02	Neutral	-2.20E-03	Neutral	-9.11E-05	Neutral
153	p.Asp153Val		6468	2574	4.30	4.40	8833	3958	4.07	4.81	-2.17E+00	Neutral	-3.38E+00	Neutral	-3.27E+00	Neutral
153	p.Asp153Tyr		6325	2136	4.20	3.65	8714	4339	4.02	5.27	-7.45E-01	Neutral	-5.68E+00	Neutral	-3.98E+00	Neutral
153	p.Asp153Cys		8817	4328	5.86	7.40	12054	4932	5.56	5.99	-3.86E+00	Neutral	-1.03E+00	Neutral	-2.76E+00	Neutral
153	p.Asp153Trp		8197	3569	5.44	6.10	11348	5242	5.23	6.37	-2.32E+00	Neutral	-2.54E+00	Neutral	-2.73E+00	Neutral
153	p.Asp153Phe		8150	2936	5.41	5.02	11400	4560	5.26	5.54	-6.37E-01	Neutral	-1.00E+00	Neutral	-5.44E-01	Neutral
154	p.Ile154Asn		2296	1516	4.81	5.02					-4.12E+00	Neutral			-4.12E+00	Neutral
154	p.Ile154Lys		2910	1596	6.10	5.29					-8.25E-01	Neutral			-8.25E-01	Neutral
154	p.Ile154Thr		2308	1518	4.84	5.03					-4.01E+00	Neutral			-4.01E+00	Neutral
154	p.Ile154Arg		3084	1930	6.46	6.40					-1.80E+00	Neutral			-1.80E+00	Neutral
154	p.Ile154Ser		2108	1096	4.42	3.63					-1.22E+00	Neutral			-1.22E+00	Neutral
154	p.Ile154Ile	Synonymous	3477	2093	7.29	6.94					-1.06E+00	Neutral			-1.06E+00	Neutral
154	p.Ile154Met		2376	1476	4.98	4.89					-2.85E+00	Neutral			-2.85E+00	Neutral
154	p.Ile154His		1658	1126	3.47	3.73					-7.49E+00	Indeterminate			-7.49E+00	Indeterminate
154	p.Ile154Gln		2231	1345	4.67	4.46					-2.71E+00	Neutral			-2.71E+00	Neutral
154	p.Ile154Pro		1218	831	2.55	2.75					-1.12E+01	Indeterminate			-1.12E+01	Indeterminate
154	p.Ile154Leu		2537	1415	5.32	4.69					-1.30E+00	Neutral			-1.30E+00	Neutral
154	p.Ile154Asp		2677	1599	5.61	5.30					-1.80E+00	Neutral			-1.80E+00	Neutral
154	p.Ile154Glu		3009	1926	6.30	6.38					-2.16E+00	Neutral			-2.16E+00	Neutral
154	p.Ile154Ala		1684	1283	3.53	4.25					-1.13E+01	Indeterminate			-1.13E+01	Indeterminate
154	p.Ile154Gly		2477	1294	5.19	4.29					-8.56E-01	Neutral			-8.56E-01	Neutral
154	p.Ile154Val		2522	1819	5.28	6.03					-5.37E+00	Neutral			-5.37E+00	Neutral
154	p.Ile154Tyr		1981	1370	4.15	4.54					-6.33E+00	Indeterminate			-6.33E+00	Indeterminate
154	p.Ile154Cys		1607	1097	3.37	3.64					-7.96E+00	Indeterminate			-7.96E+00	Indeterminate
154	p.Ile154Trp		2865	2125	6.00	7.04					-5.04E+00	Neutral			-5.04E+00	Neutral
154	p.Ile154Phe		2701	1718	5.66	5.69					-2.57E+00	Neutral			-2.57E+00	Neutral
155	p.Pro155Asn		7516	1212	4.52	4.44					-1.38E+00	Neutral			-1.38E+00	Neutral
155	p.Pro155Lys		6712	1306	4.04	4.79					-4.81E+00	Neutral			-4.81E+00	Neutral
155	p.Pro155Thr		9334	283	5.62	1.04					0.00E+00	Neutral			0.00E+00	Neutral
155	p.Pro155Arg		9747	1439	5.87	5.28					-3.44E-01	Neutral			-3.44E-01	Neutral
155	p.Pro155Ser		8082	2822	4.86	10.35					-2.83E+01	Indeterminate			-2.82E+01	Indeterminate
155	p.Pro155Ile		7620	1707	4.59	6.26					-7.35E+00	Indeterminate			-7.35E+00	Indeterminate
155	p.Pro155Met		8315	793	5.00	2.91					-1.94E-03	Neutral			-1.94E-03	Neutral
155	p.Pro155His		9630	2577	5.80	9.45					-1.07E+01	Indeterminate			-1.07E+01	Indeterminate
155	p.Pro155Gln		7547	572	4.54	2.10					-3.92E-05	Neutral			-3.92E-05	Neutral
155	p.Pro155Pro	Synonymous	8846	1450	5.32	5.32					-1.06E+00	Neutral			-1.06E+00	Neutral
155	p.Pro155Leu		12220	1003	7.35	3.68					-6.49E-06	Neutral			-6.49E-06	Neutral
155	p.Pro155Asp		9525	3729	5.73	13.67					-3.28E+01	Indeterminate			-3.20E+01	Indeterminate
155	p.Pro155Glu		8909	797	5.36	2.92					-3.84E-04	Neutral			-3.84E-04	Neutral
155	p.Pro155Ala		4663	140	2.81	0.51					-3.27E-14	Neutral			0.00E+00	Neutral
155	p.Pro155Gly		8335	970	5.02	3.56					-4.22E-02	Neutral			-4.22E-02	Neutral
155	p.Pro155Val		8856	2882	5.33	10.57					-2.16E+01	Indeterminate			-2.16E+01	Indeterminate
155	p.Pro155Tyr		9258	1066	5.57	3.91					-2.25E-02	Neutral			-2.25E-02	Neutral
155	p.Pro155Cys		7343	471	4.42	1.73					-1.03E-06	Neutral			-1.03E-06	Neutral
155	p.Pro155Trp		7079	1840	4.26	6.75					-1.37E+01	Indeterminate			-1.37E+01	Indeterminate
155	p.Pro155Phe		6611	211	3.98	0.77					-5.77E-15	Neutral			0.00E+00	Neutral
156	p.Asp156Asn		5066	1583	4.64	4.64					-3.19E+00	Neutral			-3.19E+00	Neutral
156	p.Asp156Lys		2453	728	2.25	2.13					-7.54E+00	Indeterminate			-7.54E+00	Indeterminate
156	p.Asp156Thr		7734	2477	7.09	7.26					-1.63E+00	Neutral			-1.63E+00	Neutral
156	p.Asp156Arg		3732	1122	3.42	3.29					-4.36E+00	Neutral			-4.36E+00	Neutral
156	p.Asp156Ser		8975	2716	8.23	7.96					-7.69E-01	Neutral			-7.69E-01	Neutral
156	p.Asp156Ile		4671	1355	4.28	3.97					-2.46E+00	Neutral			-2.46E+00	Neutral
156	p.Asp156Met		5697	1689	5.22	4.95					-1.90E+00	Neutral			-1.90E+00	Neutral
156	p.Asp156His		3364	981	3.08	2.87					-4.44E+00	Neutral			-4.44E+00	Neutral
156	p.Asp156Gln		4870	1727	4.46	5.06					-6.15E+00	Indeterminate			-6.15E+00	Indeterminate
156	p.Asp156Pro		7585	2236	6.95	6.55					-9.64E-01	Neutral			-9.64E-01	Neutral
156	p.Asp156Leu		5547	1799	5.08	5.27					-3.31E+00	Neutral			-3.31E+00	Neutral
156	p.Asp156Asp	Synonymous	7593	2269	6.96	6.65					-1.06E+00	Neutral			-1.06E+00	Neutral
156	p.Asp156Glu		6739	2108	6.18	6.17					-1.87E+00	Neutral			-1.87E+00	Neutral
156	p.Asp156Ala		4389	1665	4.02	4.88					-9.22E+00	Indeterminate			-9.22E+00	Indeterminate
156	p.Asp156Gly		5406	1778	4.96	5.21					-3.71E+00	Neutral			-3.71E+00	Neutral
156	p.Asp156Val		5781	1953	5.30	5.72					-3.79E+00	Neutral			-3.79E+00	Neutral
156	p.Asp156Tyr		4106	1384	3.76	4.05					-6.31E+00	Indeterminate			-6.31E+00	Indeterminate
156	p.Asp156Cys		3646	1198	3.34	3.51					-6.69E+00	Indeterminate			-6.69E+00	Indeterminate
156	p.Asp156Trp		4783	1291	4.38	3.78					-1.51E+00	Neutral			-1.51E+00	Neutral
156	p.Asp156Phe		6952	2080	6.37	6.09					-1.31E+00	Neutral			-1.31E+00	Neutral
