# Supplementary material for: Functional characterization of all CDKN2A missense variants and comparison to in silico models of pathogenicity
Source: bioRxiv. 2025 Feb 11:2023.12.28.573507. Originally published 2023 Dec 28. Preprint. [Version 3] doi: 10.1101/2023.12.28.573507 (PMC10793438; doi:10.1101/2023.12.28.573507)
Supplement: Supplement 5 [file media-5.pdf]

Appendix 1-table 5. Day of confluency by experiment and residue.

Residue	Exp_1	Exp_2
1	32	
2	31	
3	33	
4	31	
5	29	
6	29	
7	33	
8	29	
9	25	
10	29	
11	25	
12	34	
13	33	
14	32	
15	29	30
16	21	
17	23	
18	29	
19	30	
20	23	17
21	17	
22	29	
23	23	
24	27	25
25	30	
26	33	
27	31	30
28	22	
29	29	
30	27	
31	29	
32	16	20
33	33	
34	34	30
35	25	25
36	28	
37	30	
38	22	
39	24	
40	28	
41	30	
42	20	
43	30	
44	32	
45	31	30
46	27	
47	31	
48	16	
49	23	

50	22	
51	23	
52	30	
53	25	
54	33	
55	21	
56	27	
57	30	31
58	27	
59	21	
60	21	
61	28	
62	33	33
63	20	
64	28	
65	23	
66	23	
67	27	
68	20	
69	31	
70	26	
71	20	
72	29	28
73	27	29
74	21	23
75	27	28
76	27	
77	28	
78	30	
79	29	
80	27	
81	20	
82	23	
83	20	
84	23	
85	30	
86	20	
87	22	
88	29	
89	19	
90	28	
91	28	
92	30	
93	25	
94	21	
95	34	
96	34	30
97	22	
98	30	
99	27	
100	23	
101	23	
102	20	
103	29	

104	28	
105	27	
106	30	28
107	33	30
108	18	
109	28	30
110	38	
111	24	
112	27	
113	38	35
114	17	
115	33	
116	32	
117	30	
118	35	
119	38	
120	29	30
121	28	
122	27	27
123	29	27
124	30	30
125	32	30
126	23	
127	30	
128	29	
129	30	
130	25	
131	31	
132	30	
133	38	
134	32	28
135	38	
136	40	35
137	40	
138	40	
139	32	30
140	35	
141	33	
142	30	
143	32	
144	31	
145	30	
146	35	
147	29	
148	31	
149	30	
150	31	
151	32	
152	29	
153	31	28
154	30	
155	32	
156	30	
