# Supplementary material for: Functional characterization of all CDKN2A missense variants and comparison to in silico models of pathogenicity
Source: bioRxiv. 2025 Feb 11:2023.12.28.573507. Originally published 2023 Dec 28. Preprint. [Version 3] doi: 10.1101/2023.12.28.573507 (PMC10793438; doi:10.1101/2023.12.28.573507)
Supplement: Supplement 6 [file media-6.pdf]

Appendix 1-table 6. Normalized fold change for all possible *CDKN2A* missense and synonymous variants.

Residue	Variant	Normalized fold change_1	Log2 normalized fold change_1	Classification_1	Normalized fold change_2	Log2 normalized fold change_2	Classification_2	Mean of normalized fold change	Mean of log normalized fold change	Classification_ merged
1	p.Met1Asn	0.92	-0.12	Neutral				0.92	-0.12	Neutral
1	p.Met1Lys	0.82	-0.29	Neutral				0.82	-0.29	Neutral
1	p.Met1Thr	0.94	-0.09	Neutral				0.94	-0.09	Neutral
1	p.Met1Arg	0.79	-0.33	Neutral				0.79	-0.33	Neutral
1	p.Met1Ser	0.92	-0.12	Neutral				0.92	-0.12	Neutral
1	p.Met1Ile	0.92	-0.12	Neutral				0.92	-0.12	Neutral
1	p.Met1Met	1.00	0.00	Neutral				1.00	0.00	Neutral
1	p.Met1His	1.01	0.02	Neutral				1.01	0.02	Neutral
1	p.Met1Gln	0.96	-0.05	Neutral				0.96	-0.05	Neutral
1	p.Met1Pro	0.87	-0.20	Neutral				0.87	-0.20	Neutral
1	p.Met1Leu	0.96	-0.06	Neutral				0.96	-0.06	Neutral
1	p.Met1Asp	0.86	-0.22	Neutral				0.86	-0.22	Neutral
1	p.Met1Glu	0.97	-0.04	Neutral				0.97	-0.04	Neutral
1	p.Met1Ala	0.90	-0.16	Neutral				0.90	-0.16	Neutral
1	p.Met1Gly	0.94	-0.09	Neutral				0.94	-0.09	Neutral
1	p.Met1Val	1.61	0.69	Indeterminate				1.61	0.69	Indeterminate
1	p.Met1Tyr	0.92	-0.13	Neutral				0.92	-0.13	Neutral
1	p.Met1Cys	0.90	-0.15	Neutral				0.90	-0.15	Neutral
1	p.Met1Trp	0.92	-0.12	Neutral				0.92	-0.12	Neutral
1	p.Met1Phe	0.97	-0.04	Neutral				0.97	-0.04	Neutral
2	p.Glu2Asn	0.20	-2.29	Neutral				0.20	-2.29	Neutral
2	p.Glu2Lys	0.39	-1.35	Neutral				0.39	-1.35	Neutral
2	p.Glu2Thr	0.41	-1.27	Neutral				0.41	-1.27	Neutral
2	p.Glu2Arg	0.21	-2.22	Neutral				0.21	-2.22	Neutral
2	p.Glu2Ser	0.64	-0.65	Neutral				0.64	-0.65	Neutral
2	p.Glu2Ile	0.24	-2.03	Neutral				0.24	-2.03	Neutral
2	p.Glu2Met	0.57	-0.81	Neutral				0.57	-0.81	Neutral
2	p.Glu2His	0.40	-1.34	Neutral				0.40	-1.34	Neutral
2	p.Glu2Gln	0.27	-1.90	Neutral				0.27	-1.90	Neutral
2	p.Glu2Pro	0.31	-1.70	Neutral				0.31	-1.70	Neutral
2	p.Glu2Leu	0.25	-2.02	Neutral				0.25	-2.02	Neutral
2	p.Glu2Asp	0.15	-2.75	Neutral				0.15	-2.75	Neutral
2	p.Glu2Glu	1.00	0.00	Neutral				1.00	0.00	Neutral
2	p.Glu2Ala	0.61	-0.71	Neutral				0.61	-0.71	Neutral
2	p.Glu2Gly	0.36	-1.49	Neutral				0.36	-1.49	Neutral
2	p.Glu2Val	0.23	-2.10	Neutral				0.23	-2.10	Neutral
2	p.Glu2Tyr	0.31	-1.69	Neutral				0.31	-1.69	Neutral
2	p.Glu2Cys	0.64	-0.64	Neutral				0.64	-0.64	Neutral
2	p.Glu2Trp	0.44	-1.18	Neutral				0.44	-1.18	Neutral
2	p.Glu2Phe	0.38	-1.38	Neutral				0.38	-1.38	Neutral
3	p.Pro3Asn	1.38	0.46	Indeterminate				1.38	0.46	Indeterminate
3	p.Pro3Lys	1.48	0.57	Indeterminate				1.48	0.57	Indeterminate
3	p.Pro3Thr	1.39	0.48	Indeterminate				1.39	0.48	Indeterminate
3	p.Pro3Arg	2.22	1.15	Deleterious				2.22	1.15	Deleterious
3	p.Pro3Ser	1.71	0.78	Indeterminate				1.71	0.78	Indeterminate
3	p.Pro3Ile	1.33	0.41	Indeterminate				1.33	0.41	Indeterminate
3	p.Pro3Met	1.69	0.76	Indeterminate				1.69	0.76	Indeterminate
3	p.Pro3His	1.08	0.11	Neutral				1.08	0.11	Neutral
3	p.Pro3Gln	0.74	-0.44	Neutral				0.74	-0.44	Neutral
3	p.Pro3Pro	1.00	0.00	Neutral				1.00	0.00	Neutral
3	p.Pro3Leu	1.52	0.60	Indeterminate				1.52	0.60	Indeterminate
3	p.Pro3Asp	1.68	0.75	Indeterminate				1.68	0.75	Indeterminate
3	p.Pro3Glu	1.15	0.20	Neutral				1.15	0.20	Neutral
3	p.Pro3Ala	1.06	0.09	Neutral				1.06	0.09	Neutral
3	p.Pro3Gly	2.75	1.46	Deleterious				2.75	1.46	Deleterious
3	p.Pro3Val	1.71	0.77	Indeterminate				1.71	0.77	Indeterminate
3	p.Pro3Tyr	1.18	0.23	Neutral				1.18	0.23	Neutral
3	p.Pro3Cys	1.49	0.58	Indeterminate				1.49	0.58	Indeterminate
3	p.Pro3Trp	1.94	0.96	Indeterminate				1.94	0.96	Indeterminate
3	p.Pro3Phe	1.69	0.76	Indeterminate				1.69	0.76	Indeterminate
4	p.Ala4Asn	1.36	0.44	Indeterminate				1.36	0.44	Indeterminate
4	p.Ala4Lys	0.98	-0.02	Neutral				0.98	-0.02	Neutral
4	p.Ala4Thr	2.78	1.48	Deleterious				2.78	1.48	Deleterious
4	p.Ala4Arg	1.07	0.10	Neutral				1.07	0.10	Neutral
4	p.Ala4Ser	2.88	1.53	Deleterious				2.88	1.53	Deleterious
4	p.Ala4Ile	0.76	-0.40	Neutral				0.76	-0.40	Neutral
4	p.Ala4Met	1.45	0.54	Indeterminate				1.45	0.54	Indeterminate
4	p.Ala4His	1.16	0.21	Neutral				1.16	0.21	Neutral
4	p.Ala4Gln	0.77	-0.37	Neutral				0.77	-0.37	Neutral
4	p.Ala4Pro	0.84	-0.25	Neutral				0.84	-0.25	Neutral
4	p.Ala4Leu	0.96	-0.06	Neutral				0.96	-0.06	Neutral
4	p.Ala4Asp	1.10	0.13	Neutral				1.10	0.13	Neutral
4	p.Ala4Glu	0.65	-0.63	Neutral				0.65	-0.63	Neutral
4	p.Ala4Ala	1.00	0.00	Neutral				1.00	0.00	Neutral
4	p.Ala4Gly	1.34	0.42	Indeterminate				1.34	0.42	Indeterminate
4	p.Ala4Val	0.78	-0.36	Neutral				0.78	-0.36	Neutral
4	p.Ala4Tyr	0.90	-0.15	Neutral				0.90	-0.15	Neutral
4	p.Ala4Cys	2.08	1.06	Indeterminate				2.08	1.06	Indeterminate
4	p.Ala4Trp	1.12	0.16	Neutral				1.12	0.16	Neutral
4	p.Ala4Phe	1.42	0.51	Indeterminate				1.42	0.51	Indeterminate
5	p.Ala5Asn	0.80	-0.32	Neutral				0.80	-0.32	Neutral
5	p.Ala5Lys	0.37	-1.44	Neutral				0.37	-1.44	Neutral
5	p.Ala5Thr	1.04	0.05	Neutral				1.04	0.05	Neutral
5	p.Ala5Arg	0.57	-0.81	Neutral				0.57	-0.81	Neutral
5	p.Ala5Ser	2.86	1.52	Deleterious				2.86	1.52	Deleterious
5	p.Ala5Ile	0.31	-1.68	Neutral				0.31	-1.68	Neutral
5	p.Ala5Met	0.87	-0.21	Neutral				0.87	-0.21	Neutral
5	p.Ala5His	1.10	0.14	Neutral				1.10	0.14	Neutral
5	p.Ala5Gln	1.11	0.15	Neutral				1.11	0.15	Neutral
5	p.Ala5Pro	0.86	-0.21	Neutral				0.86	-0.21	Neutral
5	p.Ala5Leu	1.88	0.91	Indeterminate				1.88	0.91	Indeterminate
5	p.Ala5Asp	1.14	0.19	Neutral				1.14	0.19	Neutral
5	p.Ala5Glu	1.37	0.46	Indeterminate				1.37	0.46	Indeterminate
5	p.Ala5Ala	1.00	0.00	Neutral				1.00	0.00	Neutral
5	p.Ala5Gly	0.77	-0.38	Neutral				0.77	-0.38	Neutral
5	p.Ala5Val	0.89	-0.17	Neutral				0.89	-0.17	Neutral

5	p.Ala5Tyr	4.82	2.27	Deleterious	4.82	2.27	Deleterious
5	p.Ala5Cys	1.26	0.34	Indeterminate	1.26	0.34	Indeterminate
5	p.Ala5Trp	2.44	1.29	Deleterious	2.44	1.29	Deleterious
5	p.Ala5Phe	0.90	-0.16	Neutral	0.90	-0.16	Neutral
6	p.Gly6Asn	0.95	-0.07	Neutral	0.95	-0.07	Neutral
6	p.Gly6Lys	1.40	0.48	Indeterminate	1.40	0.48	Indeterminate
6	p.Gly6Thr	1.47	0.56	Indeterminate	1.47	0.56	Indeterminate
6	p.Gly6Arg	4.48	2.16	Deleterious	4.48	2.16	Deleterious
6	p.Gly6Ser	3.45	1.79	Deleterious	3.45	1.79	Deleterious
6	p.Gly6Ile	3.76	1.91	Deleterious	3.76	1.91	Deleterious
6	p.Gly6Met	3.51	1.81	Deleterious	3.51	1.81	Deleterious
6	p.Gly6His	0.72	-0.48	Neutral	0.72	-0.48	Neutral
6	p.Gly6Gln	0.87	-0.20	Neutral	0.87	-0.20	Neutral
6	p.Gly6Pro	2.07	1.05	Indeterminate	2.07	1.05	Indeterminate
6	p.Gly6Leu	1.24	0.32	Indeterminate	1.24	0.32	Indeterminate
6	p.Gly6Asp	1.04	0.06	Neutral	1.04	0.06	Neutral
6	p.Gly6Glu	1.16	0.22	Neutral	1.16	0.22	Neutral
6	p.Gly6Ala	1.05	0.07	Neutral	1.05	0.07	Neutral
6	p.Gly6Gly	1.00	0.00	Neutral	1.00	0.00	Neutral
6	p.Gly6Val	1.00	0.00	Neutral	1.00	0.00	Neutral
6	p.Gly6Tyr	1.01	0.01	Neutral	1.01	0.01	Neutral
6	p.Gly6Cys	1.83	0.87	Indeterminate	1.83	0.87	Indeterminate
6	p.Gly6Trp	1.44	0.53	Indeterminate	1.44	0.53	Indeterminate
6	p.Gly6Phe	1.58	0.66	Indeterminate	1.58	0.66	Indeterminate
7	p.Ser7Asn	0.89	-0.17	Neutral	0.89	-0.17	Neutral
7	p.Ser7Lys	0.91	-0.14	Neutral	0.91	-0.14	Neutral
7	p.Ser7Thr	2.04	1.03	Indeterminate	2.04	1.03	Indeterminate
7	p.Ser7Arg	0.82	-0.29	Neutral	0.82	-0.29	Neutral
7	p.Ser7Ser	1.00	0.00	Neutral	1.00	0.00	Neutral
7	p.Ser7Ile	1.23	0.30	Indeterminate	1.23	0.30	Indeterminate
7	p.Ser7Met	1.18	0.24	Indeterminate	1.18	0.24	Indeterminate
7	p.Ser7His	1.47	0.56	Indeterminate	1.47	0.56	Indeterminate
7	p.Ser7Gln	1.10	0.14	Neutral	1.10	0.14	Neutral
7	p.Ser7Pro	1.26	0.34	Indeterminate	1.26	0.34	Indeterminate
7	p.Ser7Leu	1.80	0.85	Indeterminate	1.80	0.85	Indeterminate
7	p.Ser7Asp	0.99	-0.02	Neutral	0.99	-0.02	Neutral
7	p.Ser7Glu	1.65	0.73	Indeterminate	1.65	0.73	Indeterminate
7	p.Ser7Ala	1.95	0.97	Indeterminate	1.95	0.97	Indeterminate
7	p.Ser7Gly	0.93	-0.10	Neutral	0.93	-0.10	Neutral
7	p.Ser7Val	1.00	0.00	Neutral	1.00	0.00	Neutral
7	p.Ser7Tyr	2.57	1.36	Deleterious	2.57	1.36	Deleterious
7	p.Ser7Cys	1.26	0.33	Indeterminate	1.26	0.33	Indeterminate
7	p.Ser7Trp	1.31	0.39	Indeterminate	1.31	0.39	Indeterminate
7	p.Ser7Phe	1.17	0.22	Neutral	1.17	0.22	Neutral
8	p.Ser8Asn	0.57	-0.81	Neutral	0.57	-0.81	Neutral
8	p.Ser8Lys	1.73	0.79	Indeterminate	1.73	0.79	Indeterminate
8	p.Ser8Thr	0.92	-0.12	Neutral	0.92	-0.12	Neutral
8	p.Ser8Arg	0.69	-0.53	Neutral	0.69	-0.53	Neutral
8	p.Ser8Ser	1.00	0.00	Neutral	1.00	0.00	Neutral
8	p.Ser8Ile	0.72	-0.48	Neutral	0.72	-0.48	Neutral
8	p.Ser8Met	0.56	-0.85	Neutral	0.56	-0.85	Neutral
8	p.Ser8His	0.60	-0.73	Neutral	0.60	-0.73	Neutral
8	p.Ser8Gln	0.67	-0.59	Neutral	0.67	-0.59	Neutral
8	p.Ser8Pro	0.83	-0.26	Neutral	0.83	-0.26	Neutral
8	p.Ser8Leu	0.84	-0.26	Neutral	0.84	-0.26	Neutral
8	p.Ser8Asp	0.73	-0.46	Neutral	0.73	-0.46	Neutral
8	p.Ser8Glu	0.45	-1.15	Neutral	0.45	-1.15	Neutral
8	p.Ser8Ala	0.68	-0.56	Neutral	0.68	-0.56	Neutral
8	p.Ser8Gly	0.82	-0.28	Neutral	0.82	-0.28	Neutral
8	p.Ser8Val	0.71	-0.49	Neutral	0.71	-0.49	Neutral
8	p.Ser8Tyr	0.61	-0.72	Neutral	0.61	-0.72	Neutral
8	p.Ser8Cys	0.52	-0.94	Neutral	0.52	-0.94	Neutral
8	p.Ser8Trp	0.58	-0.79	Neutral	0.58	-0.79	Neutral
8	p.Ser8Phe	0.75	-0.41	Neutral	0.75	-0.41	Neutral
9	p.Met9Asn	1.10	0.14	Neutral	1.10	0.14	Neutral
9	p.Met9Lys	1.21	0.27	Indeterminate	1.21	0.27	Indeterminate
9	p.Met9Thr	1.19	0.26	Indeterminate	1.19	0.26	Indeterminate
9	p.Met9Arg	1.14	0.18	Neutral	1.14	0.18	Neutral
9	p.Met9Ser	1.16	0.22	Neutral	1.16	0.22	Neutral
9	p.Met9Ile	1.18	0.24	Indeterminate	1.18	0.24	Indeterminate
9	p.Met9Met	1.00	0.00	Neutral	1.00	0.00	Neutral
9	p.Met9His	1.05	0.07	Neutral	1.05	0.07	Neutral
9	p.Met9Gln	1.04	0.05	Neutral	1.04	0.05	Neutral
9	p.Met9Pro	1.18	0.24	Neutral	1.18	0.24	Neutral
9	p.Met9Leu	0.92	-0.11	Neutral	0.92	-0.11	Neutral
9	p.Met9Asp	1.16	0.21	Neutral	1.16	0.21	Neutral
9	p.Met9Glu	1.02	0.03	Neutral	1.02	0.03	Neutral
9	p.Met9Ala	1.07	0.10	Neutral	1.07	0.10	Neutral
9	p.Met9Gly	1.10	0.14	Neutral	1.10	0.14	Neutral
9	p.Met9Val	1.25	0.32	Indeterminate	1.25	0.32	Indeterminate
9	p.Met9Tyr	1.00	0.00	Neutral	1.00	0.00	Neutral
9	p.Met9Cys	1.08	0.11	Neutral	1.08	0.11	Neutral
9	p.Met9Trp	1.13	0.17	Neutral	1.13	0.17	Neutral
9	p.Met9Phe	1.14	0.19	Neutral	1.14	0.19	Neutral
10	p.Glu10Asn	1.08	0.11	Neutral	1.08	0.11	Neutral
10	p.Glu10Lys	2.31	1.21	Deleterious	2.31	1.21	Deleterious
10	p.Glu10Thr	1.18	0.24	Neutral	1.18	0.24	Neutral
10	p.Glu10Arg	0.94	-0.09	Neutral	0.94	-0.09	Neutral
10	p.Glu10Ser	1.65	0.72	Indeterminate	1.65	0.72	Indeterminate
10	p.Glu10Ile	1.54	0.62	Indeterminate	1.54	0.62	Indeterminate
10	p.Glu10Met	1.67	0.74	Indeterminate	1.67	0.74	Indeterminate
10	p.Glu10His	2.29	1.20	Deleterious	2.29	1.20	Deleterious
10	p.Glu10Gln	0.93	-0.10	Neutral	0.93	-0.10	Neutral
10	p.Glu10Pro	1.03	0.04	Neutral	1.03	0.04	Neutral
10	p.Glu10Leu	1.15	0.20	Neutral	1.15	0.20	Neutral
10	p.Glu10Asp	1.13	0.17	Neutral	1.13	0.17	Neutral
10	p.Glu10Glu	1.00	0.00	Neutral	1.00	0.00	Neutral
10	p.Glu10Ala	1.77	0.82	Indeterminate	1.77	0.82	Indeterminate
10	p.Glu10Gly	1.04	0.06	Neutral	1.04	0.06	Neutral
10	p.Glu10Val	1.04	0.06	Neutral	1.04	0.06	Neutral
10	p.Glu10Tyr	1.33	0.41	Indeterminate	1.33	0.41	Indeterminate

10	p.Glu10Cys	1.15	0.20	Neutral				1.15	0.20	Neutral
10	p.Glu10Trp	1.01	0.02	Neutral				1.01	0.02	Neutral
10	p.Glu10Phe	1.11	0.15	Neutral				1.11	0.15	Neutral
11	p.Pro11Asn	1.13	0.17	Neutral				1.13	0.17	Neutral
11	p.Pro11Lys	1.28	0.36	Indeterminate				1.28	0.36	Indeterminate
11	p.Pro11Thr	1.44	0.53	Indeterminate				1.44	0.53	Indeterminate
11	p.Pro11Arg	1.08	0.11	Neutral				1.08	0.11	Neutral
11	p.Pro11Ser	1.21	0.27	Indeterminate				1.21	0.27	Indeterminate
11	p.Pro11Ile	1.13	0.18	Neutral				1.13	0.18	Neutral
11	p.Pro11Met	1.07	0.09	Neutral				1.07	0.09	Neutral
11	p.Pro11His	1.12	0.17	Neutral				1.12	0.17	Neutral
11	p.Pro11Gln	0.96	-0.06	Neutral				0.96	-0.06	Neutral
11	p.Pro11Pro	1.00	0.00	Neutral				1.00	0.00	Neutral
11	p.Pro11Leu	1.12	0.16	Neutral				1.12	0.16	Neutral
11	p.Pro11Asp	1.07	0.09	Neutral				1.07	0.09	Neutral
11	p.Pro11Glu	1.01	0.01	Neutral				1.01	0.01	Neutral
11	p.Pro11Ala	1.10	0.14	Neutral				1.10	0.14	Neutral
11	p.Pro11Gly	1.20	0.26	Indeterminate				1.20	0.26	Indeterminate
11	p.Pro11Val	1.09	0.12	Neutral				1.09	0.12	Neutral
11	p.Pro11Tyr	1.22	0.28	Indeterminate				1.22	0.28	Indeterminate
11	p.Pro11Cys	1.01	0.02	Neutral				1.01	0.02	Neutral
11	p.Pro11Trp	1.31	0.39	Indeterminate				1.31	0.39	Indeterminate
11	p.Pro11Phe	1.22	0.29	Indeterminate				1.22	0.29	Indeterminate
12	p.Ser12Asn	0.92	-0.12	Neutral				0.92	-0.12	Neutral
12	p.Ser12Lys	0.71	-0.49	Neutral				0.71	-0.49	Neutral
12	p.Ser12Thr	0.76	-0.39	Neutral				0.76	-0.39	Neutral
12	p.Ser12Arg	0.84	-0.25	Neutral				0.84	-0.25	Neutral
12	p.Ser12Ser	1.00	0.00	Neutral				1.00	0.00	Neutral
12	p.Ser12Ile	0.81	-0.31	Neutral				0.81	-0.31	Neutral
12	p.Ser12Met	0.71	-0.49	Neutral				0.71	-0.49	Neutral
12	p.Ser12His	1.12	0.16	Neutral				1.12	0.16	Neutral
12	p.Ser12Gln	1.15	0.20	Neutral				1.15	0.20	Neutral
12	p.Ser12Pro	0.78	-0.36	Neutral				0.78	-0.36	Neutral
12	p.Ser12Leu	2.35	1.23	Deleterious				2.35	1.23	Deleterious
12	p.Ser12Asp	0.66	-0.59	Neutral				0.66	-0.59	Neutral
12	p.Ser12Glu	0.88	-0.18	Neutral				0.88	-0.18	Neutral
12	p.Ser12Ala	0.85	-0.23	Neutral				0.85	-0.23	Neutral
12	p.Ser12Gly	0.82	-0.29	Neutral				0.82	-0.29	Neutral
12	p.Ser12Val	1.13	0.18	Neutral				1.13	0.18	Neutral
12	p.Ser12Tyr	0.75	-0.41	Neutral				0.75	-0.41	Neutral
12	p.Ser12Cys	1.13	0.18	Neutral				1.13	0.18	Neutral
12	p.Ser12Trp	1.13	0.18	Neutral				1.13	0.18	Neutral
12	p.Ser12Phe	1.11	0.15	Neutral				1.11	0.15	Neutral
13	p.Ala13Asn	0.61	-0.72	Neutral				0.61	-0.72	Neutral
13	p.Ala13Lys	0.71	-0.49	Neutral				0.71	-0.49	Neutral
13	p.Ala13Thr	0.70	-0.51	Neutral				0.70	-0.51	Neutral
13	p.Ala13Arg	0.61	-0.72	Neutral				0.61	-0.72	Neutral
13	p.Ala13Ser	0.63	-0.66	Neutral				0.63	-0.66	Neutral
13	p.Ala13Ile	0.65	-0.61	Neutral				0.65	-0.61	Neutral
13	p.Ala13Met	1.07	0.10	Neutral				1.07	0.10	Neutral
13	p.Ala13His	0.75	-0.41	Neutral				0.75	-0.41	Neutral
13	p.Ala13Gln	0.66	-0.59	Neutral				0.66	-0.59	Neutral
13	p.Ala13Pro	0.53	-0.90	Neutral				0.53	-0.90	Neutral
13	p.Ala13Leu	0.74	-0.43	Neutral				0.74	-0.43	Neutral
13	p.Ala13Asp	0.68	-0.55	Neutral				0.68	-0.55	Neutral
13	p.Ala13Glu	0.64	-0.64	Neutral				0.64	-0.64	Neutral
13	p.Ala13Ala	1.00	0.00	Neutral				1.00	0.00	Neutral
13	p.Ala13Gly	0.57	-0.81	Neutral				0.57	-0.81	Neutral
13	p.Ala13Val	0.70	-0.51	Neutral				0.70	-0.51	Neutral
13	p.Ala13Tyr	1.03	0.04	Neutral				1.03	0.04	Neutral
13	p.Ala13Cys	0.82	-0.29	Neutral				0.82	-0.29	Neutral
13	p.Ala13Trp	0.77	-0.38	Neutral				0.77	-0.38	Neutral
13	p.Ala13Phe	0.94	-0.09	Neutral				0.94	-0.09	Neutral
14	p.Asp14Asn	1.14	0.19	Neutral				1.14	0.19	Neutral
14	p.Asp14Lys	1.10	0.14	Neutral				1.10	0.14	Neutral
14	p.Asp14Thr	0.95	-0.08	Neutral				0.95	-0.08	Neutral
14	p.Asp14Arg	1.63	0.71	Indeterminate				1.63	0.71	Indeterminate
14	p.Asp14Ser	0.92	-0.13	Neutral				0.92	-0.13	Neutral
14	p.Asp14Ile	2.30	1.20	Deleterious				2.30	1.20	Deleterious
14	p.Asp14Met	1.77	0.82	Indeterminate				1.77	0.82	Indeterminate
14	p.Asp14His	0.95	-0.07	Neutral				0.95	-0.07	Neutral
14	p.Asp14Gln	1.12	0.17	Neutral				1.12	0.17	Neutral
14	p.Asp14Pro	2.56	1.36	Deleterious				2.56	1.36	Deleterious
14	p.Asp14Leu	1.80	0.84	Indeterminate				1.80	0.84	Indeterminate
14	p.Asp14Asp	1.00	0.00	Neutral				1.00	0.00	Neutral
14	p.Asp14Glu	1.09	0.12	Neutral				1.09	0.12	Neutral
14	p.Asp14Ala	1.37	0.46	Indeterminate				1.37	0.46	Indeterminate
14	p.Asp14Gly	1.04	0.06	Neutral				1.04	0.06	Neutral
14	p.Asp14Val	2.47	1.31	Deleterious				2.47	1.31	Deleterious
14	p.Asp14Tyr	1.93	0.95	Indeterminate				1.93	0.95	Indeterminate
14	p.Asp14Cys	1.05	0.06	Neutral				1.05	0.06	Neutral
14	p.Asp14Trp	1.87	0.90	Indeterminate				1.87	0.90	Indeterminate
14	p.Asp14Phe	1.93	0.95	Indeterminate				1.93	0.95	Indeterminate
15	p.Trp15Asn	1.51	0.59	Indeterminate	0.73	-0.46	Neutral	1.12	0.16	Neutral
15	p.Trp15Lys	1.37	0.46	Indeterminate	0.72	-0.48	Neutral	1.04	0.06	Neutral
15	p.Trp15Thr	1.65	0.73	Indeterminate	1.03	0.04	Neutral	1.34	0.42	Indeterminate
15	p.Trp15Arg	1.19	0.25	Indeterminate	0.65	-0.62	Neutral	0.92	-0.12	Neutral
15	p.Trp15Ser	1.37	0.46	Indeterminate	0.69	-0.54	Neutral	1.03	0.04	Neutral
15	p.Trp15Ile	1.03	0.05	Neutral	0.99	-0.02	Neutral	1.01	0.01	Neutral
15	p.Trp15Met	1.13	0.17	Neutral	0.93	-0.10	Neutral	1.03	0.04	Neutral
15	p.Trp15His	1.48	0.57	Indeterminate	1.61	0.69	Indeterminate	1.55	0.63	Indeterminate
15	p.Trp15Gln	1.46	0.54	Indeterminate	1.13	0.18	Indeterminate	1.29	0.37	Indeterminate
15	p.Trp15Pro	1.83	0.87	Indeterminate	1.46	0.55	Indeterminate	1.65	0.72	Indeterminate
15	p.Trp15Leu	1.30	0.38	Indeterminate	1.14	0.19	Indeterminate	1.22	0.29	Indeterminate
15	p.Trp15Asp	1.28	0.35	Indeterminate	1.06	0.08	Neutral	1.17	0.23	Neutral
15	p.Trp15Glu	1.09	0.13	Neutral	1.11	0.15	Indeterminate	1.10	0.14	Neutral
15	p.Trp15Ala	1.36	0.44	Indeterminate	0.77	-0.37	Neutral	1.07	0.09	Neutral
15	p.Trp15Gly	1.18	0.24	Neutral	1.46	0.54	Indeterminate	1.32	0.40	Indeterminate
15	p.Trp15Val	1.06	0.08	Neutral	0.62	-0.68	Neutral	0.84	-0.25	Neutral
15	p.Trp15Tyr	1.67	0.74	Indeterminate	0.91	-0.13	Neutral	1.29	0.37	Indeterminate
15	p.Trp15Cys	1.51	0.59	Indeterminate	0.82	-0.28	Neutral	1.17	0.22	Neutral

15	p.Trp15Trp	1.00	0.00	Neutral	1.00	0.00	Neutral	1.00	0.00	Neutral
15	p.Trp15Phe	1.29	0.37	Indeterminate	0.77	-0.37	Neutral	1.03	0.05	Neutral
16	p.Leu16Asn	17.05	4.09	Deleterious				17.05	4.09	Deleterious
16	p.Leu16Lys	24.15	4.59	Deleterious				24.15	4.59	Deleterious
16	p.Leu16Thr	2.31	1.21	Deleterious				2.31	1.21	Deleterious
16	p.Leu16Arg	28.65	4.84	Deleterious				28.65	4.84	Deleterious
16	p.Leu16Ser	12.85	3.68	Deleterious				12.85	3.68	Deleterious
16	p.Leu16Ile	1.34	0.42	Indeterminate				1.34	0.42	Indeterminate
16	p.Leu16Met	1.18	0.24	Indeterminate				1.18	0.24	Indeterminate
16	p.Leu16His	16.82	4.07	Deleterious				16.82	4.07	Deleterious
16	p.Leu16Gln	16.71	4.06	Deleterious				16.71	4.06	Deleterious
16	p.Leu16Pro	15.82	3.98	Deleterious				15.82	3.98	Deleterious
16	p.Leu16Leu	1.00	0.00	Neutral				1.00	0.00	Neutral
16	p.Leu16Asp	24.00	4.59	Deleterious				24.00	4.59	Deleterious
16	p.Leu16Glu	21.71	4.44	Deleterious				21.71	4.44	Deleterious
16	p.Leu16Ala	4.92	2.30	Deleterious				4.92	2.30	Deleterious
16	p.Leu16Gly	19.95	4.32	Deleterious				19.95	4.32	Deleterious
16	p.Leu16Val	2.04	1.03	Indeterminate				2.04	1.03	Indeterminate
16	p.Leu16Tyr	21.66	4.44	Deleterious				21.66	4.44	Deleterious
16	p.Leu16Cys	2.04	1.03	Indeterminate				2.04	1.03	Indeterminate
16	p.Leu16Trp	21.58	4.43	Deleterious				21.58	4.43	Deleterious
16	p.Leu16Phe	1.48	0.56	Indeterminate				1.48	0.56	Indeterminate
17	p.Ala17Asn	1.60	0.68	Indeterminate				1.60	0.68	Indeterminate
17	p.Ala17Lys	36.62	5.19	Deleterious				36.62	5.19	Deleterious
17	p.Ala17Thr	1.13	0.18	Neutral				1.13	0.18	Neutral
17	p.Ala17Arg	39.11	5.29	Deleterious				39.11	5.29	Deleterious
17	p.Ala17Ser	1.14	0.19	Neutral				1.14	0.19	Neutral
17	p.Ala17Ile	1.70	0.77	Indeterminate				1.70	0.77	Indeterminate
17	p.Ala17Met	3.32	1.73	Deleterious				3.32	1.73	Deleterious
17	p.Ala17His	1.14	0.19	Neutral				1.14	0.19	Neutral
17	p.Ala17Gln	3.54	1.82	Deleterious				3.54	1.82	Deleterious
17	p.Ala17Pro	1.10	0.13	Neutral				1.10	0.13	Neutral
17	p.Ala17Leu	6.23	2.64	Deleterious				6.23	2.64	Deleterious
17	p.Ala17Asp	2.99	1.58	Deleterious				2.99	1.58	Deleterious
17	p.Ala17Glu	15.21	3.93	Deleterious				15.21	3.93	Deleterious
17	p.Ala17Ala	1.00	0.00	Neutral				1.00	0.00	Neutral
17	p.Ala17Gly	1.01	0.01	Neutral				1.01	0.01	Neutral
17	p.Ala17Val	1.04	0.06	Neutral				1.04	0.06	Neutral
17	p.Ala17Tyr	4.73	2.24	Deleterious				4.73	2.24	Deleterious
17	p.Ala17Cys	0.76	-0.40	Neutral				0.76	-0.40	Neutral
17	p.Ala17Trp	5.41	2.43	Deleterious				5.41	2.43	Deleterious
17	p.Ala17Phe	3.04	1.61	Deleterious				3.04	1.61	Deleterious
18	p.Thr18Asn	2.54	1.34	Deleterious				2.54	1.34	Deleterious
18	p.Thr18Lys	1.83	0.87	Indeterminate				1.83	0.87	Indeterminate
18	p.Thr18Thr	1.00	0.00	Neutral				1.00	0.00	Neutral
18	p.Thr18Arg	1.81	0.85	Indeterminate				1.81	0.85	Indeterminate
18	p.Thr18Ser	1.45	0.53	Indeterminate				1.45	0.53	Indeterminate
18	p.Thr18Ile	2.08	1.06	Indeterminate				2.08	1.06	Indeterminate
18	p.Thr18Met	2.38	1.25	Deleterious				2.38	1.25	Deleterious
18	p.Thr18His	1.36	0.44	Indeterminate				1.36	0.44	Indeterminate
18	p.Thr18Gln	1.66	0.73	Indeterminate				1.66	0.73	Indeterminate
18	p.Thr18Pro	11.34	3.50	Deleterious				11.34	3.50	Deleterious
18	p.Thr18Leu	2.42	1.28	Deleterious				2.42	1.28	Deleterious
18	p.Thr18Asp	1.66	0.73	Indeterminate				1.66	0.73	Indeterminate
18	p.Thr18Glu	1.46	0.55	Indeterminate				1.46	0.55	Indeterminate
18	p.Thr18Ala	2.46	1.30	Deleterious				2.46	1.30	Deleterious
18	p.Thr18Gly	1.46	0.54	Indeterminate				1.46	0.54	Indeterminate
18	p.Thr18Val	1.50	0.59	Indeterminate				1.50	0.59	Indeterminate
18	p.Thr18Tyr	2.54	1.34	Deleterious				2.54	1.34	Deleterious
18	p.Thr18Cys	1.09	0.13	Neutral				1.09	0.13	Neutral
18	p.Thr18Trp	1.89	0.92	Indeterminate				1.89	0.92	Indeterminate
18	p.Thr18Phe	1.44	0.52	Indeterminate				1.44	0.52	Indeterminate
19	p.Ala19Asn	0.44	-1.20	Neutral				0.44	-1.20	Neutral
19	p.Ala19Lys	1.07	0.10	Neutral				1.07	0.10	Neutral
19	p.Ala19Thr	0.67	-0.57	Neutral				0.67	-0.57	Neutral
19	p.Ala19Arg	0.97	-0.04	Neutral				0.97	-0.04	Neutral
19	p.Ala19Ser	0.42	-1.24	Neutral				0.42	-1.24	Neutral
19	p.Ala19Ile	0.58	-0.79	Neutral				0.58	-0.79	Neutral
19	p.Ala19Met	0.41	-1.29	Neutral				0.41	-1.29	Neutral
19	p.Ala19His	0.73	-0.46	Neutral				0.73	-0.46	Neutral
19	p.Ala19Gln	0.42	-1.26	Neutral				0.42	-1.26	Neutral
19	p.Ala19Pro	7.17	2.84	Deleterious				7.17	2.84	Deleterious
19	p.Ala19Leu	0.38	-1.40	Neutral				0.38	-1.40	Neutral
19	p.Ala19Asp	1.11	0.15	Neutral				1.11	0.15	Neutral
19	p.Ala19Glu	0.50	-0.99	Neutral				0.50	-0.99	Neutral
19	p.Ala19Ala	1.00	0.00	Neutral				1.00	0.00	Neutral
19	p.Ala19Gly	0.46	-1.12	Neutral				0.46	-1.12	Neutral
19	p.Ala19Val	0.54	-0.88	Neutral				0.54	-0.88	Neutral
19	p.Ala19Tyr	0.57	-0.81	Neutral				0.57	-0.81	Neutral
19	p.Ala19Cys	1.14	0.19	Neutral				1.14	0.19	Neutral
19	p.Ala19Trp	0.40	-1.33	Neutral				0.40	-1.33	Neutral
19	p.Ala19Phe	0.26	-1.96	Neutral				0.26	-1.96	Neutral
20	p.Ala20Asn	17.14	4.10	Deleterious	21.96	4.46	Deleterious	19.55	4.29	Deleterious
20	p.Ala20Lys	21.01	4.39	Deleterious	26.93	4.75	Deleterious	23.97	4.58	Deleterious
20	p.Ala20Thr	1.70	0.77	Indeterminate	1.50	0.58	Indeterminate	1.60	0.68	Indeterminate
20	p.Ala20Arg	19.79	4.31	Deleterious	27.73	4.79	Deleterious	23.76	4.57	Deleterious
20	p.Ala20Ser	0.95	-0.07	Neutral	1.80	0.85	Indeterminate	1.38	0.46	Indeterminate
20	p.Ala20Ile	9.71	3.28	Deleterious	10.13	3.34	Deleterious	9.92	3.31	Deleterious
20	p.Ala20Met	7.13	2.83	Deleterious	8.50	3.09	Deleterious	7.81	2.97	Deleterious
20	p.Ala20His	19.43	4.28	Deleterious	27.11	4.76	Deleterious	23.27	4.54	Deleterious
20	p.Ala20Gln	21.24	4.41	Deleterious	26.35	4.72	Deleterious	23.79	4.57	Deleterious
20	p.Ala20Pro	21.60	4.43	Deleterious	25.71	4.68	Deleterious	23.65	4.56	Deleterious
20	p.Ala20Leu	7.16	2.84	Deleterious	8.21	3.04	Deleterious	7.69	2.94	Deleterious
20	p.Ala20Asp	18.97	4.25	Deleterious	25.25	4.66	Deleterious	22.11	4.47	Deleterious
20	p.Ala20Glu	20.26	4.34	Deleterious	28.11	4.81	Deleterious	24.18	4.60	Deleterious
20	p.Ala20Ala	1.00	0.00	Neutral	1.00	0.00	Neutral	1.00	0.00	Neutral
20	p.Ala20Gly	5.42	2.44	Deleterious	1.92	0.94	Indeterminate	3.67	1.88	Deleterious
20	p.Ala20Val	1.15	0.20	Neutral	1.54	0.62	Indeterminate	1.34	0.42	Indeterminate
20	p.Ala20Tyr	21.80	4.45	Deleterious	26.74	4.74	Deleterious	24.27	4.60	Deleterious
20	p.Ala20Cys	1.18	0.24	Indeterminate	1.34	0.42	Indeterminate	1.26	0.33	Indeterminate
20	p.Ala20Trp	21.50	4.43	Deleterious	27.63	4.79	Deleterious	24.57	4.62	Deleterious

20	p.Ala20Phe	19.92	4.32	Deleterious	26.72	4.74	Deleterious	23.32	4.54	Deleterious
21	p.Ala21Asn	4.93	2.30	Deleterious				4.93	2.30	Deleterious
21	p.Ala21Lys	30.37	4.92	Deleterious				30.37	4.92	Deleterious
21	p.Ala21Thr	1.48	0.57	Indeterminate				1.48	0.57	Indeterminate
21	p.Ala21Arg	12.05	3.59	Deleterious				12.05	3.59	Deleterious
21	p.Ala21Ser	1.05	0.07	Neutral				1.05	0.07	Neutral
21	p.Ala21Ile	2.45	1.29	Deleterious				2.45	1.29	Deleterious
21	p.Ala21Met	3.33	1.73	Deleterious				3.33	1.73	Deleterious
21	p.Ala21His	9.34	3.22	Deleterious				9.34	3.22	Deleterious
21	p.Ala21Gln	6.36	2.67	Deleterious				6.36	2.67	Deleterious
21	p.Ala21Pro	37.99	5.25	Deleterious				37.99	5.25	Deleterious
21	p.Ala21Leu	1.65	0.72	Indeterminate				1.65	0.72	Indeterminate
21	p.Ala21Asp	23.39	4.55	Deleterious				23.39	4.55	Deleterious
21	p.Ala21Glu	9.77	3.29	Deleterious				9.77	3.29	Deleterious
21	p.Ala21Ala	1.00	0.00	Neutral				1.00	0.00	Neutral
21	p.Ala21Gly	0.97	-0.04	Neutral				0.97	-0.04	Neutral
21	p.Ala21Val	1.17	0.23	Neutral				1.17	0.23	Neutral
21	p.Ala21Tyr	7.25	2.86	Deleterious				7.25	2.86	Deleterious
21	p.Ala21Cys	1.05	0.07	Neutral				1.05	0.07	Neutral
21	p.Ala21Trp	7.68	2.94	Deleterious				7.68	2.94	Deleterious
21	p.Ala21Phe	6.02	2.59	Deleterious				6.02	2.59	Deleterious
22	p.Arg22Asn	0.38	-1.41	Neutral				0.38	-1.41	Neutral
22	p.Arg22Lys	0.99	-0.01	Neutral				0.99	-0.01	Neutral
22	p.Arg22Thr	2.13	1.09	Deleterious				2.13	1.09	Deleterious
22	p.Arg22Arg	1.00	0.00	Neutral				1.00	0.00	Neutral
22	p.Arg22Ser	1.50	0.58	Indeterminate				1.50	0.58	Indeterminate
22	p.Arg22Ile	0.86	-0.21	Neutral				0.86	-0.21	Neutral
22	p.Arg22Met	1.40	0.49	Indeterminate				1.40	0.49	Indeterminate
22	p.Arg22His	0.21	-2.22	Neutral				0.21	-2.22	Neutral
22	p.Arg22Gln	0.39	-1.36	Neutral				0.39	-1.36	Neutral
22	p.Arg22Pro	6.72	2.75	Deleterious				6.72	2.75	Deleterious
22	p.Arg22Leu	0.54	-0.89	Neutral				0.54	-0.89	Neutral
22	p.Arg22Asp	0.90	-0.15	Neutral				0.90	-0.15	Neutral
22	p.Arg22Glu	0.31	-1.70	Neutral				0.31	-1.70	Neutral
22	p.Arg22Ala	0.40	-1.31	Neutral				0.40	-1.31	Neutral
22	p.Arg22Gly	3.32	1.73	Deleterious				3.32	1.73	Deleterious
22	p.Arg22Val	0.51	-0.97	Neutral				0.51	-0.97	Neutral
22	p.Arg22Tyr	0.97	-0.04	Neutral				0.97	-0.04	Neutral
22	p.Arg22Cys	0.22	-2.18	Neutral				0.22	-2.18	Neutral
22	p.Arg22Trp	1.58	0.66	Indeterminate				1.58	0.66	Indeterminate
22	p.Arg22Phe	1.80	0.85	Indeterminate				1.80	0.85	Indeterminate
23	p.Gly23Asn	2.46	1.30	Deleterious				2.46	1.30	Deleterious
23	p.Gly23Lys	20.06	4.33	Deleterious				20.06	4.33	Deleterious
23	p.Gly23Thr	20.30	4.34	Deleterious				20.30	4.34	Deleterious
23	p.Gly23Arg	18.67	4.22	Deleterious				18.67	4.22	Deleterious
23	p.Gly23Ser	3.96	1.99	Deleterious				3.96	1.99	Deleterious
23	p.Gly23Ile	23.20	4.54	Deleterious				23.20	4.54	Deleterious
23	p.Gly23Met	19.90	4.31	Deleterious				19.90	4.31	Deleterious
23	p.Gly23His	18.89	4.24	Deleterious				18.89	4.24	Deleterious
23	p.Gly23Gln	18.28	4.19	Deleterious				18.28	4.19	Deleterious
23	p.Gly23Pro	19.40	4.28	Deleterious				19.40	4.28	Deleterious
23	p.Gly23Leu	22.55	4.50	Deleterious				22.55	4.50	Deleterious
23	p.Gly23Asp	14.29	3.84	Deleterious				14.29	3.84	Deleterious
23	p.Gly23Glu	21.21	4.41	Deleterious				21.21	4.41	Deleterious
23	p.Gly23Ala	1.27	0.35	Indeterminate				1.27	0.35	Indeterminate
23	p.Gly23Gly	1.00	0.00	Neutral				1.00	0.00	Neutral
23	p.Gly23Val	19.64	4.30	Deleterious				19.64	4.30	Deleterious
23	p.Gly23Tyr	20.02	4.32	Deleterious				20.02	4.32	Deleterious
23	p.Gly23Cys	13.02	3.70	Deleterious				13.02	3.70	Deleterious
23	p.Gly23Trp	23.15	4.53	Deleterious				23.15	4.53	Deleterious
23	p.Gly23Phe	20.81	4.38	Deleterious				20.81	4.38	Deleterious
24	p.Arg24Asn	0.74	-0.44	Neutral	1.02	0.02	Neutral	0.88	-0.19	Neutral
24	p.Arg24Lys	0.60	-0.75	Neutral	1.18	0.24	Indeterminate	0.89	-0.17	Neutral
24	p.Arg24Thr	0.72	-0.47	Neutral	1.28	0.36	Indeterminate	1.00	0.00	Neutral
24	p.Arg24Arg	1.00	0.00	Neutral	1.00	0.00	Neutral	1.00	0.00	Neutral
24	p.Arg24Ser	0.58	-0.79	Neutral	0.98	-0.03	Neutral	0.78	-0.36	Neutral
24	p.Arg24Ile	0.76	-0.40	Neutral	0.90	-0.16	Neutral	0.83	-0.27	Neutral
24	p.Arg24Met	0.90	-0.15	Neutral	1.08	0.12	Neutral	0.99	-0.01	Neutral
24	p.Arg24His	0.53	-0.93	Neutral	0.83	-0.27	Neutral	0.68	-0.56	Neutral
24	p.Arg24Gln	1.53	0.62	Indeterminate	1.60	0.68	Indeterminate	1.57	0.65	Indeterminate
24	p.Arg24Pro	9.29	3.22	Deleterious	7.17	2.84	Deleterious	8.23	3.04	Deleterious
24	p.Arg24Leu	0.82	-0.29	Neutral	1.06	0.08	Neutral	0.94	-0.10	Neutral
24	p.Arg24Asp	0.74	-0.43	Neutral	0.85	-0.24	Neutral	0.79	-0.33	Neutral
24	p.Arg24Glu	0.67	-0.59	Neutral	1.14	0.18	Indeterminate	0.90	-0.15	Neutral
24	p.Arg24Ala	0.53	-0.91	Neutral	0.85	-0.24	Neutral	0.69	-0.54	Neutral
24	p.Arg24Gly	0.69	-0.54	Neutral	1.23	0.30	Indeterminate	0.96	-0.06	Neutral
24	p.Arg24Val	0.67	-0.57	Neutral	1.02	0.02	Neutral	0.85	-0.24	Neutral
24	p.Arg24Tyr	0.66	-0.61	Neutral	1.14	0.19	Indeterminate	0.90	-0.16	Neutral
24	p.Arg24Cys	0.84	-0.25	Neutral	0.75	-0.42	Neutral	0.79	-0.33	Neutral
24	p.Arg24Trp	0.95	-0.07	Neutral	1.12	0.16	Indeterminate	1.04	0.05	Neutral
24	p.Arg24Phe	0.82	-0.29	Neutral	0.94	-0.09	Neutral	0.88	-0.19	Neutral
25	p.Val25Asn	1.23	0.30	Indeterminate				1.23	0.30	Indeterminate
25	p.Val25Lys	1.86	0.89	Indeterminate				1.86	0.89	Indeterminate
25	p.Val25Thr	1.00	0.00	Neutral				1.00	0.00	Neutral
25	p.Val25Arg	1.37	0.45	Indeterminate				1.37	0.45	Indeterminate
25	p.Val25Ser	0.88	-0.19	Neutral				0.88	-0.19	Neutral
25	p.Val25Ile	0.83	-0.27	Neutral				0.83	-0.27	Neutral
25	p.Val25Met	2.08	1.06	Indeterminate				2.08	1.06	Indeterminate
25	p.Val25His	0.78	-0.36	Neutral				0.78	-0.36	Neutral
25	p.Val25Gln	0.76	-0.39	Neutral				0.76	-0.39	Neutral
25	p.Val25Pro	0.45	-1.14	Neutral				0.45	-1.14	Neutral
25	p.Val25Leu	0.59	-0.76	Neutral				0.59	-0.76	Neutral
25	p.Val25Asp	2.01	1.01	Indeterminate				2.01	1.01	Indeterminate
25	p.Val25Glu	0.89	-0.18	Neutral				0.89	-0.18	Neutral
25	p.Val25Ala	6.36	2.67	Deleterious				6.36	2.67	Deleterious
25	p.Val25Gly	2.04	1.03	Indeterminate				2.04	1.03	Indeterminate
25	p.Val25Val	1.00	0.00	Neutral				1.00	0.00	Neutral
25	p.Val25Tyr	2.88	1.52	Deleterious				2.88	1.52	Deleterious
25	p.Val25Cys	0.61	-0.70	Neutral				0.61	-0.70	Neutral
25	p.Val25Trp	1.21	0.27	Indeterminate				1.21	0.27	Indeterminate
25	p.Val25Phe	1.14	0.19	Neutral				1.14	0.19	Neutral

26	p.Glu26Asn	0.81	-0.30	Neutral				0.81	-0.30	Neutral
26	p.Glu26Lys	0.82	-0.29	Neutral				0.82	-0.29	Neutral
26	p.Glu26Thr	0.92	-0.13	Neutral				0.92	-0.13	Neutral
26	p.Glu26Arg	0.90	-0.16	Neutral				0.90	-0.16	Neutral
26	p.Glu26Ser	1.19	0.25	Indeterminate				1.19	0.25	Indeterminate
26	p.Glu26Ile	0.95	-0.07	Neutral				0.95	-0.07	Neutral
26	p.Glu26Met	0.86	-0.21	Neutral				0.86	-0.21	Neutral
26	p.Glu26His	1.07	0.09	Neutral				1.07	0.09	Neutral
26	p.Glu26Gln	1.05	0.07	Neutral				1.05	0.07	Neutral
26	p.Glu26Pro	1.06	0.08	Neutral				1.06	0.08	Neutral
26	p.Glu26Leu	0.84	-0.25	Neutral				0.84	-0.25	Neutral
26	p.Glu26Asp	1.00	0.00	Neutral				1.00	0.00	Neutral
26	p.Glu26Glu	1.00	0.00	Neutral				1.00	0.00	Neutral
26	p.Glu26Ala	1.07	0.10	Neutral				1.07	0.10	Neutral
26	p.Glu26Gly	1.00	0.01	Neutral				1.00	0.01	Neutral
26	p.Glu26Val	0.98	-0.03	Neutral				0.98	-0.03	Neutral
26	p.Glu26Tyr	1.12	0.16	Neutral				1.12	0.16	Neutral
26	p.Glu26Cys	0.95	-0.08	Neutral				0.95	-0.08	Neutral
26	p.Glu26Trp	0.99	-0.01	Neutral				0.99	-0.01	Neutral
26	p.Glu26Phe	1.04	0.05	Neutral				1.04	0.05	Neutral
27	p.Glu27Asn	0.36	-1.47	Neutral	0.99	-0.01	Neutral	0.68	-0.56	Neutral
27	p.Glu27Lys	0.94	-0.09	Neutral	0.99	-0.02	Neutral	0.96	-0.05	Neutral
27	p.Glu27Thr	1.69	0.76	Indeterminate	1.13	0.17	Indeterminate	1.41	0.50	Indeterminate
27	p.Glu27Arg	0.95	-0.08	Neutral	1.09	0.13	Neutral	1.02	0.03	Neutral
27	p.Glu27Ser	0.76	-0.40	Neutral	0.99	-0.02	Neutral	0.87	-0.20	Neutral
27	p.Glu27Ile	0.66	-0.60	Neutral	1.13	0.17	Indeterminate	0.89	-0.16	Neutral
27	p.Glu27Met	0.93	-0.10	Neutral	1.05	0.06	Neutral	0.99	-0.02	Neutral
27	p.Glu27His	0.89	-0.17	Neutral	1.12	0.16	Indeterminate	1.00	0.01	Neutral
27	p.Glu27Gln	0.91	-0.14	Neutral	1.07	0.10	Neutral	0.99	-0.01	Neutral
27	p.Glu27Pro	1.16	0.22	Neutral	1.26	0.33	Indeterminate	1.21	0.27	Indeterminate
27	p.Glu27Leu	0.56	-0.83	Neutral	1.03	0.04	Neutral	0.80	-0.33	Neutral
27	p.Glu27Asp	0.78	-0.36	Neutral	1.25	0.33	Indeterminate	1.02	0.02	Neutral
27	p.Glu27Glu	1.00	0.00	Neutral	1.00	0.00	Neutral	1.00	0.00	Neutral
27	p.Glu27Ala	0.84	-0.26	Neutral	1.05	0.07	Neutral	0.94	-0.09	Neutral
27	p.Glu27Gly	0.89	-0.17	Neutral	0.97	-0.05	Neutral	0.93	-0.11	Neutral
27	p.Glu27Val	0.82	-0.29	Neutral	1.08	0.11	Neutral	0.95	-0.08	Neutral
27	p.Glu27Tyr	1.84	0.88	Indeterminate	0.95	-0.07	Neutral	1.39	0.48	Indeterminate
27	p.Glu27Cys	0.86	-0.22	Neutral	1.12	0.17	Indeterminate	0.99	-0.01	Neutral
27	p.Glu27Trp	0.78	-0.35	Neutral	0.98	-0.03	Neutral	0.88	-0.18	Neutral
27	p.Glu27Phe	0.61	-0.71	Neutral	0.93	-0.10	Neutral	0.77	-0.37	Neutral
28	p.Val28Asn	5.13	2.36	Deleterious				5.13	2.36	Deleterious
28	p.Val28Lys	7.05	2.82	Deleterious				7.05	2.82	Deleterious
28	p.Val28Thr	0.75	-0.41	Neutral				0.75	-0.41	Neutral
28	p.Val28Arg	6.34	2.66	Deleterious				6.34	2.66	Deleterious
28	p.Val28Ser	1.06	0.08	Neutral				1.06	0.08	Neutral
28	p.Val28Ile	0.79	-0.34	Neutral				0.79	-0.34	Neutral
28	p.Val28Met	0.73	-0.46	Neutral				0.73	-0.46	Neutral
28	p.Val28His	6.67	2.74	Deleterious				6.67	2.74	Deleterious
28	p.Val28Gln	4.46	2.16	Deleterious				4.46	2.16	Deleterious
28	p.Val28Pro	6.33	2.66	Deleterious				6.33	2.66	Deleterious
28	p.Val28Leu	0.60	-0.73	Neutral				0.60	-0.73	Neutral
28	p.Val28Asp	6.27	2.65	Deleterious				6.27	2.65	Deleterious
28	p.Val28Glu	4.08	2.03	Deleterious				4.08	2.03	Deleterious
28	p.Val28Ala	0.72	-0.48	Neutral				0.72	-0.48	Neutral
28	p.Val28Gly	1.76	0.81	Indeterminate				1.76	0.81	Indeterminate
28	p.Val28Val	1.00	0.00	Neutral				1.00	0.00	Neutral
28	p.Val28Tyr	6.56	2.71	Deleterious				6.56	2.71	Deleterious
28	p.Val28Cys	0.70	-0.51	Neutral				0.70	-0.51	Neutral
28	p.Val28Trp	7.13	2.83	Deleterious				7.13	2.83	Deleterious
28	p.Val28Phe	4.68	2.23	Deleterious				4.68	2.23	Deleterious
29	p.Arg29Asn	1.37	0.45	Indeterminate				1.37	0.45	Indeterminate
29	p.Arg29Lys	0.86	-0.22	Neutral				0.86	-0.22	Neutral
29	p.Arg29Thr	1.46	0.55	Indeterminate				1.46	0.55	Indeterminate
29	p.Arg29Arg	1.00	0.00	Neutral				1.00	0.00	Neutral
29	p.Arg29Ser	1.19	0.25	Indeterminate				1.19	0.25	Indeterminate
29	p.Arg29Ile	1.91	0.93	Indeterminate				1.91	0.93	Indeterminate
29	p.Arg29Met	1.05	0.08	Neutral				1.05	0.08	Neutral
29	p.Arg29His	0.93	-0.11	Neutral				0.93	-0.11	Neutral
29	p.Arg29Gln	0.88	-0.18	Neutral				0.88	-0.18	Neutral
29	p.Arg29Pro	15.53	3.96	Deleterious				15.53	3.96	Deleterious
29	p.Arg29Leu	1.01	0.01	Neutral				1.01	0.01	Neutral
29	p.Arg29Asp	1.88	0.91	Indeterminate				1.88	0.91	Indeterminate
29	p.Arg29Glu	1.21	0.28	Indeterminate				1.21	0.28	Indeterminate
29	p.Arg29Ala	0.65	-0.62	Neutral				0.65	-0.62	Neutral
29	p.Arg29Gly	1.12	0.16	Neutral				1.12	0.16	Neutral
29	p.Arg29Val	0.93	-0.11	Neutral				0.93	-0.11	Neutral
29	p.Arg29Tyr	0.87	-0.20	Neutral				0.87	-0.20	Neutral
29	p.Arg29Cys	1.09	0.12	Neutral				1.09	0.12	Neutral
29	p.Arg29Trp	0.80	-0.32	Neutral				0.80	-0.32	Neutral
29	p.Arg29Phe	0.87	-0.20	Neutral				0.87	-0.20	Neutral
30	p.Ala30Asn	3.67	1.87	Deleterious				3.67	1.87	Deleterious
30	p.Ala30Lys	0.90	-0.15	Neutral				0.90	-0.15	Neutral
30	p.Ala30Thr	2.02	1.02	Indeterminate				2.02	1.02	Indeterminate
30	p.Ala30Arg	1.23	0.30	Indeterminate				1.23	0.30	Indeterminate
30	p.Ala30Ser	1.49	0.57	Indeterminate				1.49	0.57	Indeterminate
30	p.Ala30Ile	1.39	0.47	Indeterminate				1.39	0.47	Indeterminate
30	p.Ala30Met	1.37	0.45	Indeterminate				1.37	0.45	Indeterminate
30	p.Ala30His	1.26	0.34	Indeterminate				1.26	0.34	Indeterminate
30	p.Ala30Gln	1.46	0.54	Indeterminate				1.46	0.54	Indeterminate
30	p.Ala30Pro	2.91	1.54	Deleterious				2.91	1.54	Deleterious
30	p.Ala30Leu	1.28	0.36	Indeterminate				1.28	0.36	Indeterminate
30	p.Ala30Asp	2.05	1.04	Indeterminate				2.05	1.04	Indeterminate
30	p.Ala30Glu	1.06	0.08	Neutral				1.06	0.08	Neutral
30	p.Ala30Ala	1.00	0.00	Neutral				1.00	0.00	Neutral
30	p.Ala30Gly	3.33	1.74	Deleterious				3.33	1.74	Deleterious
30	p.Ala30Val	1.56	0.64	Indeterminate				1.56	0.64	Indeterminate
30	p.Ala30Tyr	1.81	0.86	Indeterminate				1.81	0.86	Indeterminate
30	p.Ala30Cys	1.67	0.74	Indeterminate				1.67	0.74	Indeterminate
30	p.Ala30Trp	1.75	0.81	Indeterminate				1.75	0.81	Indeterminate
30	p.Ala30Phe	1.22	0.28	Indeterminate				1.22	0.28	Indeterminate
31	p.Leu31Asn	1.51	0.60	Indeterminate				1.51	0.60	Indeterminate

31	p.Leu31Lys	0.99	-0.01	Neutral				0.99	-0.01	Neutral
31	p.Leu31Thr	1.18	0.23	Neutral				1.18	0.23	Neutral
31	p.Leu31Arg	1.05	0.07	Neutral				1.05	0.07	Neutral
31	p.Leu31Ser	1.41	0.50	Indeterminate				1.41	0.50	Indeterminate
31	p.Leu31Ile	1.00	0.01	Neutral				1.00	0.01	Neutral
31	p.Leu31Met	1.36	0.44	Indeterminate				1.36	0.44	Indeterminate
31	p.Leu31His	1.06	0.08	Neutral				1.06	0.08	Neutral
31	p.Leu31Gln	1.68	0.75	Indeterminate				1.68	0.75	Indeterminate
31	p.Leu31Pro	12.45	3.64	Deleterious				12.45	3.64	Deleterious
31	p.Leu31Leu	1.00	0.00	Neutral				1.00	0.00	Neutral
31	p.Leu31Asp	3.61	1.85	Deleterious				3.61	1.85	Deleterious
31	p.Leu31Glu	1.18	0.23	Neutral				1.18	0.23	Neutral
31	p.Leu31Ala	1.30	0.38	Indeterminate				1.30	0.38	Indeterminate
31	p.Leu31Gly	1.17	0.22	Neutral				1.17	0.22	Neutral
31	p.Leu31Val	1.62	0.70	Indeterminate				1.62	0.70	Indeterminate
31	p.Leu31Tyr	1.97	0.98	Indeterminate				1.97	0.98	Indeterminate
31	p.Leu31Cys	1.81	0.86	Indeterminate				1.81	0.86	Indeterminate
31	p.Leu31Trp	1.45	0.54	Indeterminate				1.45	0.54	Indeterminate
31	p.Leu31Phe	1.04	0.05	Neutral				1.04	0.05	Neutral
32	p.Leu32Asn	2.33	1.22	Deleterious	2.62	1.39	Indeterminate	2.48	1.31	Deleterious
32	p.Leu32Lys	2.83	1.50	Deleterious	2.85	1.51	Indeterminate	2.84	1.51	Deleterious
32	p.Leu32Thr	1.82	0.87	Indeterminate	0.74	-0.43	Neutral	1.28	0.36	Indeterminate
32	p.Leu32Arg	2.57	1.36	Deleterious	2.93	1.55	Indeterminate	2.75	1.46	Deleterious
32	p.Leu32Ser	2.44	1.29	Deleterious	1.52	0.60	Indeterminate	1.98	0.98	Indeterminate
32	p.Leu32Ile	0.90	-0.15	Neutral	0.56	-0.84	Neutral	0.73	-0.45	Neutral
32	p.Leu32Met	1.12	0.17	Neutral	0.55	-0.87	Neutral	0.84	-0.26	Neutral
32	p.Leu32His	2.72	1.45	Deleterious	2.48	1.31	Indeterminate	2.60	1.38	Deleterious
32	p.Leu32Gln	1.65	0.72	Indeterminate	1.57	0.65	Indeterminate	1.61	0.69	Indeterminate
32	p.Leu32Pro	3.00	1.58	Deleterious	3.40	1.76	Deleterious	3.20	1.68	Deleterious
32	p.Leu32Leu	1.00	0.00	Neutral	1.00	0.00	Neutral	1.00	0.00	Neutral
32	p.Leu32Asp	2.58	1.37	Deleterious	3.30	1.72	Indeterminate	2.94	1.56	Deleterious
32	p.Leu32Glu	2.90	1.53	Deleterious	2.82	1.50	Indeterminate	2.86	1.51	Deleterious
32	p.Leu32Ala	1.13	0.18	Neutral	1.16	0.21	Indeterminate	1.15	0.20	Neutral
32	p.Leu32Gly	2.72	1.44	Deleterious	2.47	1.30	Indeterminate	2.59	1.38	Deleterious
32	p.Leu32Val	1.08	0.11	Neutral	0.57	-0.80	Neutral	0.83	-0.28	Neutral
32	p.Leu32Tyr	2.24	1.16	Deleterious	2.50	1.32	Indeterminate	2.37	1.24	Deleterious
32	p.Leu32Cys	1.20	0.26	Indeterminate	0.51	-0.98	Neutral	0.85	-0.23	Neutral
32	p.Leu32Trp	2.64	1.40	Deleterious	2.79	1.48	Indeterminate	2.71	1.44	Deleterious
32	p.Leu32Phe	1.36	0.45	Indeterminate	0.72	-0.47	Neutral	1.04	0.06	Neutral
33	p.Glu33Asn	0.89	-0.16	Neutral				0.89	-0.16	Neutral
33	p.Glu33Lys	0.99	-0.01	Neutral				0.99	-0.01	Neutral
33	p.Glu33Thr	0.95	-0.07	Neutral				0.95	-0.07	Neutral
33	p.Glu33Arg	0.89	-0.17	Neutral				0.89	-0.17	Neutral
33	p.Glu33Ser	1.00	0.00	Neutral				1.00	0.00	Neutral
33	p.Glu33Ile	0.89	-0.16	Neutral				0.89	-0.16	Neutral
33	p.Glu33Met	0.88	-0.18	Neutral				0.88	-0.18	Neutral
33	p.Glu33His	0.96	-0.06	Neutral				0.96	-0.06	Neutral
33	p.Glu33Gln	0.99	-0.02	Neutral				0.99	-0.02	Neutral
33	p.Glu33Pro	0.82	-0.29	Neutral				0.82	-0.29	Neutral
33	p.Glu33Leu	1.03	0.05	Neutral				1.03	0.05	Neutral
33	p.Glu33Asp	0.84	-0.25	Neutral				0.84	-0.25	Neutral
33	p.Glu33Glu	1.00	0.00	Neutral				1.00	0.00	Neutral
33	p.Glu33Ala	1.03	0.04	Neutral				1.03	0.04	Neutral
33	p.Glu33Gly	0.95	-0.08	Neutral				0.95	-0.08	Neutral
33	p.Glu33Val	0.93	-0.11	Neutral				0.93	-0.11	Neutral
33	p.Glu33Tyr	0.93	-0.10	Neutral				0.93	-0.10	Neutral
33	p.Glu33Cys	0.98	-0.03	Neutral				0.98	-0.03	Neutral
33	p.Glu33Trp	0.90	-0.16	Neutral				0.90	-0.16	Neutral
33	p.Glu33Phe	0.95	-0.07	Neutral				0.95	-0.07	Neutral
34	p.Ala34Asn	1.01	0.01	Neutral	1.20	0.26	Indeterminate	1.10	0.14	Neutral
34	p.Ala34Lys	1.01	0.01	Neutral	0.96	-0.06	Neutral	0.98	-0.03	Neutral
34	p.Ala34Thr	0.83	-0.27	Neutral	1.12	0.16	Indeterminate	0.98	-0.04	Neutral
34	p.Ala34Arg	0.93	-0.10	Neutral	0.95	-0.08	Neutral	0.94	-0.09	Neutral
34	p.Ala34Ser	0.68	-0.55	Neutral	0.96	-0.06	Neutral	0.82	-0.28	Neutral
34	p.Ala34Ile	0.83	-0.26	Neutral	0.82	-0.29	Neutral	0.83	-0.28	Neutral
34	p.Ala34Met	1.09	0.12	Neutral	0.88	-0.18	Neutral	0.99	-0.02	Neutral
34	p.Ala34His	0.71	-0.49	Neutral	0.82	-0.28	Neutral	0.77	-0.38	Neutral
34	p.Ala34Gln	1.12	0.16	Neutral	1.16	0.22	Indeterminate	1.14	0.19	Neutral
34	p.Ala34Pro	1.45	0.54	Indeterminate	1.15	0.20	Indeterminate	1.30	0.38	Indeterminate
34	p.Ala34Leu	0.96	-0.06	Neutral	0.99	-0.01	Neutral	0.97	-0.04	Neutral
34	p.Ala34Asp	1.13	0.18	Neutral	0.90	-0.14	Neutral	1.02	0.03	Neutral
34	p.Ala34Glu	1.19	0.25	Indeterminate	0.99	-0.01	Neutral	1.09	0.12	Neutral
34	p.Ala34Ala	1.00	0.00	Neutral	1.00	0.00	Neutral	1.00	0.00	Neutral
34	p.Ala34Gly	1.19	0.25	Indeterminate	0.81	-0.30	Neutral	1.00	0.00	Neutral
34	p.Ala34Val	1.34	0.42	Indeterminate	0.92	-0.12	Neutral	1.13	0.17	Neutral
34	p.Ala34Tyr	0.75	-0.41	Neutral	1.00	0.01	Neutral	0.88	-0.19	Neutral
34	p.Ala34Cys	0.98	-0.04	Neutral	0.78	-0.35	Neutral	0.88	-0.19	Neutral
34	p.Ala34Trp	0.99	-0.02	Neutral	0.88	-0.19	Neutral	0.93	-0.10	Neutral
34	p.Ala34Phe	1.06	0.09	Neutral	0.87	-0.20	Neutral	0.97	-0.05	Neutral
35	p.Gly35Asn	1.69	0.76	Indeterminate	1.30	0.37	Indeterminate	1.49	0.58	Indeterminate
35	p.Gly35Lys	2.04	1.03	Indeterminate	1.61	0.69	Indeterminate	1.83	0.87	Indeterminate
35	p.Gly35Thr	2.63	1.40	Deleterious	2.16	1.11	Indeterminate	2.40	1.26	Deleterious
35	p.Gly35Arg	1.40	0.48	Indeterminate	1.46	0.55	Indeterminate	1.43	0.51	Indeterminate
35	p.Gly35Ser	1.93	0.95	Indeterminate	1.53	0.62	Indeterminate	1.73	0.79	Indeterminate
35	p.Gly35Ile	8.80	3.14	Deleterious	4.34	2.12	Deleterious	6.57	2.72	Deleterious
35	p.Gly35Met	2.40	1.26	Deleterious	2.07	1.05	Indeterminate	2.23	1.16	Deleterious
35	p.Gly35His	1.45	0.53	Indeterminate	1.48	0.56	Indeterminate	1.46	0.55	Indeterminate
35	p.Gly35Gln	1.94	0.95	Indeterminate	1.98	0.99	Indeterminate	1.96	0.97	Indeterminate
35	p.Gly35Pro	14.31	3.84	Deleterious	6.09	2.61	Deleterious	10.20	3.35	Deleterious
35	p.Gly35Leu	3.89	1.96	Deleterious	2.62	1.39	Indeterminate	3.25	1.70	Deleterious
35	p.Gly35Asp	2.28	1.19	Deleterious	1.52	0.61	Indeterminate	1.90	0.93	Indeterminate
35	p.Gly35Glu	1.73	0.79	Indeterminate	1.81	0.85	Indeterminate	1.77	0.82	Indeterminate
35	p.Gly35Ala	1.63	0.71	Indeterminate	1.52	0.61	Indeterminate	1.58	0.66	Indeterminate
35	p.Gly35Gly	1.00	0.00	Neutral	1.00	0.00	Neutral	1.00	0.00	Neutral
35	p.Gly35Val	6.32	2.66	Deleterious	3.44	1.78	Deleterious	4.88	2.29	Deleterious
35	p.Gly35Tyr	2.55	1.35	Deleterious	1.58	0.66	Indeterminate	2.06	1.05	Indeterminate
35	p.Gly35Cys	2.12	1.08	Indeterminate	1.65	0.72	Indeterminate	1.89	0.91	Indeterminate
35	p.Gly35Trp	3.69	1.88	Deleterious	2.44	1.29	Indeterminate	3.06	1.62	Deleterious
35	p.Gly35Phe	1.96	0.97	Indeterminate	1.64	0.71	Indeterminate	1.80	0.84	Indeterminate
36	p.Ala36Asn	2.15	1.11	Deleterious				2.15	1.11	Deleterious
36	p.Ala36Lys	8.35	3.06	Deleterious				8.35	3.06	Deleterious

36	p.Ala36Thr	1.54	0.62	Indeterminate	1.54	0.62	Indeterminate
36	p.Ala36Arg	0.98	-0.03	Neutral	0.98	-0.03	Neutral
36	p.Ala36Ser	1.20	0.26	Indeterminate	1.20	0.26	Indeterminate
36	p.Ala36Ile	0.85	-0.23	Neutral	0.85	-0.23	Neutral
36	p.Ala36Met	0.13	-2.96	Neutral	0.13	-2.96	Neutral
36	p.Ala36His	0.75	-0.41	Neutral	0.75	-0.41	Neutral
36	p.Ala36Gln	0.81	-0.30	Neutral	0.81	-0.30	Neutral
36	p.Ala36Pro	4.00	2.00	Deleterious	4.00	2.00	Deleterious
36	p.Ala36Leu	1.55	0.63	Indeterminate	1.55	0.63	Indeterminate
36	p.Ala36Asp	1.15	0.21	Neutral	1.15	0.21	Neutral
36	p.Ala36Glu	1.67	0.74	Indeterminate	1.67	0.74	Indeterminate
36	p.Ala36Ala	1.00	0.00	Neutral	1.00	0.00	Neutral
36	p.Ala36Gly	1.34	0.42	Indeterminate	1.34	0.42	Indeterminate
36	p.Ala36Val	0.82	-0.28	Neutral	0.82	-0.28	Neutral
36	p.Ala36Tyr	1.32	0.40	Indeterminate	1.32	0.40	Indeterminate
36	p.Ala36Cys	0.23	-2.15	Neutral	0.23	-2.15	Neutral
36	p.Ala36Trp	0.65	-0.61	Neutral	0.65	-0.61	Neutral
36	p.Ala36Phe	1.23	0.30	Indeterminate	1.23	0.30	Indeterminate
37	p.Leu37Asn	0.78	-0.35	Neutral	0.78	-0.35	Neutral
37	p.Leu37Lys	1.29	0.37	Indeterminate	1.29	0.37	Indeterminate
37	p.Leu37Thr	1.40	0.49	Indeterminate	1.40	0.49	Indeterminate
37	p.Leu37Arg	0.43	-1.23	Neutral	0.43	-1.23	Neutral
37	p.Leu37Ser	0.98	-0.03	Neutral	0.98	-0.03	Neutral
37	p.Leu37Ile	1.73	0.79	Indeterminate	1.73	0.79	Indeterminate
37	p.Leu37Met	0.64	-0.65	Neutral	0.64	-0.65	Neutral
37	p.Leu37His	1.39	0.48	Indeterminate	1.39	0.48	Indeterminate
37	p.Leu37Gln	1.66	0.73	Indeterminate	1.66	0.73	Indeterminate
37	p.Leu37Pro	1.90	0.92	Indeterminate	1.90	0.92	Indeterminate
37	p.Leu37Leu	1.00	0.00	Neutral	1.00	0.00	Neutral
37	p.Leu37Asp	1.04	0.05	Neutral	1.04	0.05	Neutral
37	p.Leu37Glu	1.01	0.01	Neutral	1.01	0.01	Neutral
37	p.Leu37Ala	0.74	-0.44	Neutral	0.74	-0.44	Neutral
37	p.Leu37Gly	0.96	-0.06	Neutral	0.96	-0.06	Neutral
37	p.Leu37Val	1.51	0.60	Indeterminate	1.51	0.60	Indeterminate
37	p.Leu37Tyr	1.25	0.32	Indeterminate	1.25	0.32	Indeterminate
37	p.Leu37Cys	0.84	-0.24	Neutral	0.84	-0.24	Neutral
37	p.Leu37Trp	6.84	2.77	Deleterious	6.84	2.77	Deleterious
37	p.Leu37Phe	0.77	-0.37	Neutral	0.77	-0.37	Neutral
38	p.Pro38Asn	3.11	1.64	Deleterious	3.11	1.64	Deleterious
38	p.Pro38Lys	3.68	1.88	Deleterious	3.68	1.88	Deleterious
38	p.Pro38Thr	1.34	0.42	Indeterminate	1.34	0.42	Indeterminate
38	p.Pro38Arg	5.04	2.33	Deleterious	5.04	2.33	Deleterious
38	p.Pro38Ser	1.73	0.79	Indeterminate	1.73	0.79	Indeterminate
38	p.Pro38Ile	1.77	0.82	Indeterminate	1.77	0.82	Indeterminate
38	p.Pro38Met	2.30	1.20	Deleterious	2.30	1.20	Deleterious
38	p.Pro38His	3.80	1.92	Deleterious	3.80	1.92	Deleterious
38	p.Pro38Gln	4.08	2.03	Deleterious	4.08	2.03	Deleterious
38	p.Pro38Pro	1.00	0.00	Neutral	1.00	0.00	Neutral
38	p.Pro38Leu	3.22	1.69	Deleterious	3.22	1.69	Deleterious
38	p.Pro38Asp	20.44	4.35	Deleterious	20.44	4.35	Deleterious
38	p.Pro38Glu	2.65	1.41	Deleterious	2.65	1.41	Deleterious
38	p.Pro38Ala	5.59	2.48	Deleterious	5.59	2.48	Deleterious
38	p.Pro38Gly	0.99	-0.02	Neutral	0.99	-0.02	Neutral
38	p.Pro38Val	1.02	0.03	Neutral	1.02	0.03	Neutral
38	p.Pro38Tyr	3.40	1.77	Deleterious	3.40	1.77	Deleterious
38	p.Pro38Cys	0.75	-0.41	Neutral	0.75	-0.41	Neutral
38	p.Pro38Trp	31.11	4.96	Deleterious	31.11	4.96	Deleterious
38	p.Pro38Phe	4.51	2.17	Deleterious	4.51	2.17	Deleterious
39	p.Asn39Asn	1.00	0.00	Neutral	1.00	0.00	Neutral
39	p.Asn39Lys	2.13	1.09	Deleterious	2.13	1.09	Deleterious
39	p.Asn39Thr	0.63	-0.66	Neutral	0.63	-0.66	Neutral
39	p.Asn39Arg	1.12	0.16	Neutral	1.12	0.16	Neutral
39	p.Asn39Ser	1.17	0.22	Neutral	1.17	0.22	Neutral
39	p.Asn39Ile	1.45	0.53	Indeterminate	1.45	0.53	Indeterminate
39	p.Asn39Met	2.20	1.14	Deleterious	2.20	1.14	Deleterious
39	p.Asn39His	0.71	-0.50	Neutral	0.71	-0.50	Neutral
39	p.Asn39Gln	0.93	-0.10	Neutral	0.93	-0.10	Neutral
39	p.Asn39Pro	14.46	3.85	Deleterious	14.46	3.85	Deleterious
39	p.Asn39Leu	4.17	2.06	Deleterious	4.17	2.06	Deleterious
39	p.Asn39Asp	1.04	0.06	Neutral	1.04	0.06	Neutral
39	p.Asn39Glu	1.18	0.24	Indeterminate	1.18	0.24	Indeterminate
39	p.Asn39Ala	1.30	0.37	Indeterminate	1.30	0.37	Indeterminate
39	p.Asn39Gly	0.98	-0.03	Neutral	0.98	-0.03	Neutral
39	p.Asn39Val	1.33	0.41	Indeterminate	1.33	0.41	Indeterminate
39	p.Asn39Tyr	0.89	-0.17	Neutral	0.89	-0.17	Neutral
39	p.Asn39Cys	4.55	2.19	Deleterious	4.55	2.19	Deleterious
39	p.Asn39Trp	1.64	0.71	Indeterminate	1.64	0.71	Indeterminate
39	p.Asn39Phe	8.47	3.08	Deleterious	8.47	3.08	Deleterious
40	p.Ala40Asn	1.37	0.45	Indeterminate	1.37	0.45	Indeterminate
40	p.Ala40Lys	1.11	0.15	Neutral	1.11	0.15	Neutral
40	p.Ala40Thr	7.75	2.95	Deleterious	7.75	2.95	Deleterious
40	p.Ala40Arg	1.00	0.00	Neutral	1.00	0.00	Neutral
40	p.Ala40Ser	0.73	-0.46	Neutral	0.73	-0.46	Neutral
40	p.Ala40Ile	1.12	0.16	Neutral	1.12	0.16	Neutral
40	p.Ala40Met	0.60	-0.73	Neutral	0.60	-0.73	Neutral
40	p.Ala40His	0.74	-0.44	Neutral	0.74	-0.44	Neutral
40	p.Ala40Gln	0.61	-0.72	Neutral	0.61	-0.72	Neutral
40	p.Ala40Pro	1.50	0.58	Indeterminate	1.50	0.58	Indeterminate
40	p.Ala40Leu	3.93	1.97	Deleterious	3.93	1.97	Deleterious
40	p.Ala40Asp	1.66	0.73	Indeterminate	1.66	0.73	Indeterminate
40	p.Ala40Glu	0.79	-0.35	Neutral	0.79	-0.35	Neutral
40	p.Ala40Ala	1.00	0.00	Neutral	1.00	0.00	Neutral
40	p.Ala40Gly	1.03	0.05	Neutral	1.03	0.05	Neutral
40	p.Ala40Val	1.38	0.47	Indeterminate	1.38	0.47	Indeterminate
40	p.Ala40Tyr	0.73	-0.44	Neutral	0.73	-0.44	Neutral
40	p.Ala40Cys	1.01	0.01	Neutral	1.01	0.01	Neutral
40	p.Ala40Trp	1.17	0.22	Neutral	1.17	0.22	Neutral
40	p.Ala40Phe	1.12	0.16	Neutral	1.12	0.16	Neutral
41	p.Pro41Asn	0.92	-0.12	Neutral	0.92	-0.12	Neutral
41	p.Pro41Lys	1.28	0.35	Indeterminate	1.28	0.35	Indeterminate
41	p.Pro41Thr	1.03	0.04	Neutral	1.03	0.04	Neutral

41	p.Pro41Arg	2.29	1.20	Deleterious				2.29	1.20	Deleterious
41	p.Pro41Ser	0.97	-0.05	Neutral				0.97	-0.05	Neutral
41	p.Pro41Ile	1.61	0.68	Indeterminate				1.61	0.68	Indeterminate
41	p.Pro41Met	0.78	-0.37	Neutral				0.78	-0.37	Neutral
41	p.Pro41His	1.79	0.84	Indeterminate				1.79	0.84	Indeterminate
41	p.Pro41Gln	1.29	0.37	Indeterminate				1.29	0.37	Indeterminate
41	p.Pro41Pro	1.00	0.00	Neutral				1.00	0.00	Neutral
41	p.Pro41Leu	0.79	-0.34	Neutral				0.79	-0.34	Neutral
41	p.Pro41Asp	1.28	0.35	Indeterminate				1.28	0.35	Indeterminate
41	p.Pro41Glu	0.90	-0.15	Neutral				0.90	-0.15	Neutral
41	p.Pro41Ala	1.00	0.01	Neutral				1.00	0.01	Neutral
41	p.Pro41Gly	1.06	0.09	Neutral				1.06	0.09	Neutral
41	p.Pro41Val	1.09	0.13	Neutral				1.09	0.13	Neutral
41	p.Pro41Tyr	2.10	1.07	Indeterminate				2.10	1.07	Indeterminate
41	p.Pro41Cys	1.32	0.40	Indeterminate				1.32	0.40	Indeterminate
41	p.Pro41Trp	1.43	0.52	Indeterminate				1.43	0.52	Indeterminate
41	p.Pro41Phe	1.52	0.61	Indeterminate				1.52	0.61	Indeterminate
42	p.Asn42Asn	1.00	0.00	Neutral				1.00	0.00	Neutral
42	p.Asn42Lys	12.54	3.65	Deleterious				12.54	3.65	Deleterious
42	p.Asn42Thr	1.32	0.41	Indeterminate				1.32	0.41	Indeterminate
42	p.Asn42Arg	11.65	3.54	Deleterious				11.65	3.54	Deleterious
42	p.Asn42Ser	1.22	0.29	Indeterminate				1.22	0.29	Indeterminate
42	p.Asn42Ile	11.57	3.53	Deleterious				11.57	3.53	Deleterious
42	p.Asn42Met	9.77	3.29	Deleterious				9.77	3.29	Deleterious
42	p.Asn42His	7.33	2.87	Deleterious				7.33	2.87	Deleterious
42	p.Asn42Gln	7.10	2.83	Deleterious				7.10	2.83	Deleterious
42	p.Asn42Pro	10.83	3.44	Deleterious				10.83	3.44	Deleterious
42	p.Asn42Leu	11.43	3.51	Deleterious				11.43	3.51	Deleterious
42	p.Asn42Asp	1.61	0.69	Indeterminate				1.61	0.69	Indeterminate
42	p.Asn42Glu	12.48	3.64	Deleterious				12.48	3.64	Deleterious
42	p.Asn42Ala	1.15	0.20	Neutral				1.15	0.20	Neutral
42	p.Asn42Gly	0.94	-0.09	Neutral				0.94	-0.09	Neutral
42	p.Asn42Val	9.99	3.32	Deleterious				9.99	3.32	Deleterious
42	p.Asn42Tyr	11.87	3.57	Deleterious				11.87	3.57	Deleterious
42	p.Asn42Cys	1.48	0.57	Indeterminate				1.48	0.57	Indeterminate
42	p.Asn42Trp	14.65	3.87	Deleterious				14.65	3.87	Deleterious
42	p.Asn42Phe	12.61	3.66	Deleterious				12.61	3.66	Deleterious
43	p.Ser43Asn	1.10	0.14	Neutral				1.10	0.14	Neutral
43	p.Ser43Lys	1.04	0.06	Neutral				1.04	0.06	Neutral
43	p.Ser43Thr	0.97	-0.04	Neutral				0.97	-0.04	Neutral
43	p.Ser43Arg	0.99	-0.01	Neutral				0.99	-0.01	Neutral
43	p.Ser43Ser	1.00	0.00	Neutral				1.00	0.00	Neutral
43	p.Ser43Ile	1.25	0.32	Indeterminate				1.25	0.32	Indeterminate
43	p.Ser43Met	1.09	0.13	Neutral				1.09	0.13	Neutral
43	p.Ser43His	1.00	-0.01	Neutral				1.00	-0.01	Neutral
43	p.Ser43Gln	1.00	0.00	Neutral				1.00	0.00	Neutral
43	p.Ser43Pro	0.99	-0.01	Neutral				0.99	-0.01	Neutral
43	p.Ser43Leu	1.06	0.08	Neutral				1.06	0.08	Neutral
43	p.Ser43Asp	1.09	0.12	Neutral				1.09	0.12	Neutral
43	p.Ser43Glu	1.02	0.03	Neutral				1.02	0.03	Neutral
43	p.Ser43Ala	0.97	-0.05	Neutral				0.97	-0.05	Neutral
43	p.Ser43Gly	0.94	-0.08	Neutral				0.94	-0.08	Neutral
43	p.Ser43Val	1.09	0.13	Neutral				1.09	0.13	Neutral
43	p.Ser43Tyr	1.07	0.10	Neutral				1.07	0.10	Neutral
43	p.Ser43Cys	1.06	0.08	Neutral				1.06	0.08	Neutral
43	p.Ser43Trp	1.11	0.15	Neutral				1.11	0.15	Neutral
43	p.Ser43Phe	1.05	0.08	Neutral				1.05	0.08	Neutral
44	p.Tyr44Asn	1.21	0.28	Indeterminate				1.21	0.28	Indeterminate
44	p.Tyr44Lys	1.21	0.27	Indeterminate				1.21	0.27	Indeterminate
44	p.Tyr44Thr	0.99	-0.02	Neutral				0.99	-0.02	Neutral
44	p.Tyr44Arg	1.03	0.04	Neutral				1.03	0.04	Neutral
44	p.Tyr44Ser	1.09	0.12	Neutral				1.09	0.12	Neutral
44	p.Tyr44Ile	1.05	0.07	Neutral				1.05	0.07	Neutral
44	p.Tyr44Met	1.09	0.12	Neutral				1.09	0.12	Neutral
44	p.Tyr44His	1.10	0.14	Neutral				1.10	0.14	Neutral
44	p.Tyr44Gln	1.28	0.36	Indeterminate				1.28	0.36	Indeterminate
44	p.Tyr44Pro	2.30	1.20	Deleterious				2.30	1.20	Deleterious
44	p.Tyr44Leu	1.09	0.13	Neutral				1.09	0.13	Neutral
44	p.Tyr44Asp	1.24	0.31	Indeterminate				1.24	0.31	Indeterminate
44	p.Tyr44Glu	1.40	0.48	Indeterminate				1.40	0.48	Indeterminate
44	p.Tyr44Ala	1.82	0.87	Indeterminate				1.82	0.87	Indeterminate
44	p.Tyr44Gly	1.38	0.46	Indeterminate				1.38	0.46	Indeterminate
44	p.Tyr44Val	1.09	0.13	Neutral				1.09	0.13	Neutral
44	p.Tyr44Tyr	1.00	0.00	Neutral				1.00	0.00	Neutral
44	p.Tyr44Cys	0.97	-0.05	Neutral				0.97	-0.05	Neutral
44	p.Tyr44Trp	1.10	0.14	Neutral				1.10	0.14	Neutral
44	p.Tyr44Phe	1.05	0.07	Neutral				1.05	0.07	Neutral
45	p.Gly45Asn	0.71	-0.49	Neutral	1.16	0.21	Indeterminate	0.93	-0.10	Neutral
45	p.Gly45Lys	1.34	0.42	Indeterminate	1.14	0.19	Indeterminate	1.24	0.31	Indeterminate
45	p.Gly45Thr	0.64	-0.64	Neutral	1.13	0.17	Indeterminate	0.88	-0.18	Neutral
45	p.Gly45Arg	0.96	-0.05	Neutral	1.11	0.16	Indeterminate	1.04	0.06	Neutral
45	p.Gly45Ser	1.93	0.95	Indeterminate	1.09	0.12	Neutral	1.51	0.59	Indeterminate
45	p.Gly45Ile	1.92	0.94	Indeterminate	1.20	0.27	Indeterminate	1.56	0.65	Indeterminate
45	p.Gly45Met	1.24	0.31	Indeterminate	1.12	0.16	Indeterminate	1.18	0.24	Neutral
45	p.Gly45His	1.10	0.14	Neutral	1.12	0.17	Indeterminate	1.11	0.15	Neutral
45	p.Gly45Gln	0.72	-0.48	Neutral	1.02	0.04	Neutral	0.87	-0.20	Neutral
45	p.Gly45Pro	49.14	5.62	Deleterious	4.40	2.14	Deleterious	26.77	4.74	Deleterious
45	p.Gly45Leu	1.15	0.20	Neutral	1.11	0.15	Indeterminate	1.13	0.17	Neutral
45	p.Gly45Asp	0.71	-0.48	Neutral	1.05	0.07	Neutral	0.88	-0.18	Neutral
45	p.Gly45Glu	1.09	0.12	Neutral	1.08	0.11	Neutral	1.08	0.11	Neutral
45	p.Gly45Ala	0.63	-0.66	Neutral	1.13	0.18	Indeterminate	0.88	-0.18	Neutral
45	p.Gly45Gly	1.00	0.00	Neutral	1.00	0.00	Neutral	1.00	0.00	Neutral
45	p.Gly45Val	1.91	0.93	Indeterminate	1.27	0.34	Indeterminate	1.59	0.67	Indeterminate
45	p.Gly45Tyr	0.73	-0.46	Neutral	1.26	0.33	Indeterminate	0.99	-0.01	Neutral
45	p.Gly45Cys	0.85	-0.23	Neutral	1.09	0.13	Neutral	0.97	-0.04	Neutral
45	p.Gly45Trp	1.04	0.05	Neutral	1.24	0.31	Indeterminate	1.14	0.19	Neutral
45	p.Gly45Phe	0.90	-0.16	Neutral	1.02	0.03	Neutral	0.96	-0.06	Neutral
46	p.Arg46Asn	0.92	-0.13	Neutral				0.92	-0.13	Neutral
46	p.Arg46Lys	0.79	-0.34	Neutral				0.79	-0.34	Neutral
46	p.Arg46Thr	2.61	1.38	Deleterious				2.61	1.38	Deleterious
46	p.Arg46Arg	1.00	0.00	Neutral				1.00	0.00	Neutral

46	p.Arg46Ser	0.85	-0.23	Neutral	0.85	-0.23	Neutral
46	p.Arg46Ile	3.54	1.82	Deleterious	3.54	1.82	Deleterious
46	p.Arg46Met	1.31	0.39	Indeterminate	1.31	0.39	Indeterminate
46	p.Arg46His	0.79	-0.34	Neutral	0.79	-0.34	Neutral
46	p.Arg46Gln	1.54	0.62	Indeterminate	1.54	0.62	Indeterminate
46	p.Arg46Pro	17.80	4.15	Deleterious	17.80	4.15	Deleterious
46	p.Arg46Leu	1.44	0.53	Indeterminate	1.44	0.53	Indeterminate
46	p.Arg46Asp	5.92	2.56	Deleterious	5.92	2.56	Deleterious
46	p.Arg46Glu	3.02	1.59	Deleterious	3.02	1.59	Deleterious
46	p.Arg46Ala	1.39	0.48	Indeterminate	1.39	0.48	Indeterminate
46	p.Arg46Gly	1.36	0.44	Indeterminate	1.36	0.44	Indeterminate
46	p.Arg46Val	1.88	0.91	Indeterminate	1.88	0.91	Indeterminate
46	p.Arg46Tyr	1.75	0.81	Indeterminate	1.75	0.81	Indeterminate
46	p.Arg46Cys	1.36	0.44	Indeterminate	1.36	0.44	Indeterminate
46	p.Arg46Trp	0.81	-0.30	Neutral	0.81	-0.30	Neutral
46	p.Arg46Phe	1.04	0.05	Neutral	1.04	0.05	Neutral
47	p.Arg47Asn	0.91	-0.13	Neutral	0.91	-0.13	Neutral
47	p.Arg47Lys	0.99	-0.01	Neutral	0.99	-0.01	Neutral
47	p.Arg47Thr	0.99	-0.02	Neutral	0.99	-0.02	Neutral
47	p.Arg47Arg	1.00	0.00	Neutral	1.00	0.00	Neutral
47	p.Arg47Ser	1.32	0.41	Indeterminate	1.32	0.41	Indeterminate
47	p.Arg47Ile	1.04	0.05	Neutral	1.04	0.05	Neutral
47	p.Arg47Met	1.06	0.08	Neutral	1.06	0.08	Neutral
47	p.Arg47His	1.06	0.09	Neutral	1.06	0.09	Neutral
47	p.Arg47Gln	1.50	0.59	Indeterminate	1.50	0.59	Indeterminate
47	p.Arg47Pro	1.60	0.67	Indeterminate	1.60	0.67	Indeterminate
47	p.Arg47Leu	0.86	-0.21	Neutral	0.86	-0.21	Neutral
47	p.Arg47Asp	3.53	1.82	Deleterious	3.53	1.82	Deleterious
47	p.Arg47Glu	1.27	0.35	Indeterminate	1.27	0.35	Indeterminate
47	p.Arg47Ala	0.99	-0.01	Neutral	0.99	-0.01	Neutral
47	p.Arg47Gly	1.42	0.51	Indeterminate	1.42	0.51	Indeterminate
47	p.Arg47Val	0.97	-0.04	Neutral	0.97	-0.04	Neutral
47	p.Arg47Tyr	1.14	0.19	Neutral	1.14	0.19	Neutral
47	p.Arg47Cys	0.89	-0.18	Neutral	0.89	-0.18	Neutral
47	p.Arg47Trp	1.20	0.26	Indeterminate	1.20	0.26	Indeterminate
47	p.Arg47Phe	0.89	-0.17	Neutral	0.89	-0.17	Neutral
48	p.Pro48Asn	13.23	3.73	Deleterious	13.23	3.73	Deleterious
48	p.Pro48Lys	15.42	3.95	Deleterious	15.42	3.95	Deleterious
48	p.Pro48Thr	2.13	1.09	Deleterious	2.13	1.09	Deleterious
48	p.Pro48Arg	13.40	3.74	Deleterious	13.40	3.74	Deleterious
48	p.Pro48Ser	1.50	0.58	Indeterminate	1.50	0.58	Indeterminate
48	p.Pro48Ile	7.83	2.97	Deleterious	7.83	2.97	Deleterious
48	p.Pro48Met	11.91	3.57	Deleterious	11.91	3.57	Deleterious
48	p.Pro48His	11.54	3.53	Deleterious	11.54	3.53	Deleterious
48	p.Pro48Gln	14.93	3.90	Deleterious	14.93	3.90	Deleterious
48	p.Pro48Pro	1.00	0.00	Neutral	1.00	0.00	Neutral
48	p.Pro48Leu	17.50	4.13	Deleterious	17.50	4.13	Deleterious
48	p.Pro48Asp	17.91	4.16	Deleterious	17.91	4.16	Deleterious
48	p.Pro48Glu	16.63	4.06	Deleterious	16.63	4.06	Deleterious
48	p.Pro48Ala	0.75	-0.42	Neutral	0.75	-0.42	Neutral
48	p.Pro48Gly	2.08	1.05	Indeterminate	2.08	1.05	Indeterminate
48	p.Pro48Val	2.16	1.11	Deleterious	2.16	1.11	Deleterious
48	p.Pro48Tyr	18.13	4.18	Deleterious	18.13	4.18	Deleterious
48	p.Pro48Cys	2.37	1.25	Deleterious	2.37	1.25	Deleterious
48	p.Pro48Trp	17.96	4.17	Deleterious	17.96	4.17	Deleterious
48	p.Pro48Phe	15.06	3.91	Deleterious	15.06	3.91	Deleterious
49	p.Ile49Asn	6.56	2.71	Deleterious	6.56	2.71	Deleterious
49	p.Ile49Lys	8.49	3.09	Deleterious	8.49	3.09	Deleterious
49	p.Ile49Thr	2.32	1.21	Deleterious	2.32	1.21	Deleterious
49	p.Ile49Arg	7.54	2.91	Deleterious	7.54	2.91	Deleterious
49	p.Ile49Ser	4.51	2.17	Deleterious	4.51	2.17	Deleterious
49	p.Ile49Ile	1.00	0.00	Neutral	1.00	0.00	Neutral
49	p.Ile49Met	1.09	0.12	Neutral	1.09	0.12	Neutral
49	p.Ile49His	4.93	2.30	Deleterious	4.93	2.30	Deleterious
49	p.Ile49Gln	3.98	1.99	Deleterious	3.98	1.99	Deleterious
49	p.Ile49Pro	7.18	2.84	Deleterious	7.18	2.84	Deleterious
49	p.Ile49Leu	0.97	-0.05	Neutral	0.97	-0.05	Neutral
49	p.Ile49Asp	9.18	3.20	Deleterious	9.18	3.20	Deleterious
49	p.Ile49Glu	8.40	3.07	Deleterious	8.40	3.07	Deleterious
49	p.Ile49Ala	2.27	1.19	Deleterious	2.27	1.19	Deleterious
49	p.Ile49Gly	8.88	3.15	Deleterious	8.88	3.15	Deleterious
49	p.Ile49Val	1.09	0.12	Neutral	1.09	0.12	Neutral
49	p.Ile49Tyr	6.77	2.76	Deleterious	6.77	2.76	Deleterious
49	p.Ile49Cys	1.85	0.89	Indeterminate	1.85	0.89	Indeterminate
49	p.Ile49Trp	7.82	2.97	Deleterious	7.82	2.97	Deleterious
49	p.Ile49Phe	2.33	1.22	Deleterious	2.33	1.22	Deleterious
50	p.Gln50Asn	1.33	0.41	Indeterminate	1.33	0.41	Indeterminate
50	p.Gln50Lys	12.74	3.67	Deleterious	12.74	3.67	Deleterious
50	p.Gln50Thr	1.26	0.33	Indeterminate	1.26	0.33	Indeterminate
50	p.Gln50Arg	17.01	4.09	Deleterious	17.01	4.09	Deleterious
50	p.Gln50Ser	1.18	0.24	Indeterminate	1.18	0.24	Indeterminate
50	p.Gln50Ile	4.65	2.22	Deleterious	4.65	2.22	Deleterious
50	p.Gln50Met	1.04	0.05	Neutral	1.04	0.05	Neutral
50	p.Gln50His	4.31	2.11	Deleterious	4.31	2.11	Deleterious
50	p.Gln50Gln	1.00	0.00	Neutral	1.00	0.00	Neutral
50	p.Gln50Pro	18.09	4.18	Deleterious	18.09	4.18	Deleterious
50	p.Gln50Leu	1.87	0.90	Indeterminate	1.87	0.90	Indeterminate
50	p.Gln50Asp	6.89	2.78	Deleterious	6.89	2.78	Deleterious
50	p.Gln50Glu	1.05	0.08	Neutral	1.05	0.08	Neutral
50	p.Gln50Ala	1.36	0.45	Indeterminate	1.36	0.45	Indeterminate
50	p.Gln50Gly	1.47	0.55	Indeterminate	1.47	0.55	Indeterminate
50	p.Gln50Val	3.95	1.98	Deleterious	3.95	1.98	Deleterious
50	p.Gln50Tyr	16.89	4.08	Deleterious	16.89	4.08	Deleterious
50	p.Gln50Cys	1.85	0.89	Indeterminate	1.85	0.89	Indeterminate
50	p.Gln50Trp	14.91	3.90	Deleterious	14.91	3.90	Deleterious
50	p.Gln50Phe	13.94	3.80	Deleterious	13.94	3.80	Deleterious
51	p.Val51Asn	2.32	1.21	Deleterious	2.32	1.21	Deleterious
51	p.Val51Lys	33.67	5.07	Deleterious	33.67	5.07	Deleterious
51	p.Val51Thr	0.95	-0.08	Neutral	0.95	-0.08	Neutral
51	p.Val51Arg	35.63	5.16	Deleterious	35.63	5.16	Deleterious
51	p.Val51Ser	0.75	-0.42	Neutral	0.75	-0.42	Neutral

51	p.Val51Ile	1.28	0.35	Indeterminate	1.28	0.35	Indeterminate
51	p.Val51Met	2.48	1.31	Deleterious	2.48	1.31	Deleterious
51	p.Val51His	19.51	4.29	Deleterious	19.51	4.29	Deleterious
51	p.Val51Gln	3.55	1.83	Deleterious	3.55	1.83	Deleterious
51	p.Val51Pro	22.42	4.49	Deleterious	22.42	4.49	Deleterious
51	p.Val51Leu	1.07	0.10	Neutral	1.07	0.10	Neutral
51	p.Val51Asp	20.68	4.37	Deleterious	20.68	4.37	Deleterious
51	p.Val51Glu	23.51	4.56	Deleterious	23.51	4.56	Deleterious
51	p.Val51Ala	0.57	-0.82	Neutral	0.57	-0.82	Neutral
51	p.Val51Gly	1.00	0.00	Neutral	1.00	0.00	Neutral
51	p.Val51Val	1.00	0.00	Neutral	1.00	0.00	Neutral
51	p.Val51Tyr	11.39	3.51	Deleterious	11.39	3.51	Deleterious
51	p.Val51Cys	0.95	-0.08	Neutral	0.95	-0.08	Neutral
51	p.Val51Trp	24.18	4.60	Deleterious	24.18	4.60	Deleterious
51	p.Val51Phe	8.55	3.10	Deleterious	8.55	3.10	Deleterious
52	p.Met52Asn	1.75	0.81	Indeterminate	1.75	0.81	Indeterminate
52	p.Met52Lys	2.56	1.36	Deleterious	2.56	1.36	Deleterious
52	p.Met52Thr	1.16	0.21	Neutral	1.16	0.21	Neutral
52	p.Met52Arg	2.66	1.41	Deleterious	2.66	1.41	Deleterious
52	p.Met52Ser	1.03	0.04	Neutral	1.03	0.04	Neutral
52	p.Met52Ile	1.42	0.51	Indeterminate	1.42	0.51	Indeterminate
52	p.Met52Met	1.00	0.00	Neutral	1.00	0.00	Neutral
52	p.Met52His	1.54	0.62	Indeterminate	1.54	0.62	Indeterminate
52	p.Met52Gln	1.22	0.29	Indeterminate	1.22	0.29	Indeterminate
52	p.Met52Pro	2.22	1.15	Deleterious	2.22	1.15	Deleterious
52	p.Met52Leu	1.05	0.08	Neutral	1.05	0.08	Neutral
52	p.Met52Asp	2.54	1.34	Deleterious	2.54	1.34	Deleterious
52	p.Met52Glu	2.04	1.03	Indeterminate	2.04	1.03	Indeterminate
52	p.Met52Ala	1.03	0.04	Neutral	1.03	0.04	Neutral
52	p.Met52Gly	0.98	-0.03	Neutral	0.98	-0.03	Neutral
52	p.Met52Val	0.98	-0.03	Neutral	0.98	-0.03	Neutral
52	p.Met52Tyr	1.50	0.59	Indeterminate	1.50	0.59	Indeterminate
52	p.Met52Cys	0.96	-0.06	Neutral	0.96	-0.06	Neutral
52	p.Met52Trp	1.98	0.99	Indeterminate	1.98	0.99	Indeterminate
52	p.Met52Phe	1.29	0.37	Indeterminate	1.29	0.37	Indeterminate
53	p.Met53Asn	2.85	1.51	Deleterious	2.85	1.51	Deleterious
53	p.Met53Lys	1.89	0.92	Indeterminate	1.89	0.92	Indeterminate
53	p.Met53Thr	3.31	1.73	Deleterious	3.31	1.73	Deleterious
53	p.Met53Arg	1.43	0.51	Indeterminate	1.43	0.51	Indeterminate
53	p.Met53Ser	1.68	0.75	Indeterminate	1.68	0.75	Indeterminate
53	p.Met53Ile	6.83	2.77	Deleterious	6.83	2.77	Deleterious
53	p.Met53Met	1.00	0.00	Neutral	1.00	0.00	Neutral
53	p.Met53His	3.28	1.71	Deleterious	3.28	1.71	Deleterious
53	p.Met53Gln	1.45	0.53	Indeterminate	1.45	0.53	Indeterminate
53	p.Met53Pro	9.71	3.28	Deleterious	9.71	3.28	Deleterious
53	p.Met53Leu	1.81	0.86	Indeterminate	1.81	0.86	Indeterminate
53	p.Met53Asp	13.93	3.80	Deleterious	13.93	3.80	Deleterious
53	p.Met53Glu	4.03	2.01	Deleterious	4.03	2.01	Deleterious
53	p.Met53Ala	1.58	0.66	Indeterminate	1.58	0.66	Indeterminate
53	p.Met53Gly	6.11	2.61	Deleterious	6.11	2.61	Deleterious
53	p.Met53Val	7.64	2.93	Deleterious	7.64	2.93	Deleterious
53	p.Met53Tyr	6.71	2.75	Deleterious	6.71	2.75	Deleterious
53	p.Met53Cys	1.82	0.86	Indeterminate	1.82	0.86	Indeterminate
53	p.Met53Trp	11.34	3.50	Deleterious	11.34	3.50	Deleterious
53	p.Met53Phe	5.92	2.57	Deleterious	5.92	2.57	Deleterious
54	p.Met54Asn	1.34	0.43	Indeterminate	1.34	0.43	Indeterminate
54	p.Met54Lys	1.87	0.91	Indeterminate	1.87	0.91	Indeterminate
54	p.Met54Thr	0.96	-0.05	Neutral	0.96	-0.05	Neutral
54	p.Met54Arg	2.35	1.23	Deleterious	2.35	1.23	Deleterious
54	p.Met54Ser	1.12	0.17	Neutral	1.12	0.17	Neutral
54	p.Met54Ile	1.18	0.24	Indeterminate	1.18	0.24	Indeterminate
54	p.Met54Met	1.00	0.00	Neutral	1.00	0.00	Neutral
54	p.Met54His	1.26	0.33	Indeterminate	1.26	0.33	Indeterminate
54	p.Met54Gln	1.66	0.74	Indeterminate	1.66	0.74	Indeterminate
54	p.Met54Pro	2.75	1.46	Deleterious	2.75	1.46	Deleterious
54	p.Met54Leu	1.09	0.13	Neutral	1.09	0.13	Neutral
54	p.Met54Asp	2.35	1.23	Deleterious	2.35	1.23	Deleterious
54	p.Met54Glu	2.09	1.07	Indeterminate	2.09	1.07	Indeterminate
54	p.Met54Ala	1.01	0.02	Neutral	1.01	0.02	Neutral
54	p.Met54Gly	1.03	0.05	Neutral	1.03	0.05	Neutral
54	p.Met54Val	1.05	0.07	Neutral	1.05	0.07	Neutral
54	p.Met54Tyr	1.04	0.06	Neutral	1.04	0.06	Neutral
54	p.Met54Cys	0.90	-0.15	Neutral	0.90	-0.15	Neutral
54	p.Met54Trp	1.22	0.29	Indeterminate	1.22	0.29	Indeterminate
54	p.Met54Phe	1.11	0.15	Neutral	1.11	0.15	Neutral
55	p.Gly55Asn	12.63	3.66	Deleterious	12.63	3.66	Deleterious
55	p.Gly55Lys	12.04	3.59	Deleterious	12.04	3.59	Deleterious
55	p.Gly55Thr	9.96	3.32	Deleterious	9.96	3.32	Deleterious
55	p.Gly55Arg	11.97	3.58	Deleterious	11.97	3.58	Deleterious
55	p.Gly55Ser	2.68	1.42	Deleterious	2.68	1.42	Deleterious
55	p.Gly55Ile	11.78	3.56	Deleterious	11.78	3.56	Deleterious
55	p.Gly55Met	14.07	3.81	Deleterious	14.07	3.81	Deleterious
55	p.Gly55His	15.68	3.97	Deleterious	15.68	3.97	Deleterious
55	p.Gly55Gln	13.16	3.72	Deleterious	13.16	3.72	Deleterious
55	p.Gly55Pro	20.49	4.36	Deleterious	20.49	4.36	Deleterious
55	p.Gly55Leu	17.12	4.10	Deleterious	17.12	4.10	Deleterious
55	p.Gly55Asp	16.49	4.04	Deleterious	16.49	4.04	Deleterious
55	p.Gly55Glu	13.07	3.71	Deleterious	13.07	3.71	Deleterious
55	p.Gly55Ala	1.62	0.70	Indeterminate	1.62	0.70	Indeterminate
55	p.Gly55Gly	1.00	0.00	Neutral	1.00	0.00	Neutral
55	p.Gly55Val	14.09	3.82	Deleterious	14.09	3.82	Deleterious
55	p.Gly55Tyr	10.85	3.44	Deleterious	10.85	3.44	Deleterious
55	p.Gly55Cys	3.75	1.91	Deleterious	3.75	1.91	Deleterious
55	p.Gly55Trp	14.81	3.89	Deleterious	14.81	3.89	Deleterious
55	p.Gly55Phe	11.47	3.52	Deleterious	11.47	3.52	Deleterious
56	p.Ser56Asn	0.30	-1.72	Neutral	0.30	-1.72	Neutral
56	p.Ser56Lys	0.82	-0.29	Neutral	0.82	-0.29	Neutral
56	p.Ser56Thr	0.79	-0.34	Neutral	0.79	-0.34	Neutral
56	p.Ser56Arg	0.38	-1.41	Neutral	0.38	-1.41	Neutral
56	p.Ser56Ser	1.00	0.00	Neutral	1.00	0.00	Neutral
56	p.Ser56Ile	6.45	2.69	Deleterious	6.45	2.69	Deleterious

56	p.Ser56Met	0.42	-1.27	Neutral				0.42	-1.27	Neutral
56	p.Ser56His	0.43	-1.23	Neutral				0.43	-1.23	Neutral
56	p.Ser56Gln	0.57	-0.81	Neutral				0.57	-0.81	Neutral
56	p.Ser56Pro	12.14	3.60	Deleterious				12.14	3.60	Deleterious
56	p.Ser56Leu	1.87	0.90	Indeterminate				1.87	0.90	Indeterminate
56	p.Ser56Asp	0.79	-0.34	Neutral				0.79	-0.34	Neutral
56	p.Ser56Glu	1.20	0.26	Indeterminate				1.20	0.26	Indeterminate
56	p.Ser56Ala	0.77	-0.38	Neutral				0.77	-0.38	Neutral
56	p.Ser56Gly	1.18	0.24	Neutral				1.18	0.24	Neutral
56	p.Ser56Val	1.16	0.22	Neutral				1.16	0.22	Neutral
56	p.Ser56Tyr	0.50	-0.99	Neutral				0.50	-0.99	Neutral
56	p.Ser56Cys	0.43	-1.22	Neutral				0.43	-1.22	Neutral
56	p.Ser56Trp	4.65	2.22	Deleterious				4.65	2.22	Deleterious
56	p.Ser56Phe	0.72	-0.47	Neutral				0.72	-0.47	Neutral
57	p.Ala57Asn	0.47	-1.08	Neutral	1.14	0.19	Indeterminate	0.81	-0.31	Neutral
57	p.Ala57Lys	0.43	-1.22	Neutral	1.30	0.38	Indeterminate	0.87	-0.21	Neutral
57	p.Ala57Thr	0.56	-0.83	Neutral	1.31	0.39	Indeterminate	0.94	-0.09	Neutral
57	p.Ala57Arg	0.49	-1.03	Neutral	1.04	0.05	Neutral	0.76	-0.39	Neutral
57	p.Ala57Ser	0.58	-0.79	Neutral	1.58	0.66	Indeterminate	1.08	0.11	Neutral
57	p.Ala57Ile	0.71	-0.49	Neutral	1.17	0.23	Indeterminate	0.94	-0.09	Neutral
57	p.Ala57Met	0.45	-1.16	Neutral	1.26	0.33	Indeterminate	0.85	-0.23	Neutral
57	p.Ala57His	0.56	-0.84	Neutral	0.90	-0.15	Neutral	0.73	-0.45	Neutral
57	p.Ala57Gln	0.54	-0.89	Neutral	1.27	0.34	Indeterminate	0.90	-0.14	Neutral
57	p.Ala57Pro	0.62	-0.70	Neutral	1.49	0.58	Indeterminate	1.05	0.08	Neutral
57	p.Ala57Leu	0.44	-1.17	Neutral	1.62	0.70	Indeterminate	1.03	0.05	Neutral
57	p.Ala57Asp	0.77	-0.38	Neutral	1.05	0.07	Neutral	0.91	-0.14	Neutral
57	p.Ala57Glu	0.56	-0.83	Neutral	1.84	0.88	Indeterminate	1.20	0.27	Indeterminate
57	p.Ala57Ala	1.00	0.00	Neutral	1.00	0.00	Neutral	1.00	0.00	Neutral
57	p.Ala57Gly	0.50	-1.00	Neutral	1.27	0.35	Indeterminate	0.89	-0.18	Neutral
57	p.Ala57Val	0.53	-0.91	Neutral	1.10	0.14	Neutral	0.82	-0.29	Neutral
57	p.Ala57Tyr	0.52	-0.93	Neutral	1.86	0.89	Indeterminate	1.19	0.25	Indeterminate
57	p.Ala57Cys	0.38	-1.40	Neutral	1.53	0.61	Indeterminate	0.95	-0.07	Neutral
57	p.Ala57Trp	0.46	-1.13	Neutral	1.54	0.63	Indeterminate	1.00	0.00	Neutral
57	p.Ala57Phe	0.33	-1.62	Neutral	1.69	0.76	Indeterminate	1.01	0.01	Neutral
58	p.Arg58Asn	0.75	-0.41	Neutral				0.75	-0.41	Neutral
58	p.Arg58Lys	0.71	-0.49	Neutral				0.71	-0.49	Neutral
58	p.Arg58Thr	0.81	-0.31	Neutral				0.81	-0.31	Neutral
58	p.Arg58Arg	1.00	0.00	Neutral				1.00	0.00	Neutral
58	p.Arg58Ser	0.76	-0.40	Neutral				0.76	-0.40	Neutral
58	p.Arg58Ile	0.81	-0.31	Neutral				0.81	-0.31	Neutral
58	p.Arg58Met	0.71	-0.50	Neutral				0.71	-0.50	Neutral
58	p.Arg58His	0.64	-0.65	Neutral				0.64	-0.65	Neutral
58	p.Arg58Gln	0.93	-0.10	Neutral				0.93	-0.10	Neutral
58	p.Arg58Pro	1.08	0.11	Neutral				1.08	0.11	Neutral
58	p.Arg58Leu	0.83	-0.28	Neutral				0.83	-0.28	Neutral
58	p.Arg58Asp	0.72	-0.48	Neutral				0.72	-0.48	Neutral
58	p.Arg58Glu	0.83	-0.26	Neutral				0.83	-0.26	Neutral
58	p.Arg58Ala	0.88	-0.18	Neutral				0.88	-0.18	Neutral
58	p.Arg58Gly	0.93	-0.11	Neutral				0.93	-0.11	Neutral
58	p.Arg58Val	0.90	-0.16	Neutral				0.90	-0.16	Neutral
58	p.Arg58Tyr	0.62	-0.69	Neutral				0.62	-0.69	Neutral
58	p.Arg58Cys	0.71	-0.50	Neutral				0.71	-0.50	Neutral
58	p.Arg58Trp	0.76	-0.40	Neutral				0.76	-0.40	Neutral
58	p.Arg58Phe	0.90	-0.16	Neutral				0.90	-0.16	Neutral
59	p.Val59Asn	3.19	1.67	Deleterious				3.19	1.67	Deleterious
59	p.Val59Lys	12.88	3.69	Deleterious				12.88	3.69	Deleterious
59	p.Val59Thr	0.86	-0.22	Neutral				0.86	-0.22	Neutral
59	p.Val59Arg	19.15	4.26	Deleterious				19.15	4.26	Deleterious
59	p.Val59Ser	1.25	0.32	Indeterminate				1.25	0.32	Indeterminate
59	p.Val59Ile	0.74	-0.44	Neutral				0.74	-0.44	Neutral
59	p.Val59Met	1.01	0.01	Neutral				1.01	0.01	Neutral
59	p.Val59His	16.58	4.05	Deleterious				16.58	4.05	Deleterious
59	p.Val59Gln	3.62	1.86	Deleterious				3.62	1.86	Deleterious
59	p.Val59Pro	12.69	3.67	Deleterious				12.69	3.67	Deleterious
59	p.Val59Leu	1.31	0.39	Indeterminate				1.31	0.39	Indeterminate
59	p.Val59Asp	20.88	4.38	Deleterious				20.88	4.38	Deleterious
59	p.Val59Glu	11.98	3.58	Deleterious				11.98	3.58	Deleterious
59	p.Val59Ala	1.17	0.23	Neutral				1.17	0.23	Neutral
59	p.Val59Gly	2.18	1.12	Deleterious				2.18	1.12	Deleterious
59	p.Val59Val	1.00	0.00	Neutral				1.00	0.00	Neutral
59	p.Val59Tyr	18.77	4.23	Deleterious				18.77	4.23	Deleterious
59	p.Val59Cys	0.94	-0.08	Neutral				0.94	-0.08	Neutral
59	p.Val59Trp	21.32	4.41	Deleterious				21.32	4.41	Deleterious
59	p.Val59Phe	17.60	4.14	Deleterious				17.60	4.14	Deleterious
60	p.Ala60Asn	2.67	1.42	Deleterious				2.67	1.42	Deleterious
60	p.Ala60Lys	3.23	1.69	Deleterious				3.23	1.69	Deleterious
60	p.Ala60Thr	1.08	0.11	Neutral				1.08	0.11	Neutral
60	p.Ala60Arg	4.98	2.32	Deleterious				4.98	2.32	Deleterious
60	p.Ala60Ser	1.79	0.84	Indeterminate				1.79	0.84	Indeterminate
60	p.Ala60Ile	1.99	0.99	Indeterminate				1.99	0.99	Indeterminate
60	p.Ala60Met	1.23	0.29	Indeterminate				1.23	0.29	Indeterminate
60	p.Ala60His	2.62	1.39	Deleterious				2.62	1.39	Deleterious
60	p.Ala60Gln	3.17	1.67	Deleterious				3.17	1.67	Deleterious
60	p.Ala60Pro	1.71	0.78	Indeterminate				1.71	0.78	Indeterminate
60	p.Ala60Leu	1.06	0.09	Neutral				1.06	0.09	Neutral
60	p.Ala60Asp	2.68	1.42	Deleterious				2.68	1.42	Deleterious
60	p.Ala60Glu	2.83	1.50	Deleterious				2.83	1.50	Deleterious
60	p.Ala60Ala	1.00	0.00	Neutral				1.00	0.00	Neutral
60	p.Ala60Gly	1.05	0.07	Neutral				1.05	0.07	Neutral
60	p.Ala60Val	1.24	0.31	Indeterminate				1.24	0.31	Indeterminate
60	p.Ala60Tyr	2.82	1.49	Deleterious				2.82	1.49	Deleterious
60	p.Ala60Cys	1.32	0.40	Indeterminate				1.32	0.40	Indeterminate
60	p.Ala60Trp	3.21	1.68	Deleterious				3.21	1.68	Deleterious
60	p.Ala60Phe	3.38	1.76	Deleterious				3.38	1.76	Deleterious
61	p.Glu61Asn	0.99	-0.01	Neutral				0.99	-0.01	Neutral
61	p.Glu61Lys	0.78	-0.35	Neutral				0.78	-0.35	Neutral
61	p.Glu61Thr	0.88	-0.18	Neutral				0.88	-0.18	Neutral
61	p.Glu61Arg	1.23	0.30	Indeterminate				1.23	0.30	Indeterminate
61	p.Glu61Ser	1.06	0.09	Neutral				1.06	0.09	Neutral
61	p.Glu61Ile	1.10	0.14	Neutral				1.10	0.14	Neutral
61	p.Glu61Met	1.15	0.20	Neutral				1.15	0.20	Neutral

61	p.Glu61His	1.04	0.05	Neutral				1.04	0.05	Neutral
61	p.Glu61Gln	0.84	-0.26	Neutral				0.84	-0.26	Neutral
61	p.Glu61Pro	6.09	2.61	Deleterious				6.09	2.61	Deleterious
61	p.Glu61Leu	0.92	-0.12	Neutral				0.92	-0.12	Neutral
61	p.Glu61Asp	0.94	-0.09	Neutral				0.94	-0.09	Neutral
61	p.Glu61Glu	1.00	0.00	Neutral				1.00	0.00	Neutral
61	p.Glu61Ala	0.91	-0.13	Neutral				0.91	-0.13	Neutral
61	p.Glu61Gly	0.83	-0.27	Neutral				0.83	-0.27	Neutral
61	p.Glu61Val	0.87	-0.19	Neutral				0.87	-0.19	Neutral
61	p.Glu61Tyr	1.21	0.28	Indeterminate				1.21	0.28	Indeterminate
61	p.Glu61Cys	0.91	-0.14	Neutral				0.91	-0.14	Neutral
61	p.Glu61Trp	1.04	0.05	Neutral				1.04	0.05	Neutral
61	p.Glu61Phe	0.97	-0.04	Neutral				0.97	-0.04	Neutral
62	p.Leu62Asn	0.67	-0.58	Neutral	0.80	-0.32	Neutral	0.74	-0.44	Neutral
62	p.Leu62Lys	1.33	0.41	Indeterminate	0.82	-0.29	Neutral	1.07	0.10	Neutral
62	p.Leu62Thr	1.04	0.06	Neutral	0.79	-0.34	Neutral	0.92	-0.13	Neutral
62	p.Leu62Arg	2.27	1.18	Deleterious	0.53	-0.92	Neutral	1.40	0.48	Indeterminate
62	p.Leu62Ser	0.86	-0.22	Neutral	0.76	-0.40	Neutral	0.81	-0.31	Neutral
62	p.Leu62Ile	1.09	0.12	Neutral	0.45	-1.14	Neutral	0.77	-0.38	Neutral
62	p.Leu62Met	0.84	-0.26	Neutral	0.53	-0.93	Neutral	0.68	-0.56	Neutral
62	p.Leu62His	0.70	-0.51	Neutral	0.89	-0.16	Neutral	0.80	-0.32	Neutral
62	p.Leu62Gln	3.81	1.93	Deleterious	1.38	0.47	Indeterminate	2.60	1.38	Deleterious
62	p.Leu62Pro	0.43	-1.20	Neutral	0.25	-2.03	Neutral	0.34	-1.56	Neutral
62	p.Leu62Leu	1.00	0.00	Neutral	1.00	0.00	Neutral	1.00	0.00	Neutral
62	p.Leu62Asp	1.05	0.07	Neutral	1.09	0.13	Neutral	1.07	0.10	Neutral
62	p.Leu62Glu	1.16	0.21	Neutral	0.44	-1.19	Neutral	0.80	-0.32	Neutral
62	p.Leu62Ala	0.94	-0.09	Neutral	0.47	-1.08	Neutral	0.71	-0.50	Neutral
62	p.Leu62Gly	1.44	0.52	Indeterminate	1.17	0.23	Indeterminate	1.30	0.38	Indeterminate
62	p.Leu62Val	1.10	0.14	Neutral	0.58	-0.80	Neutral	0.84	-0.25	Neutral
62	p.Leu62Tyr	1.76	0.82	Indeterminate	0.96	-0.06	Neutral	1.36	0.44	Indeterminate
62	p.Leu62Cys	0.91	-0.14	Neutral	0.83	-0.27	Neutral	0.87	-0.20	Neutral
62	p.Leu62Trp	0.84	-0.25	Neutral	0.89	-0.16	Neutral	0.87	-0.20	Neutral
62	p.Leu62Phe	0.64	-0.64	Neutral	0.71	-0.49	Neutral	0.68	-0.56	Neutral
63	p.Leu63Asn	13.29	3.73	Deleterious				13.29	3.73	Deleterious
63	p.Leu63Lys	23.24	4.54	Deleterious				23.24	4.54	Deleterious
63	p.Leu63Thr	12.62	3.66	Deleterious				12.62	3.66	Deleterious
63	p.Leu63Arg	17.32	4.11	Deleterious				17.32	4.11	Deleterious
63	p.Leu63Ser	15.92	3.99	Deleterious				15.92	3.99	Deleterious
63	p.Leu63Ile	1.41	0.49	Indeterminate				1.41	0.49	Indeterminate
63	p.Leu63Met	0.85	-0.23	Neutral				0.85	-0.23	Neutral
63	p.Leu63His	16.94	4.08	Deleterious				16.94	4.08	Deleterious
63	p.Leu63Gln	19.33	4.27	Deleterious				19.33	4.27	Deleterious
63	p.Leu63Pro	20.64	4.37	Deleterious				20.64	4.37	Deleterious
63	p.Leu63Leu	1.00	0.00	Neutral				1.00	0.00	Neutral
63	p.Leu63Asp	31.92	5.00	Deleterious				31.92	5.00	Deleterious
63	p.Leu63Glu	14.81	3.89	Deleterious				14.81	3.89	Deleterious
63	p.Leu63Ala	15.76	3.98	Deleterious				15.76	3.98	Deleterious
63	p.Leu63Gly	19.84	4.31	Deleterious				19.84	4.31	Deleterious
63	p.Leu63Val	3.31	1.73	Deleterious				3.31	1.73	Deleterious
63	p.Leu63Tyr	16.24	4.02	Deleterious				16.24	4.02	Deleterious
63	p.Leu63Cys	3.30	1.72	Deleterious				3.30	1.72	Deleterious
63	p.Leu63Trp	16.99	4.09	Deleterious				16.99	4.09	Deleterious
63	p.Leu63Phe	2.23	1.16	Deleterious				2.23	1.16	Deleterious
64	p.Leu64Asn	1.51	0.59	Indeterminate				1.51	0.59	Indeterminate
64	p.Leu64Lys	1.15	0.20	Neutral				1.15	0.20	Neutral
64	p.Leu64Thr	0.91	-0.14	Neutral				0.91	-0.14	Neutral
64	p.Leu64Arg	1.25	0.32	Indeterminate				1.25	0.32	Indeterminate
64	p.Leu64Ser	0.89	-0.16	Neutral				0.89	-0.16	Neutral
64	p.Leu64Ile	1.17	0.22	Neutral				1.17	0.22	Neutral
64	p.Leu64Met	0.97	-0.05	Neutral				0.97	-0.05	Neutral
64	p.Leu64His	1.25	0.32	Indeterminate				1.25	0.32	Indeterminate
64	p.Leu64Gln	1.34	0.43	Indeterminate				1.34	0.43	Indeterminate
64	p.Leu64Pro	3.96	1.99	Deleterious				3.96	1.99	Deleterious
64	p.Leu64Leu	1.00	0.00	Neutral				1.00	0.00	Neutral
64	p.Leu64Asp	1.80	0.85	Indeterminate				1.80	0.85	Indeterminate
64	p.Leu64Glu	1.41	0.50	Indeterminate				1.41	0.50	Indeterminate
64	p.Leu64Ala	1.14	0.19	Neutral				1.14	0.19	Neutral
64	p.Leu64Gly	1.37	0.46	Indeterminate				1.37	0.46	Indeterminate
64	p.Leu64Val	0.81	-0.30	Neutral				0.81	-0.30	Neutral
64	p.Leu64Tyr	1.01	0.02	Neutral				1.01	0.02	Neutral
64	p.Leu64Cys	0.97	-0.05	Neutral				0.97	-0.05	Neutral
64	p.Leu64Trp	1.09	0.12	Neutral				1.09	0.12	Neutral
64	p.Leu64Phe	0.83	-0.27	Neutral				0.83	-0.27	Neutral
65	p.Leu65Asn	1.41	0.50	Indeterminate				1.41	0.50	Indeterminate
65	p.Leu65Lys	0.82	-0.28	Neutral				0.82	-0.28	Neutral
65	p.Leu65Thr	1.18	0.23	Neutral				1.18	0.23	Neutral
65	p.Leu65Arg	0.95	-0.07	Neutral				0.95	-0.07	Neutral
65	p.Leu65Ser	1.22	0.29	Indeterminate				1.22	0.29	Indeterminate
65	p.Leu65Ile	0.97	-0.04	Neutral				0.97	-0.04	Neutral
65	p.Leu65Met	0.81	-0.30	Neutral				0.81	-0.30	Neutral
65	p.Leu65His	1.30	0.38	Indeterminate				1.30	0.38	Indeterminate
65	p.Leu65Gln	1.26	0.33	Indeterminate				1.26	0.33	Indeterminate
65	p.Leu65Pro	11.28	3.50	Deleterious				11.28	3.50	Deleterious
65	p.Leu65Leu	1.00	0.00	Neutral				1.00	0.00	Neutral
65	p.Leu65Asp	0.96	-0.06	Neutral				0.96	-0.06	Neutral
65	p.Leu65Glu	0.90	-0.14	Neutral				0.90	-0.14	Neutral
65	p.Leu65Ala	1.18	0.23	Neutral				1.18	0.23	Neutral
65	p.Leu65Gly	1.49	0.57	Indeterminate				1.49	0.57	Indeterminate
65	p.Leu65Val	1.11	0.15	Neutral				1.11	0.15	Neutral
65	p.Leu65Tyr	0.99	-0.01	Neutral				0.99	-0.01	Neutral
65	p.Leu65Cys	1.57	0.65	Indeterminate				1.57	0.65	Indeterminate
65	p.Leu65Trp	1.40	0.49	Indeterminate				1.40	0.49	Indeterminate
65	p.Leu65Phe	0.97	-0.05	Neutral				0.97	-0.05	Neutral
66	p.His66Asn	0.87	-0.21	Neutral				0.87	-0.21	Neutral
66	p.His66Lys	0.89	-0.16	Neutral				0.89	-0.16	Neutral
66	p.His66Thr	1.15	0.20	Neutral				1.15	0.20	Neutral
66	p.His66Arg	1.21	0.28	Indeterminate				1.21	0.28	Indeterminate
66	p.His66Ser	1.03	0.05	Neutral				1.03	0.05	Neutral
66	p.His66Ile	1.57	0.65	Indeterminate				1.57	0.65	Indeterminate
66	p.His66Met	0.91	-0.14	Neutral				0.91	-0.14	Neutral
66	p.His66His	1.00	0.00	Neutral				1.00	0.00	Neutral

66	p.His66Gln	1.14	0.19	Neutral	1.14	0.19	Neutral
66	p.His66Pro	18.02	4.17	Deleterious	18.02	4.17	Deleterious
66	p.His66Leu	1.18	0.24	Indeterminate	1.18	0.24	Indeterminate
66	p.His66Asp	0.96	-0.06	Neutral	0.96	-0.06	Neutral
66	p.His66Glu	1.34	0.42	Indeterminate	1.34	0.42	Indeterminate
66	p.His66Ala	1.24	0.31	Indeterminate	1.24	0.31	Indeterminate
66	p.His66Gly	1.08	0.12	Neutral	1.08	0.12	Neutral
66	p.His66Val	1.40	0.49	Indeterminate	1.40	0.49	Indeterminate
66	p.His66Tyr	1.05	0.08	Neutral	1.05	0.08	Neutral
66	p.His66Cys	0.69	-0.53	Neutral	0.69	-0.53	Neutral
66	p.His66Trp	0.97	-0.04	Neutral	0.97	-0.04	Neutral
66	p.His66Phe	1.00	0.00	Neutral	1.00	0.00	Neutral
67	p.Gly67Asn	0.80	-0.33	Neutral	0.80	-0.33	Neutral
67	p.Gly67Lys	1.04	0.05	Neutral	1.04	0.05	Neutral
67	p.Gly67Thr	1.66	0.73	Indeterminate	1.66	0.73	Indeterminate
67	p.Gly67Arg	0.67	-0.57	Neutral	0.67	-0.57	Neutral
67	p.Gly67Ser	0.75	-0.42	Neutral	0.75	-0.42	Neutral
67	p.Gly67Ile	7.82	2.97	Deleterious	7.82	2.97	Deleterious
67	p.Gly67Met	1.18	0.24	Neutral	1.18	0.24	Neutral
67	p.Gly67His	0.76	-0.40	Neutral	0.76	-0.40	Neutral
67	p.Gly67Gln	0.61	-0.71	Neutral	0.61	-0.71	Neutral
67	p.Gly67Pro	15.51	3.95	Deleterious	15.51	3.95	Deleterious
67	p.Gly67Leu	0.96	-0.05	Neutral	0.96	-0.05	Neutral
67	p.Gly67Asp	0.82	-0.28	Neutral	0.82	-0.28	Neutral
67	p.Gly67Glu	0.81	-0.30	Neutral	0.81	-0.30	Neutral
67	p.Gly67Ala	0.64	-0.63	Neutral	0.64	-0.63	Neutral
67	p.Gly67Gly	1.00	0.00	Neutral	1.00	0.00	Neutral
67	p.Gly67Val	4.79	2.26	Deleterious	4.79	2.26	Deleterious
67	p.Gly67Tyr	0.91	-0.14	Neutral	0.91	-0.14	Neutral
67	p.Gly67Cys	1.14	0.19	Neutral	1.14	0.19	Neutral
67	p.Gly67Trp	1.46	0.54	Indeterminate	1.46	0.54	Indeterminate
67	p.Gly67Phe	1.17	0.23	Neutral	1.17	0.23	Neutral
68	p.Ala68Asn	3.18	1.67	Deleterious	3.18	1.67	Deleterious
68	p.Ala68Lys	4.02	2.01	Deleterious	4.02	2.01	Deleterious
68	p.Ala68Thr	1.35	0.43	Indeterminate	1.35	0.43	Indeterminate
68	p.Ala68Arg	4.05	2.02	Deleterious	4.05	2.02	Deleterious
68	p.Ala68Ser	0.96	-0.05	Neutral	0.96	-0.05	Neutral
68	p.Ala68Ile	3.27	1.71	Deleterious	3.27	1.71	Deleterious
68	p.Ala68Met	2.86	1.51	Deleterious	2.86	1.51	Deleterious
68	p.Ala68His	2.14	1.10	Deleterious	2.14	1.10	Deleterious
68	p.Ala68Gln	3.25	1.70	Deleterious	3.25	1.70	Deleterious
68	p.Ala68Pro	3.53	1.82	Deleterious	3.53	1.82	Deleterious
68	p.Ala68Leu	3.66	1.87	Deleterious	3.66	1.87	Deleterious
68	p.Ala68Asp	2.46	1.30	Deleterious	2.46	1.30	Deleterious
68	p.Ala68Glu	2.62	1.39	Deleterious	2.62	1.39	Deleterious
68	p.Ala68Ala	1.00	0.00	Neutral	1.00	0.00	Neutral
68	p.Ala68Gly	1.07	0.10	Neutral	1.07	0.10	Neutral
68	p.Ala68Val	1.78	0.83	Indeterminate	1.78	0.83	Indeterminate
68	p.Ala68Tyr	3.77	1.91	Deleterious	3.77	1.91	Deleterious
68	p.Ala68Cys	1.13	0.17	Neutral	1.13	0.17	Neutral
68	p.Ala68Trp	4.01	2.01	Deleterious	4.01	2.01	Deleterious
68	p.Ala68Phe	3.65	1.87	Deleterious	3.65	1.87	Deleterious
69	p.Glu69Asn	2.72	1.44	Deleterious	2.72	1.44	Deleterious
69	p.Glu69Lys	2.34	1.23	Deleterious	2.34	1.23	Deleterious
69	p.Glu69Thr	1.54	0.62	Indeterminate	1.54	0.62	Indeterminate
69	p.Glu69Arg	1.08	0.10	Neutral	1.08	0.10	Neutral
69	p.Glu69Ser	1.80	0.85	Indeterminate	1.80	0.85	Indeterminate
69	p.Glu69Ile	1.34	0.43	Indeterminate	1.34	0.43	Indeterminate
69	p.Glu69Met	2.32	1.21	Deleterious	2.32	1.21	Deleterious
69	p.Glu69His	1.43	0.52	Indeterminate	1.43	0.52	Indeterminate
69	p.Glu69Gln	1.35	0.43	Indeterminate	1.35	0.43	Indeterminate
69	p.Glu69Pro	4.86	2.28	Deleterious	4.86	2.28	Deleterious
69	p.Glu69Leu	2.65	1.40	Deleterious	2.65	1.40	Deleterious
69	p.Glu69Asp	1.27	0.35	Indeterminate	1.27	0.35	Indeterminate
69	p.Glu69Glu	1.00	0.00	Neutral	1.00	0.00	Neutral
69	p.Glu69Ala	0.81	-0.30	Neutral	0.81	-0.30	Neutral
69	p.Glu69Gly	1.67	0.74	Indeterminate	1.67	0.74	Indeterminate
69	p.Glu69Val	2.17	1.11	Deleterious	2.17	1.11	Deleterious
69	p.Glu69Tyr	1.41	0.49	Indeterminate	1.41	0.49	Indeterminate
69	p.Glu69Cys	1.41	0.49	Indeterminate	1.41	0.49	Indeterminate
69	p.Glu69Trp	1.92	0.94	Indeterminate	1.92	0.94	Indeterminate
69	p.Glu69Phe	1.60	0.68	Indeterminate	1.60	0.68	Indeterminate
70	p.Pro70Asn	1.38	0.46	Indeterminate	1.38	0.46	Indeterminate
70	p.Pro70Lys	1.94	0.96	Indeterminate	1.94	0.96	Indeterminate
70	p.Pro70Thr	1.18	0.24	Neutral	1.18	0.24	Neutral
70	p.Pro70Arg	1.09	0.13	Neutral	1.09	0.13	Neutral
70	p.Pro70Ser	2.28	1.19	Deleterious	2.28	1.19	Deleterious
70	p.Pro70Ile	0.96	-0.07	Neutral	0.96	-0.07	Neutral
70	p.Pro70Met	0.51	-0.97	Neutral	0.51	-0.97	Neutral
70	p.Pro70His	0.47	-1.09	Neutral	0.47	-1.09	Neutral
70	p.Pro70Gln	0.43	-1.23	Neutral	0.43	-1.23	Neutral
70	p.Pro70Pro	1.00	0.00	Neutral	1.00	0.00	Neutral
70	p.Pro70Leu	1.23	0.30	Indeterminate	1.23	0.30	Indeterminate
70	p.Pro70Asp	10.85	3.44	Deleterious	10.85	3.44	Deleterious
70	p.Pro70Glu	5.22	2.38	Deleterious	5.22	2.38	Deleterious
70	p.Pro70Ala	1.00	0.00	Neutral	1.00	0.00	Neutral
70	p.Pro70Gly	1.03	0.05	Neutral	1.03	0.05	Neutral
70	p.Pro70Val	0.69	-0.54	Neutral	0.69	-0.54	Neutral
70	p.Pro70Tyr	0.96	-0.07	Neutral	0.96	-0.07	Neutral
70	p.Pro70Cys	0.40	-1.32	Neutral	0.40	-1.32	Neutral
70	p.Pro70Trp	3.19	1.67	Deleterious	3.19	1.67	Deleterious
70	p.Pro70Phe	0.85	-0.23	Neutral	0.85	-0.23	Neutral
71	p.Asn71Asn	1.00	0.00	Neutral	1.00	0.00	Neutral
71	p.Asn71Lys	17.91	4.16	Deleterious	17.91	4.16	Deleterious
71	p.Asn71Thr	3.40	1.77	Deleterious	3.40	1.77	Deleterious
71	p.Asn71Arg	13.92	3.80	Deleterious	13.92	3.80	Deleterious
71	p.Asn71Ser	30.08	4.91	Deleterious	30.08	4.91	Deleterious
71	p.Asn71Ile	39.18	5.29	Deleterious	39.18	5.29	Deleterious
71	p.Asn71Met	5.00	2.32	Deleterious	5.00	2.32	Deleterious
71	p.Asn71His	1.98	0.99	Indeterminate	1.98	0.99	Indeterminate
71	p.Asn71Gln	1.66	0.73	Indeterminate	1.66	0.73	Indeterminate

71	p.Asn71Pro	84.59	6.40	Deleterious				84.59	6.40	Deleterious
71	p.Asn71Leu	32.49	5.02	Deleterious				32.49	5.02	Deleterious
71	p.Asn71Asp	2.31	1.21	Deleterious				2.31	1.21	Deleterious
71	p.Asn71Glu	1.89	0.92	Indeterminate				1.89	0.92	Indeterminate
71	p.Asn71Ala	2.26	1.18	Deleterious				2.26	1.18	Deleterious
71	p.Asn71Gly	1.06	0.08	Neutral				1.06	0.08	Neutral
71	p.Asn71Val	28.77	4.85	Deleterious				28.77	4.85	Deleterious
71	p.Asn71Tyr	17.62	4.14	Deleterious				17.62	4.14	Deleterious
71	p.Asn71Cys	1.77	0.82	Indeterminate				1.77	0.82	Indeterminate
71	p.Asn71Trp	6.47	2.69	Deleterious				6.47	2.69	Deleterious
71	p.Asn71Phe	17.55	4.13	Deleterious				17.55	4.13	Deleterious
72	p.Cys72Asn	1.42	0.51	Indeterminate	0.93	-0.10	Neutral	1.18	0.24	Neutral
72	p.Cys72Lys	1.32	0.40	Indeterminate	1.04	0.06	Neutral	1.18	0.24	Neutral
72	p.Cys72Thr	1.37	0.45	Indeterminate	0.85	-0.23	Neutral	1.11	0.15	Neutral
72	p.Cys72Arg	0.90	-0.16	Neutral	0.70	-0.51	Neutral	0.80	-0.32	Neutral
72	p.Cys72Ser	1.29	0.37	Indeterminate	1.21	0.28	Indeterminate	1.25	0.32	Indeterminate
72	p.Cys72Ile	1.37	0.46	Indeterminate	1.12	0.17	Indeterminate	1.25	0.32	Indeterminate
72	p.Cys72Met	1.00	0.01	Neutral	1.03	0.04	Neutral	1.01	0.02	Neutral
72	p.Cys72His	1.08	0.11	Neutral	1.11	0.15	Indeterminate	1.10	0.13	Neutral
72	p.Cys72Gln	1.57	0.65	Indeterminate	0.82	-0.29	Neutral	1.19	0.26	Indeterminate
72	p.Cys72Pro	1.59	0.67	Indeterminate	0.82	-0.29	Neutral	1.20	0.26	Indeterminate
72	p.Cys72Leu	1.44	0.53	Indeterminate	1.14	0.19	Indeterminate	1.29	0.37	Indeterminate
72	p.Cys72Asp	1.02	0.03	Neutral	1.28	0.35	Indeterminate	1.15	0.20	Neutral
72	p.Cys72Glu	1.20	0.27	Indeterminate	1.23	0.30	Indeterminate	1.22	0.28	Indeterminate
72	p.Cys72Ala	1.09	0.12	Neutral	1.24	0.31	Indeterminate	1.16	0.22	Neutral
72	p.Cys72Gly	1.05	0.08	Neutral	0.60	-0.74	Neutral	0.83	-0.27	Neutral
72	p.Cys72Val	1.33	0.42	Indeterminate	1.29	0.36	Indeterminate	1.31	0.39	Indeterminate
72	p.Cys72Tyr	1.12	0.17	Neutral	0.96	-0.06	Neutral	1.04	0.06	Neutral
72	p.Cys72Cys	1.00	0.00	Neutral	1.00	0.00	Neutral	1.00	0.00	Neutral
72	p.Cys72Trp	1.78	0.83	Indeterminate	1.06	0.09	Neutral	1.42	0.51	Indeterminate
72	p.Cys72Phe	0.99	-0.01	Neutral	1.57	0.65	Indeterminate	1.28	0.35	Indeterminate
73	p.Ala73Asn	1.22	0.29	Indeterminate	1.06	0.09	Neutral	1.14	0.19	Neutral
73	p.Ala73Lys	0.77	-0.37	Neutral	1.02	0.02	Neutral	0.90	-0.16	Neutral
73	p.Ala73Thr	1.07	0.10	Neutral	0.92	-0.11	Neutral	1.00	0.00	Neutral
73	p.Ala73Arg	1.17	0.23	Neutral	0.93	-0.10	Neutral	1.05	0.07	Neutral
73	p.Ala73Ser	1.11	0.15	Neutral	1.00	-0.01	Neutral	1.05	0.07	Neutral
73	p.Ala73Ile	0.81	-0.30	Neutral	0.97	-0.04	Neutral	0.89	-0.17	Neutral
73	p.Ala73Met	0.82	-0.29	Neutral	1.00	0.00	Neutral	0.91	-0.14	Neutral
73	p.Ala73His	0.90	-0.15	Neutral	0.95	-0.07	Neutral	0.93	-0.11	Neutral
73	p.Ala73Gln	1.03	0.05	Neutral	0.95	-0.07	Neutral	0.99	-0.01	Neutral
73	p.Ala73Pro	1.00	0.00	Neutral	1.08	0.11	Neutral	1.04	0.06	Neutral
73	p.Ala73Leu	0.85	-0.23	Neutral	0.99	-0.01	Neutral	0.92	-0.12	Neutral
73	p.Ala73Asp	1.01	0.02	Neutral	0.90	-0.16	Neutral	0.95	-0.07	Neutral
73	p.Ala73Glu	0.93	-0.11	Neutral	1.02	0.03	Neutral	0.97	-0.04	Neutral
73	p.Ala73Ala	1.00	0.00	Neutral	1.00	0.00	Neutral	1.00	0.00	Neutral
73	p.Ala73Gly	1.11	0.15	Neutral	1.01	0.01	Neutral	1.06	0.08	Neutral
73	p.Ala73Val	1.12	0.17	Neutral	0.88	-0.18	Neutral	1.00	0.00	Neutral
73	p.Ala73Tyr	1.08	0.11	Neutral	1.16	0.21	Indeterminate	1.12	0.16	Neutral
73	p.Ala73Cys	1.09	0.12	Neutral	0.97	-0.04	Neutral	1.03	0.04	Neutral
73	p.Ala73Trp	1.20	0.26	Indeterminate	0.97	-0.05	Neutral	1.08	0.11	Neutral
73	p.Ala73Phe	0.88	-0.19	Neutral	1.02	0.03	Neutral	0.95	-0.08	Neutral
74	p.Asp74Asn	1.63	0.70	Indeterminate	1.89	0.92	Indeterminate	1.76	0.82	Indeterminate
74	p.Asp74Lys	12.10	3.60	Deleterious	7.36	2.88	Deleterious	9.73	3.28	Deleterious
74	p.Asp74Thr	5.11	2.35	Deleterious	3.90	1.97	Deleterious	4.51	2.17	Deleterious
74	p.Asp74Arg	11.51	3.52	Deleterious	6.76	2.76	Deleterious	9.14	3.19	Deleterious
74	p.Asp74Ser	1.13	0.18	Neutral	1.57	0.65	Indeterminate	1.35	0.43	Indeterminate
74	p.Asp74Ile	13.43	3.75	Deleterious	5.56	2.47	Deleterious	9.50	3.25	Deleterious
74	p.Asp74Met	6.01	2.59	Deleterious	4.26	2.09	Deleterious	5.13	2.36	Deleterious
74	p.Asp74His	1.14	0.19	Neutral	1.02	0.02	Neutral	1.08	0.11	Neutral
74	p.Asp74Gln	6.49	2.70	Deleterious	3.93	1.97	Deleterious	5.21	2.38	Deleterious
74	p.Asp74Pro	12.91	3.69	Deleterious	6.59	2.72	Deleterious	9.75	3.29	Deleterious
74	p.Asp74Leu	7.86	2.98	Deleterious	5.09	2.35	Deleterious	6.48	2.70	Deleterious
74	p.Asp74Asp	1.00	0.00	Neutral	1.00	0.00	Neutral	1.00	0.00	Neutral
74	p.Asp74Glu	0.50	-1.00	Neutral	0.41	-1.29	Neutral	0.45	-1.14	Neutral
74	p.Asp74Ala	3.04	1.60	Deleterious	2.47	1.30	Indeterminate	2.75	1.46	Deleterious
74	p.Asp74Gly	2.17	1.12	Deleterious	1.86	0.90	Indeterminate	2.02	1.01	Indeterminate
74	p.Asp74Val	9.32	3.22	Deleterious	5.20	2.38	Deleterious	7.26	2.86	Deleterious
74	p.Asp74Tyr	6.61	2.73	Deleterious	3.82	1.93	Deleterious	5.22	2.38	Deleterious
74	p.Asp74Cys	0.66	-0.61	Neutral	0.71	-0.50	Neutral	0.68	-0.55	Neutral
74	p.Asp74Trp	7.09	2.83	Deleterious	4.05	2.02	Deleterious	5.57	2.48	Deleterious
74	p.Asp74Phe	8.17	3.03	Deleterious	5.14	2.36	Deleterious	6.65	2.73	Deleterious
75	p.Pro75Asn	1.03	0.05	Neutral	1.13	0.17	Indeterminate	1.08	0.11	Neutral
75	p.Pro75Lys	1.24	0.31	Indeterminate	1.10	0.14	Neutral	1.17	0.23	Neutral
75	p.Pro75Thr	1.17	0.22	Neutral	0.98	-0.02	Neutral	1.07	0.10	Neutral
75	p.Pro75Arg	0.88	-0.18	Neutral	1.01	0.02	Neutral	0.95	-0.08	Neutral
75	p.Pro75Ser	1.15	0.20	Neutral	0.87	-0.20	Neutral	1.01	0.01	Neutral
75	p.Pro75Ile	0.94	-0.08	Neutral	1.18	0.24	Indeterminate	1.06	0.09	Neutral
75	p.Pro75Met	1.25	0.32	Indeterminate	1.06	0.09	Neutral	1.15	0.21	Neutral
75	p.Pro75His	0.94	-0.08	Neutral	0.77	-0.38	Neutral	0.86	-0.23	Neutral
75	p.Pro75Gln	1.19	0.25	Indeterminate	0.99	-0.02	Neutral	1.09	0.12	Neutral
75	p.Pro75Pro	1.00	0.00	Neutral	1.00	0.00	Neutral	1.00	0.00	Neutral
75	p.Pro75Leu	1.03	0.04	Neutral	0.96	-0.06	Neutral	0.99	-0.01	Neutral
75	p.Pro75Asp	1.03	0.04	Neutral	0.93	-0.10	Neutral	0.98	-0.03	Neutral
75	p.Pro75Glu	1.08	0.10	Neutral	0.90	-0.16	Neutral	0.99	-0.02	Neutral
75	p.Pro75Ala	1.07	0.10	Neutral	0.98	-0.03	Neutral	1.03	0.04	Neutral
75	p.Pro75Gly	1.01	0.02	Neutral	0.92	-0.13	Neutral	0.96	-0.05	Neutral
75	p.Pro75Val	1.07	0.09	Neutral	1.10	0.14	Neutral	1.08	0.12	Neutral
75	p.Pro75Tyr	1.05	0.07	Neutral	1.06	0.09	Neutral	1.06	0.08	Neutral
75	p.Pro75Cys	1.10	0.14	Neutral	0.92	-0.12	Neutral	1.01	0.02	Neutral
75	p.Pro75Trp	1.19	0.26	Indeterminate	1.12	0.17	Indeterminate	1.16	0.21	Neutral
75	p.Pro75Phe	1.06	0.08	Neutral	1.06	0.09	Neutral	1.06	0.08	Neutral
76	p.Ala76Asn	1.90	0.93	Indeterminate				1.90	0.93	Indeterminate
76	p.Ala76Lys	1.76	0.82	Indeterminate				1.76	0.82	Indeterminate
76	p.Ala76Thr	1.39	0.47	Indeterminate				1.39	0.47	Indeterminate
76	p.Ala76Arg	1.81	0.86	Indeterminate				1.81	0.86	Indeterminate
76	p.Ala76Ser	1.74	0.80	Indeterminate				1.74	0.80	Indeterminate
76	p.Ala76Ile	1.84	0.88	Indeterminate				1.84	0.88	Indeterminate
76	p.Ala76Met	1.38	0.47	Indeterminate				1.38	0.47	Indeterminate
76	p.Ala76His	1.66	0.73	Indeterminate				1.66	0.73	Indeterminate
76	p.Ala76Gln	1.79	0.84	Indeterminate				1.79	0.84	Indeterminate
76	p.Ala76Pro	1.54	0.62	Indeterminate				1.54	0.62	Indeterminate

76	p.Ala76Leu	1.66	0.73	Indeterminate	1.66	0.73	Indeterminate
76	p.Ala76Asp	1.55	0.63	Indeterminate	1.55	0.63	Indeterminate
76	p.Ala76Glu	1.76	0.82	Indeterminate	1.76	0.82	Indeterminate
76	p.Ala76Ala	1.00	0.00	Neutral	1.00	0.00	Neutral
76	p.Ala76Gly	1.45	0.53	Indeterminate	1.45	0.53	Indeterminate
76	p.Ala76Val	1.38	0.47	Indeterminate	1.38	0.47	Indeterminate
76	p.Ala76Tyr	1.44	0.52	Indeterminate	1.44	0.52	Indeterminate
76	p.Ala76Cys	2.00	1.00	Indeterminate	2.00	1.00	Indeterminate
76	p.Ala76Trp	1.38	0.47	Indeterminate	1.38	0.47	Indeterminate
76	p.Ala76Phe	1.62	0.70	Indeterminate	1.62	0.70	Indeterminate
77	p.Thr77Asn	1.46	0.55	Indeterminate	1.46	0.55	Indeterminate
77	p.Thr77Lys	1.55	0.63	Indeterminate	1.55	0.63	Indeterminate
77	p.Thr77Thr	1.00	0.00	Neutral	1.00	0.00	Neutral
77	p.Thr77Arg	1.70	0.77	Indeterminate	1.70	0.77	Indeterminate
77	p.Thr77Ser	1.51	0.60	Indeterminate	1.51	0.60	Indeterminate
77	p.Thr77Ile	1.27	0.34	Indeterminate	1.27	0.34	Indeterminate
77	p.Thr77Met	1.32	0.40	Indeterminate	1.32	0.40	Indeterminate
77	p.Thr77His	1.34	0.42	Indeterminate	1.34	0.42	Indeterminate
77	p.Thr77Gln	1.41	0.49	Indeterminate	1.41	0.49	Indeterminate
77	p.Thr77Pro	2.68	1.42	Deleterious	2.68	1.42	Deleterious
77	p.Thr77Leu	1.55	0.63	Indeterminate	1.55	0.63	Indeterminate
77	p.Thr77Asp	1.28	0.36	Indeterminate	1.28	0.36	Indeterminate
77	p.Thr77Glu	2.04	1.03	Indeterminate	2.04	1.03	Indeterminate
77	p.Thr77Ala	1.55	0.63	Indeterminate	1.55	0.63	Indeterminate
77	p.Thr77Gly	1.27	0.34	Indeterminate	1.27	0.34	Indeterminate
77	p.Thr77Val	1.76	0.82	Indeterminate	1.76	0.82	Indeterminate
77	p.Thr77Tyr	1.33	0.41	Indeterminate	1.33	0.41	Indeterminate
77	p.Thr77Cys	1.38	0.47	Indeterminate	1.38	0.47	Indeterminate
77	p.Thr77Trp	1.20	0.27	Indeterminate	1.20	0.27	Indeterminate
77	p.Thr77Phe	1.14	0.19	Neutral	1.14	0.19	Neutral
78	p.Leu78Asn	0.71	-0.49	Neutral	0.71	-0.49	Neutral
78	p.Leu78Lys	0.81	-0.31	Neutral	0.81	-0.31	Neutral
78	p.Leu78Thr	0.82	-0.29	Neutral	0.82	-0.29	Neutral
78	p.Leu78Arg	1.48	0.57	Indeterminate	1.48	0.57	Indeterminate
78	p.Leu78Ser	0.53	-0.91	Neutral	0.53	-0.91	Neutral
78	p.Leu78Ile	0.92	-0.13	Neutral	0.92	-0.13	Neutral
78	p.Leu78Met	1.36	0.45	Indeterminate	1.36	0.45	Indeterminate
78	p.Leu78His	0.98	-0.03	Neutral	0.98	-0.03	Neutral
78	p.Leu78Gln	1.26	0.33	Indeterminate	1.26	0.33	Indeterminate
78	p.Leu78Pro	1.60	0.68	Indeterminate	1.60	0.68	Indeterminate
78	p.Leu78Leu	1.00	0.00	Neutral	1.00	0.00	Neutral
78	p.Leu78Asp	0.45	-1.14	Neutral	0.45	-1.14	Neutral
78	p.Leu78Glu	1.24	0.31	Indeterminate	1.24	0.31	Indeterminate
78	p.Leu78Ala	1.23	0.30	Indeterminate	1.23	0.30	Indeterminate
78	p.Leu78Gly	0.18	-2.45	Neutral	0.18	-2.45	Neutral
78	p.Leu78Val	0.80	-0.32	Neutral	0.80	-0.32	Neutral
78	p.Leu78Tyr	1.13	0.18	Neutral	1.13	0.18	Neutral
78	p.Leu78Cys	1.03	0.04	Neutral	1.03	0.04	Neutral
78	p.Leu78Trp	0.71	-0.49	Neutral	0.71	-0.49	Neutral
78	p.Leu78Phe	0.67	-0.59	Neutral	0.67	-0.59	Neutral
79	p.Thr79Asn	0.82	-0.29	Neutral	0.82	-0.29	Neutral
79	p.Thr79Lys	0.95	-0.07	Neutral	0.95	-0.07	Neutral
79	p.Thr79Thr	1.00	0.00	Neutral	1.00	0.00	Neutral
79	p.Thr79Arg	1.24	0.31	Indeterminate	1.24	0.31	Indeterminate
79	p.Thr79Ser	0.83	-0.26	Neutral	0.83	-0.26	Neutral
79	p.Thr79Ile	0.78	-0.35	Neutral	0.78	-0.35	Neutral
79	p.Thr79Met	0.96	-0.06	Neutral	0.96	-0.06	Neutral
79	p.Thr79His	0.94	-0.09	Neutral	0.94	-0.09	Neutral
79	p.Thr79Gln	0.81	-0.31	Neutral	0.81	-0.31	Neutral
79	p.Thr79Pro	2.62	1.39	Deleterious	2.62	1.39	Deleterious
79	p.Thr79Leu	1.06	0.08	Neutral	1.06	0.08	Neutral
79	p.Thr79Asp	0.97	-0.04	Neutral	0.97	-0.04	Neutral
79	p.Thr79Glu	0.85	-0.24	Neutral	0.85	-0.24	Neutral
79	p.Thr79Ala	0.99	-0.02	Neutral	0.99	-0.02	Neutral
79	p.Thr79Gly	1.03	0.04	Neutral	1.03	0.04	Neutral
79	p.Thr79Val	1.04	0.06	Neutral	1.04	0.06	Neutral
79	p.Thr79Tyr	0.99	-0.02	Neutral	0.99	-0.02	Neutral
79	p.Thr79Cys	1.10	0.13	Neutral	1.10	0.13	Neutral
79	p.Thr79Trp	1.14	0.18	Neutral	1.14	0.18	Neutral
79	p.Thr79Phe	0.95	-0.08	Neutral	0.95	-0.08	Neutral
80	p.Arg80Asn	1.67	0.74	Indeterminate	1.67	0.74	Indeterminate
80	p.Arg80Lys	1.22	0.29	Indeterminate	1.22	0.29	Indeterminate
80	p.Arg80Thr	1.18	0.24	Indeterminate	1.18	0.24	Indeterminate
80	p.Arg80Arg	1.00	0.00	Neutral	1.00	0.00	Neutral
80	p.Arg80Ser	1.17	0.23	Neutral	1.17	0.23	Neutral
80	p.Arg80Ile	2.45	1.29	Deleterious	2.45	1.29	Deleterious
80	p.Arg80Met	1.45	0.54	Indeterminate	1.45	0.54	Indeterminate
80	p.Arg80His	1.31	0.39	Indeterminate	1.31	0.39	Indeterminate
80	p.Arg80Gln	1.54	0.62	Indeterminate	1.54	0.62	Indeterminate
80	p.Arg80Pro	4.66	2.22	Deleterious	4.66	2.22	Deleterious
80	p.Arg80Leu	1.47	0.56	Indeterminate	1.47	0.56	Indeterminate
80	p.Arg80Asp	1.61	0.69	Indeterminate	1.61	0.69	Indeterminate
80	p.Arg80Glu	1.16	0.21	Neutral	1.16	0.21	Neutral
80	p.Arg80Ala	1.32	0.40	Indeterminate	1.32	0.40	Indeterminate
80	p.Arg80Gly	1.34	0.42	Indeterminate	1.34	0.42	Indeterminate
80	p.Arg80Val	1.64	0.71	Indeterminate	1.64	0.71	Indeterminate
80	p.Arg80Tyr	1.46	0.55	Indeterminate	1.46	0.55	Indeterminate
80	p.Arg80Cys	0.88	-0.18	Neutral	0.88	-0.18	Neutral
80	p.Arg80Trp	1.47	0.55	Indeterminate	1.47	0.55	Indeterminate
80	p.Arg80Phe	1.55	0.63	Indeterminate	1.55	0.63	Indeterminate
81	p.Pro81Asn	4.72	2.24	Deleterious	4.72	2.24	Deleterious
81	p.Pro81Lys	6.35	2.67	Deleterious	6.35	2.67	Deleterious
81	p.Pro81Thr	2.72	1.44	Deleterious	2.72	1.44	Deleterious
81	p.Pro81Arg	4.70	2.23	Deleterious	4.70	2.23	Deleterious
81	p.Pro81Ser	3.30	1.72	Deleterious	3.30	1.72	Deleterious
81	p.Pro81Ile	3.52	1.81	Deleterious	3.52	1.81	Deleterious
81	p.Pro81Met	4.37	2.13	Deleterious	4.37	2.13	Deleterious
81	p.Pro81His	5.15	2.36	Deleterious	5.15	2.36	Deleterious
81	p.Pro81Gln	7.78	2.96	Deleterious	7.78	2.96	Deleterious
81	p.Pro81Pro	1.00	0.00	Neutral	1.00	0.00	Neutral
81	p.Pro81Leu	3.71	1.89	Deleterious	3.71	1.89	Deleterious

81	p.Pro81Asp	5.03	2.33	Deleterious	5.03	2.33	Deleterious
81	p.Pro81Glu	6.20	2.63	Deleterious	6.20	2.63	Deleterious
81	p.Pro81Ala	1.67	0.74	Indeterminate	1.67	0.74	Indeterminate
81	p.Pro81Gly	3.90	1.96	Deleterious	3.90	1.96	Deleterious
81	p.Pro81Val	2.62	1.39	Deleterious	2.62	1.39	Deleterious
81	p.Pro81Tyr	4.30	2.10	Deleterious	4.30	2.10	Deleterious
81	p.Pro81Cys	2.08	1.06	Indeterminate	2.08	1.06	Indeterminate
81	p.Pro81Trp	5.08	2.35	Deleterious	5.08	2.35	Deleterious
81	p.Pro81Phe	4.51	2.17	Deleterious	4.51	2.17	Deleterious
82	p.Val82Asn	2.35	1.23	Deleterious	2.35	1.23	Deleterious
82	p.Val82Lys	3.03	1.60	Deleterious	3.03	1.60	Deleterious
82	p.Val82Thr	1.50	0.58	Indeterminate	1.50	0.58	Indeterminate
82	p.Val82Arg	3.09	1.63	Deleterious	3.09	1.63	Deleterious
82	p.Val82Ser	1.36	0.44	Indeterminate	1.36	0.44	Indeterminate
82	p.Val82Ile	1.28	0.36	Indeterminate	1.28	0.36	Indeterminate
82	p.Val82Met	0.83	-0.27	Neutral	0.83	-0.27	Neutral
82	p.Val82His	2.45	1.29	Deleterious	2.45	1.29	Deleterious
82	p.Val82Gln	1.41	0.50	Indeterminate	1.41	0.50	Indeterminate
82	p.Val82Pro	2.04	1.03	Indeterminate	2.04	1.03	Indeterminate
82	p.Val82Leu	1.14	0.19	Neutral	1.14	0.19	Neutral
82	p.Val82Asp	2.78	1.48	Deleterious	2.78	1.48	Deleterious
82	p.Val82Glu	2.14	1.10	Deleterious	2.14	1.10	Deleterious
82	p.Val82Ala	1.01	0.01	Neutral	1.01	0.01	Neutral
82	p.Val82Gly	2.04	1.03	Indeterminate	2.04	1.03	Indeterminate
82	p.Val82Val	1.00	0.00	Neutral	1.00	0.00	Neutral
82	p.Val82Tyr	2.89	1.53	Deleterious	2.89	1.53	Deleterious
82	p.Val82Cys	1.33	0.41	Indeterminate	1.33	0.41	Indeterminate
82	p.Val82Trp	3.55	1.83	Deleterious	3.55	1.83	Deleterious
82	p.Val82Phe	1.80	0.84	Indeterminate	1.80	0.84	Indeterminate
83	p.His83Asn	7.57	2.92	Deleterious	7.57	2.92	Deleterious
83	p.His83Lys	10.58	3.40	Deleterious	10.58	3.40	Deleterious
83	p.His83Thr	6.28	2.65	Deleterious	6.28	2.65	Deleterious
83	p.His83Arg	11.18	3.48	Deleterious	11.18	3.48	Deleterious
83	p.His83Ser	6.08	2.60	Deleterious	6.08	2.60	Deleterious
83	p.His83Ile	6.54	2.71	Deleterious	6.54	2.71	Deleterious
83	p.His83Met	2.21	1.14	Deleterious	2.21	1.14	Deleterious
83	p.His83His	1.00	0.00	Neutral	1.00	0.00	Neutral
83	p.His83Gln	4.00	2.00	Deleterious	4.00	2.00	Deleterious
83	p.His83Pro	11.53	3.53	Deleterious	11.53	3.53	Deleterious
83	p.His83Leu	5.89	2.56	Deleterious	5.89	2.56	Deleterious
83	p.His83Asp	10.71	3.42	Deleterious	10.71	3.42	Deleterious
83	p.His83Glu	10.06	3.33	Deleterious	10.06	3.33	Deleterious
83	p.His83Ala	2.62	1.39	Deleterious	2.62	1.39	Deleterious
83	p.His83Gly	7.34	2.87	Deleterious	7.34	2.87	Deleterious
83	p.His83Val	9.70	3.28	Deleterious	9.70	3.28	Deleterious
83	p.His83Tyr	10.76	3.43	Deleterious	10.76	3.43	Deleterious
83	p.His83Cys	6.85	2.78	Deleterious	6.85	2.78	Deleterious
83	p.His83Trp	11.28	3.50	Deleterious	11.28	3.50	Deleterious
83	p.His83Phe	6.61	2.72	Deleterious	6.61	2.72	Deleterious
84	p.Asp84Asn	15.81	3.98	Deleterious	15.81	3.98	Deleterious
84	p.Asp84Lys	22.66	4.50	Deleterious	22.66	4.50	Deleterious
84	p.Asp84Thr	16.29	4.03	Deleterious	16.29	4.03	Deleterious
84	p.Asp84Arg	22.75	4.51	Deleterious	22.75	4.51	Deleterious
84	p.Asp84Ser	6.14	2.62	Deleterious	6.14	2.62	Deleterious
84	p.Asp84Ile	23.12	4.53	Deleterious	23.12	4.53	Deleterious
84	p.Asp84Met	19.75	4.30	Deleterious	19.75	4.30	Deleterious
84	p.Asp84His	9.00	3.17	Deleterious	9.00	3.17	Deleterious
84	p.Asp84Gln	11.40	3.51	Deleterious	11.40	3.51	Deleterious
84	p.Asp84Pro	22.54	4.49	Deleterious	22.54	4.49	Deleterious
84	p.Asp84Leu	23.46	4.55	Deleterious	23.46	4.55	Deleterious
84	p.Asp84Asp	1.00	0.00	Neutral	1.00	0.00	Neutral
84	p.Asp84Glu	1.50	0.59	Indeterminate	1.50	0.59	Indeterminate
84	p.Asp84Ala	17.18	4.10	Deleterious	17.18	4.10	Deleterious
84	p.Asp84Gly	19.24	4.27	Deleterious	19.24	4.27	Deleterious
84	p.Asp84Val	20.90	4.39	Deleterious	20.90	4.39	Deleterious
84	p.Asp84Tyr	19.97	4.32	Deleterious	19.97	4.32	Deleterious
84	p.Asp84Cys	5.90	2.56	Deleterious	5.90	2.56	Deleterious
84	p.Asp84Trp	24.74	4.63	Deleterious	24.74	4.63	Deleterious
84	p.Asp84Phe	21.67	4.44	Deleterious	21.67	4.44	Deleterious
85	p.Ala85Asn	1.29	0.37	Indeterminate	1.29	0.37	Indeterminate
85	p.Ala85Lys	2.25	1.17	Deleterious	2.25	1.17	Deleterious
85	p.Ala85Thr	1.44	0.53	Indeterminate	1.44	0.53	Indeterminate
85	p.Ala85Arg	2.24	1.16	Deleterious	2.24	1.16	Deleterious
85	p.Ala85Ser	1.08	0.11	Neutral	1.08	0.11	Neutral
85	p.Ala85Ile	1.56	0.64	Indeterminate	1.56	0.64	Indeterminate
85	p.Ala85Met	0.77	-0.37	Neutral	0.77	-0.37	Neutral
85	p.Ala85His	1.66	0.73	Indeterminate	1.66	0.73	Indeterminate
85	p.Ala85Gln	1.50	0.59	Indeterminate	1.50	0.59	Indeterminate
85	p.Ala85Pro	1.51	0.60	Indeterminate	1.51	0.60	Indeterminate
85	p.Ala85Leu	1.03	0.04	Neutral	1.03	0.04	Neutral
85	p.Ala85Asp	2.03	1.02	Indeterminate	2.03	1.02	Indeterminate
85	p.Ala85Glu	1.96	0.97	Indeterminate	1.96	0.97	Indeterminate
85	p.Ala85Ala	1.00	0.00	Neutral	1.00	0.00	Neutral
85	p.Ala85Gly	0.86	-0.22	Neutral	0.86	-0.22	Neutral
85	p.Ala85Val	1.33	0.41	Indeterminate	1.33	0.41	Indeterminate
85	p.Ala85Tyr	1.82	0.87	Indeterminate	1.82	0.87	Indeterminate
85	p.Ala85Cys	0.91	-0.14	Neutral	0.91	-0.14	Neutral
85	p.Ala85Trp	2.44	1.29	Deleterious	2.44	1.29	Deleterious
85	p.Ala85Phe	1.95	0.96	Indeterminate	1.95	0.96	Indeterminate
86	p.Ala86Asn	8.60	3.10	Deleterious	8.60	3.10	Deleterious
86	p.Ala86Lys	10.11	3.34	Deleterious	10.11	3.34	Deleterious
86	p.Ala86Thr	0.79	-0.34	Neutral	0.79	-0.34	Neutral
86	p.Ala86Arg	9.67	3.27	Deleterious	9.67	3.27	Deleterious
86	p.Ala86Ser	1.40	0.49	Indeterminate	1.40	0.49	Indeterminate
86	p.Ala86Ile	1.86	0.90	Indeterminate	1.86	0.90	Indeterminate
86	p.Ala86Met	7.54	2.92	Deleterious	7.54	2.92	Deleterious
86	p.Ala86His	8.51	3.09	Deleterious	8.51	3.09	Deleterious
86	p.Ala86Gln	9.27	3.21	Deleterious	9.27	3.21	Deleterious
86	p.Ala86Pro	8.62	3.11	Deleterious	8.62	3.11	Deleterious
86	p.Ala86Leu	7.57	2.92	Deleterious	7.57	2.92	Deleterious
86	p.Ala86Asp	8.99	3.17	Deleterious	8.99	3.17	Deleterious

86	p.Ala86Glu	9.11	3.19	Deleterious	9.11	3.19	Deleterious
86	p.Ala86Ala	1.00	0.00	Neutral	1.00	0.00	Neutral
86	p.Ala86Gly	0.86	-0.21	Neutral	0.86	-0.21	Neutral
86	p.Ala86Val	1.00	-0.01	Neutral	1.00	-0.01	Neutral
86	p.Ala86Tyr	9.41	3.23	Deleterious	9.41	3.23	Deleterious
86	p.Ala86Cys	0.66	-0.59	Neutral	0.66	-0.59	Neutral
86	p.Ala86Trp	8.43	3.08	Deleterious	8.43	3.08	Deleterious
86	p.Ala86Phe	8.66	3.11	Deleterious	8.66	3.11	Deleterious
87	p.Arg87Asn	1.10	0.13	Neutral	1.10	0.13	Neutral
87	p.Arg87Lys	0.39	-1.37	Neutral	0.39	-1.37	Neutral
87	p.Arg87Thr	1.06	0.08	Neutral	1.06	0.08	Neutral
87	p.Arg87Arg	1.00	0.00	Neutral	1.00	0.00	Neutral
87	p.Arg87Ser	2.13	1.09	Deleterious	2.13	1.09	Deleterious
87	p.Arg87Ile	1.13	0.17	Neutral	1.13	0.17	Neutral
87	p.Arg87Met	1.70	0.77	Indeterminate	1.70	0.77	Indeterminate
87	p.Arg87His	1.92	0.94	Indeterminate	1.92	0.94	Indeterminate
87	p.Arg87Gln	2.09	1.07	Indeterminate	2.09	1.07	Indeterminate
87	p.Arg87Pro	34.88	5.12	Deleterious	34.88	5.12	Deleterious
87	p.Arg87Leu	1.32	0.41	Indeterminate	1.32	0.41	Indeterminate
87	p.Arg87Asp	6.02	2.59	Deleterious	6.02	2.59	Deleterious
87	p.Arg87Glu	3.27	1.71	Deleterious	3.27	1.71	Deleterious
87	p.Arg87Ala	2.60	1.38	Deleterious	2.60	1.38	Deleterious
87	p.Arg87Gly	0.81	-0.30	Neutral	0.81	-0.30	Neutral
87	p.Arg87Val	1.15	0.20	Neutral	1.15	0.20	Neutral
87	p.Arg87Tyr	1.81	0.86	Indeterminate	1.81	0.86	Indeterminate
87	p.Arg87Cys	0.74	-0.43	Neutral	0.74	-0.43	Neutral
87	p.Arg87Trp	20.35	4.35	Deleterious	20.35	4.35	Deleterious
87	p.Arg87Phe	1.26	0.34	Indeterminate	1.26	0.34	Indeterminate
88	p.Glu88Asn	1.30	0.38	Indeterminate	1.30	0.38	Indeterminate
88	p.Glu88Lys	1.95	0.96	Indeterminate	1.95	0.96	Indeterminate
88	p.Glu88Thr	1.29	0.37	Indeterminate	1.29	0.37	Indeterminate
88	p.Glu88Arg	1.89	0.92	Indeterminate	1.89	0.92	Indeterminate
88	p.Glu88Ser	1.21	0.28	Indeterminate	1.21	0.28	Indeterminate
88	p.Glu88Ile	1.18	0.23	Neutral	1.18	0.23	Neutral
88	p.Glu88Met	1.17	0.23	Neutral	1.17	0.23	Neutral
88	p.Glu88His	1.40	0.49	Indeterminate	1.40	0.49	Indeterminate
88	p.Glu88Gln	1.27	0.35	Indeterminate	1.27	0.35	Indeterminate
88	p.Glu88Pro	3.13	1.65	Deleterious	3.13	1.65	Deleterious
88	p.Glu88Leu	1.37	0.45	Indeterminate	1.37	0.45	Indeterminate
88	p.Glu88Asp	1.36	0.44	Indeterminate	1.36	0.44	Indeterminate
88	p.Glu88Glu	1.00	0.00	Neutral	1.00	0.00	Neutral
88	p.Glu88Ala	1.08	0.10	Neutral	1.08	0.10	Neutral
88	p.Glu88Gly	1.20	0.26	Indeterminate	1.20	0.26	Indeterminate
88	p.Glu88Val	1.31	0.39	Indeterminate	1.31	0.39	Indeterminate
88	p.Glu88Tyr	1.34	0.42	Indeterminate	1.34	0.42	Indeterminate
88	p.Glu88Cys	1.15	0.21	Neutral	1.15	0.21	Neutral
88	p.Glu88Trp	1.20	0.26	Indeterminate	1.20	0.26	Indeterminate
88	p.Glu88Phe	1.41	0.49	Indeterminate	1.41	0.49	Indeterminate
89	p.Gly89Asn	1.78	0.83	Indeterminate	1.78	0.83	Indeterminate
89	p.Gly89Lys	7.99	3.00	Deleterious	7.99	3.00	Deleterious
89	p.Gly89Thr	13.14	3.72	Deleterious	13.14	3.72	Deleterious
89	p.Gly89Arg	8.98	3.17	Deleterious	8.98	3.17	Deleterious
89	p.Gly89Ser	4.06	2.02	Deleterious	4.06	2.02	Deleterious
89	p.Gly89Ile	16.30	4.03	Deleterious	16.30	4.03	Deleterious
89	p.Gly89Met	11.69	3.55	Deleterious	11.69	3.55	Deleterious
89	p.Gly89His	9.73	3.28	Deleterious	9.73	3.28	Deleterious
89	p.Gly89Gln	8.25	3.04	Deleterious	8.25	3.04	Deleterious
89	p.Gly89Pro	11.31	3.50	Deleterious	11.31	3.50	Deleterious
89	p.Gly89Leu	13.57	3.76	Deleterious	13.57	3.76	Deleterious
89	p.Gly89Asp	3.52	1.82	Deleterious	3.52	1.82	Deleterious
89	p.Gly89Glu	10.29	3.36	Deleterious	10.29	3.36	Deleterious
89	p.Gly89Ala	2.07	1.05	Indeterminate	2.07	1.05	Indeterminate
89	p.Gly89Gly	1.00	0.00	Neutral	1.00	0.00	Neutral
89	p.Gly89Val	14.96	3.90	Deleterious	14.96	3.90	Deleterious
89	p.Gly89Tyr	14.02	3.81	Deleterious	14.02	3.81	Deleterious
89	p.Gly89Cys	4.06	2.02	Deleterious	4.06	2.02	Deleterious
89	p.Gly89Trp	14.23	3.83	Deleterious	14.23	3.83	Deleterious
89	p.Gly89Phe	13.71	3.78	Deleterious	13.71	3.78	Deleterious
90	p.Phe90Asn	0.89	-0.17	Neutral	0.89	-0.17	Neutral
90	p.Phe90Lys	1.21	0.28	Indeterminate	1.21	0.28	Indeterminate
90	p.Phe90Thr	1.15	0.21	Neutral	1.15	0.21	Neutral
90	p.Phe90Arg	0.96	-0.06	Neutral	0.96	-0.06	Neutral
90	p.Phe90Ser	0.94	-0.08	Neutral	0.94	-0.08	Neutral
90	p.Phe90Ile	0.87	-0.20	Neutral	0.87	-0.20	Neutral
90	p.Phe90Met	0.75	-0.42	Neutral	0.75	-0.42	Neutral
90	p.Phe90His	0.78	-0.36	Neutral	0.78	-0.36	Neutral
90	p.Phe90Gln	0.76	-0.40	Neutral	0.76	-0.40	Neutral
90	p.Phe90Pro	1.62	0.70	Indeterminate	1.62	0.70	Indeterminate
90	p.Phe90Leu	0.78	-0.36	Neutral	0.78	-0.36	Neutral
90	p.Phe90Asp	1.73	0.79	Indeterminate	1.73	0.79	Indeterminate
90	p.Phe90Glu	1.15	0.21	Neutral	1.15	0.21	Neutral
90	p.Phe90Ala	0.84	-0.24	Neutral	0.84	-0.24	Neutral
90	p.Phe90Gly	1.01	0.02	Neutral	1.01	0.02	Neutral
90	p.Phe90Val	1.11	0.15	Neutral	1.11	0.15	Neutral
90	p.Phe90Tyr	0.80	-0.32	Neutral	0.80	-0.32	Neutral
90	p.Phe90Cys	0.99	-0.01	Neutral	0.99	-0.01	Neutral
90	p.Phe90Trp	0.93	-0.10	Neutral	0.93	-0.10	Neutral
90	p.Phe90Phe	1.00	0.00	Neutral	1.00	0.00	Neutral
91	p.Leu91Asn	0.70	-0.51	Neutral	0.70	-0.51	Neutral
91	p.Leu91Lys	1.43	0.52	Indeterminate	1.43	0.52	Indeterminate
91	p.Leu91Thr	1.46	0.54	Indeterminate	1.46	0.54	Indeterminate
91	p.Leu91Arg	1.16	0.21	Neutral	1.16	0.21	Neutral
91	p.Leu91Ser	1.94	0.96	Indeterminate	1.94	0.96	Indeterminate
91	p.Leu91Ile	1.70	0.76	Indeterminate	1.70	0.76	Indeterminate
91	p.Leu91Met	2.60	1.38	Deleterious	2.60	1.38	Deleterious
91	p.Leu91His	1.68	0.75	Indeterminate	1.68	0.75	Indeterminate
91	p.Leu91Gln	0.79	-0.33	Neutral	0.79	-0.33	Neutral
91	p.Leu91Pro	1.53	0.61	Indeterminate	1.53	0.61	Indeterminate
91	p.Leu91Leu	1.00	0.00	Neutral	1.00	0.00	Neutral
91	p.Leu91Asp	1.31	0.39	Indeterminate	1.31	0.39	Indeterminate
91	p.Leu91Glu	1.34	0.43	Indeterminate	1.34	0.43	Indeterminate

91	p.Leu91Ala	1.03	0.04	Neutral				1.03	0.04	Neutral
91	p.Leu91Gly	1.39	0.47	Indeterminate				1.39	0.47	Indeterminate
91	p.Leu91Val	1.29	0.37	Indeterminate				1.29	0.37	Indeterminate
91	p.Leu91Tyr	3.09	1.63	Deleterious				3.09	1.63	Deleterious
91	p.Leu91Cys	0.96	-0.05	Neutral				0.96	-0.05	Neutral
91	p.Leu91Trp	1.59	0.67	Indeterminate				1.59	0.67	Indeterminate
91	p.Leu91Phe	2.11	1.08	Indeterminate				2.11	1.08	Indeterminate
92	p.Asp92Asn	1.06	0.08	Neutral				1.06	0.08	Neutral
92	p.Asp92Lys	1.23	0.30	Indeterminate				1.23	0.30	Indeterminate
92	p.Asp92Thr	0.90	-0.16	Neutral				0.90	-0.16	Neutral
92	p.Asp92Arg	1.02	0.03	Neutral				1.02	0.03	Neutral
92	p.Asp92Ser	0.97	-0.05	Neutral				0.97	-0.05	Neutral
92	p.Asp92Ile	1.19	0.25	Indeterminate				1.19	0.25	Indeterminate
92	p.Asp92Met	0.98	-0.03	Neutral				0.98	-0.03	Neutral
92	p.Asp92His	1.09	0.13	Neutral				1.09	0.13	Neutral
92	p.Asp92Gln	1.04	0.06	Neutral				1.04	0.06	Neutral
92	p.Asp92Pro	1.11	0.15	Neutral				1.11	0.15	Neutral
92	p.Asp92Leu	1.02	0.02	Neutral				1.02	0.02	Neutral
92	p.Asp92Asp	1.00	0.00	Neutral				1.00	0.00	Neutral
92	p.Asp92Glu	1.10	0.14	Neutral				1.10	0.14	Neutral
92	p.Asp92Ala	1.12	0.17	Neutral				1.12	0.17	Neutral
92	p.Asp92Gly	1.17	0.22	Neutral				1.17	0.22	Neutral
92	p.Asp92Val	1.03	0.04	Neutral				1.03	0.04	Neutral
92	p.Asp92Tyr	1.04	0.05	Neutral				1.04	0.05	Neutral
92	p.Asp92Cys	0.98	-0.03	Neutral				0.98	-0.03	Neutral
92	p.Asp92Trp	1.05	0.08	Neutral				1.05	0.08	Neutral
92	p.Asp92Phe	1.21	0.28	Indeterminate				1.21	0.28	Indeterminate
93	p.Thr93Asn	1.83	0.87	Indeterminate				1.83	0.87	Indeterminate
93	p.Thr93Lys	3.80	1.93	Deleterious				3.80	1.93	Deleterious
93	p.Thr93Thr	1.00	0.00	Neutral				1.00	0.00	Neutral
93	p.Thr93Arg	2.46	1.30	Deleterious				2.46	1.30	Deleterious
93	p.Thr93Ser	1.46	0.55	Indeterminate				1.46	0.55	Indeterminate
93	p.Thr93Ile	0.98	-0.03	Neutral				0.98	-0.03	Neutral
93	p.Thr93Met	1.77	0.82	Indeterminate				1.77	0.82	Indeterminate
93	p.Thr93His	2.45	1.29	Deleterious				2.45	1.29	Deleterious
93	p.Thr93Gln	2.20	1.14	Deleterious				2.20	1.14	Deleterious
93	p.Thr93Pro	6.26	2.65	Deleterious				6.26	2.65	Deleterious
93	p.Thr93Leu	1.62	0.70	Indeterminate				1.62	0.70	Indeterminate
93	p.Thr93Asp	2.94	1.56	Deleterious				2.94	1.56	Deleterious
93	p.Thr93Glu	2.43	1.28	Deleterious				2.43	1.28	Deleterious
93	p.Thr93Ala	2.32	1.21	Deleterious				2.32	1.21	Deleterious
93	p.Thr93Gly	2.45	1.29	Deleterious				2.45	1.29	Deleterious
93	p.Thr93Val	1.45	0.53	Indeterminate				1.45	0.53	Indeterminate
93	p.Thr93Tyr	2.87	1.52	Deleterious				2.87	1.52	Deleterious
93	p.Thr93Cys	1.63	0.70	Indeterminate				1.63	0.70	Indeterminate
93	p.Thr93Trp	2.31	1.21	Deleterious				2.31	1.21	Deleterious
93	p.Thr93Phe	1.98	0.98	Indeterminate				1.98	0.98	Indeterminate
94	p.Leu94Asn	2.32	1.21	Deleterious				2.32	1.21	Deleterious
94	p.Leu94Lys	6.25	2.64	Deleterious				6.25	2.64	Deleterious
94	p.Leu94Thr	2.48	1.31	Deleterious				2.48	1.31	Deleterious
94	p.Leu94Arg	8.16	3.03	Deleterious				8.16	3.03	Deleterious
94	p.Leu94Ser	0.96	-0.06	Neutral				0.96	-0.06	Neutral
94	p.Leu94Ile	0.69	-0.54	Neutral				0.69	-0.54	Neutral
94	p.Leu94Met	0.42	-1.25	Neutral				0.42	-1.25	Neutral
94	p.Leu94His	8.80	3.14	Deleterious				8.80	3.14	Deleterious
94	p.Leu94Gln	4.83	2.27	Deleterious				4.83	2.27	Deleterious
94	p.Leu94Pro	5.87	2.55	Deleterious				5.87	2.55	Deleterious
94	p.Leu94Leu	1.00	0.00	Neutral				1.00	0.00	Neutral
94	p.Leu94Asp	7.73	2.95	Deleterious				7.73	2.95	Deleterious
94	p.Leu94Glu	4.47	2.16	Deleterious				4.47	2.16	Deleterious
94	p.Leu94Ala	2.30	1.20	Deleterious				2.30	1.20	Deleterious
94	p.Leu94Gly	4.46	2.16	Deleterious				4.46	2.16	Deleterious
94	p.Leu94Val	1.22	0.28	Indeterminate				1.22	0.28	Indeterminate
94	p.Leu94Tyr	9.51	3.25	Deleterious				9.51	3.25	Deleterious
94	p.Leu94Cys	1.83	0.87	Indeterminate				1.83	0.87	Indeterminate
94	p.Leu94Trp	7.26	2.86	Deleterious				7.26	2.86	Deleterious
94	p.Leu94Phe	1.87	0.91	Indeterminate				1.87	0.91	Indeterminate
95	p.Val95Asn	1.08	0.11	Neutral				1.08	0.11	Neutral
95	p.Val95Lys	0.93	-0.10	Neutral				0.93	-0.10	Neutral
95	p.Val95Thr	0.99	-0.01	Neutral				0.99	-0.01	Neutral
95	p.Val95Arg	1.09	0.13	Neutral				1.09	0.13	Neutral
95	p.Val95Ser	1.08	0.11	Neutral				1.08	0.11	Neutral
95	p.Val95Ile	0.99	-0.02	Neutral				0.99	-0.02	Neutral
95	p.Val95Met	1.00	0.00	Neutral				1.00	0.00	Neutral
95	p.Val95His	0.78	-0.36	Neutral				0.78	-0.36	Neutral
95	p.Val95Gln	0.98	-0.03	Neutral				0.98	-0.03	Neutral
95	p.Val95Pro	4.78	2.26	Deleterious				4.78	2.26	Deleterious
95	p.Val95Leu	0.82	-0.28	Neutral				0.82	-0.28	Neutral
95	p.Val95Asp	0.96	-0.06	Neutral				0.96	-0.06	Neutral
95	p.Val95Glu	1.05	0.08	Neutral				1.05	0.08	Neutral
95	p.Val95Ala	0.74	-0.44	Neutral				0.74	-0.44	Neutral
95	p.Val95Gly	1.03	0.04	Neutral				1.03	0.04	Neutral
95	p.Val95Val	1.00	0.00	Neutral				1.00	0.00	Neutral
95	p.Val95Tyr	1.02	0.02	Neutral				1.02	0.02	Neutral
95	p.Val95Cys	0.82	-0.28	Neutral				0.82	-0.28	Neutral
95	p.Val95Trp	1.21	0.27	Indeterminate				1.21	0.27	Indeterminate
95	p.Val95Phe	1.11	0.15	Neutral				1.11	0.15	Neutral
96	p.Val96Asn	0.75	-0.41	Neutral	1.22	0.29	Indeterminate	0.99	-0.02	Neutral
96	p.Val96Lys	0.89	-0.17	Neutral	0.94	-0.09	Neutral	0.92	-0.13	Neutral
96	p.Val96Thr	0.69	-0.53	Neutral	0.92	-0.11	Neutral	0.81	-0.31	Neutral
96	p.Val96Arg	0.85	-0.24	Neutral	1.02	0.02	Neutral	0.93	-0.10	Neutral
96	p.Val96Ser	0.75	-0.41	Neutral	0.78	-0.35	Neutral	0.77	-0.38	Neutral
96	p.Val96Ile	0.84	-0.25	Neutral	1.01	0.02	Neutral	0.93	-0.11	Neutral
96	p.Val96Met	0.70	-0.52	Neutral	0.82	-0.29	Neutral	0.76	-0.40	Neutral
96	p.Val96His	0.93	-0.10	Neutral	0.92	-0.13	Neutral	0.93	-0.11	Neutral
96	p.Val96Gln	0.86	-0.23	Neutral	1.10	0.14	Indeterminate	0.98	-0.03	Neutral
96	p.Val96Pro	0.93	-0.10	Neutral	0.87	-0.20	Neutral	0.90	-0.15	Neutral
96	p.Val96Leu	0.87	-0.20	Neutral	1.21	0.28	Indeterminate	1.04	0.06	Neutral
96	p.Val96Asp	0.82	-0.29	Neutral	1.26	0.33	Indeterminate	1.04	0.05	Neutral
96	p.Val96Glu	0.65	-0.63	Neutral	1.05	0.08	Neutral	0.85	-0.23	Neutral
96	p.Val96Ala	1.10	0.14	Neutral	1.53	0.62	Indeterminate	1.32	0.40	Indeterminate

96	p.Val96Gly	1.13	0.18	Neutral	0.86	-0.22	Neutral	0.99	-0.01	Neutral
96	p.Val96Val	1.00	0.00	Neutral	1.00	0.00	Neutral	1.00	0.00	Neutral
96	p.Val96Tyr	0.82	-0.28	Neutral	1.08	0.11	Neutral	0.95	-0.07	Neutral
96	p.Val96Cys	0.82	-0.28	Neutral	1.37	0.45	Indeterminate	1.10	0.13	Neutral
96	p.Val96Trp	0.70	-0.51	Neutral	1.46	0.54	Indeterminate	1.08	0.11	Neutral
96	p.Val96Phe	0.72	-0.48	Neutral	1.08	0.11	Neutral	0.90	-0.15	Neutral
97	p.Leu97Asn	7.43	2.89	Deleterious				7.43	2.89	Deleterious
97	p.Leu97Lys	8.27	3.05	Deleterious				8.27	3.05	Deleterious
97	p.Leu97Thr	5.89	2.56	Deleterious				5.89	2.56	Deleterious
97	p.Leu97Arg	8.53	3.09	Deleterious				8.53	3.09	Deleterious
97	p.Leu97Ser	7.64	2.93	Deleterious				7.64	2.93	Deleterious
97	p.Leu97Ile	0.85	-0.24	Neutral				0.85	-0.24	Neutral
97	p.Leu97Met	0.69	-0.53	Neutral				0.69	-0.53	Neutral
97	p.Leu97His	6.20	2.63	Deleterious				6.20	2.63	Deleterious
97	p.Leu97Gln	4.19	2.07	Deleterious				4.19	2.07	Deleterious
97	p.Leu97Pro	8.72	3.12	Deleterious				8.72	3.12	Deleterious
97	p.Leu97Leu	1.00	0.00	Neutral				1.00	0.00	Neutral
97	p.Leu97Asp	8.74	3.13	Deleterious				8.74	3.13	Deleterious
97	p.Leu97Glu	6.81	2.77	Deleterious				6.81	2.77	Deleterious
97	p.Leu97Ala	4.79	2.26	Deleterious				4.79	2.26	Deleterious
97	p.Leu97Gly	8.76	3.13	Deleterious				8.76	3.13	Deleterious
97	p.Leu97Val	1.68	0.75	Indeterminate				1.68	0.75	Indeterminate
97	p.Leu97Tyr	1.01	0.01	Neutral				1.01	0.01	Neutral
97	p.Leu97Cys	1.03	0.04	Neutral				1.03	0.04	Neutral
97	p.Leu97Trp	6.72	2.75	Deleterious				6.72	2.75	Deleterious
97	p.Leu97Phe	0.92	-0.12	Neutral				0.92	-0.12	Neutral
98	p.His98Asn	1.02	0.02	Neutral				1.02	0.02	Neutral
98	p.His98Lys	0.92	-0.12	Neutral				0.92	-0.12	Neutral
98	p.His98Thr	1.07	0.10	Neutral				1.07	0.10	Neutral
98	p.His98Arg	1.00	0.00	Neutral				1.00	0.00	Neutral
98	p.His98Ser	1.29	0.37	Indeterminate				1.29	0.37	Indeterminate
98	p.His98Ile	0.98	-0.03	Neutral				0.98	-0.03	Neutral
98	p.His98Met	0.97	-0.04	Neutral				0.97	-0.04	Neutral
98	p.His98His	1.00	0.00	Neutral				1.00	0.00	Neutral
98	p.His98Gln	1.19	0.25	Indeterminate				1.19	0.25	Indeterminate
98	p.His98Pro	5.39	2.43	Deleterious				5.39	2.43	Deleterious
98	p.His98Leu	1.15	0.21	Neutral				1.15	0.21	Neutral
98	p.His98Asp	1.26	0.34	Indeterminate				1.26	0.34	Indeterminate
98	p.His98Glu	1.04	0.06	Neutral				1.04	0.06	Neutral
98	p.His98Ala	0.93	-0.10	Neutral				0.93	-0.10	Neutral
98	p.His98Gly	1.16	0.21	Neutral				1.16	0.21	Neutral
98	p.His98Val	1.07	0.10	Neutral				1.07	0.10	Neutral
98	p.His98Tyr	1.07	0.10	Neutral				1.07	0.10	Neutral
98	p.His98Cys	1.09	0.12	Neutral				1.09	0.12	Neutral
98	p.His98Trp	0.94	-0.10	Neutral				0.94	-0.10	Neutral
98	p.His98Phe	0.94	-0.08	Neutral				0.94	-0.08	Neutral
99	p.Arg99Asn	1.39	0.47	Indeterminate				1.39	0.47	Indeterminate
99	p.Arg99Lys	1.20	0.26	Indeterminate				1.20	0.26	Indeterminate
99	p.Arg99Thr	1.46	0.55	Indeterminate				1.46	0.55	Indeterminate
99	p.Arg99Arg	1.00	0.00	Neutral				1.00	0.00	Neutral
99	p.Arg99Ser	1.46	0.54	Indeterminate				1.46	0.54	Indeterminate
99	p.Arg99Ile	1.26	0.33	Indeterminate				1.26	0.33	Indeterminate
99	p.Arg99Met	1.60	0.68	Indeterminate				1.60	0.68	Indeterminate
99	p.Arg99His	0.94	-0.09	Neutral				0.94	-0.09	Neutral
99	p.Arg99Gln	0.92	-0.11	Neutral				0.92	-0.11	Neutral
99	p.Arg99Pro	38.44	5.26	Deleterious				38.44	5.26	Deleterious
99	p.Arg99Leu	1.72	0.78	Indeterminate				1.72	0.78	Indeterminate
99	p.Arg99Asp	1.92	0.94	Indeterminate				1.92	0.94	Indeterminate
99	p.Arg99Glu	1.40	0.48	Indeterminate				1.40	0.48	Indeterminate
99	p.Arg99Ala	2.31	1.21	Deleterious				2.31	1.21	Deleterious
99	p.Arg99Gly	1.02	0.03	Neutral				1.02	0.03	Neutral
99	p.Arg99Val	2.27	1.18	Deleterious				2.27	1.18	Deleterious
99	p.Arg99Tyr	1.06	0.09	Neutral				1.06	0.09	Neutral
99	p.Arg99Cys	1.77	0.82	Indeterminate				1.77	0.82	Indeterminate
99	p.Arg99Trp	1.83	0.87	Indeterminate				1.83	0.87	Indeterminate
99	p.Arg99Phe	1.14	0.19	Neutral				1.14	0.19	Neutral
100	p.Ala100Asn	1.10	0.13	Neutral				1.10	0.13	Neutral
100	p.Ala100Lys	0.94	-0.09	Neutral				0.94	-0.09	Neutral
100	p.Ala100Thr	1.34	0.42	Indeterminate				1.34	0.42	Indeterminate
100	p.Ala100Arg	0.92	-0.12	Neutral				0.92	-0.12	Neutral
100	p.Ala100Ser	0.74	-0.44	Neutral				0.74	-0.44	Neutral
100	p.Ala100Ile	1.05	0.06	Neutral				1.05	0.06	Neutral
100	p.Ala100Met	1.30	0.38	Indeterminate				1.30	0.38	Indeterminate
100	p.Ala100His	0.79	-0.35	Neutral				0.79	-0.35	Neutral
100	p.Ala100Gln	1.02	0.03	Neutral				1.02	0.03	Neutral
100	p.Ala100Pro	13.53	3.76	Deleterious				13.53	3.76	Deleterious
100	p.Ala100Leu	0.87	-0.20	Neutral				0.87	-0.20	Neutral
100	p.Ala100Asp	0.73	-0.45	Neutral				0.73	-0.45	Neutral
100	p.Ala100Glu	1.75	0.81	Indeterminate				1.75	0.81	Indeterminate
100	p.Ala100Ala	1.00	0.00	Neutral				1.00	0.00	Neutral
100	p.Ala100Gly	3.88	1.95	Deleterious				3.88	1.95	Deleterious
100	p.Ala100Val	0.82	-0.29	Neutral				0.82	-0.29	Neutral
100	p.Ala100Tyr	1.16	0.22	Neutral				1.16	0.22	Neutral
100	p.Ala100Cys	0.64	-0.64	Neutral				0.64	-0.64	Neutral
100	p.Ala100Trp	0.77	-0.38	Neutral				0.77	-0.38	Neutral
100	p.Ala100Phe	0.74	-0.44	Neutral				0.74	-0.44	Neutral
101	p.Gly101Asn	1.44	0.52	Indeterminate				1.44	0.52	Indeterminate
101	p.Gly101Lys	1.85	0.89	Indeterminate				1.85	0.89	Indeterminate
101	p.Gly101Thr	2.15	1.11	Deleterious				2.15	1.11	Deleterious
101	p.Gly101Arg	1.68	0.75	Indeterminate				1.68	0.75	Indeterminate
101	p.Gly101Ser	1.58	0.66	Indeterminate				1.58	0.66	Indeterminate
101	p.Gly101Ile	9.59	3.26	Deleterious				9.59	3.26	Deleterious
101	p.Gly101Met	2.07	1.05	Indeterminate				2.07	1.05	Indeterminate
101	p.Gly101His	1.59	0.67	Indeterminate				1.59	0.67	Indeterminate
101	p.Gly101Gln	1.58	0.66	Indeterminate				1.58	0.66	Indeterminate
101	p.Gly101Pro	8.50	3.09	Deleterious				8.50	3.09	Deleterious
101	p.Gly101Leu	3.51	1.81	Deleterious				3.51	1.81	Deleterious
101	p.Gly101Asp	1.31	0.39	Indeterminate				1.31	0.39	Indeterminate
101	p.Gly101Glu	1.85	0.89	Indeterminate				1.85	0.89	Indeterminate
101	p.Gly101Ala	1.61	0.68	Indeterminate				1.61	0.68	Indeterminate
101	p.Gly101Gly	1.00	0.00	Neutral				1.00	0.00	Neutral

101	p.Gly101Val	8.71	3.12	Deleterious				8.71	3.12	Deleterious
101	p.Gly101Tyr	2.53	1.34	Deleterious				2.53	1.34	Deleterious
101	p.Gly101Cys	1.65	0.72	Indeterminate				1.65	0.72	Indeterminate
101	p.Gly101Trp	4.49	2.17	Deleterious				4.49	2.17	Deleterious
101	p.Gly101Phe	2.19	1.13	Deleterious				2.19	1.13	Deleterious
102	p.Ala102Asn	10.55	3.40	Deleterious				10.55	3.40	Deleterious
102	p.Ala102Lys	27.85	4.80	Deleterious				27.85	4.80	Deleterious
102	p.Ala102Thr	11.34	3.50	Deleterious				11.34	3.50	Deleterious
102	p.Ala102Arg	46.34	5.53	Deleterious				46.34	5.53	Deleterious
102	p.Ala102Ser	2.38	1.25	Deleterious				2.38	1.25	Deleterious
102	p.Ala102Ile	18.46	4.21	Deleterious				18.46	4.21	Deleterious
102	p.Ala102Met	27.54	4.78	Deleterious				27.54	4.78	Deleterious
102	p.Ala102His	17.65	4.14	Deleterious				17.65	4.14	Deleterious
102	p.Ala102Gln	25.99	4.70	Deleterious				25.99	4.70	Deleterious
102	p.Ala102Pro	30.62	4.94	Deleterious				30.62	4.94	Deleterious
102	p.Ala102Leu	39.02	5.29	Deleterious				39.02	5.29	Deleterious
102	p.Ala102Asp	27.75	4.79	Deleterious				27.75	4.79	Deleterious
102	p.Ala102Glu	36.84	5.20	Deleterious				36.84	5.20	Deleterious
102	p.Ala102Ala	1.00	0.00	Neutral				1.00	0.00	Neutral
102	p.Ala102Gly	0.56	-0.83	Neutral				0.56	-0.83	Neutral
102	p.Ala102Val	2.29	1.20	Deleterious				2.29	1.20	Deleterious
102	p.Ala102Tyr	44.70	5.48	Deleterious				44.70	5.48	Deleterious
102	p.Ala102Cys	1.34	0.42	Indeterminate				1.34	0.42	Indeterminate
102	p.Ala102Trp	33.48	5.07	Deleterious				33.48	5.07	Deleterious
102	p.Ala102Phe	38.33	5.26	Deleterious				38.33	5.26	Deleterious
103	p.Arg103Asn	0.96	-0.06	Neutral				0.96	-0.06	Neutral
103	p.Arg103Lys	1.02	0.02	Neutral				1.02	0.02	Neutral
103	p.Arg103Thr	1.50	0.59	Indeterminate				1.50	0.59	Indeterminate
103	p.Arg103Arg	1.00	0.00	Neutral				1.00	0.00	Neutral
103	p.Arg103Ser	0.63	-0.67	Neutral				0.63	-0.67	Neutral
103	p.Arg103Ile	1.31	0.39	Indeterminate				1.31	0.39	Indeterminate
103	p.Arg103Met	1.03	0.04	Neutral				1.03	0.04	Neutral
103	p.Arg103His	0.82	-0.28	Neutral				0.82	-0.28	Neutral
103	p.Arg103Gln	0.99	-0.02	Neutral				0.99	-0.02	Neutral
103	p.Arg103Pro	0.81	-0.30	Neutral				0.81	-0.30	Neutral
103	p.Arg103Leu	0.65	-0.62	Neutral				0.65	-0.62	Neutral
103	p.Arg103Asp	0.73	-0.45	Neutral				0.73	-0.45	Neutral
103	p.Arg103Glu	0.80	-0.33	Neutral				0.80	-0.33	Neutral
103	p.Arg103Ala	0.81	-0.31	Neutral				0.81	-0.31	Neutral
103	p.Arg103Gly	0.96	-0.05	Neutral				0.96	-0.05	Neutral
103	p.Arg103Val	0.85	-0.23	Neutral				0.85	-0.23	Neutral
103	p.Arg103Tyr	1.09	0.12	Neutral				1.09	0.12	Neutral
103	p.Arg103Cys	1.05	0.08	Neutral				1.05	0.08	Neutral
103	p.Arg103Trp	0.90	-0.16	Neutral				0.90	-0.16	Neutral
103	p.Arg103Phe	0.92	-0.12	Neutral				0.92	-0.12	Neutral
104	p.Leu104Asn	0.44	-1.17	Neutral				0.44	-1.17	Neutral
104	p.Leu104Lys	2.06	1.04	Indeterminate				2.06	1.04	Indeterminate
104	p.Leu104Thr	0.46	-1.11	Neutral				0.46	-1.11	Neutral
104	p.Leu104Arg	4.06	2.02	Deleterious				4.06	2.02	Deleterious
104	p.Leu104Ser	0.37	-1.43	Neutral				0.37	-1.43	Neutral
104	p.Leu104Ile	0.22	-2.16	Neutral				0.22	-2.16	Neutral
104	p.Leu104Met	0.23	-2.10	Neutral				0.23	-2.10	Neutral
104	p.Leu104His	0.30	-1.73	Neutral				0.30	-1.73	Neutral
104	p.Leu104Gln	0.74	-0.43	Neutral				0.74	-0.43	Neutral
104	p.Leu104Pro	0.51	-0.99	Neutral				0.51	-0.99	Neutral
104	p.Leu104Leu	1.00	0.00	Neutral				1.00	0.00	Neutral
104	p.Leu104Asp	0.46	-1.13	Neutral				0.46	-1.13	Neutral
104	p.Leu104Glu	0.72	-0.48	Neutral				0.72	-0.48	Neutral
104	p.Leu104Ala	0.20	-2.33	Neutral				0.20	-2.33	Neutral
104	p.Leu104Gly	4.15	2.05	Deleterious				4.15	2.05	Deleterious
104	p.Leu104Val	0.14	-2.87	Neutral				0.14	-2.87	Neutral
104	p.Leu104Tyr	0.20	-2.29	Neutral				0.20	-2.29	Neutral
104	p.Leu104Cys	0.24	-2.04	Neutral				0.24	-2.04	Neutral
104	p.Leu104Trp	0.10	-3.32	Neutral				0.10	-3.32	Neutral
104	p.Leu104Phe	0.10	-3.34	Neutral				0.10	-3.34	Neutral
105	p.Asp105Asn	1.03	0.05	Neutral				1.03	0.05	Neutral
105	p.Asp105Lys	1.92	0.94	Indeterminate				1.92	0.94	Indeterminate
105	p.Asp105Thr	1.45	0.53	Indeterminate				1.45	0.53	Indeterminate
105	p.Asp105Arg	2.45	1.29	Deleterious				2.45	1.29	Deleterious
105	p.Asp105Ser	2.08	1.06	Indeterminate				2.08	1.06	Indeterminate
105	p.Asp105Ile	2.64	1.40	Deleterious				2.64	1.40	Deleterious
105	p.Asp105Met	1.49	0.57	Indeterminate				1.49	0.57	Indeterminate
105	p.Asp105His	1.65	0.72	Indeterminate				1.65	0.72	Indeterminate
105	p.Asp105Gln	1.87	0.90	Indeterminate				1.87	0.90	Indeterminate
105	p.Asp105Pro	9.48	3.25	Deleterious				9.48	3.25	Deleterious
105	p.Asp105Leu	1.85	0.89	Indeterminate				1.85	0.89	Indeterminate
105	p.Asp105Asp	1.00	0.00	Neutral				1.00	0.00	Neutral
105	p.Asp105Glu	2.10	1.07	Indeterminate				2.10	1.07	Indeterminate
105	p.Asp105Ala	1.39	0.48	Indeterminate				1.39	0.48	Indeterminate
105	p.Asp105Gly	2.69	1.43	Deleterious				2.69	1.43	Deleterious
105	p.Asp105Val	2.38	1.25	Deleterious				2.38	1.25	Deleterious
105	p.Asp105Tyr	0.54	-0.90	Neutral				0.54	-0.90	Neutral
105	p.Asp105Cys	0.58	-0.79	Neutral				0.58	-0.79	Neutral
105	p.Asp105Trp	1.37	0.46	Indeterminate				1.37	0.46	Indeterminate
105	p.Asp105Phe	1.80	0.85	Indeterminate				1.80	0.85	Indeterminate
106	p.Val106Asn	1.34	0.43	Indeterminate	1.20	0.26	Indeterminate	1.27	0.35	Indeterminate
106	p.Val106Lys	1.74	0.80	Indeterminate	1.13	0.17	Indeterminate	1.43	0.52	Indeterminate
106	p.Val106Thr	0.96	-0.05	Neutral	1.03	0.04	Neutral	1.00	-0.01	Neutral
106	p.Val106Arg	1.68	0.75	Indeterminate	0.93	-0.11	Neutral	1.30	0.38	Indeterminate
106	p.Val106Ser	1.08	0.11	Neutral	0.98	-0.03	Neutral	1.03	0.04	Neutral
106	p.Val106Ile	0.67	-0.57	Neutral	1.16	0.22	Indeterminate	0.92	-0.12	Neutral
106	p.Val106Met	1.00	0.00	Neutral	1.06	0.08	Neutral	1.03	0.04	Neutral
106	p.Val106His	0.96	-0.06	Neutral	1.06	0.08	Neutral	1.01	0.02	Neutral
106	p.Val106Gln	0.74	-0.43	Neutral	1.25	0.32	Indeterminate	0.99	-0.01	Neutral
106	p.Val106Pro	1.70	0.76	Indeterminate	1.14	0.19	Indeterminate	1.42	0.51	Indeterminate
106	p.Val106Leu	1.21	0.27	Indeterminate	1.05	0.07	Neutral	1.13	0.17	Neutral
106	p.Val106Asp	0.98	-0.03	Neutral	1.21	0.28	Indeterminate	1.10	0.13	Neutral
106	p.Val106Glu	1.58	0.66	Indeterminate	1.07	0.10	Neutral	1.33	0.41	Indeterminate
106	p.Val106Ala	1.47	0.55	Indeterminate	1.14	0.19	Indeterminate	1.30	0.38	Indeterminate
106	p.Val106Gly	1.55	0.63	Indeterminate	0.95	-0.08	Neutral	1.25	0.32	Indeterminate
106	p.Val106Val	1.00	0.00	Neutral	1.00	0.00	Neutral	1.00	0.00	Neutral

106	p.Val106Tyr	0.87	-0.20	Neutral	1.15	0.20	Indeterminate	1.01	0.01	Neutral
106	p.Val106Cys	1.26	0.33	Indeterminate	1.01	0.02	Neutral	1.14	0.18	Neutral
106	p.Val106Trp	1.07	0.09	Neutral	0.92	-0.11	Neutral	1.00	-0.01	Neutral
106	p.Val106Phe	0.97	-0.05	Neutral	1.19	0.25	Indeterminate	1.08	0.11	Neutral
107	p.Arg107Asn	0.93	-0.10	Neutral	1.13	0.18	Indeterminate	1.03	0.05	Neutral
107	p.Arg107Lys	0.89	-0.17	Neutral	0.93	-0.11	Neutral	0.91	-0.14	Neutral
107	p.Arg107Thr	0.91	-0.14	Neutral	0.76	-0.39	Neutral	0.84	-0.26	Neutral
107	p.Arg107Arg	1.00	0.00	Neutral	1.00	0.00	Neutral	1.00	0.00	Neutral
107	p.Arg107Ser	1.12	0.16	Neutral	1.10	0.14	Neutral	1.11	0.15	Neutral
107	p.Arg107Ile	0.91	-0.14	Neutral	0.72	-0.47	Neutral	0.82	-0.29	Neutral
107	p.Arg107Met	0.90	-0.16	Neutral	0.92	-0.12	Neutral	0.91	-0.14	Neutral
107	p.Arg107His	0.88	-0.19	Neutral	0.99	-0.02	Neutral	0.93	-0.10	Neutral
107	p.Arg107Gln	0.88	-0.18	Neutral	0.81	-0.31	Neutral	0.84	-0.25	Neutral
107	p.Arg107Pro	0.90	-0.15	Neutral	0.85	-0.23	Neutral	0.88	-0.19	Neutral
107	p.Arg107Leu	0.95	-0.08	Neutral	1.20	0.26	Indeterminate	1.07	0.10	Neutral
107	p.Arg107Asp	0.92	-0.12	Neutral	0.94	-0.08	Neutral	0.93	-0.10	Neutral
107	p.Arg107Glu	1.02	0.02	Neutral	1.07	0.10	Neutral	1.04	0.06	Neutral
107	p.Arg107Ala	0.89	-0.18	Neutral	0.86	-0.22	Neutral	0.87	-0.20	Neutral
107	p.Arg107Gly	0.92	-0.12	Neutral	1.04	0.06	Neutral	0.98	-0.03	Neutral
107	p.Arg107Val	0.84	-0.25	Neutral	1.00	-0.01	Neutral	0.92	-0.12	Neutral
107	p.Arg107Tyr	1.01	0.01	Neutral	0.91	-0.14	Neutral	0.96	-0.06	Neutral
107	p.Arg107Cys	0.79	-0.34	Neutral	1.27	0.34	Indeterminate	1.03	0.04	Neutral
107	p.Arg107Trp	0.88	-0.18	Neutral	0.82	-0.29	Neutral	0.85	-0.24	Neutral
107	p.Arg107Phe	0.96	-0.06	Neutral	0.99	-0.01	Neutral	0.97	-0.04	Neutral
108	p.Asp108Asn	0.97	-0.05	Neutral				0.97	-0.05	Neutral
108	p.Asp108Lys	5.96	2.58	Deleterious				5.96	2.58	Deleterious
108	p.Asp108Thr	2.49	1.32	Deleterious				2.49	1.32	Deleterious
108	p.Asp108Arg	6.55	2.71	Deleterious				6.55	2.71	Deleterious
108	p.Asp108Ser	4.10	2.04	Deleterious				4.10	2.04	Deleterious
108	p.Asp108Ile	5.55	2.47	Deleterious				5.55	2.47	Deleterious
108	p.Asp108Met	5.47	2.45	Deleterious				5.47	2.45	Deleterious
108	p.Asp108His	5.94	2.57	Deleterious				5.94	2.57	Deleterious
108	p.Asp108Gln	5.45	2.45	Deleterious				5.45	2.45	Deleterious
108	p.Asp108Pro	6.20	2.63	Deleterious				6.20	2.63	Deleterious
108	p.Asp108Leu	5.82	2.54	Deleterious				5.82	2.54	Deleterious
108	p.Asp108Asp	1.00	0.00	Neutral				1.00	0.00	Neutral
108	p.Asp108Glu	1.43	0.51	Indeterminate				1.43	0.51	Indeterminate
108	p.Asp108Ala	6.13	2.62	Deleterious				6.13	2.62	Deleterious
108	p.Asp108Gly	6.11	2.61	Deleterious				6.11	2.61	Deleterious
108	p.Asp108Val	5.68	2.50	Deleterious				5.68	2.50	Deleterious
108	p.Asp108Tyr	5.94	2.57	Deleterious				5.94	2.57	Deleterious
108	p.Asp108Cys	3.15	1.65	Deleterious				3.15	1.65	Deleterious
108	p.Asp108Trp	6.04	2.60	Deleterious				6.04	2.60	Deleterious
108	p.Asp108Phe	5.57	2.48	Deleterious				5.57	2.48	Deleterious
109	p.Ala109Asn	0.89	-0.16	Neutral	0.61	-0.71	Neutral	0.75	-0.41	Neutral
109	p.Ala109Lys	1.15	0.20	Neutral	1.20	0.26	Indeterminate	1.18	0.23	Neutral
109	p.Ala109Thr	1.03	0.05	Neutral	0.58	-0.78	Neutral	0.81	-0.31	Neutral
109	p.Ala109Arg	0.87	-0.20	Neutral	1.24	0.31	Indeterminate	1.05	0.08	Neutral
109	p.Ala109Ser	0.78	-0.36	Neutral	0.88	-0.19	Neutral	0.83	-0.27	Neutral
109	p.Ala109Ile	0.59	-0.75	Neutral	0.43	-1.23	Neutral	0.51	-0.97	Neutral
109	p.Ala109Met	0.69	-0.54	Neutral	0.71	-0.50	Neutral	0.70	-0.52	Neutral
109	p.Ala109His	0.77	-0.38	Neutral	0.75	-0.41	Neutral	0.76	-0.39	Neutral
109	p.Ala109Gln	0.96	-0.06	Neutral	0.78	-0.37	Neutral	0.87	-0.20	Neutral
109	p.Ala109Pro	13.94	3.80	Deleterious	4.34	2.12	Deleterious	9.14	3.19	Deleterious
109	p.Ala109Leu	0.52	-0.94	Neutral	1.32	0.40	Indeterminate	0.92	-0.12	Neutral
109	p.Ala109Asp	0.98	-0.03	Neutral	0.93	-0.10	Neutral	0.96	-0.07	Neutral
109	p.Ala109Glu	1.38	0.47	Indeterminate	0.98	-0.04	Neutral	1.18	0.24	Neutral
109	p.Ala109Ala	1.00	0.00	Neutral	1.00	0.00	Neutral	1.00	0.00	Neutral
109	p.Ala109Gly	0.77	-0.38	Neutral	0.76	-0.40	Neutral	0.76	-0.39	Neutral
109	p.Ala109Val	0.88	-0.19	Neutral	0.88	-0.18	Neutral	0.88	-0.18	Neutral
109	p.Ala109Tyr	0.79	-0.33	Neutral	0.78	-0.35	Neutral	0.79	-0.34	Neutral
109	p.Ala109Cys	0.84	-0.25	Neutral	0.65	-0.62	Neutral	0.75	-0.42	Neutral
109	p.Ala109Trp	1.03	0.05	Neutral	0.83	-0.27	Neutral	0.93	-0.11	Neutral
109	p.Ala109Phe	1.22	0.29	Indeterminate	0.77	-0.37	Neutral	1.00	0.00	Neutral
110	p.Trp110Asn	0.96	-0.06	Neutral				0.96	-0.06	Neutral
110	p.Trp110Lys	1.13	0.17	Neutral				1.13	0.17	Neutral
110	p.Trp110Thr	0.83	-0.26	Neutral				0.83	-0.26	Neutral
110	p.Trp110Arg	0.88	-0.19	Neutral				0.88	-0.19	Neutral
110	p.Trp110Ser	1.15	0.20	Neutral				1.15	0.20	Neutral
110	p.Trp110Ile	0.97	-0.04	Neutral				0.97	-0.04	Neutral
110	p.Trp110Met	1.01	0.01	Neutral				1.01	0.01	Neutral
110	p.Trp110His	1.06	0.08	Neutral				1.06	0.08	Neutral
110	p.Trp110Gln	1.09	0.12	Neutral				1.09	0.12	Neutral
110	p.Trp110Pro	1.25	0.32	Indeterminate				1.25	0.32	Indeterminate
110	p.Trp110Leu	0.83	-0.28	Neutral				0.83	-0.28	Neutral
110	p.Trp110Asp	0.73	-0.46	Neutral				0.73	-0.46	Neutral
110	p.Trp110Glu	1.16	0.21	Neutral				1.16	0.21	Neutral
110	p.Trp110Ala	1.21	0.28	Indeterminate				1.21	0.28	Indeterminate
110	p.Trp110Gly	0.87	-0.20	Neutral				0.87	-0.20	Neutral
110	p.Trp110Val	0.90	-0.15	Neutral				0.90	-0.15	Neutral
110	p.Trp110Tyr	0.91	-0.13	Neutral				0.91	-0.13	Neutral
110	p.Trp110Cys	0.88	-0.19	Neutral				0.88	-0.19	Neutral
110	p.Trp110Trp	1.00	0.00	Neutral				1.00	0.00	Neutral
110	p.Trp110Phe	0.77	-0.37	Neutral				0.77	-0.37	Neutral
111	p.Gly111Asn	0.87	-0.19	Neutral				0.87	-0.19	Neutral
111	p.Gly111Lys	1.36	0.44	Indeterminate				1.36	0.44	Indeterminate
111	p.Gly111Thr	2.41	1.27	Deleterious				2.41	1.27	Deleterious
111	p.Gly111Arg	1.10	0.13	Neutral				1.10	0.13	Neutral
111	p.Gly111Ser	0.90	-0.16	Neutral				0.90	-0.16	Neutral
111	p.Gly111Ile	4.46	2.16	Deleterious				4.46	2.16	Deleterious
111	p.Gly111Met	0.97	-0.05	Neutral				0.97	-0.05	Neutral
111	p.Gly111His	1.54	0.62	Indeterminate				1.54	0.62	Indeterminate
111	p.Gly111Gln	0.75	-0.41	Neutral				0.75	-0.41	Neutral
111	p.Gly111Pro	25.69	4.68	Deleterious				25.69	4.68	Deleterious
111	p.Gly111Leu	0.94	-0.09	Neutral				0.94	-0.09	Neutral
111	p.Gly111Asp	0.86	-0.22	Neutral				0.86	-0.22	Neutral
111	p.Gly111Glu	1.23	0.29	Indeterminate				1.23	0.29	Indeterminate
111	p.Gly111Ala	1.16	0.21	Neutral				1.16	0.21	Neutral
111	p.Gly111Gly	1.00	0.00	Neutral				1.00	0.00	Neutral
111	p.Gly111Val	3.78	1.92	Deleterious				3.78	1.92	Deleterious
111	p.Gly111Tyr	1.02	0.03	Neutral				1.02	0.03	Neutral

111	p.Gly111Cys	1.12	0.17	Neutral				1.12	0.17	Neutral
111	p.Gly111Trp	1.04	0.06	Neutral				1.04	0.06	Neutral
111	p.Gly111Phe	0.95	-0.07	Neutral				0.95	-0.07	Neutral
112	p.Arg112Asn	0.92	-0.12	Neutral				0.92	-0.12	Neutral
112	p.Arg112Lys	1.20	0.27	Indeterminate				1.20	0.27	Indeterminate
112	p.Arg112Thr	0.90	-0.16	Neutral				0.90	-0.16	Neutral
112	p.Arg112Arg	1.00	0.00	Neutral				1.00	0.00	Neutral
112	p.Arg112Ser	0.97	-0.05	Neutral				0.97	-0.05	Neutral
112	p.Arg112Ile	1.16	0.21	Neutral				1.16	0.21	Neutral
112	p.Arg112Met	0.66	-0.61	Neutral				0.66	-0.61	Neutral
112	p.Arg112His	1.17	0.22	Neutral				1.17	0.22	Neutral
112	p.Arg112Gln	1.29	0.37	Indeterminate				1.29	0.37	Indeterminate
112	p.Arg112Pro	40.46	5.34	Deleterious				40.46	5.34	Deleterious
112	p.Arg112Leu	1.87	0.90	Indeterminate				1.87	0.90	Indeterminate
112	p.Arg112Asp	1.48	0.56	Indeterminate				1.48	0.56	Indeterminate
112	p.Arg112Glu	1.80	0.85	Indeterminate				1.80	0.85	Indeterminate
112	p.Arg112Ala	1.35	0.43	Indeterminate				1.35	0.43	Indeterminate
112	p.Arg112Gly	2.33	1.22	Deleterious				2.33	1.22	Deleterious
112	p.Arg112Val	1.41	0.49	Indeterminate				1.41	0.49	Indeterminate
112	p.Arg112Tyr	1.16	0.22	Neutral				1.16	0.22	Neutral
112	p.Arg112Cys	1.60	0.68	Indeterminate				1.60	0.68	Indeterminate
112	p.Arg112Trp	1.15	0.20	Neutral				1.15	0.20	Neutral
112	p.Arg112Phe	1.42	0.51	Indeterminate				1.42	0.51	Indeterminate
113	p.Leu113Asn	1.12	0.16	Neutral	1.17	0.22	Indeterminate	1.14	0.19	Neutral
113	p.Leu113Lys	1.07	0.10	Neutral	0.79	-0.35	Neutral	0.93	-0.11	Neutral
113	p.Leu113Thr	1.25	0.33	Indeterminate	0.96	-0.06	Neutral	1.10	0.14	Neutral
113	p.Leu113Arg	1.23	0.30	Indeterminate	0.81	-0.31	Neutral	1.02	0.03	Neutral
113	p.Leu113Ser	1.09	0.12	Neutral	0.76	-0.39	Neutral	0.92	-0.11	Neutral
113	p.Leu113Ile	1.16	0.21	Neutral	0.66	-0.59	Neutral	0.91	-0.14	Neutral
113	p.Leu113Met	0.81	-0.30	Neutral	1.33	0.41	Indeterminate	1.07	0.10	Neutral
113	p.Leu113His	1.32	0.40	Indeterminate	1.21	0.27	Indeterminate	1.27	0.34	Indeterminate
113	p.Leu113Gln	1.06	0.09	Neutral	0.31	-1.70	Neutral	0.68	-0.55	Neutral
113	p.Leu113Pro	1.56	0.64	Indeterminate	1.38	0.47	Indeterminate	1.47	0.56	Indeterminate
113	p.Leu113Leu	1.00	0.00	Neutral	1.00	0.00	Neutral	1.00	0.00	Neutral
113	p.Leu113Asp	1.17	0.23	Neutral	0.97	-0.05	Neutral	1.07	0.10	Neutral
113	p.Leu113Glu	0.94	-0.09	Neutral	1.02	0.03	Neutral	0.98	-0.03	Neutral
113	p.Leu113Ala	1.10	0.14	Neutral	1.64	0.71	Indeterminate	1.37	0.46	Indeterminate
113	p.Leu113Gly	1.15	0.20	Neutral	1.35	0.43	Indeterminate	1.25	0.32	Indeterminate
113	p.Leu113Val	0.97	-0.04	Neutral	0.84	-0.25	Neutral	0.90	-0.15	Neutral
113	p.Leu113Tyr	1.03	0.04	Neutral	0.68	-0.56	Neutral	0.85	-0.23	Neutral
113	p.Leu113Cys	0.98	-0.03	Neutral	0.93	-0.10	Neutral	0.96	-0.06	Neutral
113	p.Leu113Trp	1.01	0.01	Neutral	1.19	0.25	Indeterminate	1.10	0.13	Neutral
113	p.Leu113Phe	1.23	0.30	Indeterminate	1.26	0.33	Indeterminate	1.24	0.31	Indeterminate
114	p.Pro114Asn	4.52	2.18	Deleterious				4.52	2.18	Deleterious
114	p.Pro114Lys	5.61	2.49	Deleterious				5.61	2.49	Deleterious
114	p.Pro114Thr	3.24	1.70	Deleterious				3.24	1.70	Deleterious
114	p.Pro114Arg	4.59	2.20	Deleterious				4.59	2.20	Deleterious
114	p.Pro114Ser	1.70	0.76	Indeterminate				1.70	0.76	Indeterminate
114	p.Pro114Ile	5.11	2.35	Deleterious				5.11	2.35	Deleterious
114	p.Pro114Met	5.18	2.37	Deleterious				5.18	2.37	Deleterious
114	p.Pro114His	5.87	2.55	Deleterious				5.87	2.55	Deleterious
114	p.Pro114Gln	5.24	2.39	Deleterious				5.24	2.39	Deleterious
114	p.Pro114Pro	1.00	0.00	Neutral				1.00	0.00	Neutral
114	p.Pro114Leu	5.02	2.33	Deleterious				5.02	2.33	Deleterious
114	p.Pro114Asp	5.01	2.32	Deleterious				5.01	2.32	Deleterious
114	p.Pro114Glu	4.76	2.25	Deleterious				4.76	2.25	Deleterious
114	p.Pro114Ala	1.11	0.15	Neutral				1.11	0.15	Neutral
114	p.Pro114Gly	3.25	1.70	Deleterious				3.25	1.70	Deleterious
114	p.Pro114Val	3.00	1.59	Deleterious				3.00	1.59	Deleterious
114	p.Pro114Tyr	5.53	2.47	Deleterious				5.53	2.47	Deleterious
114	p.Pro114Cys	1.60	0.68	Indeterminate				1.60	0.68	Indeterminate
114	p.Pro114Trp	4.96	2.31	Deleterious				4.96	2.31	Deleterious
114	p.Pro114Phe	4.35	2.12	Deleterious				4.35	2.12	Deleterious
115	p.Val115Asn	1.01	0.02	Neutral				1.01	0.02	Neutral
115	p.Val115Lys	1.02	0.03	Neutral				1.02	0.03	Neutral
115	p.Val115Thr	0.96	-0.05	Neutral				0.96	-0.05	Neutral
115	p.Val115Arg	1.48	0.56	Indeterminate				1.48	0.56	Indeterminate
115	p.Val115Ser	1.12	0.17	Neutral				1.12	0.17	Neutral
115	p.Val115Ile	0.93	-0.10	Neutral				0.93	-0.10	Neutral
115	p.Val115Met	0.98	-0.03	Neutral				0.98	-0.03	Neutral
115	p.Val115His	1.17	0.22	Neutral				1.17	0.22	Neutral
115	p.Val115Gln	1.49	0.58	Indeterminate				1.49	0.58	Indeterminate
115	p.Val115Pro	2.46	1.30	Deleterious				2.46	1.30	Deleterious
115	p.Val115Leu	1.20	0.27	Indeterminate				1.20	0.27	Indeterminate
115	p.Val115Asp	1.30	0.38	Indeterminate				1.30	0.38	Indeterminate
115	p.Val115Glu	1.20	0.27	Indeterminate				1.20	0.27	Indeterminate
115	p.Val115Ala	1.45	0.53	Indeterminate				1.45	0.53	Indeterminate
115	p.Val115Gly	1.21	0.28	Indeterminate				1.21	0.28	Indeterminate
115	p.Val115Val	1.00	0.00	Neutral				1.00	0.00	Neutral
115	p.Val115Tyr	0.96	-0.06	Neutral				0.96	-0.06	Neutral
115	p.Val115Cys	0.94	-0.09	Neutral				0.94	-0.09	Neutral
115	p.Val115Trp	1.07	0.10	Neutral				1.07	0.10	Neutral
115	p.Val115Phe	1.11	0.15	Neutral				1.11	0.15	Neutral
116	p.Asp116Asn	0.98	-0.03	Neutral				0.98	-0.03	Neutral
116	p.Asp116Lys	0.99	-0.01	Neutral				0.99	-0.01	Neutral
116	p.Asp116Thr	1.18	0.24	Neutral				1.18	0.24	Neutral
116	p.Asp116Arg	1.18	0.23	Neutral				1.18	0.23	Neutral
116	p.Asp116Ser	1.04	0.06	Neutral				1.04	0.06	Neutral
116	p.Asp116Ile	1.19	0.25	Indeterminate				1.19	0.25	Indeterminate
116	p.Asp116Met	0.82	-0.29	Neutral				0.82	-0.29	Neutral
116	p.Asp116His	1.05	0.07	Neutral				1.05	0.07	Neutral
116	p.Asp116Gln	1.04	0.06	Neutral				1.04	0.06	Neutral
116	p.Asp116Pro	4.74	2.25	Deleterious				4.74	2.25	Deleterious
116	p.Asp116Leu	1.09	0.13	Neutral				1.09	0.13	Neutral
116	p.Asp116Asp	1.00	0.00	Neutral				1.00	0.00	Neutral
116	p.Asp116Glu	1.10	0.14	Neutral				1.10	0.14	Neutral
116	p.Asp116Ala	1.01	0.01	Neutral				1.01	0.01	Neutral
116	p.Asp116Gly	1.47	0.56	Indeterminate				1.47	0.56	Indeterminate
116	p.Asp116Val	1.12	0.17	Neutral				1.12	0.17	Neutral
116	p.Asp116Tyr	0.91	-0.14	Neutral				0.91	-0.14	Neutral
116	p.Asp116Cys	1.07	0.10	Neutral				1.07	0.10	Neutral

116	p.Asp116Trp	1.36	0.45	Indeterminate				1.36	0.45	Indeterminate
116	p.Asp116Phe	1.17	0.23	Neutral				1.17	0.23	Neutral
117	p.Leu117Asn	1.03	0.04	Neutral				1.03	0.04	Neutral
117	p.Leu117Lys	1.05	0.07	Neutral				1.05	0.07	Neutral
117	p.Leu117Thr	0.96	-0.07	Neutral				0.96	-0.07	Neutral
117	p.Leu117Arg	1.09	0.13	Neutral				1.09	0.13	Neutral
117	p.Leu117Ser	1.06	0.08	Neutral				1.06	0.08	Neutral
117	p.Leu117Ile	1.00	0.01	Neutral				1.00	0.01	Neutral
117	p.Leu117Met	1.02	0.03	Neutral				1.02	0.03	Neutral
117	p.Leu117His	0.84	-0.25	Neutral				0.84	-0.25	Neutral
117	p.Leu117Gln	1.18	0.24	Indeterminate				1.18	0.24	Indeterminate
117	p.Leu117Pro	2.26	1.18	Deleterious				2.26	1.18	Deleterious
117	p.Leu117Leu	1.00	0.00	Neutral				1.00	0.00	Neutral
117	p.Leu117Asp	0.97	-0.04	Neutral				0.97	-0.04	Neutral
117	p.Leu117Glu	1.10	0.14	Neutral				1.10	0.14	Neutral
117	p.Leu117Ala	0.95	-0.08	Neutral				0.95	-0.08	Neutral
117	p.Leu117Gly	1.07	0.10	Neutral				1.07	0.10	Neutral
117	p.Leu117Val	1.03	0.05	Neutral				1.03	0.05	Neutral
117	p.Leu117Tyr	0.94	-0.09	Neutral				0.94	-0.09	Neutral
117	p.Leu117Cys	1.10	0.14	Neutral				1.10	0.14	Neutral
117	p.Leu117Trp	0.96	-0.06	Neutral				0.96	-0.06	Neutral
117	p.Leu117Phe	1.05	0.07	Neutral				1.05	0.07	Neutral
118	p.Ala118Asn	2.04	1.03	Indeterminate				2.04	1.03	Indeterminate
118	p.Ala118Lys	2.34	1.23	Deleterious				2.34	1.23	Deleterious
118	p.Ala118Thr	1.28	0.36	Indeterminate				1.28	0.36	Indeterminate
118	p.Ala118Arg	2.23	1.15	Deleterious				2.23	1.15	Deleterious
118	p.Ala118Ser	1.18	0.24	Indeterminate				1.18	0.24	Indeterminate
118	p.Ala118Ile	1.60	0.67	Indeterminate				1.60	0.67	Indeterminate
118	p.Ala118Met	1.74	0.80	Indeterminate				1.74	0.80	Indeterminate
118	p.Ala118His	2.19	1.13	Deleterious				2.19	1.13	Deleterious
118	p.Ala118Gln	2.01	1.01	Indeterminate				2.01	1.01	Indeterminate
118	p.Ala118Pro	1.63	0.71	Indeterminate				1.63	0.71	Indeterminate
118	p.Ala118Leu	1.46	0.55	Indeterminate				1.46	0.55	Indeterminate
118	p.Ala118Asp	2.15	1.10	Deleterious				2.15	1.10	Deleterious
118	p.Ala118Glu	2.11	1.08	Indeterminate				2.11	1.08	Indeterminate
118	p.Ala118Ala	1.00	0.00	Neutral				1.00	0.00	Neutral
118	p.Ala118Gly	0.98	-0.02	Neutral				0.98	-0.02	Neutral
118	p.Ala118Val	1.29	0.37	Indeterminate				1.29	0.37	Indeterminate
118	p.Ala118Tyr	2.02	1.02	Indeterminate				2.02	1.02	Indeterminate
118	p.Ala118Cys	1.11	0.15	Neutral				1.11	0.15	Neutral
118	p.Ala118Trp	2.40	1.26	Deleterious				2.40	1.26	Deleterious
118	p.Ala118Phe	2.05	1.04	Indeterminate				2.05	1.04	Indeterminate
119	p.Glu119Asn	0.89	-0.17	Neutral				0.89	-0.17	Neutral
119	p.Glu119Lys	0.74	-0.44	Neutral				0.74	-0.44	Neutral
119	p.Glu119Thr	0.71	-0.48	Neutral				0.71	-0.48	Neutral
119	p.Glu119Arg	1.28	0.36	Indeterminate				1.28	0.36	Indeterminate
119	p.Glu119Ser	0.40	-1.31	Neutral				0.40	-1.31	Neutral
119	p.Glu119Ile	0.63	-0.67	Neutral				0.63	-0.67	Neutral
119	p.Glu119Met	1.29	0.37	Indeterminate				1.29	0.37	Indeterminate
119	p.Glu119His	2.07	1.05	Indeterminate				2.07	1.05	Indeterminate
119	p.Glu119Gln	1.79	0.84	Indeterminate				1.79	0.84	Indeterminate
119	p.Glu119Pro	2.25	1.17	Deleterious				2.25	1.17	Deleterious
119	p.Glu119Leu	0.83	-0.27	Neutral				0.83	-0.27	Neutral
119	p.Glu119Asp	1.38	0.46	Indeterminate				1.38	0.46	Indeterminate
119	p.Glu119Glu	1.00	0.00	Neutral				1.00	0.00	Neutral
119	p.Glu119Ala	0.53	-0.90	Neutral				0.53	-0.90	Neutral
119	p.Glu119Gly	0.89	-0.16	Neutral				0.89	-0.16	Neutral
119	p.Glu119Val	1.27	0.35	Indeterminate				1.27	0.35	Indeterminate
119	p.Glu119Tyr	0.42	-1.26	Neutral				0.42	-1.26	Neutral
119	p.Glu119Cys	0.49	-1.04	Neutral				0.49	-1.04	Neutral
119	p.Glu119Trp	2.09	1.06	Indeterminate				2.09	1.06	Indeterminate
119	p.Glu119Phe	2.16	1.11	Deleterious				2.16	1.11	Deleterious
120	p.Glu120Asn	1.20	0.26	Indeterminate	1.02	0.03	Neutral	1.11	0.15	Neutral
120	p.Glu120Lys	1.07	0.09	Neutral	1.01	0.02	Neutral	1.04	0.06	Neutral
120	p.Glu120Thr	1.11	0.15	Neutral	0.76	-0.40	Neutral	0.93	-0.10	Neutral
120	p.Glu120Arg	1.11	0.15	Neutral	1.01	0.01	Neutral	1.06	0.08	Neutral
120	p.Glu120Ser	1.05	0.07	Neutral	0.81	-0.30	Neutral	0.93	-0.10	Neutral
120	p.Glu120Ile	1.36	0.44	Indeterminate	0.91	-0.14	Neutral	1.13	0.18	Neutral
120	p.Glu120Met	1.43	0.52	Indeterminate	1.02	0.03	Neutral	1.23	0.29	Indeterminate
120	p.Glu120His	1.10	0.14	Neutral	1.06	0.08	Neutral	1.08	0.11	Neutral
120	p.Glu120Gln	1.24	0.31	Indeterminate	1.00	0.00	Neutral	1.12	0.17	Neutral
120	p.Glu120Pro	1.20	0.27	Indeterminate	0.87	-0.20	Neutral	1.04	0.05	Neutral
120	p.Glu120Leu	0.92	-0.11	Neutral	0.95	-0.08	Neutral	0.94	-0.10	Neutral
120	p.Glu120Asp	1.13	0.17	Neutral	1.05	0.07	Neutral	1.09	0.12	Neutral
120	p.Glu120Glu	1.00	0.00	Neutral	1.00	0.00	Neutral	1.00	0.00	Neutral
120	p.Glu120Ala	1.03	0.04	Neutral	0.65	-0.62	Neutral	0.84	-0.25	Neutral
120	p.Glu120Gly	1.08	0.11	Neutral	0.76	-0.40	Neutral	0.92	-0.13	Neutral
120	p.Glu120Val	1.11	0.16	Neutral	0.96	-0.06	Neutral	1.04	0.05	Neutral
120	p.Glu120Tyr	1.05	0.07	Neutral	1.08	0.11	Neutral	1.06	0.09	Neutral
120	p.Glu120Cys	1.16	0.21	Neutral	0.99	-0.02	Neutral	1.07	0.10	Neutral
120	p.Glu120Trp	1.24	0.31	Indeterminate	0.86	-0.23	Neutral	1.05	0.07	Neutral
120	p.Glu120Phe	1.03	0.04	Neutral	0.85	-0.24	Neutral	0.94	-0.10	Neutral
121	p.Leu121Asn	1.30	0.38	Indeterminate				1.30	0.38	Indeterminate
121	p.Leu121Lys	1.12	0.16	Neutral				1.12	0.16	Neutral
121	p.Leu121Thr	1.18	0.24	Indeterminate				1.18	0.24	Indeterminate
121	p.Leu121Arg	1.76	0.81	Indeterminate				1.76	0.81	Indeterminate
121	p.Leu121Ser	1.14	0.19	Neutral				1.14	0.19	Neutral
121	p.Leu121Ile	1.13	0.18	Neutral				1.13	0.18	Neutral
121	p.Leu121Met	4.46	2.16	Deleterious				4.46	2.16	Deleterious
121	p.Leu121His	0.92	-0.12	Neutral				0.92	-0.12	Neutral
121	p.Leu121Gln	1.09	0.12	Neutral				1.09	0.12	Neutral
121	p.Leu121Pro	0.96	-0.05	Neutral				0.96	-0.05	Neutral
121	p.Leu121Leu	1.00	0.00	Neutral				1.00	0.00	Neutral
121	p.Leu121Asp	0.65	-0.62	Neutral				0.65	-0.62	Neutral
121	p.Leu121Glu	1.42	0.50	Indeterminate				1.42	0.50	Indeterminate
121	p.Leu121Ala	1.14	0.19	Neutral				1.14	0.19	Neutral
121	p.Leu121Gly	1.36	0.45	Indeterminate				1.36	0.45	Indeterminate
121	p.Leu121Val	0.84	-0.25	Neutral				0.84	-0.25	Neutral
121	p.Leu121Tyr	0.74	-0.43	Neutral				0.74	-0.43	Neutral
121	p.Leu121Cys	0.91	-0.13	Neutral				0.91	-0.13	Neutral
121	p.Leu121Trp	1.32	0.40	Indeterminate				1.32	0.40	Indeterminate

121	p.Leu121Phe	0.99	-0.02	Neutral				0.99	-0.02	Neutral
122	p.Gly122Asn	0.93	-0.11	Neutral	0.50	-0.99	Neutral	0.71	-0.48	Neutral
122	p.Gly122Lys	1.13	0.18	Neutral	0.59	-0.76	Neutral	0.86	-0.21	Neutral
122	p.Gly122Thr	1.04	0.06	Neutral	0.78	-0.35	Neutral	0.91	-0.13	Neutral
122	p.Gly122Arg	1.30	0.38	Indeterminate	1.17	0.23	Indeterminate	1.24	0.31	Indeterminate
122	p.Gly122Ser	0.94	-0.10	Neutral	0.78	-0.36	Neutral	0.86	-0.22	Neutral
122	p.Gly122Ile	1.30	0.37	Indeterminate	0.68	-0.56	Neutral	0.99	-0.02	Neutral
122	p.Gly122Met	0.97	-0.05	Neutral	0.99	-0.02	Neutral	0.98	-0.03	Neutral
122	p.Gly122His	0.95	-0.08	Neutral	0.88	-0.19	Neutral	0.91	-0.13	Neutral
122	p.Gly122Gln	1.03	0.04	Neutral	0.63	-0.67	Neutral	0.83	-0.27	Neutral
122	p.Gly122Pro	0.99	-0.01	Neutral	1.24	0.31	Indeterminate	1.12	0.16	Neutral
122	p.Gly122Leu	1.10	0.14	Neutral	0.65	-0.61	Neutral	0.88	-0.19	Neutral
122	p.Gly122Asp	1.08	0.12	Neutral	0.63	-0.67	Neutral	0.86	-0.23	Neutral
122	p.Gly122Glu	1.17	0.23	Neutral	0.65	-0.61	Neutral	0.91	-0.13	Neutral
122	p.Gly122Ala	1.10	0.14	Neutral	0.74	-0.44	Neutral	0.92	-0.12	Neutral
122	p.Gly122Gly	1.00	0.00	Neutral	1.00	0.00	Neutral	1.00	0.00	Neutral
122	p.Gly122Val	0.88	-0.19	Neutral	0.92	-0.13	Neutral	0.90	-0.16	Neutral
122	p.Gly122Tyr	1.06	0.08	Neutral	1.13	0.18	Indeterminate	1.09	0.13	Neutral
122	p.Gly122Cys	1.33	0.41	Indeterminate	0.96	-0.06	Neutral	1.15	0.20	Neutral
122	p.Gly122Trp	0.85	-0.23	Neutral	1.41	0.49	Indeterminate	1.13	0.18	Neutral
122	p.Gly122Phe	1.29	0.37	Indeterminate	1.03	0.04	Neutral	1.16	0.22	Neutral
123	p.His123Asn	1.34	0.43	Indeterminate	1.12	0.16	Indeterminate	1.23	0.30	Indeterminate
123	p.His123Lys	0.84	-0.26	Neutral	0.98	-0.03	Neutral	0.91	-0.14	Neutral
123	p.His123Thr	1.62	0.70	Indeterminate	1.50	0.58	Indeterminate	1.56	0.64	Indeterminate
123	p.His123Arg	1.13	0.17	Neutral	1.06	0.09	Neutral	1.09	0.13	Neutral
123	p.His123Ser	1.20	0.27	Indeterminate	0.95	-0.08	Neutral	1.07	0.10	Neutral
123	p.His123Ile	1.28	0.36	Indeterminate	1.04	0.05	Neutral	1.16	0.21	Neutral
123	p.His123Met	1.54	0.62	Indeterminate	1.32	0.40	Indeterminate	1.43	0.51	Indeterminate
123	p.His123His	1.00	0.00	Neutral	1.00	0.00	Neutral	1.00	0.00	Neutral
123	p.His123Gln	1.06	0.08	Neutral	0.94	-0.09	Neutral	1.00	0.00	Neutral
123	p.His123Pro	3.51	1.81	Deleterious	4.29	2.10	Deleterious	3.90	1.96	Deleterious
123	p.His123Leu	1.28	0.36	Indeterminate	1.30	0.38	Indeterminate	1.29	0.37	Indeterminate
123	p.His123Asp	1.57	0.65	Indeterminate	1.58	0.66	Indeterminate	1.57	0.65	Indeterminate
123	p.His123Glu	1.25	0.33	Indeterminate	1.82	0.86	Indeterminate	1.54	0.62	Indeterminate
123	p.His123Ala	1.09	0.12	Neutral	1.15	0.20	Indeterminate	1.12	0.16	Neutral
123	p.His123Gly	1.84	0.88	Indeterminate	1.59	0.67	Indeterminate	1.72	0.78	Indeterminate
123	p.His123Val	1.53	0.61	Indeterminate	1.98	0.99	Indeterminate	1.76	0.81	Indeterminate
123	p.His123Tyr	1.01	0.01	Neutral	0.81	-0.30	Neutral	0.91	-0.14	Neutral
123	p.His123Cys	1.28	0.36	Indeterminate	0.95	-0.07	Neutral	1.12	0.16	Neutral
123	p.His123Trp	1.04	0.06	Neutral	1.06	0.09	Neutral	1.05	0.07	Neutral
123	p.His123Phe	1.13	0.17	Neutral	1.93	0.95	Indeterminate	1.53	0.61	Indeterminate
124	p.Arg124Asn	0.79	-0.34	Neutral	0.64	-0.64	Neutral	0.71	-0.48	Neutral
124	p.Arg124Lys	0.91	-0.14	Neutral	0.89	-0.16	Neutral	0.90	-0.15	Neutral
124	p.Arg124Thr	0.87	-0.20	Neutral	1.31	0.39	Indeterminate	1.09	0.13	Neutral
124	p.Arg124Arg	1.00	0.00	Neutral	1.00	0.00	Neutral	1.00	0.00	Neutral
124	p.Arg124Ser	0.82	-0.29	Neutral	0.59	-0.77	Neutral	0.70	-0.51	Neutral
124	p.Arg124Ile	1.14	0.19	Neutral	2.49	1.31	Indeterminate	1.81	0.86	Indeterminate
124	p.Arg124Met	0.89	-0.17	Neutral	0.31	-1.67	Neutral	0.60	-0.74	Neutral
124	p.Arg124His	0.89	-0.17	Neutral	0.42	-1.25	Neutral	0.66	-0.61	Neutral
124	p.Arg124Gln	0.84	-0.25	Neutral	0.67	-0.57	Neutral	0.76	-0.40	Neutral
124	p.Arg124Pro	0.89	-0.18	Neutral	0.58	-0.77	Neutral	0.74	-0.44	Neutral
124	p.Arg124Leu	0.91	-0.14	Neutral	0.82	-0.28	Neutral	0.86	-0.21	Neutral
124	p.Arg124Asp	0.78	-0.37	Neutral	0.49	-1.02	Neutral	0.63	-0.66	Neutral
124	p.Arg124Glu	0.81	-0.31	Neutral	0.93	-0.10	Neutral	0.87	-0.20	Neutral
124	p.Arg124Ala	0.86	-0.22	Neutral	0.57	-0.82	Neutral	0.71	-0.49	Neutral
124	p.Arg124Gly	1.25	0.32	Indeterminate	1.07	0.10	Neutral	1.16	0.22	Neutral
124	p.Arg124Val	0.91	-0.14	Neutral	1.08	0.12	Neutral	1.00	0.00	Neutral
124	p.Arg124Tyr	0.84	-0.26	Neutral	1.76	0.82	Indeterminate	1.30	0.38	Indeterminate
124	p.Arg124Cys	1.00	0.00	Neutral	0.45	-1.14	Neutral	0.73	-0.46	Neutral
124	p.Arg124Trp	0.92	-0.12	Neutral	0.62	-0.69	Neutral	0.77	-0.38	Neutral
124	p.Arg124Phe	0.79	-0.35	Neutral	0.86	-0.22	Neutral	0.82	-0.28	Neutral
125	p.Asp125Asn	1.10	0.14	Neutral	0.80	-0.32	Neutral	0.95	-0.07	Neutral
125	p.Asp125Lys	0.90	-0.15	Neutral	0.75	-0.42	Neutral	0.82	-0.28	Neutral
125	p.Asp125Thr	0.72	-0.47	Neutral	0.81	-0.30	Neutral	0.77	-0.38	Neutral
125	p.Asp125Arg	1.00	0.00	Neutral	0.92	-0.13	Neutral	0.96	-0.06	Neutral
125	p.Asp125Ser	0.72	-0.47	Neutral	0.96	-0.07	Neutral	0.84	-0.25	Neutral
125	p.Asp125Ile	0.71	-0.50	Neutral	0.69	-0.53	Neutral	0.70	-0.52	Neutral
125	p.Asp125Met	0.68	-0.55	Neutral	0.76	-0.40	Neutral	0.72	-0.47	Neutral
125	p.Asp125His	0.88	-0.18	Neutral	0.75	-0.42	Neutral	0.81	-0.30	Neutral
125	p.Asp125Gln	0.70	-0.52	Neutral	0.69	-0.53	Neutral	0.69	-0.53	Neutral
125	p.Asp125Pro	0.72	-0.48	Neutral	0.88	-0.18	Neutral	0.80	-0.32	Neutral
125	p.Asp125Leu	0.80	-0.32	Neutral	0.98	-0.02	Neutral	0.89	-0.16	Neutral
125	p.Asp125Asp	1.00	0.00	Neutral	1.00	0.00	Neutral	1.00	0.00	Neutral
125	p.Asp125Glu	0.70	-0.51	Neutral	0.94	-0.10	Neutral	0.82	-0.29	Neutral
125	p.Asp125Ala	0.72	-0.47	Neutral	1.18	0.24	Indeterminate	0.95	-0.07	Neutral
125	p.Asp125Gly	1.17	0.22	Neutral	0.64	-0.64	Neutral	0.90	-0.15	Neutral
125	p.Asp125Val	0.51	-0.96	Neutral	0.72	-0.46	Neutral	0.62	-0.69	Neutral
125	p.Asp125Tyr	2.39	1.26	Deleterious	2.67	1.42	Indeterminate	2.53	1.34	Deleterious
125	p.Asp125Cys	1.10	0.13	Neutral	0.61	-0.72	Neutral	0.85	-0.23	Neutral
125	p.Asp125Trp	0.88	-0.18	Neutral	0.91	-0.13	Neutral	0.90	-0.15	Neutral
125	p.Asp125Phe	0.75	-0.42	Neutral	1.12	0.16	Indeterminate	0.93	-0.10	Neutral
126	p.Val126Asn	0.98	-0.03	Neutral				0.98	-0.03	Neutral
126	p.Val126Lys	6.93	2.79	Deleterious				6.93	2.79	Deleterious
126	p.Val126Thr	0.41	-1.28	Neutral				0.41	-1.28	Neutral
126	p.Val126Arg	9.89	3.31	Deleterious				9.89	3.31	Deleterious
126	p.Val126Ser	0.64	-0.65	Neutral				0.64	-0.65	Neutral
126	p.Val126Ile	0.66	-0.60	Neutral				0.66	-0.60	Neutral
126	p.Val126Met	0.64	-0.64	Neutral				0.64	-0.64	Neutral
126	p.Val126His	3.14	1.65	Deleterious				3.14	1.65	Deleterious
126	p.Val126Gln	1.21	0.27	Indeterminate				1.21	0.27	Indeterminate
126	p.Val126Pro	0.56	-0.83	Neutral				0.56	-0.83	Neutral
126	p.Val126Leu	0.56	-0.85	Neutral				0.56	-0.85	Neutral
126	p.Val126Asp	6.68	2.74	Deleterious				6.68	2.74	Deleterious
126	p.Val126Glu	0.77	-0.38	Neutral				0.77	-0.38	Neutral
126	p.Val126Ala	0.72	-0.46	Neutral				0.72	-0.46	Neutral
126	p.Val126Gly	0.96	-0.06	Neutral				0.96	-0.06	Neutral
126	p.Val126Val	1.00	0.00	Neutral				1.00	0.00	Neutral
126	p.Val126Tyr	7.32	2.87	Deleterious				7.32	2.87	Deleterious
126	p.Val126Cys	0.45	-1.15	Neutral				0.45	-1.15	Neutral
126	p.Val126Trp	8.66	3.11	Deleterious				8.66	3.11	Deleterious
126	p.Val126Phe	0.97	-0.04	Neutral				0.97	-0.04	Neutral

127	p.Ala127Asn	0.98	-0.03	Neutral	0.98	-0.03	Neutral
127	p.Ala127Lys	1.37	0.45	Indeterminate	1.37	0.45	Indeterminate
127	p.Ala127Thr	2.09	1.06	Indeterminate	2.09	1.06	Indeterminate
127	p.Ala127Arg	2.10	1.07	Indeterminate	2.10	1.07	Indeterminate
127	p.Ala127Ser	1.18	0.24	Neutral	1.18	0.24	Neutral
127	p.Ala127Ile	1.97	0.98	Indeterminate	1.97	0.98	Indeterminate
127	p.Ala127Met	1.06	0.09	Neutral	1.06	0.09	Neutral
127	p.Ala127His	2.13	1.09	Indeterminate	2.13	1.09	Indeterminate
127	p.Ala127Gln	1.23	0.30	Indeterminate	1.23	0.30	Indeterminate
127	p.Ala127Pro	5.10	2.35	Deleterious	5.10	2.35	Deleterious
127	p.Ala127Leu	1.27	0.34	Indeterminate	1.27	0.34	Indeterminate
127	p.Ala127Asp	1.87	0.90	Indeterminate	1.87	0.90	Indeterminate
127	p.Ala127Glu	1.08	0.11	Neutral	1.08	0.11	Neutral
127	p.Ala127Ala	1.00	0.00	Neutral	1.00	0.00	Neutral
127	p.Ala127Gly	1.48	0.56	Indeterminate	1.48	0.56	Indeterminate
127	p.Ala127Val	0.95	-0.07	Neutral	0.95	-0.07	Neutral
127	p.Ala127Tyr	1.38	0.47	Indeterminate	1.38	0.47	Indeterminate
127	p.Ala127Cys	0.80	-0.33	Neutral	0.80	-0.33	Neutral
127	p.Ala127Trp	1.46	0.54	Indeterminate	1.46	0.54	Indeterminate
127	p.Ala127Phe	1.36	0.44	Indeterminate	1.36	0.44	Indeterminate
128	p.Arg128Asn	0.38	-1.40	Neutral	0.38	-1.40	Neutral
128	p.Arg128Lys	0.98	-0.03	Neutral	0.98	-0.03	Neutral
128	p.Arg128Thr	0.73	-0.46	Neutral	0.73	-0.46	Neutral
128	p.Arg128Arg	1.00	0.00	Neutral	1.00	0.00	Neutral
128	p.Arg128Ser	1.43	0.51	Indeterminate	1.43	0.51	Indeterminate
128	p.Arg128Ile	1.28	0.35	Indeterminate	1.28	0.35	Indeterminate
128	p.Arg128Met	0.83	-0.26	Neutral	0.83	-0.26	Neutral
128	p.Arg128His	0.86	-0.22	Neutral	0.86	-0.22	Neutral
128	p.Arg128Gln	0.60	-0.74	Neutral	0.60	-0.74	Neutral
128	p.Arg128Pro	2.66	1.41	Deleterious	2.66	1.41	Deleterious
128	p.Arg128Leu	0.74	-0.43	Neutral	0.74	-0.43	Neutral
128	p.Arg128Asp	0.65	-0.61	Neutral	0.65	-0.61	Neutral
128	p.Arg128Glu	0.88	-0.18	Neutral	0.88	-0.18	Neutral
128	p.Arg128Ala	0.74	-0.44	Neutral	0.74	-0.44	Neutral
128	p.Arg128Gly	1.19	0.26	Indeterminate	1.19	0.26	Indeterminate
128	p.Arg128Val	0.65	-0.63	Neutral	0.65	-0.63	Neutral
128	p.Arg128Tyr	0.78	-0.35	Neutral	0.78	-0.35	Neutral
128	p.Arg128Cys	0.51	-0.97	Neutral	0.51	-0.97	Neutral
128	p.Arg128Trp	0.67	-0.58	Neutral	0.67	-0.58	Neutral
128	p.Arg128Phe	1.20	0.26	Indeterminate	1.20	0.26	Indeterminate
129	p.Tyr129Asn	2.40	1.26	Deleterious	2.40	1.26	Deleterious
129	p.Tyr129Lys	1.31	0.39	Indeterminate	1.31	0.39	Indeterminate
129	p.Tyr129Thr	1.99	0.99	Indeterminate	1.99	0.99	Indeterminate
129	p.Tyr129Arg	1.07	0.10	Neutral	1.07	0.10	Neutral
129	p.Tyr129Ser	1.08	0.11	Neutral	1.08	0.11	Neutral
129	p.Tyr129Ile	1.40	0.49	Indeterminate	1.40	0.49	Indeterminate
129	p.Tyr129Met	0.98	-0.03	Neutral	0.98	-0.03	Neutral
129	p.Tyr129His	1.07	0.10	Neutral	1.07	0.10	Neutral
129	p.Tyr129Gln	1.09	0.12	Neutral	1.09	0.12	Neutral
129	p.Tyr129Pro	8.53	3.09	Deleterious	8.53	3.09	Deleterious
129	p.Tyr129Leu	0.89	-0.17	Neutral	0.89	-0.17	Neutral
129	p.Tyr129Asp	2.12	1.09	Indeterminate	2.12	1.09	Indeterminate
129	p.Tyr129Glu	1.00	0.01	Neutral	1.00	0.01	Neutral
129	p.Tyr129Ala	1.12	0.16	Neutral	1.12	0.16	Neutral
129	p.Tyr129Gly	1.39	0.47	Indeterminate	1.39	0.47	Indeterminate
129	p.Tyr129Val	1.17	0.23	Neutral	1.17	0.23	Neutral
129	p.Tyr129Tyr	1.00	0.00	Neutral	1.00	0.00	Neutral
129	p.Tyr129Cys	1.07	0.10	Neutral	1.07	0.10	Neutral
129	p.Tyr129Trp	0.89	-0.16	Neutral	0.89	-0.16	Neutral
129	p.Tyr129Phe	1.17	0.23	Neutral	1.17	0.23	Neutral
130	p.Leu130Asn	1.95	0.96	Indeterminate	1.95	0.96	Indeterminate
130	p.Leu130Lys	3.66	1.87	Deleterious	3.66	1.87	Deleterious
130	p.Leu130Thr	1.07	0.10	Neutral	1.07	0.10	Neutral
130	p.Leu130Arg	3.66	1.87	Deleterious	3.66	1.87	Deleterious
130	p.Leu130Ser	1.22	0.29	Indeterminate	1.22	0.29	Indeterminate
130	p.Leu130Ile	0.91	-0.13	Neutral	0.91	-0.13	Neutral
130	p.Leu130Met	0.93	-0.11	Neutral	0.93	-0.11	Neutral
130	p.Leu130His	1.79	0.84	Indeterminate	1.79	0.84	Indeterminate
130	p.Leu130Gln	1.73	0.79	Indeterminate	1.73	0.79	Indeterminate
130	p.Leu130Pro	3.71	1.89	Deleterious	3.71	1.89	Deleterious
130	p.Leu130Leu	1.00	0.00	Neutral	1.00	0.00	Neutral
130	p.Leu130Asp	2.94	1.56	Deleterious	2.94	1.56	Deleterious
130	p.Leu130Glu	2.74	1.46	Deleterious	2.74	1.46	Deleterious
130	p.Leu130Ala	1.05	0.07	Neutral	1.05	0.07	Neutral
130	p.Leu130Gly	1.08	0.12	Neutral	1.08	0.12	Neutral
130	p.Leu130Val	0.87	-0.20	Neutral	0.87	-0.20	Neutral
130	p.Leu130Tyr	1.08	0.12	Neutral	1.08	0.12	Neutral
130	p.Leu130Cys	1.06	0.09	Neutral	1.06	0.09	Neutral
130	p.Leu130Trp	1.20	0.26	Indeterminate	1.20	0.26	Indeterminate
130	p.Leu130Phe	1.00	0.00	Neutral	1.00	0.00	Neutral
131	p.Arg131Asn	0.97	-0.05	Neutral	0.97	-0.05	Neutral
131	p.Arg131Lys	0.83	-0.27	Neutral	0.83	-0.27	Neutral
131	p.Arg131Thr	1.74	0.80	Indeterminate	1.74	0.80	Indeterminate
131	p.Arg131Arg	1.00	0.00	Neutral	1.00	0.00	Neutral
131	p.Arg131Ser	1.12	0.16	Neutral	1.12	0.16	Neutral
131	p.Arg131Ile	0.86	-0.21	Neutral	0.86	-0.21	Neutral
131	p.Arg131Met	0.99	-0.01	Neutral	0.99	-0.01	Neutral
131	p.Arg131His	1.13	0.18	Neutral	1.13	0.18	Neutral
131	p.Arg131Gln	2.44	1.28	Deleterious	2.44	1.28	Deleterious
131	p.Arg131Pro	2.12	1.08	Indeterminate	2.12	1.08	Indeterminate
131	p.Arg131Leu	0.76	-0.40	Neutral	0.76	-0.40	Neutral
131	p.Arg131Asp	0.78	-0.37	Neutral	0.78	-0.37	Neutral
131	p.Arg131Glu	0.87	-0.20	Neutral	0.87	-0.20	Neutral
131	p.Arg131Ala	1.15	0.21	Neutral	1.15	0.21	Neutral
131	p.Arg131Gly	1.02	0.02	Neutral	1.02	0.02	Neutral
131	p.Arg131Val	0.90	-0.16	Neutral	0.90	-0.16	Neutral
131	p.Arg131Tyr	0.94	-0.10	Neutral	0.94	-0.10	Neutral
131	p.Arg131Cys	3.75	1.91	Deleterious	3.75	1.91	Deleterious
131	p.Arg131Trp	1.21	0.28	Indeterminate	1.21	0.28	Indeterminate
131	p.Arg131Phe	0.69	-0.53	Neutral	0.69	-0.53	Neutral
132	p.Ala132Asn	2.18	1.12	Deleterious	2.18	1.12	Deleterious

132	p.Ala132Lys	1.27	0.35	Indeterminate				1.27	0.35	Indeterminate
132	p.Ala132Thr	1.19	0.25	Indeterminate				1.19	0.25	Indeterminate
132	p.Ala132Arg	1.18	0.24	Neutral				1.18	0.24	Neutral
132	p.Ala132Ser	2.27	1.18	Deleterious				2.27	1.18	Deleterious
132	p.Ala132Ile	1.48	0.56	Indeterminate				1.48	0.56	Indeterminate
132	p.Ala132Met	1.34	0.42	Indeterminate				1.34	0.42	Indeterminate
132	p.Ala132His	1.66	0.73	Indeterminate				1.66	0.73	Indeterminate
132	p.Ala132Gln	1.21	0.27	Indeterminate				1.21	0.27	Indeterminate
132	p.Ala132Pro	1.19	0.25	Indeterminate				1.19	0.25	Indeterminate
132	p.Ala132Leu	1.52	0.60	Indeterminate				1.52	0.60	Indeterminate
132	p.Ala132Asp	1.96	0.97	Indeterminate				1.96	0.97	Indeterminate
132	p.Ala132Glu	1.51	0.60	Indeterminate				1.51	0.60	Indeterminate
132	p.Ala132Ala	1.00	0.00	Neutral				1.00	0.00	Neutral
132	p.Ala132Gly	5.74	2.52	Deleterious				5.74	2.52	Deleterious
132	p.Ala132Val	1.35	0.44	Indeterminate				1.35	0.44	Indeterminate
132	p.Ala132Tyr	1.42	0.51	Indeterminate				1.42	0.51	Indeterminate
132	p.Ala132Cys	1.93	0.95	Indeterminate				1.93	0.95	Indeterminate
132	p.Ala132Trp	1.27	0.34	Indeterminate				1.27	0.34	Indeterminate
132	p.Ala132Phe	1.19	0.25	Indeterminate				1.19	0.25	Indeterminate
133	p.Ala133Asn	0.69	-0.53	Neutral				0.69	-0.53	Neutral
133	p.Ala133Lys	0.77	-0.38	Neutral				0.77	-0.38	Neutral
133	p.Ala133Thr	0.79	-0.34	Neutral				0.79	-0.34	Neutral
133	p.Ala133Arg	1.09	0.13	Neutral				1.09	0.13	Neutral
133	p.Ala133Ser	0.90	-0.15	Neutral				0.90	-0.15	Neutral
133	p.Ala133Ile	0.97	-0.04	Neutral				0.97	-0.04	Neutral
133	p.Ala133Met	0.76	-0.40	Neutral				0.76	-0.40	Neutral
133	p.Ala133His	0.69	-0.53	Neutral				0.69	-0.53	Neutral
133	p.Ala133Gln	0.77	-0.38	Neutral				0.77	-0.38	Neutral
133	p.Ala133Pro	1.00	0.00	Neutral				1.00	0.00	Neutral
133	p.Ala133Leu	0.91	-0.14	Neutral				0.91	-0.14	Neutral
133	p.Ala133Asp	1.18	0.24	Indeterminate				1.18	0.24	Indeterminate
133	p.Ala133Glu	1.16	0.22	Neutral				1.16	0.22	Neutral
133	p.Ala133Ala	1.00	0.00	Neutral				1.00	0.00	Neutral
133	p.Ala133Gly	1.05	0.07	Neutral				1.05	0.07	Neutral
133	p.Ala133Val	1.21	0.28	Indeterminate				1.21	0.28	Indeterminate
133	p.Ala133Tyr	1.06	0.09	Neutral				1.06	0.09	Neutral
133	p.Ala133Cys	0.72	-0.48	Neutral				0.72	-0.48	Neutral
133	p.Ala133Trp	0.89	-0.17	Neutral				0.89	-0.17	Neutral
133	p.Ala133Phe	0.93	-0.10	Neutral				0.93	-0.10	Neutral
134	p.Ala134Asn	1.00	0.00	Neutral	0.79	-0.34	Neutral	0.89	-0.16	Neutral
134	p.Ala134Lys	1.13	0.17	Neutral	0.84	-0.26	Neutral	0.98	-0.03	Neutral
134	p.Ala134Thr	0.97	-0.04	Neutral	1.21	0.27	Indeterminate	1.09	0.12	Neutral
134	p.Ala134Arg	1.17	0.22	Neutral	0.45	-1.16	Neutral	0.81	-0.31	Neutral
134	p.Ala134Ser	0.98	-0.03	Neutral	1.23	0.30	Indeterminate	1.10	0.14	Neutral
134	p.Ala134Ile	0.95	-0.07	Neutral	0.98	-0.02	Neutral	0.97	-0.05	Neutral
134	p.Ala134Met	1.01	0.02	Neutral	0.87	-0.20	Neutral	0.94	-0.08	Neutral
134	p.Ala134His	1.01	0.01	Neutral	0.94	-0.09	Neutral	0.98	-0.04	Neutral
134	p.Ala134Gln	1.02	0.03	Neutral	0.95	-0.07	Neutral	0.98	-0.02	Neutral
134	p.Ala134Pro	0.99	-0.01	Neutral	0.74	-0.43	Neutral	0.87	-0.21	Neutral
134	p.Ala134Leu	1.00	-0.01	Neutral	0.79	-0.33	Neutral	0.90	-0.16	Neutral
134	p.Ala134Asp	0.99	-0.02	Neutral	0.97	-0.05	Neutral	0.98	-0.03	Neutral
134	p.Ala134Glu	1.09	0.12	Neutral	0.68	-0.55	Neutral	0.88	-0.18	Neutral
134	p.Ala134Ala	1.00	0.00	Neutral	1.00	0.00	Neutral	1.00	0.00	Neutral
134	p.Ala134Gly	1.05	0.07	Neutral	0.85	-0.23	Neutral	0.95	-0.07	Neutral
134	p.Ala134Val	1.04	0.06	Neutral	1.06	0.08	Neutral	1.05	0.07	Neutral
134	p.Ala134Tyr	0.98	-0.03	Neutral	1.25	0.32	Indeterminate	1.11	0.15	Neutral
134	p.Ala134Cys	0.96	-0.06	Neutral	1.28	0.36	Indeterminate	1.12	0.16	Neutral
134	p.Ala134Trp	1.26	0.33	Indeterminate	0.49	-1.02	Neutral	0.88	-0.19	Neutral
134	p.Ala134Phe	0.96	-0.06	Neutral	0.82	-0.29	Neutral	0.89	-0.17	Neutral
135	p.Gly135Asn	0.63	-0.66	Neutral				0.63	-0.66	Neutral
135	p.Gly135Lys	1.02	0.03	Neutral				1.02	0.03	Neutral
135	p.Gly135Thr	0.87	-0.21	Neutral				0.87	-0.21	Neutral
135	p.Gly135Arg	0.65	-0.63	Neutral				0.65	-0.63	Neutral
135	p.Gly135Ser	0.88	-0.19	Neutral				0.88	-0.19	Neutral
135	p.Gly135Ile	1.11	0.14	Neutral				1.11	0.14	Neutral
135	p.Gly135Met	0.97	-0.04	Neutral				0.97	-0.04	Neutral
135	p.Gly135His	1.01	0.01	Neutral				1.01	0.01	Neutral
135	p.Gly135Gln	0.86	-0.22	Neutral				0.86	-0.22	Neutral
135	p.Gly135Pro	0.94	-0.08	Neutral				0.94	-0.08	Neutral
135	p.Gly135Leu	1.05	0.06	Neutral				1.05	0.06	Neutral
135	p.Gly135Asp	0.88	-0.18	Neutral				0.88	-0.18	Neutral
135	p.Gly135Glu	1.92	0.94	Indeterminate				1.92	0.94	Indeterminate
135	p.Gly135Ala	0.80	-0.32	Neutral				0.80	-0.32	Neutral
135	p.Gly135Gly	1.00	0.00	Neutral				1.00	0.00	Neutral
135	p.Gly135Val	0.92	-0.13	Neutral				0.92	-0.13	Neutral
135	p.Gly135Tyr	0.91	-0.14	Neutral				0.91	-0.14	Neutral
135	p.Gly135Cys	0.74	-0.43	Neutral				0.74	-0.43	Neutral
135	p.Gly135Trp	0.93	-0.10	Neutral				0.93	-0.10	Neutral
135	p.Gly135Phe	0.98	-0.04	Neutral				0.98	-0.04	Neutral
136	p.Gly136Asn	0.89	-0.16	Neutral	1.51	0.59	Indeterminate	1.20	0.26	Indeterminate
136	p.Gly136Lys	0.83	-0.27	Neutral	0.71	-0.50	Neutral	0.77	-0.38	Neutral
136	p.Gly136Thr	0.89	-0.17	Neutral	0.33	-1.61	Neutral	0.61	-0.71	Neutral
136	p.Gly136Arg	0.91	-0.14	Neutral	0.71	-0.50	Neutral	0.81	-0.31	Neutral
136	p.Gly136Ser	0.90	-0.16	Neutral	1.33	0.41	Indeterminate	1.11	0.15	Neutral
136	p.Gly136Ile	0.91	-0.13	Neutral	0.49	-1.02	Neutral	0.70	-0.51	Neutral
136	p.Gly136Met	0.90	-0.15	Neutral	0.48	-1.06	Neutral	0.69	-0.53	Neutral
136	p.Gly136His	0.83	-0.28	Neutral	1.52	0.60	Indeterminate	1.17	0.23	Neutral
136	p.Gly136Gln	0.83	-0.27	Neutral	0.90	-0.14	Neutral	0.87	-0.21	Neutral
136	p.Gly136Pro	0.92	-0.12	Neutral	1.59	0.67	Indeterminate	1.26	0.33	Indeterminate
136	p.Gly136Leu	0.89	-0.17	Neutral	1.38	0.47	Indeterminate	1.13	0.18	Neutral
136	p.Gly136Asp	0.89	-0.17	Neutral	1.95	0.96	Indeterminate	1.42	0.50	Indeterminate
136	p.Gly136Glu	0.84	-0.25	Neutral	0.56	-0.82	Neutral	0.70	-0.51	Neutral
136	p.Gly136Ala	0.97	-0.05	Neutral	0.33	-1.59	Neutral	0.65	-0.62	Neutral
136	p.Gly136Gly	1.00	0.00	Neutral	1.00	0.00	Neutral	1.00	0.00	Neutral
136	p.Gly136Val	0.96	-0.06	Neutral	0.41	-1.30	Neutral	0.68	-0.55	Neutral
136	p.Gly136Tyr	1.08	0.11	Neutral	0.74	-0.44	Neutral	0.91	-0.14	Neutral
136	p.Gly136Cys	0.90	-0.15	Neutral	1.39	0.48	Indeterminate	1.15	0.20	Neutral
136	p.Gly136Trp	0.98	-0.03	Neutral	0.24	-2.06	Neutral	0.61	-0.71	Neutral
136	p.Gly136Phe	0.86	-0.22	Neutral	1.15	0.20	Indeterminate	1.00	0.01	Neutral
137	p.Thr137Asn	1.02	0.03	Neutral				1.02	0.03	Neutral
137	p.Thr137Lys	0.94	-0.09	Neutral				0.94	-0.09	Neutral

137	p.Thr137Thr	1.00	0.00	Neutral				1.00	0.00	Neutral
137	p.Thr137Arg	1.09	0.13	Neutral				1.09	0.13	Neutral
137	p.Thr137Ser	1.05	0.08	Neutral				1.05	0.08	Neutral
137	p.Thr137Ile	1.03	0.05	Neutral				1.03	0.05	Neutral
137	p.Thr137Met	1.10	0.14	Neutral				1.10	0.14	Neutral
137	p.Thr137His	1.03	0.04	Neutral				1.03	0.04	Neutral
137	p.Thr137Gln	0.77	-0.38	Neutral				0.77	-0.38	Neutral
137	p.Thr137Pro	0.83	-0.27	Neutral				0.83	-0.27	Neutral
137	p.Thr137Leu	1.02	0.03	Neutral				1.02	0.03	Neutral
137	p.Thr137Asp	0.96	-0.06	Neutral				0.96	-0.06	Neutral
137	p.Thr137Glu	1.03	0.05	Neutral				1.03	0.05	Neutral
137	p.Thr137Ala	1.16	0.22	Neutral				1.16	0.22	Neutral
137	p.Thr137Gly	1.09	0.12	Neutral				1.09	0.12	Neutral
137	p.Thr137Val	1.12	0.17	Neutral				1.12	0.17	Neutral
137	p.Thr137Tyr	0.86	-0.21	Neutral				0.86	-0.21	Neutral
137	p.Thr137Cys	1.09	0.13	Neutral				1.09	0.13	Neutral
137	p.Thr137Trp	0.97	-0.05	Neutral				0.97	-0.05	Neutral
137	p.Thr137Phe	1.00	0.00	Neutral				1.00	0.00	Neutral
138	p.Arg138Asn	1.13	0.18	Neutral				1.13	0.18	Neutral
138	p.Arg138Lys	0.92	-0.12	Neutral				0.92	-0.12	Neutral
138	p.Arg138Thr	1.05	0.07	Neutral				1.05	0.07	Neutral
138	p.Arg138Arg	1.00	0.00	Neutral				1.00	0.00	Neutral
138	p.Arg138Ser	0.79	-0.33	Neutral				0.79	-0.33	Neutral
138	p.Arg138Ile	0.90	-0.16	Neutral				0.90	-0.16	Neutral
138	p.Arg138Met	0.90	-0.15	Neutral				0.90	-0.15	Neutral
138	p.Arg138His	0.99	-0.01	Neutral				0.99	-0.01	Neutral
138	p.Arg138Gln	0.93	-0.10	Neutral				0.93	-0.10	Neutral
138	p.Arg138Pro	1.24	0.31	Indeterminate				1.24	0.31	Indeterminate
138	p.Arg138Leu	0.94	-0.09	Neutral				0.94	-0.09	Neutral
138	p.Arg138Asp	0.97	-0.05	Neutral				0.97	-0.05	Neutral
138	p.Arg138Glu	0.94	-0.08	Neutral				0.94	-0.08	Neutral
138	p.Arg138Ala	0.58	-0.80	Neutral				0.58	-0.80	Neutral
138	p.Arg138Gly	0.96	-0.06	Neutral				0.96	-0.06	Neutral
138	p.Arg138Val	0.90	-0.15	Neutral				0.90	-0.15	Neutral
138	p.Arg138Tyr	0.72	-0.47	Neutral				0.72	-0.47	Neutral
138	p.Arg138Cys	0.86	-0.22	Neutral				0.86	-0.22	Neutral
138	p.Arg138Trp	0.81	-0.31	Neutral				0.81	-0.31	Neutral
138	p.Arg138Phe	0.89	-0.18	Neutral				0.89	-0.18	Neutral
139	p.Gly139Asn	0.98	-0.04	Neutral	1.01	0.02	Neutral	1.00	-0.01	Neutral
139	p.Gly139Lys	1.05	0.08	Neutral	1.28	0.36	Indeterminate	1.17	0.23	Neutral
139	p.Gly139Thr	0.88	-0.19	Neutral	1.27	0.35	Indeterminate	1.07	0.10	Neutral
139	p.Gly139Arg	0.87	-0.20	Neutral	1.15	0.20	Indeterminate	1.01	0.01	Neutral
139	p.Gly139Ser	1.20	0.27	Indeterminate	1.26	0.33	Indeterminate	1.23	0.30	Indeterminate
139	p.Gly139Ile	0.99	-0.02	Neutral	1.18	0.23	Indeterminate	1.08	0.11	Neutral
139	p.Gly139Met	1.14	0.19	Neutral	1.24	0.31	Indeterminate	1.19	0.25	Indeterminate
139	p.Gly139His	0.88	-0.18	Neutral	1.12	0.16	Indeterminate	1.00	0.00	Neutral
139	p.Gly139Gln	0.93	-0.11	Neutral	1.02	0.03	Neutral	0.98	-0.04	Neutral
139	p.Gly139Pro	1.30	0.38	Indeterminate	0.92	-0.12	Neutral	1.11	0.15	Neutral
139	p.Gly139Leu	0.85	-0.24	Neutral	1.34	0.42	Indeterminate	1.09	0.13	Neutral
139	p.Gly139Asp	0.89	-0.16	Neutral	1.37	0.46	Indeterminate	1.13	0.18	Neutral
139	p.Gly139Glu	1.12	0.16	Neutral	1.24	0.31	Indeterminate	1.18	0.24	Neutral
139	p.Gly139Ala	0.80	-0.32	Neutral	1.38	0.46	Indeterminate	1.09	0.12	Neutral
139	p.Gly139Gly	1.00	0.00	Neutral	1.00	0.00	Neutral	1.00	0.00	Neutral
139	p.Gly139Val	0.89	-0.17	Neutral	1.04	0.05	Neutral	0.96	-0.06	Neutral
139	p.Gly139Tyr	1.38	0.46	Indeterminate	1.35	0.43	Indeterminate	1.36	0.45	Indeterminate
139	p.Gly139Cys	0.86	-0.23	Neutral	1.03	0.04	Neutral	0.94	-0.09	Neutral
139	p.Gly139Trp	0.91	-0.14	Neutral	1.18	0.23	Indeterminate	1.04	0.06	Neutral
139	p.Gly139Phe	1.15	0.20	Neutral	1.30	0.37	Indeterminate	1.22	0.29	Indeterminate
140	p.Ser140Asn	1.02	0.03	Neutral				1.02	0.03	Neutral
140	p.Ser140Lys	0.95	-0.08	Neutral				0.95	-0.08	Neutral
140	p.Ser140Thr	0.98	-0.02	Neutral				0.98	-0.02	Neutral
140	p.Ser140Arg	1.18	0.23	Neutral				1.18	0.23	Neutral
140	p.Ser140Ser	1.00	0.00	Neutral				1.00	0.00	Neutral
140	p.Ser140Ile	0.95	-0.08	Neutral				0.95	-0.08	Neutral
140	p.Ser140Met	0.94	-0.08	Neutral				0.94	-0.08	Neutral
140	p.Ser140His	1.04	0.06	Neutral				1.04	0.06	Neutral
140	p.Ser140Gln	0.99	-0.02	Neutral				0.99	-0.02	Neutral
140	p.Ser140Pro	1.20	0.27	Indeterminate				1.20	0.27	Indeterminate
140	p.Ser140Leu	0.90	-0.15	Neutral				0.90	-0.15	Neutral
140	p.Ser140Asp	0.98	-0.03	Neutral				0.98	-0.03	Neutral
140	p.Ser140Glu	0.90	-0.16	Neutral				0.90	-0.16	Neutral
140	p.Ser140Ala	1.07	0.09	Neutral				1.07	0.09	Neutral
140	p.Ser140Gly	1.28	0.36	Indeterminate				1.28	0.36	Indeterminate
140	p.Ser140Val	0.97	-0.04	Neutral				0.97	-0.04	Neutral
140	p.Ser140Tyr	1.10	0.14	Neutral				1.10	0.14	Neutral
140	p.Ser140Cys	1.03	0.05	Neutral				1.03	0.05	Neutral
140	p.Ser140Trp	0.98	-0.03	Neutral				0.98	-0.03	Neutral
140	p.Ser140Phe	0.92	-0.12	Neutral				0.92	-0.12	Neutral
141	p.Asn141Asn	1.00	0.00	Neutral				1.00	0.00	Neutral
141	p.Asn141Lys	0.78	-0.36	Neutral				0.78	-0.36	Neutral
141	p.Asn141Thr	0.98	-0.03	Neutral				0.98	-0.03	Neutral
141	p.Asn141Arg	0.97	-0.04	Neutral				0.97	-0.04	Neutral
141	p.Asn141Ser	0.84	-0.25	Neutral				0.84	-0.25	Neutral
141	p.Asn141Ile	0.95	-0.08	Neutral				0.95	-0.08	Neutral
141	p.Asn141Met	0.92	-0.13	Neutral				0.92	-0.13	Neutral
141	p.Asn141His	0.86	-0.21	Neutral				0.86	-0.21	Neutral
141	p.Asn141Gln	1.01	0.01	Neutral				1.01	0.01	Neutral
141	p.Asn141Pro	0.87	-0.21	Neutral				0.87	-0.21	Neutral
141	p.Asn141Leu	0.86	-0.22	Neutral				0.86	-0.22	Neutral
141	p.Asn141Asp	0.92	-0.12	Neutral				0.92	-0.12	Neutral
141	p.Asn141Glu	0.97	-0.05	Neutral				0.97	-0.05	Neutral
141	p.Asn141Ala	0.90	-0.14	Neutral				0.90	-0.14	Neutral
141	p.Asn141Gly	0.88	-0.18	Neutral				0.88	-0.18	Neutral
141	p.Asn141Val	0.85	-0.24	Neutral				0.85	-0.24	Neutral
141	p.Asn141Tyr	0.87	-0.20	Neutral				0.87	-0.20	Neutral
141	p.Asn141Cys	0.87	-0.20	Neutral				0.87	-0.20	Neutral
141	p.Asn141Trp	0.87	-0.20	Neutral				0.87	-0.20	Neutral
141	p.Asn141Phe	0.95	-0.07	Neutral				0.95	-0.07	Neutral
142	p.His142Asn	0.97	-0.05	Neutral				0.97	-0.05	Neutral
142	p.His142Lys	1.05	0.08	Neutral				1.05	0.08	Neutral
142	p.His142Thr	1.08	0.11	Neutral				1.08	0.11	Neutral

142	p.His142Arg	0.81	-0.30	Neutral	0.81	-0.30	Neutral
142	p.His142Ser	0.96	-0.06	Neutral	0.96	-0.06	Neutral
142	p.His142Ile	0.96	-0.06	Neutral	0.96	-0.06	Neutral
142	p.His142Met	0.99	-0.02	Neutral	0.99	-0.02	Neutral
142	p.His142His	1.00	0.00	Neutral	1.00	0.00	Neutral
142	p.His142Gln	1.09	0.12	Neutral	1.09	0.12	Neutral
142	p.His142Pro	0.95	-0.07	Neutral	0.95	-0.07	Neutral
142	p.His142Leu	0.91	-0.14	Neutral	0.91	-0.14	Neutral
142	p.His142Asp	1.09	0.12	Neutral	1.09	0.12	Neutral
142	p.His142Glu	0.97	-0.04	Neutral	0.97	-0.04	Neutral
142	p.His142Ala	0.96	-0.05	Neutral	0.96	-0.05	Neutral
142	p.His142Gly	0.94	-0.09	Neutral	0.94	-0.09	Neutral
142	p.His142Val	1.01	0.02	Neutral	1.01	0.02	Neutral
142	p.His142Tyr	0.90	-0.15	Neutral	0.90	-0.15	Neutral
142	p.His142Cys	0.96	-0.05	Neutral	0.96	-0.05	Neutral
142	p.His142Trp	0.91	-0.13	Neutral	0.91	-0.13	Neutral
142	p.His142Phe	1.12	0.17	Neutral	1.12	0.17	Neutral
143	p.Ala143Asn	1.00	0.01	Neutral	1.00	0.01	Neutral
143	p.Ala143Lys	0.97	-0.05	Neutral	0.97	-0.05	Neutral
143	p.Ala143Thr	0.96	-0.06	Neutral	0.96	-0.06	Neutral
143	p.Ala143Arg	0.97	-0.05	Neutral	0.97	-0.05	Neutral
143	p.Ala143Ser	0.93	-0.10	Neutral	0.93	-0.10	Neutral
143	p.Ala143Ile	0.92	-0.12	Neutral	0.92	-0.12	Neutral
143	p.Ala143Met	0.98	-0.02	Neutral	0.98	-0.02	Neutral
143	p.Ala143His	0.99	-0.02	Neutral	0.99	-0.02	Neutral
143	p.Ala143Gln	1.00	0.00	Neutral	1.00	0.00	Neutral
143	p.Ala143Pro	1.02	0.03	Neutral	1.02	0.03	Neutral
143	p.Ala143Leu	0.95	-0.08	Neutral	0.95	-0.08	Neutral
143	p.Ala143Asp	0.91	-0.13	Neutral	0.91	-0.13	Neutral
143	p.Ala143Glu	0.98	-0.03	Neutral	0.98	-0.03	Neutral
143	p.Ala143Ala	1.00	0.00	Neutral	1.00	0.00	Neutral
143	p.Ala143Gly	1.03	0.04	Neutral	1.03	0.04	Neutral
143	p.Ala143Val	0.96	-0.05	Neutral	0.96	-0.05	Neutral
143	p.Ala143Tyr	1.01	0.02	Neutral	1.01	0.02	Neutral
143	p.Ala143Cys	1.04	0.06	Neutral	1.04	0.06	Neutral
143	p.Ala143Trp	1.09	0.13	Neutral	1.09	0.13	Neutral
143	p.Ala143Phe	1.01	0.01	Neutral	1.01	0.01	Neutral
144	p.Arg144Asn	0.88	-0.18	Neutral	0.88	-0.18	Neutral
144	p.Arg144Lys	0.90	-0.15	Neutral	0.90	-0.15	Neutral
144	p.Arg144Thr	0.89	-0.16	Neutral	0.89	-0.16	Neutral
144	p.Arg144Arg	1.00	0.00	Neutral	1.00	0.00	Neutral
144	p.Arg144Ser	0.88	-0.18	Neutral	0.88	-0.18	Neutral
144	p.Arg144Ile	0.92	-0.11	Neutral	0.92	-0.11	Neutral
144	p.Arg144Met	0.89	-0.17	Neutral	0.89	-0.17	Neutral
144	p.Arg144His	0.92	-0.13	Neutral	0.92	-0.13	Neutral
144	p.Arg144Gln	0.95	-0.07	Neutral	0.95	-0.07	Neutral
144	p.Arg144Pro	0.90	-0.15	Neutral	0.90	-0.15	Neutral
144	p.Arg144Leu	0.96	-0.06	Neutral	0.96	-0.06	Neutral
144	p.Arg144Asp	0.89	-0.16	Neutral	0.89	-0.16	Neutral
144	p.Arg144Glu	0.96	-0.07	Neutral	0.96	-0.07	Neutral
144	p.Arg144Ala	0.95	-0.08	Neutral	0.95	-0.08	Neutral
144	p.Arg144Gly	0.97	-0.05	Neutral	0.97	-0.05	Neutral
144	p.Arg144Val	0.91	-0.14	Neutral	0.91	-0.14	Neutral
144	p.Arg144Tyr	0.91	-0.14	Neutral	0.91	-0.14	Neutral
144	p.Arg144Cys	0.84	-0.25	Neutral	0.84	-0.25	Neutral
144	p.Arg144Trp	0.91	-0.13	Neutral	0.91	-0.13	Neutral
144	p.Arg144Phe	0.90	-0.15	Neutral	0.90	-0.15	Neutral
145	p.Ile145Asn	0.99	-0.02	Neutral	0.99	-0.02	Neutral
145	p.Ile145Lys	1.13	0.18	Neutral	1.13	0.18	Neutral
145	p.Ile145Thr	1.05	0.07	Neutral	1.05	0.07	Neutral
145	p.Ile145Arg	1.18	0.24	Indeterminate	1.18	0.24	Indeterminate
145	p.Ile145Ser	1.11	0.15	Neutral	1.11	0.15	Neutral
145	p.Ile145Ile	1.00	0.00	Neutral	1.00	0.00	Neutral
145	p.Ile145Met	1.21	0.28	Indeterminate	1.21	0.28	Indeterminate
145	p.Ile145His	0.94	-0.08	Neutral	0.94	-0.08	Neutral
145	p.Ile145Gln	1.10	0.14	Neutral	1.10	0.14	Neutral
145	p.Ile145Pro	0.97	-0.04	Neutral	0.97	-0.04	Neutral
145	p.Ile145Leu	1.04	0.05	Neutral	1.04	0.05	Neutral
145	p.Ile145Asp	1.04	0.05	Neutral	1.04	0.05	Neutral
145	p.Ile145Glu	1.05	0.06	Neutral	1.05	0.06	Neutral
145	p.Ile145Ala	1.15	0.20	Neutral	1.15	0.20	Neutral
145	p.Ile145Gly	0.98	-0.03	Neutral	0.98	-0.03	Neutral
145	p.Ile145Val	1.13	0.17	Neutral	1.13	0.17	Neutral
145	p.Ile145Tyr	1.02	0.03	Neutral	1.02	0.03	Neutral
145	p.Ile145Cys	0.92	-0.12	Neutral	0.92	-0.12	Neutral
145	p.Ile145Trp	1.04	0.05	Neutral	1.04	0.05	Neutral
145	p.Ile145Phe	0.98	-0.03	Neutral	0.98	-0.03	Neutral
146	p.Asp146Asn	1.05	0.07	Neutral	1.05	0.07	Neutral
146	p.Asp146Lys	0.78	-0.36	Neutral	0.78	-0.36	Neutral
146	p.Asp146Thr	0.95	-0.07	Neutral	0.95	-0.07	Neutral
146	p.Asp146Arg	0.95	-0.07	Neutral	0.95	-0.07	Neutral
146	p.Asp146Ser	0.88	-0.18	Neutral	0.88	-0.18	Neutral
146	p.Asp146Ile	0.79	-0.33	Neutral	0.79	-0.33	Neutral
146	p.Asp146Met	0.96	-0.05	Neutral	0.96	-0.05	Neutral
146	p.Asp146His	1.01	0.01	Neutral	1.01	0.01	Neutral
146	p.Asp146Gln	0.98	-0.03	Neutral	0.98	-0.03	Neutral
146	p.Asp146Pro	1.00	0.00	Neutral	1.00	0.00	Neutral
146	p.Asp146Leu	0.93	-0.11	Neutral	0.93	-0.11	Neutral
146	p.Asp146Asp	1.00	0.00	Neutral	1.00	0.00	Neutral
146	p.Asp146Glu	0.87	-0.20	Neutral	0.87	-0.20	Neutral
146	p.Asp146Ala	0.86	-0.22	Neutral	0.86	-0.22	Neutral
146	p.Asp146Gly	0.86	-0.22	Neutral	0.86	-0.22	Neutral
146	p.Asp146Val	0.86	-0.21	Neutral	0.86	-0.21	Neutral
146	p.Asp146Tyr	1.00	0.00	Neutral	1.00	0.00	Neutral
146	p.Asp146Cys	1.01	0.02	Neutral	1.01	0.02	Neutral
146	p.Asp146Trp	0.85	-0.23	Neutral	0.85	-0.23	Neutral
146	p.Asp146Phe	1.03	0.05	Neutral	1.03	0.05	Neutral
147	p.Ala147Asn	1.05	0.07	Neutral	1.05	0.07	Neutral
147	p.Ala147Lys	0.91	-0.13	Neutral	0.91	-0.13	Neutral
147	p.Ala147Thr	1.03	0.04	Neutral	1.03	0.04	Neutral
147	p.Ala147Arg	0.91	-0.14	Neutral	0.91	-0.14	Neutral

147	p.Ala147Ser	0.91	-0.14	Neutral	0.91	-0.14	Neutral
147	p.Ala147Ile	1.12	0.16	Neutral	1.12	0.16	Neutral
147	p.Ala147Met	1.08	0.11	Neutral	1.08	0.11	Neutral
147	p.Ala147His	0.89	-0.17	Neutral	0.89	-0.17	Neutral
147	p.Ala147Gln	1.42	0.50	Indeterminate	1.42	0.50	Indeterminate
147	p.Ala147Pro	0.88	-0.18	Neutral	0.88	-0.18	Neutral
147	p.Ala147Leu	1.07	0.09	Neutral	1.07	0.09	Neutral
147	p.Ala147Asp	1.12	0.17	Neutral	1.12	0.17	Neutral
147	p.Ala147Glu	0.94	-0.09	Neutral	0.94	-0.09	Neutral
147	p.Ala147Ala	1.00	0.00	Neutral	1.00	0.00	Neutral
147	p.Ala147Gly	0.98	-0.03	Neutral	0.98	-0.03	Neutral
147	p.Ala147Val	1.04	0.06	Neutral	1.04	0.06	Neutral
147	p.Ala147Tyr	0.89	-0.16	Neutral	0.89	-0.16	Neutral
147	p.Ala147Cys	0.92	-0.12	Neutral	0.92	-0.12	Neutral
147	p.Ala147Trp	0.93	-0.10	Neutral	0.93	-0.10	Neutral
147	p.Ala147Phe	0.89	-0.16	Neutral	0.89	-0.16	Neutral
148	p.Ala148Asn	1.32	0.40	Indeterminate	1.32	0.40	Indeterminate
148	p.Ala148Lys	2.00	1.00	Indeterminate	2.00	1.00	Indeterminate
148	p.Ala148Thr	0.92	-0.12	Neutral	0.92	-0.12	Neutral
148	p.Ala148Arg	1.44	0.53	Indeterminate	1.44	0.53	Indeterminate
148	p.Ala148Ser	1.08	0.11	Neutral	1.08	0.11	Neutral
148	p.Ala148Ile	1.15	0.20	Neutral	1.15	0.20	Neutral
148	p.Ala148Met	1.54	0.62	Indeterminate	1.54	0.62	Indeterminate
148	p.Ala148His	1.27	0.34	Indeterminate	1.27	0.34	Indeterminate
148	p.Ala148Gln	1.31	0.39	Indeterminate	1.31	0.39	Indeterminate
148	p.Ala148Pro	1.19	0.25	Indeterminate	1.19	0.25	Indeterminate
148	p.Ala148Leu	1.21	0.27	Indeterminate	1.21	0.27	Indeterminate
148	p.Ala148Asp	1.27	0.35	Indeterminate	1.27	0.35	Indeterminate
148	p.Ala148Glu	1.39	0.47	Indeterminate	1.39	0.47	Indeterminate
148	p.Ala148Ala	1.00	0.00	Neutral	1.00	0.00	Neutral
148	p.Ala148Gly	1.01	0.01	Neutral	1.01	0.01	Neutral
148	p.Ala148Val	1.55	0.63	Indeterminate	1.55	0.63	Indeterminate
148	p.Ala148Tyr	0.94	-0.09	Neutral	0.94	-0.09	Neutral
148	p.Ala148Cys	0.66	-0.60	Neutral	0.66	-0.60	Neutral
148	p.Ala148Trp	1.28	0.35	Indeterminate	1.28	0.35	Indeterminate
148	p.Ala148Phe	1.94	0.96	Indeterminate	1.94	0.96	Indeterminate
149	p.Glu149Asn	0.80	-0.31	Neutral	0.80	-0.31	Neutral
149	p.Glu149Lys	1.03	0.04	Neutral	1.03	0.04	Neutral
149	p.Glu149Thr	1.08	0.12	Neutral	1.08	0.12	Neutral
149	p.Glu149Arg	0.85	-0.24	Neutral	0.85	-0.24	Neutral
149	p.Glu149Ser	1.14	0.19	Neutral	1.14	0.19	Neutral
149	p.Glu149Ile	1.10	0.14	Neutral	1.10	0.14	Neutral
149	p.Glu149Met	0.79	-0.34	Neutral	0.79	-0.34	Neutral
149	p.Glu149His	0.93	-0.11	Neutral	0.93	-0.11	Neutral
149	p.Glu149Gln	0.88	-0.19	Neutral	0.88	-0.19	Neutral
149	p.Glu149Pro	0.91	-0.14	Neutral	0.91	-0.14	Neutral
149	p.Glu149Leu	0.92	-0.13	Neutral	0.92	-0.13	Neutral
149	p.Glu149Asp	0.86	-0.22	Neutral	0.86	-0.22	Neutral
149	p.Glu149Glu	1.00	0.00	Neutral	1.00	0.00	Neutral
149	p.Glu149Ala	1.00	0.00	Neutral	1.00	0.00	Neutral
149	p.Glu149Gly	0.98	-0.02	Neutral	0.98	-0.02	Neutral
149	p.Glu149Val	0.99	-0.01	Neutral	0.99	-0.01	Neutral
149	p.Glu149Tyr	1.02	0.03	Neutral	1.02	0.03	Neutral
149	p.Glu149Cys	0.86	-0.22	Neutral	0.86	-0.22	Neutral
149	p.Glu149Trp	1.00	0.00	Neutral	1.00	0.00	Neutral
149	p.Glu149Phe	1.06	0.09	Neutral	1.06	0.09	Neutral
150	p.Gly150Asn	0.89	-0.17	Neutral	0.89	-0.17	Neutral
150	p.Gly150Lys	1.03	0.04	Neutral	1.03	0.04	Neutral
150	p.Gly150Thr	0.96	-0.06	Neutral	0.96	-0.06	Neutral
150	p.Gly150Arg	1.03	0.04	Neutral	1.03	0.04	Neutral
150	p.Gly150Ser	0.99	-0.01	Neutral	0.99	-0.01	Neutral
150	p.Gly150Ile	0.92	-0.12	Neutral	0.92	-0.12	Neutral
150	p.Gly150Met	1.07	0.10	Neutral	1.07	0.10	Neutral
150	p.Gly150His	1.04	0.05	Neutral	1.04	0.05	Neutral
150	p.Gly150Gln	0.93	-0.10	Neutral	0.93	-0.10	Neutral
150	p.Gly150Pro	1.00	-0.01	Neutral	1.00	-0.01	Neutral
150	p.Gly150Leu	0.91	-0.14	Neutral	0.91	-0.14	Neutral
150	p.Gly150Asp	0.97	-0.05	Neutral	0.97	-0.05	Neutral
150	p.Gly150Glu	0.88	-0.18	Neutral	0.88	-0.18	Neutral
150	p.Gly150Ala	0.86	-0.21	Neutral	0.86	-0.21	Neutral
150	p.Gly150Gly	1.00	0.00	Neutral	1.00	0.00	Neutral
150	p.Gly150Val	0.97	-0.05	Neutral	0.97	-0.05	Neutral
150	p.Gly150Tyr	0.91	-0.13	Neutral	0.91	-0.13	Neutral
150	p.Gly150Cys	0.77	-0.38	Neutral	0.77	-0.38	Neutral
150	p.Gly150Trp	0.92	-0.12	Neutral	0.92	-0.12	Neutral
150	p.Gly150Phe	0.99	-0.01	Neutral	0.99	-0.01	Neutral
151	p.Pro151Asn	1.03	0.04	Neutral	1.03	0.04	Neutral
151	p.Pro151Lys	0.94	-0.09	Neutral	0.94	-0.09	Neutral
151	p.Pro151Thr	1.03	0.05	Neutral	1.03	0.05	Neutral
151	p.Pro151Arg	0.84	-0.26	Neutral	0.84	-0.26	Neutral
151	p.Pro151Ser	1.03	0.04	Neutral	1.03	0.04	Neutral
151	p.Pro151Ile	0.99	-0.01	Neutral	0.99	-0.01	Neutral
151	p.Pro151Met	0.92	-0.12	Neutral	0.92	-0.12	Neutral
151	p.Pro151His	1.06	0.09	Neutral	1.06	0.09	Neutral
151	p.Pro151Gln	0.90	-0.15	Neutral	0.90	-0.15	Neutral
151	p.Pro151Pro	1.00	0.00	Neutral	1.00	0.00	Neutral
151	p.Pro151Leu	1.01	0.02	Neutral	1.01	0.02	Neutral
151	p.Pro151Asp	1.02	0.03	Neutral	1.02	0.03	Neutral
151	p.Pro151Glu	1.06	0.08	Neutral	1.06	0.08	Neutral
151	p.Pro151Ala	1.09	0.12	Neutral	1.09	0.12	Neutral
151	p.Pro151Gly	1.13	0.17	Neutral	1.13	0.17	Neutral
151	p.Pro151Val	0.95	-0.08	Neutral	0.95	-0.08	Neutral
151	p.Pro151Tyr	0.88	-0.18	Neutral	0.88	-0.18	Neutral
151	p.Pro151Cys	0.89	-0.17	Neutral	0.89	-0.17	Neutral
151	p.Pro151Trp	0.88	-0.18	Neutral	0.88	-0.18	Neutral
151	p.Pro151Phe	0.92	-0.12	Neutral	0.92	-0.12	Neutral
152	p.Ser152Asn	0.87	-0.20	Neutral	0.87	-0.20	Neutral
152	p.Ser152Lys	0.96	-0.06	Neutral	0.96	-0.06	Neutral
152	p.Ser152Thr	0.96	-0.06	Neutral	0.96	-0.06	Neutral
152	p.Ser152Arg	0.89	-0.17	Neutral	0.89	-0.17	Neutral
152	p.Ser152Ser	1.00	0.00	Neutral	1.00	0.00	Neutral

152	p.Ser152Ile	0.76	-0.40	Neutral				0.76	-0.40	Neutral
152	p.Ser152Met	0.97	-0.05	Neutral				0.97	-0.05	Neutral
152	p.Ser152His	0.89	-0.17	Neutral				0.89	-0.17	Neutral
152	p.Ser152Gln	0.79	-0.33	Neutral				0.79	-0.33	Neutral
152	p.Ser152Pro	0.98	-0.03	Neutral				0.98	-0.03	Neutral
152	p.Ser152Leu	0.96	-0.06	Neutral				0.96	-0.06	Neutral
152	p.Ser152Asp	0.89	-0.17	Neutral				0.89	-0.17	Neutral
152	p.Ser152Glu	0.91	-0.13	Neutral				0.91	-0.13	Neutral
152	p.Ser152Ala	1.04	0.06	Neutral				1.04	0.06	Neutral
152	p.Ser152Gly	0.97	-0.04	Neutral				0.97	-0.04	Neutral
152	p.Ser152Val	0.98	-0.03	Neutral				0.98	-0.03	Neutral
152	p.Ser152Tyr	1.10	0.14	Neutral				1.10	0.14	Neutral
152	p.Ser152Cys	0.75	-0.42	Neutral				0.75	-0.42	Neutral
152	p.Ser152Trp	0.89	-0.17	Neutral				0.89	-0.17	Neutral
152	p.Ser152Phe	0.88	-0.19	Neutral				0.88	-0.19	Neutral
153	p.Asp153Asn	1.33	0.41	Indeterminate	0.83	-0.28	Neutral	1.08	0.11	Neutral
153	p.Asp153Lys	0.99	-0.02	Neutral	1.33	0.41	Indeterminate	1.16	0.21	Neutral
153	p.Asp153Thr	1.47	0.56	Indeterminate	1.10	0.14	Neutral	1.29	0.36	Indeterminate
153	p.Asp153Arg	1.25	0.32	Indeterminate	1.37	0.46	Indeterminate	1.31	0.39	Indeterminate
153	p.Asp153Ser	0.82	-0.29	Neutral	0.72	-0.47	Neutral	0.77	-0.37	Neutral
153	p.Asp153Ile	0.91	-0.14	Neutral	1.16	0.22	Indeterminate	1.04	0.05	Neutral
153	p.Asp153Met	1.31	0.39	Indeterminate	0.92	-0.12	Neutral	1.11	0.16	Neutral
153	p.Asp153His	1.34	0.42	Indeterminate	0.92	-0.12	Neutral	1.13	0.18	Neutral
153	p.Asp153Gln	1.39	0.48	Indeterminate	0.95	-0.07	Neutral	1.17	0.23	Neutral
153	p.Asp153Pro	0.68	-0.56	Neutral	0.53	-0.93	Neutral	0.60	-0.73	Neutral
153	p.Asp153Leu	1.34	0.43	Indeterminate	0.94	-0.09	Neutral	1.14	0.19	Neutral
153	p.Asp153Asp	1.00	0.00	Neutral	1.00	0.00	Neutral	1.00	0.00	Neutral
153	p.Asp153Glu	1.18	0.24	Indeterminate	0.82	-0.29	Neutral	1.00	0.00	Neutral
153	p.Asp153Ala	0.67	-0.57	Neutral	0.98	-0.03	Neutral	0.83	-0.27	Neutral
153	p.Asp153Gly	0.72	-0.47	Neutral	0.64	-0.64	Neutral	0.68	-0.55	Neutral
153	p.Asp153Val	1.14	0.19	Neutral	1.20	0.26	Indeterminate	1.17	0.23	Neutral
153	p.Asp153Tyr	0.97	-0.04	Neutral	1.33	0.41	Indeterminate	1.15	0.20	Neutral
153	p.Asp153Cys	1.41	0.50	Indeterminate	1.09	0.13	Neutral	1.25	0.32	Indeterminate
153	p.Asp153Trp	1.25	0.32	Indeterminate	1.23	0.30	Indeterminate	1.24	0.31	Indeterminate
153	p.Asp153Phe	1.04	0.05	Neutral	1.07	0.09	Neutral	1.05	0.07	Neutral
154	p.Ile154Asn	1.10	0.13	Neutral				1.10	0.13	Neutral
154	p.Ile154Lys	0.91	-0.13	Neutral				0.91	-0.13	Neutral
154	p.Ile154Thr	1.09	0.13	Neutral				1.09	0.13	Neutral
154	p.Ile154Arg	1.04	0.06	Neutral				1.04	0.06	Neutral
154	p.Ile154Ser	0.86	-0.21	Neutral				0.86	-0.21	Neutral
154	p.Ile154Ile	1.00	0.00	Neutral				1.00	0.00	Neutral
154	p.Ile154Met	1.03	0.05	Neutral				1.03	0.05	Neutral
154	p.Ile154His	1.13	0.17	Neutral				1.13	0.17	Neutral
154	p.Ile154Gln	1.00	0.00	Neutral				1.00	0.00	Neutral
154	p.Ile154Pro	1.13	0.18	Neutral				1.13	0.18	Neutral
154	p.Ile154Leu	0.93	-0.11	Neutral				0.93	-0.11	Neutral
154	p.Ile154Asp	0.99	-0.01	Neutral				0.99	-0.01	Neutral
154	p.Ile154Glu	1.06	0.09	Neutral				1.06	0.09	Neutral
154	p.Ile154Ala	1.27	0.34	Indeterminate				1.27	0.34	Indeterminate
154	p.Ile154Gly	0.87	-0.20	Neutral				0.87	-0.20	Neutral
154	p.Ile154Val	1.20	0.26	Indeterminate				1.20	0.26	Indeterminate
154	p.Ile154Tyr	1.15	0.20	Neutral				1.15	0.20	Neutral
154	p.Ile154Cys	1.13	0.18	Neutral				1.13	0.18	Neutral
154	p.Ile154Trp	1.23	0.30	Indeterminate				1.23	0.30	Indeterminate
154	p.Ile154Phe	1.06	0.08	Neutral				1.06	0.08	Neutral
155	p.Pro155Asn	0.98	-0.02	Neutral				0.98	-0.02	Neutral
155	p.Pro155Lys	1.19	0.25	Indeterminate				1.19	0.25	Indeterminate
155	p.Pro155Thr	0.18	-2.43	Neutral				0.18	-2.43	Neutral
155	p.Pro155Arg	0.90	-0.15	Neutral				0.90	-0.15	Neutral
155	p.Pro155Ser	2.13	1.09	Deleterious				2.13	1.09	Deleterious
155	p.Pro155Ile	1.37	0.45	Indeterminate				1.37	0.45	Indeterminate
155	p.Pro155Met	0.58	-0.78	Neutral				0.58	-0.78	Neutral
155	p.Pro155His	1.63	0.71	Indeterminate				1.63	0.71	Indeterminate
155	p.Pro155Gln	0.46	-1.11	Neutral				0.46	-1.11	Neutral
155	p.Pro155Pro	1.00	0.00	Neutral				1.00	0.00	Neutral
155	p.Pro155Leu	0.50	-1.00	Neutral				0.50	-1.00	Neutral
155	p.Pro155Asp	2.39	1.26	Deleterious				2.39	1.26	Deleterious
155	p.Pro155Glu	0.55	-0.87	Neutral				0.55	-0.87	Neutral
155	p.Pro155Ala	0.18	-2.45	Neutral				0.18	-2.45	Neutral
155	p.Pro155Gly	0.71	-0.49	Neutral				0.71	-0.49	Neutral
155	p.Pro155Val	1.99	0.99	Indeterminate				1.99	0.99	Indeterminate
155	p.Pro155Tyr	0.70	-0.51	Neutral				0.70	-0.51	Neutral
155	p.Pro155Cys	0.39	-1.35	Neutral				0.39	-1.35	Neutral
155	p.Pro155Trp	1.59	0.67	Indeterminate				1.59	0.67	Indeterminate
155	p.Pro155Phe	0.19	-2.36	Neutral				0.19	-2.36	Neutral
156	p.Asp156Asn	1.05	0.06	Neutral				1.05	0.06	Neutral
156	p.Asp156Lys	0.99	-0.01	Neutral				0.99	-0.01	Neutral
156	p.Asp156Thr	1.07	0.10	Neutral				1.07	0.10	Neutral
156	p.Asp156Arg	1.01	0.01	Neutral				1.01	0.01	Neutral
156	p.Asp156Ser	1.01	0.02	Neutral				1.01	0.02	Neutral
156	p.Asp156Ile	0.97	-0.04	Neutral				0.97	-0.04	Neutral
156	p.Asp156Met	0.99	-0.01	Neutral				0.99	-0.01	Neutral
156	p.Asp156His	0.98	-0.04	Neutral				0.98	-0.04	Neutral
156	p.Asp156Gln	1.19	0.25	Indeterminate				1.19	0.25	Indeterminate
156	p.Asp156Pro	0.99	-0.02	Neutral				0.99	-0.02	Neutral
156	p.Asp156Leu	1.09	0.12	Neutral				1.09	0.12	Neutral
156	p.Asp156Asp	1.00	0.00	Neutral				1.00	0.00	Neutral
156	p.Asp156Glu	1.05	0.07	Neutral				1.05	0.07	Neutral
156	p.Asp156Ala	1.27	0.34	Indeterminate				1.27	0.34	Indeterminate
156	p.Asp156Gly	1.10	0.14	Neutral				1.10	0.14	Neutral
156	p.Asp156Val	1.13	0.18	Neutral				1.13	0.18	Neutral
156	p.Asp156Tyr	1.13	0.17	Neutral				1.13	0.17	Neutral
156	p.Asp156Cys	1.10	0.14	Neutral				1.10	0.14	Neutral
156	p.Asp156Trp	0.90	-0.15	Neutral				0.90	-0.15	Neutral
156	p.Asp156Phe	1.00	0.00	Neutral				1.00	0.00	Neutral
