# Supplementary material for: Functional characterization of all CDKN2A missense variants and comparison to in silico models of pathogenicity
Source: bioRxiv. 2025 Feb 11:2023.12.28.573507. Originally published 2023 Dec 28. Preprint. [Version 3] doi: 10.1101/2023.12.28.573507 (PMC10793438; doi:10.1101/2023.12.28.573507)
Supplement: Supplement 7 [file media-7.pdf]

Appendix 1-table 7. In silico variant effect predictions for *CDKN2A* missense variants.

Protein	ACMG Guidline	This study		AlphaMisssense		ESM1b		VEST		Polyphen-2		SIFT		CADD	
Consequence	Classification	Log P value	Characteritic	Score	Prediction	Score	Prediction	Score	Prediction	Score	Prediction	Score	Prediction	Score	Prediction
p.Met1Asn	VUS	-0.15	Neutral	0.34	Benign	-11.02	Pathogenic	0.44	Neutral	0.19	Benign	0	Deleterious		
p.Met1Lys	VUS	-0.01	Neutral	0.18	Benign	-10.15	Pathogenic	0.48	Neutral	0.04	Benign	0	Deleterious	14.26	Neutral
p.Met1Thr	VUS	-0.14	Neutral	0.11	Benign	-8.46	Pathogenic	0.43	Neutral	0.00	Benign	0	Deleterious	12.50	Neutral
p.Met1Arg	VUS	-0.22	Neutral	0.10	Benign	-8.48	Pathogenic	0.57	Disease	0.00	Benign	0	Deleterious	14.15	Neutral
p.Met1Ser	VUS	-0.01	Neutral	0.15	Benign	-7.86	Pathogenic	0.43	Neutral	0.05	Benign	0	Deleterious		
p.Met1Ile	VUS	-0.44	Neutral	0.22	Benign	-10.40	Pathogenic	0.36	Neutral	0.15	Benign	0	Deleterious	18.53	Deleterious
p.Met1His	VUS	-1.27	Neutral	0.22	Benign	-10.63	Pathogenic	0.41	Neutral	0.62	Possibly damaging	0	Deleterious		
p.Met1Gln	VUS	-0.12	Neutral	0.07	Benign	-9.16	Pathogenic	0.40	Neutral	0.10	Benign	0	Deleterious		
p.Met1Pro	VUS	-0.36	Neutral	0.22	Benign	-7.53	Pathogenic	0.44	Neutral	0.32	Benign	0	Deleterious		
p.Met1Leu	VUS	-0.02	Neutral	0.11	Benign	-8.18	Pathogenic	0.35	Neutral	0.03	Benign	0	Deleterious	16.05	Deleterious
p.Met1Asp	VUS	0.00	Neutral	0.33	Benign	-9.42	Pathogenic	0.43	Neutral	0.05	Benign	0	Deleterious		
p.Met1Glu	VUS	-0.96	Neutral	0.18	Benign	-8.42	Pathogenic	0.46	Neutral	0.00	Benign	0	Deleterious		
p.Met1Ala	VUS	-0.07	Neutral	0.08	Benign	-7.47	Benign	0.39	Neutral	0.02	Benign	0	Deleterious		
p.Met1Gly	VUS	-0.36	Neutral	0.16	Benign	-7.93	Pathogenic	0.44	Neutral	0.00	Benign	0	Deleterious		
p.Met1Val	VUS	-4.16	Neutral	0.07	Benign	-8.26	Pathogenic	0.30	Neutral	0.04	Benign	0	Deleterious	15.11	Deleterious
p.Met1Tyr	VUS	-0.02	Neutral	0.31	Benign	-11.51	Pathogenic	0.37	Neutral	0.83	Possibly damaging	0	Deleterious		
p.Met1Cys	VUS	-0.01	Neutral	0.14	Benign	-10.28	Pathogenic	0.41	Neutral	0.95	Possibly damaging	0	Deleterious		
p.Met1Trp	VUS	-1.15	Neutral	0.19	Benign	-10.35	Pathogenic	0.37	Neutral	0.95	Possibly damaging	0	Deleterious		
p.Met1Phe	VUS	-0.27	Neutral	0.12	Benign	-10.79	Pathogenic	0.34	Neutral	0.83	Possibly damaging	0	Deleterious		
p.Glu2Asn	VUS	0.00	Neutral	0.16	Benign	-6.38	Benign	0.25	Neutral	0.21	Benign	0	Deleterious		
p.Glu2Lys	VUS	0.00	Neutral	0.18	Benign	-6.28	Benign	0.22	Neutral	0.08	Benign	0	Deleterious	18.01	Deleterious
p.Glu2Thr	VUS	0.00	Neutral	0.14	Benign	-5.22	Benign	0.26	Neutral	0.34	Benign	0	Deleterious		
p.Glu2Arg	VUS	0.00	Neutral	0.14	Benign	-5.17	Benign	0.29	Neutral	0.00	Benign	0	Deleterious		
p.Glu2Ser	VUS	-0.13	Neutral	0.11	Benign	-4.30	Benign	0.30	Neutral	0.34	Benign	0	Deleterious		
p.Glu2Ile	VUS	0.00	Neutral	0.25	Benign	-7.33	Benign	0.33	Neutral	0.65	Possibly damaging	0	Deleterious		
p.Glu2Met	VUS	-0.01	Neutral	0.36	Ambiguous	-7.24	Benign	0.33	Neutral	0.93	Possibly damaging	0	Deleterious		
p.Glu2His	VUS	0.00	Neutral	0.18	Benign	-6.40	Benign	0.28	Neutral	0.65	Possibly damaging	0	Deleterious		
p.Glu2Gln	VUS	0.00	Neutral	0.09	Benign	-5.39	Benign	0.17	Neutral	0.01	Benign	0	Deleterious	12.97	Neutral
p.Glu2Pro	VUS	0.00	Neutral	0.12	Benign	-4.12	Benign	0.32	Neutral	0.79	Possibly damaging	0	Deleterious		
p.Glu2Leu	VUS	0.00	Neutral	0.20	Benign	-5.40	Benign	0.32	Neutral	0.21	Benign	0	Deleterious		
p.Glu2Asp	VUS	0.00	Neutral	0.10	Benign	-4.35	Benign	0.17	Neutral	0.00	Benign	0	Deleterious	13.41	Neutral
p.Glu2Ala	VUS	-0.02	Neutral	0.09	Benign	-3.30	Benign	0.19	Neutral	0.16	Benign	0	Deleterious	18.03	Deleterious
p.Glu2Gly	VUS	0.00	Neutral	0.10	Benign	-4.46	Benign	0.24	Neutral	0.28	Benign	0	Deleterious	20.50	Deleterious
p.Glu2Val	VUS	0.00	Neutral	0.18	Benign	-5.70	Benign	0.21	Neutral	0.58	Possibly damaging	0	Deleterious	19.22	Deleterious
p.Glu2Tyr	VUS	0.00	Neutral	0.29	Benign	-7.99	Pathogenic	0.32	Neutral	0.00	Benign	0	Deleterious		
p.Glu2Cys	VUS	-0.05	Neutral	0.57	Pathogenic	-6.92	Benign	0.34	Neutral	0.98	Probably damaging	0	Deleterious		
p.Glu2Trp	VUS	0.00	Neutral	0.56	Ambiguous	-7.47	Benign	0.35	Neutral	0.93	Possibly damaging	0	Deleterious		
p.Glu2Phe	VUS	0.00	Neutral	0.33	Benign	-7.07	Benign	0.34	Neutral	0.48	Possibly damaging	0	Deleterious		
p.Pro3Asn	VUS	-7.64	Indeterminate	0.15	Benign	-6.32	Benign	0.28	Neutral	0.05	Benign	0	Deleterious		
p.Pro3Lys	VUS	-18.39	Indeterminate	0.14	Benign	-6.26	Benign	0.24	Neutral	0.01	Benign	0	Deleterious		
p.Pro3Thr	VUS	-10.80	Indeterminate	0.06	Benign	-4.43	Benign	0.18	Neutral	0.02	Benign	0	Deleterious	12.72	Neutral
p.Pro3Arg	VUS	-33.21	Indeterminate	0.08	Benign	-4.81	Benign	0.21	Neutral	0.00	Benign	0	Deleterious	0.00	Neutral
p.Pro3Ser	VUS	-22.54	Indeterminate	0.07	Benign	-3.83	Benign	0.16	Neutral	0.00	Benign	0	Deleterious	12.92	Neutral
p.Pro3Ile	VUS	-11.27	Indeterminate	0.17	Benign	-6.97	Benign	0.25	Neutral	0.03	Benign	0	Deleterious		
p.Pro3Met	VUS	-23.54	Indeterminate	0.21	Benign	-6.54	Benign	0.24	Neutral	0.32	Benign	0	Deleterious		
p.Pro3His	VUS	-5.11	Neutral	0.11	Benign	-6.20	Benign	0.18	Neutral	0.00	Benign	0	Deleterious		
p.Pro3Gln	VUS	-0.28	Neutral	0.08	Benign	-5.60	Benign	0.25	Neutral	0.00	Benign	0	Deleterious	0.01	Neutral
p.Pro3Leu	VUS	-15.02	Indeterminate	0.11	Benign	-5.31	Benign	0.14	Neutral	0.00	Benign	0	Deleterious	0.02	Neutral
p.Pro3Asp	VUS	-14.44	Indeterminate	0.12	Benign	-4.22	Benign	0.26	Neutral	0.05	Benign	0	Deleterious		
p.Pro3Glu	VUS	-4.70	Neutral	0.08	Benign	-3.98	Benign	0.28	Neutral	0.01	Benign	0	Deleterious		
p.Pro3Ala	VUS	-3.70	Neutral	0.04	Benign	-2.61	Benign	0.21	Neutral	0.01	Benign	0	Deleterious	10.50	Neutral
p.Pro3Gly	VUS	-53.15	Deleterious	0.10	Benign	-3.74	Benign	0.25	Neutral	0.03	Benign	0	Deleterious		
p.Pro3Val	VUS	-14.34	Indeterminate	0.10	Benign	-5.05	Benign	0.24	Neutral	0.03	Benign	0	Deleterious		
p.Pro3Tyr	VUS	-5.47	Neutral	0.25	Benign	-7.97	Pathogenic	0.26	Neutral	0.00	Benign	0	Deleterious		
p.Pro3Cys	VUS	-11.32	Indeterminate	0.33	Benign	-6.71	Benign	0.25	Neutral	0.75	Possibly damaging	0	Deleterious		
p.Pro3Trp	VUS	-31.79	Indeterminate	0.32	Benign	-7.79	Pathogenic	0.27	Neutral	0.49	Possibly damaging	0	Deleterious		
p.Pro3Phe	VUS	-12.58	Indeterminate	0.25	Benign	-7.36	Benign	0.26	Neutral	0.06	Benign	0	Deleterious		
p.Ala4Asn	VUS	-5.71	Neutral	0.20	Benign	-5.54	Benign	0.24	Neutral	0.19	Benign	0.05	Deleterious		
p.Ala4Lys	VUS	-1.31	Neutral	0.22	Benign	-6.17	Benign	0.26	Neutral	0.19	Benign	0.07	Tolerated		
p.Ala4Thr	VUS	-33.22	Indeterminate	0.09	Benign	-4.69	Benign	0.20	Neutral	0.15	Benign	0.11	Tolerated	15.19	Deleterious
p.Ala4Arg	VUS	-1.41	Neutral	0.14	Benign	-4.55	Benign	0.26	Neutral	0.45	Benign	0.05	Deleterious		
p.Ala4Ser	VUS	-53.15	Deleterious	0.09	Benign	-2.95	Benign	0.19	Neutral	0.07	Benign	0.5	Tolerated	10.21	Neutral
p.Ala4Ile	VUS	-0.18	Neutral	0.22	Benign	-6.32	Benign	0.24	Neutral	0.10	Benign	0.03	Deleterious		
p.Ala4Met	VUS	-7.81	Indeterminate	0.25	Benign	-6.25	Benign	0.23	Neutral	0.62	Possibly damaging	0.02	Deleterious		
p.Ala4His	VUS	-1.31	Neutral	0.20	Benign	-6.23	Benign	0.24	Neutral	0.83	Possibly damaging	0.02	Deleterious		
p.Ala4Gln	VUS	-0.32	Neutral	0.14	Benign	-5.70	Benign	0.24	Neutral	0.45	Benign	0.05	Deleterious		
p.Ala4Pro	VUS	-0.63	Neutral	0.08	Benign	-3.99	Benign	0.23	Neutral	0.79	Possibly damaging	0.07	Tolerated	15.45	Deleterious
p.Ala4Leu	VUS	-0.91	Neutral	0.14	Benign	-4.80	Benign	0.27	Neutral	0.05	Benign	0.04	Deleterious		
p.Ala4Asp	VUS	-3.20	Neutral	0.10	Benign	-4.74	Benign	0.23	Neutral	0.10	Benign	0.06	Tolerated		
p.Ala4Glu	VUS	-0.02	Neutral	0.10	Benign	-4.54	Benign	0.21	Neutral	0.01	Benign	0.08	Tolerated	12.91	Neutral
p.Ala4Gly	VUS	-6.58	Indeterminate	0.08	Benign	-3.58	Benign	0.21	Neutral	0.00	Benign	0.13	Tolerated	14.74	Neutral
p.Ala4Val	VUS	-0.11	Neutral	0.11	Benign	-4.86	Benign	0.13	Neutral	0.00	Benign	0.06	Tolerated	14.00	Neutral
p.Ala4Tyr	VUS	-0.67	Neutral	0.27	Benign	-7.04	Benign	0.27	Neutral	0.45	Benign	0.02	Deleterious		
p.Ala4Cys	VUS	-32.26	Indeterminate	0.29	Benign	-5.69	Benign	0.25	Neutral	0.95	Possibly damaging	0.02	Deleterious		
p.Ala4Trp	VUS	-2.02	Neutral	0.33	Benign	-7.03	Benign	0.23	Neutral	0.00	Benign	0.01	Deleterious		
p.Ala4Phe	VUS	-8.96	Indeterminate	0.20	Benign	-6.43	Benign	0.25	Neutral	0.45	Benign	0.02	Deleterious		
p.Ala5Asn	VUS	-0.01	Neutral	0.19	Benign	-5.78	Benign	0.27	Neutral	0.48	Possibly damaging	0.06	Tolerated		
p.Ala5Lys	VUS	0.00	Neutral	0.21	Benign	-5.65	Benign	0.27	Neutral	0.21	Benign	0.07	Tolerated		
p.Ala5Thr	VUS	-0.77	Neutral	0.09	Benign	-4.08	Benign	0.17	Neutral	0.28	Benign	0.08	Tolerated	9.47	Neutral
p.Ala5Arg	VUS	0.00	Neutral	0.13	Benign	-4.42	Benign	0.29	Neutral	0.65	Possibly damaging	0.05	Deleterious		
p.Ala5Ser	VUS	-33.22	Indeterminate	0.08	Benign	-3.80	Benign	0.18	Neutral	0.02	Benign	0.19	Tolerated	6.32	Neutral
p.Ala5Ile	VUS	0.00	Neutral	0.22	Benign	-6.07	Benign	0.28	Neutral	0.21	Benign	0.02	Deleterious		
p.Ala5Met	VUS	-0.27	Neutral	0.25	Benign	-4.19	Benign	0.29	Neutral	0.65	Possibly damaging	0.02	Deleterious		
p.Ala5His	VUS	-0.66	Neutral	0.20	Benign	-6.30	Benign	0.28	Neutral	0.93	Possibly damaging	0.02	Deleterious		
p.Ala5Gln	VUS	-0.83	Neutral	0.14	Benign	-5.76	Benign	0.27	Neutral	0.48	Possibly damaging	0.05	Deleterious		
p.Ala5Pro	VUS	-0.07	Neutral	0.07	Benign	-3.82	Benign	0.26	Neutral	0.74	Possibly damaging	0.09	Tolerated	9.87	Neutral
p.Ala5Leu	VUS	-9.21	Indeterminate	0.13	Benign	-4.31	Benign	0.28	Neutral	0.00	Benign	0.05	Deleterious		
p.Ala5Asp	VUS	-1.63	Neutral	0.11	Benign	-4.48	Benign	0.23	Neutral	0.00	Benign	0.06	Tolerated		
p.Ala5Glu	VUS	-6.48	Indeterminate	0.10	Benign	-4.50	Benign	0.23	Neutral	0.00	Benign	0.08	Tolerated	7.82	Neutral
p.Ala5Gly	VUS	-0.22	Neutral	0.07	Benign	-3.84	Benign	0.21	Neutral	0.29	Benign	0.48	Tolerated	9.20	Neutral
p.Ala5Val	VUS	-0.09	Neutral	0.12	Benign	-4.34	Benign	0.20	Neutral	0.08	Benign	0.06	Tolerated	9.18	Neutral
p.Ala5Tyr	VUS	-53.15	Deleterious	0.27	Benign	-7.06	Benign	0.31	Neutral	0.79	Possibly damaging	0.02	Deleterious		
p.Ala5Cys	VUS	-4.14	Neutral	0.29	Benign	-5.94	Benign	0.26	Neutral	0.98	Probably damaging	0.03	Deleterious		
p.Ala5Trp	VUS	-25.53	Indeterminate	0.30	Benign	-6.63	Benign	0.32	Neutral	0.98	Probably damaging	0.01	Deleterious		
p.Ala5Phe	VUS	-0.47	Neutral	0.19	Benign	-6.40	Benign	0.27	Neutral	0.65	Possibly damaging	0.02	Deleterious		
p.Gly6Asn	VUS	-0.09	Neutral	0.19	Benign	-6.09	Benign	0.23	Neutral	1.00	Probably damaging	0	Deleterious		
p.Gly6Lys	VUS	-2.25	Neutral	0.26	Benign	-5.94	Benign	0.23	Neutral	1.00	Probably damaging	0	Deleterious		
p.Gly6Thr	VUS	-4.22	Neutral	0.08	Benign	-4.52	Benign	0.25	Neutral	1.00	Probably damaging	0	Deleterious		
p.Gly6Arg	VUS	-53.15	Deleterious	0.16	Benign	-4.42	Benign	0.14	Neutral	1.00	Probably damaging	0	Deleterious	14.96	Neutral
p.Gly6Ser	VUS	-33.22	Indeterminate	0.09	Benign	-3.21	Benign	0.18	Neutral	1.00	Probably damaging	0	Deleterious		
p.Gly6Ile	VUS	-53.15	Deleterious	0.16	Benign	-6.38	Benign	0.22	Neutral	1.00	Probably damaging	0	Deleterious		
p.Gly6Met	VUS	-33.20	Indeterminate	0.30	Benign	-6.24	Benign	0.21	Neutral	1.00	Probably damaging	0	Deleterious		
p.Gly6His	VUS	0.00	Neutral	0.19	Benign	-6.10	Benign	0.24	Neutral	1.00	Probably damaging	0	Deleterious		
p.Gly6Gln	VUS	-0.08	Neutral	0.17	Benign	-5.44	Benign	0.25	Neutral	1.00	Probably damaging	0	Deleterious		
p.Gly6Pro	VUS	-20.27	Indeterminate	0.14	Benign	-3.48	Benign	0.25	Neutral	1.00	Probably damaging	0	Deleterious		
p.Gly6Leu	VUS	-0.87	Neutral	0.19	Benign	-4.70	Benign	0.21	Neutral	1.00	Probably damaging	0	Deleterious		
p.Gly6Asp	VUS	-0.53	Neutral	0.09	Benign	-3.84	Benign	0.16	Neutral	1.00	Probably damaging	0	Deleterious		

p.Gly6Glu	VUS	-0.72	Neutral	0.09	Benign	-3.51	Benign	0.13	Neutral	1.00	Probably damaging	0	Deleterious	6.13	Neutral
p.Gly6Ala	VUS	-0.20	Neutral	0.07	Benign	-3.05	Benign	0.11	Neutral	1.00	Probably damaging	0	Deleterious	0.10	Neutral
p.Gly6Val	VUS	-0.05	Neutral	0.10	Benign	-4.63	Benign	0.14	Neutral	1.00	Probably damaging	0	Deleterious	7.89	Neutral
p.Gly6Tyr	VUS	-0.77	Neutral	0.24	Benign	-7.14	Benign	0.23	Neutral	1.00	Probably damaging	0	Deleterious		
p.Gly6Cys	VUS	-13.23	Indeterminate	0.15	Benign	-5.88	Benign	0.18	Neutral	1.00	Probably damaging	0	Deleterious		
p.Gly6Trp	VUS	-3.24	Neutral	0.18	Benign	-6.33	Benign	0.21	Neutral	1.00	Probably damaging	0	Deleterious	20.50	Deleterious
p.Gly6Phe	VUS	-5.31	Neutral	0.27	Benign	-6.22	Benign	0.21	Neutral	1.00	Probably damaging	0	Deleterious		
p.Ser7Asn	VUS	-0.07	Neutral	0.16	Benign	-6.24	Benign	0.16	Neutral	0.91	Possibly damaging	0.41	Tolerated	10.26	Neutral
p.Ser7Lys	VUS	-0.18	Neutral	0.31	Benign	-6.64	Benign	0.27	Neutral	0.93	Possibly damaging	0.31	Tolerated		
p.Ser7Thr	VUS	-11.20	Indeterminate	0.09	Benign	-4.38	Benign	0.19	Neutral	0.81	Possibly damaging	0.38	Tolerated	10.06	Neutral
p.Ser7Arg	VUS	-0.03	Neutral	0.19	Benign	-4.15	Benign	0.16	Neutral	0.97	Probably damaging	0.19	Tolerated	11.49	Neutral
p.Ser7Ile	VUS	-1.33	Neutral	0.16	Benign	-6.23	Benign	0.25	Neutral	0.97	Probably damaging	0.09	Tolerated	15.55	Deleterious
p.Ser7Met	VUS	-0.71	Neutral	0.25	Benign	-6.72	Benign	0.30	Neutral	0.98	Probably damaging	0.06	Tolerated		
p.Ser7His	VUS	-3.32	Neutral	0.15	Benign	-5.85	Benign	0.28	Neutral	0.98	Probably damaging	0.1	Tolerated		
p.Ser7Gln	VUS	-0.38	Neutral	0.20	Benign	-5.54	Benign	0.25	Neutral	0.98	Probably damaging	0.2	Tolerated		
p.Ser7Pro	VUS	-0.71	Neutral	0.08	Benign	-2.24	Benign	0.22	Neutral	0.93	Possibly damaging	0.25	Tolerated		
p.Ser7Leu	VUS	-8.57	Indeterminate	0.15	Benign	-4.35	Benign	0.25	Neutral	0.93	Possibly damaging	0.15	Tolerated		
p.Ser7Asp	VUS	-0.96	Neutral	0.15	Benign	-4.70	Benign	0.24	Neutral	0.93	Possibly damaging	0.39	Tolerated		
p.Ser7Glu	VUS	-29.43	Indeterminate	0.16	Benign	-4.81	Benign	0.25	Neutral	0.93	Possibly damaging	0.31	Tolerated		
p.Ser7Ala	VUS	-19.51	Indeterminate	0.07	Benign	-3.25	Benign	0.20	Neutral	0.71	Possibly damaging	0.82	Tolerated		
p.Ser7Gly	VUS	-0.02	Neutral	0.07	Benign	-3.27	Benign	0.16	Neutral	0.81	Possibly damaging	0.72	Tolerated	9.07	Neutral
p.Ser7Val	VUS	-4.25	Neutral	0.15	Benign	-4.90	Benign	0.31	Neutral	0.93	Possibly damaging	0.18	Tolerated		
p.Ser7Tyr	VUS	-33.19	Indeterminate	0.14	Benign	-7.30	Benign	0.25	Neutral	0.98	Probably damaging	0.09	Tolerated		
p.Ser7Cys	VUS	-3.14	Neutral	0.11	Benign	-5.61	Benign	0.23	Neutral	0.99	Probably damaging	0.09	Tolerated	13.90	Neutral
p.Ser7Trp	VUS	-6.60	Indeterminate	0.14	Benign	-6.50	Benign	0.32	Neutral	0.99	Probably damaging	0.02	Deleterious		
p.Ser7Phe	VUS	-1.35	Neutral	0.15	Benign	-6.30	Benign	0.27	Neutral	0.98	Probably damaging	0.07	Tolerated		
p.Ser8Asn	VUS	0.00	Neutral	0.19	Benign	-5.65	Benign	0.15	Neutral	0.91	Possibly damaging	0	Deleterious	11.20	Neutral
p.Ser8Lys	VUS	-21.11	Indeterminate	0.35	Ambiguous	-6.11	Benign	0.26	Neutral	0.93	Possibly damaging	0	Deleterious		
p.Ser8Thr	VUS	-0.54	Neutral	0.09	Benign	-4.19	Benign	0.15	Neutral	0.81	Possibly damaging	0	Deleterious	9.91	Neutral
p.Ser8Arg	VUS	-0.04	Neutral	0.20	Benign	-4.19	Benign	0.21	Neutral	0.97	Probably damaging	0	Deleterious	13.46	Neutral
p.Ser8Ile	VUS	-0.01	Neutral	0.20	Benign	-5.85	Benign	0.21	Neutral	0.97	Probably damaging	0	Deleterious	19.43	Deleterious
p.Ser8Met	VUS	-0.01	Neutral	0.29	Benign	-6.03	Benign	0.28	Neutral	0.98	Probably damaging	0	Deleterious		
p.Ser8His	VUS	-0.01	Neutral	0.18	Benign	-6.03	Benign	0.28	Neutral	0.98	Probably damaging	0	Deleterious		
p.Ser8Gln	VUS	-0.02	Neutral	0.22	Benign	-5.47	Benign	0.26	Neutral	0.98	Probably damaging	0	Deleterious		
p.Ser8Pro	VUS	-0.80	Neutral	0.08	Benign	-3.55	Benign	0.20	Neutral	0.93	Possibly damaging	0	Deleterious		
p.Ser8Leu	VUS	-0.43	Neutral	0.17	Benign	-4.58	Benign	0.23	Neutral	0.93	Possibly damaging	0	Deleterious		
p.Ser8Asp	VUS	-0.03	Neutral	0.18	Benign	-4.38	Benign	0.26	Neutral	0.93	Possibly damaging	0	Deleterious		
p.Ser8Glu	VUS	0.00	Neutral	0.18	Benign	-4.18	Benign	0.26	Neutral	0.93	Possibly damaging	0	Deleterious		
p.Ser8Ala	VUS	-0.09	Neutral	0.07	Benign	-2.85	Benign	0.18	Neutral	0.71	Possibly damaging	0	Deleterious		
p.Ser8Gly	VUS	-0.42	Neutral	0.07	Benign	-3.03	Benign	0.16	Neutral	0.81	Possibly damaging	0	Deleterious	11.53	Neutral
p.Ser8Val	VUS	-0.03	Neutral	0.15	Benign	-4.45	Benign	0.27	Neutral	0.93	Possibly damaging	0	Deleterious		
p.Ser8Tyr	VUS	-0.01	Neutral	0.18	Benign	-6.89	Benign	0.25	Neutral	0.98	Probably damaging	0	Deleterious		
p.Ser8Cys	VUS	0.00	Neutral	0.12	Benign	-5.77	Benign	0.22	Neutral	0.99	Probably damaging	0	Deleterious	19.53	Deleterious
p.Ser8Trp	VUS	0.00	Neutral	0.17	Benign	-5.94	Benign	0.32	Neutral	0.99	Probably damaging	0	Deleterious		
p.Ser8Phe	VUS	-0.23	Neutral	0.18	Benign	-6.25	Benign	0.24	Neutral	0.98	Probably damaging	0	Deleterious		
p.Met9Asn	VUS	-0.21	Neutral	0.28	Benign	-4.02	Benign	0.33	Neutral	0.13	Benign	0.49	Tolerated		
p.Met9Lys	VUS	-0.23	Neutral	0.13	Benign	-3.57	Benign	0.35	Neutral	0.04	Benign	0.52	Tolerated	10.96	Neutral
p.Met9Thr	VUS	-0.27	Neutral	0.11	Benign	-1.57	Benign	0.17	Neutral	0.00	Benign	0.51	Tolerated	9.04	Neutral
p.Met9Arg	VUS	-0.07	Neutral	0.08	Benign	-1.77	Benign	0.33	Neutral	0.10	Benign	0.31	Tolerated	11.88	Neutral
p.Met9Ser	VUS	-1.02	Neutral	0.14	Benign	-1.00	Benign	0.33	Neutral	0.01	Benign	0.6	Tolerated		
p.Met9Ile	VUS	-0.29	Neutral	0.33	Benign	-3.66	Benign	0.27	Neutral	0.04	Benign	0.36	Tolerated	14.95	Neutral
p.Met9His	VUS	-0.70	Neutral	0.19	Benign	-3.54	Benign	0.33	Neutral	0.76	Possibly damaging	0.21	Tolerated		
p.Met9Gln	VUS	-0.37	Neutral	0.08	Benign	-2.68	Benign	0.30	Neutral	0.23	Benign	0.42	Tolerated		
p.Met9Pro	VUS	-0.63	Neutral	0.12	Benign	-0.65	Benign	0.35	Neutral	0.23	Benign	0.28	Tolerated		
p.Met9Leu	VUS	-0.01	Neutral	0.12	Benign	-1.65	Benign	0.21	Neutral	0.00	Benign	0.68	Tolerated	6.18	Neutral
p.Met9Asp	VUS	-0.13	Neutral	0.21	Benign	-2.24	Benign	0.33	Neutral	0.13	Benign	0.77	Tolerated		
p.Met9Glu	VUS	-0.05	Neutral	0.12	Benign	-1.93	Benign	0.35	Neutral	0.06	Benign	1	Tolerated		
p.Met9Ala	VUS	-0.12	Neutral	0.11	Benign	-0.64	Benign	0.32	Neutral	0.01	Benign	0.68	Tolerated		
p.Met9Gly	VUS	-0.35	Neutral	0.10	Benign	-1.14	Benign	0.28	Neutral	0.06	Benign	0.44	Tolerated		
p.Met9Val	VUS	-0.46	Neutral	0.07	Benign	-2.11	Benign	0.22	Neutral	0.00	Benign	0.43	Tolerated	9.64	Neutral
p.Met9Tyr	VUS	-0.02	Neutral	0.31	Benign	-4.70	Benign	0.34	Neutral	0.47	Possibly damaging	0.24	Tolerated		
p.Met9Cys	VUS	-0.13	Neutral	0.30	Benign	-3.72	Benign	0.34	Neutral	0.76	Possibly damaging	0.09	Tolerated		
p.Met9Trp	VUS	-0.13	Neutral	0.25	Benign	-3.88	Benign	0.37	Neutral	0.76	Possibly damaging	0.06	Tolerated		
p.Met9Phe	VUS	-0.23	Neutral	0.17	Benign	-4.09	Benign	0.33	Neutral	0.23	Benign	0.24	Tolerated		
p.Glu10Asn	VUS	-2.65	Neutral	0.33	Benign	-6.10	Benign	0.30	Neutral	0.26	Benign	0	Deleterious		
p.Glu10Lys	VUS	-33.21	Indeterminate	0.26	Benign	-5.90	Benign	0.25	Neutral	0.35	Benign	0	Deleterious	13.32	Neutral
p.Glu10Thr	VUS	-5.04	Neutral	0.22	Benign	-4.77	Benign	0.30	Neutral	0.59	Possibly damaging	0	Deleterious		
p.Glu10Arg	VUS	-0.85	Neutral	0.20	Benign	-4.48	Benign	0.25	Neutral	0.83	Possibly damaging	0	Deleterious		
p.Glu10Ser	VUS	-22.02	Indeterminate	0.21	Benign	-4.04	Benign	0.31	Neutral	0.41	Benign	0	Deleterious		
p.Glu10Ile	VUS	-13.01	Indeterminate	0.38	Ambiguous	-6.67	Benign	0.33	Neutral	0.94	Possibly damaging	0	Deleterious		
p.Glu10Met	VUS	-9.12	Indeterminate	0.50	Ambiguous	-6.58	Benign	0.36	Neutral	0.98	Probably damaging	0	Deleterious		
p.Glu10His	VUS	-32.21	Indeterminate	0.31	Benign	-5.89	Benign	0.31	Neutral	0.94	Possibly damaging	0	Deleterious		
p.Glu10Gln	VUS	-0.37	Neutral	0.12	Benign	-5.01	Benign	0.22	Neutral	0.52	Possibly damaging	0	Deleterious	11.32	Neutral
p.Glu10Pro	VUS	-0.83	Neutral	0.20	Benign	-3.49	Benign	0.35	Neutral	0.94	Possibly damaging	0	Deleterious		
p.Glu10Leu	VUS	-5.49	Neutral	0.30	Benign	-4.45	Benign	0.30	Neutral	0.83	Possibly damaging	0	Deleterious		
p.Glu10Asp	VUS	-2.12	Neutral	0.16	Benign	-3.82	Benign	0.14	Neutral	0.00	Benign	0	Deleterious	3.76	Neutral
p.Glu10Ala	VUS	-32.81	Indeterminate	0.12	Benign	-3.21	Benign	0.23	Neutral	0.35	Benign	0	Deleterious	11.44	Neutral
p.Glu10Gly	VUS	-1.38	Neutral	0.15	Benign	-2.92	Benign	0.21	Neutral	0.35	Benign	0	Deleterious	13.55	Neutral
p.Glu10Val	VUS	-1.13	Neutral	0.22	Benign	-4.69	Benign	0.26	Neutral	0.79	Possibly damaging	0	Deleterious	12.78	Neutral
p.Glu10Tyr	VUS	-2.56	Neutral	0.45	Ambiguous	-6.99	Benign	0.32	Neutral	0.94	Possibly damaging	0	Deleterious		
p.Glu10Cys	VUS	-1.13	Neutral	0.77	Pathogenic	-6.29	Benign	0.34	Neutral	0.98	Probably damaging	0	Deleterious		
p.Glu10Trp	VUS	-1.25	Neutral	0.76	Pathogenic	-6.63	Benign	0.36	Neutral	0.98	Probably damaging	0	Deleterious		
p.Glu10Phe	VUS	-2.65	Neutral	0.53	Ambiguous	-6.37	Benign	0.33	Neutral	0.94	Possibly damaging	0	Deleterious		
p.Pro11Asn	VUS	-1.32	Neutral	0.32	Benign	-6.47	Benign	0.27	Neutral	0.02	Benign	0.04	Deleterious		
p.Pro11Lys	VUS	-4.63	Neutral	0.28	Benign	-7.00	Benign	0.25	Neutral	0.12	Benign	0.03	Deleterious		
p.Pro11Thr	VUS	-8.84	Indeterminate	0.10	Benign	-4.72	Benign	0.36	Neutral	0.00	Benign	0.08	Tolerated	12.11	Neutral
p.Pro11Arg	VUS	-1.90	Neutral	0.14	Benign	-4.49	Benign	0.18	Neutral	0.26	Benign	0.02	Deleterious	15.68	Deleterious
p.Pro11Ser	VUS	-2.27	Neutral	0.13	Benign	-3.34	Benign	0.19	Neutral	0.00	Benign	0.39	Tolerated	13.98	Neutral
p.Pro11Ile	VUS	-1.45	Neutral	0.27	Benign	-6.46	Benign	0.29	Neutral	0.06	Benign	0.01	Deleterious		
p.Pro11Met	VUS	-0.32	Neutral	0.35	Ambiguous	-6.10	Benign	0.28	Neutral	0.15	Benign	0.01	Deleterious		
p.Pro11His	VUS	-1.60	Neutral	0.21	Benign	-6.18	Benign	0.21	Neutral	0.45	Benign	0.01	Deleterious	16.24	Deleterious
p.Pro11Gln	VUS	-0.33	Neutral	0.16	Benign	-5.73	Benign	0.28	Neutral	0.26	Benign	0.02	Deleterious		
p.Pro11Leu	VUS	-1.78	Neutral	0.17	Benign	-4.57	Benign	0.17	Neutral	0.00	Benign	0.02	Deleterious	16.66	Deleterious
p.Pro11Asp	VUS	-0.52	Neutral	0.29	Benign	-4.51	Benign	0.27	Neutral	0.01	Benign	0.03	Deleterious		
p.Pro11Glu	VUS	-0.68	Neutral	0.20	Benign	-4.43	Benign	0.28	Neutral	0.00	Benign	0.03	Deleterious		
p.Pro11Ala	VUS	-0.43	Neutral	0.07	Benign	-3.36	Benign	0.24	Neutral	0.00	Benign	0.12	Tolerated	8.49	Neutral
p.Pro11Gly	VUS	-1.89	Neutral	0.19	Benign	-3.65	Benign	0.27	Neutral	0.03	Benign	0.07	Tolerated		
p.Pro11Val	VUS	-0.96	Neutral	0.16	Benign	-4.78	Benign	0.27	Neutral	0.01	Benign	0.03	Deleterious		
p.Pro11Tyr	VUS	-2.83	Neutral	0.35	Ambiguous	-6.83	Benign	0.26	Neutral	0.49	Possibly damaging	0.01	Deleterious		
p.Pro11Cys	VUS	-0.76	Neutral	0.50	Ambiguous	-6.12	Benign	0.27	Neutral	0.99	Probably damaging	0.01	Deleterious		
p.Pro11Trp	VUS	-3.64	Neutral	0.51	Ambiguous	-6.73	Benign	0.28	Neutral	0.92	Possibly damaging	0	Deleterious		
p.Pro11Phe	VUS	-2.45	Neutral	0.38	Ambiguous	-6.30	Benign	0.28	Neutral	0.32	Benign	0.01	Deleterious		
p.Ser12Asn	VUS	-1.24	Neutral	0.22	Benign	-5.32	Benign	0.21	Neutral	0.01	Benign	0.19	Tolerated		
p.Ser12Lys	VUS	-0.08	Neutral	0.49	Ambiguous	-5.64	Benign	0.28	Neutral	0.10	Benign	0.2	Tolerated		
p.Ser12Thr	VUS	-0.10	Neutral	0.10	Benign	-3.60	Benign	0.12	Neutral	0.00	Benign	0.56	Tolerated	9.20	Neutral
p.Ser12Arg	VUS	-0.44	Neutral	0.36	Ambiguous	-4.09	Benign	0.24	Neutral	0.18	Benign	0.15	Tolerated		
p.Ser12Ile	VUS	-0.29	Neutral	0.37	Ambiguous	-6.38	Benign	0.23	Neutral	0.05	Benign	0.12	Tolerated		
p.Ser12Met	VUS	-0.04	Neutral	0.39	Ambiguous	-5.38	Benign	0.22	Neutral	0.28	Benign	0.08	Tolerated		
p.Ser12His	VUS	-2.76	Neutral	0.28	Benign	-5.49	Benign	0.29	Neutral	0.70	Possibly damaging	0.09	Tolerated		
p.Ser12Gln	VUS	-2.38	Neutral	0.33	Benign	-4.82	Benign	0.26	Neutral	0.44	Benign	0.14	Tolerated		
p.Ser12Pro	VUS	-0.05	Neutral	0.10	Benign	-3.18	Benign	0.27	Neutral	0.00	Benign	0.27	Tolerated	13.08	Neutral

p.Ser12Leu	VUS	-28.42	Indeterminate	0.23	Benign	-3.77	Benign	0.16	Neutral	0.00	Benign	0.19	Tolerated	17.06	Deleterious
p.Ser12Asp	VUS	-0.01	Neutral	0.26	Benign	-3.26	Benign	0.28	Neutral	0.06	Benign	0.17	Tolerated		
p.Ser12Glu	VUS	-1.21	Neutral	0.30	Benign	-3.82	Benign	0.28	Neutral	0.04	Benign	0.18	Tolerated		
p.Ser12Ala	VUS	-0.70	Neutral	0.08	Benign	-1.94	Benign	0.15	Neutral	0.00	Benign	1	Tolerated	8.47	Neutral
p.Ser12Gly	VUS	-0.16	Neutral	0.09	Benign	-3.33	Benign	0.16	Neutral	0.14	Benign	0.53	Tolerated		
p.Ser12Val	VUS	-4.85	Neutral	0.25	Benign	-4.02	Benign	0.21	Neutral	0.01	Benign	0.25	Tolerated		
p.Ser12Tyr	VUS	-0.13	Neutral	0.28	Benign	-6.41	Benign	0.24	Neutral	0.44	Benign	0.15	Tolerated		
p.Ser12Cys	VUS	-2.60	Neutral	0.15	Benign	-5.63	Benign	0.23	Neutral	0.39	Benign	0.12	Tolerated		
p.Ser12Trp	VUS	-3.60	Neutral	0.30	Benign	-6.43	Benign	0.24	Neutral	0.95	Possibly damaging	0.05	Deleterious	20.80	Deleterious
p.Ser12Phe	VUS	-1.39	Neutral	0.35	Ambiguous	-6.44	Benign	0.24	Neutral	0.28	Benign	0.13	Tolerated		
p.Ala13Asn	VUS	0.00	Neutral	0.44	Ambiguous	-6.94	Benign	0.29	Neutral	0.09	Benign	0.02	Deleterious		
p.Ala13Lys	VUS	-0.10	Neutral	0.62	Pathogenic	-7.44	Benign	0.49	Neutral	0.65	Possibly damaging	0.02	Deleterious		
p.Ala13Thr	VUS	-0.02	Neutral	0.17	Benign	-5.53	Benign	0.43	Neutral	0.33	Benign	0.04	Deleterious	23.30	Deleterious
p.Ala13Arg	VUS	0.00	Neutral	0.39	Ambiguous	-5.27	Benign	0.48	Neutral	0.93	Possibly damaging	0.02	Deleterious		
p.Ala13Ser	VUS	0.00	Neutral	0.13	Benign	-4.23	Benign	0.43	Neutral	0.03	Benign	0.11	Tolerated	21.70	Deleterious
p.Ala13Ile	VUS	-0.01	Neutral	0.50	Ambiguous	-8.08	Pathogenic	0.49	Neutral	0.93	Possibly damaging	0.01	Deleterious		
p.Ala13Met	VUS	-0.82	Neutral	0.55	Ambiguous	-7.42	Benign	0.45	Neutral	0.99	Probably damaging	0.01	Deleterious		
p.Ala13His	VUS	-0.17	Neutral	0.51	Ambiguous	-6.96	Benign	0.46	Neutral	0.99	Probably damaging	0.01	Deleterious		
p.Ala13Gln	VUS	-0.02	Neutral	0.39	Ambiguous	-5.95	Benign	0.47	Neutral	0.87	Possibly damaging	0.02	Deleterious		
p.Ala13Pro	VUS	0.00	Neutral	0.11	Benign	-4.31	Benign	0.53	Disease	0.73	Possibly damaging	0.05	Deleterious	23.60	Deleterious
p.Ala13Leu	VUS	-0.18	Neutral	0.35	Ambiguous	-6.06	Benign	0.33	Neutral	0.79	Possibly damaging	0.02	Deleterious		
p.Ala13Asp	VUS	-0.01	Neutral	0.37	Ambiguous	-4.88	Benign	0.33	Neutral	0.00	Benign	0.02	Deleterious	15.22	Deleterious
p.Ala13Glu	VUS	-0.01	Neutral	0.32	Benign	-4.65	Benign	0.46	Neutral	0.03	Benign	0.02	Deleterious		
p.Ala13Gly	VUS	0.00	Neutral	0.14	Benign	-2.03	Benign	0.26	Neutral	0.00	Benign	1	Tolerated	10.78	Neutral
p.Ala13Val	VUS	-0.05	Neutral	0.25	Benign	-5.39	Benign	0.28	Neutral	0.52	Possibly damaging	0.03	Deleterious	16.07	Deleterious
p.Ala13Tyr	VUS	-1.26	Neutral	0.58	Pathogenic	-8.64	Pathogenic	0.50	Neutral	0.98	Probably damaging	0.01	Deleterious		
p.Ala13Cys	VUS	-0.59	Neutral	0.54	Ambiguous	-6.73	Benign	0.44	Neutral	0.98	Probably damaging	0.02	Deleterious		
p.Ala13Trp	VUS	-0.39	Neutral	0.66	Pathogenic	-6.89	Benign	0.56	Disease	0.99	Probably damaging	0	Deleterious		
p.Ala13Phe	VUS	-0.31	Neutral	0.51	Ambiguous	-8.31	Pathogenic	0.50	Neutral	0.98	Probably damaging	0.01	Deleterious		
p.Asp14Asn	VUS	-3.70	Neutral	0.26	Benign	-6.07	Benign	0.45	Neutral	0.87	Possibly damaging	0.51	Tolerated	23.20	Deleterious
p.Asp14Lys	VUS	-2.50	Neutral	0.72	Pathogenic	-7.26	Benign	0.38	Neutral	0.97	Probably damaging	0.05	Deleterious		
p.Asp14Thr	VUS	-0.45	Neutral	0.43	Ambiguous	-5.05	Benign	0.55	Disease	0.90	Possibly damaging	0.03	Deleterious		
p.Asp14Arg	VUS	-15.07	Indeterminate	0.53	Ambiguous	-4.63	Benign	0.55	Disease	0.99	Probably damaging	0.02	Deleterious		
p.Asp14Ser	VUS	-0.40	Neutral	0.25	Benign	-3.92	Benign	0.50	Disease	0.94	Possibly damaging	0.06	Tolerated		
p.Asp14Ile	VUS	-31.69	Indeterminate	0.67	Pathogenic	-7.71	Pathogenic	0.55	Disease	0.99	Probably damaging	0.01	Deleterious		
p.Asp14Met	VUS	-21.59	Indeterminate	0.87	Pathogenic	-7.47	Benign	0.58	Disease	1.00	Probably damaging	0	Deleterious		
p.Asp14His	VUS	-0.67	Neutral	0.44	Ambiguous	-5.88	Benign	0.50	Disease	0.99	Probably damaging	0.02	Deleterious	24.40	Deleterious
p.Asp14Gln	VUS	-1.64	Neutral	0.50	Ambiguous	-5.12	Benign	0.45	Neutral	0.96	Probably damaging	0.04	Deleterious		
p.Asp14Pro	VUS	-33.22	Indeterminate	0.62	Pathogenic	-4.81	Benign	0.40	Neutral	0.73	Possibly damaging	0.03	Deleterious		
p.Asp14Leu	VUS	-11.64	Indeterminate	0.59	Pathogenic	-5.32	Benign	0.58	Disease	0.99	Probably damaging	0.01	Deleterious		
p.Asp14Glu	VUS	-1.99	Neutral	0.28	Benign	-3.12	Benign	0.23	Neutral	0.00	Benign	0.23	Tolerated	15.97	Deleterious
p.Asp14Ala	VUS	-10.01	Indeterminate	0.30	Benign	-3.02	Benign	0.30	Neutral	0.14	Benign	0.04	Deleterious	19.29	Deleterious
p.Asp14Gly	VUS	-0.82	Neutral	0.31	Benign	-3.87	Benign	0.51	Disease	0.96	Possibly damaging	0.08	Tolerated	22.80	Deleterious
p.Asp14Val	VUS	-31.29	Indeterminate	0.46	Ambiguous	-5.24	Benign	0.60	Disease	0.82	Possibly damaging	0.01	Deleterious	22.80	Deleterious
p.Asp14Tyr	VUS	-18.85	Indeterminate	0.41	Ambiguous	-8.06	Pathogenic	0.54	Disease	1.00	Probably damaging	0.01	Deleterious	24.80	Deleterious
p.Asp14Cys	VUS	-1.62	Neutral	0.78	Pathogenic	-6.51	Benign	0.56	Disease	1.00	Probably damaging	0.01	Deleterious		
p.Asp14Trp	VUS	-19.43	Indeterminate	0.88	Pathogenic	-7.13	Benign	0.59	Disease	1.00	Probably damaging	0	Deleterious		
p.Asp14Phe	VUS	-21.99	Indeterminate	0.76	Pathogenic	-7.76	Pathogenic	0.58	Disease	1.00	Probably damaging	0.01	Deleterious		
p.Trp15Asn	VUS	-4.97	Neutral	0.23	Benign	-3.04	Benign	0.27	Neutral	0.89	Possibly damaging	0.28	Tolerated		
p.Trp15Lys	VUS	-2.58	Neutral	0.23	Benign	-2.62	Benign	0.26	Neutral	0.01	Benign	1	Tolerated		
p.Trp15Thr	VUS	-11.33	Indeterminate	0.08	Benign	-1.18	Benign	0.30	Neutral	0.64	Possibly damaging	0.31	Tolerated		
p.Trp15Arg	VUS	-1.65	Neutral	0.11	Benign	0.64	Benign	0.12	Neutral	0.01	Benign	0.78	Tolerated	0.23	Neutral
p.Trp15Ser	VUS	-2.11	Neutral	0.06	Benign	0.17	Benign	0.19	Neutral	0.08	Benign	0.34	Tolerated	8.05	Neutral
p.Trp15Ile	VUS	-1.10	Neutral	0.16	Benign	-4.07	Benign	0.31	Neutral	0.49	Possibly damaging	0.12	Tolerated		
p.Trp15Met	VUS	-1.86	Neutral	0.26	Benign	-3.17	Benign	0.25	Neutral	0.44	Benign	0.08	Tolerated		
p.Trp15His	VUS	-23.94	Indeterminate	0.15	Benign	-2.56	Benign	0.33	Neutral	0.99	Probably damaging	0.16	Tolerated		
p.Trp15Gln	VUS	-4.61	Neutral	0.17	Benign	-0.15	Benign	0.22	Neutral	0.10	Benign	0.44	Tolerated		
p.Trp15Pro	VUS	-27.76	Indeterminate	0.31	Benign	1.37	Benign	0.32	Neutral	0.98	Probably damaging	0.18	Tolerated		
p.Trp15Leu	VUS	-0.52	Neutral	0.10	Benign	-0.60	Benign	0.18	Neutral	0.01	Benign	0.23	Tolerated	9.65	Neutral
p.Trp15Asp	VUS	-0.47	Neutral	0.11	Benign	0.24	Benign	0.28	Neutral	0.89	Possibly damaging	0.26	Tolerated		
p.Trp15Glu	VUS	-0.29	Neutral	0.09	Benign	0.93	Benign	0.26	Neutral	0.10	Benign	0.53	Tolerated		
p.Trp15Ala	VUS	-1.52	Neutral	0.04	Benign	1.49	Benign	0.31	Neutral	0.03	Benign	0.37	Tolerated		
p.Trp15Gly	VUS	-9.01	Indeterminate	0.05	Benign	-0.71	Benign	0.20	Neutral	0.57	Possibly damaging	0.23	Tolerated	1.39	Neutral
p.Trp15Val	VUS	-1.17	Neutral	0.09	Benign	-1.43	Benign	0.32	Neutral	0.64	Possibly damaging	0.18	Tolerated		
p.Trp15Tyr	VUS	-5.94	Indeterminate	0.24	Benign	-5.65	Benign	0.24	Neutral	0.74	Possibly damaging	0.12	Tolerated		
p.Trp15Cys	VUS	-5.76	Neutral	0.44	Ambiguous	-3.13	Benign	0.30	Neutral	0.99	Probably damaging	0.04	Deleterious	22.95	Deleterious
p.Trp15Phe	VUS	-0.61	Neutral	0.14	Benign	-4.96	Benign	0.25	Neutral	0.86	Possibly damaging	0.08	Tolerated		
p.Leu16Asn	VUS	-53.15	Deleterious	0.97	Pathogenic	-15.85	Pathogenic	0.80	Disease	1.00	Probably damaging	0	Deleterious		
p.Leu16Lys	VUS	-53.15	Deleterious	0.93	Pathogenic	-15.37	Pathogenic	0.80	Disease	0.99	Probably damaging	0	Deleterious		
p.Leu16Thr	VUS	-27.55	Indeterminate	0.83	Pathogenic	-12.66	Pathogenic	0.79	Disease	1.00	Probably damaging	0	Deleterious		
p.Leu16Arg	Pathogenic	-53.15	Deleterious	0.84	Pathogenic	-11.98	Pathogenic	0.92	Disease	1.00	Probably damaging	0	Deleterious	22.80	Deleterious
p.Leu16Ser	VUS	-53.15	Deleterious	0.97	Pathogenic	-12.29	Pathogenic	0.89	Disease	1.00	Probably damaging	0	Deleterious		
p.Leu16Ile	VUS	-6.96	Indeterminate	0.23	Benign	-9.94	Pathogenic	0.53	Disease	1.00	Probably damaging	0	Deleterious		
p.Leu16Met	VUS	-2.63	Neutral	0.60	Pathogenic	-9.00	Pathogenic	0.66	Disease	1.00	Probably damaging	0	Deleterious	20.30	Deleterious
p.Leu16His	VUS	-53.15	Deleterious	0.97	Pathogenic	-12.95	Pathogenic	0.86	Disease	1.00	Probably damaging	0	Deleterious		
p.Leu16Gln	VUS	-53.15	Deleterious	0.92	Pathogenic	-12.59	Pathogenic	0.74	Disease	1.00	Probably damaging	0	Deleterious	22.60	Deleterious
p.Leu16Pro	Pathogenic	-53.15	Deleterious	0.87	Pathogenic	-9.98	Pathogenic	0.94	Disease	1.00	Probably damaging	0	Deleterious	22.70	Deleterious
p.Leu16Asp	VUS	-53.15	Deleterious	0.99	Pathogenic	-14.19	Pathogenic	0.80	Disease	1.00	Probably damaging	0	Deleterious		
p.Leu16Glu	VUS	-53.15	Deleterious	0.94	Pathogenic	-13.68	Pathogenic	0.82	Disease	1.00	Probably damaging	0	Deleterious		
p.Leu16Ala	VUS	-53.15	Deleterious	0.85	Pathogenic	-11.52	Pathogenic	0.78	Disease	1.00	Probably damaging	0	Deleterious		
p.Leu16Gly	VUS	-53.15	Deleterious	0.95	Pathogenic	-12.24	Pathogenic	0.81	Disease	1.00	Probably damaging	0	Deleterious		
p.Leu16Val	VUS	-14.21	Indeterminate	0.29	Benign	-9.22	Pathogenic	0.61	Disease	0.98	Probably damaging	0	Deleterious	19.96	Deleterious
p.Leu16Tyr	VUS	-53.15	Deleterious	0.97	Pathogenic	-13.00	Pathogenic	0.75	Disease	0.92	Possibly damaging	0	Deleterious		
p.Leu16Cys	VUS	-16.70	Indeterminate	0.97	Pathogenic	-12.75	Pathogenic	0.74	Disease	1.00	Probably damaging	0	Deleterious		
p.Leu16Trp	VUS	-53.15	Deleterious	0.95	Pathogenic	-11.31	Pathogenic	0.81	Disease	0.78	Possibly damaging	0	Deleterious		
p.Leu16Phe	VUS	-4.61	Neutral	0.87	Pathogenic	-9.34	Pathogenic	0.67	Disease	0.99	Probably damaging	0	Deleterious		
p.Ala17Asn	VUS	-9.84	Indeterminate	0.69	Pathogenic	-9.33	Pathogenic	0.50	Neutral	0.74	Possibly damaging	0.31	Tolerated		
p.Ala17Lys	VUS	-53.15	Deleterious	0.93	Pathogenic	-14.13	Pathogenic	0.55	Disease	0.99	Probably damaging	0.34	Tolerated		
p.Ala17Thr	VUS	-4.30	Neutral	0.33	Benign	-5.99	Benign	0.20	Neutral	0.04	Benign	1	Tolerated	8.81	Neutral
p.Ala17Arg	VUS	-53.15	Deleterious	0.77	Pathogenic	-7.44	Benign	0.52	Disease	0.99	Probably damaging	0.26	Tolerated		
p.Ala17Ser	VUS	-1.96	Neutral	0.15	Benign	-1.74	Benign	0.20	Neutral	0.01	Benign	0.86	Tolerated	7.00	Neutral
p.Ala17Ile	VUS	-9.63	Indeterminate	0.73	Pathogenic	-11.13	Pathogenic	0.48	Neutral	0.99	Probably damaging	0.24	Tolerated		
p.Ala17Met	VUS	-53.15	Deleterious	0.64	Pathogenic	-10.49	Pathogenic	0.44	Neutral	1.00	Probably damaging	0.12	Tolerated		
p.Ala17His	VUS	-2.96	Neutral	0.64	Pathogenic	-9.79	Pathogenic	0.51	Disease	1.00	Probably damaging	0.3	Tolerated		
p.Ala17Gln	VUS	-53.15	Deleterious	0.64	Pathogenic	-10.72	Pathogenic	0.50	Neutral	1.00	Probably damaging	0.25	Tolerated		
p.Ala17Pro	VUS	-1.63	Neutral	0.70	Pathogenic	-8.27	Pathogenic	0.59	Disease	0.97	Probably damaging	0.22	Tolerated	18.05	Deleterious
p.Ala17Leu	VUS	-53.15	Deleterious	0.46	Ambiguous	-8.16	Pathogenic	0.47	Neutral	0.93	Possibly damaging	0.44	Tolerated		
p.Ala17Asp	VUS	-33.22	Indeterminate	0.83	Pathogenic	-11.49	Pathogenic	0.62	Disease	0.95	Possibly damaging	0.25	Tolerated	19.75	Deleterious
p.Ala17Glu	VUS	-53.15	Deleterious	0.72	Pathogenic	-11.65	Pathogenic	0.49	Neutral	0.97	Probably damaging	0.31	Tolerated		
p.Ala17Gly	VUS	-0.70	Neutral	0.23	Benign	-3.66	Benign	0.28	Neutral	0.02	Benign	0.28	Tolerated	12.08	Neutral
p.Ala17Val	VUS	-1.04	Neutral	0.40	Ambiguous	-8.46	Pathogenic	0.42	Neutral	0.88	Possibly damaging	0.37	Tolerated	18.67	Deleterious
p.Ala17Tyr	VUS	-53.15	Deleterious	0.71	Pathogenic	-8.94	Pathogenic	0.49	Neutral	1.00	Probably damaging	0.35	Tolerated		
p.Ala17Cys	VUS	-0.41	Neutral	0.44	Ambiguous	-0.72	Benign	0.29	Neutral	0.10	Benign	0.11	Tolerated		
p.Ala17Trp	VUS	-53.15	Deleterious	0.79	Pathogenic	-8.74	Pathogenic	0.54	Disease	1.00	Probably damaging	0.07	Tolerated		
p.Ala17Phe	VUS	-53.15	Deleterious	0.56	Ambiguous	-8.36	Pathogenic	0.47	Neutral	1.00	Probably damaging	0.26	Tolerated		
p.Thr18Asn	VUS	-33.21	Indeterminate	0.18	Benign	-4.86	Benign	0.19	Neutral	0.12	Benign	0.54	Tolerated		
p.Thr18Lys	VUS	-14.00	Indeterminate	0.24	Benign	-3.90	Benign	0.32	Neutral	0.76	Possibly damaging	0.86	Tolerated	11.22	Neutral
p.Thr18Arg	VUS	-12.80	Indeterminate	0.16	Benign	-1.73	Benign	0.25	Neutral	0.05	Benign	0.52	Tolerated	10.25	Neutral
p.Thr18Ser	VUS	-5.57	Neutral	0.14	Benign	-2.82	Benign	0.09	Neutral	0.01	Benign	0.88	Tolerated	0.39	Neutral
p.Thr18Ile	VUS	-28.46	Indeterminate	0.33	Benign	-5.32	Benign	0.25	Neutral	0.92	Possibly damaging	0.18	Tolerated		
p.Thr18Met	VUS	-20.84	Indeterminate	0.19	Benign	-5.46	Benign	0.25	Neutral	1.00	Probably damaging	0.1	Tolerated	18.95	Deleterious

p.Thr18His	VUS	-10.39	Indeterminate	0.27	Benign	-4.59	Benign	0.33	Neutral	0.99	Probably damaging	0.13	Tolerated		
p.Thr18Gln	VUS	-10.00	Indeterminate	0.23	Benign	-2.84	Benign	0.25	Neutral	0.84	Possibly damaging	0.55	Tolerated		
p.Thr18Pro	VUS	-53.15	Deleterious	0.32	Benign	-5.58	Benign	0.31	Neutral	1.00	Probably damaging	0.27	Tolerated	16.16	Deleterious
p.Thr18Leu	VUS	-29.34	Indeterminate	0.17	Benign	-3.26	Benign	0.31	Neutral	0.62	Possibly damaging	0.28	Tolerated		
p.Thr18Asp	VUS	-12.03	Indeterminate	0.35	Ambiguous	-0.56	Benign	0.30	Neutral	0.83	Possibly damaging	0.63	Tolerated		
p.Thr18Glu	VUS	-6.41	Indeterminate	0.27	Benign	0.00	Benign	0.34	Neutral	0.03	Benign	1	Tolerated		
p.Thr18Ala	VUS	-32.50	Indeterminate	0.09	Benign	-1.41	Benign	0.11	Neutral	0.00	Benign	0.71	Tolerated	4.84	Neutral
p.Thr18Gly	VUS	-10.65	Indeterminate	0.18	Benign	-3.12	Benign	0.34	Neutral	0.99	Probably damaging	0.44	Tolerated		
p.Thr18Val	VUS	-4.77	Neutral	0.20	Benign	-3.12	Benign	0.28	Neutral	0.54	Possibly damaging	0.27	Tolerated		
p.Thr18Tyr	VUS	-33.22	Indeterminate	0.37	Ambiguous	-7.10	Benign	0.32	Neutral	1.00	Probably damaging	0.05	Deleterious		
p.Thr18Cys	VUS	-1.62	Neutral	0.52	Ambiguous	-4.88	Benign	0.34	Neutral	1.00	Probably damaging	0.06	Tolerated		
p.Thr18Trp	VUS	-11.94	Indeterminate	0.56	Ambiguous	-5.04	Benign	0.36	Neutral	1.00	Probably damaging	0.02	Deleterious		
p.Thr18Phe	VUS	-16.79	Indeterminate	0.35	Ambiguous	-6.88	Benign	0.32	Neutral	0.98	Probably damaging	0.05	Deleterious		
p.Ala19Asn	VUS	0.00	Neutral	0.92	Pathogenic	-10.76	Pathogenic	0.66	Disease	1.00	Probably damaging	0	Deleterious		
p.Ala19Lys	VUS	-2.42	Neutral	0.97	Pathogenic	-11.61	Pathogenic	0.68	Disease	1.00	Probably damaging	0	Deleterious		
p.Ala19Thr	VUS	0.00	Neutral	0.62	Pathogenic	-8.56	Pathogenic	0.40	Neutral	1.00	Probably damaging	0	Deleterious	24.90	Deleterious
p.Ala19Arg	VUS	-0.43	Neutral	0.90	Pathogenic	-9.41	Pathogenic	0.69	Disease	1.00	Probably damaging	0	Deleterious		
p.Ala19Ser	VUS	0.00	Neutral	0.30	Benign	-7.07	Benign	0.63	Disease	0.99	Probably damaging	0	Deleterious	24.40	Deleterious
p.Ala19Ile	VUS	0.00	Neutral	0.93	Pathogenic	-10.54	Pathogenic	0.71	Disease	1.00	Probably damaging	0	Deleterious		
p.Ala19Met	VUS	0.00	Neutral	0.86	Pathogenic	-9.74	Pathogenic	0.68	Disease	1.00	Probably damaging	0	Deleterious		
p.Ala19His	VUS	0.00	Neutral	0.92	Pathogenic	-9.09	Pathogenic	0.70	Disease	1.00	Probably damaging	0	Deleterious		
p.Ala19Gln	VUS	0.00	Neutral	0.84	Pathogenic	-8.78	Pathogenic	0.70	Disease	1.00	Probably damaging	0	Deleterious		
p.Ala19Pro	VUS	-53.15	Deleterious	0.94	Pathogenic	-9.95	Pathogenic	0.81	Disease	1.00	Probably damaging	0	Deleterious	25.00	Deleterious
p.Ala19Leu	VUS	0.00	Neutral	0.71	Pathogenic	-6.76	Benign	0.70	Disease	1.00	Probably damaging	0	Deleterious		
p.Ala19Asp	VUS	-2.14	Neutral	0.94	Pathogenic	-9.76	Pathogenic	0.71	Disease	0.99	Probably damaging	0	Deleterious	24.40	Deleterious
p.Ala19Glu	VUS	0.00	Neutral	0.84	Pathogenic	-7.55	Pathogenic	0.75	Disease	1.00	Probably damaging	0	Deleterious		
p.Ala19Gly	VUS	0.00	Neutral	0.43	Ambiguous	-8.65	Pathogenic	0.44	Neutral	1.00	Probably damaging	0	Deleterious	24.30	Deleterious
p.Ala19Val	VUS	0.00	Neutral	0.65	Pathogenic	-7.64	Pathogenic	0.42	Neutral	1.00	Probably damaging	0	Deleterious	24.40	Deleterious
p.Ala19Tyr	VUS	0.00	Neutral	0.94	Pathogenic	-10.25	Pathogenic	0.70	Disease	1.00	Probably damaging	0	Deleterious		
p.Ala19Cys	VUS	-1.56	Neutral	0.76	Pathogenic	-8.25	Pathogenic	0.65	Disease	1.00	Probably damaging	0	Deleterious		
p.Ala19Trp	VUS	0.00	Neutral	0.98	Pathogenic	-10.40	Pathogenic	0.69	Disease	1.00	Probably damaging	0	Deleterious		
p.Ala19Phe	VUS	0.00	Neutral	0.93	Pathogenic	-9.57	Pathogenic	0.71	Disease	1.00	Probably damaging	0	Deleterious		
p.Ala20Asn	VUS	-53.15	Deleterious	0.99	Pathogenic	-20.00	Pathogenic	0.64	Disease	0.96	Probably damaging	0.02	Deleterious		
p.Ala20Lys	VUS	-53.15	Deleterious	0.99	Pathogenic	-21.46	Pathogenic	0.68	Disease	1.00	Probably damaging	0.02	Deleterious		
p.Ala20Thr	VUS	-21.91	Indeterminate	0.50	Ambiguous	-8.44	Pathogenic	0.62	Disease	1.00	Probably damaging	0.21	Tolerated	22.50	Deleterious
p.Ala20Arg	VUS	-53.15	Deleterious	0.97	Pathogenic	-18.12	Pathogenic	0.69	Disease	1.00	Probably damaging	0.06	Tolerated		
p.Ala20Ser	VUS	-24.89	Indeterminate	0.31	Benign	-7.00	Benign	0.37	Neutral	0.20	Benign	0.26	Tolerated	14.86	Neutral
p.Ala20Ile	VUS	-53.15	Deleterious	0.86	Pathogenic	-14.21	Pathogenic	0.69	Disease	1.00	Probably damaging	0.01	Deleterious		
p.Ala20Met	VUS	-53.15	Deleterious	0.92	Pathogenic	-17.29	Pathogenic	0.64	Disease	1.00	Probably damaging	0.01	Deleterious		
p.Ala20His	VUS	-53.15	Deleterious	0.99	Pathogenic	-21.47	Pathogenic	0.68	Disease	1.00	Probably damaging	0	Deleterious		
p.Ala20Gln	VUS	-53.15	Deleterious	0.96	Pathogenic	-19.57	Pathogenic	0.68	Disease	1.00	Probably damaging	0.01	Deleterious		
p.Ala20Pro	VUS	-53.15	Deleterious	0.95	Pathogenic	-15.03	Pathogenic	0.77	Disease	1.00	Probably damaging	0.02	Deleterious	22.70	Deleterious
p.Ala20Leu	VUS	-53.15	Deleterious	0.81	Pathogenic	-16.05	Pathogenic	0.68	Disease	0.99	Probably damaging	0.01	Deleterious		
p.Ala20Asp	VUS	-53.15	Deleterious	0.99	Pathogenic	-19.89	Pathogenic	0.73	Disease	1.00	Probably damaging	0.01	Deleterious		
p.Ala20Glu	VUS	-53.15	Deleterious	0.98	Pathogenic	-18.55	Pathogenic	0.72	Disease	1.00	Probably damaging	0.01	Deleterious	22.90	Deleterious
p.Ala20Gly	VUS	-53.15	Deleterious	0.54	Ambiguous	-11.28	Pathogenic	0.55	Disease	0.99	Probably damaging	0.03	Deleterious	22.90	Deleterious
p.Ala20Val	VUS	-9.53	Indeterminate	0.50	Ambiguous	-9.61	Pathogenic	0.62	Disease	0.85	Possibly damaging	0.02	Deleterious	20.60	Deleterious
p.Ala20Tyr	VUS	-53.15	Deleterious	0.99	Pathogenic	-19.34	Pathogenic	0.68	Disease	1.00	Probably damaging	0	Deleterious		
p.Ala20Cys	VUS	-5.52	Neutral	0.74	Pathogenic	-8.55	Pathogenic	0.59	Disease	1.00	Probably damaging	0.01	Deleterious		
p.Ala20Trp	VUS	-53.15	Deleterious	1.00	Pathogenic	-18.45	Pathogenic	0.69	Disease	1.00	Probably damaging	0	Deleterious		
p.Ala20Phe	VUS	-53.15	Deleterious	0.98	Pathogenic	-19.05	Pathogenic	0.69	Disease	1.00	Probably damaging	0	Deleterious		
p.Ala21Asn	VUS	-53.15	Deleterious	0.8048	Pathogenic	-13.28	Pathogenic	0.63	Disease	0.99	Probably damaging	0.02	Deleterious		
p.Ala21Lys	VUS	-53.15	Deleterious	0.8769	Pathogenic	-13.50	Pathogenic	0.64	Disease	1.00	Probably damaging	0.03	Deleterious		
p.Ala21Thr	VUS	-9.18	Indeterminate	0.4417	Ambiguous	-9.89	Pathogenic	0.53	Disease	1.00	Probably damaging	0.06	Tolerated	23.30	Deleterious
p.Ala21Arg	VUS	-53.15	Deleterious	0.6339	Pathogenic	-11.05	Pathogenic	0.61	Disease	1.00	Probably damaging	0.02	Deleterious		
p.Ala21Ser	VUS	-2.09	Neutral	0.1823	Benign	-6.29	Benign	0.52	Disease	0.52	Possibly damaging	0.22	Tolerated	21.10	Deleterious
p.Ala21Ile	VUS	-53.15	Deleterious	0.74	Pathogenic	-13.32	Pathogenic	0.64	Disease	1.00	Probably damaging	0.03	Deleterious		
p.Ala21Met	VUS	-53.15	Deleterious	0.6696	Pathogenic	-11.97	Pathogenic	0.63	Disease	1.00	Probably damaging	0.02	Deleterious		
p.Ala21His	VUS	-53.15	Deleterious	0.80	Pathogenic	-13.25	Pathogenic	0.63	Disease	1.00	Probably damaging	0.01	Deleterious		
p.Ala21Gln	VUS	-53.15	Deleterious	0.5501	Ambiguous	-11.63	Pathogenic	0.62	Disease	1.00	Probably damaging	0.02	Deleterious		
p.Ala21Pro	VUS	-53.15	Deleterious	0.9341	Pathogenic	-14.22	Pathogenic	0.67	Disease	1.00	Probably damaging	0.06	Tolerated	23.50	Deleterious
p.Ala21Leu	VUS	-5.31	Neutral	0.5239	Ambiguous	-11.90	Pathogenic	0.63	Disease	1.00	Probably damaging	0.18	Tolerated		
p.Ala21Asp	VUS	-53.15	Deleterious	0.91	Pathogenic	-11.71	Pathogenic	0.74	Disease	1.00	Probably damaging	0.02	Deleterious	25.20	Deleterious
p.Ala21Glu	VUS	-53.15	Deleterious	0.71	Pathogenic	-9.30	Pathogenic	0.67	Disease	1.00	Probably damaging	0.03	Deleterious		
p.Ala21Gly	VUS	-0.52	Neutral	0.25	Benign	-0.84	Benign	0.51	Disease	0.99	Probably damaging	0.13	Tolerated	24.60	Deleterious
p.Ala21Val	VUS	-1.47	Neutral	0.4395	Ambiguous	-10.01	Pathogenic	0.46	Neutral	1.00	Probably damaging	0.06	Tolerated	25.00	Deleterious
p.Ala21Tyr	VUS	-53.15	Deleterious	0.7762	Pathogenic	-11.79	Pathogenic	0.66	Disease	1.00	Probably damaging	0.01	Deleterious		
p.Ala21Cys	VUS	-0.74	Neutral	0.63	Pathogenic	-8.76	Pathogenic	0.59	Disease	1.00	Probably damaging	0.03	Deleterious		
p.Ala21Trp	VUS	-53.15	Deleterious	0.8406	Pathogenic	-11.16	Pathogenic	0.67	Disease	1.00	Probably damaging	0	Deleterious		
p.Ala21Phe	VUS	-53.15	Deleterious	0.70	Pathogenic	-12.11	Pathogenic	0.67	Disease	1.00	Probably damaging	0.01	Deleterious		
p.Arg22Asn	VUS	0.00	Neutral	0.5974	Pathogenic	-6.51	Benign	0.44	Neutral	0.99	Probably damaging	0.07	Tolerated		
p.Arg22Lys	VUS	-1.44	Neutral	0.2087	Benign	-6.50	Benign	0.16	Neutral	0.28	Benign	0.25	Tolerated		
p.Arg22Thr	VUS	-27.66	Indeterminate	0.3244	Benign	-7.75	Pathogenic	0.28	Neutral	0.77	Possibly damaging	0.09	Tolerated		
p.Arg22Ser	VUS	-23.87	Indeterminate	0.4527	Ambiguous	-6.49	Benign	0.36	Neutral	0.99	Probably damaging	0.23	Tolerated		
p.Arg22Ile	VUS	-0.98	Neutral	0.4942	Ambiguous	-11.18	Pathogenic	0.45	Neutral	1.00	Probably damaging	0.03	Deleterious		
p.Arg22Met	VUS	-8.42	Indeterminate	0.4456	Ambiguous	-9.14	Pathogenic	0.47	Neutral	0.98	Probably damaging	0.02	Deleterious		
p.Arg22His	VUS	0.00	Neutral	0.16	Benign	-4.40	Benign	0.32	Neutral	0.99	Probably damaging	0.04	Deleterious		
p.Arg22Gln	VUS	0.00	Neutral	0.1157	Benign	-5.42	Benign	0.16	Neutral	0.78	Possibly damaging	0.15	Tolerated	12.71	Neutral
p.Arg22Pro	VUS	-53.15	Deleterious	0.8905	Pathogenic	-11.55	Pathogenic	0.54	Disease	1.00	Probably damaging	0.05	Deleterious	20.40	Deleterious
p.Arg22Leu	VUS	-0.02	Neutral	0.2206	Benign	-6.54	Benign	0.22	Neutral	0.51	Possibly damaging	0.1	Tolerated	12.51	Neutral
p.Arg22Asp	VUS	-6.69	Indeterminate	0.6151	Pathogenic	-6.66	Benign	0.47	Neutral	1.00	Probably damaging	0.05	Deleterious		
p.Arg22Glu	VUS	0.00	Neutral	0.2995	Benign	-6.72	Benign	0.37	Neutral	0.84	Possibly damaging	0.11	Tolerated		
p.Arg22Ala	VUS	-0.01	Neutral	0.2965	Benign	-5.51	Benign	0.41	Neutral	0.95	Possibly damaging	0.22	Tolerated		
p.Arg22Gly	VUS	-53.15	Deleterious	0.2016	Benign	-6.85	Benign	0.25	Neutral	0.47	Possibly damaging	0.06	Tolerated	16.11	Deleterious
p.Arg22Val	VUS	-0.16	Neutral	0.4546	Ambiguous	-8.96	Pathogenic	0.45	Neutral	0.99	Probably damaging	0.05	Deleterious		
p.Arg22Tyr	VUS	-3.79	Neutral	0.4817	Ambiguous	-6.03	Benign	0.41	Neutral	0.94	Possibly damaging	0.03	Deleterious		
p.Arg22Cys	VUS	0.00	Neutral	0.2916	Benign	-6.74	Benign	0.27	Neutral	1.00	Probably damaging	0.01	Deleterious		
p.Arg22Trp	VUS	-9.64	Indeterminate	0.2423	Benign	-8.27	Pathogenic	0.53	Disease	1.00	Probably damaging	0.01	Deleterious	23.50	Deleterious
p.Arg22Phe	VUS	-32.64	Indeterminate	0.5572	Ambiguous	-7.96	Pathogenic	0.45	Neutral	0.89	Possibly damaging	0.02	Deleterious		
p.Gly23Asn	VUS	-21.58	Indeterminate	0.88	Pathogenic	-9.28	Pathogenic	0.66	Disease	1.00	Probably damaging	0.21	Tolerated		
p.Gly23Lys	VUS	-53.15	Deleterious	0.9761	Pathogenic	-13.72	Pathogenic	0.70	Disease	1.00	Probably damaging	0.01	Deleterious		
p.Gly23Thr	VUS	-53.15	Deleterious	0.9417	Pathogenic	-11.02	Pathogenic	0.64	Disease	1.00	Probably damaging	0.01	Deleterious		
p.Gly23Arg	Likely pathogenic	-53.15	Deleterious	0.9175	Pathogenic	-11.14	Pathogenic	0.49	Neutral	1.00	Probably damaging	0.01	Deleterious	27.20	Deleterious
p.Gly23Ser	Likely pathogenic	-53.15	Deleterious	0.6877	Pathogenic	-8.75	Pathogenic	0.67	Disease	0.99	Probably damaging	0.03	Deleterious	26.90	Deleterious
p.Gly23Ile	VUS	-53.15	Deleterious	0.9805	Pathogenic	-13.32	Pathogenic	0.71	Disease	1.00	Probably damaging	0	Deleterious		
p.Gly23Met	VUS	-53.15	Deleterious	0.9793	Pathogenic	-13.18	Pathogenic	0.71	Disease	1.00	Probably damaging	0	Deleterious		
p.Gly23His	VUS	-53.15	Deleterious	0.9519	Pathogenic	-12.10	Pathogenic	0.68	Disease	1.00	Probably damaging	0.01	Deleterious		
p.Gly23Gln	VUS	-53.15	Deleterious	0.9087	Pathogenic	-11.13	Pathogenic	0.65	Disease	1.00	Probably damaging	0.01	Deleterious		
p.Gly23Pro	VUS	-53.15	Deleterious	0.9957	Pathogenic	-12.38	Pathogenic	0.47	Neutral	1.00	Probably damaging	0.01	Deleterious		
p.Gly23Leu	VUS	-53.15	Deleterious	0.9462	Pathogenic	-11.12	Pathogenic	0.70	Disease	1.00	Probably damaging	0	Deleterious		
p.Gly23Asp	Pathogenic	-53.15	Deleterious	0.8537	Pathogenic	-9.16	Pathogenic	0.83	Disease	1.00	Probably damaging	0.04	Deleterious	27.70	Deleterious
p.Gly23Glu	VUS	-53.15	Deleterious	0.9158	Pathogenic	-9.00	Pathogenic	0.78	Disease	1.00	Probably damaging	0.01	Deleterious		
p.Gly23Ala	VUS	-1.29	Neutral	0.7359	Pathogenic	-7.21	Benign	0.65	Disease	1.00	Probably damaging	0.02	Deleterious	26.10	Deleterious
p.Gly23Val	VUS	-53.15	Deleterious	0.955	Pathogenic	-10.22	Pathogenic	0.81	Disease	1.00	Probably damaging	0	Deleterious	27.30	Deleterious
p.Gly23Tyr	VUS	-53.15	Deleterious	0.9386	Pathogenic	-15.29	Pathogenic	0.71	Disease	1.00	Probably damaging	0	Deleterious		
p.Gly23Cys	VUS	-53.15	Deleterious	0.8139	Pathogenic	-9.87	Pathogenic	0.84	Disease	1.00	Probably damaging	0	Deleterious	28.10	Deleterious
p.Gly23Trp	VUS	-53.15	Deleterious	0.9369	Pathogenic	-13.94	Pathogenic	0.76	Disease	1.00	Probably damaging	0	Deleterious		
p.Gly23Phe	VUS	-53.15	Deleterious	0.9795	Pathogenic	-14.73	Pathogenic	0.73	Disease	1.00	Probably damaging	0	Deleterious		
p.Arg24Asn	VUS	-0.62	Neutral	0.4681	Ambiguous	-4.43	Benign	0.22	Neutral	0.85	Possibly damaging	0.43	Tolerated		
p.Arg24Lys	VUS	-1.72	Neutral	0.2416	Benign	-5.53	Benign	0.23	Neutral	0.62	Possibly damaging	1	Tolerated		
p.Arg24Thr	VUS	-3.98	Neutral	0.4732	Ambiguous	-5.85	Benign	0.39	Neutral	0.99	Probably damaging	0.42	Tolerated		

p.Arg24Ser	VUS	-0.21	Neutral	0.4235	Ambiguous	-3.88	Benign	0.38	Neutral	1.00	Probably damaging	0.49	Tolerated		
p.Arg24Ile	VUS	-0.34	Neutral	0.6604	Pathogenic	-9.38	Pathogenic	0.48	Neutral	1.00	Probably damaging	0.19	Tolerated		
p.Arg24Met	VUS	-0.60	Neutral	0.5378	Ambiguous	-8.07	Pathogenic	0.46	Neutral	0.68	Possibly damaging	0.11	Tolerated		
p.Arg24His	VUS	-0.01	Neutral	0.1286	Benign	-5.36	Benign	0.11	Neutral	0.99	Probably damaging	0.27	Tolerated		
p.Arg24Gln	VUS	-16.21	Indeterminate	0.12	Benign	-4.47	Benign	0.08	Neutral	0.73	Possibly damaging	0.61	Tolerated	18.45	Deleterious
p.Arg24Pro	Pathogenic	-53.15	Deleterious	0.9136	Pathogenic	-8.08	Pathogenic	0.63	Disease	1.00	Probably damaging	0.24	Tolerated	22.80	Deleterious
p.Arg24Leu	VUS	-1.07	Neutral	0.2995	Benign	-5.72	Benign	0.13	Neutral	1.00	Probably damaging	0.35	Tolerated	19.08	Deleterious
p.Arg24Asp	VUS	0.00	Neutral	0.4363	Ambiguous	-1.34	Benign	0.19	Neutral	0.79	Possibly damaging	0.64	Tolerated		
p.Arg24Glu	VUS	-1.01	Neutral	0.3064	Benign	-2.84	Benign	0.20	Neutral	0.80	Possibly damaging	0.8	Tolerated		
p.Arg24Ala	VUS	-0.03	Neutral	0.3784	Ambiguous	-3.76	Benign	0.39	Neutral	0.99	Probably damaging	0.53	Tolerated		
p.Arg24Gly	VUS	-2.96	Neutral	0.2927	Benign	-5.24	Benign	0.14	Neutral	1.00	Probably damaging	0.33	Tolerated	23.00	Deleterious
p.Arg24Val	VUS	-0.12	Neutral	0.5914	Pathogenic	-6.67	Benign	0.48	Neutral	1.00	Probably damaging	0.26	Tolerated		
p.Arg24Tyr	VUS	-1.12	Neutral	0.5087	Ambiguous	-8.91	Pathogenic	0.38	Neutral	0.76	Possibly damaging	0.24	Tolerated		
p.Arg24Cys	VUS	-0.09	Neutral	0.2899	Benign	-5.58	Benign	0.17	Neutral	1.00	Probably damaging	0.07	Tolerated		
p.Arg24Trp	VUS	-0.43	Neutral	0.2888	Benign	-8.64	Pathogenic	0.19	Neutral	0.32	Benign	0.06	Tolerated	22.70	Deleterious
p.Arg24Phe	VUS	-0.09	Neutral	0.6672	Pathogenic	-9.23	Pathogenic	0.46	Neutral	0.76	Possibly damaging	0.17	Tolerated		
p.Val25Asn	VUS	-4.24	Neutral	0.4525	Ambiguous	-10.67	Pathogenic	0.30	Neutral	0.84	Possibly damaging	0.02	Deleterious		
p.Val25Lys	VUS	-17.81	Indeterminate	0.4628	Ambiguous	-10.72	Pathogenic	0.29	Neutral	0.12	Benign	0.03	Deleterious		
p.Val25Thr	VUS	-1.42	Neutral	0.1379	Benign	-5.74	Benign	0.16	Neutral	0.04	Benign	0.32	Tolerated		
p.Val25Arg	VUS	-3.28	Neutral	0.3039	Benign	-8.25	Pathogenic	0.18	Neutral	0.38	Benign	0.02	Deleterious		
p.Val25Ser	VUS	-0.43	Neutral	0.2142	Benign	-7.24	Benign	0.27	Neutral	0.80	Possibly damaging	0.03	Deleterious		
p.Val25Ile	VUS	-0.50	Neutral	0.1369	Benign	-7.02	Benign	0.13	Neutral	0.01	Benign	0.39	Tolerated	14.12	Neutral
p.Val25Met	VUS	-21.03	Indeterminate	0.3185	Benign	-7.60	Pathogenic	0.16	Neutral	0.60	Possibly damaging	0.05	Deleterious		
p.Val25His	VUS	-0.26	Neutral	0.4947	Ambiguous	-7.06	Benign	0.20	Neutral	0.90	Possibly damaging	0.01	Deleterious		
p.Val25Gln	VUS	-0.52	Neutral	0.3048	Benign	-8.28	Pathogenic	0.27	Neutral	0.41	Benign	0.03	Deleterious		
p.Val25Pro	VUS	0.00	Neutral	0.2132	Benign	-6.08	Benign	0.29	Neutral	0.84	Possibly damaging	0.04	Deleterious		
p.Val25Leu	VUS	-0.06	Neutral	0.2415	Benign	-3.55	Benign	0.12	Neutral	0.00	Benign	0.33	Tolerated	12.09	Neutral
p.Val25Asp	VUS	-17.55	Indeterminate	0.2921	Benign	-6.73	Benign	0.33	Neutral	0.96	Probably damaging	0.01	Deleterious		
p.Val25Glu	VUS	-0.18	Neutral	0.1475	Benign	-3.45	Benign	0.16	Neutral	0.03	Benign	0.08	Tolerated	4.46	Neutral
p.Val25Ala	VUS	-53.15	Deleterious	0.10	Benign	-4.13	Benign	0.06	Neutral	0.01	Benign	0.38	Tolerated	0.34	Neutral
p.Val25Gly	VUS	-19.94	Indeterminate	0.1571	Benign	-7.74	Pathogenic	0.15	Neutral	0.91	Possibly damaging	0	Deleterious	5.28	Neutral
p.Val25Tyr	VUS	-33.22	Indeterminate	0.5066	Ambiguous	-6.86	Benign	0.27	Neutral	0.81	Possibly damaging	0.01	Deleterious		
p.Val25Cys	VUS	0.00	Neutral	0.6914	Pathogenic	-6.54	Benign	0.19	Neutral	0.93	Possibly damaging	0.04	Deleterious		
p.Val25Trp	VUS	-2.22	Neutral	0.6234	Pathogenic	-7.79	Pathogenic	0.29	Neutral	0.99	Probably damaging	0	Deleterious		
p.Val25Phe	VUS	-3.13	Neutral	0.2408	Benign	-7.96	Pathogenic	0.25	Neutral	0.68	Possibly damaging	0.03	Deleterious		
p.Glu26Asn	VUS	-1.41	Neutral	0.3554	Ambiguous	-8.72	Pathogenic	0.27	Neutral	0.05	Benign	0.02	Deleterious		
p.Glu26Lys	VUS	-0.44	Neutral	0.3023	Benign	-7.86	Pathogenic	0.19	Neutral	0.01	Benign	0.04	Deleterious	14.84	Neutral
p.Glu26Thr	VUS	-9.37	Indeterminate	0.2484	Benign	-6.86	Benign	0.29	Neutral	0.13	Benign	0.01	Deleterious		
p.Glu26Arg	VUS	-0.10	Neutral	0.2163	Benign	-5.10	Benign	0.23	Neutral	0.00	Benign	0.02	Deleterious		
p.Glu26Ser	VUS	-27.38	Indeterminate	0.2275	Benign	-5.96	Benign	0.31	Neutral	0.01	Benign	0.02	Deleterious		
p.Glu26Ile	VUS	-0.52	Neutral	0.5949	Pathogenic	-10.16	Pathogenic	0.33	Neutral	0.11	Benign	0	Deleterious		
p.Glu26Met	VUS	-0.27	Neutral	0.6984	Pathogenic	-9.89	Pathogenic	0.34	Neutral	0.02	Benign	0	Deleterious		
p.Glu26His	VUS	-4.15	Neutral	0.3936	Ambiguous	-6.68	Benign	0.22	Neutral	0.00	Benign	0.21	Tolerated		
p.Glu26Gln	VUS	-2.58	Neutral	0.1534	Benign	-5.86	Benign	0.11	Neutral	0.00	Benign	0.18	Tolerated	11.94	Neutral
p.Glu26Pro	VUS	-0.39	Neutral	0.3451	Ambiguous	-5.81	Benign	0.37	Neutral	0.21	Benign	0.02	Deleterious		
p.Glu26Leu	VUS	-0.53	Neutral	0.4372	Ambiguous	-6.92	Benign	0.34	Neutral	0.02	Benign	0.01	Deleterious		
p.Glu26Asp	VUS	-3.00	Neutral	0.1188	Benign	-3.77	Benign	0.11	Neutral	0.00	Benign	0.08	Tolerated	8.36	Neutral
p.Glu26Ala	VUS	-1.96	Neutral	0.1106	Benign	-4.01	Benign	0.12	Neutral	0.00	Benign	0.03	Deleterious	14.91	Neutral
p.Glu26Gly	VUS	-4.39	Neutral	0.1345	Benign	-5.02	Benign	0.21	Neutral	0.23	Benign	0.01	Deleterious	19.55	Deleterious
p.Glu26Val	VUS	-0.20	Neutral	0.3096	Benign	-6.90	Benign	0.28	Neutral	0.01	Benign	0.01	Deleterious	17.11	Deleterious
p.Glu26Tyr	VUS	-5.42	Neutral	0.7028	Pathogenic	-9.93	Pathogenic	0.32	Neutral	0.26	Benign	0	Deleterious		
p.Glu26Cys	VUS	-0.02	Neutral	0.8412	Pathogenic	-8.04	Pathogenic	0.33	Neutral	1.00	Probably damaging	0	Deleterious		
p.Glu26Trp	VUS	-0.96	Neutral	0.8423	Pathogenic	-8.77	Pathogenic	0.37	Neutral	0.89	Possibly damaging	0	Deleterious		
p.Glu26Phe	VUS	-3.59	Neutral	0.8086	Pathogenic	-10.05	Pathogenic	0.33	Neutral	0.21	Benign	0	Deleterious		
p.Glu27Asn	VUS	-15.33	Indeterminate	0.8108	Pathogenic	-8.82	Pathogenic	0.37	Neutral	0.97	Probably damaging	0.38	Tolerated		
p.Glu27Lys	VUS	-0.27	Neutral	0.6297	Pathogenic	-7.32	Benign	0.18	Neutral	0.66	Possibly damaging	0.74	Tolerated	22.50	Deleterious
p.Glu27Thr	VUS	-27.81	Indeterminate	0.582	Pathogenic	-5.89	Benign	0.29	Neutral	0.25	Benign	0.47	Tolerated		
p.Glu27Arg	VUS	-6.08	Indeterminate	0.5915	Pathogenic	-4.60	Benign	0.24	Neutral	0.90	Possibly damaging	0.4	Tolerated		
p.Glu27Ser	VUS	-4.04	Neutral	0.6781	Pathogenic	-6.61	Benign	0.42	Neutral	0.91	Possibly damaging	0.56	Tolerated		
p.Glu27Ile	VUS	-2.89	Neutral	0.7775	Pathogenic	-8.88	Pathogenic	0.44	Neutral	0.68	Possibly damaging	0.14	Tolerated		
p.Glu27Met	VUS	-2.28	Neutral	0.8549	Pathogenic	-8.51	Pathogenic	0.35	Neutral	0.74	Possibly damaging	0.13	Tolerated		
p.Glu27His	VUS	-4.17	Neutral	0.7579	Pathogenic	-6.22	Benign	0.40	Neutral	0.99	Probably damaging	0.1	Tolerated		
p.Glu27Gln	VUS	-3.17	Neutral	0.3021	Benign	-4.07	Benign	0.13	Neutral	0.11	Benign	0.53	Tolerated	21.00	Deleterious
p.Glu27Pro	VUS	-7.45	Indeterminate	0.9	Pathogenic	-6.85	Benign	0.47	Neutral	0.95	Possibly damaging	0.21	Tolerated		
p.Glu27Leu	VUS	-2.14	Neutral	0.777	Pathogenic	-4.75	Benign	0.31	Neutral	0.01	Benign	0.23	Tolerated		
p.Glu27Asp	VUS	-25.59	Indeterminate	0.3212	Benign	-4.41	Benign	0.12	Neutral	0.01	Benign	0.51	Tolerated	21.80	Deleterious
p.Glu27Ala	VUS	-3.09	Neutral	0.2891	Benign	-3.86	Benign	0.21	Neutral	0.07	Benign	0.6	Tolerated	21.30	Deleterious
p.Glu27Gly	VUS	-17.81	Indeterminate	0.6044	Pathogenic	-6.56	Benign	0.24	Neutral	0.98	Probably damaging	0.29	Tolerated	23.10	Deleterious
p.Glu27Val	VUS	-0.71	Neutral	0.5387	Ambiguous	-5.68	Benign	0.43	Neutral	0.02	Benign	0.3	Tolerated	22.20	Deleterious
p.Glu27Tyr	VUS	-53.15	Deleterious	0.9134	Pathogenic	-10.09	Pathogenic	0.45	Neutral	0.98	Probably damaging	0.03	Deleterious		
p.Glu27Cys	VUS	-18.51	Indeterminate	0.9673	Pathogenic	-8.40	Pathogenic	0.44	Neutral	1.00	Probably damaging	0.04	Deleterious		
p.Glu27Trp	VUS	-0.31	Neutral	0.9791	Pathogenic	-9.54	Pathogenic	0.49	Neutral	1.00	Probably damaging	0.01	Deleterious		
p.Glu27Phe	VUS	-0.20	Neutral	0.9594	Pathogenic	-10.44	Pathogenic	0.45	Neutral	0.90	Possibly damaging	0.04	Deleterious		
p.Val28Asn	VUS	-53.15	Deleterious	0.9413	Pathogenic	-17.77	Pathogenic	0.75	Disease	1.00	Probably damaging	0	Deleterious		
p.Val28Lys	VUS	-53.15	Deleterious	0.9711	Pathogenic	-19.40	Pathogenic	0.78	Disease	1.00	Probably damaging	0	Deleterious		
p.Val28Thr	VUS	-0.16	Neutral	0.5482	Ambiguous	-7.25	Benign	0.66	Disease	1.00	Probably damaging	0.01	Deleterious		
p.Val28Arg	VUS	-53.15	Deleterious	0.9327	Pathogenic	-15.40	Pathogenic	0.76	Disease	1.00	Probably damaging	0	Deleterious		
p.Val28Ser	VUS	-1.43	Neutral	0.8134	Pathogenic	-10.22	Pathogenic	0.70	Disease	1.00	Probably damaging	0	Deleterious		
p.Val28Ile	VUS	-0.17	Neutral	0.2771	Benign	-8.20	Pathogenic	0.44	Neutral	1.00	Probably damaging	0.05	Deleterious		
p.Val28Met	VUS	-0.29	Neutral	0.7568	Pathogenic	-9.48	Pathogenic	0.62	Disease	1.00	Probably damaging	0.01	Deleterious	31.00	Deleterious
p.Val28His	VUS	-53.15	Deleterious	0.9896	Pathogenic	-16.15	Pathogenic	0.74	Disease	1.00	Probably damaging	0	Deleterious		
p.Val28Gln	VUS	-53.15	Deleterious	0.9319	Pathogenic	-15.73	Pathogenic	0.76	Disease	1.00	Probably damaging	0	Deleterious		
p.Val28Pro	VUS	-53.15	Deleterious	0.9875	Pathogenic	-12.89	Pathogenic	0.74	Disease	1.00	Probably damaging	0	Deleterious		
p.Val28Leu	VUS	0.00	Neutral	0.7196	Pathogenic	-7.49	Benign	0.43	Neutral	1.00	Probably damaging	0.2	Tolerated	25.95	Deleterious
p.Val28Asp	VUS	-53.15	Deleterious	0.9632	Pathogenic	-15.94	Pathogenic	0.81	Disease	1.00	Probably damaging	0	Deleterious		
p.Val28Glu	VUS	-53.15	Deleterious	0.9105	Pathogenic	-14.49	Pathogenic	0.81	Disease	1.00	Probably damaging	0	Deleterious	32.00	Deleterious
p.Val28Ala	VUS	-0.04	Neutral	0.4679	Ambiguous	-5.32	Benign	0.56	Disease	1.00	Probably damaging	0.01	Deleterious	29.60	Deleterious
p.Val28Gly	VUS	-20.39	Indeterminate	0.57	Pathogenic	-11.97	Pathogenic	0.76	Disease	1.00	Probably damaging	0	Deleterious	32.00	Deleterious
p.Val28Tyr	VUS	-53.15	Deleterious	0.9801	Pathogenic	-16.38	Pathogenic	0.75	Disease	1.00	Probably damaging	0	Deleterious		
p.Val28Cys	VUS	-0.11	Neutral	0.9384	Pathogenic	-5.74	Benign	0.68	Disease	1.00	Probably damaging	0	Deleterious		
p.Val28Trp	VUS	-53.15	Deleterious	0.9963	Pathogenic	-15.93	Pathogenic	0.71	Disease	1.00	Probably damaging	0	Deleterious		
p.Val28Phe	VUS	-53.15	Deleterious	0.8948	Pathogenic	-13.19	Pathogenic	0.77	Disease	1.00	Probably damaging	0.01	Deleterious		
p.Arg29Asn	VUS	-4.64	Neutral	0.7626	Pathogenic	-10.66	Pathogenic	0.49	Neutral	0.98	Probably damaging	0.03	Deleterious		
p.Arg29Lys	VUS	-0.17	Neutral	0.191	Benign	-7.10	Benign	0.20	Neutral	0.15	Benign	0.3	Tolerated		
p.Arg29Thr	VUS	-12.09	Indeterminate	0.2316	Benign	-6.41	Benign	0.35	Neutral	0.55	Possibly damaging	0.26	Tolerated		
p.Arg29Ser	VUS	-0.79	Neutral	0.4961	Ambiguous	-5.40	Benign	0.46	Neutral	0.99	Probably damaging	0.03	Deleterious		
p.Arg29Ile	VUS	-21.20	Indeterminate	0.335	Benign	-10.02	Pathogenic	0.48	Neutral	1.00	Probably damaging	0.01	Deleterious		
p.Arg29Met	VUS	-1.63	Neutral	0.4175	Ambiguous	-9.57	Pathogenic	0.47	Neutral	1.00	Probably damaging	0.01	Deleterious		
p.Arg29His	VUS	-3.49	Neutral	0.1851	Benign	-8.02	Pathogenic	0.44	Neutral	0.98	Probably damaging	0.02	Deleterious		
p.Arg29Gln	VUS	-0.28	Neutral	0.1091	Benign	-5.72	Benign	0.18	Neutral	0.79	Possibly damaging	0.06	Tolerated	22.70	Deleterious
p.Arg29Pro	VUS	-53.15	Deleterious	0.929	Pathogenic	-11.13	Pathogenic	0.59	Disease	1.00	Probably damaging	0.02	Deleterious	26.10	Deleterious
p.Arg29Leu	VUS	-0.88	Neutral	0.1876	Benign	-7.71	Pathogenic	0.28	Neutral	1.00	Probably damaging	0.03	Deleterious	25.30	Deleterious
p.Arg29Asp	VUS	-18.67	Indeterminate	0.7182	Pathogenic	-9.12	Pathogenic	0.54	Disease	0.99	Probably damaging	0.01	Deleterious		
p.Arg29Glu	VUS	-4.92	Neutral	0.2562	Benign	-5.39	Benign	0.28	Neutral	0.31	Benign	0.04	Deleterious		
p.Arg29Ala	VUS	-0.01	Neutral	0.2363	Benign	-2.97	Benign	0.45	Neutral	0.94	Possibly damaging	0.03	Deleterious		
p.Arg29Gly	VUS	-1.76	Neutral	0.2867	Benign	-5.63	Benign	0.46	Neutral	0.81	Possibly damaging	0.02	Deleterious	25.40	Deleterious
p.Arg29Val	VUS	-1.92	Neutral	0.2848	Benign	-7.20	Benign	0.50	Neutral	1.00	Probably damaging	0.02	Deleterious		
p.Arg29Tyr	VUS	-0.27	Neutral	0.5312	Ambiguous	-11.00	Pathogenic	0.45	Neutral	0.96	Probably damaging	0.01	Deleterious		
p.Arg29Cys	VUS	-0.99	Neutral	0.3189	Benign	-7.40	Benign	0.47	Neutral	1.00	Probably damaging	0	Deleterious		
p.Arg29Trp	VUS	-0.91	Neutral	0.262	Benign	-8.98	Pathogenic	0.46	Neutral	1.00	Probably damaging	0	Deleterious	32.00	Deleterious
p.Arg29Phe	VUS	-3.19	Neutral	0.5847	Pathogenic	-10.68	Pathogenic	0.48	Neutral	0.96	Probably damaging	0.01	Deleterious		

p.Ala30Asn	VUS	-53.15	Deleterious	0.2215	Benign	-8.71	Pathogenic	0.35	Neutral	0.09	Benign	0.41	Tolerated		
p.Ala30Lys	VUS	-1.25	Neutral	0.2472	Benign	-6.24	Benign	0.36	Neutral	0.60	Possibly damaging	0.5	Tolerated		
p.Ala30Thr	VUS	-23.96	Indeterminate	0.1066	Benign	-6.59	Benign	0.24	Neutral	0.02	Benign	0.51	Tolerated	20.30	Deleterious
p.Ala30Arg	VUS	-3.40	Neutral	0.1463	Benign	-3.24	Benign	0.34	Neutral	0.84	Possibly damaging	0.47	Tolerated		
p.Ala30Ser	VUS	-11.06	Indeterminate	0.1102	Benign	-5.07	Benign	0.26	Neutral	0.00	Benign	0.56	Tolerated	18.06	Deleterious
p.Ala30Ile	VUS	-10.02	Indeterminate	0.2831	Benign	-8.89	Pathogenic	0.47	Neutral	0.60	Possibly damaging	0.43	Tolerated		
p.Ala30Met	VUS	-5.52	Neutral	0.2343	Benign	-8.27	Pathogenic	0.34	Neutral	0.38	Benign	0.24	Tolerated		
p.Ala30His	VUS	-6.94	Indeterminate	0.2277	Benign	-7.13	Benign	0.44	Neutral	0.96	Possibly damaging	0.56	Tolerated		
p.Ala30Gln	VUS	-11.53	Indeterminate	0.1304	Benign	-5.04	Benign	0.30	Neutral	0.09	Benign	0.42	Tolerated		
p.Ala30Pro	VUS	-53.15	Deleterious	0.7136	Pathogenic	-8.71	Pathogenic	0.53	Disease	0.81	Possibly damaging	0.26	Tolerated	23.10	Deleterious
p.Ala30Leu	VUS	-9.74	Indeterminate	0.1437	Benign	-5.95	Benign	0.29	Neutral	0.04	Benign	0.71	Tolerated		
p.Ala30Asp	VUS	-33.22	Indeterminate	0.1445	Benign	-6.67	Benign	0.33	Neutral	0.01	Benign	0.34	Tolerated		
p.Ala30Glu	VUS	-2.24	Neutral	0.0953	Benign	-4.64	Benign	0.27	Neutral	0.01	Benign	0.49	Tolerated	14.41	Neutral
p.Ala30Gly	VUS	-53.15	Deleterious	0.1178	Benign	-5.15	Benign	0.36	Neutral	0.01	Benign	0.41	Tolerated	17.42	Deleterious
p.Ala30Val	VUS	-7.22	Indeterminate	0.1334	Benign	-6.66	Benign	0.35	Neutral	0.83	Possibly damaging	0.56	Tolerated	17.56	Deleterious
p.Ala30Tyr	VUS	-25.61	Indeterminate	0.3408	Ambiguous	-9.70	Pathogenic	0.45	Neutral	0.99	Probably damaging	1	Tolerated		
p.Ala30Cys	VUS	-21.54	Indeterminate	0.4009	Ambiguous	-7.39	Benign	0.42	Neutral	0.99	Probably damaging	0.19	Tolerated		
p.Ala30Trp	VUS	-14.31	Indeterminate	0.4175	Ambiguous	-8.02	Pathogenic	0.52	Disease	1.00	Probably damaging	0.19	Tolerated		
p.Ala30Phe	VUS	-9.80	Indeterminate	0.2629	Benign	-8.95	Pathogenic	0.46	Neutral	0.96	Possibly damaging	0.7	Tolerated		
p.Leu31Asn	VUS	-15.80	Indeterminate	0.8943	Pathogenic	-12.27	Pathogenic	0.74	Disease	1.00	Probably damaging	0	Deleterious		
p.Leu31Lys	VUS	-1.84	Neutral	0.6424	Pathogenic	-10.83	Pathogenic	0.76	Disease	1.00	Probably damaging	0	Deleterious		
p.Leu31Thr	VUS	-7.23	Indeterminate	0.7298	Pathogenic	-8.94	Pathogenic	0.72	Disease	1.00	Probably damaging	0	Deleterious		
p.Leu31Arg	VUS	-3.31	Neutral	0.3758	Ambiguous	-6.01	Benign	0.84	Disease	1.00	Probably damaging	0	Deleterious	31.00	Deleterious
p.Leu31Ser	VUS	-12.36	Indeterminate	0.8197	Pathogenic	-6.81	Benign	0.82	Disease	1.00	Probably damaging	0	Deleterious		
p.Leu31Ile	VUS	-1.36	Neutral	0.3859	Ambiguous	-8.78	Pathogenic	0.53	Disease	1.00	Probably damaging	0.04	Deleterious		
p.Leu31Met	VUS	-6.95	Indeterminate	0.3112	Benign	-6.60	Benign	0.54	Disease	1.00	Probably damaging	0.02	Deleterious	28.90	Deleterious
p.Leu31His	VUS	-5.62	Neutral	0.5671	Pathogenic	-5.44	Benign	0.82	Disease	1.00	Probably damaging	0	Deleterious		
p.Leu31Gln	VUS	-18.98	Indeterminate	0.4554	Ambiguous	-6.92	Benign	0.83	Disease	1.00	Probably damaging	0	Deleterious	30.00	Deleterious
p.Leu31Pro	VUS	-53.15	Deleterious	0.9258	Pathogenic	-12.46	Pathogenic	0.71	Disease	1.00	Probably damaging	0	Deleterious	31.00	Deleterious
p.Leu31Asp	VUS	-53.15	Deleterious	0.9567	Pathogenic	-11.70	Pathogenic	0.73	Disease	1.00	Probably damaging	0	Deleterious		
p.Leu31Glu	VUS	-9.76	Indeterminate	0.7905	Pathogenic	-7.46	Benign	0.76	Disease	1.00	Probably damaging	0	Deleterious		
p.Leu31Ala	VUS	-9.65	Indeterminate	0.447	Ambiguous	-2.53	Benign	0.70	Disease	1.00	Probably damaging	0.01	Deleterious		
p.Leu31Gly	VUS	-6.62	Indeterminate	0.7501	Pathogenic	-8.20	Pathogenic	0.75	Disease	1.00	Probably damaging	0	Deleterious		
p.Leu31Val	VUS	-21.43	Indeterminate	0.3177	Benign	-6.65	Benign	0.44	Neutral	1.00	Probably damaging	0.02	Deleterious	28.50	Deleterious
p.Leu31Tyr	VUS	-21.21	Indeterminate	0.6131	Pathogenic	-6.76	Benign	0.71	Disease	1.00	Probably damaging	0.01	Deleterious		
p.Leu31Cys	VUS	-33.20	Indeterminate	0.7024	Pathogenic	-4.19	Benign	0.66	Disease	1.00	Probably damaging	0	Deleterious		
p.Leu31Trp	VUS	-18.55	Indeterminate	0.5192	Ambiguous	-6.83	Benign	0.76	Disease	1.00	Probably damaging	0	Deleterious		
p.Leu31Phe	VUS	-5.13	Neutral	0.3289	Benign	-6.42	Benign	0.54	Disease	1.00	Probably damaging	0.18	Tolerated		
p.Leu32Asn	VUS	-53.15	Deleterious	0.9879	Pathogenic	-19.16	Pathogenic	0.72	Disease	1.00	Probably damaging	0	Deleterious		
p.Leu32Lys	VUS	-53.15	Deleterious	0.9561	Pathogenic	-18.46	Pathogenic	0.72	Disease	1.00	Probably damaging	0	Deleterious		
p.Leu32Thr	VUS	-15.95	Indeterminate	0.8634	Pathogenic	-14.45	Pathogenic	0.72	Disease	1.00	Probably damaging	0	Deleterious		
p.Leu32Arg	VUS	-53.15	Deleterious	0.8215	Pathogenic	-13.76	Pathogenic	0.75	Disease	1.00	Probably damaging	0	Deleterious	32.00	Deleterious
p.Leu32Ser	VUS	-32.53	Indeterminate	0.9828	Pathogenic	-14.35	Pathogenic	0.82	Disease	1.00	Probably damaging	0	Deleterious		
p.Leu32Ile	VUS	-0.06	Neutral	0.256	Benign	-10.71	Pathogenic	0.46	Neutral	1.00	Probably damaging	0	Deleterious		
p.Leu32Met	VUS	-0.11	Neutral	0.6061	Pathogenic	-10.80	Pathogenic	0.47	Neutral	1.00	Probably damaging	0	Deleterious	29.00	Deleterious
p.Leu32His	VUS	-53.15	Deleterious	0.9762	Pathogenic	-15.47	Pathogenic	0.79	Disease	1.00	Probably damaging	0	Deleterious		
p.Leu32Gln	VUS	-10.66	Indeterminate	0.9367	Pathogenic	-15.08	Pathogenic	0.82	Disease	1.00	Probably damaging	0	Deleterious	29.90	Deleterious
p.Leu32Pro	Pathogenic	-53.15	Deleterious	0.9446	Pathogenic	-13.13	Pathogenic	0.80	Disease	1.00	Probably damaging	0	Deleterious	31.00	Deleterious
p.Leu32Asp	VUS	-53.15	Deleterious	0.9957	Pathogenic	-18.43	Pathogenic	0.72	Disease	1.00	Probably damaging	0	Deleterious		
p.Leu32Glu	VUS	-53.15	Deleterious	0.9666	Pathogenic	-17.68	Pathogenic	0.75	Disease	1.00	Probably damaging	0	Deleterious		
p.Leu32Ala	VUS	-18.61	Indeterminate	0.8518	Pathogenic	-14.08	Pathogenic	0.70	Disease	1.00	Probably damaging	0	Deleterious		
p.Leu32Gly	VUS	-53.15	Deleterious	0.9602	Pathogenic	-15.95	Pathogenic	0.71	Disease	1.00	Probably damaging	0	Deleterious		
p.Leu32Val	VUS	-0.05	Neutral	0.3013	Benign	-11.51	Pathogenic	0.43	Neutral	1.00	Probably damaging	0	Deleterious	28.60	Deleterious
p.Leu32Tyr	VUS	-53.15	Deleterious	0.9847	Pathogenic	-15.88	Pathogenic	0.70	Disease	1.00	Probably damaging	0	Deleterious		
p.Leu32Cys	VUS	-3.18	Neutral	0.9346	Pathogenic	-11.44	Pathogenic	0.65	Disease	1.00	Probably damaging	0	Deleterious		
p.Leu32Trp	VUS	-53.15	Deleterious	0.9562	Pathogenic	-14.68	Pathogenic	0.74	Disease	1.00	Probably damaging	0	Deleterious		
p.Leu32Phe	VUS	-5.09	Neutral	0.8974	Pathogenic	-9.81	Pathogenic	0.52	Disease	1.00	Probably damaging	0	Deleterious		
p.Glu33Asn	VUS	-0.12	Neutral	0.4922	Ambiguous	-8.59	Pathogenic	0.26	Neutral	0.97	Probably damaging	0.06	Tolerated		
p.Glu33Lys	VUS	-0.41	Neutral	0.2662	Benign	-7.44	Benign	0.17	Neutral	0.82	Possibly damaging	0.56	Tolerated	22.20	Deleterious
p.Glu33Thr	VUS	-0.19	Neutral	0.3245	Benign	-7.16	Benign	0.38	Neutral	0.99	Probably damaging	0.06	Tolerated		
p.Glu33Arg	VUS	-9.24	Indeterminate	0.1813	Benign	-5.53	Benign	0.27	Neutral	0.98	Probably damaging	0.46	Tolerated		
p.Glu33Ser	VUS	-3.59	Neutral	0.2948	Benign	-6.08	Benign	0.37	Neutral	0.99	Probably damaging	0.06	Tolerated		
p.Glu33Ile	VUS	-0.01	Neutral	0.6202	Pathogenic	-10.00	Pathogenic	0.38	Neutral	0.99	Probably damaging	0.02	Deleterious		
p.Glu33Met	VUS	-0.01	Neutral	0.6565	Pathogenic	-9.14	Pathogenic	0.44	Neutral	0.99	Probably damaging	0.02	Deleterious		
p.Glu33His	VUS	-0.18	Neutral	0.3876	Ambiguous	-7.31	Benign	0.31	Neutral	0.98	Probably damaging	0.25	Tolerated		
p.Glu33Gln	VUS	-0.09	Neutral	0.1304	Benign	-5.18	Benign	0.16	Neutral	0.20	Benign	0.38	Tolerated	15.26	Deleterious
p.Glu33Pro	VUS	-0.04	Neutral	0.9028	Pathogenic	-10.54	Pathogenic	0.43	Neutral	1.00	Probably damaging	0.04	Deleterious		
p.Glu33Leu	VUS	-0.82	Neutral	0.3512	Ambiguous	-6.18	Benign	0.28	Neutral	0.95	Possibly damaging	0.05	Deleterious		
p.Glu33Asp	VUS	-0.15	Neutral	0.2215	Benign	-4.30	Benign	0.17	Neutral	0.03	Benign	0.05	Deleterious	14.99	Neutral
p.Glu33Ala	VUS	-11.39	Indeterminate	0.1146	Benign	-3.90	Benign	0.21	Neutral	0.96	Probably damaging	0.07	Tolerated	19.25	Deleterious
p.Glu33Gly	VUS	-0.32	Neutral	0.1955	Benign	-6.08	Benign	0.21	Neutral	1.00	Probably damaging	0.04	Deleterious	23.50	Deleterious
p.Glu33Val	VUS	-1.44	Neutral	0.3459	Ambiguous	-7.67	Pathogenic	0.23	Neutral	0.95	Possibly damaging	0.03	Deleterious	23.10	Deleterious
p.Glu33Tyr	VUS	-9.22	Indeterminate	0.7106	Pathogenic	-10.60	Pathogenic	0.38	Neutral	1.00	Probably damaging	0.02	Deleterious		
p.Glu33Cys	VUS	-3.97	Neutral	0.8469	Pathogenic	-8.41	Pathogenic	0.38	Neutral	1.00	Probably damaging	0.01	Deleterious		
p.Glu33Trp	VUS	-0.26	Neutral	0.8764	Pathogenic	-10.04	Pathogenic	0.45	Neutral	1.00	Probably damaging	0.01	Deleterious		
p.Glu33Phe	VUS	-3.58	Neutral	0.7972	Pathogenic	-10.77	Pathogenic	0.39	Neutral	1.00	Probably damaging	0.01	Deleterious		
p.Ala34Asn	VUS	-11.51	Indeterminate	0.275	Benign	-8.01	Pathogenic	0.32	Neutral	0.99	Probably damaging	0.45	Tolerated		
p.Ala34Lys	VUS	-1.08	Neutral	0.3383	Benign	-6.90	Benign	0.45	Neutral	1.00	Probably damaging	0.57	Tolerated		
p.Ala34Thr	VUS	-5.63	Neutral	0.1519	Benign	-7.78	Pathogenic	0.31	Neutral	0.99	Probably damaging	0.57	Tolerated	20.50	Deleterious
p.Ala34Arg	VUS	-0.01	Neutral	0.2107	Benign	-3.95	Benign	0.32	Neutral	1.00	Probably damaging	0.51	Tolerated		
p.Ala34Ser	VUS	-2.77	Neutral	0.1039	Benign	-5.61	Benign	0.21	Neutral	0.94	Possibly damaging	0.62	Tolerated	18.38	Deleterious
p.Ala34Ile	VUS	-0.97	Neutral	0.4646	Ambiguous	-11.26	Pathogenic	0.44	Neutral	1.00	Probably damaging	0.45	Tolerated		
p.Ala34Met	VUS	-0.41	Neutral	0.3905	Ambiguous	-9.67	Pathogenic	0.42	Neutral	1.00	Probably damaging	0.25	Tolerated		
p.Ala34His	VUS	-0.11	Neutral	0.3442	Ambiguous	-6.92	Benign	0.46	Neutral	1.00	Probably damaging	0.57	Tolerated		
p.Ala34Gln	VUS	-1.75	Neutral	0.1966	Benign	-4.53	Benign	0.34	Neutral	1.00	Probably damaging	0.46	Tolerated		
p.Ala34Pro	VUS	-11.89	Indeterminate	0.8853	Pathogenic	-10.72	Pathogenic	0.55	Disease	1.00	Probably damaging	0.29	Tolerated	23.10	Deleterious
p.Ala34Leu	VUS	-0.40	Neutral	0.2923	Benign	-7.01	Benign	0.44	Neutral	1.00	Probably damaging	0.75	Tolerated		
p.Ala34Asp	VUS	-1.54	Neutral	0.1828	Benign	-5.86	Benign	0.33	Neutral	1.00	Probably damaging	0.38	Tolerated		
p.Ala34Glu	VUS	-2.19	Neutral	0.167	Benign	-4.98	Benign	0.31	Neutral	1.00	Probably damaging	0.54	Tolerated	18.56	Deleterious
p.Ala34Gly	VUS	-2.72	Neutral	0.1321	Benign	-4.75	Benign	0.23	Neutral	1.00	Probably damaging	0.45	Tolerated	22.60	Deleterious
p.Ala34Val	VUS	-12.28	Indeterminate	0.2212	Benign	-8.47	Pathogenic	0.23	Neutral	1.00	Probably damaging	0.59	Tolerated	21.90	Deleterious
p.Ala34Tyr	VUS	-0.23	Neutral	0.5224	Ambiguous	-10.87	Pathogenic	0.44	Neutral	1.00	Probably damaging	1	Tolerated		
p.Ala34Cys	VUS	-0.28	Neutral	0.562	Ambiguous	-8.03	Pathogenic	0.41	Neutral	1.00	Probably damaging	0.19	Tolerated		
p.Ala34Trp	VUS	-1.47	Neutral	0.6783	Pathogenic	-10.10	Pathogenic	0.47	Neutral	1.00	Probably damaging	0.19	Tolerated		
p.Ala34Phe	VUS	-0.11	Neutral	0.483	Ambiguous	-11.46	Pathogenic	0.42	Neutral	1.00	Probably damaging	0.71	Tolerated		
p.Gly35Asn	VUS	-7.34	Indeterminate	0.6621	Pathogenic	-12.62	Pathogenic	0.49	Neutral	1.00	Probably damaging	0.49	Tolerated		
p.Gly35Lys	VUS	-13.25	Indeterminate	0.6573	Pathogenic	-13.14	Pathogenic	0.65	Disease	1.00	Probably damaging	0.33	Tolerated		
p.Gly35Thr	VUS	-53.15	Deleterious	0.6714	Pathogenic	-13.85	Pathogenic	0.62	Disease	1.00	Probably damaging	0.08	Tolerated		
p.Gly35Arg	VUS	-2.42	Neutral	0.4978	Ambiguous	-10.24	Pathogenic	0.68	Disease	1.00	Probably damaging	0.09	Tolerated	27.55	Deleterious
p.Gly35Ser	VUS	-13.90	Indeterminate	0.3827	Ambiguous	-11.39	Pathogenic	0.62	Disease	1.00	Probably damaging	0.16	Tolerated		
p.Gly35Ile	VUS	-53.15	Deleterious	0.9411	Pathogenic	-17.95	Pathogenic	0.65	Disease	1.00	Probably damaging	0.01	Deleterious		
p.Gly35Met	VUS	-21.31	Indeterminate	0.936	Pathogenic	-15.80	Pathogenic	0.67	Disease	1.00	Probably damaging	0.01	Deleterious		
p.Gly35His	VUS	-2.71	Neutral	0.6966	Pathogenic	-12.46	Pathogenic	0.63	Disease	1.00	Probably damaging	0.06	Tolerated		
p.Gly35Gln	VUS	-9.22	Indeterminate	0.6005	Pathogenic	-12.67	Pathogenic	0.48	Neutral	1.00	Probably damaging	0.09	Tolerated		
p.Gly35Pro	VUS	-53.15	Deleterious	0.9693	Pathogenic	-14.73	Pathogenic	0.61	Disease	1.00	Probably damaging	0.06	Tolerated		
p.Gly35Leu	VUS	-53.15	Deleterious	0.9122	Pathogenic	-14.52	Pathogenic	0.67	Disease	1.00	Probably damaging	0.03	Deleterious		
p.Gly35Asp	VUS	-17.21	Indeterminate	0.4675	Ambiguous	-11.44	Pathogenic	0.70	Disease	1.00	Probably damaging	0.28	Tolerated		
p.Gly35Glu	VUS	-10.41	Indeterminate	0.5927	Pathogenic	-12.25	Pathogenic	0.71	Disease	1.00	Probably damaging	0.31	Tolerated	26.50	Deleterious
p.Gly35Ala	VUS	-7.55	Indeterminate	0.483	Ambiguous	-11.44	Pathogenic	0.62	Disease	1.00	Probably damaging	0.09	Tolerated	25.70	Deleterious
p.Gly35Val	VUS	-53.15	Deleterious	0.8532	Pathogenic	-14.01	Pathogenic	0.78	Disease	1.00	Probably damaging	0.02	Deleterious	31.00	Deleterious
p.Gly35Tyr	VUS	-26.28	Indeterminate	0.8898	Pathogenic	-15.99	Pathogenic	0.66	Disease	1.00	Probably damaging	0.03	Deleterious		

p.Gly35Cys	VUS	-12.62	Indeterminate	0.7264	Pathogenic	-12.78	Pathogenic	0.75	Disease	1.00	Probably damaging	0.01	Deleterious		
p.Gly35Trp	VUS	-53.15	Deleterious	0.8878	Pathogenic	-14.67	Pathogenic	0.70	Disease	1.00	Probably damaging	0.01	Deleterious	32.00	Deleterious
p.Gly35Phe	VUS	-8.93	Indeterminate	0.9481	Pathogenic	-16.86	Pathogenic	0.67	Disease	1.00	Probably damaging	0.01	Deleterious		
p.Ala36Asn	VUS	-21.29	Indeterminate	0.4997	Ambiguous	-11.26	Pathogenic	0.44	Neutral	0.55	Possibly damaging	0.02	Deleterious		
p.Ala36Lys	VUS	-53.15	Deleterious	0.7005	Pathogenic	-10.58	Pathogenic	0.49	Neutral	0.98	Probably damaging	0.03	Deleterious		
p.Ala36Thr	VUS	-15.57	Indeterminate	0.1621	Benign	-5.22	Benign	0.20	Neutral	0.02	Benign	0.2	Tolerated	22.70	Deleterious
p.Ala36Arg	VUS	-0.86	Neutral	0.4371	Ambiguous	-7.93	Pathogenic	0.44	Neutral	0.98	Probably damaging	0.02	Deleterious		
p.Ala36Ser	VUS	-3.16	Neutral	0.1364	Benign	-6.94	Benign	0.34	Neutral	0.08	Benign	0.04	Deleterious	23.70	Deleterious
p.Ala36Ile	VUS	-0.14	Neutral	0.3407	Ambiguous	-8.07	Pathogenic	0.45	Neutral	0.85	Possibly damaging	0.22	Tolerated		
p.Ala36Met	VUS	0.00	Neutral	0.3645	Ambiguous	-6.87	Benign	0.31	Neutral	0.99	Probably damaging	0.05	Deleterious		
p.Ala36His	VUS	-0.02	Neutral	0.4481	Ambiguous	-7.60	Pathogenic	0.43	Neutral	1.00	Probably damaging	0.01	Deleterious		
p.Ala36Gln	VUS	-0.07	Neutral	0.3982	Ambiguous	-5.70	Benign	0.44	Neutral	0.99	Probably damaging	0.02	Deleterious		
p.Ala36Pro	VUS	-53.15	Deleterious	0.7831	Pathogenic	-11.46	Pathogenic	0.59	Disease	0.94	Possibly damaging	0.05	Deleterious	26.20	Deleterious
p.Ala36Leu	VUS	-5.94	Indeterminate	0.2434	Benign	-5.75	Benign	0.47	Neutral	0.85	Possibly damaging	0.29	Tolerated		
p.Ala36Asp	VUS	-3.54	Neutral	0.5068	Ambiguous	-9.06	Pathogenic	0.52	Disease	0.91	Possibly damaging	0.01	Deleterious		
p.Ala36Glu	VUS	-6.72	Indeterminate	0.4466	Ambiguous	-5.04	Benign	0.46	Neutral	0.97	Probably damaging	0.03	Deleterious	21.60	Deleterious
p.Ala36Gly	VUS	-11.46	Indeterminate	0.2002	Benign	-7.89	Pathogenic	0.32	Neutral	0.89	Possibly damaging	0.01	Deleterious	22.40	Deleterious
p.Ala36Val	VUS	-0.06	Neutral	0.1435	Benign	-3.71	Benign	0.18	Neutral	0.05	Benign	1	Tolerated	15.30	Deleterious
p.Ala36Tyr	VUS	-4.78	Neutral	0.4751	Ambiguous	-9.39	Pathogenic	0.43	Neutral	0.99	Probably damaging	0.01	Deleterious		
p.Ala36Cys	VUS	0.00	Neutral	0.3531	Ambiguous	-6.44	Benign	0.42	Neutral	1.00	Probably damaging	0.04	Deleterious		
p.Ala36Trp	VUS	0.00	Neutral	0.6743	Pathogenic	-8.05	Pathogenic	0.52	Disease	1.00	Probably damaging	0	Deleterious		
p.Ala36Phe	VUS	-2.39	Neutral	0.3369	Benign	-9.00	Pathogenic	0.45	Neutral	0.98	Probably damaging	0.03	Deleterious		
p.Leu37Asn	VUS	-0.07	Neutral	0.1197	Benign	-0.16	Benign	0.20	Neutral	0.08	Benign	0.53	Tolerated		
p.Leu37Lys	VUS	-8.20	Indeterminate	0.1038	Benign	-5.18	Benign	0.27	Neutral	0.08	Benign	0.86	Tolerated		
p.Leu37Thr	VUS	-15.43	Indeterminate	0.1027	Benign	-4.14	Benign	0.24	Neutral	0.02	Benign	0.54	Tolerated		
p.Leu37Arg	VUS	0.00	Neutral	0.0572	Benign	-3.63	Benign	0.12	Neutral	0.14	Benign	0.48	Tolerated	13.65	Neutral
p.Leu37Ser	VUS	-3.05	Neutral	0.0723	Benign	0.97	Benign	0.12	Neutral	0.00	Benign	0.76	Tolerated		
p.Leu37Ile	VUS	-15.63	Indeterminate	0.1386	Benign	-6.99	Benign	0.18	Neutral	0.15	Benign	0.18	Tolerated		
p.Leu37Met	VUS	-0.02	Neutral	0.1321	Benign	-6.51	Benign	0.20	Neutral	0.78	Possibly damaging	0.1	Tolerated	13.49	Neutral
p.Leu37His	VUS	-8.27	Indeterminate	0.0924	Benign	-2.66	Benign	0.13	Neutral	0.82	Possibly damaging	0.13	Tolerated		
p.Leu37Gln	VUS	-9.51	Indeterminate	0.0564	Benign	-3.34	Benign	0.11	Neutral	0.14	Benign	0.55	Tolerated	12.85	Neutral
p.Leu37Pro	VUS	-27.67	Indeterminate	0.0683	Benign	-0.36	Benign	0.12	Neutral	0.00	Benign	0.26	Tolerated	14.31	Neutral
p.Leu37Asp	VUS	-2.34	Neutral	0.1044	Benign	3.18	Benign	0.21	Neutral	0.00	Benign	0.68	Tolerated		
p.Leu37Glu	VUS	-2.57	Neutral	0.0784	Benign	-0.46	Benign	0.29	Neutral	0.02	Benign	1	Tolerated		
p.Leu37Ala	VUS	-0.14	Neutral	0.0689	Benign	-1.70	Benign	0.20	Neutral	0.01	Benign	0.71	Tolerated		
p.Leu37Gly	VUS	-2.03	Neutral	0.2015	Benign	-2.31	Benign	0.27	Neutral	0.02	Benign	0.36	Tolerated		
p.Leu37Val	VUS	-9.83	Indeterminate	0.076	Benign	-4.47	Benign	0.13	Neutral	0.06	Benign	0.31	Tolerated	10.87	Neutral
p.Leu37Tyr	VUS	-8.28	Indeterminate	0.2359	Benign	-5.79	Benign	0.25	Neutral	0.82	Possibly damaging	0.06	Tolerated		
p.Leu37Cys	VUS	-0.85	Neutral	0.326	Benign	-2.31	Benign	0.26	Neutral	0.82	Possibly damaging	0.05	Deleterious		
p.Leu37Trp	VUS	-53.15	Deleterious	0.1527	Benign	-5.76	Benign	0.25	Neutral	0.95	Possibly damaging	0.02	Deleterious		
p.Leu37Phe	VUS	-0.24	Neutral	0.1341	Benign	-5.89	Benign	0.10	Neutral	0.30	Benign	0.06	Tolerated		
p.Pro38Asn	VUS	-53.15	Deleterious	0.9812	Pathogenic	-17.83	Pathogenic	0.47	Neutral	0.97	Probably damaging	0.03	Deleterious		
p.Pro38Lys	VUS	-53.15	Deleterious	0.9373	Pathogenic	-15.62	Pathogenic	0.53	Disease	0.99	Probably damaging	0.06	Tolerated		
p.Pro38Thr	VUS	-6.12	Indeterminate	0.6142	Pathogenic	-11.08	Pathogenic	0.40	Neutral	0.75	Possibly damaging	0.25	Tolerated	20.60	Deleterious
p.Pro38Arg	VUS	-53.15	Deleterious	0.7822	Pathogenic	-13.17	Pathogenic	0.58	Disease	0.99	Probably damaging	0.04	Deleterious	24.30	Deleterious
p.Pro38Ser	VUS	-17.33	Indeterminate	0.7699	Pathogenic	-11.06	Pathogenic	0.59	Disease	0.95	Possibly damaging	0.07	Tolerated	17.36	Deleterious
p.Pro38Ile	VUS	-18.09	Indeterminate	0.7013	Pathogenic	-12.16	Pathogenic	0.47	Neutral	0.99	Probably damaging	0.61	Tolerated		
p.Pro38Met	VUS	-27.70	Indeterminate	0.9268	Pathogenic	-14.33	Pathogenic	0.47	Neutral	1.00	Probably damaging	0.1	Tolerated		
p.Pro38His	VUS	-53.15	Deleterious	0.9314	Pathogenic	-14.31	Pathogenic	0.46	Neutral	1.00	Probably damaging	0.02	Deleterious	24.60	Deleterious
p.Pro38Gln	VUS	-53.15	Deleterious	0.8702	Pathogenic	-14.75	Pathogenic	0.44	Neutral	0.99	Probably damaging	0.04	Deleterious		
p.Pro38Leu	VUS	-33.22	Indeterminate	0.6615	Pathogenic	-11.99	Pathogenic	0.42	Neutral	0.98	Probably damaging	0.39	Tolerated	24.50	Deleterious
p.Pro38Asp	VUS	-53.15	Deleterious	0.9831	Pathogenic	-16.69	Pathogenic	0.50	Neutral	0.96	Probably damaging	0.02	Deleterious		
p.Pro38Glu	VUS	-25.68	Indeterminate	0.9228	Pathogenic	-14.43	Pathogenic	0.47	Neutral	0.78	Possibly damaging	0.06	Tolerated		
p.Pro38Ala	VUS	-53.15	Deleterious	0.297	Benign	-6.83	Benign	0.19	Neutral	0.03	Benign	0.37	Tolerated	15.27	Deleterious
p.Pro38Gly	VUS	-0.71	Neutral	0.8417	Pathogenic	-11.38	Pathogenic	0.46	Neutral	0.95	Possibly damaging	0.02	Deleterious		
p.Pro38Val	VUS	-0.53	Neutral	0.3993	Ambiguous	-7.82	Pathogenic	0.28	Neutral	0.74	Possibly damaging	1	Tolerated		
p.Pro38Tyr	VUS	-53.15	Deleterious	0.9766	Pathogenic	-16.85	Pathogenic	0.48	Neutral	1.00	Probably damaging	0.02	Deleterious		
p.Pro38Cys	VUS	-0.04	Neutral	0.8647	Pathogenic	-11.25	Pathogenic	0.47	Neutral	1.00	Probably damaging	0.07	Tolerated		
p.Pro38Trp	VUS	-53.15	Deleterious	0.9811	Pathogenic	-16.36	Pathogenic	0.53	Disease	1.00	Probably damaging	0.01	Deleterious		
p.Pro38Phe	VUS	-53.15	Deleterious	0.9761	Pathogenic	-16.36	Pathogenic	0.48	Neutral	1.00	Probably damaging	0.07	Tolerated		
p.Asn39Lys	VUS	-22.94	Indeterminate	0.9206	Pathogenic	-10.77	Pathogenic	0.56	Disease	0.97	Probably damaging	0.02	Deleterious	22.45	Deleterious
p.Asn39Thr	VUS	-0.01	Neutral	0.479	Ambiguous	-7.19	Benign	0.47	Neutral	0.65	Possibly damaging	0.01	Deleterious	23.90	Deleterious
p.Asn39Arg	VUS	-3.95	Neutral	0.8501	Pathogenic	-8.02	Pathogenic	0.52	Disease	0.99	Probably damaging	0.01	Deleterious		
p.Asn39Ser	VUS	-4.05	Neutral	0.1805	Benign	-5.65	Benign	0.64	Disease	0.95	Possibly damaging	0.03	Deleterious	22.20	Deleterious
p.Asn39Ile	VUS	-7.95	Indeterminate	0.9261	Pathogenic	-10.35	Pathogenic	0.68	Disease	0.98	Probably damaging	0	Deleterious	24.70	Deleterious
p.Asn39Met	VUS	-25.20	Indeterminate	0.8921	Pathogenic	-9.61	Pathogenic	0.61	Disease	1.00	Probably damaging	0	Deleterious		
p.Asn39His	VUS	-0.03	Neutral	0.4608	Ambiguous	-7.72	Pathogenic	0.36	Neutral	0.99	Probably damaging	0.01	Deleterious	16.04	Deleterious
p.Asn39Gln	VUS	-1.24	Neutral	0.7922	Pathogenic	-8.55	Pathogenic	0.52	Disease	0.98	Probably damaging	0.01	Deleterious		
p.Asn39Pro	VUS	-53.15	Deleterious	0.9481	Pathogenic	-11.48	Pathogenic	0.53	Disease	0.99	Probably damaging	0.01	Deleterious		
p.Asn39Leu	VUS	-53.15	Deleterious	0.7873	Pathogenic	-8.94	Pathogenic	0.60	Disease	0.98	Probably damaging	0	Deleterious		
p.Asn39Asp	VUS	-0.66	Neutral	0.171	Benign	-4.01	Benign	0.26	Neutral	0.05	Benign	0.62	Tolerated	12.26	Neutral
p.Asn39Glu	VUS	-1.56	Neutral	0.7546	Pathogenic	-6.00	Benign	0.51	Disease	0.72	Possibly damaging	0.02	Deleterious		
p.Asn39Ala	VUS	-6.60	Indeterminate	0.6305	Pathogenic	-6.35	Benign	0.50	Neutral	0.74	Possibly damaging	0.01	Deleterious		
p.Asn39Gly	VUS	-0.34	Neutral	0.5207	Ambiguous	-6.75	Benign	0.50	Disease	0.99	Probably damaging	0.04	Deleterious		
p.Asn39Val	VUS	-4.58	Neutral	0.8577	Pathogenic	-7.98	Pathogenic	0.59	Disease	0.81	Possibly damaging	0	Deleterious		
p.Asn39Tyr	VUS	-0.72	Neutral	0.77	Pathogenic	-12.28	Pathogenic	0.58	Disease	1.00	Probably damaging	0.01	Deleterious	22.00	Deleterious
p.Asn39Cys	VUS	-53.15	Deleterious	0.7817	Pathogenic	-7.10	Benign	0.59	Disease	1.00	Probably damaging	0	Deleterious		
p.Asn39Trp	VUS	-13.56	Indeterminate	0.9764	Pathogenic	-12.04	Pathogenic	0.62	Disease	1.00	Probably damaging	0	Deleterious		
p.Asn39Phe	VUS	-53.15	Deleterious	0.9575	Pathogenic	-12.35	Pathogenic	0.62	Disease	1.00	Probably damaging	0	Deleterious		
p.Ala40Asn	VUS	-6.02	Indeterminate	0.4103	Ambiguous	-12.26	Pathogenic	0.37	Neutral	0.88	Possibly damaging	0.35	Tolerated		
p.Ala40Lys	VUS	-1.40	Neutral	0.3873	Ambiguous	-9.78	Pathogenic	0.42	Neutral	0.94	Possibly damaging	0.65	Tolerated		
p.Ala40Thr	VUS	-53.15	Deleterious	0.1094	Benign	-5.35	Benign	0.13	Neutral	0.98	Probably damaging	0.49	Tolerated	20.10	Deleterious
p.Ala40Arg	VUS	-2.18	Neutral	0.2217	Benign	-7.59	Pathogenic	0.40	Neutral	0.19	Benign	0.4	Tolerated		
p.Ala40Ser	VUS	-0.83	Neutral	0.1333	Benign	-6.22	Benign	0.17	Neutral	0.54	Possibly damaging	0.52	Tolerated	22.60	Deleterious
p.Ala40Ile	VUS	-6.06	Indeterminate	0.2938	Benign	-9.12	Pathogenic	0.29	Neutral	1.00	Probably damaging	0.15	Tolerated		
p.Ala40Met	VUS	-0.01	Neutral	0.3271	Benign	-9.25	Pathogenic	0.40	Neutral	1.00	Probably damaging	0.08	Tolerated		
p.Ala40His	VUS	-0.39	Neutral	0.3516	Ambiguous	-10.41	Pathogenic	0.39	Neutral	1.00	Probably damaging	0.11	Tolerated		
p.Ala40Gln	VUS	-0.02	Neutral	0.1982	Benign	-7.81	Pathogenic	0.38	Neutral	0.99	Probably damaging	0.41	Tolerated		
p.Ala40Pro	VUS	-12.20	Indeterminate	0.5607	Ambiguous	-8.42	Pathogenic	0.36	Neutral	1.00	Probably damaging	0.2	Tolerated	24.40	Deleterious
p.Ala40Leu	VUS	-53.15	Deleterious	0.1751	Benign	-8.21	Pathogenic	0.47	Neutral	0.97	Probably damaging	0.36	Tolerated		
p.Ala40Asp	VUS	-17.56	Indeterminate	0.3014	Benign	-9.74	Pathogenic	0.36	Neutral	0.98	Probably damaging	0.43	Tolerated		
p.Ala40Glu	VUS	-0.05	Neutral	0.1761	Benign	-6.70	Benign	0.36	Neutral	0.90	Possibly damaging	0.8	Tolerated	16.96	Deleterious
p.Ala40Gly	VUS	-0.94	Neutral	0.1478	Benign	-5.36	Benign	0.15	Neutral	0.01	Benign	0.33	Tolerated	15.48	Deleterious
p.Ala40Val	VUS	-6.48	Indeterminate	0.13	Benign	-6.01	Benign	0.19	Neutral	0.95	Possibly damaging	0.26	Tolerated	21.60	Deleterious
p.Ala40Tyr	VUS	-0.12	Neutral	0.4958	Ambiguous	-11.90	Pathogenic	0.42	Neutral	1.00	Probably damaging	0.08	Tolerated		
p.Ala40Cys	VUS	-1.56	Neutral	0.5347	Ambiguous	-8.14	Pathogenic	0.38	Neutral	1.00	Probably damaging	0.05	Deleterious		
p.Ala40Trp	VUS	-5.12	Neutral	0.6552	Pathogenic	-10.32	Pathogenic	0.49	Neutral	1.00	Probably damaging	0.02	Deleterious		
p.Ala40Phe	VUS	-2.21	Neutral	0.3808	Ambiguous	-10.62	Pathogenic	0.45	Neutral	1.00	Probably damaging	0.08	Tolerated		
p.Pro41Asn	VUS	-0.39	Neutral	0.5988	Pathogenic	-10.56	Pathogenic	0.27	Neutral	0.67	Possibly damaging	0.07	Tolerated		
p.Pro41Lys	VUS	-4.11	Neutral	0.3597	Ambiguous	-8.97	Pathogenic	0.24	Neutral	0.32	Benign	0.11	Tolerated		
p.Pro41Thr	VUS	-1.20	Neutral	0.1485	Benign	-4.89	Benign	0.11	Neutral	0.08	Benign	0.35	Tolerated	11.57	Neutral
p.Pro41Arg	VUS	-29.93	Indeterminate	0.1457	Benign	-5.20	Benign	0.09	Neutral	0.06	Benign	0.08	Tolerated	4.17	Neutral
p.Pro41Ser	VUS	-0.17	Neutral	0.1671	Benign	-7.00	Benign	0.17	Neutral	0.84	Possibly damaging	0.13	Tolerated	12.33	Neutral
p.Pro41Ile	VUS	-11.50	Indeterminate	0.4238	Ambiguous	-4.31	Benign	0.28	Neutral	0.32	Benign	0.51	Tolerated		
p.Pro41Met	VUS	-0.01	Neutral	0.506	Ambiguous	-5.75	Benign	0.26	Neutral	0.75	Possibly damaging	0.14	Tolerated		
p.Pro41His	VUS	-18.07	Indeterminate	0.3498	Ambiguous	-5.42	Benign	0.17	Neutral	0.95	Possibly damaging	0.07	Tolerated		
p.Pro41Gln	VUS	-2.48	Neutral	0.1882	Benign	-5.74	Benign	0.21	Neutral	0.85	Possibly damaging	0.09	Tolerated	12.54	Neutral
p.Pro41Leu	VUS	-0.03	Neutral	0.2279	Benign	-0.97	Benign	0.11	Neutral	0.01	Benign	0.51	Tolerated	8.79	Neutral
p.Pro41Asp	VUS	-2.62	Neutral	0.584	Pathogenic	-11.96	Pathogenic	0.28	Neutral	0.34	Benign	0.05	Deleterious		
p.Pro41Glu	VUS	-0.14	Neutral	0.3055	Benign	-7.52	Pathogenic	0.24	Neutral	0.07	Benign	0.1	Tolerated		
p.Pro41Ala	VUS	-0.27	Neutral	0.07	Benign	-4.16	Benign	0.15	Neutral	0.12	Benign	0.51	Tolerated	9.37	Neutral

p.Pro41Gly	VUS	-1.82	Neutral	0.2599	Benign	-7.59	Pathogenic	0.26	Neutral	0.84	Possibly damaging	0.05	Deleterious		
p.Pro41Val	VUS	-0.30	Neutral	0.2118	Benign	-1.60	Benign	0.19	Neutral	0.00	Benign	1	Tolerated		
p.Pro41Tyr	VUS	-16.93	Indeterminate	0.6575	Pathogenic	-7.76	Pathogenic	0.26	Neutral	0.94	Possibly damaging	0.11	Tolerated		
p.Pro41Cys	VUS	-3.07	Neutral	0.5137	Ambiguous	-6.71	Benign	0.26	Neutral	1.00	Probably damaging	0.11	Tolerated		
p.Pro41Trp	VUS	-6.53	Indeterminate	0.6471	Pathogenic	-10.69	Pathogenic	0.29	Neutral	1.00	Probably damaging	0.03	Deleterious		
p.Pro41Phe	VUS	-4.10	Neutral	0.6351	Pathogenic	-6.87	Benign	0.26	Neutral	0.89	Possibly damaging	0.14	Tolerated		
p.Asn42Lys	VUS	-53.15	Deleterious	0.9801	Pathogenic	-13.18	Pathogenic	0.71	Disease	1.00	Probably damaging	0	Deleterious	22.60	Deleterious
p.Asn42Thr	VUS	-3.68	Neutral	0.6044	Pathogenic	-9.85	Pathogenic	0.64	Disease	0.99	Probably damaging	0	Deleterious	22.70	Deleterious
p.Asn42Arg	VUS	-53.15	Deleterious	0.944	Pathogenic	-10.75	Pathogenic	0.67	Disease	1.00	Probably damaging	0	Deleterious		
p.Asn42Ser	VUS	-3.19	Neutral	0.2969	Benign	-8.85	Pathogenic	0.61	Disease	1.00	Probably damaging	0	Deleterious	22.30	Deleterious
p.Asn42Ile	VUS	-53.15	Deleterious	0.9567	Pathogenic	-12.14	Pathogenic	0.82	Disease	1.00	Probably damaging	0	Deleterious	23.00	Deleterious
p.Asn42Met	VUS	-53.15	Deleterious	0.9535	Pathogenic	-13.26	Pathogenic	0.76	Disease	1.00	Probably damaging	0	Deleterious		
p.Asn42His	VUS	-53.15	Deleterious	0.7557	Pathogenic	-10.08	Pathogenic	0.73	Disease	1.00	Probably damaging	0	Deleterious	23.60	Deleterious
p.Asn42Gln	VUS	-53.15	Deleterious	0.9405	Pathogenic	-12.35	Pathogenic	0.66	Disease	1.00	Probably damaging	0	Deleterious		
p.Asn42Pro	VUS	-53.15	Deleterious	0.966	Pathogenic	-12.38	Pathogenic	0.71	Disease	1.00	Probably damaging	0	Deleterious		
p.Asn42Leu	VUS	-53.15	Deleterious	0.8813	Pathogenic	-11.52	Pathogenic	0.75	Disease	1.00	Probably damaging	0	Deleterious		
p.Asn42Asp	VUS	-10.68	Indeterminate	0.5772	Pathogenic	-7.97	Pathogenic	0.64	Disease	1.00	Probably damaging	0	Deleterious	23.80	Deleterious
p.Asn42Glu	VUS	-53.15	Deleterious	0.9572	Pathogenic	-14.75	Pathogenic	0.62	Disease	1.00	Probably damaging	0	Deleterious		
p.Asn42Ala	VUS	-3.46	Neutral	0.87	Pathogenic	-10.39	Pathogenic	0.69	Disease	1.00	Probably damaging	0	Deleterious		
p.Asn42Gly	VUS	-0.24	Neutral	0.7349	Pathogenic	-9.50	Pathogenic	0.65	Disease	1.00	Probably damaging	0	Deleterious		
p.Asn42Val	VUS	-53.15	Deleterious	0.9156	Pathogenic	-12.01	Pathogenic	0.74	Disease	1.00	Probably damaging	0	Deleterious		
p.Asn42Tyr	VUS	-53.15	Deleterious	0.9089	Pathogenic	-14.34	Pathogenic	0.82	Disease	1.00	Probably damaging	0	Deleterious	24.20	Deleterious
p.Asn42Cys	VUS	-6.36	Indeterminate	0.776	Pathogenic	-12.33	Pathogenic	0.73	Disease	1.00	Probably damaging	0	Deleterious		
p.Asn42Trp	VUS	-53.15	Deleterious	0.9933	Pathogenic	-14.74	Pathogenic	0.76	Disease	1.00	Probably damaging	0	Deleterious		
p.Asn42Phe	VUS	-53.15	Deleterious	0.9846	Pathogenic	-14.82	Pathogenic	0.75	Disease	1.00	Probably damaging	0	Deleterious		
p.Ser43Asn	VUS	-3.02	Neutral	0.2256	Benign	-5.25	Benign	0.16	Neutral	0.02	Benign	0.53	Tolerated	15.04	Deleterious
p.Ser43Lys	VUS	-0.48	Neutral	0.4613	Ambiguous	-2.21	Benign	0.23	Neutral	0.17	Benign	0.87	Tolerated		
p.Ser43Thr	VUS	-0.40	Neutral	0.1071	Benign	-4.90	Benign	0.13	Neutral	0.02	Benign	0.56	Tolerated	14.53	Neutral
p.Ser43Arg	VUS	-0.31	Neutral	0.23	Benign	0.31	Benign	0.07	Neutral	0.00	Benign	0.56	Tolerated	5.19	Neutral
p.Ser43Ile	VUS	-6.55	Indeterminate	0.2521	Benign	-6.92	Benign	0.17	Neutral	0.77	Possibly damaging	0.17	Tolerated	16.76	Deleterious
p.Ser43Met	VUS	-0.84	Neutral	0.4134	Ambiguous	-7.16	Benign	0.22	Neutral	0.98	Probably damaging	0.1	Tolerated		
p.Ser43His	VUS	-0.33	Neutral	0.2069	Benign	-4.76	Benign	0.21	Neutral	0.00	Benign	0.13	Tolerated		
p.Ser43Gln	VUS	-0.30	Neutral	0.279	Benign	-4.04	Benign	0.22	Neutral	0.47	Possibly damaging	0.55	Tolerated		
p.Ser43Pro	VUS	-0.74	Neutral	0.1875	Benign	-5.93	Benign	0.16	Neutral	0.78	Possibly damaging	0.26	Tolerated		
p.Ser43Leu	VUS	-1.74	Neutral	0.2431	Benign	-6.35	Benign	0.16	Neutral	0.47	Possibly damaging	0.28	Tolerated		
p.Ser43Asp	VUS	-1.70	Neutral	0.3709	Ambiguous	-6.10	Benign	0.21	Neutral	0.33	Benign	0.67	Tolerated		
p.Ser43Glu	VUS	-0.60	Neutral	0.3541	Ambiguous	-4.04	Benign	0.20	Neutral	0.13	Benign	1	Tolerated		
p.Ser43Ala	VUS	-0.70	Neutral	0.0769	Benign	-2.11	Benign	0.11	Neutral	0.01	Benign	0.71	Tolerated		
p.Ser43Gly	VUS	-4.02	Neutral	0.0829	Benign	-4.55	Benign	0.13	Neutral	0.01	Benign	0.43	Tolerated	8.63	Neutral
p.Ser43Val	VUS	-1.68	Neutral	0.2302	Benign	-5.21	Benign	0.21	Neutral	0.62	Possibly damaging	0.26	Tolerated		
p.Ser43Tyr	VUS	-2.22	Neutral	0.2925	Benign	-8.82	Pathogenic	0.22	Neutral	0.69	Possibly damaging	0.05	Deleterious		
p.Ser43Cys	VUS	-6.00	Indeterminate	0.1382	Benign	-5.33	Benign	0.14	Neutral	0.91	Possibly damaging	0.05	Deleterious	10.53	Neutral
p.Ser43Trp	VUS	-2.10	Neutral	0.3461	Ambiguous	-7.76	Pathogenic	0.27	Neutral	0.99	Probably damaging	0.02	Deleterious		
p.Ser43Phe	VUS	-1.32	Neutral	0.3716	Ambiguous	-9.45	Pathogenic	0.18	Neutral	0.82	Possibly damaging	0.05	Deleterious		
p.Tyr44Asn	VUS	-3.69	Neutral	0.5574	Ambiguous	-12.78	Pathogenic	0.27	Neutral	0.99	Probably damaging	0.26	Tolerated	14.06	Neutral
p.Tyr44Lys	VUS	-8.10	Indeterminate	0.7979	Pathogenic	-13.39	Pathogenic	0.31	Neutral	0.97	Probably damaging	0.15	Tolerated		
p.Tyr44Thr	VUS	-1.08	Neutral	0.7134	Pathogenic	-12.72	Pathogenic	0.35	Neutral	1.00	Probably damaging	0.2	Tolerated		
p.Tyr44Arg	VUS	-3.23	Neutral	0.5169	Ambiguous	-10.93	Pathogenic	0.29	Neutral	1.00	Probably damaging	0.15	Tolerated		
p.Tyr44Ser	VUS	-3.02	Neutral	0.4284	Ambiguous	-10.59	Pathogenic	0.23	Neutral	1.00	Probably damaging	0.32	Tolerated	13.61	Neutral
p.Tyr44Ile	VUS	-3.05	Neutral	0.7981	Pathogenic	-14.02	Pathogenic	0.30	Neutral	0.09	Benign	0.18	Tolerated		
p.Tyr44Met	VUS	-3.22	Neutral	0.816	Pathogenic	-11.39	Pathogenic	0.30	Neutral	0.99	Probably damaging	0.1	Tolerated		
p.Tyr44His	VUS	-1.55	Neutral	0.3798	Ambiguous	-9.04	Pathogenic	0.14	Neutral	0.09	Benign	0.24	Tolerated	9.59	Neutral
p.Tyr44Gln	VUS	-18.21	Indeterminate	0.6722	Pathogenic	-11.73	Pathogenic	0.23	Neutral	1.00	Probably damaging	0.14	Tolerated		
p.Tyr44Pro	VUS	-32.89	Indeterminate	0.9497	Pathogenic	-13.45	Pathogenic	0.30	Neutral	1.00	Probably damaging	0.11	Tolerated		
p.Tyr44Leu	VUS	-2.69	Neutral	0.6422	Pathogenic	-8.40	Pathogenic	0.27	Neutral	0.00	Benign	0.31	Tolerated		
p.Tyr44Asp	VUS	-6.48	Indeterminate	0.6152	Pathogenic	-12.63	Pathogenic	0.25	Neutral	1.00	Probably damaging	0.32	Tolerated	14.26	Neutral
p.Tyr44Glu	VUS	-13.61	Indeterminate	0.8125	Pathogenic	-13.47	Pathogenic	0.31	Neutral	0.99	Probably damaging	0.16	Tolerated		
p.Tyr44Ala	VUS	-26.29	Indeterminate	0.5926	Pathogenic	-11.13	Pathogenic	0.29	Neutral	0.98	Probably damaging	0.25	Tolerated		
p.Tyr44Gly	VUS	-11.44	Indeterminate	0.5878	Pathogenic	-12.28	Pathogenic	0.30	Neutral	1.00	Probably damaging	0.22	Tolerated		
p.Tyr44Val	VUS	-0.88	Neutral	0.6303	Pathogenic	-12.57	Pathogenic	0.32	Neutral	0.86	Possibly damaging	0.22	Tolerated		
p.Tyr44Cys	VUS	-2.08	Neutral	0.3951	Ambiguous	-9.38	Pathogenic	0.20	Neutral	1.00	Probably damaging	0.08	Tolerated	17.56	Deleterious
p.Tyr44Trp	VUS	-1.96	Neutral	0.3594	Ambiguous	-11.11	Pathogenic	0.29	Neutral	0.96	Probably damaging	0.12	Tolerated		
p.Tyr44Phe	VUS	-2.38	Neutral	0.1446	Benign	-3.70	Benign	0.18	Neutral	0.00	Benign	1	Tolerated	0.48	Neutral
p.Gly45Asn	VUS	-1.75	Neutral	0.7484	Pathogenic	-11.78	Pathogenic	0.77	Disease	1.00	Probably damaging	0.02	Deleterious		
p.Gly45Lys	VUS	-3.95	Neutral	0.877	Pathogenic	-15.08	Pathogenic	0.79	Disease	1.00	Probably damaging	0.12	Tolerated		
p.Gly45Thr	VUS	-1.80	Neutral	0.8377	Pathogenic	-13.43	Pathogenic	0.77	Disease	1.00	Probably damaging	0.01	Deleterious		
p.Gly45Arg	VUS	-0.50	Neutral	0.6263	Pathogenic	-12.97	Pathogenic	0.83	Disease	1.00	Probably damaging	0.02	Deleterious	26.00	Deleterious
p.Gly45Ser	VUS	-10.54	Indeterminate	0.4477	Ambiguous	-10.50	Pathogenic	0.83	Disease	1.00	Probably damaging	0.03	Deleterious	26.00	Deleterious
p.Gly45Ile	VUS	-13.67	Indeterminate	0.9659	Pathogenic	-18.24	Pathogenic	0.79	Disease	1.00	Probably damaging	0	Deleterious		
p.Gly45Met	VUS	-2.56	Neutral	0.9583	Pathogenic	-15.46	Pathogenic	0.79	Disease	1.00	Probably damaging	0	Deleterious		
p.Gly45His	VUS	-2.01	Neutral	0.7485	Pathogenic	-14.87	Pathogenic	0.77	Disease	1.00	Probably damaging	0.01	Deleterious		
p.Gly45Gln	VUS	-0.28	Neutral	0.6642	Pathogenic	-14.02	Pathogenic	0.78	Disease	1.00	Probably damaging	0.01	Deleterious		
p.Gly45Pro	VUS	-53.15	Deleterious	0.9945	Pathogenic	-18.63	Pathogenic	0.76	Disease	1.00	Probably damaging	0.01	Deleterious		
p.Gly45Leu	VUS	-0.82	Neutral	0.8676	Pathogenic	-14.32	Pathogenic	0.78	Disease	1.00	Probably damaging	0.01	Deleterious		
p.Gly45Asp	VUS	-0.26	Neutral	0.7037	Pathogenic	-10.05	Pathogenic	0.86	Disease	1.00	Probably damaging	0.02	Deleterious	25.50	Deleterious
p.Gly45Glu	VUS	-1.15	Neutral	0.8048	Pathogenic	-12.40	Pathogenic	0.88	Disease	1.00	Probably damaging	0.01	Deleterious		
p.Gly45Ala	VUS	-2.19	Neutral	0.5934	Pathogenic	-11.16	Pathogenic	0.83	Disease	1.00	Probably damaging	0.03	Deleterious	24.90	Deleterious
p.Gly45Val	VUS	-21.77	Indeterminate	0.9261	Pathogenic	-14.58	Pathogenic	0.87	Disease	1.00	Probably damaging	0.01	Deleterious	25.70	Deleterious
p.Gly45Tyr	VUS	-3.37	Neutral	0.8664	Pathogenic	-16.39	Pathogenic	0.80	Disease	1.00	Probably damaging	0	Deleterious		
p.Gly45Cys	VUS	-0.52	Neutral	0.7436	Pathogenic	-13.81	Pathogenic	0.89	Disease	1.00	Probably damaging	0	Deleterious	26.90	Deleterious
p.Gly45Trp	VUS	-3.99	Neutral	0.8636	Pathogenic	-14.99	Pathogenic	0.85	Disease	1.00	Probably damaging	0	Deleterious		
p.Gly45Phe	VUS	-0.09	Neutral	0.9352	Pathogenic	-16.79	Pathogenic	0.80	Disease	1.00	Probably damaging	0	Deleterious		
p.Arg46Asn	VUS	-0.71	Neutral	0.9806	Pathogenic	-15.60	Pathogenic	0.67	Disease	1.00	Probably damaging	0.18	Tolerated		
p.Arg46Lys	VUS	-0.41	Neutral	0.731	Pathogenic	-13.24	Pathogenic	0.48	Neutral	1.00	Probably damaging	0.58	Tolerated		
p.Arg46Thr	VUS	-33.07	Indeterminate	0.9723	Pathogenic	-15.00	Pathogenic	0.80	Disease	1.00	Probably damaging	0.08	Tolerated		
p.Arg46Ser	VUS	-0.32	Neutral	0.9811	Pathogenic	-13.34	Pathogenic	0.80	Disease	1.00	Probably damaging	0.06	Tolerated		
p.Arg46Ile	VUS	-53.15	Deleterious	0.9128	Pathogenic	-17.10	Pathogenic	0.74	Disease	1.00	Probably damaging	0.03	Deleterious		
p.Arg46Met	VUS	-10.71	Indeterminate	0.9584	Pathogenic	-16.82	Pathogenic	0.72	Disease	1.00	Probably damaging	0.02	Deleterious		
p.Arg46His	VUS	-0.53	Neutral	0.6345	Pathogenic	-13.33	Pathogenic	0.66	Disease	1.00	Probably damaging	0.06	Tolerated		
p.Arg46Gln	VUS	-14.28	Indeterminate	0.5586	Ambiguous	-13.16	Pathogenic	0.62	Disease	1.00	Probably damaging	0.16	Tolerated	26.20	Deleterious
p.Arg46Pro	VUS	-53.15	Deleterious	0.9714	Pathogenic	-16.65	Pathogenic	0.87	Disease	1.00	Probably damaging	0.05	Deleterious	25.50	Deleterious
p.Arg46Leu	VUS	-5.97	Indeterminate	0.7987	Pathogenic	-15.22	Pathogenic	0.81	Disease	1.00	Probably damaging	0.21	Tolerated	25.70	Deleterious
p.Arg46Asp	VUS	-53.15	Deleterious	0.9842	Pathogenic	-18.85	Pathogenic	0.77	Disease	1.00	Probably damaging	0.02	Deleterious		
p.Arg46Glu	VUS	-53.15	Deleterious	0.9098	Pathogenic	-17.42	Pathogenic	0.68	Disease	1.00	Probably damaging	0.1	Tolerated		
p.Arg46Ala	VUS	-10.45	Indeterminate	0.9674	Pathogenic	-13.76	Pathogenic	0.66	Disease	1.00	Probably damaging	0.07	Tolerated		
p.Arg46Gly	VUS	-6.54	Indeterminate	0.933	Pathogenic	-11.73	Pathogenic	0.82	Disease	1.00	Probably damaging	0.05	Deleterious	23.50	Deleterious
p.Arg46Val	VUS	-27.58	Indeterminate	0.9279	Pathogenic	-16.09	Pathogenic	0.73	Disease	1.00	Probably damaging	0.04	Deleterious		
p.Arg46Tyr	VUS	-14.34	Indeterminate	0.883	Pathogenic	-15.10	Pathogenic	0.70	Disease	1.00	Probably damaging	0.03	Deleterious		
p.Arg46Cys	VUS	-5.51	Neutral	0.8016	Pathogenic	-11.14	Pathogenic	0.82	Disease	1.00	Probably damaging	0.01	Deleterious		
p.Arg46Trp	VUS	-0.33	Neutral	0.6392	Pathogenic	-12.93	Pathogenic	0.80	Disease	1.00	Probably damaging	0.01	Deleterious	24.50	Deleterious
p.Arg46Phe	VUS	-1.84	Neutral	0.9478	Pathogenic	-15.17	Pathogenic	0.71	Disease	1.00	Probably damaging	0.02	Deleterious		
p.Arg47Asn	VUS	-2.20	Neutral	0.7485	Pathogenic	-9.56	Pathogenic	0.35	Neutral	0.79	Possibly damaging	0.13	Tolerated		
p.Arg47Lys	VUS	-2.24	Neutral	0.3095	Benign	-7.65	Pathogenic	0.14	Neutral	0.25	Benign	0.14	Tolerated	14.41	Neutral
p.Arg47Thr	VUS	-1.27	Neutral	0.3093	Benign	-5.10	Benign	0.15	Neutral	0.01	Benign	1	Tolerated	2.99	Neutral
p.Arg47Ser	VUS	-8.25	Indeterminate	0.5326	Ambiguous	-6.22	Benign	0.11	Neutral	0.05	Benign	0.52	Tolerated	9.65	Neutral
p.Arg47Ile	VUS	-3.02	Neutral	0.6194	Pathogenic	-11.61	Pathogenic	0.30	Neutral	0.98	Probably damaging	0.05	Deleterious		
p.Arg47Met	VUS	-1.94	Neutral	0.5667	Pathogenic	-7.27	Benign	0.33	Neutral	0.96	Probably damaging	0.03	Deleterious	18.80	Deleterious
p.Arg47His	VUS	-2.42	Neutral	0.2039	Benign	-5.44	Benign	0.19	Neutral	0.97	Probably damaging	0.03	Deleterious		
p.Arg47Gln	VUS	-10.65	Indeterminate	0.2172	Benign	-6.44	Benign	0.16	Neutral	0.87	Possibly damaging	0.08	Tolerated		
p.Arg47Pro	VUS	-11.61	Indeterminate	0.7548	Pathogenic	-8.24	Pathogenic	0.29	Neutral	0.99	Probably damaging	0.09	Tolerated		
p.Arg47Leu	VUS	-0.37	Neutral	0.3066	Benign	-5.62	Benign	0.24	Neutral	0.90	Possibly damaging	0.05	Deleterious		

p.Arg47Asp	VUS	-53.15	Deleterious	0.9177	Pathogenic	-15.01	Pathogenic	0.37	Neutral	0.98	Probably damaging	0.08	Tolerated		
p.Arg47Glu	VUS	-3.92	Neutral	0.748	Pathogenic	-13.09	Pathogenic	0.30	Neutral	0.53	Possibly damaging	0.06	Tolerated		
p.Arg47Ala	VUS	-2.77	Neutral	0.5277	Ambiguous	-7.58	Pathogenic	0.30	Neutral	0.45	Possibly damaging	0.18	Tolerated		
p.Arg47Gly	VUS	-9.18	Indeterminate	0.4602	Ambiguous	-10.59	Pathogenic	0.16	Neutral	0.86	Possibly damaging	0.07	Tolerated	16.41	Deleterious
p.Arg47Val	VUS	-1.23	Neutral	0.5757	Pathogenic	-9.79	Pathogenic	0.32	Neutral	0.94	Possibly damaging	0.08	Tolerated		
p.Arg47Tyr	VUS	-1.99	Neutral	0.5044	Ambiguous	-5.05	Benign	0.30	Neutral	0.85	Possibly damaging	0.02	Deleterious		
p.Arg47Cys	VUS	-0.31	Neutral	0.2597	Benign	-7.45	Benign	0.15	Neutral	1.00	Probably damaging	0.03	Deleterious		
p.Arg47Trp	VUS	-3.86	Neutral	0.2964	Benign	-8.90	Pathogenic	0.26	Neutral	0.98	Probably damaging	0.01	Deleterious	22.40	Deleterious
p.Arg47Phe	VUS	-0.20	Neutral	0.7065	Pathogenic	-7.03	Benign	0.32	Neutral	0.65	Possibly damaging	0.02	Deleterious		
p.Pro48Asn	VUS	-53.15	Deleterious	0.9787	Pathogenic	-17.26	Pathogenic	0.64	Disease	0.96	Possibly damaging	0.04	Deleterious		
p.Pro48Lys	VUS	-53.15	Deleterious	0.9943	Pathogenic	-20.62	Pathogenic	0.70	Disease	0.99	Probably damaging	0.06	Tolerated		
p.Pro48Thr	Pathogenic	-19.30	Indeterminate	0.8084	Pathogenic	-11.01	Pathogenic	0.61	Disease	0.69	Possibly damaging	0.11	Tolerated	16.24	Deleterious
p.Pro48Arg	VUS	-53.15	Deleterious	0.9677	Pathogenic	-16.85	Pathogenic	0.73	Disease	1.00	Probably damaging	0.04	Deleterious	25.60	Deleterious
p.Pro48Ser	VUS	-4.30	Neutral	0.6914	Pathogenic	-9.33	Pathogenic	0.57	Disease	0.93	Possibly damaging	0.29	Tolerated	16.81	Deleterious
p.Pro48Ile	VUS	-53.15	Deleterious	0.9751	Pathogenic	-16.41	Pathogenic	0.66	Disease	0.99	Probably damaging	0.04	Deleterious		
p.Pro48Met	VUS	-53.15	Deleterious	0.9761	Pathogenic	-15.41	Pathogenic	0.65	Disease	1.00	Probably damaging	0.03	Deleterious		
p.Pro48His	VUS	-53.15	Deleterious	0.9692	Pathogenic	-17.19	Pathogenic	0.64	Disease	1.00	Probably damaging	0.01	Deleterious		
p.Pro48Gln	VUS	-53.15	Deleterious	0.9466	Pathogenic	-16.15	Pathogenic	0.63	Disease	0.99	Probably damaging	0.04	Deleterious	26.00	Deleterious
p.Pro48Leu	VUS	-53.15	Deleterious	0.8968	Pathogenic	-13.24	Pathogenic	0.75	Disease	0.98	Probably damaging	0.06	Tolerated	26.60	Deleterious
p.Pro48Asp	VUS	-53.15	Deleterious	0.9925	Pathogenic	-18.20	Pathogenic	0.68	Disease	0.95	Possibly damaging	0.05	Deleterious		
p.Pro48Glu	VUS	-53.15	Deleterious	0.9801	Pathogenic	-16.52	Pathogenic	0.66	Disease	0.72	Possibly damaging	0.06	Tolerated		
p.Pro48Ala	VUS	-0.04	Neutral	0.314	Benign	-5.17	Benign	0.33	Neutral	0.01	Benign	1	Tolerated	7.25	Neutral
p.Pro48Gly	VUS	-14.99	Indeterminate	0.8399	Pathogenic	-12.29	Pathogenic	0.49	Neutral	0.93	Possibly damaging	0.31	Tolerated		
p.Pro48Val	VUS	-17.05	Indeterminate	0.8808	Pathogenic	-11.13	Pathogenic	0.63	Disease	0.68	Possibly damaging	0.11	Tolerated		
p.Pro48Tyr	VUS	-53.15	Deleterious	0.9952	Pathogenic	-19.09	Pathogenic	0.65	Disease	1.00	Probably damaging	0.02	Deleterious		
p.Pro48Cys	VUS	-29.75	Indeterminate	0.9269	Pathogenic	-12.01	Pathogenic	0.64	Disease	1.00	Probably damaging	0.06	Tolerated		
p.Pro48Trp	VUS	-53.15	Deleterious	0.9966	Pathogenic	-19.53	Pathogenic	0.65	Disease	1.00	Probably damaging	0.01	Deleterious		
p.Pro48Phe	VUS	-53.15	Deleterious	0.9958	Pathogenic	-17.68	Pathogenic	0.66	Disease	0.99	Probably damaging	0.02	Deleterious		
p.Ile49Asn	VUS	-53.15	Deleterious	0.9737	Pathogenic	-15.57	Pathogenic	0.87	Disease	1.00	Probably damaging	0	Deleterious	26.40	Deleterious
p.Ile49Lys	VUS	-53.15	Deleterious	0.9874	Pathogenic	-19.12	Pathogenic	0.84	Disease	0.98	Probably damaging	0	Deleterious		
p.Ile49Thr	Likely pathogenic	-29.95	Indeterminate	0.9628	Pathogenic	-12.04	Pathogenic	0.68	Disease	1.00	Probably damaging	0.01	Deleterious	25.70	Deleterious
p.Ile49Arg	VUS	-53.15	Deleterious	0.9636	Pathogenic	-15.14	Pathogenic	0.81	Disease	1.00	Probably damaging	0	Deleterious		
p.Ile49Ser	Likely pathogenic	-53.15	Deleterious	0.941	Pathogenic	-14.12	Pathogenic	0.87	Disease	1.00	Probably damaging	0	Deleterious	26.50	Deleterious
p.Ile49Met	VUS	-1.12	Neutral	0.4493	Ambiguous	-9.30	Pathogenic	0.61	Disease	1.00	Probably damaging	0.04	Deleterious	19.76	Deleterious
p.Ile49His	VUS	-53.15	Deleterious	0.9776	Pathogenic	-14.20	Pathogenic	0.79	Disease	1.00	Probably damaging	0	Deleterious		
p.Ile49Gln	VUS	-53.15	Deleterious	0.9677	Pathogenic	-13.76	Pathogenic	0.81	Disease	1.00	Probably damaging	0.01	Deleterious		
p.Ile49Pro	VUS	-53.15	Deleterious	0.9578	Pathogenic	-13.00	Pathogenic	0.77	Disease	1.00	Probably damaging	0	Deleterious		
p.Ile49Leu	VUS	-0.37	Neutral	0.2537	Benign	-1.42	Benign	0.30	Neutral	0.93	Possibly damaging	1	Tolerated	17.23	Deleterious
p.Ile49Asp	VUS	-53.15	Deleterious	0.9963	Pathogenic	-18.44	Pathogenic	0.79	Disease	1.00	Probably damaging	0	Deleterious		
p.Ile49Glu	VUS	-53.15	Deleterious	0.9845	Pathogenic	-17.50	Pathogenic	0.79	Disease	1.00	Probably damaging	0	Deleterious		
p.Ile49Ala	VUS	-29.55	Indeterminate	0.9073	Pathogenic	-13.41	Pathogenic	0.64	Disease	1.00	Probably damaging	0.01	Deleterious		
p.Ile49Gly	VUS	-53.15	Deleterious	0.9822	Pathogenic	-16.64	Pathogenic	0.73	Disease	1.00	Probably damaging	0	Deleterious		
p.Ile49Val	VUS	-0.79	Neutral	0.3085	Benign	-6.85	Benign	0.41	Neutral	0.95	Possibly damaging	0.06	Tolerated	19.03	Deleterious
p.Ile49Tyr	VUS	-53.15	Deleterious	0.9512	Pathogenic	-16.37	Pathogenic	0.69	Disease	0.99	Probably damaging	0	Deleterious		
p.Ile49Cys	VUS	-19.59	Indeterminate	0.9731	Pathogenic	-14.94	Pathogenic	0.69	Disease	1.00	Probably damaging	0	Deleterious		
p.Ile49Trp	VUS	-53.15	Deleterious	0.9727	Pathogenic	-16.39	Pathogenic	0.71	Disease	1.00	Probably damaging	0	Deleterious		
p.Ile49Phe	VUS	-27.97	Indeterminate	0.6763	Pathogenic	-11.84	Pathogenic	0.75	Disease	1.00	Probably damaging	0.03	Deleterious	24.40	Deleterious
p.Gln50Asn	VUS	-12.49	Indeterminate	0.8074	Pathogenic	-10.43	Pathogenic	0.70	Disease	1.00	Probably damaging	0.03	Deleterious		
p.Gln50Lys	VUS	-53.15	Deleterious	0.9228	Pathogenic	-17.58	Pathogenic	0.76	Disease	0.93	Possibly damaging	0.07	Tolerated	26.30	Deleterious
p.Gln50Thr	VUS	-10.88	Indeterminate	0.6796	Pathogenic	-13.74	Pathogenic	0.68	Disease	0.96	Probably damaging	0.02	Deleterious		
p.Gln50Arg	VUS	-53.15	Deleterious	0.84	Pathogenic	-14.09	Pathogenic	0.78	Disease	0.96	Possibly damaging	0.05	Deleterious	28.50	Deleterious
p.Gln50Ser	VUS	-5.39	Neutral	0.555	Ambiguous	-12.73	Pathogenic	0.68	Disease	1.00	Probably damaging	0.02	Deleterious		
p.Gln50Ile	VUS	-53.15	Deleterious	0.9391	Pathogenic	-17.14	Pathogenic	0.75	Disease	0.99	Probably damaging	0.01	Deleterious		
p.Gln50Met	VUS	-2.05	Neutral	0.8088	Pathogenic	-14.73	Pathogenic	0.70	Disease	0.99	Probably damaging	0	Deleterious		
p.Gln50His	VUS	-53.15	Deleterious	0.7198	Pathogenic	-3.60	Benign	0.75	Disease	0.99	Probably damaging	0.27	Tolerated	33.00	Deleterious
p.Gln50Pro	VUS	-53.15	Deleterious	0.7863	Pathogenic	-11.25	Pathogenic	0.86	Disease	1.00	Probably damaging	0.02	Deleterious	27.60	Deleterious
p.Gln50Leu	VUS	-19.95	Indeterminate	0.7182	Pathogenic	-13.31	Pathogenic	0.80	Disease	0.97	Probably damaging	0.01	Deleterious	29.80	Deleterious
p.Gln50Asp	VUS	-53.15	Deleterious	0.8569	Pathogenic	-12.67	Pathogenic	0.73	Disease	1.00	Probably damaging	0.03	Deleterious		
p.Gln50Glu	VUS	-1.37	Neutral	0.1941	Benign	-13.92	Pathogenic	0.74	Disease	1.00	Probably damaging	0.04	Deleterious	25.50	Deleterious
p.Gln50Ala	VUS	-10.39	Indeterminate	0.5825	Pathogenic	-13.36	Pathogenic	0.72	Disease	1.00	Probably damaging	0.02	Deleterious		
p.Gln50Gly	VUS	-13.64	Indeterminate	0.7192	Pathogenic	-12.93	Pathogenic	0.70	Disease	1.00	Probably damaging	0.03	Deleterious		
p.Gln50Val	VUS	-53.15	Deleterious	0.87	Pathogenic	-15.58	Pathogenic	0.74	Disease	1.00	Probably damaging	0.01	Deleterious		
p.Gln50Tyr	VUS	-53.15	Deleterious	0.9728	Pathogenic	-11.61	Pathogenic	0.76	Disease	1.00	Probably damaging	0.01	Deleterious		
p.Gln50Cys	VUS	-22.37	Indeterminate	0.9729	Pathogenic	-15.46	Pathogenic	0.75	Disease	1.00	Probably damaging	0	Deleterious		
p.Gln50Trp	VUS	-53.15	Deleterious	0.9737	Pathogenic	-15.75	Pathogenic	0.77	Disease	1.00	Probably damaging	0	Deleterious		
p.Gln50Phe	VUS	-53.15	Deleterious	0.9641	Pathogenic	-15.27	Pathogenic	0.75	Disease	1.00	Probably damaging	0	Deleterious		
p.Val51Asn	VUS	-53.15	Deleterious	0.8296	Pathogenic	-10.66	Pathogenic	0.78	Disease	1.00	Probably damaging	0.01	Deleterious		
p.Val51Lys	VUS	-53.15	Deleterious	0.9357	Pathogenic	-12.52	Pathogenic	0.81	Disease	1.00	Probably damaging	0.01	Deleterious		
p.Val51Thr	VUS	-4.90	Neutral	0.6936	Pathogenic	-7.68	Pathogenic	0.55	Disease	1.00	Probably damaging	0.03	Deleterious		
p.Val51Arg	VUS	-53.15	Deleterious	0.8385	Pathogenic	-11.71	Pathogenic	0.81	Disease	1.00	Probably damaging	0.01	Deleterious		
p.Val51Ser	VUS	-0.22	Neutral	0.7805	Pathogenic	-8.95	Pathogenic	0.73	Disease	1.00	Probably damaging	0.01	Deleterious		
p.Val51Ile	VUS	-5.58	Neutral	0.2785	Benign	-7.63	Pathogenic	0.53	Disease	0.96	Probably damaging	0.41	Tolerated	28.50	Deleterious
p.Val51Met	VUS	-53.15	Deleterious	0.7295	Pathogenic	-9.08	Pathogenic	0.68	Disease	1.00	Probably damaging	0.02	Deleterious		
p.Val51His	VUS	-53.15	Deleterious	0.9476	Pathogenic	-13.56	Pathogenic	0.80	Disease	1.00	Probably damaging	0	Deleterious		
p.Val51Gln	VUS	-53.15	Deleterious	0.8361	Pathogenic	-11.73	Pathogenic	0.78	Disease	1.00	Probably damaging	0.01	Deleterious		
p.Val51Pro	VUS	-53.15	Deleterious	0.9692	Pathogenic	-12.62	Pathogenic	0.80	Disease	1.00	Probably damaging	0.01	Deleterious		
p.Val51Leu	VUS	-1.84	Neutral	0.7283	Pathogenic	-8.30	Pathogenic	0.75	Disease	0.96	Probably damaging	0.11	Tolerated	31.00	Deleterious
p.Val51Asp	VUS	-53.15	Deleterious	0.9045	Pathogenic	-15.14	Pathogenic	0.88	Disease	1.00	Probably damaging	0.01	Deleterious	26.90	Deleterious
p.Val51Glu	VUS	-53.15	Deleterious	0.8206	Pathogenic	-14.07	Pathogenic	0.86	Disease	1.00	Probably damaging	0.01	Deleterious		
p.Val51Ala	VUS	-0.01	Neutral	0.6612	Pathogenic	-6.73	Benign	0.75	Disease	1.00	Probably damaging	0.04	Deleterious	26.30	Deleterious
p.Val51Gly	VUS	-3.26	Neutral	0.5831	Pathogenic	-8.94	Pathogenic	0.68	Disease	1.00	Probably damaging	0.01	Deleterious	26.40	Deleterious
p.Val51Tyr	VUS	-53.15	Deleterious	0.909	Pathogenic	-14.19	Pathogenic	0.77	Disease	1.00	Probably damaging	0.12	Tolerated		
p.Val51Cys	VUS	-0.37	Neutral	0.93	Pathogenic	-11.25	Pathogenic	0.76	Disease	1.00	Probably damaging	0.16	Tolerated		
p.Val51Trp	VUS	-53.15	Deleterious	0.9773	Pathogenic	-13.51	Pathogenic	0.76	Disease	1.00	Probably damaging	0	Deleterious		
p.Val51Phe	VUS	-53.15	Deleterious	0.7444	Pathogenic	-12.38	Pathogenic	0.81	Disease	1.00	Probably damaging	0.03	Deleterious	32.00	Deleterious
p.Met52Asn	VUS	-16.42	Indeterminate	0.9627	Pathogenic	-13.68	Pathogenic	0.72	Disease	1.00	Probably damaging	0.14	Tolerated		
p.Met52Lys	VUS	-53.15	Deleterious	0.954	Pathogenic	-14.14	Pathogenic	0.82	Disease	0.50	Possibly damaging	0.14	Tolerated	27.90	Deleterious
p.Met52Thr	VUS	-1.17	Neutral	0.7826	Pathogenic	-9.07	Pathogenic	0.77	Disease	0.82	Possibly damaging	0.5	Tolerated	26.40	Deleterious
p.Met52Arg	VUS	-53.15	Deleterious	0.9272	Pathogenic	-12.64	Pathogenic	0.82	Disease	0.68	Possibly damaging	0.1	Tolerated	28.50	Deleterious
p.Met52Ser	VUS	-0.07	Neutral	0.8548	Pathogenic	-11.63	Pathogenic	0.72	Disease	0.98	Probably damaging	0.72	Tolerated		
p.Met52Ile	VUS	-7.82	Indeterminate	0.9224	Pathogenic	-11.19	Pathogenic	0.74	Disease	0.09	Benign	0.16	Tolerated	24.97	Deleterious
p.Met52His	VUS	-4.19	Neutral	0.9567	Pathogenic	-12.75	Pathogenic	0.74	Disease	1.00	Probably damaging	0.04	Deleterious		
p.Met52Gln	VUS	-1.31	Neutral	0.7907	Pathogenic	-9.56	Pathogenic	0.70	Disease	0.99	Probably damaging	0.1	Tolerated		
p.Met52Pro	VUS	-13.05	Indeterminate	0.9812	Pathogenic	-11.77	Pathogenic	0.73	Disease	0.99	Probably damaging	0.26	Tolerated		
p.Met52Leu	VUS	-0.01	Neutral	0.3933	Ambiguous	-6.10	Benign	0.66	Disease	0.02	Benign	0.28	Tolerated	23.35	Deleterious
p.Met52Asp	VUS	-28.20	Indeterminate	0.9925	Pathogenic	-17.57	Pathogenic	0.71	Disease	0.99	Probably damaging	0.11	Tolerated		
p.Met52Glu	VUS	-11.11	Indeterminate	0.9593	Pathogenic	-14.71	Pathogenic	0.71	Disease	0.98	Probably damaging	0.11	Tolerated		
p.Met52Ala	VUS	-0.01	Neutral	0.6987	Pathogenic	-9.19	Pathogenic	0.72	Disease	0.54	Possibly damaging	1	Tolerated		
p.Met52Gly	VUS	0.00	Neutral	0.9223	Pathogenic	-13.23	Pathogenic	0.70	Disease	0.99	Probably damaging	0.5	Tolerated		
p.Met52Val	VUS	-0.01	Neutral	0.4159	Ambiguous	-8.75	Pathogenic	0.46	Neutral	0.20	Benign	0.32	Tolerated	22.80	Deleterious
p.Met52Tyr	VUS	-8.17	Indeterminate	0.9589	Pathogenic	-16.79	Pathogenic	0.70	Disease	0.93	Possibly damaging	0.07	Tolerated		
p.Met52Cys	VUS	-0.01	Neutral	0.8433	Pathogenic	-11.90	Pathogenic	0.70	Disease	1.00	Probably damaging	0.25	Tolerated		
p.Met52Trp	VUS	-9.17	Indeterminate	0.9696	Pathogenic	-14.81	Pathogenic	0.70	Disease	1.00	Probably damaging	0.03	Deleterious		
p.Met52Phe	VUS	-4.81	Neutral	0.7519	Pathogenic	-12.44	Pathogenic	0.66	Disease	0.14	Benign	0.09	Tolerated		
p.Met53Asn	VUS	-32.99	Indeterminate	0.9195	Pathogenic	-15.29	Pathogenic	0.72	Disease	1.00	Probably damaging	0.3	Tolerated		
p.Met53Lys	VUS	-8.88	Indeterminate	0.8127	Pathogenic	-11.47	Pathogenic	0.51	Disease	0.93	Possibly damaging	0.59	Tolerated	24.50	Deleterious
p.Met53Thr	VUS	-33.22	Indeterminate	0.8028	Pathogenic	-10.33	Pathogenic	0.76	Disease	1.00	Probably damaging	0.41	Tolerated	25.80	Deleterious
p.Met53Arg	VUS	-3.54	Neutral	0.6785	Pathogenic	-10.14	Pathogenic	0.58	Disease	0.99	Probably damaging	0.42	Tolerated	26.60	Deleterious
p.Met53Ser	VUS	-6.07	Indeterminate	0.8555	Pathogenic	-9.89	Pathogenic	0.72	Disease	1.00	Probably damaging	0.52	Tolerated		
p.Met53Ile	Pathogenic	-53.15	Deleterious	0.9254	Pathogenic	-10.44	Pathogenic	0.64	Disease	0.94	Possibly damaging	0.59	Tolerated	28.80	Deleterious
p.Met53His	VUS	-33.18	Indeterminate	0.889	Pathogenic	-13.75	Pathogenic	0.73	Disease	1.00	Probably damaging	0.48	Tolerated		

p.Met53Gln	VUS	-1.49	Neutral	0.4944	Ambiguous	-10.26	Pathogenic	0.49	Neutral	1.00	Probably damaging	0.31	Tolerated		
p.Met53Pro	VUS	-53.15	Deleterious	0.9676	Pathogenic	-10.04	Pathogenic	0.72	Disease	1.00	Probably damaging	0.2	Tolerated		
p.Met53Leu	VUS	-6.97	Indeterminate	0.3533	Ambiguous	-5.70	Benign	0.61	Disease	0.88	Possibly damaging	0.67	Tolerated	25.65	Deleterious
p.Met53Asp	VUS	-53.15	Deleterious	0.9768	Pathogenic	-16.24	Pathogenic	0.70	Disease	1.00	Probably damaging	0.2	Tolerated		
p.Met53Glu	VUS	-53.15	Deleterious	0.8544	Pathogenic	-13.12	Pathogenic	0.70	Disease	1.00	Probably damaging	0.29	Tolerated		
p.Met53Ala	VUS	-4.27	Neutral	0.7197	Pathogenic	-9.61	Pathogenic	0.69	Disease	1.00	Probably damaging	0.5	Tolerated		
p.Met53Gly	VUS	-53.15	Deleterious	0.8671	Pathogenic	-12.13	Pathogenic	0.70	Disease	0.55	Possibly damaging	0.31	Tolerated		
p.Met53Val	Likely pathogenic	-53.15	Deleterious	0.4637	Ambiguous	-8.22	Pathogenic	0.67	Disease	1.00	Probably damaging	0.51	Tolerated	25.00	Deleterious
p.Met53Tyr	VUS	-53.15	Deleterious	0.8966	Pathogenic	-14.30	Pathogenic	0.66	Disease	1.00	Probably damaging	1	Tolerated		
p.Met53Cys	VUS	-11.19	Indeterminate	0.9022	Pathogenic	-12.07	Pathogenic	0.72	Disease	1.00	Probably damaging	0.16	Tolerated		
p.Met53Trp	VUS	-53.15	Deleterious	0.9197	Pathogenic	-12.97	Pathogenic	0.67	Disease	1.00	Probably damaging	0.16	Tolerated		
p.Met53Phe	VUS	-53.15	Deleterious	0.647	Pathogenic	-12.53	Pathogenic	0.64	Disease	0.93	Possibly damaging	0.61	Tolerated		
p.Met54Asn	VUS	-1.00	Neutral	0.7165	Pathogenic	-10.22	Pathogenic	0.60	Disease	1.00	Probably damaging	0.1	Tolerated		
p.Met54Lys	VUS	-23.49	Indeterminate	0.5076	Ambiguous	-13.23	Pathogenic	0.71	Disease	0.93	Possibly damaging	0.16	Tolerated	28.70	Deleterious
p.Met54Thr	VUS	0.00	Neutral	0.6814	Pathogenic	-8.51	Pathogenic	0.58	Disease	0.98	Probably damaging	0.14	Tolerated	27.00	Deleterious
p.Met54Arg	VUS	-24.87	Indeterminate	0.3292	Benign	-12.14	Pathogenic	0.72	Disease	0.97	Probably damaging	0.12	Tolerated	29.00	Deleterious
p.Met54Ser	VUS	-0.03	Neutral	0.6661	Pathogenic	-9.69	Pathogenic	0.61	Disease	1.00	Probably damaging	0.13	Tolerated		
p.Met54Ile	VUS	-0.31	Neutral	0.8666	Pathogenic	-7.90	Pathogenic	0.35	Neutral	0.84	Possibly damaging	0.36	Tolerated	28.60	Deleterious
p.Met54His	VUS	-3.79	Neutral	0.584	Pathogenic	-10.15	Pathogenic	0.62	Disease	1.00	Probably damaging	0.14	Tolerated		
p.Met54Gln	VUS	-5.09	Neutral	0.2563	Benign	-10.50	Pathogenic	0.58	Disease	1.00	Probably damaging	0.11	Tolerated		
p.Met54Pro	VUS	-14.17	Indeterminate	0.8247	Pathogenic	-10.81	Pathogenic	0.62	Disease	1.00	Probably damaging	0.08	Tolerated		
p.Met54Leu	VUS	-0.08	Neutral	0.324	Benign	-5.24	Benign	0.30	Neutral	0.33	Benign	1	Tolerated	24.60	Deleterious
p.Met54Asp	VUS	-12.93	Indeterminate	0.8666	Pathogenic	-13.16	Pathogenic	0.59	Disease	1.00	Probably damaging	0.13	Tolerated		
p.Met54Glu	VUS	-14.17	Indeterminate	0.6117	Pathogenic	-13.35	Pathogenic	0.59	Disease	1.00	Probably damaging	0.11	Tolerated		
p.Met54Ala	VUS	-0.10	Neutral	0.5595	Ambiguous	-8.78	Pathogenic	0.59	Disease	0.94	Possibly damaging	0.19	Tolerated		
p.Met54Gly	VUS	-0.03	Neutral	0.6583	Pathogenic	-9.91	Pathogenic	0.60	Disease	1.00	Probably damaging	0.1	Tolerated		
p.Met54Val	VUS	-0.07	Neutral	0.4549	Ambiguous	-6.74	Benign	0.58	Disease	1.00	Probably damaging	0.27	Tolerated	26.20	Deleterious
p.Met54Tyr	VUS	-0.82	Neutral	0.6609	Pathogenic	-9.24	Pathogenic	0.59	Disease	1.00	Probably damaging	0.25	Tolerated		
p.Met54Cys	VUS	0.00	Neutral	0.8017	Pathogenic	-8.84	Pathogenic	0.60	Disease	1.00	Probably damaging	0.06	Tolerated		
p.Met54Trp	VUS	-0.70	Neutral	0.6948	Pathogenic	-13.35	Pathogenic	0.55	Disease	1.00	Probably damaging	0.06	Tolerated		
p.Met54Phe	VUS	-0.04	Neutral	0.4123	Ambiguous	-6.99	Benign	0.39	Neutral	0.93	Possibly damaging	0.35	Tolerated		
p.Gly55Asn	VUS	-53.15	Deleterious	0.9308	Pathogenic	-12.60	Pathogenic	0.76	Disease	1.00	Probably damaging	0.21	Tolerated		
p.Gly55Lys	VUS	-53.15	Deleterious	0.9741	Pathogenic	-14.48	Pathogenic	0.77	Disease	1.00	Probably damaging	0.01	Deleterious		
p.Gly55Thr	VUS	-53.15	Deleterious	0.9206	Pathogenic	-13.28	Pathogenic	0.77	Disease	1.00	Probably damaging	0.01	Deleterious		
p.Gly55Arg	VUS	-53.15	Deleterious	0.9318	Pathogenic	-10.66	Pathogenic	0.78	Disease	1.00	Probably damaging	0.01	Deleterious	30.00	Deleterious
p.Gly55Ser	VUS	-31.93	Indeterminate	0.7309	Pathogenic	-8.58	Pathogenic	0.75	Disease	1.00	Probably damaging	0.03	Deleterious	29.80	Deleterious
p.Gly55Ile	VUS	-53.15	Deleterious	0.9786	Pathogenic	-18.91	Pathogenic	0.84	Disease	1.00	Probably damaging	0	Deleterious		
p.Gly55Met	VUS	-53.15	Deleterious	0.987	Pathogenic	-18.38	Pathogenic	0.83	Disease	1.00	Probably damaging	0	Deleterious		
p.Gly55His	VUS	-53.15	Deleterious	0.971	Pathogenic	-14.25	Pathogenic	0.79	Disease	1.00	Probably damaging	0.01	Deleterious		
p.Gly55Gln	VUS	-53.15	Deleterious	0.9509	Pathogenic	-14.01	Pathogenic	0.77	Disease	1.00	Probably damaging	0.01	Deleterious		
p.Gly55Pro	VUS	-53.15	Deleterious	0.9783	Pathogenic	-12.38	Pathogenic	0.78	Disease	0.66	Possibly damaging	0.01	Deleterious		
p.Gly55Leu	VUS	-53.15	Deleterious	0.9734	Pathogenic	-17.29	Pathogenic	0.81	Disease	1.00	Probably damaging	0	Deleterious		
p.Gly55Asp	VUS	-53.15	Deleterious	0.9053	Pathogenic	-11.43	Pathogenic	0.81	Disease	1.00	Probably damaging	0.04	Deleterious	29.20	Deleterious
p.Gly55Glu	VUS	-53.15	Deleterious	0.9242	Pathogenic	-12.71	Pathogenic	0.83	Disease	1.00	Probably damaging	0.01	Deleterious		
p.Gly55Ala	VUS	-11.05	Indeterminate	0.6234	Pathogenic	-7.81	Pathogenic	0.79	Disease	0.99	Probably damaging	0.02	Deleterious	27.40	Deleterious
p.Gly55Val	VUS	-53.15	Deleterious	0.9375	Pathogenic	-15.51	Pathogenic	0.89	Disease	1.00	Probably damaging	0	Deleterious	28.90	Deleterious
p.Gly55Tyr	VUS	-53.15	Deleterious	0.9725	Pathogenic	-16.46	Pathogenic	0.82	Disease	1.00	Probably damaging	0	Deleterious		
p.Gly55Cys	VUS	-53.15	Deleterious	0.9277	Pathogenic	-12.03	Pathogenic	0.87	Disease	1.00	Probably damaging	0	Deleterious	32.00	Deleterious
p.Gly55Trp	VUS	-53.15	Deleterious	0.9668	Pathogenic	-16.50	Pathogenic	0.86	Disease	1.00	Probably damaging	0	Deleterious		
p.Gly55Phe	VUS	-53.15	Deleterious	0.9875	Pathogenic	-18.26	Pathogenic	0.84	Disease	1.00	Probably damaging	0	Deleterious		
p.Ser56Asn	VUS	0.00	Neutral	0.2869	Benign	-3.98	Benign	0.16	Neutral	0.03	Benign	0.9	Tolerated	12.86	Neutral
p.Ser56Lys	VUS	-0.10	Neutral	0.925	Pathogenic	-9.82	Pathogenic	0.50	Neutral	0.99	Probably damaging	0.95	Tolerated		
p.Ser56Thr	VUS	-0.47	Neutral	0.3245	Benign	-7.24	Benign	0.39	Neutral	0.68	Possibly damaging	0.62	Tolerated	17.51	Deleterious
p.Ser56Arg	VUS	0.00	Neutral	0.863	Pathogenic	-8.36	Pathogenic	0.56	Disease	1.00	Probably damaging	0.78	Tolerated	24.70	Deleterious
p.Ser56Ile	Pathogenic	-53.15	Deleterious	0.8369	Pathogenic	-14.63	Pathogenic	0.57	Disease	1.00	Probably damaging	0.26	Tolerated	22.80	Deleterious
p.Ser56Met	VUS	0.00	Neutral	0.7197	Pathogenic	-13.25	Pathogenic	0.54	Disease	1.00	Probably damaging	0.16	Tolerated		
p.Ser56His	VUS	0.00	Neutral	0.4565	Ambiguous	-5.87	Benign	0.33	Neutral	1.00	Probably damaging	0.37	Tolerated		
p.Ser56Gln	VUS	0.00	Neutral	0.7042	Pathogenic	-8.58	Pathogenic	0.48	Neutral	1.00	Probably damaging	0.62	Tolerated		
p.Ser56Pro	VUS	-53.15	Deleterious	0.8374	Pathogenic	-7.37	Benign	0.64	Disease	1.00	Probably damaging	0.29	Tolerated		
p.Ser56Leu	VUS	-17.53	Indeterminate	0.6522	Pathogenic	-10.78	Pathogenic	0.55	Disease	0.99	Probably damaging	0.45	Tolerated		
p.Ser56Asp	VUS	-0.20	Neutral	0.6454	Pathogenic	-8.35	Pathogenic	0.48	Neutral	0.92	Possibly damaging	0.62	Tolerated		
p.Ser56Glu	VUS	-3.79	Neutral	0.7859	Pathogenic	-9.46	Pathogenic	0.50	Neutral	0.97	Probably damaging	0.67	Tolerated		
p.Ser56Ala	VUS	-0.09	Neutral	0.2536	Benign	-8.17	Pathogenic	0.43	Neutral	0.80	Possibly damaging	0.64	Tolerated		
p.Ser56Gly	VUS	-2.34	Neutral	0.2343	Benign	-7.26	Benign	0.44	Neutral	0.39	Benign	0.52	Tolerated	25.70	Deleterious
p.Ser56Val	VUS	-2.86	Neutral	0.7218	Pathogenic	-11.82	Pathogenic	0.53	Disease	1.00	Probably damaging	0.36	Tolerated		
p.Ser56Tyr	VUS	-0.01	Neutral	0.5949	Pathogenic	-8.44	Pathogenic	0.53	Disease	1.00	Probably damaging	0.47	Tolerated		
p.Ser56Cys	VUS	0.00	Neutral	0.2089	Benign	-8.00	Pathogenic	0.30	Neutral	1.00	Probably damaging	0.12	Tolerated	27.30	Deleterious
p.Ser56Trp	VUS	-53.15	Deleterious	0.7018	Pathogenic	-10.80	Pathogenic	0.61	Disease	1.00	Probably damaging	0.1	Tolerated		
p.Ser56Phe	VUS	-0.08	Neutral	0.7895	Pathogenic	-10.42	Pathogenic	0.51	Disease	1.00	Probably damaging	0.42	Tolerated		
p.Ala57Asn	VUS	-0.57	Neutral	0.4468	Ambiguous	-8.31	Pathogenic	0.37	Neutral	0.56	Possibly damaging	0.46	Tolerated		
p.Ala57Lys	VUS	-2.18	Neutral	0.4994	Ambiguous	-10.63	Pathogenic	0.39	Neutral	0.92	Possibly damaging	0.87	Tolerated		
p.Ala57Thr	VUS	-3.45	Neutral	0.1004	Benign	-4.76	Benign	0.10	Neutral	0.01	Benign	0.56	Tolerated	5.39	Neutral
p.Ala57Arg	VUS	-0.24	Neutral	0.319	Benign	-7.61	Pathogenic	0.39	Neutral	0.98	Probably damaging	0.49	Tolerated		
p.Ala57Ser	VUS	-4.10	Neutral	0.1098	Benign	-5.17	Benign	0.20	Neutral	0.08	Benign	0.72	Tolerated	8.12	Neutral
p.Ala57Ile	VUS	-1.13	Neutral	0.3926	Ambiguous	-11.03	Pathogenic	0.29	Neutral	0.85	Possibly damaging	0.18	Tolerated		
p.Ala57Met	VUS	-3.27	Neutral	0.3899	Ambiguous	-10.89	Pathogenic	0.37	Neutral	0.99	Probably damaging	0.1	Tolerated		
p.Ala57His	VUS	-0.01	Neutral	0.4664	Ambiguous	-8.74	Pathogenic	0.38	Neutral	1.00	Probably damaging	0.12	Tolerated		
p.Ala57Gln	VUS	-1.67	Neutral	0.3253	Benign	-9.50	Pathogenic	0.37	Neutral	0.99	Probably damaging	0.56	Tolerated		
p.Ala57Pro	VUS	-4.88	Neutral	0.1227	Benign	-4.00	Benign	0.18	Neutral	0.02	Benign	0.3	Tolerated	7.88	Neutral
p.Ala57Leu	VUS	-9.30	Indeterminate	0.267	Benign	-8.91	Pathogenic	0.37	Neutral	0.71	Possibly damaging	0.29	Tolerated		
p.Ala57Asp	VUS	-0.12	Neutral	0.4412	Ambiguous	-10.51	Pathogenic	0.35	Neutral	0.70	Possibly damaging	0.62	Tolerated	18.96	Deleterious
p.Ala57Glu	VUS	-9.67	Indeterminate	0.2968	Benign	-10.32	Pathogenic	0.32	Neutral	0.81	Possibly damaging	1	Tolerated		
p.Ala57Gly	VUS	-1.14	Neutral	0.1392	Benign	-6.10	Benign	0.32	Neutral	0.57	Possibly damaging	0.36	Tolerated	14.86	Neutral
p.Ala57Val	Benign	-0.16	Neutral	0.1532	Benign	-7.22	Benign	0.15	Neutral	0.01	Benign	0.3	Tolerated	9.79	Neutral
p.Ala57Tyr	VUS	-15.97	Indeterminate	0.6368	Pathogenic	-9.29	Pathogenic	0.37	Neutral	0.99	Probably damaging	0.04	Deleterious		
p.Ala57Cys	VUS	-4.97	Neutral	0.471	Ambiguous	-8.78	Pathogenic	0.26	Neutral	1.00	Probably damaging	0.05	Deleterious		
p.Ala57Trp	VUS	-8.41	Indeterminate	0.6804	Pathogenic	-11.74	Pathogenic	0.42	Neutral	1.00	Probably damaging	0.02	Deleterious		
p.Ala57Phe	VUS	-15.14	Indeterminate	0.5515	Ambiguous	-11.25	Pathogenic	0.38	Neutral	0.98	Probably damaging	0.05	Deleterious		
p.Arg58Asn	VUS	0.00	Neutral	0.5027	Ambiguous	-7.88	Pathogenic	0.46	Neutral	0.96	Probably damaging	0.35	Tolerated		
p.Arg58Lys	VUS	0.00	Neutral	0.1612	Benign	-5.06	Benign	0.12	Neutral	0.36	Benign	0.69	Tolerated		
p.Arg58Thr	VUS	-0.10	Neutral	0.1907	Benign	-5.72	Benign	0.41	Neutral	0.96	Probably damaging	0.44	Tolerated		
p.Arg58Ser	VUS	0.00	Neutral	0.311	Benign	-4.61	Benign	0.17	Neutral	0.99	Probably damaging	0.51	Tolerated		
p.Arg58Ile	VUS	0.00	Neutral	0.3846	Ambiguous	-9.99	Pathogenic	0.50	Neutral	1.00	Probably damaging	0.13	Tolerated		
p.Arg58Met	VUS	0.00	Neutral	0.4241	Ambiguous	-8.79	Pathogenic	0.49	Neutral	0.99	Probably damaging	0.07	Tolerated		
p.Arg58His	VUS	0.00	Neutral	0.1485	Benign	-6.11	Benign	0.19	Neutral	0.01	Benign	0.13	Tolerated		
p.Arg58Gln	VUS	-0.05	Neutral	0.1117	Benign	-4.30	Benign	0.17	Neutral	0.29	Benign	0.47	Tolerated	12.76	Neutral
p.Arg58Pro	VUS	-0.22	Neutral	0.2054	Benign	-3.63	Benign	0.18	Neutral	0.37	Benign	0.19	Tolerated	12.91	Neutral
p.Arg58Leu	VUS	0.00	Neutral	0.1855	Benign	-6.61	Benign	0.47	Neutral	1.00	Probably damaging	0.21	Tolerated	14.45	Neutral
p.Arg58Asp	VUS	0.00	Neutral	0.3324	Benign	-4.88	Benign	0.48	Neutral	0.99	Probably damaging	0.54	Tolerated		
p.Arg58Glu	VUS	0.00	Neutral	0.203	Benign	-3.13	Benign	0.25	Neutral	0.59	Possibly damaging	1	Tolerated		
p.Arg58Ala	VUS	-0.03	Neutral	0.1563	Benign	-2.13	Benign	0.27	Neutral	0.90	Possibly damaging	0.72	Tolerated		
p.Arg58Gly	VUS	-0.04	Neutral	0.1835	Benign	-4.40	Benign	0.20	Neutral	1.00	Probably damaging	0.27	Tolerated	17.53	Deleterious
p.Arg58Val	VUS	0.00	Neutral	0.303	Benign	-6.25	Benign	0.52	Disease	0.99	Probably damaging	0.2	Tolerated		
p.Arg58Tyr	VUS	0.00	Neutral	0.498	Ambiguous	-9.91	Pathogenic	0.45	Neutral	0.69	Possibly damaging	0.03	Deleterious		
p.Arg58Cys	VUS	0.00	Neutral	0.3604	Ambiguous	-7.46	Benign	0.48	Neutral	1.00	Probably damaging	0.04	Deleterious		
p.Arg58Trp	VUS	0.00	Neutral	0.282	Benign	-9.55	Pathogenic	0.48	Neutral	1.00	Probably damaging	0.01	Deleterious		
p.Arg58Phe	VUS	-0.01	Neutral	0.5892	Pathogenic	-9.88	Pathogenic	0.52	Disease	0.82	Possibly damaging	0.04	Deleterious		
p.Val59Asn	VUS	-53.15	Deleterious	0.9093	Pathogenic	-15.23	Pathogenic	0.69	Disease	1.00	Probably damaging	0	Deleterious		
p.Val59Lys	VUS	-53.15	Deleterious	0.9586	Pathogenic	-16.15	Pathogenic	0.75	Disease	0.86	Possibly damaging	0	Deleterious		
p.Val59Thr	VUS	-0.21	Neutral	0.4091	Ambiguous	-8.59	Pathogenic	0.47	Neutral	1.00	Probably damaging	0.01	Deleterious		
p.Val59Arg	VUS	-53.15	Deleterious	0.9158	Pathogenic	-14.30	Pathogenic	0.75	Disease	0.26	Benign	0	Deleterious		

p.Val59Ser	VUS	-4.70	Neutral	0.7444	Pathogenic	-10.49	Pathogenic	0.68	Disease	1.00	Probably damaging	0	Deleterious		
p.Val59Ile	VUS	-0.16	Neutral	0.1519	Benign	-7.73	Pathogenic	0.25	Neutral	0.32	Benign	0.93	Tolerated		
p.Val59Met	VUS	-1.55	Neutral	0.6448	Pathogenic	-9.24	Pathogenic	0.42	Neutral	0.56	Possibly damaging	0.01	Deleterious	21.90	Deleterious
p.Val59His	VUS	-53.15	Deleterious	0.979	Pathogenic	-15.47	Pathogenic	0.68	Disease	1.00	Probably damaging	0	Deleterious		
p.Val59Gln	VUS	-53.15	Deleterious	0.905	Pathogenic	-14.47	Pathogenic	0.72	Disease	0.97	Probably damaging	0	Deleterious		
p.Val59Pro	VUS	-53.15	Deleterious	0.8617	Pathogenic	-12.37	Pathogenic	0.71	Disease	1.00	Probably damaging	0	Deleterious		
p.Val59Leu	VUS	-4.37	Neutral	0.6958	Pathogenic	-9.84	Pathogenic	0.34	Neutral	0.26	Benign	0.14	Tolerated	16.58	Deleterious
p.Val59Asp	VUS	-53.15	Deleterious	0.959	Pathogenic	-14.03	Pathogenic	0.79	Disease	1.00	Probably damaging	0	Deleterious		
p.Val59Glu	VUS	-53.15	Deleterious	0.8776	Pathogenic	-12.84	Pathogenic	0.82	Disease	1.00	Probably damaging	0	Deleterious	24.40	Deleterious
p.Val59Ala	VUS	-5.43	Neutral	0.4051	Ambiguous	-6.68	Benign	0.59	Disease	1.00	Probably damaging	0.01	Deleterious	23.80	Deleterious
p.Val59Gly	Pathogenic	-33.22	Indeterminate	0.6413	Pathogenic	-11.20	Pathogenic	0.60	Disease	1.00	Probably damaging	0	Deleterious	24.30	Deleterious
p.Val59Tyr	VUS	-53.15	Deleterious	0.9861	Pathogenic	-16.57	Pathogenic	0.69	Disease	1.00	Probably damaging	0.01	Deleterious		
p.Val59Cys	VUS	-0.94	Neutral	0.9254	Pathogenic	-8.71	Pathogenic	0.64	Disease	1.00	Probably damaging	0	Deleterious		
p.Val59Trp	VUS	-53.15	Deleterious	0.9946	Pathogenic	-17.04	Pathogenic	0.65	Disease	1.00	Probably damaging	0	Deleterious		
p.Val59Phe	VUS	-53.15	Deleterious	0.8967	Pathogenic	-15.45	Pathogenic	0.74	Disease	0.99	Probably damaging	0.01	Deleterious		
p.Ala60Asn	VUS	-53.15	Deleterious	0.9602	Pathogenic	-17.64	Pathogenic	0.72	Disease	0.98	Probably damaging	0.01	Deleterious		
p.Ala60Lys	VUS	-53.15	Deleterious	0.9921	Pathogenic	-19.21	Pathogenic	0.76	Disease	0.99	Probably damaging	0.01	Deleterious		
p.Ala60Thr	VUS	-1.18	Neutral	0.3475	Ambiguous	-7.22	Benign	0.62	Disease	0.99	Probably damaging	0.04	Deleterious	25.80	Deleterious
p.Ala60Arg	VUS	-53.15	Deleterious	0.9613	Pathogenic	-15.85	Pathogenic	0.73	Disease	1.00	Probably damaging	0.01	Deleterious		
p.Ala60Ser	VUS	-14.38	Indeterminate	0.3268	Benign	-8.42	Pathogenic	0.62	Disease	0.79	Possibly damaging	0.03	Deleterious	25.10	Deleterious
p.Ala60Ile	VUS	-22.52	Indeterminate	0.8372	Pathogenic	-11.44	Pathogenic	0.74	Disease	1.00	Probably damaging	0.04	Deleterious		
p.Ala60Met	VUS	-2.94	Neutral	0.7943	Pathogenic	-10.80	Pathogenic	0.68	Disease	1.00	Probably damaging	0.01	Deleterious		
p.Ala60His	VUS	-33.18	Indeterminate	0.9832	Pathogenic	-18.02	Pathogenic	0.74	Disease	1.00	Probably damaging	0	Deleterious		
p.Ala60Gln	VUS	-53.15	Deleterious	0.9314	Pathogenic	-16.75	Pathogenic	0.74	Disease	0.96	Probably damaging	0.01	Deleterious		
p.Ala60Pro	VUS	-18.86	Indeterminate	0.9469	Pathogenic	-10.86	Pathogenic	0.80	Disease	1.00	Probably damaging	0.02	Deleterious	25.70	Deleterious
p.Ala60Leu	VUS	-2.51	Neutral	0.6467	Pathogenic	-8.43	Pathogenic	0.69	Disease	1.00	Probably damaging	0.03	Deleterious		
p.Ala60Asp	VUS	-33.22	Indeterminate	0.9837	Pathogenic	-16.66	Pathogenic	0.84	Disease	1.00	Probably damaging	0.01	Deleterious		
p.Ala60Glu	Likely pathogenic	-33.22	Indeterminate	0.962	Pathogenic	-15.15	Pathogenic	0.79	Disease	0.99	Probably damaging	0.01	Deleterious	25.40	Deleterious
p.Ala60Gly	VUS	-1.98	Neutral	0.3107	Benign	-9.00	Pathogenic	0.62	Disease	1.00	Probably damaging	0.03	Deleterious	26.20	Deleterious
p.Ala60Val	VUS	-5.18	Neutral	0.4418	Ambiguous	-7.31	Benign	0.49	Neutral	1.00	Probably damaging	0.37	Tolerated	25.80	Deleterious
p.Ala60Tyr	VUS	-53.15	Deleterious	0.9901	Pathogenic	-17.64	Pathogenic	0.75	Disease	1.00	Probably damaging	0	Deleterious		
p.Ala60Cys	VUS	-7.26	Indeterminate	0.6622	Pathogenic	-8.74	Pathogenic	0.67	Disease	1.00	Probably damaging	0.01	Deleterious		
p.Ala60Trp	VUS	-53.15	Deleterious	0.9927	Pathogenic	-15.47	Pathogenic	0.79	Disease	1.00	Probably damaging	0	Deleterious		
p.Ala60Phe	VUS	-53.15	Deleterious	0.9672	Pathogenic	-13.94	Pathogenic	0.74	Disease	1.00	Probably damaging	0.01	Deleterious		
p.Glu61Asn	VUS	-1.02	Neutral	0.7007	Pathogenic	-10.25	Pathogenic	0.41	Neutral	0.73	Possibly damaging	0.48	Tolerated		
p.Glu61Lys	VUS	-0.20	Neutral	0.3723	Ambiguous	-9.29	Pathogenic	0.23	Neutral	0.12	Benign	0.89	Tolerated	11.10	Neutral
p.Glu61Thr	VUS	-0.31	Neutral	0.5214	Ambiguous	-8.09	Pathogenic	0.32	Neutral	0.79	Possibly damaging	0.52	Tolerated		
p.Glu61Arg	VUS	-4.80	Neutral	0.2715	Benign	-6.99	Benign	0.23	Neutral	0.00	Benign	0.52	Tolerated		
p.Glu61Ser	VUS	-1.62	Neutral	0.3724	Ambiguous	-6.91	Benign	0.29	Neutral	0.59	Possibly damaging	0.66	Tolerated		
p.Glu61Ile	VUS	-2.46	Neutral	0.8797	Pathogenic	-12.01	Pathogenic	0.50	Disease	0.75	Possibly damaging	0.17	Tolerated		
p.Glu61Met	VUS	-2.35	Neutral	0.8462	Pathogenic	-10.24	Pathogenic	0.54	Disease	0.62	Possibly damaging	0.1	Tolerated		
p.Glu61His	VUS	-1.51	Neutral	0.5801	Pathogenic	-8.60	Pathogenic	0.39	Neutral	0.82	Possibly damaging	0.11	Tolerated		
p.Glu61Gln	VUS	-0.25	Neutral	0.1632	Benign	-6.32	Benign	0.13	Neutral	0.02	Benign	0.53	Tolerated	7.74	Neutral
p.Glu61Pro	VUS	-53.15	Deleterious	0.9409	Pathogenic	-13.42	Pathogenic	0.52	Disease	0.86	Possibly damaging	0.25	Tolerated		
p.Glu61Leu	VUS	-0.27	Neutral	0.7588	Pathogenic	-8.93	Pathogenic	0.38	Neutral	0.29	Benign	0.31	Tolerated		
p.Glu61Asp	VUS	-0.83	Neutral	0.4425	Ambiguous	-7.21	Benign	0.33	Neutral	0.22	Benign	0.6	Tolerated	12.29	Neutral
p.Glu61Ala	VUS	-0.62	Neutral	0.2695	Benign	-6.58	Benign	0.19	Neutral	0.03	Benign	0.72	Tolerated	13.52	Neutral
p.Glu61Gly	VUS	-0.43	Neutral	0.2971	Benign	-8.01	Pathogenic	0.38	Neutral	0.02	Benign	0.34	Tolerated	18.67	Deleterious
p.Glu61Val	VUS	-0.35	Neutral	0.6779	Pathogenic	-9.71	Pathogenic	0.31	Neutral	0.18	Benign	0.26	Tolerated	17.51	Deleterious
p.Glu61Tyr	VUS	-4.64	Neutral	0.871	Pathogenic	-12.66	Pathogenic	0.50	Neutral	0.98	Probably damaging	0.04	Deleterious		
p.Glu61Cys	VUS	-0.54	Neutral	0.9517	Pathogenic	-10.31	Pathogenic	0.53	Disease	1.00	Probably damaging	0.05	Deleterious		
p.Glu61Trp	VUS	-1.42	Neutral	0.9523	Pathogenic	-11.63	Pathogenic	0.55	Disease	1.00	Probably damaging	0.02	Deleterious		
p.Glu61Phe	VUS	-2.00	Neutral	0.9411	Pathogenic	-12.68	Pathogenic	0.51	Disease	0.95	Possibly damaging	0.05	Deleterious		
p.Leu62Asn	VUS	0.00	Neutral	0.8298	Pathogenic	-13.59	Pathogenic	0.68	Disease	1.00	Probably damaging	0.09	Tolerated		
p.Leu62Lys	VUS	-1.24	Neutral	0.7361	Pathogenic	-12.18	Pathogenic	0.69	Disease	0.44	Benign	0.36	Tolerated		
p.Leu62Thr	VUS	-0.19	Neutral	0.5488	Ambiguous	-7.20	Benign	0.64	Disease	1.00	Probably damaging	0.14	Tolerated		
p.Leu62Arg	VUS	-28.58	Indeterminate	0.5057	Ambiguous	-10.44	Pathogenic	0.69	Disease	0.98	Probably damaging	0.31	Tolerated	24.20	Deleterious
p.Leu62Ser	VUS	-0.05	Neutral	0.8354	Pathogenic	-9.57	Pathogenic	0.70	Disease	1.00	Probably damaging	0.11	Tolerated		
p.Leu62Ile	VUS	-7.52	Indeterminate	0.1772	Benign	-7.87	Pathogenic	0.28	Neutral	0.99	Probably damaging	0.37	Tolerated		
p.Leu62Met	VUS	0.00	Neutral	0.2382	Benign	-7.24	Benign	0.43	Neutral	1.00	Probably damaging	0.21	Tolerated	23.00	Deleterious
p.Leu62His	VUS	0.00	Neutral	0.6562	Pathogenic	-8.78	Pathogenic	0.69	Disease	1.00	Probably damaging	0.08	Tolerated		
p.Leu62Gln	VUS	-29.45	Indeterminate	0.4796	Ambiguous	-7.67	Pathogenic	0.69	Disease	0.99	Probably damaging	0.17	Tolerated	25.70	Deleterious
p.Leu62Pro	VUS	-13.28	Indeterminate	0.9602	Pathogenic	-12.35	Pathogenic	0.83	Disease	1.00	Probably damaging	0.09	Tolerated	26.00	Deleterious
p.Leu62Asp	VUS	-0.04	Neutral	0.8998	Pathogenic	-9.24	Pathogenic	0.68	Disease	1.00	Probably damaging	0.07	Tolerated		
p.Leu62Glu	VUS	-0.08	Neutral	0.5869	Pathogenic	-4.51	Benign	0.68	Disease	1.00	Probably damaging	0.21	Tolerated		
p.Leu62Ala	VUS	-0.01	Neutral	0.5589	Ambiguous	-5.18	Benign	0.46	Neutral	1.00	Probably damaging	0.19	Tolerated		
p.Leu62Gly	VUS	-4.09	Neutral	0.8549	Pathogenic	-11.88	Pathogenic	0.63	Disease	1.00	Probably damaging	0.07	Tolerated		
p.Leu62Val	VUS	-0.18	Neutral	0.1734	Benign	-4.38	Benign	0.26	Neutral	0.95	Possibly damaging	0.29	Tolerated	19.78	Deleterious
p.Leu62Tyr	VUS	-8.39	Indeterminate	0.5464	Ambiguous	-8.73	Pathogenic	0.61	Disease	0.98	Probably damaging	0.08	Tolerated		
p.Leu62Cys	VUS	-5.15	Neutral	0.7386	Pathogenic	-8.67	Pathogenic	0.59	Disease	1.00	Probably damaging	0.03	Deleterious		
p.Leu62Trp	VUS	0.00	Neutral	0.543	Ambiguous	-10.97	Pathogenic	0.63	Disease	1.00	Probably damaging	0.03	Deleterious		
p.Leu62Phe	VUS	0.00	Neutral	0.4535	Ambiguous	-8.48	Pathogenic	0.47	Neutral	1.00	Probably damaging	0.19	Tolerated		
p.Leu63Asn	VUS	-53.15	Deleterious	0.9953	Pathogenic	-17.75	Pathogenic	0.84	Disease	1.00	Probably damaging	0	Deleterious		
p.Leu63Lys	VUS	-53.15	Deleterious	0.9902	Pathogenic	-18.24	Pathogenic	0.82	Disease	0.99	Probably damaging	0	Deleterious		
p.Leu63Thr	VUS	-53.15	Deleterious	0.9863	Pathogenic	-13.92	Pathogenic	0.84	Disease	1.00	Probably damaging	0	Deleterious		
p.Leu63Arg	VUS	-53.15	Deleterious	0.9585	Pathogenic	-13.96	Pathogenic	0.93	Disease	1.00	Probably damaging	0	Deleterious	27.60	Deleterious
p.Leu63Ser	VUS	-53.15	Deleterious	0.9945	Pathogenic	-12.87	Pathogenic	0.92	Disease	1.00	Probably damaging	0	Deleterious		
p.Leu63Ile	VUS	-5.21	Neutral	0.7278	Pathogenic	-10.80	Pathogenic	0.70	Disease	1.00	Probably damaging	0	Deleterious		
p.Leu63Met	VUS	-0.30	Neutral	0.7228	Pathogenic	-9.02	Pathogenic	0.76	Disease	1.00	Probably damaging	0	Deleterious	25.20	Deleterious
p.Leu63His	VUS	-53.15	Deleterious	0.9796	Pathogenic	-14.33	Pathogenic	0.91	Disease	1.00	Probably damaging	0	Deleterious		
p.Leu63Gln	VUS	-53.15	Deleterious	0.9753	Pathogenic	-14.73	Pathogenic	0.93	Disease	1.00	Probably damaging	0	Deleterious	27.10	Deleterious
p.Leu63Pro	VUS	-53.15	Deleterious	0.9779	Pathogenic	-11.69	Pathogenic	0.93	Disease	1.00	Probably damaging	0	Deleterious	27.80	Deleterious
p.Leu63Asp	VUS	-53.15	Deleterious	0.9988	Pathogenic	-17.88	Pathogenic	0.84	Disease	1.00	Probably damaging	0	Deleterious		
p.Leu63Glu	VUS	-53.15	Deleterious	0.9929	Pathogenic	-17.56	Pathogenic	0.85	Disease	1.00	Probably damaging	0	Deleterious		
p.Leu63Ala	VUS	-53.15	Deleterious	0.9699	Pathogenic	-12.36	Pathogenic	0.83	Disease	1.00	Probably damaging	0	Deleterious		
p.Leu63Gly	VUS	-53.15	Deleterious	0.9899	Pathogenic	-16.36	Pathogenic	0.86	Disease	1.00	Probably damaging	0	Deleterious		
p.Leu63Val	VUS	-53.15	Deleterious	0.7804	Pathogenic	-10.79	Pathogenic	0.83	Disease	0.99	Probably damaging	0	Deleterious	25.00	Deleterious
p.Leu63Tyr	VUS	-53.15	Deleterious	0.9691	Pathogenic	-15.35	Pathogenic	0.82	Disease	0.99	Probably damaging	0	Deleterious		
p.Leu63Cys	VUS	-53.15	Deleterious	0.9661	Pathogenic	-12.61	Pathogenic	0.79	Disease	1.00	Probably damaging	0	Deleterious		
p.Leu63Trp	VUS	-53.15	Deleterious	0.9618	Pathogenic	-14.79	Pathogenic	0.87	Disease	1.00	Probably damaging	0	Deleterious		
p.Leu63Phe	VUS	-33.17	Indeterminate	0.768	Pathogenic	-9.89	Pathogenic	0.81	Disease	1.00	Probably damaging	0	Deleterious		
p.Leu64Asn	VUS	-11.07	Indeterminate	0.9826	Pathogenic	-17.64	Pathogenic	0.80	Disease	1.00	Probably damaging	0	Deleterious		
p.Leu64Lys	VUS	-2.27	Neutral	0.9752	Pathogenic	-17.22	Pathogenic	0.80	Disease	0.99	Probably damaging	0	Deleterious		
p.Leu64Thr	VUS	-0.60	Neutral	0.8919	Pathogenic	-12.34	Pathogenic	0.83	Disease	1.00	Probably damaging	0.01	Deleterious		
p.Leu64Arg	VUS	-2.37	Neutral	0.89	Pathogenic	-13.68	Pathogenic	0.88	Disease	1.00	Probably damaging	0	Deleterious	27.70	Deleterious
p.Leu64Ser	VUS	-0.65	Neutral	0.9742	Pathogenic	-12.85	Pathogenic	0.90	Disease	1.00	Probably damaging	0	Deleterious		
p.Leu64Ile	VUS	-10.16	Indeterminate	0.2904	Benign	-7.11	Benign	0.55	Disease	1.00	Probably damaging	0.04	Deleterious		
p.Leu64Met	VUS	-1.56	Neutral	0.5884	Pathogenic	-8.33	Pathogenic	0.68	Disease	1.00	Probably damaging	0.02	Deleterious	25.30	Deleterious
p.Leu64His	VUS	-2.25	Neutral	0.9626	Pathogenic	-15.14	Pathogenic	0.86	Disease	1.00	Probably damaging	0	Deleterious		
p.Leu64Gln	VUS	-5.58	Neutral	0.944	Pathogenic	-13.81	Pathogenic	0.87	Disease	1.00	Probably damaging	0	Deleterious	27.20	Deleterious
p.Leu64Pro	VUS	-53.15	Deleterious	0.9709	Pathogenic	-13.51	Pathogenic	0.87	Disease	1.00	Probably damaging	0	Deleterious	27.80	Deleterious
p.Leu64Asp	VUS	-17.65	Indeterminate	0.997	Pathogenic	-17.53	Pathogenic	0.81	Disease	1.00	Probably damaging	0	Deleterious		
p.Leu64Glu	VUS	-7.32	Indeterminate	0.9826	Pathogenic	-16.64	Pathogenic	0.83	Disease	1.00	Probably damaging	0	Deleterious		
p.Leu64Ala	VUS	-1.79	Neutral	0.8713	Pathogenic	-11.12	Pathogenic	0.81	Disease	1.00	Probably damaging	0.01	Deleterious		
p.Leu64Gly	VUS	-4.23	Neutral	0.9676	Pathogenic	-13.74	Pathogenic	0.82	Disease	1.00	Probably damaging	0	Deleterious		
p.Leu64Val	VUS	-0.62	Neutral	0.3207	Benign	-8.37	Pathogenic	0.69	Disease	1.00	Probably damaging	0.15	Tolerated	25.00	Deleterious
p.Leu64Tyr	VUS	-1.41	Neutral	0.9719	Pathogenic	-15.94	Pathogenic	0.79	Disease	1.00	Probably damaging	0	Deleterious		
p.Leu64Cys	VUS	-0.83	Neutral	0.9069	Pathogenic	-10.92	Pathogenic	0.76	Disease	1.00	Probably damaging	0	Deleterious		
p.Leu64Trp	VUS	-1.00	Neutral	0.9526	Pathogenic	-14.38	Pathogenic	0.81	Disease	1.00	Probably damaging	0	Deleterious		
p.Leu64Phe	VUS	-0.19	Neutral	0.8411	Pathogenic	-11.58	Pathogenic	0.74	Disease	1.00	Probably damaging	0.02	Deleterious		
p.Leu65Asn	VUS	-12.04	Indeterminate	0.3576	Ambiguous	-7.55	Pathogenic	0.33	Neutral	1.00	Probably damaging	0.54	Tolerated		

p.Leu65Lys	VUS	-0.29	Neutral	0.1691	Benign	-5.40	Benign	0.32	Neutral	0.97	Probably damaging	1	Tolerated		
p.Leu65Thr	VUS	-3.35	Neutral	0.1612	Benign	-5.99	Benign	0.28	Neutral	1.00	Probably damaging	0.75	Tolerated		
p.Leu65Arg	VUS	-0.66	Neutral	0.0718	Benign	-2.33	Benign	0.24	Neutral	1.00	Probably damaging	0.6	Tolerated	17.13	Deleterious
p.Leu65Ser	VUS	-2.83	Neutral	0.1969	Benign	-4.68	Benign	0.21	Neutral	1.00	Probably damaging	0.86	Tolerated		
p.Leu65Ile	VUS	-1.69	Neutral	0.1806	Benign	-8.58	Pathogenic	0.21	Neutral	0.17	Benign	0.21	Tolerated	17.98	Deleterious
p.Leu65Met	VUS	-0.21	Neutral	0.1757	Benign	-7.29	Benign	0.36	Neutral	1.00	Probably damaging	0.12	Tolerated		
p.Leu65His	VUS	-10.93	Indeterminate	0.1732	Benign	-4.99	Benign	0.37	Neutral	1.00	Probably damaging	0.16	Tolerated	22.50	Deleterious
p.Leu65Gln	VUS	-3.84	Neutral	0.0746	Benign	-2.31	Benign	0.19	Neutral	1.00	Probably damaging	0.61	Tolerated		
p.Leu65Pro	Likely pathogenic	-53.15	Deleterious	0.7377	Pathogenic	-9.74	Pathogenic	0.31	Neutral	1.00	Probably damaging	0.3	Tolerated	22.60	Deleterious
p.Leu65Asp	VUS	-0.82	Neutral	0.4034	Ambiguous	-5.28	Benign	0.33	Neutral	1.00	Probably damaging	0.62	Tolerated		
p.Leu65Glu	VUS	-0.42	Neutral	0.1215	Benign	-3.10	Benign	0.26	Neutral	1.00	Probably damaging	0.98	Tolerated		
p.Leu65Ala	VUS	-4.46	Neutral	0.1044	Benign	-2.87	Benign	0.27	Neutral	1.00	Probably damaging	0.78	Tolerated		
p.Leu65Gly	VUS	-8.22	Indeterminate	0.2405	Benign	-5.06	Benign	0.43	Neutral	1.00	Probably damaging	0.4	Tolerated		
p.Leu65Val	VUS	-2.08	Neutral	0.1378	Benign	-6.10	Benign	0.20	Neutral	0.62	Possibly damaging	0.31	Tolerated	16.61	Deleterious
p.Leu65Tyr	VUS	-1.23	Neutral	0.3546	Ambiguous	-8.87	Pathogenic	0.38	Neutral	0.98	Probably damaging	0.09	Tolerated		
p.Leu65Cys	VUS	-13.13	Indeterminate	0.4305	Ambiguous	-6.24	Benign	0.30	Neutral	1.00	Probably damaging	0.07	Tolerated		
p.Leu65Trp	VUS	-7.73	Indeterminate	0.2329	Benign	-7.17	Benign	0.43	Neutral	1.00	Probably damaging	0.03	Deleterious		
p.Leu65Phe	VUS	-1.85	Neutral	0.1992	Benign	-9.10	Pathogenic	0.28	Neutral	0.99	Probably damaging	0.08	Tolerated	22.60	Deleterious
p.His66Asn	VUS	-0.16	Neutral	0.3884	Ambiguous	-7.58	Pathogenic	0.26	Neutral	1.00	Probably damaging	0.04	Deleterious	22.90	Deleterious
p.His66Lys	VUS	-0.18	Neutral	0.5891	Pathogenic	-6.91	Benign	0.30	Neutral	0.60	Possibly damaging	0.16	Tolerated		
p.His66Thr	VUS	-2.36	Neutral	0.7957	Pathogenic	-8.87	Pathogenic	0.49	Neutral	0.99	Probably damaging	0.05	Deleterious		
p.His66Arg	VUS	-1.93	Neutral	0.1748	Benign	-4.29	Benign	0.17	Neutral	0.01	Benign	0.41	Tolerated	22.90	Deleterious
p.His66Ser	VUS	-0.93	Neutral	0.5326	Ambiguous	-5.19	Benign	0.28	Neutral	1.00	Probably damaging	0.04	Deleterious		
p.His66Ile	VUS	-7.66	Indeterminate	0.8784	Pathogenic	-12.43	Pathogenic	0.51	Disease	0.99	Probably damaging	0.03	Deleterious		
p.His66Met	VUS	-0.24	Neutral	0.8768	Pathogenic	-8.28	Pathogenic	0.47	Neutral	1.00	Probably damaging	0.02	Deleterious		
p.His66Gln	VUS	-1.10	Neutral	0.4119	Ambiguous	-6.28	Benign	0.24	Neutral	0.99	Probably damaging	0.29	Tolerated	22.85	Deleterious
p.His66Pro	VUS	-53.15	Deleterious	0.7036	Pathogenic	-10.87	Pathogenic	0.51	Disease	1.00	Probably damaging	0.03	Deleterious	25.00	Deleterious
p.His66Leu	VUS	-1.55	Neutral	0.467	Ambiguous	-6.67	Benign	0.28	Neutral	0.70	Possibly damaging	0.06	Tolerated	23.40	Deleterious
p.His66Asp	VUS	-1.70	Neutral	0.7664	Pathogenic	-8.82	Pathogenic	0.47	Neutral	1.00	Probably damaging	0.02	Deleterious	24.00	Deleterious
p.His66Glu	VUS	-3.12	Neutral	0.6101	Pathogenic	-6.34	Benign	0.43	Neutral	1.00	Probably damaging	0.05	Deleterious		
p.His66Ala	VUS	-2.15	Neutral	0.4953	Ambiguous	-2.59	Benign	0.31	Neutral	1.00	Probably damaging	0.05	Deleterious		
p.His66Gly	VUS	-0.74	Neutral	0.5323	Ambiguous	-5.55	Benign	0.46	Neutral	1.00	Probably damaging	0.04	Deleterious		
p.His66Val	VUS	-5.55	Neutral	0.735	Pathogenic	-8.00	Pathogenic	0.46	Neutral	1.00	Probably damaging	0.04	Deleterious		
p.His66Tyr	VUS	-1.24	Neutral	0.1475	Benign	-5.71	Benign	0.25	Neutral	0.95	Possibly damaging	0.54	Tolerated	18.56	Deleterious
p.His66Cys	VUS	-0.01	Neutral	0.3504	Ambiguous	-7.06	Benign	0.46	Neutral	1.00	Probably damaging	0.01	Deleterious		
p.His66Trp	VUS	-0.25	Neutral	0.4002	Ambiguous	-6.01	Benign	0.50	Neutral	1.00	Probably damaging	0.02	Deleterious		
p.His66Phe	VUS	-0.70	Neutral	0.4537	Ambiguous	-7.03	Benign	0.44	Neutral	0.99	Probably damaging	0.05	Deleterious		
p.Gly67Asn	VUS	-0.13	Neutral	0.7348	Pathogenic	-10.55	Pathogenic	0.66	Disease	1.00	Probably damaging	0	Deleterious		
p.Gly67Lys	VUS	-0.88	Neutral	0.7226	Pathogenic	-10.78	Pathogenic	0.69	Disease	1.00	Probably damaging	0	Deleterious		
p.Gly67Thr	VUS	-9.71	Indeterminate	0.7928	Pathogenic	-13.00	Pathogenic	0.66	Disease	1.00	Probably damaging	0	Deleterious		
p.Gly67Arg	VUS	0.00	Neutral	0.5034	Ambiguous	-8.24	Pathogenic	0.76	Disease	1.00	Probably damaging	0	Deleterious	28.20	Deleterious
p.Gly67Ser	VUS	-0.07	Neutral	0.4193	Ambiguous	-9.72	Pathogenic	0.68	Disease	1.00	Probably damaging	0	Deleterious	27.90	Deleterious
p.Gly67Ile	VUS	-53.15	Deleterious	0.9677	Pathogenic	-17.56	Pathogenic	0.72	Disease	1.00	Probably damaging	0	Deleterious		
p.Gly67Met	VUS	-2.62	Neutral	0.9648	Pathogenic	-14.65	Pathogenic	0.73	Disease	1.00	Probably damaging	0	Deleterious		
p.Gly67His	VUS	-0.01	Neutral	0.687	Pathogenic	-9.44	Pathogenic	0.70	Disease	1.00	Probably damaging	0	Deleterious		
p.Gly67Gln	VUS	0.00	Neutral	0.6087	Pathogenic	-10.71	Pathogenic	0.67	Disease	1.00	Probably damaging	0	Deleterious		
p.Gly67Pro	VUS	-53.15	Deleterious	0.9938	Pathogenic	-15.59	Pathogenic	0.66	Disease	1.00	Probably damaging	0	Deleterious		
p.Gly67Leu	VUS	-0.43	Neutral	0.9284	Pathogenic	-12.85	Pathogenic	0.71	Disease	1.00	Probably damaging	0	Deleterious		
p.Gly67Asp	VUS	-0.22	Neutral	0.4936	Ambiguous	-9.88	Pathogenic	0.74	Disease	1.00	Probably damaging	0	Deleterious	26.10	Deleterious
p.Gly67Glu	VUS	-0.17	Neutral	0.7102	Pathogenic	-11.05	Pathogenic	0.76	Disease	1.00	Probably damaging	0	Deleterious		
p.Gly67Ala	VUS	0.00	Neutral	0.5522	Ambiguous	-9.56	Pathogenic	0.49	Neutral	1.00	Probably damaging	0	Deleterious	25.00	Deleterious
p.Gly67Val	VUS	-53.15	Deleterious	0.9212	Pathogenic	-13.26	Pathogenic	0.81	Disease	1.00	Probably damaging	0	Deleterious	25.90	Deleterious
p.Gly67Tyr	VUS	-0.17	Neutral	0.9176	Pathogenic	-13.91	Pathogenic	0.73	Disease	1.00	Probably damaging	0	Deleterious		
p.Gly67Cys	VUS	-1.96	Neutral	0.7922	Pathogenic	-11.77	Pathogenic	0.56	Disease	1.00	Probably damaging	0	Deleterious	29.30	Deleterious
p.Gly67Trp	VUS	-5.92	Indeterminate	0.9304	Pathogenic	-13.32	Pathogenic	0.75	Disease	1.00	Probably damaging	0	Deleterious		
p.Gly67Phe	VUS	-1.32	Neutral	0.9653	Pathogenic	-14.69	Pathogenic	0.72	Disease	1.00	Probably damaging	0	Deleterious		
p.Ala68Asn	VUS	-53.15	Deleterious	0.9679	Pathogenic	-18.24	Pathogenic	0.75	Disease	1.00	Probably damaging	0	Deleterious		
p.Ala68Lys	VUS	-53.15	Deleterious	0.9925	Pathogenic	-22.22	Pathogenic	0.78	Disease	1.00	Probably damaging	0	Deleterious		
p.Ala68Thr	VUS	-2.88	Neutral	0.5914	Pathogenic	-11.27	Pathogenic	0.73	Disease	1.00	Probably damaging	0	Deleterious	26.20	Deleterious
p.Ala68Arg	VUS	-53.15	Deleterious	0.9558	Pathogenic	-20.11	Pathogenic	0.76	Disease	1.00	Probably damaging	0	Deleterious		
p.Ala68Ser	VUS	-0.59	Neutral	0.293	Benign	-10.10	Pathogenic	0.54	Disease	0.99	Probably damaging	0	Deleterious	25.50	Deleterious
p.Ala68Ile	VUS	-53.15	Deleterious	0.9189	Pathogenic	-17.35	Pathogenic	0.78	Disease	1.00	Probably damaging	0	Deleterious		
p.Ala68Met	VUS	-33.17	Indeterminate	0.9229	Pathogenic	-16.44	Pathogenic	0.72	Disease	1.00	Probably damaging	0	Deleterious		
p.Ala68His	VUS	-24.88	Indeterminate	0.9743	Pathogenic	-19.04	Pathogenic	0.79	Disease	1.00	Probably damaging	0	Deleterious		
p.Ala68Gln	VUS	-53.15	Deleterious	0.9384	Pathogenic	-18.21	Pathogenic	0.77	Disease	1.00	Probably damaging	0	Deleterious		
p.Ala68Pro	VUS	-53.15	Deleterious	0.9539	Pathogenic	-15.22	Pathogenic	0.82	Disease	1.00	Probably damaging	0	Deleterious	26.30	Deleterious
p.Ala68Leu	Likely pathogenic	-53.15	Deleterious	0.8086	Pathogenic	-16.58	Pathogenic	0.76	Disease	1.00	Probably damaging	0	Deleterious		
p.Ala68Asp	VUS	-32.95	Indeterminate	0.9549	Pathogenic	-15.96	Pathogenic	0.84	Disease	1.00	Probably damaging	0	Deleterious		
p.Ala68Glu	VUS	-32.02	Indeterminate	0.9619	Pathogenic	-16.60	Pathogenic	0.81	Disease	1.00	Probably damaging	0	Deleterious	25.70	Deleterious
p.Ala68Gly	VUS	-1.38	Neutral	0.3176	Benign	-8.95	Pathogenic	0.62	Disease	1.00	Probably damaging	0	Deleterious	26.00	Deleterious
p.Ala68Val	VUS	-10.00	Indeterminate	0.676	Pathogenic	-10.83	Pathogenic	0.70	Disease	1.00	Probably damaging	0	Deleterious	26.10	Deleterious
p.Ala68Tyr	VUS	-53.15	Deleterious	0.9825	Pathogenic	-19.51	Pathogenic	0.78	Disease	1.00	Probably damaging	0	Deleterious		
p.Ala68Cys	VUS	-0.84	Neutral	0.7551	Pathogenic	-11.43	Pathogenic	0.70	Disease	1.00	Probably damaging	0	Deleterious		
p.Ala68Trp	VUS	-53.15	Deleterious	0.9882	Pathogenic	-19.71	Pathogenic	0.78	Disease	1.00	Probably damaging	0	Deleterious		
p.Ala68Phe	VUS	-53.15	Deleterious	0.9666	Pathogenic	-18.42	Pathogenic	0.79	Disease	1.00	Probably damaging	0	Deleterious		
p.Glu69Asn	VUS	-53.15	Deleterious	0.3183	Benign	-3.89	Benign	0.21	Neutral	0.04	Benign	1	Tolerated		
p.Glu69Lys	VUS	-33.00	Indeterminate	0.472	Ambiguous	-9.75	Pathogenic	0.25	Neutral	0.00	Benign	0.49	Tolerated	22.00	Deleterious
p.Glu69Thr	VUS	-9.93	Indeterminate	0.4956	Ambiguous	-8.39	Pathogenic	0.33	Neutral	0.11	Benign	0.22	Tolerated		
p.Glu69Arg	VUS	-1.75	Neutral	0.3922	Ambiguous	-8.31	Pathogenic	0.24	Neutral	0.01	Benign	0.18	Tolerated		
p.Glu69Ser	VUS	-13.93	Indeterminate	0.3272	Benign	-5.69	Benign	0.33	Neutral	0.04	Benign	0.51	Tolerated		
p.Glu69Ile	VUS	-3.57	Neutral	0.7085	Pathogenic	-11.52	Pathogenic	0.37	Neutral	0.09	Benign	0.06	Tolerated		
p.Glu69Met	VUS	-30.66	Indeterminate	0.7565	Pathogenic	-10.83	Pathogenic	0.38	Neutral	0.17	Benign	0.04	Deleterious		
p.Glu69His	VUS	-7.51	Indeterminate	0.5094	Ambiguous	-6.31	Benign	0.30	Neutral	0.13	Benign	0.09	Tolerated		
p.Glu69Gln	VUS	-6.51	Indeterminate	0.2181	Benign	-6.81	Benign	0.19	Neutral	0.01	Benign	0.25	Tolerated	20.60	Deleterious
p.Glu69Pro	VUS	-53.15	Deleterious	0.6727	Pathogenic	-10.70	Pathogenic	0.40	Neutral	0.06	Benign	0.13	Tolerated		
p.Glu69Leu	VUS	-33.22	Indeterminate	0.5672	Pathogenic	-8.78	Pathogenic	0.35	Neutral	0.01	Benign	0.1	Tolerated		
p.Glu69Asp	VUS	-5.22	Neutral	0.0782	Benign	0.95	Benign	0.09	Neutral	0.00	Benign	0.65	Tolerated	5.42	Neutral
p.Glu69Ala	VUS	-0.17	Neutral	0.2904	Benign	-6.89	Benign	0.27	Neutral	0.01	Benign	0.28	Tolerated	23.60	Deleterious
p.Glu69Gly	VUS	-13.03	Indeterminate	0.5256	Ambiguous	-8.23	Pathogenic	0.26	Neutral	0.36	Benign	0.27	Tolerated	24.00	Deleterious
p.Glu69Val	VUS	-25.39	Indeterminate	0.4395	Ambiguous	-9.19	Pathogenic	0.32	Neutral	0.01	Benign	0.09	Tolerated	24.10	Deleterious
p.Glu69Tyr	VUS	-8.93	Indeterminate	0.7874	Pathogenic	-8.99	Pathogenic	0.36	Neutral	0.36	Benign	0.04	Deleterious		
p.Glu69Cys	VUS	-5.94	Indeterminate	0.8994	Pathogenic	-7.55	Pathogenic	0.39	Neutral	1.00	Probably damaging	0.03	Deleterious		
p.Glu69Trp	VUS	-19.38	Indeterminate	0.9321	Pathogenic	-10.11	Pathogenic	0.40	Neutral	0.86	Possibly damaging	0.01	Deleterious		
p.Glu69Phe	VUS	-11.30	Indeterminate	0.8778	Pathogenic	-8.34	Pathogenic	0.38	Neutral	0.39	Benign	0.03	Deleterious		
p.Pro70Asn	VUS	-3.10	Neutral	0.9617	Pathogenic	-19.19	Pathogenic	0.66	Disease	1.00	Probably damaging	0.44	Tolerated		
p.Pro70Lys	VUS	-33.21	Indeterminate	0.8971	Pathogenic	-17.55	Pathogenic	0.71	Disease	1.00	Probably damaging	0.25	Tolerated		
p.Pro70Thr	VUS	-4.42	Neutral	0.4658	Ambiguous	-10.15	Pathogenic	0.61	Disease	1.00	Probably damaging	0.97	Tolerated	24.30	Deleterious
p.Pro70Arg	VUS	-0.46	Neutral	0.76	Pathogenic	-14.70	Pathogenic	0.71	Disease	1.00	Probably damaging	0.16	Tolerated	27.30	Deleterious
p.Pro70Ser	VUS	-32.63	Indeterminate	0.6826	Pathogenic	-12.10	Pathogenic	0.31	Neutral	1.00	Probably damaging	1	Tolerated	24.50	Deleterious
p.Pro70Ile	VUS	-0.38	Neutral	0.628	Pathogenic	-9.42	Pathogenic	0.67	Disease	1.00	Probably damaging	0.18	Tolerated		
p.Pro70Met	VUS	0.00	Neutral	0.8289	Pathogenic	-11.40	Pathogenic	0.67	Disease	1.00	Probably damaging	0.08	Tolerated		
p.Pro70His	VUS	0.00	Neutral	0.9162	Pathogenic	-15.45	Pathogenic	0.66	Disease	1.00	Probably damaging	0.09	Tolerated	27.90	Deleterious
p.Pro70Gln	VUS	0.00	Neutral	0.805	Pathogenic	-14.98	Pathogenic	0.63	Disease	1.00	Probably damaging	0.17	Tolerated		
p.Pro70Leu	VUS	-3.39	Neutral	0.6032	Pathogenic	-9.00	Pathogenic	0.68	Disease	1.00	Probably damaging	0.19	Tolerated	28.80	Deleterious
p.Pro70Asp	VUS	-53.15	Deleterious	0.9737	Pathogenic	-19.20	Pathogenic	0.70	Disease	1.00	Probably damaging	0.29	Tolerated		
p.Pro70Glu	VUS	-53.15	Deleterious	0.8773	Pathogenic	-17.44	Pathogenic	0.65	Disease	1.00	Probably damaging	0.22	Tolerated		
p.Pro70Ala	VUS	-2.01	Neutral	0.2232	Benign	-7.48	Benign	0.33	Neutral	1.00	Probably damaging	0.82	Tolerated	23.80	Deleterious
p.Pro70Gly	VUS	-2.54	Neutral	0.7794	Pathogenic	-11.94	Pathogenic	0.61	Disease	1.00	Probably damaging	0.44	Tolerated		
p.Pro70Val	VUS	-0.02	Neutral	0.363	Ambiguous	-5.39	Benign	0.64	Disease	1.00	Probably damaging	0.57	Tolerated		
p.Pro70Tyr	VUS	-1.90	Neutral	0.9689	Pathogenic	-17.90	Pathogenic	0.66	Disease	1.00	Probably damaging	0.09	Tolerated		
p.Pro70Cys	VUS	0.00	Neutral	0.8804	Pathogenic	-13.04	Pathogenic	0.66	Disease	1.00	Probably damaging	0.11	Tolerated		

p.Pro70Trp	VUS	-53.15	Deleterious	0.9728	Pathogenic	-16.09	Pathogenic	0.64	Disease	1.00	Probably damaging	0.03	Deleterious		
p.Pro70Phe	VUS	-0.11	Neutral	0.9674	Pathogenic	-15.59	Pathogenic	0.68	Disease	1.00	Probably damaging	0.08	Tolerated		
p.Asn71Lys	VUS	-53.15	Deleterious	0.9616	Pathogenic	-13.50	Pathogenic	0.76	Disease	1.00	Probably damaging	0.02	Deleterious	24.40	Deleterious
p.Asn71Thr	VUS	-53.15	Deleterious	0.5918	Pathogenic	-7.81	Pathogenic	0.69	Disease	0.98	Probably damaging	0.02	Deleterious	26.70	Deleterious
p.Asn71Arg	VUS	-53.15	Deleterious	0.9005	Pathogenic	-9.38	Pathogenic	0.69	Disease	1.00	Probably damaging	0.12	Tolerated		
p.Asn71Ser	Likely pathogenic	-53.15	Deleterious	0.2129	Benign	-6.58	Benign	0.67	Disease	1.00	Probably damaging	0.04	Deleterious	25.30	Deleterious
p.Asn71Ile	VUS	-53.15	Deleterious	0.9352	Pathogenic	-11.05	Pathogenic	0.83	Disease	1.00	Probably damaging	0	Deleterious	28.10	Deleterious
p.Asn71Met	VUS	-53.15	Deleterious	0.9288	Pathogenic	-9.36	Pathogenic	0.77	Disease	1.00	Probably damaging	0	Deleterious		
p.Asn71His	VUS	-20.64	Indeterminate	0.5902	Pathogenic	-8.69	Pathogenic	0.75	Disease	1.00	Probably damaging	0.02	Deleterious	25.90	Deleterious
p.Asn71Gln	VUS	-13.47	Indeterminate	0.8669	Pathogenic	-9.79	Pathogenic	0.67	Disease	1.00	Probably damaging	0.02	Deleterious		
p.Asn71Pro	VUS	-53.15	Deleterious	0.9656	Pathogenic	-11.37	Pathogenic	0.73	Disease	1.00	Probably damaging	0.01	Deleterious		
p.Asn71Leu	VUS	-53.15	Deleterious	0.8076	Pathogenic	-9.54	Pathogenic	0.74	Disease	1.00	Probably damaging	0	Deleterious		
p.Asn71Asp	VUS	-26.26	Indeterminate	0.223	Benign	-2.53	Benign	0.43	Neutral	1.00	Probably damaging	0.16	Tolerated	24.00	Deleterious
p.Asn71Glu	VUS	-12.42	Indeterminate	0.7821	Pathogenic	-6.87	Benign	0.63	Disease	0.99	Probably damaging	0.02	Deleterious		
p.Asn71Ala	VUS	-20.51	Indeterminate	0.7043	Pathogenic	-6.89	Benign	0.71	Disease	0.99	Probably damaging	0.02	Deleterious		
p.Asn71Gly	VUS	-1.93	Neutral	0.5961	Pathogenic	-7.24	Benign	0.67	Disease	1.00	Probably damaging	0.05	Deleterious		
p.Asn71Val	VUS	-53.15	Deleterious	0.8789	Pathogenic	-9.19	Pathogenic	0.75	Disease	0.98	Probably damaging	0	Deleterious		
p.Asn71Tyr	VUS	-53.15	Deleterious	0.7956	Pathogenic	-12.45	Pathogenic	0.82	Disease	1.00	Probably damaging	0.01	Deleterious	26.80	Deleterious
p.Asn71Cys	VUS	-16.50	Indeterminate	0.8283	Pathogenic	-5.76	Benign	0.73	Disease	1.00	Probably damaging	0	Deleterious		
p.Asn71Trp	VUS	-53.15	Deleterious	0.9803	Pathogenic	-11.43	Pathogenic	0.77	Disease	1.00	Probably damaging	0	Deleterious		
p.Asn71Phe	VUS	-53.15	Deleterious	0.9648	Pathogenic	-11.10	Pathogenic	0.76	Disease	1.00	Probably damaging	0	Deleterious		
p.Cys72Asn	VUS	-5.60	Neutral	0.4565	Ambiguous	-7.49	Benign	0.43	Neutral	0.79	Possibly damaging	0.27	Tolerated		
p.Cys72Lys	VUS	-6.71	Indeterminate	0.7865	Pathogenic	-9.86	Pathogenic	0.43	Neutral	0.67	Possibly damaging	0.51	Tolerated		
p.Cys72Thr	VUS	-3.40	Neutral	0.256	Benign	-3.64	Benign	0.25	Neutral	0.52	Possibly damaging	0.56	Tolerated		
p.Cys72Arg	VUS	-0.61	Neutral	0.3115	Benign	-6.41	Benign	0.11	Neutral	0.01	Benign	0.3	Tolerated	14.36	Neutral
p.Cys72Ser	VUS	-8.34	Indeterminate	0.2006	Benign	-3.02	Benign	0.35	Neutral	0.98	Probably damaging	0.41	Tolerated	16.24	Deleterious
p.Cys72Ile	VUS	-7.24	Indeterminate	0.3465	Ambiguous	-6.65	Benign	0.42	Neutral	0.97	Probably damaging	0.49	Tolerated		
p.Cys72Met	VUS	-3.24	Neutral	0.5647	Pathogenic	-6.74	Benign	0.43	Neutral	0.99	Probably damaging	0.16	Tolerated		
p.Cys72His	VUS	-10.13	Indeterminate	0.3639	Ambiguous	-5.64	Benign	0.42	Neutral	0.98	Probably damaging	0.17	Tolerated		
p.Cys72Gln	VUS	-12.58	Indeterminate	0.4744	Ambiguous	-5.57	Benign	0.41	Neutral	0.84	Possibly damaging	0.38	Tolerated		
p.Cys72Pro	VUS	-7.71	Indeterminate	0.7099	Pathogenic	-5.40	Benign	0.43	Neutral	0.92	Possibly damaging	0.22	Tolerated		
p.Cys72Leu	VUS	-19.97	Indeterminate	0.2665	Benign	-4.26	Benign	0.26	Neutral	0.80	Possibly damaging	0.55	Tolerated		
p.Cys72Asp	VUS	-1.91	Neutral	0.4226	Ambiguous	-4.46	Benign	0.46	Neutral	0.78	Possibly damaging	0.29	Tolerated		
p.Cys72Glu	VUS	-3.08	Neutral	0.5005	Ambiguous	-3.91	Benign	0.43	Neutral	0.84	Possibly damaging	0.48	Tolerated		
p.Cys72Ala	VUS	-4.81	Neutral	0.1581	Benign	-0.64	Benign	0.27	Neutral	0.97	Probably damaging	0.79	Tolerated		
p.Cys72Gly	VUS	-2.97	Neutral	0.134	Benign	-3.04	Benign	0.37	Neutral	0.99	Probably damaging	0.21	Tolerated	15.02	Deleterious
p.Cys72Val	VUS	-5.23	Neutral	0.2264	Benign	-3.23	Benign	0.21	Neutral	0.75	Possibly damaging	1	Tolerated		
p.Cys72Tyr	VUS	-0.68	Neutral	0.3384	Benign	-7.29	Benign	0.19	Neutral	0.99	Probably damaging	0.23	Tolerated	18.34	Deleterious
p.Cys72Trp	VUS	-11.02	Indeterminate	0.4589	Ambiguous	-8.48	Pathogenic	0.40	Neutral	1.00	Probably damaging	0.05	Deleterious	24.00	Deleterious
p.Cys72Phe	VUS	-8.51	Indeterminate	0.1717	Benign	-6.38	Benign	0.37	Neutral	0.99	Probably damaging	0.2	Tolerated	19.45	Deleterious
p.Ala73Asn	VUS	-7.83	Indeterminate	0.4928	Ambiguous	-12.71	Pathogenic	0.38	Neutral	0.50	Possibly damaging	0.3	Tolerated		
p.Ala73Lys	VUS	-2.90	Neutral	0.6144	Pathogenic	-15.55	Pathogenic	0.24	Neutral	0.82	Possibly damaging	0.65	Tolerated		
p.Ala73Thr	VUS	-0.56	Neutral	0.132	Benign	-6.87	Benign	0.17	Neutral	0.87	Possibly damaging	0.35	Tolerated	23.20	Deleterious
p.Ala73Arg	VUS	-4.04	Neutral	0.3606	Ambiguous	-9.94	Pathogenic	0.24	Neutral	0.97	Probably damaging	0.31	Tolerated		
p.Ala73Ser	VUS	-3.48	Neutral	0.1403	Benign	-5.74	Benign	0.30	Neutral	0.26	Benign	0.44	Tolerated	22.90	Deleterious
p.Ala73Ile	VUS	-1.38	Neutral	0.3864	Ambiguous	-10.33	Pathogenic	0.39	Neutral	0.99	Probably damaging	0.11	Tolerated		
p.Ala73Met	VUS	-2.96	Neutral	0.4562	Ambiguous	-10.25	Pathogenic	0.40	Neutral	1.00	Probably damaging	0.07	Tolerated		
p.Ala73His	VUS	-0.20	Neutral	0.4991	Ambiguous	-10.25	Pathogenic	0.39	Neutral	1.00	Probably damaging	0.09	Tolerated		
p.Ala73Gln	VUS	-5.57	Neutral	0.3172	Benign	-9.39	Pathogenic	0.21	Neutral	0.94	Possibly damaging	0.45	Tolerated		
p.Ala73Pro	VUS	-2.47	Neutral	0.1182	Benign	-4.70	Benign	0.16	Neutral	0.97	Probably damaging	0.23	Tolerated	20.90	Deleterious
p.Ala73Leu	VUS	-1.54	Neutral	0.2858	Benign	-7.24	Benign	0.38	Neutral	0.90	Possibly damaging	0.18	Tolerated		
p.Ala73Asp	VUS	-5.39	Neutral	0.3899	Ambiguous	-12.17	Pathogenic	0.40	Neutral	0.48	Possibly damaging	0.58	Tolerated	21.90	Deleterious
p.Ala73Glu	VUS	-1.95	Neutral	0.2439	Benign	-10.59	Pathogenic	0.16	Neutral	0.01	Benign	1	Tolerated		
p.Ala73Gly	VUS	-2.99	Neutral	0.1805	Benign	-6.60	Benign	0.19	Neutral	0.72	Possibly damaging	0.24	Tolerated	21.00	Deleterious
p.Ala73Val	VUS	-2.21	Neutral	0.1819	Benign	-6.67	Benign	0.20	Neutral	0.84	Possibly damaging	0.17	Tolerated	17.40	Deleterious
p.Ala73Tyr	VUS	-4.06	Neutral	0.6755	Pathogenic	-12.54	Pathogenic	0.42	Neutral	1.00	Probably damaging	0.03	Deleterious		
p.Ala73Cys	VUS	-2.99	Neutral	0.4259	Ambiguous	-8.85	Pathogenic	0.36	Neutral	1.00	Probably damaging	0.03	Deleterious		
p.Ala73Trp	VUS	-7.09	Indeterminate	0.7274	Pathogenic	-11.99	Pathogenic	0.48	Neutral	1.00	Probably damaging	0.01	Deleterious		
p.Ala73Phe	VUS	-3.00	Neutral	0.5232	Ambiguous	-10.97	Pathogenic	0.39	Neutral	1.00	Probably damaging	0.03	Deleterious		
p.Asp74Asn	VUS	-53.15	Deleterious	0.8381	Pathogenic	-9.62	Pathogenic	0.80	Disease	1.00	Probably damaging	0.17	Tolerated	31.00	Deleterious
p.Asp74Lys	VUS	-53.15	Deleterious	0.9891	Pathogenic	-14.48	Pathogenic	0.80	Disease	1.00	Probably damaging	0.01	Deleterious		
p.Asp74Thr	VUS	-53.15	Deleterious	0.9723	Pathogenic	-10.83	Pathogenic	0.79	Disease	1.00	Probably damaging	0.01	Deleterious		
p.Asp74Arg	VUS	-53.15	Deleterious	0.9747	Pathogenic	-13.09	Pathogenic	0.80	Disease	1.00	Probably damaging	0.01	Deleterious		
p.Asp74Ser	VUS	-12.50	Indeterminate	0.8258	Pathogenic	-11.42	Pathogenic	0.74	Disease	1.00	Probably damaging	0.02	Deleterious		
p.Asp74Ile	VUS	-53.15	Deleterious	0.9679	Pathogenic	-14.48	Pathogenic	0.80	Disease	1.00	Probably damaging	0	Deleterious		
p.Asp74Met	VUS	-53.15	Deleterious	0.9937	Pathogenic	-15.23	Pathogenic	0.81	Disease	1.00	Probably damaging	0	Deleterious		
p.Asp74His	VUS	-5.47	Neutral	0.8861	Pathogenic	-9.18	Pathogenic	0.90	Disease	1.00	Probably damaging	0.01	Deleterious	29.80	Deleterious
p.Asp74Gln	VUS	-53.15	Deleterious	0.9741	Pathogenic	-12.13	Pathogenic	0.79	Disease	1.00	Probably damaging	0.01	Deleterious		
p.Asp74Pro	VUS	-53.15	Deleterious	0.9834	Pathogenic	-14.64	Pathogenic	0.75	Disease	0.98	Probably damaging	0.01	Deleterious		
p.Asp74Leu	VUS	-53.15	Deleterious	0.95	Pathogenic	-13.73	Pathogenic	0.79	Disease	1.00	Probably damaging	0	Deleterious		
p.Asp74Glu	VUS	0.00	Neutral	0.867	Pathogenic	-7.60	Pathogenic	0.78	Disease	0.89	Possibly damaging	0.01	Deleterious	24.05	Deleterious
p.Asp74Ala	VUS	-53.15	Deleterious	0.8943	Pathogenic	-10.97	Pathogenic	0.85	Disease	1.00	Probably damaging	0.01	Deleterious	29.20	Deleterious
p.Asp74Gly	VUS	-53.15	Deleterious	0.9501	Pathogenic	-11.00	Pathogenic	0.88	Disease	1.00	Probably damaging	0.03	Deleterious	29.20	Deleterious
p.Asp74Val	VUS	-53.15	Deleterious	0.9289	Pathogenic	-12.81	Pathogenic	0.90	Disease	1.00	Probably damaging	0	Deleterious	29.00	Deleterious
p.Asp74Tyr	VUS	-53.15	Deleterious	0.8956	Pathogenic	-13.08	Pathogenic	0.90	Disease	1.00	Probably damaging	0	Deleterious	30.00	Deleterious
p.Asp74Cys	VUS	-0.01	Neutral	0.929	Pathogenic	-11.52	Pathogenic	0.79	Disease	1.00	Probably damaging	0	Deleterious		
p.Asp74Trp	VUS	-53.15	Deleterious	0.9936	Pathogenic	-14.65	Pathogenic	0.81	Disease	1.00	Probably damaging	0	Deleterious		
p.Asp74Phe	VUS	-53.15	Deleterious	0.9831	Pathogenic	-15.30	Pathogenic	0.82	Disease	1.00	Probably damaging	0	Deleterious		
p.Pro75Asn	VUS	-2.21	Neutral	0.6079	Pathogenic	-11.44	Pathogenic	0.44	Neutral	1.00	Probably damaging	0.68	Tolerated		
p.Pro75Lys	VUS	-4.73	Neutral	0.5067	Ambiguous	-10.14	Pathogenic	0.31	Neutral	1.00	Probably damaging	0.83	Tolerated		
p.Pro75Thr	VUS	-1.80	Neutral	0.2037	Benign	-8.88	Pathogenic	0.40	Neutral	1.00	Probably damaging	0.53	Tolerated	25.20	Deleterious
p.Pro75Arg	VUS	-0.73	Neutral	0.249	Benign	-7.82	Pathogenic	0.23	Neutral	1.00	Probably damaging	0.64	Tolerated	23.60	Deleterious
p.Pro75Ser	VUS	-1.67	Neutral	0.2505	Benign	-7.87	Pathogenic	0.17	Neutral	1.00	Probably damaging	0.57	Tolerated	24.00	Deleterious
p.Pro75Ile	VUS	-2.22	Neutral	0.5196	Ambiguous	-12.88	Pathogenic	0.52	Disease	1.00	Probably damaging	0.14	Tolerated		
p.Pro75Met	VUS	-4.48	Neutral	0.5736	Pathogenic	-12.25	Pathogenic	0.48	Neutral	1.00	Probably damaging	0.08	Tolerated		
p.Pro75His	VUS	-0.31	Neutral	0.3661	Ambiguous	-10.10	Pathogenic	0.43	Neutral	1.00	Probably damaging	0.13	Tolerated	26.30	Deleterious
p.Pro75Gln	VUS	-3.70	Neutral	0.3196	Benign	-9.25	Pathogenic	0.40	Neutral	1.00	Probably damaging	0.48	Tolerated		
p.Pro75Leu	VUS	-1.79	Neutral	0.2612	Benign	-9.65	Pathogenic	0.30	Neutral	1.00	Probably damaging	0.24	Tolerated	27.00	Deleterious
p.Pro75Asp	VUS	-1.93	Neutral	0.5776	Pathogenic	-10.89	Pathogenic	0.46	Neutral	1.00	Probably damaging	0.54	Tolerated		
p.Pro75Glu	VUS	-1.03	Neutral	0.3748	Ambiguous	-10.34	Pathogenic	0.43	Neutral	1.00	Probably damaging	1	Tolerated		
p.Pro75Ala	VUS	-1.00	Neutral	0.1356	Benign	-7.19	Benign	0.28	Neutral	1.00	Probably damaging	0.55	Tolerated	23.10	Deleterious
p.Pro75Gly	VUS	-1.33	Neutral	0.4257	Ambiguous	-8.31	Pathogenic	0.30	Neutral	1.00	Probably damaging	0.33	Tolerated		
p.Pro75Val	VUS	-1.31	Neutral	0.3043	Benign	-10.14	Pathogenic	0.44	Neutral	1.00	Probably damaging	0.21	Tolerated		
p.Pro75Tyr	VUS	-1.28	Neutral	0.7005	Pathogenic	-13.03	Pathogenic	0.47	Neutral	1.00	Probably damaging	0.07	Tolerated		
p.Pro75Cys	VUS	-1.58	Neutral	0.7905	Pathogenic	-11.33	Pathogenic	0.49	Neutral	1.00	Probably damaging	0.05	Deleterious		
p.Pro75Trp	VUS	-2.95	Neutral	0.7735	Pathogenic	-12.09	Pathogenic	0.52	Disease	1.00	Probably damaging	0.02	Deleterious		
p.Pro75Phe	VUS	-1.54	Neutral	0.7006	Pathogenic	-13.16	Pathogenic	0.50	Neutral	1.00	Probably damaging	0.06	Tolerated		
p.Ala76Asn	VUS	-13.40	Indeterminate	0.31	Benign	-4.57	Benign	0.29	Neutral	0.00	Benign	0.3	Tolerated		
p.Ala76Lys	VUS	-10.54	Indeterminate	0.4685	Ambiguous	-3.81	Benign	0.29	Neutral	0.80	Possibly damaging	0.74	Tolerated		
p.Ala76Thr	VUS	-2.83	Neutral	0.1157	Benign	-4.13	Benign	0.17	Neutral	0.04	Benign	0.66	Tolerated	15.16	Deleterious
p.Ala76Arg	VUS	-12.40	Indeterminate	0.3049	Benign	-2.13	Benign	0.24	Neutral	0.97	Probably damaging	0.31	Tolerated		
p.Ala76Ser	VUS	-7.14	Indeterminate	0.1173	Benign	-4.44	Benign	0.17	Neutral	0.03	Benign	0.51	Tolerated	14.18	Neutral
p.Ala76Ile	VUS	-20.17	Indeterminate	0.3135	Benign	-5.74	Benign	0.30	Neutral	0.67	Possibly damaging	0.11	Tolerated		
p.Ala76Met	VUS	-1.83	Neutral	0.3062	Benign	-7.02	Benign	0.30	Neutral	0.97	Probably damaging	0.06	Tolerated		
p.Ala76His	VUS	-4.71	Neutral	0.3524	Ambiguous	-5.19	Benign	0.24	Neutral	0.99	Probably damaging	0.07	Tolerated		
p.Ala76Gln	VUS	-16.22	Indeterminate	0.256	Benign	-5.01	Benign	0.28	Neutral	0.97	Probably damaging	0.35	Tolerated		
p.Ala76Pro	VUS	-3.41	Neutral	0.3909	Ambiguous	-7.15	Benign	0.28	Neutral	0.94	Possibly damaging	0.17	Tolerated	21.00	Deleterious
p.Ala76Leu	VUS	-9.05	Indeterminate	0.1986	Benign	-5.09	Benign	0.31	Neutral	0.45	Benign	0.18	Tolerated		
p.Ala76Asp	VUS	-4.79	Neutral	0.2923	Benign	-6.19	Benign	0.27	Neutral	0.00	Benign	0.38	Tolerated	14.35	Neutral
p.Ala76Glu	VUS	-8.61	Indeterminate	0.2402	Benign	-6.08	Benign	0.24	Neutral	0.43	Benign	0.82	Tolerated		
p.Ala76Gly	VUS	-2.53	Neutral	0.1588	Benign	-3.44	Benign	0.27	Neutral	0.32	Benign	0.35	Tolerated	13.15	Neutral

p.Ala76Val	VUS	-3.78	Neutral	0.1339	Benign	-3.22	Benign	0.16	Neutral	0.00	Benign	0.17	Tolerated	13.15	Neutral
p.Ala76Tyr	VUS	-3.09	Neutral	0.4651	Ambiguous	-7.67	Pathogenic	0.35	Neutral	0.97	Probably damaging	0.03	Deleterious		
p.Ala76Cys	VUS	-14.50	Indeterminate	0.4836	Ambiguous	-5.99	Benign	0.32	Neutral	0.99	Probably damaging	0.03	Deleterious		
p.Ala76Trp	VUS	-1.24	Neutral	0.6791	Pathogenic	-8.05	Pathogenic	0.37	Neutral	1.00	Probably damaging	0.01	Deleterious		
p.Ala76Phe	VUS	-6.82	Indeterminate	0.3737	Ambiguous	-8.80	Pathogenic	0.34	Neutral	0.93	Possibly damaging	0.03	Deleterious		
p.Thr77Asn	VUS	-4.52	Neutral	0.4401	Ambiguous	-3.96	Benign	0.53	Disease	1.00	Probably damaging	0.22	Tolerated	25.80	Deleterious
p.Thr77Lys	VUS	-4.40	Neutral	0.6986	Pathogenic	-9.95	Pathogenic	0.60	Disease	1.00	Probably damaging	0.15	Tolerated		
p.Thr77Arg	VUS	-12.26	Indeterminate	0.5249	Ambiguous	-9.09	Pathogenic	0.57	Disease	1.00	Probably damaging	0.1	Tolerated		
p.Thr77Ser	VUS	-6.48	Indeterminate	0.2652	Benign	-5.23	Benign	0.28	Neutral	0.99	Probably damaging	1	Tolerated	23.40	Deleterious
p.Thr77Ile	VUS	-2.46	Neutral	0.8141	Pathogenic	-9.67	Pathogenic	0.57	Disease	1.00	Probably damaging	0.09	Tolerated	26.80	Deleterious
p.Thr77Met	VUS	-6.23	Indeterminate	0.3921	Ambiguous	-9.21	Pathogenic	0.57	Disease	1.00	Probably damaging	0.05	Deleterious		
p.Thr77His	VUS	-4.95	Neutral	0.5887	Pathogenic	-6.65	Benign	0.57	Disease	1.00	Probably damaging	0.05	Deleterious		
p.Thr77Gln	VUS	-5.14	Neutral	0.6083	Pathogenic	-8.48	Pathogenic	0.55	Disease	1.00	Probably damaging	0.11	Tolerated		
p.Thr77Pro	VUS	-33.22	Indeterminate	0.5	Ambiguous	-12.09	Pathogenic	0.35	Neutral	1.00	Probably damaging	0.19	Tolerated	26.00	Deleterious
p.Thr77Leu	VUS	-5.03	Neutral	0.3219	Benign	-8.76	Pathogenic	0.56	Disease	0.99	Probably damaging	0.13	Tolerated		
p.Thr77Asp	VUS	-7.25	Indeterminate	0.7915	Pathogenic	-5.72	Benign	0.57	Disease	1.00	Probably damaging	0.15	Tolerated		
p.Thr77Glu	VUS	-16.56	Indeterminate	0.7672	Pathogenic	-7.61	Pathogenic	0.56	Disease	1.00	Probably damaging	0.12	Tolerated		
p.Thr77Ala	VUS	-6.61	Indeterminate	0.276	Benign	-6.63	Benign	0.51	Disease	0.98	Probably damaging	0.56	Tolerated	23.30	Deleterious
p.Thr77Gly	VUS	-1.41	Neutral	0.3456	Ambiguous	-7.91	Pathogenic	0.55	Disease	1.00	Probably damaging	0.3	Tolerated		
p.Thr77Val	VUS	-8.27	Indeterminate	0.5823	Pathogenic	-9.17	Pathogenic	0.48	Neutral	0.99	Probably damaging	0.16	Tolerated		
p.Thr77Tyr	VUS	-2.86	Neutral	0.7131	Pathogenic	-9.02	Pathogenic	0.59	Disease	1.00	Probably damaging	0.06	Tolerated		
p.Thr77Cys	VUS	-2.08	Neutral	0.7422	Pathogenic	-7.94	Pathogenic	0.58	Disease	1.00	Probably damaging	0.08	Tolerated		
p.Thr77Trp	VUS	-3.70	Neutral	0.8587	Pathogenic	-11.81	Pathogenic	0.59	Disease	1.00	Probably damaging	0.02	Deleterious		
p.Thr77Phe	VUS	-3.56	Neutral	0.7102	Pathogenic	-10.34	Pathogenic	0.58	Disease	1.00	Probably damaging	0.05	Deleterious		
p.Leu78Asn	VUS	0.00	Neutral	0.3776	Ambiguous	-8.32	Pathogenic	0.47	Neutral	1.00	Probably damaging	0.09	Tolerated		
p.Leu78Lys	VUS	-0.10	Neutral	0.2747	Benign	-7.82	Pathogenic	0.47	Neutral	0.99	Probably damaging	0.1	Tolerated		
p.Leu78Thr	VUS	-0.22	Neutral	0.3528	Ambiguous	-8.99	Pathogenic	0.45	Neutral	1.00	Probably damaging	0.14	Tolerated		
p.Leu78Arg	VUS	-16.95	Indeterminate	0.1211	Benign	-4.66	Benign	0.50	Neutral	1.00	Probably damaging	0.11	Tolerated	27.00	Deleterious
p.Leu78Ser	VUS	0.00	Neutral	0.3317	Benign	-5.84	Benign	0.42	Neutral	1.00	Probably damaging	0.13	Tolerated		
p.Leu78Ile	VUS	-0.32	Neutral	0.3375	Benign	-13.34	Pathogenic	0.34	Neutral	1.00	Probably damaging	0.38	Tolerated	18.47	Deleterious
p.Leu78Met	VUS	-11.97	Indeterminate	0.1891	Benign	-7.19	Benign	0.36	Neutral	1.00	Probably damaging	0.23	Tolerated		
p.Leu78His	VUS	-1.17	Neutral	0.2993	Benign	-6.42	Benign	0.44	Neutral	1.00	Probably damaging	0.15	Tolerated	27.00	Deleterious
p.Leu78Gln	VUS	-5.66	Neutral	0.1461	Benign	-6.01	Benign	0.45	Neutral	1.00	Probably damaging	0.1	Tolerated		
p.Leu78Pro	VUS	-10.97	Indeterminate	0.4528	Ambiguous	-11.56	Pathogenic	0.52	Disease	1.00	Probably damaging	0.08	Tolerated	27.50	Deleterious
p.Leu78Asp	VUS	0.00	Neutral	0.606	Pathogenic	-9.01	Pathogenic	0.49	Neutral	1.00	Probably damaging	0.06	Tolerated		
p.Leu78Glu	VUS	-5.41	Neutral	0.3396	Benign	-8.87	Pathogenic	0.48	Neutral	1.00	Probably damaging	0.09	Tolerated		
p.Leu78Ala	VUS	-14.32	Indeterminate	0.2132	Benign	-5.39	Benign	0.40	Neutral	1.00	Probably damaging	0.21	Tolerated		
p.Leu78Gly	VUS	0.00	Neutral	0.1888	Benign	-1.07	Benign	0.21	Neutral	1.00	Probably damaging	0.11	Tolerated		
p.Leu78Val	VUS	-0.08	Neutral	0.1929	Benign	-9.48	Pathogenic	0.34	Neutral	0.99	Probably damaging	0.3	Tolerated	18.18	Deleterious
p.Leu78Tyr	VUS	-3.31	Neutral	0.3986	Ambiguous	-10.11	Pathogenic	0.40	Neutral	0.99	Probably damaging	0.52	Tolerated		
p.Leu78Cys	VUS	-2.33	Neutral	0.5088	Ambiguous	-5.35	Benign	0.41	Neutral	1.00	Probably damaging	0.07	Tolerated		
p.Leu78Trp	VUS	-0.17	Neutral	0.3248	Benign	-10.06	Pathogenic	0.42	Neutral	1.00	Probably damaging	0.13	Tolerated		
p.Leu78Phe	VUS	-0.03	Neutral	0.2207	Benign	-9.28	Pathogenic	0.23	Neutral	1.00	Probably damaging	0.61	Tolerated	19.60	Deleterious
p.Thr79Asn	VUS	-0.32	Neutral	0.5472	Ambiguous	-10.60	Pathogenic	0.38	Neutral	0.93	Possibly damaging	0.04	Deleterious	25.30	Deleterious
p.Thr79Lys	VUS	-2.10	Neutral	0.773	Pathogenic	-13.86	Pathogenic	0.39	Neutral	0.85	Possibly damaging	0.02	Deleterious		
p.Thr79Arg	VUS	-12.64	Indeterminate	0.5924	Pathogenic	-10.01	Pathogenic	0.41	Neutral	0.98	Probably damaging	0.02	Deleterious		
p.Thr79Ser	VUS	-0.66	Neutral	0.2456	Benign	-3.94	Benign	0.12	Neutral	0.06	Benign	0.38	Tolerated	20.74	Deleterious
p.Thr79Ile	VUS	-0.34	Neutral	0.8117	Pathogenic	-6.63	Benign	0.17	Neutral	0.94	Possibly damaging	0.02	Deleterious	26.40	Deleterious
p.Thr79Met	VUS	-1.24	Neutral	0.5079	Ambiguous	-7.08	Benign	0.36	Neutral	1.00	Probably damaging	0.01	Deleterious		
p.Thr79His	VUS	-1.61	Neutral	0.6363	Pathogenic	-11.02	Pathogenic	0.40	Neutral	1.00	Probably damaging	0.01	Deleterious		
p.Thr79Gln	VUS	-0.84	Neutral	0.5363	Ambiguous	-8.94	Pathogenic	0.39	Neutral	0.95	Possibly damaging	0.02	Deleterious		
p.Thr79Pro	VUS	-53.15	Deleterious	0.6573	Pathogenic	-11.22	Pathogenic	0.34	Neutral	1.00	Probably damaging	0.03	Deleterious	24.80	Deleterious
p.Thr79Leu	VUS	-4.80	Neutral	0.48	Ambiguous	-5.73	Benign	0.40	Neutral	0.85	Possibly damaging	0.02	Deleterious		
p.Thr79Asp	VUS	-3.25	Neutral	0.8238	Pathogenic	-11.56	Pathogenic	0.41	Neutral	0.95	Possibly damaging	0.02	Deleterious		
p.Thr79Glu	VUS	-1.74	Neutral	0.7004	Pathogenic	-10.32	Pathogenic	0.41	Neutral	0.81	Possibly damaging	0.02	Deleterious		
p.Thr79Ala	VUS	-1.95	Neutral	0.3031	Benign	-4.35	Benign	0.09	Neutral	0.01	Benign	0.16	Tolerated	19.41	Deleterious
p.Thr79Gly	VUS	-2.54	Neutral	0.4236	Ambiguous	-8.45	Pathogenic	0.38	Neutral	1.00	Probably damaging	0.03	Deleterious		
p.Thr79Val	VUS	-1.05	Neutral	0.5979	Pathogenic	-3.11	Benign	0.28	Neutral	0.67	Possibly damaging	0.03	Deleterious		
p.Thr79Tyr	VUS	-4.54	Neutral	0.8001	Pathogenic	-9.48	Pathogenic	0.42	Neutral	1.00	Probably damaging	0.01	Deleterious		
p.Thr79Cys	VUS	-3.24	Neutral	0.7324	Pathogenic	-6.03	Benign	0.23	Neutral	1.00	Probably damaging	0.01	Deleterious		
p.Thr79Trp	VUS	-5.36	Neutral	0.8715	Pathogenic	-7.97	Pathogenic	0.42	Neutral	1.00	Probably damaging	0	Deleterious		
p.Thr79Phe	VUS	-1.91	Neutral	0.8125	Pathogenic	-8.81	Pathogenic	0.23	Neutral	0.99	Probably damaging	0.09	Tolerated		
p.Arg80Asn	VUS	-17.84	Indeterminate	0.8009	Pathogenic	-13.18	Pathogenic	0.46	Neutral	1.00	Probably damaging	0.47	Tolerated		
p.Arg80Lys	VUS	-4.55	Neutral	0.4406	Ambiguous	-11.68	Pathogenic	0.32	Neutral	0.99	Probably damaging	0.9	Tolerated		
p.Arg80Thr	VUS	-10.86	Indeterminate	0.4117	Ambiguous	-4.45	Benign	0.16	Neutral	1.00	Probably damaging	0.82	Tolerated		
p.Arg80Ser	VUS	-3.31	Neutral	0.6392	Pathogenic	-6.99	Benign	0.13	Neutral	1.00	Probably damaging	0.93	Tolerated		
p.Arg80Ile	VUS	-53.15	Deleterious	0.5159	Ambiguous	-12.71	Pathogenic	0.48	Neutral	1.00	Probably damaging	0.24	Tolerated		
p.Arg80Met	VUS	-12.61	Indeterminate	0.5624	Ambiguous	-9.13	Pathogenic	0.47	Neutral	0.99	Probably damaging	0.13	Tolerated		
p.Arg80His	VUS	-7.91	Indeterminate	0.2263	Benign	-7.35	Benign	0.15	Neutral	1.00	Probably damaging	0.18	Tolerated		
p.Arg80Gln	VUS	-17.50	Indeterminate	0.2243	Benign	-7.28	Benign	0.14	Neutral	1.00	Probably damaging	0.54	Tolerated	22.70	Deleterious
p.Arg80Pro	VUS	-53.15	Deleterious	0.8603	Pathogenic	-9.74	Pathogenic	0.23	Neutral	1.00	Probably damaging	0.31	Tolerated	24.50	Deleterious
p.Arg80Leu	VUS	-10.61	Indeterminate	0.2514	Benign	-7.89	Pathogenic	0.14	Neutral	0.87	Possibly damaging	0.4	Tolerated	22.30	Deleterious
p.Arg80Asp	VUS	-15.11	Indeterminate	0.9354	Pathogenic	-15.84	Pathogenic	0.50	Neutral	1.00	Probably damaging	0.51	Tolerated		
p.Arg80Glu	VUS	-4.74	Neutral	0.6812	Pathogenic	-13.85	Pathogenic	0.47	Neutral	1.00	Probably damaging	0.81	Tolerated		
p.Arg80Ala	VUS	-6.72	Indeterminate	0.5359	Ambiguous	-7.56	Pathogenic	0.27	Neutral	1.00	Probably damaging	1	Tolerated		
p.Arg80Gly	VUS	-7.39	Indeterminate	0.5276	Ambiguous	-9.90	Pathogenic	0.42	Neutral	1.00	Probably damaging	0.43	Tolerated	24.50	Deleterious
p.Arg80Val	VUS	-20.05	Indeterminate	0.4465	Ambiguous	-10.29	Pathogenic	0.49	Neutral	1.00	Probably damaging	0.38	Tolerated		
p.Arg80Tyr	VUS	-9.69	Indeterminate	0.4691	Ambiguous	-6.99	Benign	0.42	Neutral	0.99	Probably damaging	0.16	Tolerated		
p.Arg80Cys	VUS	-2.00	Neutral	0.2644	Benign	-8.00	Pathogenic	0.48	Neutral	1.00	Probably damaging	0.09	Tolerated		
p.Arg80Trp	VUS	-9.72	Indeterminate	0.2873	Benign	-9.49	Pathogenic	0.46	Neutral	1.00	Probably damaging	0.04	Deleterious		
p.Arg80Phe	VUS	-14.98	Indeterminate	0.6496	Pathogenic	-7.79	Pathogenic	0.34	Neutral	0.96	Possibly damaging	0.14	Tolerated		
p.Pro81Asn	VUS	-53.15	Deleterious	0.9894	Pathogenic	-18.84	Pathogenic	0.82	Disease	1.00	Probably damaging	0.02	Deleterious		
p.Pro81Lys	VUS	-53.15	Deleterious	0.9967	Pathogenic	-21.45	Pathogenic	0.85	Disease	1.00	Probably damaging	0.03	Deleterious		
p.Pro81Thr	VUS	-33.22	Indeterminate	0.8388	Pathogenic	-13.29	Pathogenic	0.85	Disease	1.00	Probably damaging	0.07	Tolerated	27.80	Deleterious
p.Pro81Arg	VUS	-53.15	Deleterious	0.9753	Pathogenic	-17.08	Pathogenic	0.93	Disease	1.00	Probably damaging	0.02	Deleterious	29.10	Deleterious
p.Pro81Ser	VUS	-53.15	Deleterious	0.8599	Pathogenic	-12.55	Pathogenic	0.82	Disease	1.00	Probably damaging	0.07	Tolerated	29.00	Deleterious
p.Pro81Ile	VUS	-53.15	Deleterious	0.9406	Pathogenic	-18.39	Pathogenic	0.81	Disease	1.00	Probably damaging	0.07	Tolerated		
p.Pro81Met	VUS	-53.15	Deleterious	0.9665	Pathogenic	-15.36	Pathogenic	0.83	Disease	1.00	Probably damaging	0.02	Deleterious		
p.Pro81His	VUS	-53.15	Deleterious	0.9778	Pathogenic	-18.01	Pathogenic	0.87	Disease	1.00	Probably damaging	0.01	Deleterious	29.60	Deleterious
p.Pro81Gln	VUS	-53.15	Deleterious	0.9612	Pathogenic	-16.67	Pathogenic	0.82	Disease	1.00	Probably damaging	0.02	Deleterious		
p.Pro81Leu	VUS	-53.15	Deleterious	0.8411	Pathogenic	-14.01	Pathogenic	0.90	Disease	1.00	Probably damaging	0.07	Tolerated	31.00	Deleterious
p.Pro81Asp	VUS	-53.15	Deleterious	0.994	Pathogenic	-19.16	Pathogenic	0.85	Disease	1.00	Probably damaging	0.02	Deleterious		
p.Pro81Glu	VUS	-53.15	Deleterious	0.9834	Pathogenic	-17.89	Pathogenic	0.81	Disease	1.00	Probably damaging	0.03	Deleterious		
p.Pro81Ala	VUS	-20.08	Indeterminate	0.4524	Ambiguous	-10.68	Pathogenic	0.71	Disease	1.00	Probably damaging	0.17	Tolerated	27.00	Deleterious
p.Pro81Gly	VUS	-53.15	Deleterious	0.9254	Pathogenic	-15.73	Pathogenic	0.77	Disease	1.00	Probably damaging	0.07	Tolerated		
p.Pro81Val	VUS	-33.22	Indeterminate	0.8183	Pathogenic	-15.02	Pathogenic	0.62	Disease	1.00	Probably damaging	0.31	Tolerated		
p.Pro81Tyr	VUS	-53.15	Deleterious	0.9942	Pathogenic	-20.20	Pathogenic	0.83	Disease	1.00	Probably damaging	0.01	Deleterious		
p.Pro81Cys	VUS	-19.66	Indeterminate	0.9474	Pathogenic	-14.43	Pathogenic	0.82	Disease	1.00	Probably damaging	0.02	Deleterious		
p.Pro81Trp	VUS	-53.15	Deleterious	0.996	Pathogenic	-20.71	Pathogenic	0.84	Disease	1.00	Probably damaging	0	Deleterious		
p.Pro81Phe	VUS	-53.15	Deleterious	0.994	Pathogenic	-18.68	Pathogenic	0.82	Disease	1.00	Probably damaging	0.02	Deleterious		
p.Val82Asn	VUS	-53.15	Deleterious	0.9718	Pathogenic	-16.39	Pathogenic	0.70	Disease	1.00	Probably damaging	0	Deleterious		
p.Val82Lys	VUS	-53.15	Deleterious	0.9855	Pathogenic	-18.37	Pathogenic	0.77	Disease	0.99	Probably damaging	0.01	Deleterious		
p.Val82Thr	VUS	-8.29	Indeterminate	0.7058	Pathogenic	-6.91	Benign	0.50	Neutral	1.00	Probably damaging	0.03	Deleterious		
p.Val82Arg	VUS	-53.15	Deleterious	0.9597	Pathogenic	-14.36	Pathogenic	0.74	Disease	1.00	Probably damaging	0	Deleterious		
p.Val82Ser	VUS	-7.82	Indeterminate	0.7613	Pathogenic	-8.13	Pathogenic	0.69	Disease	1.00	Probably damaging	0.01	Deleterious		
p.Val82Ile	VUS	-7.73	Indeterminate	0.1255	Benign	-7.88	Pathogenic	0.25	Neutral	0.77	Possibly damaging	0.6	Tolerated		
p.Val82Met	VUS	-1.59	Neutral	0.5783	Pathogenic	-8.20	Pathogenic	0.45	Neutral	1.00	Probably damaging	0.02	Deleterious	27.60	Deleterious
p.Val82His	VUS	-33.22	Indeterminate	0.9819	Pathogenic	-15.18	Pathogenic	0.68	Disease	1.00	Probably damaging	0	Deleterious		
p.Val82Gln	VUS	-13.11	Indeterminate	0.9388	Pathogenic	-12.30	Pathogenic	0.71	Disease	1.00	Probably damaging	0	Deleterious		
p.Val82Pro	VUS	-30.46	Indeterminate	0.8379	Pathogenic	-11.82	Pathogenic	0.68	Disease	1.00	Probably damaging	0.01	Deleterious		
p.Val82Leu	VUS	-5.30	Neutral	0.422	Ambiguous	-6.55	Benign	0.22	Neutral	0.76	Possibly damaging	0.53	Tolerated	22.70	Deleterious

p.Val82Asp	VUS	-53.15	Deleterious	0.9883	Pathogenic	-16.64	Pathogenic	0.79	Disease	1.00	Probably damaging	0	Deleterious		
p.Val82Glu	VUS	-33.21	Indeterminate	0.9406	Pathogenic	-13.40	Pathogenic	0.80	Disease	1.00	Probably damaging	0.01	Deleterious	27.70	Deleterious
p.Val82Ala	VUS	-2.05	Neutral	0.3786	Ambiguous	-1.31	Benign	0.32	Neutral	0.87	Possibly damaging	0.1	Tolerated	22.80	Deleterious
p.Val82Gly	VUS	-33.22	Indeterminate	0.6991	Pathogenic	-9.38	Pathogenic	0.55	Disease	1.00	Probably damaging	0	Deleterious	26.80	Deleterious
p.Val82Tyr	VUS	-53.15	Deleterious	0.9744	Pathogenic	-15.76	Pathogenic	0.67	Disease	1.00	Probably damaging	0.01	Deleterious		
p.Val82Cys	VUS	-13.90	Indeterminate	0.8291	Pathogenic	-10.10	Pathogenic	0.64	Disease	1.00	Probably damaging	0.01	Deleterious		
p.Val82Trp	VUS	-53.15	Deleterious	0.9889	Pathogenic	-14.31	Pathogenic	0.64	Disease	1.00	Probably damaging	0	Deleterious		
p.Val82Phe	VUS	-21.29	Indeterminate	0.7499	Pathogenic	-13.04	Pathogenic	0.64	Disease	1.00	Probably damaging	0.02	Deleterious		
p.His83Asn	VUS	-53.15	Deleterious	0.7844	Pathogenic	-16.37	Pathogenic	0.91	Disease	1.00	Probably damaging	0	Deleterious	29.40	Deleterious
p.His83Lys	VUS	-53.15	Deleterious	0.9871	Pathogenic	-18.73	Pathogenic	0.84	Disease	0.97	Probably damaging	0	Deleterious		
p.His83Thr	VUS	-53.15	Deleterious	0.96	Pathogenic	-15.15	Pathogenic	0.84	Disease	1.00	Probably damaging	0	Deleterious		
p.His83Arg	Pathogenic	-53.15	Deleterious	0.9431	Pathogenic	-14.58	Pathogenic	0.90	Disease	0.96	Probably damaging	0	Deleterious	27.10	Deleterious
p.His83Ser	VUS	-53.15	Deleterious	0.9106	Pathogenic	-15.71	Pathogenic	0.83	Disease	1.00	Probably damaging	0	Deleterious		
p.His83Ile	VUS	-53.15	Deleterious	0.9602	Pathogenic	-14.15	Pathogenic	0.87	Disease	0.99	Probably damaging	0	Deleterious		
p.His83Met	VUS	-30.49	Indeterminate	0.9481	Pathogenic	-12.73	Pathogenic	0.87	Disease	1.00	Probably damaging	0	Deleterious		
p.His83Gln	VUS	-53.15	Deleterious	0.8794	Pathogenic	-10.99	Pathogenic	0.88	Disease	1.00	Probably damaging	0	Deleterious	25.70	Deleterious
p.His83Pro	VUS	-53.15	Deleterious	0.8982	Pathogenic	-12.25	Pathogenic	0.90	Disease	1.00	Probably damaging	0	Deleterious	28.50	Deleterious
p.His83Leu	VUS	-53.15	Deleterious	0.8031	Pathogenic	-12.52	Pathogenic	0.90	Disease	0.99	Probably damaging	0	Deleterious	29.40	Deleterious
p.His83Asp	Likely pathogenic	-53.15	Deleterious	0.8932	Pathogenic	-14.15	Pathogenic	0.90	Disease	1.00	Probably damaging	0	Deleterious	29.80	Deleterious
p.His83Glu	VUS	-53.15	Deleterious	0.937	Pathogenic	-13.40	Pathogenic	0.84	Disease	1.00	Probably damaging	0	Deleterious		
p.His83Ala	VUS	-33.22	Indeterminate	0.9429	Pathogenic	-12.39	Pathogenic	0.82	Disease	1.00	Probably damaging	0	Deleterious		
p.His83Gly	VUS	-53.15	Deleterious	0.9562	Pathogenic	-14.62	Pathogenic	0.83	Disease	1.00	Probably damaging	0	Deleterious		
p.His83Val	VUS	-53.15	Deleterious	0.9547	Pathogenic	-13.93	Pathogenic	0.87	Disease	1.00	Probably damaging	0	Deleterious		
p.His83Tyr	Likely pathogenic	-53.15	Deleterious	0.7859	Pathogenic	-14.36	Pathogenic	0.90	Disease	0.97	Probably damaging	0	Deleterious	29.40	Deleterious
p.His83Cys	VUS	-53.15	Deleterious	0.7785	Pathogenic	-14.26	Pathogenic	0.82	Disease	1.00	Probably damaging	0	Deleterious		
p.His83Trp	VUS	-53.15	Deleterious	0.8026	Pathogenic	-16.77	Pathogenic	0.86	Disease	1.00	Probably damaging	0	Deleterious		
p.His83Phe	VUS	-53.15	Deleterious	0.8484	Pathogenic	-14.90	Pathogenic	0.86	Disease	0.99	Probably damaging	0	Deleterious		
p.Asp84Asn	Likely pathogenic	-53.15	Deleterious	0.8357	Pathogenic	-11.47	Pathogenic	0.71	Disease	1.00	Probably damaging	0.52	Tolerated	32.00	Deleterious
p.Asp84Lys	VUS	-53.15	Deleterious	0.9685	Pathogenic	-16.42	Pathogenic	0.78	Disease	1.00	Probably damaging	0.45	Tolerated		
p.Asp84Thr	VUS	-53.15	Deleterious	0.9381	Pathogenic	-12.78	Pathogenic	0.77	Disease	1.00	Probably damaging	0.36	Tolerated		
p.Asp84Arg	VUS	-53.15	Deleterious	0.9309	Pathogenic	-15.98	Pathogenic	0.77	Disease	1.00	Probably damaging	0.34	Tolerated		
p.Asp84Ser	VUS	-53.15	Deleterious	0.8767	Pathogenic	-11.88	Pathogenic	0.69	Disease	1.00	Probably damaging	0.55	Tolerated		
p.Asp84Ile	VUS	-53.15	Deleterious	0.9329	Pathogenic	-15.86	Pathogenic	0.78	Disease	1.00	Probably damaging	0.17	Tolerated		
p.Asp84Met	VUS	-53.15	Deleterious	0.9818	Pathogenic	-15.99	Pathogenic	0.77	Disease	1.00	Probably damaging	0.1	Tolerated		
p.Asp84His	VUS	-53.15	Deleterious	0.8323	Pathogenic	-12.46	Pathogenic	0.84	Disease	1.00	Probably damaging	0.24	Tolerated	32.00	Deleterious
p.Asp84Gln	VUS	-53.15	Deleterious	0.9182	Pathogenic	-14.08	Pathogenic	0.67	Disease	1.00	Probably damaging	0.36	Tolerated		
p.Asp84Pro	VUS	-53.15	Deleterious	0.9957	Pathogenic	-18.92	Pathogenic	0.77	Disease	0.99	Probably damaging	0.26	Tolerated		
p.Asp84Leu	VUS	-53.15	Deleterious	0.8899	Pathogenic	-15.56	Pathogenic	0.78	Disease	1.00	Probably damaging	0.29	Tolerated		
p.Asp84Glu	VUS	-11.57	Indeterminate	0.7934	Pathogenic	-9.49	Pathogenic	0.69	Disease	0.98	Probably damaging	0.78	Tolerated	27.30	Deleterious
p.Asp84Ala	VUS	-53.15	Deleterious	0.8398	Pathogenic	-11.92	Pathogenic	0.83	Disease	1.00	Probably damaging	0.73	Tolerated	31.00	Deleterious
p.Asp84Gly	VUS	-53.15	Deleterious	0.9245	Pathogenic	-12.25	Pathogenic	0.82	Disease	1.00	Probably damaging	0.5	Tolerated	32.00	Deleterious
p.Asp84Val	VUS	-53.15	Deleterious	0.8435	Pathogenic	-13.70	Pathogenic	0.84	Disease	1.00	Probably damaging	0.26	Tolerated	31.00	Deleterious
p.Asp84Tyr	VUS	-53.15	Deleterious	0.7537	Pathogenic	-13.75	Pathogenic	0.84	Disease	1.00	Probably damaging	0.31	Tolerated	32.00	Deleterious
p.Asp84Cys	VUS	-53.15	Deleterious	0.9561	Pathogenic	-14.29	Pathogenic	0.78	Disease	1.00	Probably damaging	0.09	Tolerated		
p.Asp84Trp	VUS	-53.15	Deleterious	0.9764	Pathogenic	-15.31	Pathogenic	0.78	Disease	1.00	Probably damaging	0.06	Tolerated		
p.Asp84Phe	VUS	-53.15	Deleterious	0.9455	Pathogenic	-14.89	Pathogenic	0.79	Disease	1.00	Probably damaging	0.28	Tolerated		
p.Ala85Asn	VUS	-18.26	Indeterminate	0.9879	Pathogenic	-15.33	Pathogenic	0.79	Disease	1.00	Probably damaging	0	Deleterious		
p.Ala85Lys	VUS	-33.22	Indeterminate	0.9973	Pathogenic	-17.93	Pathogenic	0.83	Disease	1.00	Probably damaging	0	Deleterious		
p.Ala85Thr	VUS	-17.82	Indeterminate	0.6836	Pathogenic	-7.99	Pathogenic	0.83	Disease	1.00	Probably damaging	0	Deleterious	32.00	Deleterious
p.Ala85Arg	VUS	-33.22	Indeterminate	0.978	Pathogenic	-14.78	Pathogenic	0.82	Disease	1.00	Probably damaging	0	Deleterious		
p.Ala85Ser	VUS	-3.75	Neutral	0.4337	Ambiguous	-9.74	Pathogenic	0.70	Disease	0.99	Probably damaging	0	Deleterious	31.00	Deleterious
p.Ala85Ile	VUS	-14.32	Indeterminate	0.9512	Pathogenic	-15.31	Pathogenic	0.83	Disease	1.00	Probably damaging	0	Deleterious		
p.Ala85Met	VUS	-1.40	Neutral	0.9375	Pathogenic	-12.33	Pathogenic	0.78	Disease	1.00	Probably damaging	0	Deleterious		
p.Ala85His	VUS	-27.36	Indeterminate	0.9894	Pathogenic	-14.46	Pathogenic	0.83	Disease	1.00	Probably damaging	0	Deleterious		
p.Ala85Gln	VUS	-17.67	Indeterminate	0.9782	Pathogenic	-10.38	Pathogenic	0.83	Disease	1.00	Probably damaging	0	Deleterious		
p.Ala85Pro	VUS	-13.79	Indeterminate	0.9715	Pathogenic	-13.26	Pathogenic	0.86	Disease	1.00	Probably damaging	0	Deleterious	32.00	Deleterious
p.Ala85Leu	VUS	-2.77	Neutral	0.8177	Pathogenic	-11.90	Pathogenic	0.81	Disease	1.00	Probably damaging	0	Deleterious		
p.Ala85Asp	VUS	-33.22	Indeterminate	0.9893	Pathogenic	-18.56	Pathogenic	0.87	Disease	1.00	Probably damaging	0	Deleterious	32.00	Deleterious
p.Ala85Glu	VUS	-33.20	Indeterminate	0.986	Pathogenic	-16.15	Pathogenic	0.87	Disease	1.00	Probably damaging	0	Deleterious		
p.Ala85Gly	VUS	-0.46	Neutral	0.5102	Ambiguous	-10.76	Pathogenic	0.76	Disease	1.00	Probably damaging	0	Deleterious	31.00	Deleterious
p.Ala85Val	VUS	-9.08	Indeterminate	0.7195	Pathogenic	-9.69	Pathogenic	0.64	Disease	1.00	Probably damaging	0	Deleterious	27.20	Deleterious
p.Ala85Tyr	VUS	-33.10	Indeterminate	0.9938	Pathogenic	-16.68	Pathogenic	0.83	Disease	1.00	Probably damaging	0	Deleterious		
p.Ala85Cys	VUS	-1.78	Neutral	0.8193	Pathogenic	-9.92	Pathogenic	0.76	Disease	1.00	Probably damaging	0	Deleterious		
p.Ala85Trp	VUS	-53.15	Deleterious	0.9971	Pathogenic	-15.39	Pathogenic	0.85	Disease	1.00	Probably damaging	0	Deleterious		
p.Ala85Phe	VUS	-30.24	Indeterminate	0.9849	Pathogenic	-15.98	Pathogenic	0.83	Disease	1.00	Probably damaging	0	Deleterious		
p.Ala86Asn	VUS	-53.15	Deleterious	0.9923	Pathogenic	-21.15	Pathogenic	0.82	Disease	0.98	Probably damaging	0	Deleterious		
p.Ala86Lys	VUS	-53.15	Deleterious	0.9984	Pathogenic	-22.11	Pathogenic	0.86	Disease	1.00	Probably damaging	0	Deleterious		
p.Ala86Thr	VUS	-0.06	Neutral	0.4991	Ambiguous	-8.25	Pathogenic	0.87	Disease	0.96	Probably damaging	0.01	Deleterious	32.00	Deleterious
p.Ala86Arg	VUS	-53.15	Deleterious	0.9881	Pathogenic	-17.11	Pathogenic	0.82	Disease	1.00	Probably damaging	0	Deleterious		
p.Ala86Ser	VUS	-2.71	Neutral	0.281	Benign	-7.75	Pathogenic	0.75	Disease	0.17	Benign	0.12	Tolerated	29.60	Deleterious
p.Ala86Ile	VUS	-5.80	Indeterminate	0.8519	Pathogenic	-14.37	Pathogenic	0.85	Disease	1.00	Probably damaging	0	Deleterious		
p.Ala86Met	VUS	-53.15	Deleterious	0.906	Pathogenic	-15.48	Pathogenic	0.80	Disease	1.00	Probably damaging	0	Deleterious		
p.Ala86His	VUS	-53.15	Deleterious	0.9941	Pathogenic	-21.22	Pathogenic	0.85	Disease	1.00	Probably damaging	0	Deleterious		
p.Ala86Gln	VUS	-53.15	Deleterious	0.9798	Pathogenic	-20.63	Pathogenic	0.85	Disease	1.00	Probably damaging	0	Deleterious		
p.Ala86Pro	VUS	-53.15	Deleterious	0.9789	Pathogenic	-15.35	Pathogenic	0.92	Disease	1.00	Probably damaging	0.01	Deleterious	32.00	Deleterious
p.Ala86Leu	VUS	-53.15	Deleterious	0.7887	Pathogenic	-13.86	Pathogenic	0.84	Disease	1.00	Probably damaging	0	Deleterious		
p.Ala86Asp	VUS	-53.15	Deleterious	0.9955	Pathogenic	-18.42	Pathogenic	0.92	Disease	1.00	Probably damaging	0	Deleterious	32.00	Deleterious
p.Ala86Glu	VUS	-53.15	Deleterious	0.9873	Pathogenic	-18.10	Pathogenic	0.92	Disease	1.00	Probably damaging	0	Deleterious		
p.Ala86Gly	VUS	-0.02	Neutral	0.5397	Ambiguous	-10.00	Pathogenic	0.72	Disease	0.99	Probably damaging	0.03	Deleterious	31.00	Deleterious
p.Ala86Val	VUS	-0.10	Neutral	0.4564	Ambiguous	-8.22	Pathogenic	0.84	Disease	1.00	Probably damaging	0.01	Deleterious	32.00	Deleterious
p.Ala86Tyr	VUS	-53.15	Deleterious	0.9965	Pathogenic	-20.61	Pathogenic	0.85	Disease	1.00	Probably damaging	0	Deleterious		
p.Ala86Cys	VUS	0.00	Neutral	0.716	Pathogenic	-9.01	Pathogenic	0.77	Disease	1.00	Probably damaging	0	Deleterious		
p.Ala86Trp	VUS	-53.15	Deleterious	0.998	Pathogenic	-18.82	Pathogenic	0.86	Disease	1.00	Probably damaging	0	Deleterious		
p.Ala86Phe	VUS	-53.15	Deleterious	0.9859	Pathogenic	-17.77	Pathogenic	0.87	Disease	1.00	Probably damaging	0	Deleterious		
p.Arg87Asn	VUS	-3.51	Neutral	0.972	Pathogenic	-12.31	Pathogenic	0.66	Disease	1.00	Probably damaging	0.12	Tolerated		
p.Arg87Lys	VUS	0.00	Neutral	0.583	Pathogenic	-7.99	Pathogenic	0.58	Disease	0.99	Probably damaging	0.17	Tolerated		
p.Arg87Thr	VUS	-7.09	Indeterminate	0.9022	Pathogenic	-9.79	Pathogenic	0.74	Disease	1.00	Probably damaging	0.88	Tolerated		
p.Arg87Ser	VUS	-53.15	Deleterious	0.9346	Pathogenic	-6.62	Benign	0.73	Disease	1.00	Probably damaging	0.53	Tolerated		
p.Arg87Ile	VUS	-9.73	Indeterminate	0.8709	Pathogenic	-11.77	Pathogenic	0.71	Disease	1.00	Probably damaging	0.04	Deleterious		
p.Arg87Met	VUS	-19.92	Indeterminate	0.9082	Pathogenic	-11.21	Pathogenic	0.68	Disease	1.00	Probably damaging	0.02	Deleterious		
p.Arg87His	VUS	-33.21	Indeterminate	0.6262	Pathogenic	-11.02	Pathogenic	0.64	Disease	1.00	Probably damaging	0.03	Deleterious		
p.Arg87Gln	VUS	-31.79	Indeterminate	0.4766	Ambiguous	-9.61	Pathogenic	0.62	Disease	1.00	Probably damaging	0.08	Tolerated	32.00	Deleterious
p.Arg87Pro	Pathogenic	-53.15	Deleterious	0.9811	Pathogenic	-17.26	Pathogenic	0.81	Disease	1.00	Probably damaging	0.08	Tolerated	32.00	Deleterious
p.Arg87Leu	VUS	-15.67	Indeterminate	0.7068	Pathogenic	-12.08	Pathogenic	0.77	Disease	1.00	Probably damaging	0.05	Deleterious	32.00	Deleterious
p.Arg87Asp	VUS	-53.15	Deleterious	0.9683	Pathogenic	-16.23	Pathogenic	0.69	Disease	1.00	Probably damaging	0.07	Tolerated		
p.Arg87Glu	VUS	-53.15	Deleterious	0.8133	Pathogenic	-13.22	Pathogenic	0.65	Disease	1.00	Probably damaging	0.06	Tolerated		
p.Arg87Ala	VUS	-33.22	Indeterminate	0.8485	Pathogenic	-7.66	Pathogenic	0.60	Disease	1.00	Probably damaging	0.16	Tolerated		
p.Arg87Gly	VUS	-1.07	Neutral	0.8043	Pathogenic	-8.75	Pathogenic	0.76	Disease	1.00	Probably damaging	0.07	Tolerated	27.80	Deleterious
p.Arg87Val	VUS	-6.11	Indeterminate	0.8731	Pathogenic	-11.39	Pathogenic	0.70	Disease	1.00	Probably damaging	0.07	Tolerated		
p.Arg87Tyr	VUS	-53.15	Deleterious	0.8984	Pathogenic	-14.41	Pathogenic	0.66	Disease	0.98	Probably damaging	0.02	Deleterious		
p.Arg87Cys	VUS	-0.11	Neutral	0.7911	Pathogenic	-7.86	Pathogenic	0.76	Disease	1.00	Probably damaging	0.03	Deleterious		
p.Arg87Trp	Likely pathogenic	-53.15	Deleterious	0.6316	Pathogenic	-12.63	Pathogenic	0.58	Disease	0.40	Benign	0.01	Deleterious	31.00	Deleterious
p.Arg87Phe	VUS	-6.79	Indeterminate	0.9377	Pathogenic	-14.69	Pathogenic	0.68	Disease	0.98	Probably damaging	0.02	Deleterious		
p.Glu88Asn	VUS	-8.96	Indeterminate	0.7893	Pathogenic	-10.27	Pathogenic	0.44	Neutral	1.00	Probably damaging	0.63	Tolerated		
p.Glu88Lys	VUS	-26.55	Indeterminate	0.7692	Pathogenic	-11.55	Pathogenic	0.46	Neutral	0.98	Probably damaging	0.84	Tolerated	29.00	Deleterious
p.Glu88Thr	VUS	-13.66	Indeterminate	0.8562	Pathogenic	-7.38	Benign	0.26	Neutral	0.99	Probably damaging	0.93	Tolerated		
p.Glu88Arg	VUS	-23.65	Indeterminate	0.5865	Pathogenic	-11.02	Pathogenic	0.46	Neutral	1.00	Probably damaging	0.49	Tolerated		
p.Glu88Ser	VUS	-8.02	Indeterminate	0.6826	Pathogenic	-8.33	Pathogenic	0.29	Neutral	0.99	Probably damaging	1	Tolerated		
p.Glu88Ile	VUS	-5.97	Indeterminate	0.9304	Pathogenic	-11.79	Pathogenic	0.48	Neutral	0.99	Probably damaging	0.23	Tolerated		
p.Glu88Met	VUS	-4.02	Neutral	0.8959	Pathogenic	-10.44	Pathogenic	0.53	Disease	1.00	Probably damaging	0.12	Tolerated		
p.Glu88His	VUS	-13.18	Indeterminate	0.7291	Pathogenic	-10.46	Pathogenic	0.43	Neutral	1.00	Probably damaging	0.16	Tolerated		

p.Glu88Gln	VUS	-9.17	Indeterminate	0.3834	Ambiguous	-8.59	Pathogenic	0.36	Neutral	0.99	Probably damaging	0.55	Tolerated	26.90	Deleterious
p.Glu88Pro	VUS	-53.15	Deleterious	0.9891	Pathogenic	-14.03	Pathogenic	0.55	Disease	1.00	Probably damaging	0.33	Tolerated		
p.Glu88Leu	VUS	-11.28	Indeterminate	0.8529	Pathogenic	-9.54	Pathogenic	0.49	Neutral	0.94	Possibly damaging	0.33	Tolerated		
p.Glu88Asp	VUS	-8.89	Indeterminate	0.7631	Pathogenic	-8.59	Pathogenic	0.26	Neutral	0.98	Probably damaging	0.68	Tolerated	21.45	Deleterious
p.Glu88Ala	VUS	-4.75	Neutral	0.5098	Ambiguous	-5.30	Benign	0.18	Neutral	0.50	Possibly damaging	0.9	Tolerated	22.70	Deleterious
p.Glu88Gly	VUS	-5.36	Neutral	0.5315	Ambiguous	-8.43	Pathogenic	0.22	Neutral	1.00	Probably damaging	0.48	Tolerated	23.40	Deleterious
p.Glu88Val	VUS	-3.02	Neutral	0.8221	Pathogenic	-9.90	Pathogenic	0.44	Neutral	0.89	Possibly damaging	0.38	Tolerated	26.90	Deleterious
p.Glu88Tyr	VUS	-7.59	Indeterminate	0.8342	Pathogenic	-12.66	Pathogenic	0.50	Neutral	1.00	Probably damaging	0.11	Tolerated		
p.Glu88Cys	VUS	-4.88	Neutral	0.8882	Pathogenic	-10.65	Pathogenic	0.53	Disease	1.00	Probably damaging	0.1	Tolerated		
p.Glu88Trp	VUS	-4.25	Neutral	0.9523	Pathogenic	-14.19	Pathogenic	0.57	Disease	1.00	Probably damaging	0.03	Deleterious		
p.Glu88Phe	VUS	-10.21	Indeterminate	0.9337	Pathogenic	-12.78	Pathogenic	0.50	Neutral	1.00	Probably damaging	0.09	Tolerated		
p.Gly89Asn	VUS	-16.97	Indeterminate	0.9434	Pathogenic	-10.52	Pathogenic	0.81	Disease	1.00	Probably damaging	0	Deleterious		
p.Gly89Lys	VUS	-53.15	Deleterious	0.9852	Pathogenic	-17.07	Pathogenic	0.84	Disease	1.00	Probably damaging	0	Deleterious		
p.Gly89Thr	VUS	-53.15	Deleterious	0.9692	Pathogenic	-18.41	Pathogenic	0.82	Disease	1.00	Probably damaging	0	Deleterious		
p.Gly89Arg	VUS	-53.15	Deleterious	0.9629	Pathogenic	-15.21	Pathogenic	0.87	Disease	1.00	Probably damaging	0	Deleterious	32.00	Deleterious
p.Gly89Ser	VUS	-53.15	Deleterious	0.8246	Pathogenic	-13.01	Pathogenic	0.84	Disease	1.00	Probably damaging	0	Deleterious	32.00	Deleterious
p.Gly89Ile	VUS	-53.15	Deleterious	0.9909	Pathogenic	-21.63	Pathogenic	0.86	Disease	1.00	Probably damaging	0	Deleterious		
p.Gly89Met	VUS	-53.15	Deleterious	0.9948	Pathogenic	-19.70	Pathogenic	0.87	Disease	1.00	Probably damaging	0	Deleterious		
p.Gly89His	VUS	-53.15	Deleterious	0.9876	Pathogenic	-16.30	Pathogenic	0.84	Disease	1.00	Probably damaging	0	Deleterious		
p.Gly89Gln	VUS	-53.15	Deleterious	0.9814	Pathogenic	-17.42	Pathogenic	0.82	Disease	1.00	Probably damaging	0	Deleterious		
p.Gly89Pro	VUS	-53.15	Deleterious	0.998	Pathogenic	-17.63	Pathogenic	0.83	Disease	1.00	Probably damaging	0	Deleterious		
p.Gly89Leu	VUS	-53.15	Deleterious	0.991	Pathogenic	-18.75	Pathogenic	0.85	Disease	1.00	Probably damaging	0	Deleterious		
p.Gly89Asp	VUS	-53.15	Deleterious	0.9296	Pathogenic	-10.89	Pathogenic	0.86	Disease	1.00	Probably damaging	0	Deleterious	32.00	Deleterious
p.Gly89Glu	VUS	-53.15	Deleterious	0.9738	Pathogenic	-15.05	Pathogenic	0.85	Disease	1.00	Probably damaging	0	Deleterious		
p.Gly89Ala	VUS	-23.61	Indeterminate	0.8544	Pathogenic	-12.94	Pathogenic	0.87	Disease	1.00	Probably damaging	0	Deleterious	29.30	Deleterious
p.Gly89Val	VUS	-53.15	Deleterious	0.981	Pathogenic	-17.26	Pathogenic	0.91	Disease	1.00	Probably damaging	0	Deleterious	31.00	Deleterious
p.Gly89Tyr	VUS	-53.15	Deleterious	0.9872	Pathogenic	-18.70	Pathogenic	0.85	Disease	1.00	Probably damaging	0	Deleterious		
p.Gly89Cys	VUS	-53.15	Deleterious	0.9645	Pathogenic	-14.56	Pathogenic	0.91	Disease	1.00	Probably damaging	0	Deleterious	32.00	Deleterious
p.Gly89Trp	VUS	-53.15	Deleterious	0.9907	Pathogenic	-17.79	Pathogenic	0.89	Disease	1.00	Probably damaging	0	Deleterious		
p.Gly89Phe	VUS	-53.15	Deleterious	0.9964	Pathogenic	-19.66	Pathogenic	0.87	Disease	1.00	Probably damaging	0	Deleterious		
p.Phe90Asn	VUS	-0.08	Neutral	0.8549	Pathogenic	-9.53	Pathogenic	0.83	Disease	1.00	Probably damaging	0.15	Tolerated		
p.Phe90Lys	VUS	-2.63	Neutral	0.9421	Pathogenic	-5.70	Benign	0.79	Disease	1.00	Probably damaging	0.13	Tolerated		
p.Phe90Thr	VUS	-3.69	Neutral	0.9749	Pathogenic	-11.43	Pathogenic	0.83	Disease	1.00	Probably damaging	0.19	Tolerated		
p.Phe90Arg	VUS	-0.31	Neutral	0.8195	Pathogenic	-6.11	Benign	0.83	Disease	1.00	Probably damaging	0.14	Tolerated		
p.Phe90Ser	VUS	-0.47	Neutral	0.8915	Pathogenic	-8.46	Pathogenic	0.87	Disease	1.00	Probably damaging	0.33	Tolerated	32.00	Deleterious
p.Phe90Ile	VUS	-0.42	Neutral	0.9075	Pathogenic	-9.55	Pathogenic	0.77	Disease	1.00	Probably damaging	0.15	Tolerated	32.00	Deleterious
p.Phe90Met	VUS	-0.09	Neutral	0.9148	Pathogenic	-6.68	Benign	0.76	Disease	1.00	Probably damaging	0.09	Tolerated		
p.Phe90His	VUS	-0.17	Neutral	0.6115	Pathogenic	-6.31	Benign	0.85	Disease	1.00	Probably damaging	0.36	Tolerated		
p.Phe90Gln	VUS	-0.06	Neutral	0.8916	Pathogenic	-7.91	Pathogenic	0.81	Disease	1.00	Probably damaging	0.12	Tolerated		
p.Phe90Pro	VUS	-8.66	Indeterminate	0.9992	Pathogenic	-15.49	Pathogenic	0.83	Disease	1.00	Probably damaging	0.1	Tolerated		
p.Phe90Leu	VUS	-0.06	Neutral	0.9825	Pathogenic	-4.19	Benign	0.80	Disease	1.00	Probably damaging	0.25	Tolerated	29.17	Deleterious
p.Phe90Asp	VUS	-17.51	Indeterminate	0.9453	Pathogenic	-13.21	Pathogenic	0.77	Disease	1.00	Probably damaging	0.11	Tolerated		
p.Phe90Glu	VUS	-1.91	Neutral	0.963	Pathogenic	-12.39	Pathogenic	0.82	Disease	1.00	Probably damaging	0.12	Tolerated		
p.Phe90Ala	VUS	-0.40	Neutral	0.9492	Pathogenic	-8.68	Pathogenic	0.81	Disease	1.00	Probably damaging	0.43	Tolerated		
p.Phe90Gly	VUS	-0.76	Neutral	0.9762	Pathogenic	-11.21	Pathogenic	0.82	Disease	1.00	Probably damaging	0.19	Tolerated		
p.Phe90Val	VUS	-1.40	Neutral	0.8919	Pathogenic	-9.16	Pathogenic	0.85	Disease	1.00	Probably damaging	0.2	Tolerated	31.00	Deleterious
p.Phe90Tyr	VUS	-0.05	Neutral	0.1801	Benign	-7.16	Benign	0.72	Disease	0.99	Probably damaging	0.36	Tolerated	32.00	Deleterious
p.Phe90Cys	VUS	-0.46	Neutral	0.7307	Pathogenic	-7.78	Pathogenic	0.88	Disease	1.00	Probably damaging	0.07	Tolerated	32.00	Deleterious
p.Phe90Trp	VUS	-0.34	Neutral	0.6305	Pathogenic	-9.88	Pathogenic	0.71	Disease	1.00	Probably damaging	0.07	Tolerated		
p.Leu91Asn	VUS	-0.02	Neutral	0.8419	Pathogenic	-17.48	Pathogenic	0.70	Disease	1.00	Probably damaging	0	Deleterious		
p.Leu91Lys	VUS	-7.34	Indeterminate	0.5184	Ambiguous	-13.81	Pathogenic	0.69	Disease	0.99	Probably damaging	0	Deleterious		
p.Leu91Thr	VUS	-8.95	Indeterminate	0.3834	Ambiguous	-11.50	Pathogenic	0.66	Disease	1.00	Probably damaging	0.01	Deleterious		
p.Leu91Arg	VUS	-1.49	Neutral	0.2806	Benign	-11.67	Pathogenic	0.83	Disease	1.00	Probably damaging	0	Deleterious	32.00	Deleterious
p.Leu91Ser	VUS	-18.24	Indeterminate	0.6895	Pathogenic	-13.58	Pathogenic	0.72	Disease	1.00	Probably damaging	0	Deleterious		
p.Leu91Ile	VUS	-19.53	Indeterminate	0.2114	Benign	-10.91	Pathogenic	0.47	Neutral	0.57	Possibly damaging	0.24	Tolerated		
p.Leu91Met	VUS	-33.05	Indeterminate	0.3357	Benign	-8.49	Pathogenic	0.46	Neutral	1.00	Probably damaging	0.02	Deleterious	23.90	Deleterious
p.Leu91His	VUS	-16.61	Indeterminate	0.5598	Ambiguous	-13.10	Pathogenic	0.72	Disease	1.00	Probably damaging	0	Deleterious		
p.Leu91Gln	VUS	-0.16	Neutral	0.4213	Ambiguous	-11.72	Pathogenic	0.52	Disease	1.00	Probably damaging	0	Deleterious	31.00	Deleterious
p.Leu91Pro	VUS	-7.49	Indeterminate	0.4079	Ambiguous	-11.70	Pathogenic	0.84	Disease	1.00	Probably damaging	0	Deleterious	32.00	Deleterious
p.Leu91Asp	VUS	-6.28	Indeterminate	0.9074	Pathogenic	-15.92	Pathogenic	0.69	Disease	1.00	Probably damaging	0	Deleterious		
p.Leu91Glu	VUS	-4.76	Neutral	0.6245	Pathogenic	-13.29	Pathogenic	0.68	Disease	1.00	Probably damaging	0	Deleterious		
p.Leu91Ala	VUS	-1.03	Neutral	0.4238	Ambiguous	-11.83	Pathogenic	0.61	Disease	1.00	Probably damaging	0.01	Deleterious		
p.Leu91Gly	VUS	-4.74	Neutral	0.6454	Pathogenic	-14.23	Pathogenic	0.68	Disease	1.00	Probably damaging	0	Deleterious		
p.Leu91Val	VUS	-2.65	Neutral	0.1689	Benign	-8.72	Pathogenic	0.39	Neutral	0.89	Possibly damaging	0.18	Tolerated	23.40	Deleterious
p.Leu91Tyr	VUS	-53.15	Deleterious	0.7318	Pathogenic	-14.27	Pathogenic	0.60	Disease	1.00	Probably damaging	0	Deleterious		
p.Leu91Cys	VUS	-0.69	Neutral	0.749	Pathogenic	-13.62	Pathogenic	0.61	Disease	1.00	Probably damaging	0	Deleterious		
p.Leu91Trp	VUS	-8.02	Indeterminate	0.5174	Ambiguous	-13.64	Pathogenic	0.65	Disease	1.00	Probably damaging	0	Deleterious		
p.Leu91Phe	VUS	-21.09	Indeterminate	0.4145	Ambiguous	-10.97	Pathogenic	0.61	Disease	1.00	Probably damaging	0.02	Deleterious		
p.Asp92Asn	VUS	-3.34	Neutral	0.4396	Ambiguous	-10.26	Pathogenic	0.60	Disease	0.99	Probably damaging	0.35	Tolerated	32.00	Deleterious
p.Asp92Lys	VUS	-7.05	Indeterminate	0.7779	Pathogenic	-13.34	Pathogenic	0.66	Disease	1.00	Probably damaging	0.05	Deleterious		
p.Asp92Thr	VUS	-1.00	Neutral	0.6917	Pathogenic	-11.07	Pathogenic	0.65	Disease	1.00	Probably damaging	0.04	Deleterious		
p.Asp92Arg	VUS	-1.01	Neutral	0.643	Pathogenic	-11.51	Pathogenic	0.64	Disease	1.00	Probably damaging	0.02	Deleterious		
p.Asp92Ser	VUS	-3.99	Neutral	0.4136	Ambiguous	-8.28	Pathogenic	0.58	Disease	1.00	Probably damaging	0.08	Tolerated		
p.Asp92Ile	VUS	-2.32	Neutral	0.9298	Pathogenic	-16.37	Pathogenic	0.64	Disease	1.00	Probably damaging	0	Deleterious		
p.Asp92Met	VUS	-0.61	Neutral	0.9641	Pathogenic	-14.36	Pathogenic	0.68	Disease	1.00	Probably damaging	0	Deleterious		
p.Asp92His	VUS	-4.28	Neutral	0.5663	Pathogenic	-11.15	Pathogenic	0.66	Disease	1.00	Probably damaging	0.03	Deleterious	32.00	Deleterious
p.Asp92Gln	VUS	-0.89	Neutral	0.5609	Ambiguous	-10.85	Pathogenic	0.59	Disease	1.00	Probably damaging	0.03	Deleterious		
p.Asp92Pro	VUS	-1.59	Neutral	0.4936	Ambiguous	-10.20	Pathogenic	0.66	Disease	0.99	Probably damaging	0.03	Deleterious		
p.Asp92Leu	VUS	-0.53	Neutral	0.8186	Pathogenic	-12.76	Pathogenic	0.68	Disease	1.00	Probably damaging	0.01	Deleterious		
p.Asp92Glu	VUS	-3.93	Neutral	0.3017	Benign	-6.36	Benign	0.19	Neutral	0.93	Possibly damaging	0.07	Tolerated	22.80	Deleterious
p.Asp92Ala	VUS	-0.99	Neutral	0.3843	Ambiguous	-7.59	Pathogenic	0.39	Neutral	1.00	Probably damaging	0.16	Tolerated	28.20	Deleterious
p.Asp92Gly	VUS	-5.17	Neutral	0.3826	Ambiguous	-6.77	Benign	0.40	Neutral	1.00	Probably damaging	0.1	Tolerated	31.00	Deleterious
p.Asp92Val	VUS	-0.82	Neutral	0.7553	Pathogenic	-12.77	Pathogenic	0.72	Disease	1.00	Probably damaging	0.01	Deleterious	30.00	Deleterious
p.Asp92Tyr	VUS	-4.06	Neutral	0.7255	Pathogenic	-15.33	Pathogenic	0.78	Disease	1.00	Probably damaging	0.02	Deleterious	32.00	Deleterious
p.Asp92Cys	VUS	-0.57	Neutral	0.9254	Pathogenic	-13.02	Pathogenic	0.63	Disease	1.00	Probably damaging	0.01	Deleterious		
p.Asp92Trp	VUS	-3.72	Neutral	0.9587	Pathogenic	-13.87	Pathogenic	0.67	Disease	1.00	Probably damaging	0	Deleterious		
p.Asp92Phe	VUS	-4.82	Neutral	0.9489	Pathogenic	-16.79	Pathogenic	0.68	Disease	1.00	Probably damaging	0.01	Deleterious		
p.Thr93Asn	VUS	-16.51	Indeterminate	0.697	Pathogenic	-9.47	Pathogenic	0.81	Disease	1.00	Probably damaging	0.07	Tolerated		
p.Thr93Lys	VUS	-53.15	Deleterious	0.9499	Pathogenic	-12.30	Pathogenic	0.86	Disease	1.00	Probably damaging	0.07	Tolerated	29.50	Deleterious
p.Thr93Arg	VUS	-33.14	Indeterminate	0.914	Pathogenic	-15.35	Pathogenic	0.87	Disease	1.00	Probably damaging	0.04	Deleterious	29.00	Deleterious
p.Thr93Ser	VUS	-8.91	Indeterminate	0.4599	Ambiguous	-8.45	Pathogenic	0.68	Disease	1.00	Probably damaging	0.29	Tolerated	26.90	Deleterious
p.Thr93Ile	VUS	-0.81	Neutral	0.6269	Pathogenic	-9.66	Pathogenic	0.86	Disease	1.00	Probably damaging	0.23	Tolerated		
p.Thr93Met	VUS	-13.29	Indeterminate	0.4101	Ambiguous	-9.46	Pathogenic	0.80	Disease	1.00	Probably damaging	0.08	Tolerated	29.70	Deleterious
p.Thr93His	VUS	-25.89	Indeterminate	0.8809	Pathogenic	-11.97	Pathogenic	0.78	Disease	1.00	Probably damaging	0.02	Deleterious		
p.Thr93Gln	VUS	-20.83	Indeterminate	0.8851	Pathogenic	-11.62	Pathogenic	0.75	Disease	1.00	Probably damaging	0.05	Deleterious		
p.Thr93Pro	VUS	-53.15	Deleterious	0.6064	Pathogenic	-14.33	Pathogenic	0.72	Disease	1.00	Probably damaging	0.08	Tolerated	28.20	Deleterious
p.Thr93Leu	VUS	-9.12	Indeterminate	0.4735	Ambiguous	-11.97	Pathogenic	0.78	Disease	1.00	Probably damaging	0.39	Tolerated		
p.Thr93Asp	VUS	-53.15	Deleterious	0.933	Pathogenic	-15.10	Pathogenic	0.79	Disease	1.00	Probably damaging	0.05	Deleterious		
p.Thr93Glu	VUS	-53.15	Deleterious	0.9352	Pathogenic	-14.21	Pathogenic	0.79	Disease	1.00	Probably damaging	0.05	Deleterious		
p.Thr93Ala	VUS	-23.13	Indeterminate	0.2995	Benign	-8.21	Pathogenic	0.68	Disease	1.00	Probably damaging	0.28	Tolerated	27.10	Deleterious
p.Thr93Gly	VUS	-23.79	Indeterminate	0.6733	Pathogenic	-10.98	Pathogenic	0.73	Disease	1.00	Probably damaging	0.06	Tolerated		
p.Thr93Val	VUS	-5.99	Indeterminate	0.3711	Ambiguous	-7.09	Benign	0.51	Disease	1.00	Probably damaging	0.4	Tolerated		
p.Thr93Tyr	VUS	-33.22	Indeterminate	0.9494	Pathogenic	-16.27	Pathogenic	0.81	Disease	1.00	Probably damaging	0.03	Deleterious		
p.Thr93Cys	VUS	-9.44	Indeterminate	0.6612	Pathogenic	-10.25	Pathogenic	0.77	Disease	1.00	Probably damaging	0.14	Tolerated		
p.Thr93Trp	VUS	-24.67	Indeterminate	0.9837	Pathogenic	-17.35	Pathogenic	0.79	Disease	1.00	Probably damaging	0.01	Deleterious		
p.Thr93Phe	VUS	-15.25	Indeterminate	0.9286	Pathogenic	-14.73	Pathogenic	0.81	Disease	1.00	Probably damaging	0.06	Tolerated		
p.Leu94Asn	VUS	-32.69	Indeterminate	0.986	Pathogenic	-19.97	Pathogenic	0.76	Disease	1.00	Probably damaging	0	Deleterious		
p.Leu94Lys	VUS	-53.15	Deleterious	0.9865	Pathogenic	-23.40	Pathogenic	0.75	Disease	0.99	Probably damaging	0.01	Deleterious		
p.Leu94Thr	VUS	-32.22	Indeterminate	0.6064	Pathogenic	-12.32	Pathogenic	0.70	Disease	1.00	Probably damaging	0.01	Deleterious		
p.Leu94Arg	VUS	-53.15	Deleterious	0.9491	Pathogenic	-19.07	Pathogenic	0.71	Disease	1.00	Probably damaging	0	Deleterious	32.00	Deleterious
p.Leu94Ser	VUS	-0.47	Neutral	0.9449	Pathogenic	-15.68	Pathogenic	0.75	Disease	1.00	Probably damaging	0	Deleterious		

p.Leu94Ile	VUS	-0.01	Neutral	0.1938	Benign	-6.65	Benign	0.31	Neutral	1.00	Probably damaging	0.09	Tolerated		
p.Leu94Met	VUS	-0.01	Neutral	0.2911	Benign	-9.48	Pathogenic	0.47	Neutral	1.00	Probably damaging	0.03	Deleterious	24.10	Deleterious
p.Leu94His	VUS	-53.15	Deleterious	0.9832	Pathogenic	-17.38	Pathogenic	0.80	Disease	1.00	Probably damaging	0	Deleterious		
p.Leu94Gln	Likely pathogenic	-53.15	Deleterious	0.9434	Pathogenic	-17.94	Pathogenic	0.86	Disease	1.00	Probably damaging	0.01	Deleterious	31.00	Deleterious
p.Leu94Pro	Likely pathogenic	-53.15	Deleterious	0.9693	Pathogenic	-14.99	Pathogenic	0.92	Disease	1.00	Probably damaging	0.01	Deleterious	31.00	Deleterious
p.Leu94Asp	VUS	-53.15	Deleterious	0.9968	Pathogenic	-19.02	Pathogenic	0.73	Disease	1.00	Probably damaging	0	Deleterious		
p.Leu94Glu	VUS	-53.15	Deleterious	0.9784	Pathogenic	-18.62	Pathogenic	0.74	Disease	1.00	Probably damaging	0	Deleterious		
p.Leu94Ala	VUS	-33.22	Indeterminate	0.5738	Pathogenic	-10.94	Pathogenic	0.64	Disease	1.00	Probably damaging	0.02	Deleterious		
p.Leu94Gly	VUS	-53.15	Deleterious	0.9556	Pathogenic	-16.15	Pathogenic	0.70	Disease	1.00	Probably damaging	0	Deleterious		
p.Leu94Val	VUS	-3.56	Neutral	0.096	Benign	-5.53	Benign	0.22	Neutral	1.00	Probably damaging	0.45	Tolerated	18.64	Deleterious
p.Leu94Tyr	VUS	-53.15	Deleterious	0.9829	Pathogenic	-15.31	Pathogenic	0.68	Disease	1.00	Probably damaging	0.01	Deleterious		
p.Leu94Cys	VUS	-8.77	Indeterminate	0.8797	Pathogenic	-12.71	Pathogenic	0.63	Disease	1.00	Probably damaging	0	Deleterious		
p.Leu94Trp	VUS	-53.15	Deleterious	0.9769	Pathogenic	-16.43	Pathogenic	0.72	Disease	1.00	Probably damaging	0	Deleterious		
p.Leu94Phe	VUS	-9.94	Indeterminate	0.8065	Pathogenic	-10.88	Pathogenic	0.52	Disease	1.00	Probably damaging	0.03	Deleterious		
p.Val95Asn	VUS	-4.64	Neutral	0.5539	Ambiguous	-12.79	Pathogenic	0.37	Neutral	0.90	Possibly damaging	0.38	Tolerated		
p.Val95Lys	VUS	-0.83	Neutral	0.3308	Benign	-8.07	Pathogenic	0.19	Neutral	0.33	Benign	1	Tolerated		
p.Val95Thr	VUS	-4.38	Neutral	0.2304	Benign	-7.25	Benign	0.28	Neutral	0.67	Possibly damaging	0.45	Tolerated		
p.Val95Arg	VUS	-3.32	Neutral	0.1836	Benign	-6.75	Benign	0.17	Neutral	0.52	Possibly damaging	0.65	Tolerated		
p.Val95Ser	VUS	-4.99	Neutral	0.3177	Benign	-9.04	Pathogenic	0.33	Neutral	0.83	Possibly damaging	0.52	Tolerated		
p.Val95Ile	VUS	-2.11	Neutral	0.1425	Benign	-6.02	Benign	0.20	Neutral	0.01	Benign	0.18	Tolerated		
p.Val95Met	VUS	-5.88	Indeterminate	0.2892	Benign	-7.98	Benign	0.12	Neutral	0.02	Benign	0.1	Tolerated	15.05	Deleterious
p.Val95His	VUS	-0.48	Neutral	0.5271	Ambiguous	-10.30	Pathogenic	0.23	Neutral	0.95	Possibly damaging	0.15	Tolerated		
p.Val95Gln	VUS	-11.31	Indeterminate	0.2673	Benign	-7.77	Pathogenic	0.20	Neutral	0.42	Benign	0.56	Tolerated		
p.Val95Pro	VUS	-53.15	Deleterious	0.7501	Pathogenic	-13.48	Pathogenic	0.38	Neutral	0.82	Possibly damaging	0.25	Tolerated		
p.Val95Leu	VUS	-1.93	Neutral	0.1973	Benign	-7.70	Pathogenic	0.10	Neutral	0.00	Benign	0.28	Tolerated	13.66	Neutral
p.Val95Asp	VUS	-2.24	Neutral	0.5199	Ambiguous	-13.25	Pathogenic	0.37	Neutral	0.99	Probably damaging	0.53	Tolerated		
p.Val95Glu	VUS	-3.95	Neutral	0.2418	Benign	-10.87	Pathogenic	0.41	Neutral	0.91	Possibly damaging	0.97	Tolerated	12.13	Neutral
p.Val95Ala	VUS	-0.53	Neutral	0.1371	Benign	-5.31	Benign	0.09	Neutral	0.02	Benign	0.6	Tolerated	10.96	Neutral
p.Val95Gly	VUS	-2.19	Neutral	0.2822	Benign	-9.77	Pathogenic	0.27	Neutral	0.93	Possibly damaging	0.29	Tolerated	14.08	Neutral
p.Val95Tyr	VUS	-5.69	Neutral	0.6414	Pathogenic	-12.32	Pathogenic	0.37	Neutral	0.68	Possibly damaging	0.08	Tolerated		
p.Val95Cys	VUS	-1.01	Neutral	0.7598	Pathogenic	-7.33	Benign	0.37	Neutral	0.96	Probably damaging	0.05	Deleterious		
p.Val95Trp	VUS	-8.18	Indeterminate	0.8085	Pathogenic	-11.12	Pathogenic	0.40	Neutral	0.99	Probably damaging	0.03	Deleterious		
p.Val95Phe	VUS	-6.73	Indeterminate	0.2799	Benign	-12.40	Pathogenic	0.35	Neutral	0.52	Possibly damaging	0.07	Tolerated		
p.Val96Asn	VUS	-5.06	Neutral	0.7289	Pathogenic	-13.54	Pathogenic	0.51	Disease	0.77	Possibly damaging	0.02	Deleterious		
p.Val96Lys	VUS	-0.44	Neutral	0.7191	Pathogenic	-14.08	Pathogenic	0.51	Disease	0.05	Benign	0.04	Deleterious		
p.Val96Thr	VUS	-0.49	Neutral	0.2976	Benign	-6.39	Benign	0.23	Neutral	0.56	Possibly damaging	0.23	Tolerated		
p.Val96Arg	VUS	-0.44	Neutral	0.4778	Ambiguous	-10.88	Pathogenic	0.50	Disease	0.19	Benign	0.12	Tolerated		
p.Val96Ser	VUS	-0.05	Neutral	0.4376	Ambiguous	-8.35	Pathogenic	0.41	Neutral	0.61	Possibly damaging	0.04	Deleterious		
p.Val96Ile	VUS	-5.81	Indeterminate	0.1363	Benign	-7.68	Pathogenic	0.10	Neutral	0.00	Benign	0.48	Tolerated		
p.Val96Met	VUS	-0.70	Neutral	0.3646	Ambiguous	-8.53	Pathogenic	0.11	Neutral	0.01	Benign	0.06	Tolerated	20.90	Deleterious
p.Val96His	VUS	-4.73	Neutral	0.7703	Pathogenic	-12.65	Pathogenic	0.46	Neutral	0.54	Possibly damaging	0.01	Deleterious		
p.Val96Gln	VUS	-2.82	Neutral	0.538	Ambiguous	-11.35	Pathogenic	0.45	Neutral	0.07	Benign	0.03	Deleterious		
p.Val96Pro	VUS	-1.23	Neutral	0.7634	Pathogenic	-10.28	Pathogenic	0.48	Neutral	0.66	Possibly damaging	0.05	Deleterious		
p.Val96Leu	VUS	-12.83	Indeterminate	0.25	Benign	-5.17	Benign	0.07	Neutral	0.00	Benign	0.36	Tolerated	15.88	Deleterious
p.Val96Asp	VUS	-10.72	Indeterminate	0.7853	Pathogenic	-13.82	Pathogenic	0.59	Disease	0.90	Possibly damaging	0.02	Deleterious		
p.Val96Glu	VUS	-1.00	Neutral	0.5405	Ambiguous	-12.30	Pathogenic	0.29	Neutral	0.01	Benign	0.04	Deleterious	14.61	Neutral
p.Val96Ala	VUS	-53.15	Deleterious	0.2136	Benign	-4.20	Benign	0.08	Neutral	0.00	Benign	0.32	Tolerated	6.24	Neutral
p.Val96Gly	VUS	-5.35	Neutral	0.4053	Ambiguous	-10.47	Pathogenic	0.37	Neutral	0.80	Possibly damaging	0.01	Deleterious	18.21	Deleterious
p.Val96Tyr	VUS	-7.43	Indeterminate	0.7911	Pathogenic	-13.57	Pathogenic	0.40	Neutral	0.83	Possibly damaging	0.02	Deleterious		
p.Val96Cys	VUS	-8.48	Indeterminate	0.766	Pathogenic	-6.53	Benign	0.38	Neutral	0.83	Possibly damaging	0.05	Deleterious		
p.Val96Trp	VUS	-17.98	Indeterminate	0.8816	Pathogenic	-11.55	Pathogenic	0.40	Neutral	0.98	Probably damaging	0.01	Deleterious		
p.Val96Phe	VUS	-3.30	Neutral	0.3632	Ambiguous	-10.93	Pathogenic	0.38	Neutral	0.45	Benign	0.04	Deleterious		
p.Leu97Asn	VUS	-53.15	Deleterious	0.9929	Pathogenic	-18.02	Pathogenic	0.90	Disease	1.00	Probably damaging	0	Deleterious		
p.Leu97Lys	VUS	-53.15	Deleterious	0.9869	Pathogenic	-20.26	Pathogenic	0.87	Disease	0.99	Probably damaging	0	Deleterious		
p.Leu97Thr	VUS	-53.15	Deleterious	0.974	Pathogenic	-14.28	Pathogenic	0.89	Disease	1.00	Probably damaging	0	Deleterious		
p.Leu97Arg	VUS	-53.15	Deleterious	0.9325	Pathogenic	-14.97	Pathogenic	0.98	Disease	1.00	Probably damaging	0	Deleterious	29.90	Deleterious
p.Leu97Ser	VUS	-53.15	Deleterious	0.9912	Pathogenic	-12.95	Pathogenic	0.97	Disease	1.00	Probably damaging	0	Deleterious		
p.Leu97Ile	VUS	-0.26	Neutral	0.5969	Pathogenic	-12.02	Pathogenic	0.76	Disease	1.00	Probably damaging	0	Deleterious		
p.Leu97Met	VUS	-0.03	Neutral	0.6247	Pathogenic	-8.97	Pathogenic	0.82	Disease	1.00	Probably damaging	0	Deleterious	28.20	Deleterious
p.Leu97His	VUS	-53.15	Deleterious	0.9765	Pathogenic	-13.98	Pathogenic	0.96	Disease	1.00	Probably damaging	0	Deleterious		
p.Leu97Gln	VUS	-53.15	Deleterious	0.9516	Pathogenic	-13.04	Pathogenic	0.98	Disease	1.00	Probably damaging	0	Deleterious	29.20	Deleterious
p.Leu97Pro	VUS	-53.15	Deleterious	0.9583	Pathogenic	-11.16	Pathogenic	0.99	Disease	1.00	Probably damaging	0	Deleterious	29.70	Deleterious
p.Leu97Asp	VUS	-53.15	Deleterious	0.9983	Pathogenic	-18.54	Pathogenic	0.88	Disease	1.00	Probably damaging	0	Deleterious		
p.Leu97Glu	VUS	-53.15	Deleterious	0.9855	Pathogenic	-17.43	Pathogenic	0.90	Disease	1.00	Probably damaging	0	Deleterious		
p.Leu97Ala	VUS	-53.15	Deleterious	0.9489	Pathogenic	-12.12	Pathogenic	0.87	Disease	1.00	Probably damaging	0	Deleterious		
p.Leu97Gly	VUS	-53.15	Deleterious	0.98	Pathogenic	-16.62	Pathogenic	0.90	Disease	1.00	Probably damaging	0	Deleterious		
p.Leu97Val	VUS	-11.89	Indeterminate	0.6824	Pathogenic	-12.00	Pathogenic	0.86	Disease	1.00	Probably damaging	0	Deleterious	27.70	Deleterious
p.Leu97Tyr	VUS	-0.83	Neutral	0.9699	Pathogenic	-14.71	Pathogenic	0.86	Disease	1.00	Probably damaging	0	Deleterious		
p.Leu97Cys	VUS	-0.82	Neutral	0.9414	Pathogenic	-13.95	Pathogenic	0.82	Disease	1.00	Probably damaging	0	Deleterious		
p.Leu97Trp	VUS	-53.15	Deleterious	0.9348	Pathogenic	-13.49	Pathogenic	0.89	Disease	1.00	Probably damaging	0	Deleterious		
p.Leu97Phe	VUS	-0.45	Neutral	0.7408	Pathogenic	-11.26	Pathogenic	0.86	Disease	1.00	Probably damaging	0	Deleterious		
p.His98Asn	VUS	-0.67	Neutral	0.6006	Pathogenic	-11.17	Pathogenic	0.57	Disease	1.00	Probably damaging	0.05	Deleterious	24.30	Deleterious
p.His98Lys	VUS	-1.47	Neutral	0.8132	Pathogenic	-11.46	Pathogenic	0.54	Disease	0.65	Possibly damaging	0.08	Tolerated		
p.His98Thr	VUS	-2.77	Neutral	0.8741	Pathogenic	-13.12	Pathogenic	0.58	Disease	1.00	Probably damaging	0.16	Tolerated		
p.His98Arg	VUS	-0.45	Neutral	0.4032	Ambiguous	-6.80	Benign	0.32	Neutral	0.02	Benign	0.07	Tolerated	21.30	Deleterious
p.His98Ser	VUS	-3.55	Neutral	0.6155	Pathogenic	-10.39	Pathogenic	0.55	Disease	1.00	Probably damaging	0.07	Tolerated		
p.His98Ile	VUS	-0.50	Neutral	0.8886	Pathogenic	-13.55	Pathogenic	0.61	Disease	0.98	Probably damaging	0.45	Tolerated		
p.His98Met	VUS	-0.87	Neutral	0.9337	Pathogenic	-13.32	Pathogenic	0.56	Disease	1.00	Probably damaging	0.23	Tolerated		
p.His98Gln	VUS	-4.71	Neutral	0.5458	Ambiguous	-7.97	Pathogenic	0.54	Disease	0.80	Possibly damaging	0.07	Tolerated	21.20	Deleterious
p.His98Pro	VUS	-53.15	Deleterious	0.9124	Pathogenic	-16.70	Pathogenic	0.60	Disease	1.00	Probably damaging	0.07	Tolerated	23.90	Deleterious
p.His98Leu	VUS	-2.42	Neutral	0.4531	Ambiguous	-10.14	Pathogenic	0.33	Neutral	0.87	Possibly damaging	1	Tolerated	21.10	Deleterious
p.His98Asp	VUS	-3.97	Neutral	0.6312	Pathogenic	-11.83	Pathogenic	0.62	Disease	1.00	Probably damaging	0.03	Deleterious	24.60	Deleterious
p.His98Glu	VUS	-0.45	Neutral	0.714	Pathogenic	-12.40	Pathogenic	0.55	Disease	0.99	Probably damaging	0.06	Tolerated		
p.His98Ala	VUS	-0.20	Neutral	0.6583	Pathogenic	-10.81	Pathogenic	0.56	Disease	1.00	Probably damaging	0.23	Tolerated		
p.His98Gly	VUS	-2.35	Neutral	0.7349	Pathogenic	-11.64	Pathogenic	0.56	Disease	1.00	Probably damaging	0.04	Deleterious		
p.His98Val	VUS	-0.16	Neutral	0.7307	Pathogenic	-10.20	Pathogenic	0.29	Neutral	1.00	Probably damaging	0.49	Tolerated		
p.His98Tyr	VUS	-0.56	Neutral	0.2623	Benign	-8.49	Pathogenic	0.54	Disease	0.98	Probably damaging	0.08	Tolerated	23.20	Deleterious
p.His98Cys	VUS	-0.94	Neutral	0.6208	Pathogenic	-10.68	Pathogenic	0.54	Disease	1.00	Probably damaging	0.05	Deleterious		
p.His98Trp	VUS	-0.29	Neutral	0.6278	Pathogenic	-10.62	Pathogenic	0.59	Disease	1.00	Probably damaging	0.03	Deleterious		
p.His98Phe	VUS	-0.37	Neutral	0.7347	Pathogenic	-10.97	Pathogenic	0.38	Neutral	1.00	Probably damaging	0.21	Tolerated		
p.Arg99Asn	VUS	-8.76	Indeterminate	0.5313	Ambiguous	-11.41	Pathogenic	0.50	Neutral	0.96	Probably damaging	0.48	Tolerated		
p.Arg99Lys	VUS	-6.84	Indeterminate	0.2293	Benign	-7.88	Pathogenic	0.40	Neutral	0.02	Benign	0.79	Tolerated		
p.Arg99Thr	VUS	-18.03	Indeterminate	0.3207	Benign	-12.37	Pathogenic	0.53	Disease	0.96	Probably damaging	0.25	Tolerated		
p.Arg99Ser	VUS	-10.56	Indeterminate	0.4364	Ambiguous	-10.45	Pathogenic	0.30	Neutral	0.99	Probably damaging	0.3	Tolerated		
p.Arg99Ile	VUS	-7.17	Indeterminate	0.5564	Ambiguous	-15.76	Pathogenic	0.54	Disease	1.00	Probably damaging	0.08	Tolerated		
p.Arg99Met	VUS	-15.32	Indeterminate	0.5306	Ambiguous	-14.31	Pathogenic	0.51	Disease	0.99	Probably damaging	0.05	Deleterious		
p.Arg99His	VUS	-1.53	Neutral	0.1437	Benign	-8.54	Pathogenic	0.47	Neutral	0.96	Probably damaging	0.07	Tolerated		
p.Arg99Gln	VUS	-2.51	Neutral	0.1211	Benign	-8.63	Pathogenic	0.31	Neutral	0.49	Possibly damaging	0.68	Tolerated	14.30	Neutral
p.Arg99Pro	Likely pathogenic	-53.15	Deleterious	0.9146	Pathogenic	-15.88	Pathogenic	0.65	Disease	1.00	Probably damaging	0.13	Tolerated	22.40	Deleterious
p.Arg99Leu	VUS	-26.04	Indeterminate	0.2472	Benign	-11.47	Pathogenic	0.35	Neutral	1.00	Probably damaging	0.14	Tolerated	17.52	Deleterious
p.Arg99Asp	VUS	-30.54	Indeterminate	0.4469	Ambiguous	-12.39	Pathogenic	0.39	Neutral	0.99	Probably damaging	0.25	Tolerated		
p.Arg99Glu	VUS	-10.51	Indeterminate	0.2565	Benign	-11.97	Pathogenic	0.36	Neutral	0.59	Possibly damaging	1	Tolerated		
p.Arg99Ala	VUS	-33.08	Indeterminate	0.2617	Benign	-10.29	Pathogenic	0.39	Neutral	0.90	Possibly damaging	0.31	Tolerated		
p.Arg99Gly	VUS	-1.22	Neutral	0.1947	Benign	-9.37	Pathogenic	0.32	Neutral	1.00	Probably damaging	0.26	Tolerated	22.90	Deleterious
p.Arg99Val	VUS	-33.22	Indeterminate	0.4828	Ambiguous	-13.31	Pathogenic	0.57	Disease	1.00	Probably damaging	0.12	Tolerated		
p.Arg99Tyr	VUS	-3.07	Neutral	0.5805	Pathogenic	-14.21	Pathogenic	0.49	Neutral	0.93	Possibly damaging	0.03	Deleterious		
p.Arg99Cys	VUS	-21.73	Indeterminate	0.3804	Ambiguous	-10.67	Pathogenic	0.55	Disease	1.00	Probably damaging	0.02	Deleterious		
p.Arg99Trp	VUS	-21.35	Indeterminate	0.3409	Ambiguous	-11.97	Pathogenic	0.52	Disease	1.00	Probably damaging	0.01	Deleterious	26.30	Deleterious
p.Arg99Phe	VUS	-4.69	Neutral	0.6897	Pathogenic	-15.15	Pathogenic	0.55	Disease	0.93	Possibly damaging	0.02	Deleterious		
p.Ala100Asn	VUS	-1.08	Neutral	0.4496	Ambiguous	-6.83	Benign	0.33	Neutral	1.00	Probably damaging	0.32	Tolerated		
p.Ala100Lys	VUS	0.00	Neutral	0.6223	Pathogenic	-8.59	Pathogenic	0.53	Disease	1.00	Probably damaging	0.29	Tolerated		

p.Ala100Thr	VUS	-1.44	Neutral	0.2533	Benign	-10.20	Pathogenic	0.41	Neutral	1.00	Probably damaging	0.39	Tolerated	18.57	Deleterious
p.Ala100Arg	VUS	0.00	Neutral	0.3656	Ambiguous	-4.54	Benign	0.52	Disease	1.00	Probably damaging	0.35	Tolerated		
p.Ala100Ser	Benign	-0.01	Neutral	0.1502	Benign	-5.63	Benign	0.25	Neutral	0.97	Probably damaging	0.4	Tolerated	16.80	Deleterious
p.Ala100Ile	VUS	-0.08	Neutral	0.6148	Pathogenic	-13.16	Pathogenic	0.37	Neutral	1.00	Probably damaging	0.39	Tolerated		
p.Ala100Met	VUS	-0.05	Neutral	0.4862	Ambiguous	-10.76	Pathogenic	0.47	Neutral	1.00	Probably damaging	0.22	Tolerated		
p.Ala100His	VUS	0.00	Neutral	0.2773	Benign	-2.80	Benign	0.47	Neutral	1.00	Probably damaging	0.54	Tolerated		
p.Ala100Gln	VUS	-0.06	Neutral	0.3302	Benign	-8.15	Pathogenic	0.48	Neutral	1.00	Probably damaging	0.29	Tolerated		
p.Ala100Pro	VUS	-53.15	Deleterious	0.9671	Pathogenic	-12.76	Pathogenic	0.56	Disease	1.00	Probably damaging	0.2	Tolerated	22.60	Deleterious
p.Ala100Leu	VUS	-0.01	Neutral	0.4268	Ambiguous	-8.48	Pathogenic	0.34	Neutral	1.00	Probably damaging	0.65	Tolerated		
p.Ala100Asp	VUS	-1.07	Neutral	0.6518	Pathogenic	-11.39	Pathogenic	0.60	Disease	1.00	Probably damaging	0.21	Tolerated	22.80	Deleterious
p.Ala100Glu	VUS	-3.66	Neutral	0.4117	Ambiguous	-10.24	Pathogenic	0.32	Neutral	1.00	Probably damaging	0.28	Tolerated		
p.Ala100Gly	VUS	-53.15	Deleterious	0.1742	Benign	-5.19	Benign	0.27	Neutral	1.00	Probably damaging	0.34	Tolerated	19.20	Deleterious
p.Ala100Val	VUS	0.00	Neutral	0.2647	Benign	-8.74	Pathogenic	0.26	Neutral	1.00	Probably damaging	0.5	Tolerated	22.40	Deleterious
p.Ala100Tyr	VUS	-1.42	Neutral	0.5139	Ambiguous	-4.04	Benign	0.51	Disease	1.00	Probably damaging	1	Tolerated		
p.Ala100Cys	VUS	0.00	Neutral	0.4116	Ambiguous	-6.21	Benign	0.46	Neutral	1.00	Probably damaging	0.18	Tolerated		
p.Ala100Trp	VUS	0.00	Neutral	0.6812	Pathogenic	-4.53	Benign	0.55	Disease	1.00	Probably damaging	0.18	Tolerated		
p.Ala100Phe	VUS	0.00	Neutral	0.4423	Ambiguous	-4.04	Benign	0.36	Neutral	1.00	Probably damaging	0.7	Tolerated		
p.Gly101Asn	VUS	-6.43	Indeterminate	0.8106	Pathogenic	-12.58	Pathogenic	0.56	Disease	1.00	Probably damaging	0.09	Tolerated		
p.Gly101Lys	VUS	-13.62	Indeterminate	0.7814	Pathogenic	-12.51	Pathogenic	0.42	Neutral	1.00	Probably damaging	0.09	Tolerated		
p.Gly101Thr	VUS	-26.84	Indeterminate	0.8392	Pathogenic	-13.88	Pathogenic	0.47	Neutral	1.00	Probably damaging	0.04	Deleterious		
p.Gly101Arg	VUS	-10.33	Indeterminate	0.6226	Pathogenic	-9.29	Pathogenic	0.53	Disease	1.00	Probably damaging	0.04	Deleterious	26.75	Deleterious
p.Gly101Ser	VUS	-7.05	Indeterminate	0.5163	Ambiguous	-10.72	Pathogenic	0.54	Disease	1.00	Probably damaging	0.07	Tolerated		
p.Gly101Ile	VUS	-53.15	Deleterious	0.9774	Pathogenic	-19.70	Pathogenic	0.65	Disease	1.00	Probably damaging	0.01	Deleterious		
p.Gly101Met	VUS	-14.55	Indeterminate	0.9705	Pathogenic	-15.37	Pathogenic	0.66	Disease	1.00	Probably damaging	0.01	Deleterious		
p.Gly101His	VUS	-12.96	Indeterminate	0.8225	Pathogenic	-11.05	Pathogenic	0.40	Neutral	1.00	Probably damaging	0.03	Deleterious		
p.Gly101Gln	VUS	-7.18	Indeterminate	0.7227	Pathogenic	-12.08	Pathogenic	0.43	Neutral	1.00	Probably damaging	0.25	Tolerated		
p.Gly101Pro	VUS	-53.15	Deleterious	0.9934	Pathogenic	-17.02	Pathogenic	0.62	Disease	1.00	Probably damaging	0.04	Deleterious		
p.Gly101Leu	VUS	-53.15	Deleterious	0.9599	Pathogenic	-14.19	Pathogenic	0.65	Disease	1.00	Probably damaging	0.02	Deleterious		
p.Gly101Asp	VUS	-3.90	Neutral	0.6576	Pathogenic	-11.78	Pathogenic	0.61	Disease	1.00	Probably damaging	0.17	Tolerated		
p.Gly101Glu	VUS	-15.02	Indeterminate	0.759	Pathogenic	-13.11	Pathogenic	0.63	Disease	1.00	Probably damaging	0.35	Tolerated	26.80	Deleterious
p.Gly101Ala	VUS	-11.65	Indeterminate	0.6391	Pathogenic	-10.21	Pathogenic	0.51	Disease	1.00	Probably damaging	0.09	Tolerated	25.60	Deleterious
p.Gly101Val	VUS	-53.15	Deleterious	0.9342	Pathogenic	-15.06	Pathogenic	0.67	Disease	1.00	Probably damaging	0.02	Deleterious	26.60	Deleterious
p.Gly101Tyr	VUS	-33.17	Indeterminate	0.9532	Pathogenic	-15.85	Pathogenic	0.65	Disease	1.00	Probably damaging	0.01	Deleterious		
p.Gly101Cys	VUS	-5.64	Neutral	0.8484	Pathogenic	-12.72	Pathogenic	0.65	Disease	1.00	Probably damaging	0.01	Deleterious		
p.Gly101Trp	Pathogenic	-53.15	Deleterious	0.9495	Pathogenic	-14.73	Pathogenic	0.71	Disease	1.00	Probably damaging	0	Deleterious	28.90	Deleterious
p.Gly101Phe	VUS	-32.01	Indeterminate	0.9773	Pathogenic	-15.88	Pathogenic	0.65	Disease	1.00	Probably damaging	0.01	Deleterious		
p.Ala102Asn	VUS	-53.15	Deleterious	0.9525	Pathogenic	-17.84	Pathogenic	0.80	Disease	0.91	Possibly damaging	0	Deleterious		
p.Ala102Lys	VUS	-53.15	Deleterious	0.9904	Pathogenic	-22.38	Pathogenic	0.83	Disease	1.00	Probably damaging	0	Deleterious		
p.Ala102Thr	VUS	-53.15	Deleterious	0.6023	Pathogenic	-11.13	Pathogenic	0.79	Disease	0.92	Possibly damaging	0.01	Deleterious	28.40	Deleterious
p.Ala102Arg	VUS	-53.15	Deleterious	0.9445	Pathogenic	-18.16	Pathogenic	0.79	Disease	1.00	Probably damaging	0	Deleterious		
p.Ala102Ser	VUS	-53.15	Deleterious	0.2741	Benign	-9.44	Pathogenic	0.74	Disease	0.39	Benign	0.02	Deleterious	27.00	Deleterious
p.Ala102Ile	VUS	-53.15	Deleterious	0.9049	Pathogenic	-16.43	Pathogenic	0.83	Disease	1.00	Probably damaging	0	Deleterious		
p.Ala102Met	VUS	-53.15	Deleterious	0.8938	Pathogenic	-15.76	Pathogenic	0.78	Disease	1.00	Probably damaging	0	Deleterious		
p.Ala102His	VUS	-53.15	Deleterious	0.9635	Pathogenic	-18.36	Pathogenic	0.81	Disease	1.00	Probably damaging	0	Deleterious		
p.Ala102Gln	VUS	-53.15	Deleterious	0.9233	Pathogenic	-17.47	Pathogenic	0.84	Disease	1.00	Probably damaging	0	Deleterious		
p.Ala102Pro	VUS	-53.15	Deleterious	0.9353	Pathogenic	-13.22	Pathogenic	0.86	Disease	1.00	Probably damaging	0.01	Deleterious	28.30	Deleterious
p.Ala102Leu	VUS	-53.15	Deleterious	0.7181	Pathogenic	-14.15	Pathogenic	0.82	Disease	0.99	Probably damaging	0	Deleterious		
p.Ala102Asp	VUS	-53.15	Deleterious	0.9578	Pathogenic	-15.69	Pathogenic	0.88	Disease	0.99	Probably damaging	0	Deleterious		
p.Ala102Glu	VUS	-53.15	Deleterious	0.9565	Pathogenic	-16.01	Pathogenic	0.86	Disease	1.00	Probably damaging	0	Deleterious	27.70	Deleterious
p.Ala102Gly	VUS	-0.03	Neutral	0.2432	Benign	-8.21	Pathogenic	0.66	Disease	0.04	Benign	0.06	Tolerated	28.80	Deleterious
p.Ala102Val	VUS	-18.01	Indeterminate	0.639	Pathogenic	-9.19	Pathogenic	0.75	Disease	1.00	Probably damaging	0.01	Deleterious	28.70	Deleterious
p.Ala102Tyr	VUS	-53.15	Deleterious	0.9663	Pathogenic	-19.07	Pathogenic	0.83	Disease	1.00	Probably damaging	0	Deleterious		
p.Ala102Cys	VUS	-12.41	Indeterminate	0.6812	Pathogenic	-10.37	Pathogenic	0.75	Disease	1.00	Probably damaging	0	Deleterious		
p.Ala102Trp	VUS	-53.15	Deleterious	0.981	Pathogenic	-17.78	Pathogenic	0.83	Disease	1.00	Probably damaging	0	Deleterious		
p.Ala102Phe	VUS	-53.15	Deleterious	0.9308	Pathogenic	-18.43	Pathogenic	0.84	Disease	1.00	Probably damaging	0	Deleterious		
p.Arg103Asn	VUS	-0.01	Neutral	0.4251	Ambiguous	-11.54	Pathogenic	0.38	Neutral	1.00	Probably damaging	0.89	Tolerated		
p.Arg103Lys	VUS	-0.48	Neutral	0.2025	Benign	-7.78	Pathogenic	0.36	Neutral	0.78	Possibly damaging	0.26	Tolerated		
p.Arg103Thr	VUS	-6.16	Indeterminate	0.1995	Benign	-10.46	Pathogenic	0.50	Neutral	1.00	Probably damaging	0.17	Tolerated		
p.Arg103Ser	VUS	-0.01	Neutral	0.2587	Benign	-9.19	Pathogenic	0.30	Neutral	1.00	Probably damaging	0.41	Tolerated		
p.Arg103Ile	VUS	-3.14	Neutral	0.3389	Benign	-12.16	Pathogenic	0.53	Disease	1.00	Probably damaging	0.02	Deleterious		
p.Arg103Met	VUS	-3.90	Neutral	0.3543	Ambiguous	-11.90	Pathogenic	0.54	Disease	1.00	Probably damaging	0.02	Deleterious		
p.Arg103His	VUS	-0.99	Neutral	0.1323	Benign	-7.95	Pathogenic	0.32	Neutral	0.99	Probably damaging	0.16	Tolerated		
p.Arg103Gln	VUS	-0.97	Neutral	0.1029	Benign	-8.15	Pathogenic	0.28	Neutral	0.94	Possibly damaging	0.17	Tolerated	19.34	Deleterious
p.Arg103Pro	VUS	-0.63	Neutral	0.3065	Benign	-10.10	Pathogenic	0.58	Disease	1.00	Probably damaging	0.13	Tolerated	22.90	Deleterious
p.Arg103Leu	VUS	-0.01	Neutral	0.1593	Benign	-9.92	Pathogenic	0.55	Disease	1.00	Probably damaging	0.04	Deleterious	20.30	Deleterious
p.Arg103Asp	VUS	0.00	Neutral	0.3581	Ambiguous	-10.15	Pathogenic	0.29	Neutral	1.00	Probably damaging	1	Tolerated		
p.Arg103Glu	VUS	-2.72	Neutral	0.2591	Benign	-10.02	Pathogenic	0.37	Neutral	0.90	Possibly damaging	0.28	Tolerated		
p.Arg103Ala	VUS	0.00	Neutral	0.1801	Benign	-8.30	Pathogenic	0.45	Neutral	0.98	Probably damaging	0.17	Tolerated		
p.Arg103Gly	VUS	-0.01	Neutral	0.1779	Benign	-10.95	Pathogenic	0.32	Neutral	1.00	Probably damaging	0.52	Tolerated	22.60	Deleterious
p.Arg103Val	VUS	-0.16	Neutral	0.2474	Benign	-9.95	Pathogenic	0.55	Disease	1.00	Probably damaging	0.03	Deleterious		
p.Arg103Tyr	VUS	-0.63	Neutral	0.4737	Ambiguous	-11.61	Pathogenic	0.50	Neutral	0.99	Probably damaging	0.08	Tolerated		
p.Arg103Cys	VUS	-0.01	Neutral	0.2144	Benign	-9.27	Pathogenic	0.56	Disease	1.00	Probably damaging	0.04	Deleterious		
p.Arg103Trp	VUS	-0.24	Neutral	0.2676	Benign	-11.13	Pathogenic	0.54	Disease	1.00	Probably damaging	0.02	Deleterious	26.40	Deleterious
p.Arg103Phe	VUS	0.00	Neutral	0.5257	Ambiguous	-12.21	Pathogenic	0.53	Disease	0.99	Probably damaging	0.03	Deleterious		
p.Leu104Asn	VUS	0.00	Neutral	0.8502	Pathogenic	-17.13	Pathogenic	0.62	Disease	1.00	Probably damaging	0	Deleterious		
p.Leu104Lys	VUS	-11.35	Indeterminate	0.7882	Pathogenic	-17.24	Pathogenic	0.64	Disease	0.98	Probably damaging	0.01	Deleterious		
p.Leu104Thr	VUS	0.00	Neutral	0.3934	Ambiguous	-9.66	Pathogenic	0.61	Disease	1.00	Probably damaging	0.02	Deleterious		
p.Leu104Arg	VUS	-53.15	Deleterious	0.5814	Pathogenic	-13.14	Pathogenic	0.73	Disease	1.00	Probably damaging	0.01	Deleterious	28.00	Deleterious
p.Leu104Ser	VUS	0.00	Neutral	0.7933	Pathogenic	-12.46	Pathogenic	0.65	Disease	1.00	Probably damaging	0.01	Deleterious		
p.Leu104Ile	VUS	0.00	Neutral	0.2168	Benign	-7.73	Pathogenic	0.45	Neutral	1.00	Probably damaging	0.41	Tolerated		
p.Leu104Met	VUS	0.00	Neutral	0.2598	Benign	-5.42	Benign	0.46	Neutral	1.00	Probably damaging	0.04	Deleterious	25.70	Deleterious
p.Leu104His	VUS	0.00	Neutral	0.7805	Pathogenic	-14.47	Pathogenic	0.62	Disease	1.00	Probably damaging	0	Deleterious		
p.Leu104Gln	VUS	0.00	Neutral	0.6621	Pathogenic	-13.35	Pathogenic	0.69	Disease	1.00	Probably damaging	0.01	Deleterious	27.40	Deleterious
p.Leu104Pro	VUS	-0.01	Neutral	0.2401	Benign	-9.68	Pathogenic	0.27	Neutral	1.00	Probably damaging	0.01	Deleterious	24.50	Deleterious
p.Leu104Asp	VUS	0.00	Neutral	0.9278	Pathogenic	-16.57	Pathogenic	0.64	Disease	1.00	Probably damaging	0	Deleterious		
p.Leu104Glu	VUS	0.00	Neutral	0.7798	Pathogenic	-15.90	Pathogenic	0.64	Disease	1.00	Probably damaging	0.01	Deleterious		
p.Leu104Ala	VUS	0.00	Neutral	0.5071	Ambiguous	-10.47	Pathogenic	0.37	Neutral	1.00	Probably damaging	0.02	Deleterious		
p.Leu104Gly	VUS	-53.15	Deleterious	0.7488	Pathogenic	-12.87	Pathogenic	0.61	Disease	1.00	Probably damaging	0.01	Deleterious		
p.Leu104Val	VUS	0.00	Neutral	0.1816	Benign	-7.26	Benign	0.16	Neutral	0.99	Probably damaging	0.84	Tolerated	23.50	Deleterious
p.Leu104Tyr	VUS	0.00	Neutral	0.8168	Pathogenic	-14.67	Pathogenic	0.55	Disease	0.99	Probably damaging	0.01	Deleterious		
p.Leu104Cys	VUS	0.00	Neutral	0.7713	Pathogenic	-11.82	Pathogenic	0.56	Disease	1.00	Probably damaging	0.01	Deleterious		
p.Leu104Trp	VUS	0.00	Neutral	0.5661	Pathogenic	-12.84	Pathogenic	0.57	Disease	1.00	Probably damaging	0	Deleterious		
p.Leu104Phe	VUS	0.00	Neutral	0.439	Ambiguous	-10.35	Pathogenic	0.50	Neutral	1.00	Probably damaging	0.03	Deleterious		
p.Asp105Asn	VUS	-2.86	Neutral	0.2727	Benign	-8.41	Pathogenic	0.24	Neutral	0.98	Probably damaging	1	Tolerated	19.47	Deleterious
p.Asp105Lys	VUS	-15.87	Indeterminate	0.8635	Pathogenic	-11.29	Pathogenic	0.50	Neutral	1.00	Probably damaging	0.15	Tolerated		
p.Asp105Thr	VUS	-12.21	Indeterminate	0.5806	Pathogenic	-7.97	Pathogenic	0.47	Neutral	1.00	Probably damaging	0.12	Tolerated		
p.Asp105Arg	VUS	-32.93	Indeterminate	0.7381	Pathogenic	-7.21	Benign	0.48	Neutral	1.00	Probably damaging	0.08	Tolerated		
p.Asp105Ser	VUS	-53.15	Deleterious	0.4241	Ambiguous	-7.28	Benign	0.43	Neutral	1.00	Probably damaging	0.26	Tolerated		
p.Asp105Ile	VUS	-53.15	Deleterious	0.861	Pathogenic	-13.45	Pathogenic	0.50	Neutral	1.00	Probably damaging	0.02	Deleterious		
p.Asp105Met	VUS	-15.69	Indeterminate	0.9269	Pathogenic	-11.01	Pathogenic	0.52	Disease	1.00	Probably damaging	0.01	Deleterious		
p.Asp105His	VUS	-11.71	Indeterminate	0.5656	Pathogenic	-6.84	Benign	0.30	Neutral	1.00	Probably damaging	0.1	Tolerated	23.10	Deleterious
p.Asp105Gln	VUS	-18.85	Indeterminate	0.6735	Pathogenic	-7.92	Pathogenic	0.29	Neutral	1.00	Probably damaging	0.1	Tolerated		
p.Asp105Pro	VUS	-53.15	Deleterious	0.9092	Pathogenic	-11.63	Pathogenic	0.51	Disease	0.97	Probably damaging	0.08	Tolerated		
p.Asp105Leu	VUS	-18.17	Indeterminate	0.8148	Pathogenic	-8.91	Pathogenic	0.50	Disease	1.00	Probably damaging	0.18	Tolerated		
p.Asp105Glu	VUS	-17.38	Indeterminate	0.4885	Ambiguous	-6.27	Benign	0.27	Neutral	0.92	Possibly damaging	0.2	Tolerated	13.08	Neutral
p.Asp105Ala	VUS	-6.08	Indeterminate	0.4992	Ambiguous	-6.15	Benign	0.42	Neutral	1.00	Probably damaging	0.11	Tolerated	24.10	Deleterious
p.Asp105Gly	VUS	-53.15	Deleterious	0.4131	Ambiguous	-4.39	Benign	0.38	Neutral	1.00	Probably damaging	0.33	Tolerated	24.60	Deleterious
p.Asp105Val	VUS	-29.16	Indeterminate	0.6699	Pathogenic	-9.73	Pathogenic	0.53	Disease	1.00	Probably damaging	0.03	Deleterious	25.90	Deleterious
p.Asp105Tyr	VUS	0.00	Neutral	0.6581	Pathogenic	-11.05	Pathogenic	0.57	Disease	1.00	Probably damaging	0.05	Deleterious	23.40	Deleterious
p.Asp105Cys	VUS	-0.23	Neutral	0.888	Pathogenic	-9.07	Pathogenic	0.49	Neutral	1.00	Probably damaging	0.02	Deleterious		
p.Asp105Trp	VUS	-6.73	Indeterminate	0.9483	Pathogenic	-10.19	Pathogenic	0.42	Neutral	1.00	Probably damaging	0.01	Deleterious		

p.Asp105Phe	VUS	-16.15	Indeterminate	0.9163	Pathogenic	-12.06	Pathogenic	0.51	Disease	1.00	Probably damaging	0.03	Deleterious		
p.Val106Asn	VUS	-8.17	Indeterminate	0.5709	Pathogenic	-13.95	Pathogenic	0.51	Disease	1.00	Probably damaging	0.03	Deleterious		
p.Val106Lys	VUS	-16.70	Indeterminate	0.5522	Ambiguous	-15.08	Pathogenic	0.55	Disease	1.00	Probably damaging	0.05	Deleterious		
p.Val106Thr	VUS	-1.68	Neutral	0.2756	Benign	-7.63	Pathogenic	0.30	Neutral	1.00	Probably damaging	0.16	Tolerated		
p.Val106Arg	VUS	-24.14	Indeterminate	0.3403	Ambiguous	-10.26	Pathogenic	0.51	Disease	1.00	Probably damaging	0.04	Deleterious		
p.Val106Ser	VUS	-0.72	Neutral	0.3524	Ambiguous	-8.63	Pathogenic	0.48	Neutral	1.00	Probably damaging	0.14	Tolerated		
p.Val106Ile	VUS	-1.09	Neutral	0.1505	Benign	-7.97	Pathogenic	0.11	Neutral	0.97	Probably damaging	0.55	Tolerated		
p.Val106Met	VUS	-0.81	Neutral	0.3682	Ambiguous	-7.68	Pathogenic	0.36	Neutral	1.00	Probably damaging	0.07	Tolerated	25.60	Deleterious
p.Val106His	VUS	-0.71	Neutral	0.6751	Pathogenic	-10.67	Pathogenic	0.51	Disease	1.00	Probably damaging	0.03	Deleterious		
p.Val106Gln	VUS	-1.67	Neutral	0.3212	Benign	-8.12	Pathogenic	0.30	Neutral	1.00	Probably damaging	0.04	Deleterious		
p.Val106Pro	VUS	-21.83	Indeterminate	0.8011	Pathogenic	-9.45	Pathogenic	0.51	Disease	1.00	Probably damaging	0.05	Deleterious		
p.Val106Leu	VUS	-9.78	Indeterminate	0.3179	Benign	-5.92	Benign	0.09	Neutral	0.98	Probably damaging	0.25	Tolerated	21.55	Deleterious
p.Val106Asp	VUS	-1.29	Neutral	0.4428	Ambiguous	-10.12	Pathogenic	0.59	Disease	1.00	Probably damaging	0.02	Deleterious		
p.Val106Glu	VUS	-29.44	Indeterminate	0.2415	Benign	-8.47	Pathogenic	0.60	Disease	1.00	Probably damaging	0.04	Deleterious	26.30	Deleterious
p.Val106Ala	VUS	-5.62	Neutral	0.2098	Benign	-4.32	Benign	0.13	Neutral	1.00	Probably damaging	0.4	Tolerated	23.40	Deleterious
p.Val106Gly	VUS	-9.59	Indeterminate	0.2586	Benign	-8.95	Pathogenic	0.43	Neutral	1.00	Probably damaging	0.03	Deleterious	26.10	Deleterious
p.Val106Tyr	VUS	-1.56	Neutral	0.7948	Pathogenic	-14.22	Pathogenic	0.50	Neutral	1.00	Probably damaging	0.06	Tolerated		
p.Val106Cys	VUS	-1.64	Neutral	0.6917	Pathogenic	-9.60	Pathogenic	0.44	Neutral	1.00	Probably damaging	0.05	Deleterious		
p.Val106Trp	VUS	-13.39	Indeterminate	0.8982	Pathogenic	-12.41	Pathogenic	0.47	Neutral	1.00	Probably damaging	0.01	Deleterious		
p.Val106Phe	VUS	-1.15	Neutral	0.3839	Ambiguous	-11.84	Pathogenic	0.49	Neutral	1.00	Probably damaging	0.17	Tolerated		
p.Arg107Asn	VUS	-2.70	Neutral	0.6572	Pathogenic	-14.08	Pathogenic	0.44	Neutral	0.27	Benign	0.5	Tolerated		
p.Arg107Lys	VUS	-0.88	Neutral	0.2096	Benign	-7.96	Pathogenic	0.20	Neutral	0.02	Benign	0.92	Tolerated		
p.Arg107Thr	VUS	-0.06	Neutral	0.2177	Benign	-9.41	Pathogenic	0.43	Neutral	0.43	Benign	0.6	Tolerated		
p.Arg107Ser	VUS	-3.51	Neutral	0.4114	Ambiguous	-10.34	Pathogenic	0.38	Neutral	0.86	Possibly damaging	0.73	Tolerated	23.80	Deleterious
p.Arg107Ile	VUS	-1.12	Neutral	0.3829	Ambiguous	-13.41	Pathogenic	0.49	Neutral	0.93	Possibly damaging	0.22	Tolerated		
p.Arg107Met	VUS	-0.08	Neutral	0.4767	Ambiguous	-11.83	Pathogenic	0.49	Neutral	0.72	Possibly damaging	0.12	Tolerated		
p.Arg107His	VUS	-1.66	Neutral	0.2041	Benign	-8.80	Pathogenic	0.19	Neutral	0.00	Benign	0.16	Tolerated	18.27	Deleterious
p.Arg107Gln	VUS	0.00	Neutral	0.1418	Benign	-8.82	Pathogenic	0.19	Neutral	0.02	Benign	0.6	Tolerated		
p.Arg107Pro	VUS	-0.08	Neutral	0.1271	Benign	-6.34	Benign	0.22	Neutral	0.98	Probably damaging	0.29	Tolerated	16.64	Deleterious
p.Arg107Leu	VUS	-2.08	Neutral	0.2011	Benign	-8.63	Pathogenic	0.26	Neutral	0.88	Possibly damaging	0.35	Tolerated	22.00	Deleterious
p.Arg107Asp	VUS	-1.62	Neutral	0.6577	Pathogenic	-14.97	Pathogenic	0.47	Neutral	0.76	Possibly damaging	0.61	Tolerated		
p.Arg107Glu	VUS	-5.67	Neutral	0.3408	Ambiguous	-10.44	Pathogenic	0.29	Neutral	0.04	Benign	1	Tolerated		
p.Arg107Ala	VUS	-0.01	Neutral	0.1877	Benign	-7.89	Pathogenic	0.24	Neutral	0.24	Benign	0.77	Tolerated		
p.Arg107Gly	VUS	-1.60	Neutral	0.1981	Benign	-9.62	Pathogenic	0.43	Neutral	0.86	Possibly damaging	0.39	Tolerated	24.10	Deleterious
p.Arg107Val	VUS	-0.10	Neutral	0.3024	Benign	-10.32	Pathogenic	0.51	Disease	0.73	Possibly damaging	0.35	Tolerated		
p.Arg107Tyr	VUS	-1.06	Neutral	0.5547	Ambiguous	-10.26	Pathogenic	0.45	Neutral	0.04	Benign	0.12	Tolerated		
p.Arg107Cys	VUS	-8.51	Indeterminate	0.2239	Benign	-9.83	Pathogenic	0.50	Neutral	0.22	Benign	0.08	Tolerated	28.50	Deleterious
p.Arg107Trp	VUS	-0.08	Neutral	0.3155	Benign	-12.74	Pathogenic	0.49	Neutral	0.86	Possibly damaging	0.03	Deleterious		
p.Arg107Phe	VUS	-1.19	Neutral	0.6012	Pathogenic	-10.67	Pathogenic	0.51	Disease	0.07	Benign	0.11	Tolerated		
p.Asp108Asn	VUS	-0.78	Neutral	0.8442	Pathogenic	-9.82	Pathogenic	0.70	Disease	1.00	Probably damaging	0.43	Tolerated	31.00	Deleterious
p.Asp108Lys	VUS	-53.15	Deleterious	0.9973	Pathogenic	-18.69	Pathogenic	0.78	Disease	1.00	Probably damaging	0.02	Deleterious		
p.Asp108Thr	VUS	-31.02	Indeterminate	0.9831	Pathogenic	-13.75	Pathogenic	0.77	Disease	1.00	Probably damaging	0.02	Deleterious		
p.Asp108Arg	VUS	-53.15	Deleterious	0.9944	Pathogenic	-19.00	Pathogenic	0.78	Disease	1.00	Probably damaging	0.01	Deleterious		
p.Asp108Ser	VUS	-53.15	Deleterious	0.9524	Pathogenic	-14.48	Pathogenic	0.74	Disease	1.00	Probably damaging	0.22	Tolerated		
p.Asp108Ile	VUS	-53.15	Deleterious	0.9979	Pathogenic	-19.67	Pathogenic	0.82	Disease	1.00	Probably damaging	0	Deleterious		
p.Asp108Met	VUS	-53.15	Deleterious	0.9983	Pathogenic	-18.14	Pathogenic	0.81	Disease	1.00	Probably damaging	0	Deleterious		
p.Asp108His	VUS	-53.15	Deleterious	0.9917	Pathogenic	-16.58	Pathogenic	0.87	Disease	1.00	Probably damaging	0.02	Deleterious	29.20	Deleterious
p.Asp108Gln	VUS	-53.15	Deleterious	0.9959	Pathogenic	-18.53	Pathogenic	0.74	Disease	1.00	Probably damaging	0.02	Deleterious		
p.Asp108Pro	VUS	-53.15	Deleterious	0.9971	Pathogenic	-20.06	Pathogenic	0.79	Disease	0.97	Probably damaging	0.01	Deleterious		
p.Asp108Leu	VUS	-53.15	Deleterious	0.9897	Pathogenic	-19.60	Pathogenic	0.81	Disease	1.00	Probably damaging	0	Deleterious		
p.Asp108Glu	VUS	-11.70	Indeterminate	0.9837	Pathogenic	-10.69	Pathogenic	0.73	Disease	0.82	Possibly damaging	0.03	Deleterious	22.30	Deleterious
p.Asp108Ala	VUS	-53.15	Deleterious	0.9849	Pathogenic	-14.98	Pathogenic	0.83	Disease	1.00	Probably damaging	0.02	Deleterious	29.00	Deleterious
p.Asp108Gly	VUS	-53.15	Deleterious	0.9533	Pathogenic	-13.96	Pathogenic	0.87	Disease	1.00	Probably damaging	0.06	Tolerated	29.60	Deleterious
p.Asp108Val	VUS	-53.15	Deleterious	0.989	Pathogenic	-17.56	Pathogenic	0.90	Disease	1.00	Probably damaging	0	Deleterious	29.30	Deleterious
p.Asp108Tyr	VUS	-53.15	Deleterious	0.9811	Pathogenic	-16.66	Pathogenic	0.73	Disease	1.00	Probably damaging	0.01	Deleterious	31.00	Deleterious
p.Asp108Cys	VUS	-53.15	Deleterious	0.9907	Pathogenic	-12.83	Pathogenic	0.82	Disease	1.00	Probably damaging	0	Deleterious		
p.Asp108Trp	VUS	-53.15	Deleterious	0.9992	Pathogenic	-21.27	Pathogenic	0.81	Disease	1.00	Probably damaging	0	Deleterious		
p.Asp108Phe	VUS	-53.15	Deleterious	0.9984	Pathogenic	-21.33	Pathogenic	0.83	Disease	1.00	Probably damaging	0	Deleterious		
p.Ala109Asn	VUS	0.00	Neutral	0.4117	Ambiguous	-13.00	Pathogenic	0.22	Neutral	0.91	Possibly damaging	0.87	Tolerated		
p.Ala109Lys	VUS	-0.84	Neutral	0.5698	Pathogenic	-12.24	Pathogenic	0.25	Neutral	0.99	Probably damaging	0.74	Tolerated		
p.Ala109Thr	VUS	-0.16	Neutral	0.1665	Benign	-9.23	Pathogenic	0.20	Neutral	0.07	Benign	0.49	Tolerated	20.30	Deleterious
p.Ala109Arg	VUS	-0.44	Neutral	0.345	Ambiguous	-8.44	Pathogenic	0.28	Neutral	1.00	Probably damaging	0.38	Tolerated		
p.Ala109Ser	VUS	0.00	Neutral	0.1441	Benign	-6.48	Benign	0.23	Neutral	0.25	Benign	0.91	Tolerated	18.06	Deleterious
p.Ala109Ile	VUS	0.00	Neutral	0.4344	Ambiguous	-13.28	Pathogenic	0.36	Neutral	0.99	Probably damaging	0.13	Tolerated		
p.Ala109Met	VUS	0.00	Neutral	0.491	Ambiguous	-12.83	Pathogenic	0.28	Neutral	1.00	Probably damaging	0.08	Tolerated		
p.Ala109His	VUS	0.00	Neutral	0.5129	Ambiguous	-12.47	Pathogenic	0.25	Neutral	1.00	Probably damaging	0.16	Tolerated		
p.Ala109Gln	VUS	-0.04	Neutral	0.3616	Ambiguous	-10.25	Pathogenic	0.21	Neutral	1.00	Probably damaging	0.42	Tolerated		
p.Ala109Pro	VUS	-53.15	Deleterious	0.376	Ambiguous	-8.64	Pathogenic	0.35	Neutral	1.00	Probably damaging	0.29	Tolerated	22.90	Deleterious
p.Ala109Leu	VUS	-0.83	Neutral	0.3332	Benign	-11.69	Pathogenic	0.27	Neutral	0.98	Probably damaging	0.22	Tolerated		
p.Ala109Asp	VUS	-0.01	Neutral	0.4589	Ambiguous	-13.00	Pathogenic	0.37	Neutral	0.98	Probably damaging	0.73	Tolerated	18.50	Deleterious
p.Ala109Glu	VUS	-1.19	Neutral	0.3553	Ambiguous	-11.40	Pathogenic	0.31	Neutral	0.99	Probably damaging	0.69	Tolerated		
p.Ala109Gly	VUS	0.00	Neutral	0.142	Benign	-5.89	Benign	0.22	Neutral	0.96	Probably damaging	0.67	Tolerated	16.99	Deleterious
p.Ala109Val	VUS	-0.01	Neutral	0.2152	Benign	-9.47	Pathogenic	0.19	Neutral	0.92	Possibly damaging	0.22	Tolerated	18.10	Deleterious
p.Ala109Tyr	VUS	-0.01	Neutral	0.6138	Pathogenic	-15.63	Pathogenic	0.39	Neutral	1.00	Probably damaging	0.08	Tolerated		
p.Ala109Cys	VUS	0.00	Neutral	0.4761	Ambiguous	-9.32	Pathogenic	0.37	Neutral	1.00	Probably damaging	0.08	Tolerated		
p.Ala109Trp	VUS	-0.02	Neutral	0.7885	Pathogenic	-14.26	Pathogenic	0.43	Neutral	1.00	Probably damaging	0.02	Deleterious		
p.Ala109Phe	VUS	-0.40	Neutral	0.5707	Pathogenic	-15.34	Pathogenic	0.40	Neutral	1.00	Probably damaging	0.06	Tolerated		
p.Trp110Asn	VUS	-0.41	Neutral	0.7983	Pathogenic	-8.67	Pathogenic	0.31	Neutral	1.00	Probably damaging	0.14	Tolerated		
p.Trp110Lys	VUS	-1.92	Neutral	0.8356	Pathogenic	-7.22	Benign	0.46	Neutral	1.00	Probably damaging	0.21	Tolerated		
p.Trp110Thr	VUS	-0.13	Neutral	0.699	Pathogenic	-6.84	Benign	0.43	Neutral	1.00	Probably damaging	0.14	Tolerated		
p.Trp110Arg	VUS	-0.12	Neutral	0.618	Pathogenic	-5.19	Benign	0.23	Neutral	1.00	Probably damaging	0.07	Tolerated	18.20	Deleterious
p.Trp110Ser	VUS	-1.65	Neutral	0.4616	Ambiguous	-5.68	Benign	0.24	Neutral	1.00	Probably damaging	0.16	Tolerated	22.30	Deleterious
p.Trp110Ile	VUS	-0.14	Neutral	0.8704	Pathogenic	-8.29	Pathogenic	0.47	Neutral	1.00	Probably damaging	0.03	Deleterious		
p.Trp110Met	VUS	-1.18	Neutral	0.8504	Pathogenic	-5.64	Benign	0.48	Neutral	1.00	Probably damaging	0.02	Deleterious		
p.Trp110His	VUS	-4.10	Neutral	0.5532	Ambiguous	-6.71	Benign	0.45	Neutral	1.00	Probably damaging	0.06	Tolerated		
p.Trp110Gln	VUS	-1.38	Neutral	0.7159	Pathogenic	-6.04	Benign	0.31	Neutral	1.00	Probably damaging	0.25	Tolerated		
p.Trp110Pro	VUS	-6.95	Indeterminate	0.9657	Pathogenic	-9.39	Pathogenic	0.42	Neutral	1.00	Probably damaging	0.12	Tolerated		
p.Trp110Leu	VUS	-0.06	Neutral	0.6424	Pathogenic	-4.43	Benign	0.31	Neutral	1.00	Probably damaging	0.05	Deleterious	21.60	Deleterious
p.Trp110Asp	VUS	-0.01	Neutral	0.7311	Pathogenic	-7.29	Benign	0.29	Neutral	1.00	Probably damaging	0.54	Tolerated		
p.Trp110Glu	VUS	-1.76	Neutral	0.7096	Pathogenic	-7.33	Benign	0.32	Neutral	1.00	Probably damaging	1	Tolerated		
p.Trp110Ala	VUS	-6.88	Indeterminate	0.5241	Ambiguous	-4.22	Benign	0.29	Neutral	1.00	Probably damaging	0.21	Tolerated		
p.Trp110Gly	VUS	-0.16	Neutral	0.4853	Ambiguous	-5.73	Benign	0.25	Neutral	0.38	Benign	0.11	Tolerated	18.78	Deleterious
p.Trp110Val	VUS	-0.02	Neutral	0.7336	Pathogenic	-6.25	Benign	0.45	Neutral	1.00	Probably damaging	0.06	Tolerated		
p.Trp110Tyr	VUS	-0.11	Neutral	0.368	Ambiguous	-6.32	Benign	0.42	Neutral	1.00	Probably damaging	0.03	Deleterious		
p.Trp110Cys	VUS	-0.29	Neutral	0.8142	Pathogenic	-5.17	Benign	0.45	Neutral	1.00	Probably damaging	0.01	Deleterious	25.85	Deleterious
p.Trp110Phe	VUS	-0.03	Neutral	0.2728	Benign	-2.04	Benign	0.33	Neutral	1.00	Probably damaging	0.02	Deleterious		
p.Gly111Asn	VUS	-0.20	Neutral	0.46	Ambiguous	-8.63	Pathogenic	0.34	Neutral	1.00	Probably damaging	0	Deleterious		
p.Gly111Lys	VUS	-4.17	Neutral	0.8154	Pathogenic	-13.08	Pathogenic	0.46	Neutral	1.00	Probably damaging	0	Deleterious		
p.Gly111Thr	VUS	-33.22	Indeterminate	0.774	Pathogenic	-13.32	Pathogenic	0.62	Disease	1.00	Probably damaging	0	Deleterious		
p.Gly111Arg	VUS	-1.14	Neutral	0.6573	Pathogenic	-10.53	Pathogenic	0.63	Disease	1.00	Probably damaging	0	Deleterious	32.00	Deleterious
p.Gly111Ser	VUS	-0.30	Neutral	0.3456	Ambiguous	-8.72	Pathogenic	0.43	Neutral	1.00	Probably damaging	0	Deleterious	31.00	Deleterious
p.Gly111Ile	VUS	-53.15	Deleterious	0.9449	Pathogenic	-17.92	Pathogenic	0.66	Disease	1.00	Probably damaging	0	Deleterious		
p.Gly111Met	VUS	-0.76	Neutral	0.9062	Pathogenic	-14.61	Pathogenic	0.66	Disease	1.00	Probably damaging	0	Deleterious		
p.Gly111His	VUS	-6.80	Indeterminate	0.6661	Pathogenic	-10.62	Pathogenic	0.63	Disease	1.00	Probably damaging	0	Deleterious		
p.Gly111Gln	VUS	-0.03	Neutral	0.5589	Ambiguous	-10.39	Pathogenic	0.60	Disease	1.00	Probably damaging	0	Deleterious		
p.Gly111Pro	VUS	-53.15	Deleterious	0.994	Pathogenic	-17.33	Pathogenic	0.61	Disease	1.00	Probably damaging	0	Deleterious		
p.Gly111Leu	VUS	-0.79	Neutral	0.8297	Pathogenic	-13.47	Pathogenic	0.65	Disease	1.00	Probably damaging	0	Deleterious		
p.Gly111Asp	VUS	-0.28	Neutral	0.3488	Ambiguous	-5.39	Benign	0.39	Neutral	1.00	Probably damaging	0	Deleterious	23.30	Deleterious
p.Gly111Glu	VUS	-1.42	Neutral	0.4928	Ambiguous	-8.31	Pathogenic	0.65	Disease	1.00	Probably damaging	0	Deleterious		
p.Gly111Ala	VUS	-4.33	Neutral	0.5207	Ambiguous	-9.36	Pathogenic	0.36	Neutral	1.00	Probably damaging	0	Deleterious	23.00	Deleterious
p.Gly111Val	VUS	-53.15	Deleterious	0.8854	Pathogenic	-13.29	Pathogenic	0.71	Disease	1.00	Probably damaging	0	Deleterious	25.40	Deleterious

p.Gly111Tyr	VUS	-1.17	Neutral	0.8014	Pathogenic	-14.27	Pathogenic	0.66	Disease	1.00	Probably damaging	0	Deleterious		
p.Gly111Cys	VUS	-0.80	Neutral	0.6902	Pathogenic	-11.37	Pathogenic	0.66	Disease	1.00	Probably damaging	0	Deleterious	32.00	Deleterious
p.Gly111Trp	VUS	-0.54	Neutral	0.8024	Pathogenic	-14.45	Pathogenic	0.69	Disease	1.00	Probably damaging	0	Deleterious		
p.Gly111Phe	VUS	-0.47	Neutral	0.9281	Pathogenic	-15.88	Pathogenic	0.66	Disease	1.00	Probably damaging	0	Deleterious		
p.Arg112Asn	VUS	-0.21	Neutral	0.8545	Pathogenic	-11.02	Pathogenic	0.34	Neutral	1.00	Probably damaging	0.9	Tolerated		
p.Arg112Lys	VUS	-2.10	Neutral	0.4874	Ambiguous	-6.79	Benign	0.50	Neutral	0.99	Probably damaging	0.92	Tolerated		
p.Arg112Thr	VUS	-0.24	Neutral	0.8492	Pathogenic	-12.48	Pathogenic	0.67	Disease	1.00	Probably damaging	0.46	Tolerated		
p.Arg112Ser	VUS	-0.35	Neutral	0.8846	Pathogenic	-11.11	Pathogenic	0.35	Neutral	1.00	Probably damaging	0.66	Tolerated	27.30	Deleterious
p.Arg112Ile	VUS	-1.31	Neutral	0.782	Pathogenic	-15.13	Pathogenic	0.68	Disease	1.00	Probably damaging	0.14	Tolerated		
p.Arg112Met	VUS	-0.01	Neutral	0.8234	Pathogenic	-12.83	Pathogenic	0.67	Disease	1.00	Probably damaging	0.08	Tolerated		
p.Arg112His	VUS	-1.24	Neutral	0.2973	Benign	-8.43	Pathogenic	0.32	Neutral	1.00	Probably damaging	0.12	Tolerated	27.60	Deleterious
p.Arg112Gln	VUS	-2.70	Neutral	0.2994	Benign	-9.10	Pathogenic	0.56	Disease	1.00	Probably damaging	0.47	Tolerated		
p.Arg112Pro	VUS	-53.15	Deleterious	0.9671	Pathogenic	-14.73	Pathogenic	0.76	Disease	1.00	Probably damaging	0.22	Tolerated	32.00	Deleterious
p.Arg112Leu	VUS	-14.52	Indeterminate	0.5683	Pathogenic	-10.93	Pathogenic	0.70	Disease	1.00	Probably damaging	0.24	Tolerated	31.00	Deleterious
p.Arg112Asp	VUS	-7.02	Indeterminate	0.915	Pathogenic	-13.50	Pathogenic	0.69	Disease	1.00	Probably damaging	0.7	Tolerated		
p.Arg112Glu	VUS	-15.47	Indeterminate	0.7571	Pathogenic	-12.45	Pathogenic	0.61	Disease	1.00	Probably damaging	0.79	Tolerated		
p.Arg112Ala	VUS	-4.46	Neutral	0.8545	Pathogenic	-11.59	Pathogenic	0.35	Neutral	1.00	Probably damaging	0.71	Tolerated		
p.Arg112Gly	Pathogenic	-29.42	Indeterminate	0.7369	Pathogenic	-11.75	Pathogenic	0.69	Disease	1.00	Probably damaging	0.35	Tolerated	32.00	Deleterious
p.Arg112Val	VUS	-5.85	Indeterminate	0.7993	Pathogenic	-13.18	Pathogenic	0.67	Disease	1.00	Probably damaging	0.22	Tolerated		
p.Arg112Tyr	VUS	-0.95	Neutral	0.7175	Pathogenic	-13.47	Pathogenic	0.61	Disease	0.99	Probably damaging	0.04	Deleterious		
p.Arg112Cys	VUS	-16.03	Indeterminate	0.5496	Ambiguous	-10.16	Pathogenic	0.42	Neutral	1.00	Probably damaging	0.05	Deleterious	33.00	Deleterious
p.Arg112Trp	VUS	-1.48	Neutral	0.4479	Ambiguous	-12.78	Pathogenic	0.69	Disease	1.00	Probably damaging	0.02	Deleterious		
p.Arg112Phe	VUS	-4.69	Neutral	0.8447	Pathogenic	-14.05	Pathogenic	0.67	Disease	0.99	Probably damaging	0.04	Deleterious		
p.Leu113Asn	VUS	-7.95	Indeterminate	0.4174	Ambiguous	-14.49	Pathogenic	0.66	Disease	1.00	Probably damaging	0.03	Deleterious		
p.Leu113Lys	VUS	-0.38	Neutral	0.1897	Benign	-11.50	Pathogenic	0.68	Disease	0.98	Probably damaging	0.03	Deleterious		
p.Leu113Thr	VUS	-3.67	Neutral	0.211	Benign	-5.96	Benign	0.45	Neutral	1.00	Probably damaging	0.24	Tolerated		
p.Leu113Arg	VUS	-5.57	Neutral	0.1019	Benign	-5.81	Benign	0.45	Neutral	1.00	Probably damaging	0.02	Deleterious	23.30	Deleterious
p.Leu113Ser	VUS	-0.49	Neutral	0.3002	Benign	-8.80	Pathogenic	0.69	Disease	1.00	Probably damaging	0.22	Tolerated		
p.Leu113Ile	VUS	-1.77	Neutral	0.2739	Benign	-9.90	Pathogenic	0.49	Neutral	1.00	Probably damaging	0.13	Tolerated		
p.Leu113Met	VUS	-5.88	Indeterminate	0.1896	Benign	-6.39	Benign	0.49	Neutral	1.00	Probably damaging	0.22	Tolerated	26.00	Deleterious
p.Leu113His	VUS	-14.07	Indeterminate	0.2142	Benign	-10.56	Pathogenic	0.75	Disease	1.00	Probably damaging	0.01	Deleterious		
p.Leu113Gln	VUS	-1.19	Neutral	0.1148	Benign	-8.15	Pathogenic	0.47	Neutral	1.00	Probably damaging	0.03	Deleterious	26.40	Deleterious
p.Leu113Pro	VUS	-53.15	Deleterious	0.4469	Ambiguous	-11.83	Pathogenic	0.80	Disease	1.00	Probably damaging	0.03	Deleterious	28.20	Deleterious
p.Leu113Asp	VUS	-1.75	Neutral	0.6505	Pathogenic	-13.75	Pathogenic	0.70	Disease	1.00	Probably damaging	0.02	Deleterious		
p.Leu113Glu	VUS	-0.57	Neutral	0.2218	Benign	-11.71	Pathogenic	0.68	Disease	1.00	Probably damaging	0.02	Deleterious		
p.Leu113Ala	VUS	-22.19	Indeterminate	0.2436	Benign	-9.41	Pathogenic	0.65	Disease	1.00	Probably damaging	0.08	Tolerated		
p.Leu113Gly	VUS	-6.02	Indeterminate	0.4156	Ambiguous	-11.03	Pathogenic	0.69	Disease	1.00	Probably damaging	0.02	Deleterious		
p.Leu113Val	VUS	-0.13	Neutral	0.1729	Benign	-8.54	Pathogenic	0.52	Disease	0.99	Probably damaging	0.11	Tolerated	25.20	Deleterious
p.Leu113Tyr	VUS	-2.67	Neutral	0.2983	Benign	-11.35	Pathogenic	0.62	Disease	0.99	Probably damaging	0.02	Deleterious		
p.Leu113Cys	VUS	-0.85	Neutral	0.4819	Ambiguous	-9.89	Pathogenic	0.64	Disease	1.00	Probably damaging	0.02	Deleterious		
p.Leu113Trp	VUS	-3.29	Neutral	0.2657	Benign	-9.90	Pathogenic	0.69	Disease	1.00	Probably damaging	0.01	Deleterious		
p.Leu113Phe	VUS	-13.98	Indeterminate	0.1755	Benign	-9.75	Pathogenic	0.55	Disease	1.00	Probably damaging	0.07	Tolerated		
p.Pro114Asn	VUS	-53.15	Deleterious	0.995	Pathogenic	-18.60	Pathogenic	0.89	Disease	1.00	Probably damaging	0	Deleterious		
p.Pro114Lys	VUS	-53.15	Deleterious	0.9972	Pathogenic	-20.74	Pathogenic	0.92	Disease	1.00	Probably damaging	0	Deleterious		
p.Pro114Thr	Likely pathogenic	-53.15	Deleterious	0.8814	Pathogenic	-14.09	Pathogenic	0.93	Disease	1.00	Probably damaging	0	Deleterious	28.90	Deleterious
p.Pro114Arg	VUS	-53.15	Deleterious	0.973	Pathogenic	-15.01	Pathogenic	0.94	Disease	1.00	Probably damaging	0	Deleterious	31.00	Deleterious
p.Pro114Ser	VUS	-9.07	Indeterminate	0.8619	Pathogenic	-9.87	Pathogenic	0.91	Disease	1.00	Probably damaging	0	Deleterious	29.90	Deleterious
p.Pro114Ile	VUS	-53.15	Deleterious	0.9784	Pathogenic	-19.22	Pathogenic	0.88	Disease	1.00	Probably damaging	0	Deleterious		
p.Pro114Met	VUS	-53.15	Deleterious	0.9889	Pathogenic	-17.99	Pathogenic	0.91	Disease	1.00	Probably damaging	0	Deleterious		
p.Pro114His	Likely pathogenic	-53.15	Deleterious	0.9886	Pathogenic	-15.83	Pathogenic	0.91	Disease	1.00	Probably damaging	0	Deleterious	31.00	Deleterious
p.Pro114Gln	VUS	-53.15	Deleterious	0.9769	Pathogenic	-17.49	Pathogenic	0.90	Disease	1.00	Probably damaging	0	Deleterious		
p.Pro114Leu	VUS	-53.15	Deleterious	0.8805	Pathogenic	-15.28	Pathogenic	0.93	Disease	1.00	Probably damaging	0	Deleterious	32.00	Deleterious
p.Pro114Asp	VUS	-53.15	Deleterious	0.9963	Pathogenic	-18.40	Pathogenic	0.91	Disease	1.00	Probably damaging	0	Deleterious		
p.Pro114Glu	VUS	-53.15	Deleterious	0.9878	Pathogenic	-17.85	Pathogenic	0.87	Disease	1.00	Probably damaging	0	Deleterious		
p.Pro114Ala	VUS	-8.63	Indeterminate	0.4367	Ambiguous	-5.69	Benign	0.81	Disease	1.00	Probably damaging	0	Deleterious	27.90	Deleterious
p.Pro114Gly	VUS	-53.15	Deleterious	0.9382	Pathogenic	-11.20	Pathogenic	0.86	Disease	1.00	Probably damaging	0	Deleterious		
p.Pro114Val	VUS	-32.81	Indeterminate	0.9005	Pathogenic	-13.48	Pathogenic	0.85	Disease	1.00	Probably damaging	0	Deleterious		
p.Pro114Tyr	VUS	-53.15	Deleterious	0.9981	Pathogenic	-18.72	Pathogenic	0.91	Disease	1.00	Probably damaging	0	Deleterious		
p.Pro114Cys	VUS	-7.47	Indeterminate	0.9625	Pathogenic	-10.61	Pathogenic	0.90	Disease	1.00	Probably damaging	0	Deleterious		
p.Pro114Trp	VUS	-53.15	Deleterious	0.9989	Pathogenic	-17.70	Pathogenic	0.90	Disease	1.00	Probably damaging	0	Deleterious		
p.Pro114Phe	VUS	-53.15	Deleterious	0.9984	Pathogenic	-16.89	Pathogenic	0.90	Disease	1.00	Probably damaging	0	Deleterious		
p.Val115Asn	VUS	-2.41	Neutral	0.5598	Ambiguous	-12.03	Pathogenic	0.69	Disease	1.00	Probably damaging	0.01	Deleterious		
p.Val115Lys	VUS	-3.03	Neutral	0.4431	Ambiguous	-9.06	Pathogenic	0.75	Disease	1.00	Probably damaging	0.01	Deleterious		
p.Val115Thr	VUS	-1.57	Neutral	0.2098	Benign	-6.92	Benign	0.53	Disease	1.00	Probably damaging	0.03	Deleterious		
p.Val115Arg	VUS	-21.93	Indeterminate	0.2736	Benign	-6.10	Benign	0.75	Disease	1.00	Probably damaging	0.01	Deleterious		
p.Val115Ser	VUS	-4.66	Neutral	0.2381	Benign	-6.40	Benign	0.62	Disease	1.00	Probably damaging	0.01	Deleterious		
p.Val115Ile	VUS	-2.54	Neutral	0.1129	Benign	-5.62	Benign	0.11	Neutral	0.45	Benign	0.72	Tolerated		
p.Val115Met	VUS	-1.44	Neutral	0.2528	Benign	-7.13	Benign	0.44	Neutral	1.00	Probably damaging	0.05	Deleterious	26.30	Deleterious
p.Val115His	VUS	-7.13	Indeterminate	0.6119	Pathogenic	-8.48	Pathogenic	0.67	Disease	1.00	Probably damaging	0	Deleterious		
p.Val115Gln	VUS	-19.06	Indeterminate	0.3317	Benign	-5.81	Benign	0.68	Disease	1.00	Probably damaging	0.01	Deleterious		
p.Val115Pro	VUS	-33.13	Indeterminate	0.5724	Pathogenic	-9.79	Pathogenic	0.66	Disease	1.00	Probably damaging	0.01	Deleterious		
p.Val115Leu	VUS	-2.89	Neutral	0.2356	Benign	-5.26	Benign	0.11	Neutral	0.01	Benign	0.97	Tolerated	22.20	Deleterious
p.Val115Asp	VUS	-9.37	Indeterminate	0.5529	Ambiguous	-10.51	Pathogenic	0.82	Disease	1.00	Probably damaging	0.01	Deleterious		
p.Val115Glu	VUS	-9.20	Indeterminate	0.3085	Benign	-4.96	Benign	0.78	Disease	1.00	Probably damaging	0.01	Deleterious	29.60	Deleterious
p.Val115Ala	VUS	-22.86	Indeterminate	0.1017	Benign	-2.70	Benign	0.24	Neutral	1.00	Probably damaging	0.03	Deleterious	27.50	Deleterious
p.Val115Gly	VUS	-3.24	Neutral	0.1657	Benign	-5.53	Benign	0.59	Disease	1.00	Probably damaging	0.01	Deleterious	29.20	Deleterious
p.Val115Tyr	VUS	-2.24	Neutral	0.6985	Pathogenic	-11.15	Pathogenic	0.63	Disease	1.00	Probably damaging	0.25	Tolerated		
p.Val115Cys	VUS	-2.73	Neutral	0.6361	Pathogenic	-6.77	Benign	0.59	Disease	1.00	Probably damaging	0.01	Deleterious		
p.Val115Trp	VUS	-3.56	Neutral	0.7798	Pathogenic	-8.45	Pathogenic	0.59	Disease	1.00	Probably damaging	0.01	Deleterious		
p.Val115Phe	VUS	-3.04	Neutral	0.342	Ambiguous	-10.76	Pathogenic	0.65	Disease	0.99	Probably damaging	0.05	Deleterious		
p.Asp116Asn	VUS	-3.49	Neutral	0.4	Ambiguous	-10.46	Pathogenic	0.65	Disease	0.98	Probably damaging	0.59	Tolerated	32.00	Deleterious
p.Asp116Lys	VUS	-0.50	Neutral	0.8451	Pathogenic	-11.20	Pathogenic	0.74	Disease	1.00	Probably damaging	0.18	Tolerated		
p.Asp116Thr	VUS	-5.85	Indeterminate	0.6412	Pathogenic	-9.90	Pathogenic	0.70	Disease	0.96	Probably damaging	0.13	Tolerated		
p.Asp116Arg	VUS	-4.81	Neutral	0.7232	Pathogenic	-8.52	Pathogenic	0.73	Disease	1.00	Probably damaging	0.09	Tolerated		
p.Asp116Ser	VUS	-1.21	Neutral	0.4038	Ambiguous	-9.67	Pathogenic	0.64	Disease	1.00	Probably damaging	0.29	Tolerated		
p.Asp116Ile	VUS	-3.94	Neutral	0.8019	Pathogenic	-13.22	Pathogenic	0.69	Disease	0.45	Benign	0.02	Deleterious		
p.Asp116Met	VUS	-0.33	Neutral	0.8692	Pathogenic	-10.86	Pathogenic	0.70	Disease	1.00	Probably damaging	0.01	Deleterious		
p.Asp116His	VUS	-1.16	Neutral	0.3546	Ambiguous	-8.67	Pathogenic	0.30	Neutral	1.00	Probably damaging	0.49	Tolerated	25.20	Deleterious
p.Asp116Gln	VUS	-1.92	Neutral	0.5174	Ambiguous	-5.49	Benign	0.64	Disease	1.00	Probably damaging	0.14	Tolerated		
p.Asp116Pro	VUS	-53.15	Deleterious	0.9689	Pathogenic	-13.02	Pathogenic	0.70	Disease	0.92	Possibly damaging	0.11	Tolerated		
p.Asp116Leu	VUS	-2.83	Neutral	0.6897	Pathogenic	-10.07	Pathogenic	0.71	Disease	0.93	Possibly damaging	0.04	Deleterious		
p.Asp116Glu	VUS	-1.73	Neutral	0.319	Benign	-4.80	Benign	0.28	Neutral	0.79	Possibly damaging	0.32	Tolerated	24.95	Deleterious
p.Asp116Ala	VUS	-2.29	Neutral	0.4161	Ambiguous	-7.45	Benign	0.75	Disease	0.97	Probably damaging	0.14	Tolerated	32.00	Deleterious
p.Asp116Gly	VUS	-7.40	Indeterminate	0.5678	Pathogenic	-8.44	Pathogenic	0.77	Disease	1.00	Probably damaging	0.37	Tolerated	32.00	Deleterious
p.Asp116Val	VUS	-2.63	Neutral	0.5947	Pathogenic	-9.75	Pathogenic	0.77	Disease	0.63	Possibly damaging	0.03	Deleterious	32.00	Deleterious
p.Asp116Tyr	VUS	-0.83	Neutral	0.5072	Ambiguous	-12.24	Pathogenic	0.78	Disease	1.00	Probably damaging	0.06	Tolerated	32.00	Deleterious
p.Asp116Cys	VUS	-0.88	Neutral	0.8589	Pathogenic	-10.57	Pathogenic	0.69	Disease	1.00	Probably damaging	0.03	Deleterious		
p.Asp116Trp	VUS	-8.45	Indeterminate	0.9209	Pathogenic	-12.10	Pathogenic	0.70	Disease	1.00	Probably damaging	0.01	Deleterious		
p.Asp116Phe	VUS	-3.01	Neutral	0.7835	Pathogenic	-12.89	Pathogenic	0.69	Disease	0.99	Probably damaging	0.03	Deleterious		
p.Leu117Asn	VUS	-1.87	Neutral	0.9031	Pathogenic	-12.87	Pathogenic	0.70	Disease	1.00	Probably damaging	0	Deleterious		
p.Leu117Lys	VUS	-1.02	Neutral	0.7514	Pathogenic	-9.51	Pathogenic	0.74	Disease	0.99	Probably damaging	0	Deleterious		
p.Leu117Thr	VUS	-0.87	Neutral	0.6291	Pathogenic	-8.55	Pathogenic	0.72	Disease	1.00	Probably damaging	0	Deleterious		
p.Leu117Arg	VUS	-1.66	Neutral	0.4913	Ambiguous	-7.85	Pathogenic	0.87	Disease	1.00	Probably damaging	0	Deleterious	32.00	Deleterious
p.Leu117Ser	VUS	-1.67	Neutral	0.8948	Pathogenic	-9.99	Pathogenic	0.77	Disease	1.00	Probably damaging	0	Deleterious		
p.Leu117Ile	VUS	-1.14	Neutral	0.1638	Benign	-8.89	Pathogenic	0.27	Neutral	1.00	Probably damaging	0.14	Tolerated		
p.Leu117Met	VUS	-2.16	Neutral	0.3229	Benign	-7.59	Pathogenic	0.47	Neutral	1.00	Probably damaging	0.16	Tolerated	26.10	Deleterious
p.Leu117His	VUS	-0.50	Neutral	0.6986	Pathogenic	-9.81	Pathogenic	0.75	Disease	1.00	Probably damaging	0	Deleterious		
p.Leu117Gln	VUS	-3.86	Neutral	0.6392	Pathogenic	-9.14	Pathogenic	0.80	Disease	1.00	Probably damaging	0	Deleterious	31.00	Deleterious
p.Leu117Pro	VUS	-33.22	Indeterminate	0.9393	Pathogenic	-12.56	Pathogenic	0.88	Disease	1.00	Probably damaging	0	Deleterious	32.00	Deleterious
p.Leu117Asp	VUS	-0.74	Neutral	0.9488	Pathogenic	-12.70	Pathogenic	0.71	Disease	1.00	Probably damaging	0	Deleterious		
p.Leu117Glu	VUS	-1.45	Neutral	0.7894	Pathogenic	-10.29	Pathogenic	0.73	Disease	1.00	Probably damaging	0	Deleterious		

p.Leu117Ala	VUS	-3.34	Neutral	0.6184	Pathogenic	-7.30	Benign	0.64	Disease	1.00	Probably damaging	0.01	Deleterious		
p.Leu117Gly	VUS	-1.70	Neutral	0.8729	Pathogenic	-10.95	Pathogenic	0.71	Disease	1.00	Probably damaging	0	Deleterious		
p.Leu117Val	VUS	-1.09	Neutral	0.1608	Benign	-5.80	Benign	0.18	Neutral	1.00	Probably damaging	0.02	Deleterious	23.80	Deleterious
p.Leu117Tyr	VUS	-0.42	Neutral	0.7173	Pathogenic	-10.15	Pathogenic	0.63	Disease	1.00	Probably damaging	0	Deleterious		
p.Leu117Cys	VUS	-2.13	Neutral	0.8485	Pathogenic	-9.32	Pathogenic	0.65	Disease	1.00	Probably damaging	0	Deleterious		
p.Leu117Trp	VUS	-1.72	Neutral	0.6017	Pathogenic	-6.36	Benign	0.71	Disease	1.00	Probably damaging	0	Deleterious		
p.Leu117Phe	VUS	-1.86	Neutral	0.4215	Ambiguous	-9.89	Pathogenic	0.51	Disease	1.00	Probably damaging	0.02	Deleterious		
p.Ala118Asn	VUS	-32.02	Indeterminate	0.9907	Pathogenic	-18.64	Pathogenic	0.85	Disease	1.00	Probably damaging	0.01	Deleterious		
p.Ala118Lys	VUS	-33.22	Indeterminate	0.9948	Pathogenic	-19.83	Pathogenic	0.89	Disease	1.00	Probably damaging	0.01	Deleterious		
p.Ala118Thr	VUS	-4.89	Neutral	0.8464	Pathogenic	-12.28	Pathogenic	0.90	Disease	1.00	Probably damaging	0.02	Deleterious	32.00	Deleterious
p.Ala118Arg	VUS	-27.39	Indeterminate	0.9534	Pathogenic	-15.22	Pathogenic	0.87	Disease	1.00	Probably damaging	0.01	Deleterious		
p.Ala118Ser	VUS	-6.37	Indeterminate	0.418	Ambiguous	-10.49	Pathogenic	0.80	Disease	0.97	Probably damaging	0.04	Deleterious	29.80	Deleterious
p.Ala118Ile	VUS	-12.28	Indeterminate	0.9817	Pathogenic	-17.46	Pathogenic	0.89	Disease	1.00	Probably damaging	0.01	Deleterious		
p.Ala118Met	VUS	-26.26	Indeterminate	0.9763	Pathogenic	-17.51	Pathogenic	0.83	Disease	1.00	Probably damaging	0	Deleterious		
p.Ala118His	VUS	-33.22	Indeterminate	0.9896	Pathogenic	-18.95	Pathogenic	0.87	Disease	1.00	Probably damaging	0	Deleterious		
p.Ala118Gln	VUS	-29.56	Indeterminate	0.966	Pathogenic	-18.83	Pathogenic	0.89	Disease	1.00	Probably damaging	0.01	Deleterious		
p.Ala118Pro	VUS	-33.04	Indeterminate	0.92	Pathogenic	-13.05	Pathogenic	0.90	Disease	1.00	Probably damaging	0.02	Deleterious	31.00	Deleterious
p.Ala118Leu	VUS	-10.18	Indeterminate	0.8613	Pathogenic	-16.67	Pathogenic	0.85	Disease	1.00	Probably damaging	0.01	Deleterious		
p.Ala118Asp	VUS	-28.32	Indeterminate	0.98	Pathogenic	-16.04	Pathogenic	0.94	Disease	1.00	Probably damaging	0.01	Deleterious	32.00	Deleterious
p.Ala118Glu	VUS	-31.29	Indeterminate	0.9708	Pathogenic	-16.79	Pathogenic	0.91	Disease	1.00	Probably damaging	0.01	Deleterious		
p.Ala118Gly	VUS	-2.90	Neutral	0.3954	Ambiguous	-10.74	Pathogenic	0.82	Disease	1.00	Probably damaging	0.05	Deleterious	32.00	Deleterious
p.Ala118Val	VUS	-6.66	Indeterminate	0.8495	Pathogenic	-12.26	Pathogenic	0.85	Disease	1.00	Probably damaging	0.02	Deleterious	32.00	Deleterious
p.Ala118Tyr	VUS	-32.85	Indeterminate	0.9941	Pathogenic	-19.07	Pathogenic	0.89	Disease	1.00	Probably damaging	0	Deleterious		
p.Ala118Cys	VUS	-2.10	Neutral	0.8877	Pathogenic	-12.96	Pathogenic	0.80	Disease	1.00	Probably damaging	0.16	Tolerated		
p.Ala118Trp	VUS	-33.22	Indeterminate	0.9968	Pathogenic	-19.92	Pathogenic	0.90	Disease	1.00	Probably damaging	0	Deleterious		
p.Ala118Phe	VUS	-31.19	Indeterminate	0.9903	Pathogenic	-19.66	Pathogenic	0.89	Disease	1.00	Probably damaging	0	Deleterious		
p.Glu119Asn	VUS	-1.46	Neutral	0.5101	Ambiguous	-10.85	Pathogenic	0.39	Neutral	1.00	Probably damaging	0.29	Tolerated		
p.Glu119Lys	VUS	-0.30	Neutral	0.2273	Benign	-9.38	Pathogenic	0.25	Neutral	1.00	Probably damaging	0.55	Tolerated	19.51	Deleterious
p.Glu119Thr	VUS	-0.06	Neutral	0.294	Benign	-7.87	Pathogenic	0.42	Neutral	1.00	Probably damaging	0.57	Tolerated		
p.Glu119Arg	VUS	-10.02	Indeterminate	0.1837	Benign	-7.03	Benign	0.25	Neutral	1.00	Probably damaging	0.32	Tolerated		
p.Glu119Ser	VUS	0.00	Neutral	0.3272	Benign	-9.06	Pathogenic	0.43	Neutral	1.00	Probably damaging	0.45	Tolerated		
p.Glu119Ile	VUS	-0.05	Neutral	0.5279	Ambiguous	-9.42	Pathogenic	0.31	Neutral	1.00	Probably damaging	0.51	Tolerated		
p.Glu119Met	VUS	-4.89	Neutral	0.5649	Pathogenic	-9.59	Pathogenic	0.49	Neutral	1.00	Probably damaging	0.15	Tolerated		
p.Glu119His	VUS	-30.83	Indeterminate	0.453	Ambiguous	-9.34	Pathogenic	0.42	Neutral	1.00	Probably damaging	0.11	Tolerated		
p.Glu119Gln	VUS	-23.79	Indeterminate	0.1483	Benign	-6.81	Benign	0.17	Neutral	0.99	Probably damaging	0.37	Tolerated	17.96	Deleterious
p.Glu119Pro	VUS	-33.21	Indeterminate	0.945	Pathogenic	-13.45	Pathogenic	0.47	Neutral	1.00	Probably damaging	0.23	Tolerated		
p.Glu119Leu	VUS	-1.13	Neutral	0.3708	Ambiguous	-8.04	Pathogenic	0.29	Neutral	1.00	Probably damaging	0.49	Tolerated		
p.Glu119Asp	VUS	-9.98	Indeterminate	0.2843	Benign	-7.45	Benign	0.33	Neutral	0.99	Probably damaging	0.37	Tolerated	15.22	Deleterious
p.Glu119Ala	VUS	-0.01	Neutral	0.1304	Benign	-6.79	Benign	0.16	Neutral	1.00	Probably damaging	0.84	Tolerated	18.66	Deleterious
p.Glu119Gly	VUS	-0.65	Neutral	0.1783	Benign	-8.90	Pathogenic	0.25	Neutral	1.00	Probably damaging	0.22	Tolerated	22.20	Deleterious
p.Glu119Val	VUS	-5.91	Indeterminate	0.2797	Benign	-6.98	Benign	0.22	Neutral	1.00	Probably damaging	1	Tolerated	21.70	Deleterious
p.Glu119Tyr	VUS	0.00	Neutral	0.6978	Pathogenic	-12.23	Pathogenic	0.42	Neutral	1.00	Probably damaging	0.1	Tolerated		
p.Glu119Cys	VUS	0.00	Neutral	0.8272	Pathogenic	-10.09	Pathogenic	0.44	Neutral	1.00	Probably damaging	0.11	Tolerated		
p.Glu119Trp	VUS	-31.32	Indeterminate	0.8444	Pathogenic	-11.54	Pathogenic	0.49	Neutral	1.00	Probably damaging	0.03	Deleterious		
p.Glu119Phe	VUS	-33.21	Indeterminate	0.733	Pathogenic	-12.37	Pathogenic	0.43	Neutral	1.00	Probably damaging	0.12	Tolerated		
p.Glu120Asn	VUS	-1.82	Neutral	0.6455	Pathogenic	-8.71	Pathogenic	0.46	Neutral	1.00	Probably damaging	0.2	Tolerated		
p.Glu120Lys	VUS	-1.90	Neutral	0.5939	Pathogenic	-7.47	Benign	0.53	Disease	0.79	Possibly damaging	0.54	Tolerated	24.20	Deleterious
p.Glu120Thr	VUS	-1.40	Neutral	0.4767	Ambiguous	-6.27	Benign	0.50	Neutral	1.00	Probably damaging	0.24	Tolerated		
p.Glu120Arg	VUS	-5.70	Neutral	0.4604	Ambiguous	-6.28	Benign	0.46	Neutral	0.91	Possibly damaging	0.3	Tolerated		
p.Glu120Ser	VUS	-1.67	Neutral	0.4157	Ambiguous	-5.36	Benign	0.46	Neutral	0.99	Probably damaging	0.3	Tolerated		
p.Glu120Ile	VUS	-5.03	Neutral	0.6566	Pathogenic	-7.99	Pathogenic	0.49	Neutral	0.99	Probably damaging	0.19	Tolerated		
p.Glu120Met	VUS	-12.94	Indeterminate	0.7051	Pathogenic	-6.86	Benign	0.55	Disease	0.99	Probably damaging	0.04	Deleterious		
p.Glu120His	VUS	-2.96	Neutral	0.6129	Pathogenic	-6.68	Benign	0.46	Neutral	0.98	Probably damaging	0.06	Tolerated		
p.Glu120Gln	VUS	-4.15	Neutral	0.2187	Benign	-3.82	Benign	0.28	Neutral	0.17	Benign	0.31	Tolerated	21.90	Deleterious
p.Glu120Pro	VUS	-4.57	Neutral	0.9068	Pathogenic	-10.42	Pathogenic	0.56	Disease	1.00	Probably damaging	0.12	Tolerated		
p.Glu120Leu	VUS	-1.27	Neutral	0.6466	Pathogenic	-6.07	Benign	0.50	Neutral	0.94	Possibly damaging	0.13	Tolerated		
p.Glu120Asp	VUS	-2.28	Neutral	0.2992	Benign	-5.91	Benign	0.26	Neutral	0.75	Possibly damaging	0.45	Tolerated	21.85	Deleterious
p.Glu120Ala	VUS	-0.79	Neutral	0.2356	Benign	-4.11	Benign	0.30	Neutral	0.96	Possibly damaging	0.32	Tolerated	22.90	Deleterious
p.Glu120Gly	VUS	-1.95	Neutral	0.3373	Benign	-6.30	Benign	0.46	Neutral	1.00	Probably damaging	0.16	Tolerated	25.20	Deleterious
p.Glu120Val	VUS	-1.61	Neutral	0.482	Ambiguous	-6.19	Benign	0.50	Disease	0.95	Possibly damaging	0.12	Tolerated	26.50	Deleterious
p.Glu120Tyr	VUS	-11.75	Indeterminate	0.7664	Pathogenic	-8.90	Pathogenic	0.49	Neutral	1.00	Probably damaging	0.02	Deleterious		
p.Glu120Cys	VUS	-1.14	Neutral	0.9161	Pathogenic	-8.29	Pathogenic	0.54	Disease	1.00	Probably damaging	0.02	Deleterious		
p.Glu120Trp	VUS	-2.61	Neutral	0.9213	Pathogenic	-10.28	Pathogenic	0.43	Neutral	1.00	Probably damaging	0.01	Deleterious		
p.Glu120Phe	VUS	-0.12	Neutral	0.8696	Pathogenic	-9.00	Pathogenic	0.52	Disease	1.00	Probably damaging	0.02	Deleterious		
p.Leu121Asn	VUS	-4.57	Neutral	0.2029	Benign	0.79	Benign	0.18	Neutral	0.05	Benign	0.38	Tolerated		
p.Leu121Lys	VUS	-1.77	Neutral	0.147	Benign	-2.38	Benign	0.20	Neutral	0.00	Benign	0.79	Tolerated		
p.Leu121Thr	VUS	-1.63	Neutral	0.1938	Benign	-4.38	Benign	0.24	Neutral	0.01	Benign	0.47	Tolerated		
p.Leu121Arg	VUS	-12.00	Indeterminate	0.0651	Benign	1.68	Benign	0.11	Neutral	0.00	Benign	0.85	Tolerated	11.52	Neutral
p.Leu121Ser	VUS	-2.80	Neutral	0.2532	Benign	-0.34	Benign	0.09	Neutral	0.02	Benign	0.48	Tolerated		
p.Leu121Ile	VUS	-1.47	Neutral	0.19	Benign	-8.56	Pathogenic	0.18	Neutral	0.00	Benign	0.4	Tolerated		
p.Leu121Met	VUS	-53.15	Deleterious	0.1454	Benign	-5.63	Benign	0.22	Neutral	0.00	Benign	0.23	Tolerated	21.30	Deleterious
p.Leu121His	VUS	-0.28	Neutral	0.1246	Benign	0.35	Benign	0.15	Neutral	0.18	Benign	0.64	Tolerated		
p.Leu121Gln	VUS	-1.06	Neutral	0.0787	Benign	-1.63	Benign	0.11	Neutral	0.00	Benign	0.55	Tolerated	13.36	Neutral
p.Leu121Pro	VUS	-0.56	Neutral	0.7069	Pathogenic	-7.54	Pathogenic	0.23	Neutral	0.07	Benign	0.26	Tolerated	14.80	Neutral
p.Leu121Asp	VUS	0.00	Neutral	0.4718	Ambiguous	-5.15	Benign	0.29	Neutral	0.05	Benign	0.27	Tolerated		
p.Leu121Glu	VUS	-3.77	Neutral	0.1833	Benign	-2.75	Benign	0.18	Neutral	0.01	Benign	0.76	Tolerated		
p.Leu121Ala	VUS	-2.68	Neutral	0.1338	Benign	-0.09	Benign	0.22	Neutral	0.01	Benign	0.59	Tolerated		
p.Leu121Gly	VUS	-3.55	Neutral	0.168	Benign	0.18	Benign	0.15	Neutral	0.02	Benign	0.38	Tolerated		
p.Leu121Val	VUS	-0.04	Neutral	0.1629	Benign	-5.47	Benign	0.14	Neutral	0.00	Benign	0.51	Tolerated	17.90	Deleterious
p.Leu121Tyr	VUS	-0.11	Neutral	0.2306	Benign	-1.88	Benign	0.24	Neutral	0.00	Benign	1	Tolerated		
p.Leu121Cys	VUS	-0.25	Neutral	0.2808	Benign	-0.78	Benign	0.25	Neutral	0.56	Possibly damaging	0.17	Tolerated		
p.Leu121Trp	VUS	-3.11	Neutral	0.1596	Benign	-5.17	Benign	0.26	Neutral	0.26	Benign	0.18	Tolerated		
p.Leu121Phe	VUS	-0.56	Neutral	0.1576	Benign	-2.40	Benign	0.12	Neutral	0.00	Benign	0.65	Tolerated		
p.Gly122Asn	VUS	-0.06	Neutral	0.6676	Pathogenic	-9.46	Pathogenic	0.41	Neutral	1.00	Probably damaging	0.27	Tolerated		
p.Gly122Lys	VUS	-0.45	Neutral	0.8542	Pathogenic	-8.95	Pathogenic	0.64	Disease	1.00	Probably damaging	0.05	Deleterious		
p.Gly122Thr	VUS	-0.11	Neutral	0.7899	Pathogenic	-9.54	Pathogenic	0.43	Neutral	1.00	Probably damaging	0.04	Deleterious		
p.Gly122Arg	VUS	-2.36	Neutral	0.6425	Pathogenic	-6.20	Benign	0.66	Disease	1.00	Probably damaging	0.03	Deleterious	25.80	Deleterious
p.Gly122Ser	VUS	-0.02	Neutral	0.4055	Ambiguous	-8.12	Pathogenic	0.55	Disease	1.00	Probably damaging	0.1	Tolerated	25.80	Deleterious
p.Gly122Ile	VUS	-3.72	Neutral	0.9444	Pathogenic	-12.62	Pathogenic	0.68	Disease	1.00	Probably damaging	0.01	Deleterious		
p.Gly122Met	VUS	-0.24	Neutral	0.9514	Pathogenic	-6.51	Benign	0.68	Disease	1.00	Probably damaging	0	Deleterious		
p.Gly122His	VUS	-0.06	Neutral	0.7213	Pathogenic	-7.17	Benign	0.62	Disease	1.00	Probably damaging	0.03	Deleterious		
p.Gly122Gln	VUS	-0.10	Neutral	0.6852	Pathogenic	-6.15	Benign	0.59	Disease	1.00	Probably damaging	0.03	Deleterious		
p.Gly122Pro	VUS	-1.75	Neutral	0.972	Pathogenic	-10.91	Pathogenic	0.43	Neutral	1.00	Probably damaging	0.19	Tolerated		
p.Gly122Leu	VUS	-0.96	Neutral	0.8733	Pathogenic	-4.19	Benign	0.66	Disease	1.00	Probably damaging	0.01	Deleterious		
p.Gly122Asp	VUS	-0.29	Neutral	0.4509	Ambiguous	-8.31	Pathogenic	0.66	Disease	1.00	Probably damaging	0.14	Tolerated	25.50	Deleterious
p.Gly122Glu	VUS	-0.41	Neutral	0.6464	Pathogenic	-7.32	Benign	0.67	Disease	1.00	Probably damaging	0.05	Deleterious		
p.Gly122Ala	VUS	-0.34	Neutral	0.5326	Ambiguous	-6.70	Benign	0.48	Neutral	1.00	Probably damaging	0.08	Tolerated	24.70	Deleterious
p.Gly122Val	VUS	-0.08	Neutral	0.8833	Pathogenic	-9.66	Pathogenic	0.74	Disease	1.00	Probably damaging	0.01	Deleterious	25.30	Deleterious
p.Gly122Tyr	VUS	-0.60	Neutral	0.8741	Pathogenic	-10.48	Pathogenic	0.66	Disease	1.00	Probably damaging	0.02	Deleterious		
p.Gly122Cys	VUS	-4.58	Neutral	0.6653	Pathogenic	-7.96	Pathogenic	0.68	Disease	1.00	Probably damaging	0.01	Deleterious	26.40	Deleterious
p.Gly122Trp	VUS	-5.31	Neutral	0.8381	Pathogenic	-9.89	Pathogenic	0.72	Disease	1.00	Probably damaging	0	Deleterious		
p.Gly122Phe	VUS	-1.39	Neutral	0.9486	Pathogenic	-9.80	Pathogenic	0.69	Disease	1.00	Probably damaging	0.01	Deleterious		
p.His123Asn	VUS	-6.00	Indeterminate	0.4955	Ambiguous	-9.45	Pathogenic	0.64	Disease	1.00	Probably damaging	0.12	Tolerated	25.50	Deleterious
p.His123Lys	VUS	-0.43	Neutral	0.7284	Pathogenic	-11.00	Pathogenic	0.65	Disease	1.00	Probably damaging	0.1	Tolerated		
p.His123Thr	VUS	-26.63	Indeterminate	0.9262	Pathogenic	-14.31	Pathogenic	0.67	Disease	1.00	Probably damaging	0.37	Tolerated		
p.His123Arg	VUS	-3.23	Neutral	0.3271	Benign	-7.70	Pathogenic	0.65	Disease	0.99	Probably damaging	0.07	Tolerated	24.20	Deleterious
p.His123Ser	VUS	-4.18	Neutral	0.7649	Pathogenic	-11.43	Pathogenic	0.68	Disease	1.00	Probably damaging	0.17	Tolerated		
p.His123Ile	VUS	-5.85	Indeterminate	0.9083	Pathogenic	-18.43	Pathogenic	0.73	Disease	1.00	Probably damaging	0.03	Deleterious		
p.His123Met	VUS	-13.15	Indeterminate	0.8867	Pathogenic	-12.84	Pathogenic	0.72	Disease	1.00	Probably damaging	0.02	Deleterious		
p.His123Gln	Benign	-1.24	Neutral	0.56	Ambiguous	-6.66	Benign	0.44	Neutral	1.00	Probably damaging	0.05	Deleterious	21.45	Deleterious
p.His123Pro	VUS	-53.15	Deleterious	0.8604	Pathogenic	-14.98	Pathogenic	0.82	Disease	1.00	Probably damaging	0.05	Deleterious	25.00	Deleterious

p.His123Leu	VUS	-8.40	Indeterminate	0.5414	Ambiguous	-11.64	Pathogenic	0.77	Disease	1.00	Probably damaging	0.04	Deleterious	25.20	Deleterious
p.His123Asp	VUS	-19.23	Indeterminate	0.8124	Pathogenic	-12.97	Pathogenic	0.75	Disease	1.00	Probably damaging	0.1	Tolerated	25.60	Deleterious
p.His123Glu	VUS	-16.55	Indeterminate	0.8202	Pathogenic	-11.12	Pathogenic	0.64	Disease	1.00	Probably damaging	0.06	Tolerated		
p.His123Ala	VUS	-5.96	Indeterminate	0.8083	Pathogenic	-11.49	Pathogenic	0.69	Disease	1.00	Probably damaging	0.09	Tolerated		
p.His123Gly	VUS	-53.15	Deleterious	0.8408	Pathogenic	-12.93	Pathogenic	0.71	Disease	1.00	Probably damaging	0.09	Tolerated		
p.His123Val	VUS	-27.77	Indeterminate	0.8496	Pathogenic	-15.85	Pathogenic	0.72	Disease	1.00	Probably damaging	0.05	Deleterious		
p.His123Tyr	VUS	-0.91	Neutral	0.2345	Benign	-9.44	Pathogenic	0.69	Disease	1.00	Probably damaging	0.03	Deleterious	25.50	Deleterious
p.His123Cys	VUS	-1.67	Neutral	0.4191	Ambiguous	-11.97	Pathogenic	0.46	Neutral	1.00	Probably damaging	0.02	Deleterious		
p.His123Trp	VUS	-0.86	Neutral	0.5355	Ambiguous	-10.89	Pathogenic	0.71	Disease	1.00	Probably damaging	0.01	Deleterious		
p.His123Phe	VUS	-24.07	Indeterminate	0.5384	Ambiguous	-10.33	Pathogenic	0.71	Disease	1.00	Probably damaging	0.02	Deleterious		
p.Arg124Asn	VUS	-0.04	Neutral	0.3597	Ambiguous	-8.79	Pathogenic	0.39	Neutral	0.82	Possibly damaging	0.5	Tolerated		
p.Arg124Lys	VUS	-0.74	Neutral	0.1647	Benign	-6.83	Benign	0.23	Neutral	0.18	Benign	1	Tolerated		
p.Arg124Thr	VUS	-8.50	Indeterminate	0.1369	Benign	-7.10	Benign	0.21	Neutral	0.82	Possibly damaging	0.59	Tolerated		
p.Arg124Ser	VUS	-0.05	Neutral	0.211	Benign	-7.20	Benign	0.18	Neutral	0.97	Probably damaging	0.72	Tolerated	6.28	Neutral
p.Arg124Ile	VUS	-32.97	Indeterminate	0.2337	Benign	-10.01	Pathogenic	0.38	Neutral	0.99	Probably damaging	0.24	Tolerated		
p.Arg124Met	VUS	-0.21	Neutral	0.3405	Ambiguous	-9.58	Pathogenic	0.37	Neutral	0.94	Possibly damaging	0.14	Tolerated		
p.Arg124His	VUS	-0.31	Neutral	0.1059	Benign	-6.32	Benign	0.10	Neutral	0.01	Benign	0.23	Tolerated	11.48	Neutral
p.Arg124Gln	VUS	-0.20	Neutral	0.1057	Benign	-6.73	Benign	0.12	Neutral	0.87	Possibly damaging	0.61	Tolerated		
p.Arg124Pro	VUS	-0.31	Neutral	0.1531	Benign	-8.06	Pathogenic	0.42	Neutral	1.00	Probably damaging	0.29	Tolerated	16.31	Deleterious
p.Arg124Leu	VUS	-0.46	Neutral	0.1291	Benign	-7.80	Pathogenic	0.16	Neutral	0.96	Possibly damaging	0.41	Tolerated	10.34	Neutral
p.Arg124Asp	VUS	-0.04	Neutral	0.2316	Benign	-6.81	Benign	0.29	Neutral	0.98	Probably damaging	0.58	Tolerated		
p.Arg124Glu	VUS	-0.26	Neutral	0.1569	Benign	-6.75	Benign	0.36	Neutral	0.58	Possibly damaging	0.94	Tolerated		
p.Arg124Ala	VUS	-0.23	Neutral	0.118	Benign	-6.06	Benign	0.27	Neutral	0.48	Possibly damaging	0.76	Tolerated		
p.Arg124Gly	VUS	-11.05	Indeterminate	0.0959	Benign	-6.10	Benign	0.11	Neutral	0.05	Benign	0.4	Tolerated	6.52	Neutral
p.Arg124Val	VUS	-1.45	Neutral	0.1826	Benign	-7.60	Pathogenic	0.27	Neutral	0.89	Possibly damaging	0.35	Tolerated		
p.Arg124Tyr	VUS	-15.60	Indeterminate	0.3596	Ambiguous	-10.13	Pathogenic	0.36	Neutral	0.31	Benign	0.23	Tolerated		
p.Arg124Cys	VUS	-1.37	Neutral	0.2354	Benign	-9.11	Pathogenic	0.17	Neutral	0.63	Possibly damaging	0.08	Tolerated	12.16	Neutral
p.Arg124Trp	VUS	-0.21	Neutral	0.1848	Benign	-8.64	Pathogenic	0.19	Neutral	0.98	Probably damaging	0.05	Deleterious		
p.Arg124Phe	VUS	-0.13	Neutral	0.3872	Ambiguous	-10.36	Pathogenic	0.40	Neutral	0.47	Possibly damaging	0.18	Tolerated		
p.Asp125Asn	VUS	-0.69	Neutral	0.238	Benign	-6.08	Benign	0.41	Neutral	0.11	Benign	0.27	Tolerated	15.76	Deleterious
p.Asp125Lys	VUS	-0.03	Neutral	0.4591	Ambiguous	-8.56	Pathogenic	0.47	Neutral	0.19	Benign	0.47	Tolerated		
p.Asp125Thr	VUS	0.00	Neutral	0.3727	Ambiguous	-6.64	Benign	0.45	Neutral	0.06	Benign	0.31	Tolerated		
p.Asp125Arg	VUS	-0.24	Neutral	0.3515	Ambiguous	-6.43	Benign	0.45	Neutral	0.62	Possibly damaging	0.35	Tolerated		
p.Asp125Ser	VUS	-0.29	Neutral	0.2393	Benign	-5.78	Benign	0.39	Neutral	0.27	Benign	0.49	Tolerated		
p.Asp125Ile	VUS	0.00	Neutral	0.6176	Pathogenic	-10.50	Pathogenic	0.50	Neutral	0.77	Possibly damaging	0.1	Tolerated		
p.Asp125Met	VUS	-0.01	Neutral	0.8052	Pathogenic	-9.40	Pathogenic	0.52	Disease	0.94	Possibly damaging	0.06	Tolerated		
p.Asp125His	VUS	-0.08	Neutral	0.3071	Benign	-4.75	Benign	0.27	Neutral	0.00	Benign	0.08	Tolerated	11.51	Neutral
p.Asp125Gln	VUS	0.00	Neutral	0.2999	Benign	-5.99	Benign	0.26	Neutral	0.24	Benign	0.3	Tolerated		
p.Asp125Pro	VUS	-0.63	Neutral	0.4534	Ambiguous	-6.85	Benign	0.30	Neutral	0.06	Benign	0.23	Tolerated		
p.Asp125Leu	VUS	-0.52	Neutral	0.5063	Ambiguous	-7.72	Pathogenic	0.30	Neutral	0.62	Possibly damaging	0.22	Tolerated		
p.Asp125Glu	VUS	-0.31	Neutral	0.1526	Benign	-4.16	Benign	0.08	Neutral	0.00	Benign	0.53	Tolerated	3.76	Neutral
p.Asp125Ala	VUS	-3.17	Neutral	0.1713	Benign	-3.78	Benign	0.17	Neutral	0.01	Benign	0.47	Tolerated	14.59	Neutral
p.Asp125Gly	VUS	-1.02	Neutral	0.2285	Benign	-5.41	Benign	0.41	Neutral	0.34	Benign	0.22	Tolerated	18.70	Deleterious
p.Asp125Val	VUS	0.00	Neutral	0.354	Ambiguous	-7.27	Benign	0.32	Neutral	0.06	Benign	0.16	Tolerated	18.83	Deleterious
p.Asp125Tyr	VUS	-53.15	Deleterious	0.4002	Ambiguous	-10.54	Pathogenic	0.54	Disease	0.62	Possibly damaging	0.04	Deleterious	19.73	Deleterious
p.Asp125Cys	VUS	-0.23	Neutral	0.8053	Pathogenic	-8.69	Pathogenic	0.51	Disease	1.00	Probably damaging	0.03	Deleterious		
p.Asp125Trp	VUS	-0.08	Neutral	0.8612	Pathogenic	-10.59	Pathogenic	0.51	Disease	0.98	Probably damaging	0.01	Deleterious		
p.Asp125Phe	VUS	-0.75	Neutral	0.7897	Pathogenic	-11.42	Pathogenic	0.52	Disease	0.62	Possibly damaging	0.04	Deleterious		
p.Val126Asn	VUS	-0.47	Neutral	0.9138	Pathogenic	-15.68	Pathogenic	0.71	Disease	1.00	Probably damaging	0	Deleterious		
p.Val126Lys	VUS	-53.15	Deleterious	0.9309	Pathogenic	-16.52	Pathogenic	0.78	Disease	1.00	Probably damaging	0	Deleterious		
p.Val126Thr	VUS	0.00	Neutral	0.4014	Ambiguous	-8.18	Pathogenic	0.60	Disease	1.00	Probably damaging	0.01	Deleterious		
p.Val126Arg	VUS	-53.15	Deleterious	0.8469	Pathogenic	-14.45	Pathogenic	0.78	Disease	1.00	Probably damaging	0	Deleterious		
p.Val126Ser	VUS	0.00	Neutral	0.7069	Pathogenic	-11.39	Pathogenic	0.71	Disease	1.00	Probably damaging	0	Deleterious		
p.Val126Ile	VUS	0.00	Neutral	0.1435	Benign	-4.94	Benign	0.24	Neutral	0.02	Benign	0.18	Tolerated	14.41	Neutral
p.Val126Met	VUS	0.00	Neutral	0.6606	Pathogenic	-9.28	Pathogenic	0.58	Disease	1.00	Probably damaging	0.01	Deleterious		
p.Val126His	VUS	-33.22	Indeterminate	0.9725	Pathogenic	-14.49	Pathogenic	0.72	Disease	1.00	Probably damaging	0	Deleterious		
p.Val126Gln	VUS	-1.39	Neutral	0.9026	Pathogenic	-15.09	Pathogenic	0.75	Disease	1.00	Probably damaging	0	Deleterious		
p.Val126Pro	VUS	0.00	Neutral	0.8918	Pathogenic	-11.16	Pathogenic	0.73	Disease	1.00	Probably damaging	0	Deleterious		
p.Val126Leu	VUS	0.00	Neutral	0.6571	Pathogenic	-6.58	Benign	0.55	Disease	0.38	Benign	0.03	Deleterious	18.01	Deleterious
p.Val126Asp	Pathogenic	-53.15	Deleterious	0.9359	Pathogenic	-15.06	Pathogenic	0.85	Disease	1.00	Probably damaging	0	Deleterious	24.20	Deleterious
p.Val126Glu	VUS	-0.02	Neutral	0.876	Pathogenic	-13.79	Pathogenic	0.89	Disease	1.00	Probably damaging	0	Deleterious		
p.Val126Ala	VUS	-0.01	Neutral	0.3085	Benign	-6.81	Benign	0.60	Disease	1.00	Probably damaging	0.01	Deleterious	23.60	Deleterious
p.Val126Gly	VUS	-0.41	Neutral	0.627	Pathogenic	-12.00	Pathogenic	0.69	Disease	1.00	Probably damaging	0	Deleterious	23.80	Deleterious
p.Val126Tyr	VUS	-53.15	Deleterious	0.982	Pathogenic	-16.16	Pathogenic	0.71	Disease	1.00	Probably damaging	0	Deleterious		
p.Val126Cys	VUS	0.00	Neutral	0.9051	Pathogenic	-8.45	Pathogenic	0.67	Disease	1.00	Probably damaging	0	Deleterious		
p.Val126Trp	VUS	-53.15	Deleterious	0.9938	Pathogenic	-15.53	Pathogenic	0.68	Disease	1.00	Probably damaging	0	Deleterious		
p.Val126Phe	VUS	-0.42	Neutral	0.8624	Pathogenic	-13.84	Pathogenic	0.78	Disease	0.99	Probably damaging	0	Deleterious	22.60	Deleterious
p.Ala127Asn	VUS	-1.01	Neutral	0.6811	Pathogenic	-13.00	Pathogenic	0.62	Disease	0.97	Probably damaging	0.01	Deleterious		
p.Ala127Lys	VUS	-6.63	Indeterminate	0.7578	Pathogenic	-13.09	Pathogenic	0.67	Disease	1.00	Probably damaging	0.02	Deleterious		
p.Ala127Thr	VUS	-24.84	Indeterminate	0.1629	Benign	-7.48	Benign	0.39	Neutral	0.97	Probably damaging	0.08	Tolerated	22.80	Deleterious
p.Ala127Arg	VUS	-19.93	Indeterminate	0.501	Ambiguous	-10.02	Pathogenic	0.51	Disease	1.00	Probably damaging	0.01	Deleterious		
p.Ala127Ser	Likely benign	-2.10	Neutral	0.1514	Benign	-6.46	Benign	0.33	Neutral	0.77	Possibly damaging	0.02	Deleterious	19.18	Deleterious
p.Ala127Ile	VUS	-19.64	Indeterminate	0.384	Ambiguous	-9.36	Pathogenic	0.51	Disease	0.96	Possibly damaging	0.41	Tolerated		
p.Ala127Met	VUS	-0.87	Neutral	0.4705	Ambiguous	-9.42	Pathogenic	0.53	Disease	1.00	Probably damaging	0.03	Deleterious		
p.Ala127His	VUS	-26.04	Indeterminate	0.7222	Pathogenic	-11.09	Pathogenic	0.61	Disease	1.00	Probably damaging	0	Deleterious		
p.Ala127Gln	VUS	-2.66	Neutral	0.4734	Ambiguous	-9.09	Pathogenic	0.62	Disease	1.00	Probably damaging	0.01	Deleterious		
p.Ala127Pro	VUS	-53.15	Deleterious	0.729	Pathogenic	-8.93	Pathogenic	0.72	Disease	1.00	Probably damaging	0.02	Deleterious	23.10	Deleterious
p.Ala127Leu	VUS	-3.30	Neutral	0.2703	Benign	-6.02	Benign	0.36	Neutral	0.96	Possibly damaging	0.12	Tolerated		
p.Ala127Asp	VUS	-13.68	Indeterminate	0.6679	Pathogenic	-12.66	Pathogenic	0.72	Disease	1.00	Probably damaging	0.01	Deleterious		
p.Ala127Glu	VUS	-0.78	Neutral	0.4097	Ambiguous	-9.59	Pathogenic	0.51	Disease	1.00	Probably damaging	0.02	Deleterious	19.09	Deleterious
p.Ala127Gly	VUS	-7.66	Indeterminate	0.1776	Benign	-7.74	Pathogenic	0.34	Neutral	0.99	Probably damaging	0	Deleterious	12.32	Neutral
p.Ala127Val	VUS	-0.26	Neutral	0.1436	Benign	-4.38	Benign	0.16	Neutral	0.14	Benign	1	Tolerated	6.92	Neutral
p.Ala127Tyr	VUS	-6.56	Indeterminate	0.7619	Pathogenic	-12.16	Pathogenic	0.60	Disease	1.00	Probably damaging	0.01	Deleterious		
p.Ala127Cys	VUS	-0.09	Neutral	0.5407	Ambiguous	-8.02	Pathogenic	0.49	Neutral	1.00	Probably damaging	0.02	Deleterious		
p.Ala127Trp	VUS	-5.00	Neutral	0.8559	Pathogenic	-11.20	Pathogenic	0.59	Disease	1.00	Probably damaging	0	Deleterious		
p.Ala127Phe	VUS	-3.71	Neutral	0.6267	Pathogenic	-10.86	Pathogenic	0.54	Disease	1.00	Probably damaging	0.02	Deleterious		
p.Arg128Asn	VUS	0.00	Neutral	0.3288	Benign	-6.64	Benign	0.23	Neutral	0.79	Possibly damaging	0.24	Tolerated		
p.Arg128Lys	VUS	-2.71	Neutral	0.2077	Benign	-6.72	Benign	0.12	Neutral	0.08	Benign	0.44	Tolerated		
p.Arg128Thr	VUS	-0.32	Neutral	0.1483	Benign	-6.10	Benign	0.29	Neutral	0.79	Possibly damaging	0.27	Tolerated		
p.Arg128Ser	VUS	-19.13	Indeterminate	0.1872	Benign	-4.93	Benign	0.14	Neutral	0.76	Possibly damaging	0.53	Tolerated		
p.Arg128Ile	VUS	-8.33	Indeterminate	0.2361	Benign	-8.69	Pathogenic	0.36	Neutral	0.90	Possibly damaging	0.08	Tolerated		
p.Arg128Met	VUS	-1.08	Neutral	0.3178	Benign	-8.05	Pathogenic	0.37	Neutral	0.68	Possibly damaging	0.05	Deleterious		
p.Arg128His	VUS	-3.37	Neutral	0.1143	Benign	-5.66	Benign	0.14	Neutral	0.81	Possibly damaging	0.07	Tolerated		
p.Arg128Gln	VUS	-0.11	Neutral	0.118	Benign	-5.62	Benign	0.12	Neutral	0.13	Benign	0.28	Tolerated	17.20	Deleterious
p.Arg128Pro	VUS	-53.15	Deleterious	0.6461	Pathogenic	-10.17	Pathogenic	0.40	Neutral	1.00	Probably damaging	0.15	Tolerated	19.02	Deleterious
p.Arg128Leu	VUS	-1.25	Neutral	0.1061	Benign	-5.79	Benign	0.14	Neutral	0.04	Benign	0.14	Tolerated	18.39	Deleterious
p.Arg128Asp	VUS	-0.12	Neutral	0.2566	Benign	-5.30	Benign	0.21	Neutral	0.94	Possibly damaging	0.31	Tolerated		
p.Arg128Glu	VUS	-0.71	Neutral	0.2037	Benign	-5.06	Benign	0.23	Neutral	0.19	Benign	1	Tolerated		
p.Arg128Ala	VUS	-0.48	Neutral	0.1075	Benign	-3.42	Benign	0.23	Neutral	0.25	Benign	0.5	Tolerated		
p.Arg128Gly	VUS	-6.37	Indeterminate	0.0979	Benign	-4.91	Benign	0.12	Neutral	0.01	Benign	0.31	Tolerated	10.62	Neutral
p.Arg128Val	VUS	-0.07	Neutral	0.1998	Benign	-6.73	Benign	0.38	Neutral	0.15	Benign	0.14	Tolerated		
p.Arg128Tyr	VUS	-0.76	Neutral	0.3589	Ambiguous	-7.14	Benign	0.36	Neutral	0.69	Possibly damaging	0.03	Deleterious		
p.Arg128Cys	VUS	0.00	Neutral	0.1985	Benign	-7.01	Benign	0.33	Neutral	1.00	Probably damaging	0.04	Deleterious		
p.Arg128Trp	VUS	-0.10	Neutral	0.1984	Benign	-7.82	Pathogenic	0.17	Neutral	0.99	Probably damaging	0.01	Deleterious	21.80	Deleterious
p.Arg128Phe	VUS	-6.69	Indeterminate	0.3814	Ambiguous	-6.18	Benign	0.40	Neutral	0.26	Benign	0.03	Deleterious		
p.Tyr129Asn	VUS	-33.22	Indeterminate	0.9556	Pathogenic	-15.00	Pathogenic	0.66	Disease	1.00	Probably damaging	0.09	Tolerated	32.00	Deleterious
p.Tyr129Lys	VUS	-3.97	Neutral	0.9891	Pathogenic	-15.47	Pathogenic	0.61	Disease	1.00	Probably damaging	0.09	Tolerated		
p.Tyr129Thr	VUS	-27.35	Indeterminate	0.9689	Pathogenic	-12.26	Pathogenic	0.59	Disease	1.00	Probably damaging	0.27	Tolerated		
p.Tyr129Arg	VUS	-0.71	Neutral	0.9606	Pathogenic	-11.05	Pathogenic	0.57	Disease	1.00	Probably damaging	0.1	Tolerated		
p.Tyr129Ser	VUS	-2.79	Neutral	0.9417	Pathogenic	-10.96	Pathogenic	0.62	Disease	1.00	Probably damaging	0.14	Tolerated	30.00	Deleterious
p.Tyr129Ile	VUS	-4.50	Neutral	0.9257	Pathogenic	-11.98	Pathogenic	0.56	Disease	0.98	Probably damaging	0.15	Tolerated		

p.Tyr129Met	VUS	-0.78	Neutral	0.9612	Pathogenic	-12.23	Pathogenic	0.53	Disease	1.00	Probably damaging	0.08	Tolerated		
p.Tyr129His	VUS	-1.08	Neutral	0.8201	Pathogenic	-7.47	Benign	0.53	Disease	1.00	Probably damaging	0.16	Tolerated	28.00	Deleterious
p.Tyr129Gln	VUS	-0.73	Neutral	0.9784	Pathogenic	-11.98	Pathogenic	0.54	Disease	1.00	Probably damaging	0.09	Tolerated		
p.Tyr129Pro	VUS	-53.15	Deleterious	0.9962	Pathogenic	-14.76	Pathogenic	0.59	Disease	1.00	Probably damaging	0.07	Tolerated		
p.Tyr129Leu	VUS	-0.36	Neutral	0.8573	Pathogenic	-9.20	Pathogenic	0.52	Disease	0.94	Possibly damaging	0.28	Tolerated		
p.Tyr129Asp	VUS	-16.50	Indeterminate	0.9656	Pathogenic	-13.41	Pathogenic	0.64	Disease	1.00	Probably damaging	0.06	Tolerated	32.00	Deleterious
p.Tyr129Glu	VUS	-0.57	Neutral	0.9774	Pathogenic	-12.49	Pathogenic	0.60	Disease	1.00	Probably damaging	0.08	Tolerated		
p.Tyr129Ala	VUS	-0.56	Neutral	0.9607	Pathogenic	-10.40	Pathogenic	0.39	Neutral	1.00	Probably damaging	0.17	Tolerated		
p.Tyr129Gly	VUS	-3.93	Neutral	0.9617	Pathogenic	-14.19	Pathogenic	0.56	Disease	1.00	Probably damaging	0.1	Tolerated		
p.Tyr129Val	VUS	-0.90	Neutral	0.8873	Pathogenic	-9.78	Pathogenic	0.57	Disease	1.00	Probably damaging	0.18	Tolerated		
p.Tyr129Cys	VUS	-0.95	Neutral	0.8807	Pathogenic	-9.02	Pathogenic	0.41	Neutral	1.00	Probably damaging	0.15	Tolerated	27.90	Deleterious
p.Tyr129Trp	VUS	-0.17	Neutral	0.7312	Pathogenic	-7.42	Benign	0.52	Disease	1.00	Probably damaging	0.14	Tolerated		
p.Tyr129Phe	VUS	-1.69	Neutral	0.2033	Benign	-2.91	Benign	0.24	Neutral	0.18	Benign	0.96	Tolerated	22.70	Deleterious
p.Leu130Asn	VUS	-29.02	Indeterminate	0.9902	Pathogenic	-16.37	Pathogenic	0.76	Disease	1.00	Probably damaging	0.01	Deleterious		
p.Leu130Lys	VUS	-53.15	Deleterious	0.9732	Pathogenic	-16.08	Pathogenic	0.78	Disease	0.56	Possibly damaging	0.02	Deleterious		
p.Leu130Thr	VUS	-2.31	Neutral	0.9455	Pathogenic	-11.34	Pathogenic	0.79	Disease	1.00	Probably damaging	0.19	Tolerated		
p.Leu130Arg	VUS	-53.15	Deleterious	0.8515	Pathogenic	-12.89	Pathogenic	0.90	Disease	0.99	Probably damaging	0.01	Deleterious	26.80	Deleterious
p.Leu130Ser	VUS	-3.13	Neutral	0.9847	Pathogenic	-12.78	Pathogenic	0.87	Disease	1.00	Probably damaging	0.04	Deleterious		
p.Leu130Ile	VUS	-0.30	Neutral	0.4013	Ambiguous	-6.51	Benign	0.60	Disease	1.00	Probably damaging	0.11	Tolerated		
p.Leu130Met	VUS	-0.88	Neutral	0.6895	Pathogenic	-8.81	Pathogenic	0.66	Disease	1.00	Probably damaging	0.05	Deleterious	24.90	Deleterious
p.Leu130His	VUS	-26.23	Indeterminate	0.9657	Pathogenic	-14.64	Pathogenic	0.87	Disease	1.00	Probably damaging	0.01	Deleterious		
p.Leu130Gln	VUS	-21.89	Indeterminate	0.9262	Pathogenic	-13.79	Pathogenic	0.89	Disease	0.99	Probably damaging	0.01	Deleterious	27.00	Deleterious
p.Leu130Pro	VUS	-53.15	Deleterious	0.9537	Pathogenic	-13.21	Pathogenic	0.92	Disease	1.00	Probably damaging	0.02	Deleterious	27.00	Deleterious
p.Leu130Asp	VUS	-53.15	Deleterious	0.9973	Pathogenic	-15.79	Pathogenic	0.74	Disease	1.00	Probably damaging	0.01	Deleterious		
p.Leu130Glu	VUS	-33.22	Indeterminate	0.9695	Pathogenic	-14.38	Pathogenic	0.79	Disease	1.00	Probably damaging	0.01	Deleterious		
p.Leu130Ala	VUS	-3.07	Neutral	0.8823	Pathogenic	-9.53	Pathogenic	0.76	Disease	1.00	Probably damaging	0.08	Tolerated		
p.Leu130Gly	VUS	-2.06	Neutral	0.9579	Pathogenic	-14.41	Pathogenic	0.79	Disease	1.00	Probably damaging	0.01	Deleterious		
p.Leu130Val	VUS	-0.32	Neutral	0.3976	Ambiguous	-6.54	Benign	0.67	Disease	0.97	Probably damaging	0.13	Tolerated	25.80	Deleterious
p.Leu130Tyr	VUS	-3.10	Neutral	0.9672	Pathogenic	-14.10	Pathogenic	0.74	Disease	0.99	Probably damaging	0.01	Deleterious		
p.Leu130Cys	VUS	-1.46	Neutral	0.8681	Pathogenic	-11.99	Pathogenic	0.72	Disease	1.00	Probably damaging	0.16	Tolerated		
p.Leu130Trp	VUS	-8.91	Indeterminate	0.9236	Pathogenic	-12.53	Pathogenic	0.62	Disease	1.00	Probably damaging	0.01	Deleterious		
p.Leu130Phe	VUS	-0.48	Neutral	0.7227	Pathogenic	-8.71	Pathogenic	0.67	Disease	1.00	Probably damaging	0.04	Deleterious		
p.Arg131Asn	VUS	-0.68	Neutral	0.451	Ambiguous	-8.74	Pathogenic	0.52	Disease	0.99	Probably damaging	0.27	Tolerated		
p.Arg131Lys	VUS	-0.01	Neutral	0.1996	Benign	-7.54	Pathogenic	0.48	Neutral	0.87	Possibly damaging	0.7	Tolerated		
p.Arg131Thr	VUS	-8.63	Indeterminate	0.2073	Benign	-7.55	Pathogenic	0.54	Disease	1.00	Probably damaging	0.38	Tolerated		
p.Arg131Ser	VUS	-1.15	Neutral	0.3002	Benign	-6.80	Benign	0.53	Disease	1.00	Probably damaging	0.34	Tolerated	25.70	Deleterious
p.Arg131Ile	VUS	-0.04	Neutral	0.2953	Benign	-9.77	Pathogenic	0.59	Disease	1.00	Probably damaging	0.34	Tolerated		
p.Arg131Met	VUS	-0.35	Neutral	0.3648	Ambiguous	-9.38	Pathogenic	0.58	Disease	1.00	Probably damaging	0.35	Tolerated		
p.Arg131His	VUS	-0.64	Neutral	0.1147	Benign	-6.76	Benign	0.33	Neutral	0.23	Benign	0.41	Tolerated	24.10	Deleterious
p.Arg131Gln	VUS	-22.33	Indeterminate	0.1263	Benign	-6.16	Benign	0.34	Neutral	1.00	Probably damaging	0.37	Tolerated		
p.Arg131Pro	VUS	-19.41	Indeterminate	0.7075	Pathogenic	-10.28	Pathogenic	0.64	Disease	1.00	Probably damaging	0.2	Tolerated	23.80	Deleterious
p.Arg131Leu	VUS	-0.02	Neutral	0.1195	Benign	-6.72	Benign	0.37	Neutral	1.00	Probably damaging	0.54	Tolerated	22.80	Deleterious
p.Arg131Asp	VUS	0.00	Neutral	0.3993	Ambiguous	-8.15	Pathogenic	0.59	Disease	1.00	Probably damaging	0.17	Tolerated		
p.Arg131Glu	VUS	-0.04	Neutral	0.2321	Benign	-6.63	Benign	0.53	Disease	0.98	Probably damaging	0.32	Tolerated		
p.Arg131Ala	VUS	-0.74	Neutral	0.1688	Benign	-5.48	Benign	0.53	Disease	1.00	Probably damaging	0.52	Tolerated		
p.Arg131Gly	VUS	-1.08	Neutral	0.1641	Benign	-6.87	Benign	0.38	Neutral	1.00	Probably damaging	0.26	Tolerated	25.70	Deleterious
p.Arg131Val	VUS	-0.61	Neutral	0.2515	Benign	-8.11	Pathogenic	0.58	Disease	1.00	Probably damaging	0.56	Tolerated		
p.Arg131Tyr	VUS	-0.93	Neutral	0.3346	Benign	-9.54	Pathogenic	0.53	Disease	0.93	Possibly damaging	0.58	Tolerated		
p.Arg131Cys	VUS	-53.15	Deleterious	0.2663	Benign	-8.50	Pathogenic	0.41	Neutral	1.00	Probably damaging	0.12	Tolerated	28.30	Deleterious
p.Arg131Trp	VUS	-0.79	Neutral	0.1928	Benign	-8.69	Pathogenic	0.58	Disease	1.00	Probably damaging	0.11	Tolerated		
p.Arg131Phe	VUS	0.00	Neutral	0.3727	Ambiguous	-10.29	Pathogenic	0.57	Disease	0.97	Probably damaging	0.41	Tolerated		
p.Ala132Asn	VUS	-33.21	Indeterminate	0.2461	Benign	-6.61	Benign	0.35	Neutral	0.00	Benign	0.08	Tolerated		
p.Ala132Lys	VUS	-3.78	Neutral	0.3425	Ambiguous	-6.43	Benign	0.46	Neutral	0.85	Possibly damaging	0.41	Tolerated		
p.Ala132Thr	VUS	-20.37	Indeterminate	0.1104	Benign	-5.18	Benign	0.31	Neutral	0.05	Benign	0.35	Tolerated	12.63	Neutral
p.Ala132Arg	VUS	-1.61	Neutral	0.2268	Benign	-4.54	Benign	0.38	Neutral	0.95	Possibly damaging	0.07	Tolerated		
p.Ala132Ser	VUS	-33.21	Indeterminate	0.1128	Benign	-4.01	Benign	0.29	Neutral	0.04	Benign	0.56	Tolerated	10.21	Neutral
p.Ala132Ile	VUS	-12.89	Indeterminate	0.3068	Benign	-7.48	Benign	0.47	Neutral	0.74	Possibly damaging	0.03	Deleterious		
p.Ala132Met	VUS	-8.62	Indeterminate	0.2506	Benign	-7.04	Benign	0.43	Neutral	0.98	Probably damaging	0.02	Deleterious		
p.Ala132His	VUS	-19.91	Indeterminate	0.2825	Benign	-6.65	Benign	0.46	Neutral	0.99	Probably damaging	0.02	Deleterious		
p.Ala132Gln	VUS	-1.62	Neutral	0.1939	Benign	-4.86	Benign	0.36	Neutral	0.98	Probably damaging	0.07	Tolerated		
p.Ala132Pro	VUS	-3.53	Neutral	0.5877	Pathogenic	-6.77	Benign	0.52	Disease	0.95	Possibly damaging	0.06	Tolerated	18.35	Deleterious
p.Ala132Leu	VUS	-9.42	Indeterminate	0.1643	Benign	-5.30	Benign	0.45	Neutral	0.74	Possibly damaging	0.05	Deleterious		
p.Ala132Asp	VUS	-29.25	Indeterminate	0.1618	Benign	-4.14	Benign	0.36	Neutral	0.01	Benign	0.08	Tolerated		
p.Ala132Glu	VUS	-9.67	Indeterminate	0.1341	Benign	-2.89	Benign	0.31	Neutral	0.67	Possibly damaging	0.1	Tolerated	10.63	Neutral
p.Ala132Gly	VUS	-53.15	Deleterious	0.1244	Benign	-3.15	Benign	0.29	Neutral	0.63	Possibly damaging	0.09	Tolerated	14.01	Neutral
p.Ala132Val	VUS	-4.37	Neutral	0.1471	Benign	-5.64	Benign	0.30	Neutral	0.11	Benign	0.06	Tolerated	10.93	Neutral
p.Ala132Tyr	VUS	-8.74	Indeterminate	0.3593	Ambiguous	-8.46	Pathogenic	0.46	Neutral	0.99	Probably damaging	0.01	Deleterious		
p.Ala132Cys	VUS	-14.40	Indeterminate	0.4755	Ambiguous	-6.02	Benign	0.44	Neutral	0.99	Probably damaging	0.02	Deleterious		
p.Ala132Trp	VUS	-4.92	Neutral	0.4766	Ambiguous	-7.45	Benign	0.51	Disease	1.00	Probably damaging	0.01	Deleterious		
p.Ala132Phe	VUS	-4.29	Neutral	0.29	Benign	-8.13	Pathogenic	0.46	Neutral	0.98	Probably damaging	0.01	Deleterious		
p.Ala133Asn	VUS	-0.02	Neutral	0.4307	Ambiguous	-7.78	Pathogenic	0.40	Neutral	0.00	Benign	0.33	Tolerated		
p.Ala133Lys	VUS	-0.09	Neutral	0.5727	Pathogenic	-5.53	Benign	0.51	Disease	0.65	Possibly damaging	0.36	Tolerated		
p.Ala133Thr	VUS	-0.13	Neutral	0.125	Benign	-5.14	Benign	0.43	Neutral	0.00	Benign	0.42	Tolerated	18.39	Deleterious
p.Ala133Arg	VUS	-2.27	Neutral	0.3825	Ambiguous	-3.20	Benign	0.50	Disease	0.87	Possibly damaging	0.39	Tolerated		
p.Ala133Ser	VUS	-0.11	Neutral	0.1281	Benign	-5.87	Benign	0.31	Neutral	0.01	Benign	0.43	Tolerated	16.65	Deleterious
p.Ala133Ile	VUS	-0.71	Neutral	0.3424	Ambiguous	-5.89	Benign	0.39	Neutral	0.48	Possibly damaging	0.4	Tolerated		
p.Ala133Met	VUS	-0.01	Neutral	0.3164	Benign	-4.42	Benign	0.49	Neutral	0.93	Possibly damaging	0.22	Tolerated		
p.Ala133His	VUS	-0.26	Neutral	0.449	Ambiguous	-5.71	Benign	0.50	Neutral	0.98	Probably damaging	0.55	Tolerated		
p.Ala133Gln	VUS	-0.08	Neutral	0.3327	Benign	-3.97	Benign	0.51	Disease	0.87	Possibly damaging	0.33	Tolerated		
p.Ala133Pro	VUS	-0.37	Neutral	0.5773	Pathogenic	-6.87	Benign	0.38	Neutral	0.73	Possibly damaging	0.22	Tolerated	22.30	Deleterious
p.Ala133Leu	VUS	-0.18	Neutral	0.2337	Benign	-3.65	Benign	0.49	Neutral	0.48	Possibly damaging	0.66	Tolerated		
p.Ala133Asp	VUS	-7.43	Indeterminate	0.4543	Ambiguous	-7.93	Pathogenic	0.57	Disease	0.16	Benign	0.24	Tolerated	17.88	Deleterious
p.Ala133Glu	VUS	-2.21	Neutral	0.2918	Benign	-5.22	Benign	0.50	Neutral	0.41	Benign	0.32	Tolerated		
p.Ala133Gly	VUS	-1.09	Neutral	0.178	Benign	-5.15	Benign	0.45	Neutral	0.00	Benign	0.35	Tolerated	16.79	Deleterious
p.Ala133Val	VUS	-2.44	Neutral	0.1407	Benign	-3.13	Benign	0.31	Neutral	0.01	Benign	0.51	Tolerated	13.93	Neutral
p.Ala133Tyr	VUS	-1.01	Neutral	0.5866	Pathogenic	-8.24	Pathogenic	0.53	Disease	0.98	Probably damaging	1	Tolerated		
p.Ala133Cys	VUS	-0.27	Neutral	0.4304	Ambiguous	-6.36	Benign	0.50	Neutral	0.98	Probably damaging	0.18	Tolerated		
p.Ala133Trp	VUS	-0.43	Neutral	0.7089	Pathogenic	-6.23	Benign	0.55	Disease	0.99	Probably damaging	0.18	Tolerated		
p.Ala133Phe	VUS	-0.96	Neutral	0.4143	Ambiguous	-7.72	Pathogenic	0.53	Disease	0.93	Possibly damaging	0.7	Tolerated		
p.Ala134Asn	VUS	-0.10	Neutral	0.2328	Benign	-7.73	Pathogenic	0.37	Neutral	0.03	Benign	0.2	Tolerated		
p.Ala134Lys	VUS	-0.93	Neutral	0.3067	Benign	-7.53	Pathogenic	0.38	Neutral	0.29	Benign	0.31	Tolerated		
p.Ala134Thr	VUS	-2.44	Neutral	0.0865	Benign	-5.69	Benign	0.23	Neutral	0.00	Benign	1	Tolerated	0.94	Neutral
p.Ala134Arg	VUS	-2.27	Neutral	0.1773	Benign	-5.05	Benign	0.37	Neutral	0.45	Possibly damaging	0.19	Tolerated		
p.Ala134Ser	VUS	-1.36	Neutral	0.0891	Benign	-5.10	Benign	0.19	Neutral	0.00	Benign	0.47	Tolerated	0.19	Neutral
p.Ala134Ile	VUS	-0.33	Neutral	0.2793	Benign	-7.47	Benign	0.39	Neutral	0.29	Benign	0.55	Tolerated		
p.Ala134Met	VUS	-0.23	Neutral	0.2727	Benign	-5.48	Benign	0.33	Neutral	0.86	Possibly damaging	0.11	Tolerated		
p.Ala134His	VUS	-0.11	Neutral	0.2608	Benign	-5.82	Benign	0.37	Neutral	0.86	Possibly damaging	0.08	Tolerated		
p.Ala134Gln	VUS	-0.26	Neutral	0.1889	Benign	-3.90	Benign	0.29	Neutral	0.59	Possibly damaging	0.38	Tolerated		
p.Ala134Pro	VUS	-0.55	Neutral	0.1837	Benign	-5.78	Benign	0.39	Neutral	0.25	Benign	0.18	Tolerated	9.21	Neutral
p.Ala134Leu	VUS	-0.05	Neutral	0.1652	Benign	-4.97	Benign	0.31	Neutral	0.15	Benign	0.35	Tolerated		
p.Ala134Asp	VUS	-0.09	Neutral	0.1632	Benign	-6.35	Benign	0.37	Neutral	0.10	Benign	0.19	Tolerated		
p.Ala134Glu	VUS	-0.09	Neutral	0.1294	Benign	-4.64	Benign	0.25	Neutral	0.00	Benign	0.3	Tolerated	10.08	Neutral
p.Ala134Gly	VUS	-0.40	Neutral	0.0788	Benign	-4.12	Benign	0.28	Neutral	0.00	Benign	0.14	Tolerated	10.93	Neutral
p.Ala134Val	VUS	-0.10	Neutral	0.1345	Benign	-5.35	Benign	0.27	Neutral	0.02	Benign	0.81	Tolerated	10.60	Neutral
p.Ala134Tyr	VUS	-0.75	Neutral	0.3656	Ambiguous	-8.41	Pathogenic	0.39	Neutral	0.95	Possibly damaging	0.08	Tolerated		
p.Ala134Cys	VUS	-1.54	Neutral	0.3103	Benign	-6.67	Benign	0.37	Neutral	0.95	Possibly damaging	0.1	Tolerated		
p.Ala134Trp	VUS	-0.83	Neutral	0.4264	Ambiguous	-7.45	Benign	0.40	Neutral	0.99	Probably damaging	0.02	Deleterious		
p.Ala134Phe	VUS	-0.06	Neutral	0.2995	Benign	-8.52	Pathogenic	0.39	Neutral	0.86	Possibly damaging	0.09	Tolerated		
p.Gly135Asn	VUS	-0.01	Neutral	0.2191	Benign	-6.23	Benign	0.26	Neutral	0.98	Probably damaging	0.07	Tolerated		
p.Gly135Lys	VUS	-3.29	Neutral	0.3025	Benign	-6.29	Benign	0.46	Neutral	0.96	Probably damaging	0.11	Tolerated		
p.Gly135Thr	VUS	-0.86	Neutral	0.1142	Benign	-4.91	Benign	0.42	Neutral	0.98	Probably damaging	0.16	Tolerated		

p.Gly135Arg	VUS	0.00	Neutral	0.1684	Benign	-4.16	Benign	0.29	Neutral	0.97	Probably damaging	0.08	Tolerated	14.69	Neutral
p.Gly135Ser	VUS	-1.39	Neutral	0.1081	Benign	-4.09	Benign	0.18	Neutral	0.93	Possibly damaging	0.44	Tolerated		
p.Gly135Ile	VUS	-2.16	Neutral	0.2628	Benign	-7.30	Benign	0.45	Neutral	1.00	Probably damaging	0.06	Tolerated		
p.Gly135Met	VUS	-0.29	Neutral	0.4175	Ambiguous	-6.81	Benign	0.46	Neutral	1.00	Probably damaging	0.05	Deleterious		
p.Gly135His	VUS	-0.47	Neutral	0.2218	Benign	-6.08	Benign	0.45	Neutral	1.00	Probably damaging	0.03	Deleterious		
p.Gly135Gln	VUS	-0.74	Neutral	0.2094	Benign	-4.77	Benign	0.42	Neutral	0.96	Probably damaging	0.07	Tolerated		
p.Gly135Pro	VUS	-0.58	Neutral	0.3056	Benign	-4.07	Benign	0.41	Neutral	0.99	Probably damaging	0.22	Tolerated		
p.Gly135Leu	VUS	-1.45	Neutral	0.2535	Benign	-5.17	Benign	0.43	Neutral	0.98	Probably damaging	0.23	Tolerated		
p.Gly135Asp	VUS	-1.16	Neutral	0.1262	Benign	-4.55	Benign	0.30	Neutral	0.88	Possibly damaging	0.08	Tolerated		
p.Gly135Glu	VUS	-31.01	Indeterminate	0.1313	Benign	-3.64	Benign	0.20	Neutral	0.07	Benign	0.1	Tolerated	11.99	Neutral
p.Gly135Ala	VUS	-0.30	Neutral	0.0882	Benign	-3.00	Benign	0.31	Neutral	0.91	Possibly damaging	1	Tolerated	14.70	Neutral
p.Gly135Val	VUS	-1.45	Neutral	0.1561	Benign	-5.17	Benign	0.40	Neutral	0.99	Probably damaging	0.16	Tolerated	19.92	Deleterious
p.Gly135Tyr	VUS	-0.60	Neutral	0.3453	Ambiguous	-7.46	Benign	0.45	Neutral	1.00	Probably damaging	0.04	Deleterious		
p.Gly135Cys	VUS	-0.05	Neutral	0.2046	Benign	-6.28	Benign	0.39	Neutral	1.00	Probably damaging	0.09	Tolerated		
p.Gly135Trp	VUS	-0.45	Neutral	0.2645	Benign	-6.78	Benign	0.43	Neutral	1.00	Probably damaging	0.02	Deleterious	21.20	Deleterious
p.Gly135Phe	VUS	-0.98	Neutral	0.4177	Ambiguous	-7.49	Benign	0.45	Neutral	1.00	Probably damaging	0.05	Deleterious		
p.Gly136Asn	VUS	-7.38	Indeterminate	0.1736	Benign	-5.90	Benign	0.24	Neutral	0.00	Benign	0.21	Tolerated		
p.Gly136Lys	VUS	-0.08	Neutral	0.2592	Benign	-5.97	Benign	0.32	Neutral	0.36	Benign	0.23	Tolerated		
p.Gly136Thr	VUS	-0.05	Neutral	0.1004	Benign	-4.43	Benign	0.26	Neutral	0.52	Possibly damaging	0.41	Tolerated		
p.Gly136Arg	VUS	-0.04	Neutral	0.1522	Benign	-4.07	Benign	0.19	Neutral	0.03	Benign	0.19	Tolerated	12.18	Neutral
p.Gly136Ser	VUS	-5.18	Neutral	0.0975	Benign	-3.77	Benign	0.16	Neutral	0.15	Benign	0.65	Tolerated	12.00	Neutral
p.Gly136Ile	VUS	-0.50	Neutral	0.1965	Benign	-6.12	Benign	0.35	Neutral	0.79	Possibly damaging	0.24	Tolerated		
p.Gly136Met	VUS	-0.11	Neutral	0.3032	Benign	-6.66	Benign	0.33	Neutral	0.99	Probably damaging	0.1	Tolerated		
p.Gly136His	VUS	-8.50	Indeterminate	0.1781	Benign	-5.62	Benign	0.33	Neutral	0.02	Benign	0.16	Tolerated		
p.Gly136Gln	VUS	-0.68	Neutral	0.1665	Benign	-4.91	Benign	0.29	Neutral	0.89	Possibly damaging	0.18	Tolerated		
p.Gly136Pro	VUS	-17.41	Indeterminate	0.2606	Benign	-3.68	Benign	0.34	Neutral	0.89	Possibly damaging	0.27	Tolerated		
p.Gly136Leu	VUS	-6.43	Indeterminate	0.2113	Benign	-4.75	Benign	0.33	Neutral	0.52	Possibly damaging	0.29	Tolerated		
p.Gly136Asp	VUS	-22.04	Indeterminate	0.0963	Benign	-4.26	Benign	0.15	Neutral	0.00	Benign	0.19	Tolerated	0.01	Neutral
p.Gly136Glu	VUS	-0.28	Neutral	0.0992	Benign	-3.98	Benign	0.19	Neutral	0.36	Benign	0.22	Tolerated		
p.Gly136Ala	VUS	-0.61	Neutral	0.09	Benign	-3.42	Benign	0.12	Neutral	0.00	Benign	1	Tolerated	0.04	Neutral
p.Gly136Val	VUS	-0.13	Neutral	0.1269	Benign	-4.79	Benign	0.26	Neutral	0.29	Benign	0.35	Tolerated	7.01	Neutral
p.Gly136Tyr	VUS	-2.12	Neutral	0.2855	Benign	-7.52	Pathogenic	0.37	Neutral	0.89	Possibly damaging	0.27	Tolerated		
p.Gly136Cys	VUS	-9.10	Indeterminate	0.1768	Benign	-6.13	Benign	0.32	Neutral	0.01	Benign	0.13	Tolerated	13.10	Neutral
p.Gly136Trp	VUS	-3.05	Neutral	0.2066	Benign	-6.85	Benign	0.34	Neutral	0.99	Probably damaging	0.06	Tolerated		
p.Gly136Phe	VUS	-3.24	Neutral	0.3221	Benign	-7.08	Benign	0.34	Neutral	0.96	Probably damaging	0.21	Tolerated		
p.Thr137Asn	VUS	-0.55	Neutral	0.0965	Benign	-4.57	Benign	0.23	Neutral	0.65	Possibly damaging	0.55	Tolerated	14.08	Neutral
p.Thr137Lys	VUS	-0.52	Neutral	0.1405	Benign	-4.41	Benign	0.35	Neutral	0.55	Possibly damaging	0.23	Tolerated		
p.Thr137Arg	VUS	-1.34	Neutral	0.0794	Benign	-2.14	Benign	0.29	Neutral	0.02	Benign	0.15	Tolerated		
p.Thr137Ser	VUS	-0.38	Neutral	0.0779	Benign	-2.45	Benign	0.16	Neutral	0.01	Benign	0.45	Tolerated	6.14	Neutral
p.Thr137Ile	VUS	-3.13	Neutral	0.1468	Benign	-5.11	Benign	0.35	Neutral	0.48	Possibly damaging	0.06	Tolerated	15.18	Deleterious
p.Thr137Met	VUS	-0.62	Neutral	0.1281	Benign	-5.43	Benign	0.37	Neutral	0.95	Possibly damaging	0.03	Deleterious		
p.Thr137His	VUS	-1.76	Neutral	0.1225	Benign	-3.97	Benign	0.41	Neutral	0.99	Probably damaging	0.06	Tolerated		
p.Thr137Gln	VUS	-3.36	Neutral	0.1096	Benign	-3.44	Benign	0.36	Neutral	0.90	Possibly damaging	0.36	Tolerated		
p.Thr137Pro	VUS	-0.52	Neutral	0.056	Benign	-2.24	Benign	0.19	Neutral	0.01	Benign	0.1	Tolerated	6.04	Neutral
p.Thr137Leu	VUS	-0.74	Neutral	0.0931	Benign	-3.41	Benign	0.40	Neutral	0.34	Benign	0.08	Tolerated		
p.Thr137Asp	VUS	-1.83	Neutral	0.1409	Benign	-2.83	Benign	0.40	Neutral	0.71	Possibly damaging	0.24	Tolerated		
p.Thr137Glu	VUS	-1.17	Neutral	0.1202	Benign	-2.54	Benign	0.40	Neutral	0.71	Possibly damaging	0.2	Tolerated		
p.Thr137Ala	VUS	-3.04	Neutral	0.0728	Benign	-2.00	Benign	0.13	Neutral	0.00	Benign	0.21	Tolerated	2.64	Neutral
p.Thr137Gly	VUS	-0.57	Neutral	0.0918	Benign	-1.59	Benign	0.43	Neutral	0.34	Benign	0.16	Tolerated		
p.Thr137Val	VUS	-0.45	Neutral	0.1196	Benign	-3.47	Benign	0.39	Neutral	0.03	Benign	0.09	Tolerated		
p.Thr137Tyr	VUS	-0.56	Neutral	0.1962	Benign	-6.09	Benign	0.42	Neutral	0.98	Probably damaging	0.04	Deleterious		
p.Thr137Cys	VUS	-6.22	Indeterminate	0.2876	Benign	-4.48	Benign	0.41	Neutral	0.99	Probably damaging	0.03	Deleterious		
p.Thr137Trp	VUS	-0.80	Neutral	0.2606	Benign	-4.81	Benign	0.47	Neutral	1.00	Probably damaging	0.01	Deleterious		
p.Thr137Phe	VUS	-0.80	Neutral	0.1667	Benign	-5.57	Benign	0.43	Neutral	0.95	Possibly damaging	0.03	Deleterious		
p.Arg138Asn	VUS	-1.57	Neutral	0.2106	Benign	-4.35	Benign	0.27	Neutral	0.00	Benign	0.34	Tolerated		
p.Arg138Lys	VUS	-0.03	Neutral	0.142	Benign	-5.07	Benign	0.17	Neutral	0.00	Benign	1	Tolerated	0.10	Neutral
p.Arg138Thr	VUS	-0.25	Neutral	0.0777	Benign	-3.90	Benign	0.13	Neutral	0.00	Benign	0.38	Tolerated	0.07	Neutral
p.Arg138Ser	VUS	0.00	Neutral	0.1182	Benign	-3.18	Benign	0.19	Neutral	0.00	Benign	0.43	Tolerated	7.95	Neutral
p.Arg138Ile	VUS	-0.01	Neutral	0.1574	Benign	-5.73	Benign	0.25	Neutral	0.01	Benign	0.16	Tolerated	6.18	Neutral
p.Arg138Met	VUS	0.00	Neutral	0.2112	Benign	-5.98	Benign	0.26	Neutral	0.11	Benign	0.09	Tolerated		
p.Arg138His	VUS	-1.18	Neutral	0.0894	Benign	-4.65	Benign	0.17	Neutral	0.07	Benign	0.17	Tolerated		
p.Arg138Gln	VUS	-0.05	Neutral	0.0888	Benign	-4.23	Benign	0.12	Neutral	0.00	Benign	0.47	Tolerated		
p.Arg138Pro	VUS	-4.52	Neutral	0.081	Benign	-2.99	Benign	0.24	Neutral	0.03	Benign	0.2	Tolerated		
p.Arg138Leu	VUS	0.00	Neutral	0.1027	Benign	-4.21	Benign	0.22	Neutral	0.00	Benign	0.27	Tolerated		
p.Arg138Asp	VUS	-0.07	Neutral	0.1354	Benign	-3.15	Benign	0.17	Neutral	0.00	Benign	0.32	Tolerated		
p.Arg138Glu	VUS	-0.95	Neutral	0.1114	Benign	-3.13	Benign	0.15	Neutral	0.00	Benign	0.57	Tolerated		
p.Arg138Ala	VUS	-1.36	Neutral	0.0753	Benign	-2.56	Benign	0.26	Neutral	0.00	Benign	0.46	Tolerated		
p.Arg138Gly	VUS	-0.38	Neutral	0.0609	Benign	-2.55	Benign	0.10	Neutral	0.00	Benign	0.26	Tolerated	9.06	Neutral
p.Arg138Val	VUS	-0.15	Neutral	0.1281	Benign	-4.09	Benign	0.29	Neutral	0.00	Benign	0.23	Tolerated		
p.Arg138Tyr	VUS	0.00	Neutral	0.225	Benign	-6.28	Benign	0.28	Neutral	0.18	Benign	0.14	Tolerated		
p.Arg138Cys	VUS	-0.01	Neutral	0.1701	Benign	-5.08	Benign	0.23	Neutral	0.43	Benign	0.05	Deleterious		
p.Arg138Trp	VUS	0.00	Neutral	0.1311	Benign	-6.12	Benign	0.21	Neutral	0.43	Benign	0.04	Deleterious		
p.Arg138Phe	VUS	-0.11	Neutral	0.2553	Benign	-6.12	Benign	0.29	Neutral	0.03	Benign	0.1	Tolerated		
p.Gly139Asn	VUS	-2.55	Neutral	0.1977	Benign	-5.50	Benign	0.40	Neutral	0.30	Benign	0	Deleterious		
p.Gly139Lys	VUS	-4.57	Neutral	0.3065	Benign	-5.76	Benign	0.43	Neutral	0.02	Benign	0	Deleterious		
p.Gly139Thr	VUS	-7.65	Indeterminate	0.1081	Benign	-4.38	Benign	0.31	Neutral	0.18	Benign	0	Deleterious		
p.Gly139Arg	VUS	-1.61	Neutral	0.17	Benign	-3.75	Benign	0.26	Neutral	0.00	Benign	0	Deleterious	10.44	Neutral
p.Gly139Ser	VUS	-4.27	Neutral	0.1007	Benign	-3.68	Benign	0.18	Neutral	0.00	Benign	0	Deleterious	4.19	Neutral
p.Gly139Ile	VUS	-1.29	Neutral	0.1859	Benign	-5.82	Benign	0.46	Neutral	0.18	Benign	0	Deleterious		
p.Gly139Met	VUS	-5.11	Neutral	0.3266	Benign	-6.63	Benign	0.46	Neutral	0.90	Possibly damaging	0	Deleterious		
p.Gly139His	VUS	-1.68	Neutral	0.191	Benign	-5.23	Benign	0.32	Neutral	0.00	Benign	0	Deleterious		
p.Gly139Gln	VUS	-1.02	Neutral	0.185	Benign	-4.97	Benign	0.42	Neutral	0.04	Benign	0	Deleterious		
p.Gly139Pro	VUS	-6.78	Indeterminate	0.2134	Benign	-3.36	Benign	0.42	Neutral	0.75	Possibly damaging	0	Deleterious		
p.Gly139Leu	VUS	-3.93	Neutral	0.2184	Benign	-4.61	Benign	0.44	Neutral	0.18	Benign	0	Deleterious		
p.Gly139Asp	VUS	-7.08	Indeterminate	0.1074	Benign	-4.34	Benign	0.27	Neutral	0.01	Benign	0	Deleterious	15.62	Deleterious
p.Gly139Glu	VUS	-3.30	Neutral	0.1158	Benign	-4.40	Benign	0.38	Neutral	0.15	Benign	0	Deleterious		
p.Gly139Ala	VUS	-7.05	Indeterminate	0.0943	Benign	-3.44	Benign	0.19	Neutral	0.03	Benign	0	Deleterious	14.30	Neutral
p.Gly139Val	VUS	-0.26	Neutral	0.1219	Benign	-4.71	Benign	0.29	Neutral	0.01	Benign	0	Deleterious	15.89	Deleterious
p.Gly139Tyr	VUS	-11.79	Indeterminate	0.2451	Benign	-6.64	Benign	0.47	Neutral	0.60	Possibly damaging	0	Deleterious		
p.Gly139Cys	VUS	-0.55	Neutral	0.1649	Benign	-5.70	Benign	0.42	Neutral	0.00	Benign	0	Deleterious	11.46	Neutral
p.Gly139Trp	VUS	-2.00	Neutral	0.1747	Benign	-6.41	Benign	0.44	Neutral	0.97	Probably damaging	0	Deleterious		
p.Gly139Phe	VUS	-4.58	Neutral	0.3282	Benign	-6.45	Benign	0.45	Neutral	0.75	Possibly damaging	0	Deleterious		
p.Ser140Asn	VUS	-0.75	Neutral	0.1255	Benign	-4.78	Benign	0.20	Neutral	0.07	Benign	0.86	Tolerated	10.99	Neutral
p.Ser140Lys	VUS	-0.33	Neutral	0.3318	Benign	-4.95	Benign	0.28	Neutral	0.05	Benign	0.48	Tolerated		
p.Ser140Thr	VUS	-0.31	Neutral	0.0958	Benign	-4.00	Benign	0.19	Neutral	0.26	Benign	0.74	Tolerated	14.43	Neutral
p.Ser140Arg	VUS	-3.36	Neutral	0.1902	Benign	-3.15	Benign	0.21	Neutral	0.00	Benign	0.34	Tolerated	3.22	Neutral
p.Ser140Ile	VUS	-0.10	Neutral	0.2008	Benign	-5.33	Benign	0.30	Neutral	0.55	Possibly damaging	0.14	Tolerated	16.39	Deleterious
p.Ser140Met	VUS	-0.98	Neutral	0.2592	Benign	-6.10	Benign	0.34	Neutral	0.83	Possibly damaging	0.08	Tolerated		
p.Ser140His	VUS	-1.04	Neutral	0.1677	Benign	-4.67	Benign	0.28	Neutral	0.00	Benign	0.33	Tolerated		
p.Ser140Gln	VUS	-0.44	Neutral	0.2021	Benign	-4.32	Benign	0.28	Neutral	0.01	Benign	0.3	Tolerated		
p.Ser140Pro	VUS	-6.82	Indeterminate	0.0917	Benign	-3.10	Benign	0.20	Neutral	0.59	Possibly damaging	0.26	Tolerated		
p.Ser140Leu	VUS	-0.36	Neutral	0.1651	Benign	-4.23	Benign	0.21	Neutral	0.19	Benign	0.23	Tolerated		
p.Ser140Asp	VUS	-0.17	Neutral	0.1572	Benign	-3.67	Benign	0.25	Neutral	0.00	Benign	0.73	Tolerated		
p.Ser140Glu	VUS	-0.32	Neutral	0.1861	Benign	-3.54	Benign	0.28	Neutral	0.05	Benign	0.41	Tolerated		
p.Ser140Ala	VUS	-5.04	Neutral	0.0814	Benign	-3.00	Benign	0.21	Neutral	0.02	Benign	0.55	Tolerated		
p.Ser140Gly	VUS	-7.24	Indeterminate	0.0608	Benign	-2.61	Benign	0.11	Neutral	0.00	Benign	0.62	Tolerated	0.04	Neutral
p.Ser140Val	VUS	-0.10	Neutral	0.1746	Benign	-4.28	Benign	0.30	Neutral	0.32	Benign	0.22	Tolerated		
p.Ser140Tyr	VUS	-0.45	Neutral	0.1917	Benign	-6.16	Benign	0.34	Neutral	0.45	Benign	0.25	Tolerated		
p.Ser140Cys	VUS	-0.72	Neutral	0.1042	Benign	-5.25	Benign	0.26	Neutral	0.93	Possibly damaging	0.1	Tolerated	14.40	Neutral
p.Ser140Trp	VUS	-1.21	Neutral	0.15	Benign	-5.53	Benign	0.36	Neutral	0.95	Possibly damaging	0.06	Tolerated		
p.Ser140Phe	VUS	-1.17	Neutral	0.2242	Benign	-6.05	Benign	0.31	Neutral	0.62	Possibly damaging	0.16	Tolerated		

p.Asn141Lys	VUS	-0.39	Neutral	0.2591	Benign	-0.77	Benign	0.11	Neutral	0.01	Benign	0.72	Tolerated	11.59	Neutral
p.Asn141Thr	VUS	-2.77	Neutral	0.0964	Benign	-1.83	Benign	0.11	Neutral	0.01	Benign	0.25	Tolerated	1.26	Neutral
p.Asn141Arg	VUS	-0.97	Neutral	0.1387	Benign	-0.94	Benign	0.15	Neutral	0.00	Benign	1	Tolerated		
p.Asn141Ser	VUS	-0.22	Neutral	0.06	Benign	-0.87	Benign	0.05	Neutral	0.00	Benign	0.31	Tolerated	0.01	Neutral
p.Asn141Ile	VUS	-0.37	Neutral	0.1859	Benign	-3.06	Benign	0.22	Neutral	0.01	Benign	0.08	Tolerated	9.60	Neutral
p.Asn141Met	VUS	-0.27	Neutral	0.3507	Ambiguous	-3.84	Benign	0.25	Neutral	0.10	Benign	0.05	Deleterious		
p.Asn141His	VUS	-2.15	Neutral	0.0972	Benign	-2.50	Benign	0.15	Neutral	0.14	Benign	0.15	Tolerated	11.25	Neutral
p.Asn141Gln	VUS	-0.80	Neutral	0.2108	Benign	-2.04	Benign	0.13	Neutral	0.03	Benign	0.31	Tolerated		
p.Asn141Pro	VUS	-1.00	Neutral	0.1131	Benign	-0.78	Benign	0.20	Neutral	0.03	Benign	0.14	Tolerated		
p.Asn141Leu	VUS	-0.14	Neutral	0.1576	Benign	-1.46	Benign	0.21	Neutral	0.00	Benign	0.15	Tolerated		
p.Asn141Asp	VUS	-0.21	Neutral	0.10	Benign	-1.68	Benign	0.07	Neutral	0.00	Benign	0.33	Tolerated	5.52	Neutral
p.Asn141Glu	VUS	-1.28	Neutral	0.2015	Benign	-1.38	Benign	0.13	Neutral	0.01	Benign	0.32	Tolerated		
p.Asn141Ala	VUS	-0.22	Neutral	0.1119	Benign	-0.72	Benign	0.19	Neutral	0.00	Benign	0.25	Tolerated		
p.Asn141Gly	VUS	-0.33	Neutral	0.128	Benign	-0.38	Benign	0.10	Neutral	0.00	Benign	0.28	Tolerated		
p.Asn141Val	VUS	-0.04	Neutral	0.1653	Benign	-1.97	Benign	0.23	Neutral	0.01	Benign	0.11	Tolerated		
p.Asn141Tyr	VUS	-0.90	Neutral	0.14	Benign	-4.40	Benign	0.19	Neutral	0.14	Benign	0.1	Tolerated	12.95	Neutral
p.Asn141Cys	VUS	-2.57	Neutral	0.1935	Benign	-2.75	Benign	0.24	Neutral	0.18	Benign	0.03	Deleterious		
p.Asn141Trp	VUS	-0.06	Neutral	0.4218	Ambiguous	-3.02	Benign	0.28	Neutral	0.71	Possibly damaging	0.03	Deleterious		
p.Asn141Phe	VUS	-0.47	Neutral	0.3924	Ambiguous	-3.45	Benign	0.25	Neutral	0.10	Benign	0.06	Tolerated		
p.His142Asn	VUS	0.00	Neutral	0.1197	Benign	-3.33	Benign	0.25	Neutral	0.02	Benign	1	Tolerated	11.88	Neutral
p.His142Lys	VUS	0.00	Neutral	0.2146	Benign	-3.01	Benign	0.28	Neutral	0.01	Benign	0.17	Tolerated		
p.His142Thr	VUS	0.00	Neutral	0.1444	Benign	-2.18	Benign	0.30	Neutral	0.01	Benign	0.12	Tolerated		
p.His142Arg	VUS	0.00	Neutral	0.0718	Benign	-1.68	Benign	0.23	Neutral	0.00	Benign	0.11	Tolerated	0.02	Neutral
p.His142Ser	VUS	0.00	Neutral	0.1269	Benign	-1.78	Benign	0.25	Neutral	0.00	Benign	0.22	Tolerated		
p.His142Ile	VUS	0.00	Neutral	0.2557	Benign	-3.13	Benign	0.31	Neutral	0.09	Benign	0.03	Deleterious		
p.His142Met	VUS	0.00	Neutral	0.3804	Ambiguous	-3.62	Benign	0.32	Neutral	0.52	Possibly damaging	0.02	Deleterious		
p.His142Gln	VUS	-0.01	Neutral	0.1289	Benign	-2.77	Benign	0.19	Neutral	0.00	Benign	0.1	Tolerated	0.01	Neutral
p.His142Pro	VUS	0.00	Neutral	0.0702	Benign	-1.34	Benign	0.18	Neutral	0.00	Benign	0.07	Tolerated	0.05	Neutral
p.His142Leu	VUS	0.00	Neutral	0.1061	Benign	-1.95	Benign	0.31	Neutral	0.02	Benign	0.06	Tolerated	4.22	Neutral
p.His142Asp	VUS	0.00	Neutral	0.1004	Benign	-2.16	Benign	0.22	Neutral	0.02	Benign	0.35	Tolerated	12.83	Neutral
p.His142Glu	VUS	0.00	Neutral	0.1352	Benign	-1.77	Benign	0.27	Neutral	0.01	Benign	0.15	Tolerated		
p.His142Ala	VUS	0.00	Neutral	0.1025	Benign	-1.08	Benign	0.33	Neutral	0.01	Benign	0.11	Tolerated		
p.His142Gly	VUS	0.00	Neutral	0.1126	Benign	-0.62	Benign	0.26	Neutral	0.01	Benign	0.25	Tolerated		
p.His142Val	VUS	0.00	Neutral	0.1537	Benign	-2.18	Benign	0.32	Neutral	0.05	Benign	0.05	Deleterious		
p.His142Tyr	VUS	0.00	Neutral	0.1167	Benign	-4.44	Benign	0.29	Neutral	0.07	Benign	0.09	Tolerated	15.56	Deleterious
p.His142Cys	VUS	0.00	Neutral	0.2166	Benign	-3.85	Benign	0.26	Neutral	0.81	Possibly damaging	0.03	Deleterious		
p.His142Trp	VUS	0.00	Neutral	0.2488	Benign	-4.24	Benign	0.32	Neutral	0.81	Possibly damaging	0.02	Deleterious		
p.His142Phe	VUS	-0.01	Neutral	0.2291	Benign	-3.98	Benign	0.32	Neutral	0.27	Benign	0.05	Deleterious		
p.Ala143Asn	VUS	-1.04	Neutral	0.1998	Benign	-4.62	Benign	0.35	Neutral	0.69	Possibly damaging	0.54	Tolerated		
p.Ala143Lys	VUS	-0.58	Neutral	0.2701	Benign	-4.70	Benign	0.27	Neutral	0.69	Possibly damaging	0.67	Tolerated		
p.Ala143Thr	VUS	-0.48	Neutral	0.0847	Benign	-4.30	Benign	0.28	Neutral	0.00	Benign	0.56	Tolerated	8.17	Neutral
p.Ala143Arg	VUS	-0.80	Neutral	0.1332	Benign	-3.14	Benign	0.35	Neutral	0.69	Possibly damaging	0.52	Tolerated		
p.Ala143Ser	VUS	-0.86	Neutral	0.0842	Benign	-3.57	Benign	0.26	Neutral	0.19	Benign	0.72	Tolerated	12.41	Neutral
p.Ala143Ile	VUS	-0.71	Neutral	0.2309	Benign	-5.25	Benign	0.27	Neutral	0.24	Benign	0.33	Tolerated		
p.Ala143Met	VUS	-0.89	Neutral	0.2533	Benign	-5.97	Benign	0.39	Neutral	0.94	Possibly damaging	0.18	Tolerated		
p.Ala143His	VUS	-1.17	Neutral	0.1867	Benign	-4.21	Benign	0.37	Neutral	0.98	Probably damaging	0.42	Tolerated		
p.Ala143Gln	VUS	-0.91	Neutral	0.1507	Benign	-4.16	Benign	0.33	Neutral	0.82	Possibly damaging	0.44	Tolerated		
p.Ala143Pro	VUS	-1.82	Neutral	0.0784	Benign	-3.37	Benign	0.27	Neutral	0.77	Possibly damaging	0.34	Tolerated	16.83	Deleterious
p.Ala143Leu	VUS	-0.93	Neutral	0.1214	Benign	-4.00	Benign	0.37	Neutral	0.24	Benign	0.53	Tolerated		
p.Ala143Asp	VUS	-0.87	Neutral	0.118	Benign	-3.80	Benign	0.38	Neutral	0.62	Possibly damaging	0.54	Tolerated	15.10	Deleterious
p.Ala143Glu	VUS	-0.69	Neutral	0.117	Benign	-3.79	Benign	0.33	Neutral	0.69	Possibly damaging	0.65	Tolerated		
p.Ala143Gly	VUS	-0.83	Neutral	0.0776	Benign	-3.01	Benign	0.30	Neutral	0.32	Benign	0.61	Tolerated	14.00	Neutral
p.Ala143Val	VUS	-0.68	Neutral	0.1113	Benign	-4.29	Benign	0.28	Neutral	0.00	Benign	0.53	Tolerated	10.91	Neutral
p.Ala143Tyr	VUS	-1.11	Neutral	0.2593	Benign	-5.92	Benign	0.39	Neutral	0.93	Possibly damaging	0.53	Tolerated		
p.Ala143Cys	VUS	-1.23	Neutral	0.2989	Benign	-5.09	Benign	0.39	Neutral	0.98	Probably damaging	0.16	Tolerated		
p.Ala143Trp	VUS	-1.68	Neutral	0.3151	Benign	-5.77	Benign	0.44	Neutral	0.98	Probably damaging	0.11	Tolerated		
p.Ala143Phe	VUS	-0.74	Neutral	0.2193	Benign	-5.72	Benign	0.40	Neutral	0.82	Possibly damaging	0.38	Tolerated		
p.Arg144Asn	VUS	-0.11	Neutral	0.2474	Benign	-4.32	Benign	0.31	Neutral	0.01	Benign	0.09	Tolerated		
p.Arg144Lys	VUS	-0.74	Neutral	0.1626	Benign	-4.96	Benign	0.20	Neutral	0.01	Benign	1	Tolerated		
p.Arg144Thr	VUS	-0.34	Neutral	0.1087	Benign	-3.81	Benign	0.26	Neutral	0.01	Benign	0.1	Tolerated		
p.Arg144Ser	VUS	-0.41	Neutral	0.1479	Benign	-3.10	Benign	0.17	Neutral	0.00	Benign	0.11	Tolerated	0.04	Neutral
p.Arg144Ile	VUS	-0.67	Neutral	0.1729	Benign	-4.85	Benign	0.23	Neutral	0.03	Benign	0.04	Deleterious		
p.Arg144Met	VUS	-0.35	Neutral	0.218	Benign	-5.62	Benign	0.31	Neutral	0.39	Benign	0.03	Deleterious		
p.Arg144His	VUS	-0.56	Neutral	0.0933	Benign	-4.43	Benign	0.11	Neutral	0.00	Benign	0.06	Tolerated	4.38	Neutral
p.Arg144Gln	VUS	-0.43	Neutral	0.0911	Benign	-4.18	Benign	0.17	Neutral	0.03	Benign	0.17	Tolerated		
p.Arg144Pro	VUS	-1.27	Neutral	0.0937	Benign	-3.26	Benign	0.24	Neutral	0.00	Benign	0.07	Tolerated	6.21	Neutral
p.Arg144Leu	VUS	-0.17	Neutral	0.1097	Benign	-4.07	Benign	0.19	Neutral	0.01	Benign	0.08	Tolerated	2.32	Neutral
p.Arg144Asp	VUS	-0.45	Neutral	0.1536	Benign	-2.90	Benign	0.29	Neutral	0.01	Benign	0.08	Tolerated		
p.Arg144Glu	VUS	-0.46	Neutral	0.127	Benign	-3.09	Benign	0.26	Neutral	0.01	Benign	0.66	Tolerated		
p.Arg144Ala	VUS	-0.38	Neutral	0.1021	Benign	-2.55	Benign	0.27	Neutral	0.00	Benign	0.12	Tolerated		
p.Arg144Gly	VUS	-0.84	Neutral	0.0753	Benign	-2.52	Benign	0.14	Neutral	0.00	Benign	0.07	Tolerated	0.14	Neutral
p.Arg144Val	VUS	-0.16	Neutral	0.1463	Benign	-3.69	Benign	0.25	Neutral	0.01	Benign	0.05	Deleterious		
p.Arg144Tyr	VUS	-0.32	Neutral	0.2413	Benign	-5.87	Benign	0.27	Neutral	0.02	Benign	0.03	Deleterious		
p.Arg144Cys	Likely benign	-0.03	Neutral	0.1551	Benign	-5.12	Benign	0.26	Neutral	0.00	Benign	0.01	Deleterious	4.63	Neutral
p.Arg144Trp	VUS	-0.28	Neutral	0.1153	Benign	-5.87	Benign	0.27	Neutral	0.71	Possibly damaging	0.01	Deleterious		
p.Arg144Phe	VUS	-0.11	Neutral	0.2614	Benign	-5.77	Benign	0.34	Neutral	0.03	Benign	0.02	Deleterious		
p.Ile145Asn	VUS	-0.68	Neutral	0.1287	Benign	-2.73	Benign	0.13	Neutral	0.03	Benign	0.13	Tolerated		
p.Ile145Lys	VUS	-0.74	Neutral	0.2074	Benign	-2.37	Benign	0.25	Neutral	0.02	Benign	0.16	Tolerated	9.74	Neutral
p.Ile145Thr	VUS	-1.03	Neutral	0.1185	Benign	-1.66	Benign	0.09	Neutral	0.00	Benign	0.2	Tolerated	5.59	Neutral
p.Ile145Arg	VUS	-1.68	Neutral	0.1187	Benign	-0.84	Benign	0.19	Neutral	0.05	Benign	0.12	Tolerated	8.58	Neutral
p.Ile145Ser	VUS	-0.64	Neutral	0.1266	Benign	-1.11	Benign	0.12	Neutral	0.00	Benign	0.29	Tolerated		
p.Ile145Met	VUS	-3.12	Neutral	0.0964	Benign	-3.42	Benign	0.13	Neutral	0.29	Benign	0.13	Tolerated	0.23	Neutral
p.Ile145His	VUS	-0.55	Neutral	0.2337	Benign	-2.10	Benign	0.20	Neutral	0.62	Possibly damaging	0.06	Tolerated		
p.Ile145Gln	VUS	-1.06	Neutral	0.1739	Benign	-1.49	Benign	0.19	Neutral	0.13	Benign	0.1	Tolerated		
p.Ile145Pro	VUS	-0.38	Neutral	0.1772	Benign	-0.58	Benign	0.21	Neutral	0.00	Benign	0.14	Tolerated		
p.Ile145Leu	VUS	-0.77	Neutral	0.1002	Benign	-1.63	Benign	0.22	Neutral	0.01	Benign	0.61	Tolerated	0.07	Neutral
p.Ile145Asp	VUS	-1.09	Neutral	0.2116	Benign	-0.72	Benign	0.30	Neutral	0.03	Benign	0.13	Tolerated		
p.Ile145Glu	VUS	-0.60	Neutral	0.1666	Benign	-0.54	Benign	0.30	Neutral	0.03	Benign	0.13	Tolerated		
p.Ile145Ala	VUS	-3.86	Neutral	0.1271	Benign	-0.29	Benign	0.18	Neutral	0.00	Benign	0.47	Tolerated		
p.Ile145Gly	VUS	-0.33	Neutral	0.2128	Benign	-0.19	Benign	0.17	Neutral	0.00	Benign	0.3	Tolerated		
p.Ile145Val	VUS	-2.24	Neutral	0.0996	Benign	-1.76	Benign	0.12	Neutral	0.00	Benign	0.97	Tolerated	0.00	Neutral
p.Ile145Tyr	VUS	-0.82	Neutral	0.2778	Benign	-4.33	Benign	0.30	Neutral	0.32	Benign	0.12	Tolerated		
p.Ile145Cys	VUS	-0.23	Neutral	0.4874	Ambiguous	-2.99	Benign	0.29	Neutral	0.35	Benign	0.07	Tolerated		
p.Ile145Trp	VUS	-1.01	Neutral	0.3666	Ambiguous	-3.77	Benign	0.32	Neutral	0.86	Possibly damaging	0.03	Deleterious		
p.Ile145Phe	VUS	-0.95	Neutral	0.1128	Benign	-3.99	Benign	0.25	Neutral	0.13	Benign	0.16	Tolerated		
p.Asp146Asn	VUS	-3.08	Neutral	0.148	Benign	-4.76	Benign	0.13	Neutral	0.00	Benign	0	Deleterious	6.95	Neutral
p.Asp146Lys	VUS	-0.48	Neutral	0.2876	Benign	-4.30	Benign	0.35	Neutral	0.03	Benign	0	Deleterious		
p.Asp146Thr	VUS	-2.33	Neutral	0.161	Benign	-3.26	Benign	0.23	Neutral	0.03	Benign	0	Deleterious		
p.Asp146Arg	VUS	-0.62	Neutral	0.1628	Benign	-2.67	Benign	0.23	Neutral	0.00	Benign	0	Deleterious		
p.Asp146Ser	VUS	-0.81	Neutral	0.1182	Benign	-2.77	Benign	0.25	Neutral	0.00	Benign	0	Deleterious		
p.Asp146Ile	VUS	-0.60	Neutral	0.2896	Benign	-4.82	Benign	0.34	Neutral	0.03	Benign	0	Deleterious		
p.Asp146Met	VUS	-1.10	Neutral	0.5411	Ambiguous	-5.12	Benign	0.38	Neutral	0.27	Benign	0	Deleterious		
p.Asp146His	VUS	-2.20	Neutral	0.1334	Benign	-4.22	Benign	0.28	Neutral	0.12	Benign	0	Deleterious	15.17	Deleterious
p.Asp146Gln	VUS	-2.14	Neutral	0.1883	Benign	-3.57	Benign	0.35	Neutral	0.08	Benign	0	Deleterious		
p.Asp146Pro	VUS	-1.91	Neutral	0.2096	Benign	-2.18	Benign	0.34	Neutral	0.27	Benign	0	Deleterious		
p.Asp146Leu	VUS	-1.92	Neutral	0.2342	Benign	-3.28	Benign	0.37	Neutral	0.03	Benign	0	Deleterious		
p.Asp146Glu	VUS	-0.72	Neutral	0.1395	Benign	-2.93	Benign	0.09	Neutral	0.00	Benign	0	Deleterious	0.64	Neutral
p.Asp146Ala	VUS	-1.04	Neutral	0.0971	Benign	-2.12	Benign	0.13	Neutral	0.00	Benign	0	Deleterious	9.20	Neutral
p.Asp146Gly	VUS	-0.79	Neutral	0.1138	Benign	-2.52	Benign	0.16	Neutral	0.00	Benign	0	Deleterious	11.57	Neutral
p.Asp146Val	VUS	-0.77	Neutral	0.1656	Benign	-3.61	Benign	0.30	Neutral	0.00	Benign	0	Deleterious	14.02	Neutral
p.Asp146Tyr	VUS	-3.39	Neutral	0.1614	Benign	-5.88	Benign	0.28	Neutral	0.00	Benign	0	Deleterious	11.95	Neutral

p.Asp146Cys	VUS	-3.78	Neutral	0.3974	Ambiguous	-4.71	Benign	0.37	Neutral	0.58	Possibly damaging	0	Deleterious		
p.Asp146Trp	VUS	-0.56	Neutral	0.5798	Pathogenic	-5.33	Benign	0.37	Neutral	0.58	Possibly damaging	0	Deleterious		
p.Asp146Phe	VUS	-1.81	Neutral	0.3732	Ambiguous	-5.37	Benign	0.35	Neutral	0.08	Benign	0	Deleterious		
p.Ala147Asn	VUS	-0.93	Neutral	0.2019	Benign	-5.67	Benign	0.38	Neutral	0.45	Benign	0.17	Tolerated		
p.Ala147Lys	VUS	-1.03	Neutral	0.2553	Benign	-5.44	Benign	0.39	Neutral	0.19	Benign	0.18	Tolerated		
p.Ala147Thr	VUS	-0.92	Neutral	0.0804	Benign	-4.49	Benign	0.16	Neutral	0.08	Benign	0.91	Tolerated	15.28	Deleterious
p.Ala147Arg	VUS	-0.26	Neutral	0.1538	Benign	-4.27	Benign	0.29	Neutral	0.45	Benign	0.12	Tolerated		
p.Ala147Ser	VUS	-1.41	Neutral	0.0998	Benign	-4.21	Benign	0.15	Neutral	0.00	Benign	0.7	Tolerated	7.95	Neutral
p.Ala147Ile	VUS	-0.68	Neutral	0.1893	Benign	-5.47	Benign	0.38	Neutral	0.10	Benign	0.3	Tolerated		
p.Ala147Met	VUS	-1.38	Neutral	0.2114	Benign	-6.10	Benign	0.36	Neutral	0.62	Possibly damaging	0.1	Tolerated		
p.Ala147His	VUS	-0.23	Neutral	0.2085	Benign	-5.71	Benign	0.29	Neutral	0.95	Possibly damaging	0.06	Tolerated		
p.Ala147Gln	VUS	-5.59	Neutral	0.1828	Benign	-5.29	Benign	0.38	Neutral	0.45	Benign	0.13	Tolerated		
p.Ala147Pro	VUS	-1.86	Neutral	0.0928	Benign	-3.80	Benign	0.21	Neutral	0.00	Benign	0.24	Tolerated	12.40	Neutral
p.Ala147Leu	VUS	-1.49	Neutral	0.1278	Benign	-4.42	Benign	0.37	Neutral	0.05	Benign	0.27	Tolerated		
p.Ala147Asp	VUS	-2.43	Neutral	0.1068	Benign	-4.04	Benign	0.25	Neutral	0.15	Benign	0.13	Tolerated	14.09	Neutral
p.Ala147Glu	VUS	-0.24	Neutral	0.1148	Benign	-3.75	Benign	0.35	Neutral	0.19	Benign	0.16	Tolerated		
p.Ala147Gly	VUS	-0.97	Neutral	0.09	Benign	-3.42	Benign	0.18	Neutral	0.00	Benign	0.34	Tolerated	9.12	Neutral
p.Ala147Val	VUS	-1.68	Neutral	0.1016	Benign	-4.38	Benign	0.17	Neutral	0.00	Benign	0.64	Tolerated	9.43	Neutral
p.Ala147Tyr	VUS	-0.40	Neutral	0.2901	Benign	-6.98	Benign	0.31	Neutral	0.83	Possibly damaging	0.08	Tolerated		
p.Ala147Cys	VUS	-0.81	Neutral	0.3345	Benign	-6.15	Benign	0.27	Neutral	0.95	Possibly damaging	0.23	Tolerated		
p.Ala147Trp	VUS	-0.55	Neutral	0.3676	Ambiguous	-6.70	Benign	0.40	Neutral	0.95	Possibly damaging	0.03	Deleterious		
p.Ala147Phe	VUS	-0.22	Neutral	0.2218	Benign	-6.35	Benign	0.39	Neutral	0.62	Possibly damaging	0.09	Tolerated		
p.Ala148Asn	VUS	-6.43	Indeterminate	0.2079	Benign	-5.63	Benign	0.46	Neutral	0.77	Possibly damaging	0	Deleterious		
p.Ala148Lys	VUS	-26.41	Indeterminate	0.2504	Benign	-5.49	Benign	0.48	Neutral	0.33	Benign	0	Deleterious		
p.Ala148Thr	Likely benign	-1.32	Neutral	0.0801	Benign	-4.63	Benign	0.12	Neutral	0.80	Possibly damaging	0	Deleterious	15.70	Deleterious
p.Ala148Arg	VUS	-8.95	Indeterminate	0.1519	Benign	-4.24	Benign	0.36	Neutral	0.63	Possibly damaging	0	Deleterious		
p.Ala148Ser	VUS	-2.30	Neutral	0.0873	Benign	-4.09	Benign	0.17	Neutral	0.07	Benign	0	Deleterious	14.77	Neutral
p.Ala148Ile	VUS	-3.57	Neutral	0.2128	Benign	-5.17	Benign	0.46	Neutral	0.63	Possibly damaging	0	Deleterious		
p.Ala148Met	VUS	-12.46	Indeterminate	0.219	Benign	-5.53	Benign	0.46	Neutral	0.96	Probably damaging	0	Deleterious		
p.Ala148His	VUS	-6.12	Indeterminate	0.2355	Benign	-5.65	Benign	0.45	Neutral	0.96	Probably damaging	0	Deleterious		
p.Ala148Gln	VUS	-9.43	Indeterminate	0.1675	Benign	-4.87	Benign	0.31	Neutral	0.03	Benign	0	Deleterious		
p.Ala148Pro	VUS	-4.20	Neutral	0.0883	Benign	-3.89	Benign	0.48	Neutral	0.01	Benign	0	Deleterious	15.93	Deleterious
p.Ala148Leu	VUS	-4.52	Neutral	0.1365	Benign	-4.13	Benign	0.47	Neutral	0.33	Benign	0	Deleterious		
p.Ala148Asp	VUS	-3.31	Neutral	0.1202	Benign	-3.97	Benign	0.48	Neutral	0.33	Benign	0	Deleterious		
p.Ala148Glu	VUS	-7.75	Indeterminate	0.1156	Benign	-3.57	Benign	0.26	Neutral	0.01	Benign	0	Deleterious	13.03	Neutral
p.Ala148Gly	VUS	-1.38	Neutral	0.0837	Benign	-3.48	Benign	0.22	Neutral	0.01	Benign	0	Deleterious	10.10	Neutral
p.Ala148Val	VUS	-10.84	Indeterminate	0.1065	Benign	-4.20	Benign	0.16	Neutral	0.03	Benign	0	Deleterious	9.76	Neutral
p.Ala148Tyr	VUS	-0.81	Neutral	0.3042	Benign	-6.69	Benign	0.47	Neutral	0.87	Possibly damaging	0	Deleterious		
p.Ala148Cys	VUS	-0.04	Neutral	0.3346	Benign	-5.91	Benign	0.41	Neutral	0.99	Probably damaging	0	Deleterious		
p.Ala148Trp	VUS	-23.29	Indeterminate	0.3527	Ambiguous	-6.33	Benign	0.51	Disease	0.99	Probably damaging	0	Deleterious		
p.Ala148Phe	VUS	-24.04	Indeterminate	0.2336	Benign	-6.05	Benign	0.49	Neutral	0.77	Possibly damaging	0	Deleterious		
p.Glu149Asn	VUS	-0.21	Neutral	0.2363	Benign	-4.72	Benign	0.38	Neutral	0.12	Benign	0.08	Tolerated		
p.Glu149Lys	VUS	-1.24	Neutral	0.1483	Benign	-5.09	Benign	0.35	Neutral	0.04	Benign	0.12	Tolerated	16.13	Deleterious
p.Glu149Thr	VUS	-3.47	Neutral	0.1487	Benign	-3.51	Benign	0.39	Neutral	0.05	Benign	0.06	Tolerated		
p.Glu149Arg	VUS	-0.32	Neutral	0.1196	Benign	-3.11	Benign	0.37	Neutral	0.01	Benign	0.04	Deleterious		
p.Glu149Ser	VUS	-5.62	Neutral	0.1372	Benign	-3.06	Benign	0.39	Neutral	0.03	Benign	0.08	Tolerated		
p.Glu149Ile	VUS	-3.18	Neutral	0.3233	Benign	-5.12	Benign	0.46	Neutral	0.00	Benign	0.02	Deleterious		
p.Glu149Met	VUS	-0.48	Neutral	0.4196	Ambiguous	-5.39	Benign	0.34	Neutral	0.01	Benign	0.01	Deleterious		
p.Glu149His	VUS	-1.17	Neutral	0.215	Benign	-4.58	Benign	0.25	Neutral	0.74	Possibly damaging	0.03	Deleterious		
p.Glu149Gln	VUS	-0.22	Neutral	0.1027	Benign	-4.20	Benign	0.15	Neutral	0.17	Benign	0.13	Tolerated	10.92	Neutral
p.Glu149Pro	VUS	-0.16	Neutral	0.1886	Benign	-2.71	Benign	0.43	Neutral	0.00	Benign	0.06	Tolerated		
p.Glu149Leu	VUS	-0.12	Neutral	0.258	Benign	-3.72	Benign	0.45	Neutral	0.01	Benign	0.03	Deleterious		
p.Glu149Asp	VUS	-1.68	Neutral	0.1	Benign	-3.21	Benign	0.25	Neutral	0.04	Benign	0.76	Tolerated	6.26	Neutral
p.Glu149Ala	VUS	-2.07	Neutral	0.0853	Benign	-2.16	Benign	0.23	Neutral	0.00	Benign	0.11	Tolerated	11.61	Neutral
p.Glu149Gly	VUS	-1.58	Neutral	0.0873	Benign	-2.74	Benign	0.19	Neutral	0.00	Benign	0.06	Tolerated	12.78	Neutral
p.Glu149Val	VUS	-1.34	Neutral	0.1769	Benign	-3.95	Benign	0.42	Neutral	0.00	Benign	0.03	Deleterious	16.59	Deleterious
p.Glu149Tyr	VUS	-1.10	Neutral	0.3748	Ambiguous	-5.81	Benign	0.43	Neutral	0.74	Possibly damaging	0.01	Deleterious		
p.Glu149Cys	VUS	-1.40	Neutral	0.5637	Ambiguous	-4.96	Benign	0.33	Neutral	0.91	Possibly damaging	0	Deleterious		
p.Glu149Trp	VUS	-1.72	Neutral	0.5825	Pathogenic	-6.09	Benign	0.46	Neutral	0.91	Possibly damaging	0	Deleterious		
p.Glu149Phe	VUS	-4.67	Neutral	0.4663	Ambiguous	-5.34	Benign	0.46	Neutral	0.31	Benign	0.01	Deleterious		
p.Gly150Asn	VUS	-0.22	Neutral	0.1638	Benign	-5.06	Benign	0.39	Neutral	0.02	Benign	0.41	Tolerated		
p.Gly150Lys	VUS	-2.08	Neutral	0.2131	Benign	-5.12	Benign	0.41	Neutral	0.51	Possibly damaging	1	Tolerated		
p.Gly150Thr	VUS	-1.15	Neutral	0.0936	Benign	-4.15	Benign	0.27	Neutral	0.51	Possibly damaging	0.27	Tolerated		
p.Gly150Arg	VUS	-0.34	Neutral	0.1683	Benign	-3.78	Benign	0.41	Neutral	0.03	Benign	0.47	Tolerated	14.19	Neutral
p.Gly150Ser	VUS	-1.31	Neutral	0.0985	Benign	-3.67	Benign	0.14	Neutral	0.05	Benign	0.42	Tolerated	10.05	Neutral
p.Gly150Ile	VUS	-0.16	Neutral	0.1346	Benign	-4.58	Benign	0.44	Neutral	0.94	Possibly damaging	0.09	Tolerated		
p.Gly150Met	VUS	-0.78	Neutral	0.231	Benign	-5.39	Benign	0.45	Neutral	0.99	Probably damaging	0.06	Tolerated		
p.Gly150His	VUS	-1.35	Neutral	0.1691	Benign	-5.13	Benign	0.32	Neutral	0.98	Probably damaging	0.14	Tolerated		
p.Gly150Gln	VUS	-1.46	Neutral	0.1539	Benign	-4.40	Benign	0.38	Neutral	0.88	Possibly damaging	0.32	Tolerated		
p.Gly150Pro	VUS	-0.78	Neutral	0.2298	Benign	-3.31	Benign	0.39	Neutral	0.94	Possibly damaging	0.19	Tolerated		
p.Gly150Leu	VUS	-0.48	Neutral	0.1639	Benign	-3.85	Benign	0.30	Neutral	0.88	Possibly damaging	0.17	Tolerated		
p.Gly150Asp	VUS	-1.76	Neutral	0.0881	Benign	-3.69	Benign	0.29	Neutral	0.01	Benign	0.39	Tolerated	10.58	Neutral
p.Gly150Glu	VUS	-0.18	Neutral	0.084	Benign	-3.41	Benign	0.37	Neutral	0.51	Possibly damaging	0.42	Tolerated		
p.Gly150Ala	VUS	-0.14	Neutral	0.0866	Benign	-3.32	Benign	0.13	Neutral	0.06	Benign	0.47	Tolerated	8.81	Neutral
p.Gly150Val	VUS	-0.76	Neutral	0.0977	Benign	-3.95	Benign	0.29	Neutral	0.85	Possibly damaging	0.14	Tolerated	17.64	Deleterious
p.Gly150Tyr	VUS	-0.13	Neutral	0.1895	Benign	-6.43	Benign	0.44	Neutral	0.98	Probably damaging	0.09	Tolerated		
p.Gly150Cys	VUS	-0.02	Neutral	0.1781	Benign	-5.86	Benign	0.43	Neutral	0.99	Probably damaging	0.05	Deleterious	15.99	Deleterious
p.Gly150Trp	VUS	-0.49	Neutral	0.1743	Benign	-6.25	Benign	0.44	Neutral	0.99	Probably damaging	0.03	Deleterious		
p.Gly150Phe	VUS	-0.87	Neutral	0.2684	Benign	-5.52	Benign	0.44	Neutral	0.98	Probably damaging	0.06	Tolerated		
p.Pro151Asn	VUS	-0.72	Neutral	0.1899	Benign	-4.86	Benign	0.44	Neutral	0.77	Possibly damaging	0.41	Tolerated		
p.Pro151Lys	VUS	-0.10	Neutral	0.1679	Benign	-5.17	Benign	0.48	Neutral	0.49	Possibly damaging	0.45	Tolerated		
p.Pro151Thr	VUS	-1.96	Neutral	0.0763	Benign	-3.99	Benign	0.20	Neutral	0.00	Benign	0.46	Tolerated	9.37	Neutral
p.Pro151Arg	VUS	-0.63	Neutral	0.0798	Benign	-3.63	Benign	0.29	Neutral	0.72	Possibly damaging	0.42	Tolerated	14.93	Neutral
p.Pro151Ser	VUS	-15.51	Indeterminate	0.0798	Benign	-3.16	Benign	0.24	Neutral	0.01	Benign	0.54	Tolerated	9.05	Neutral
p.Pro151Ile	VUS	-0.49	Neutral	0.1737	Benign	-4.99	Benign	0.51	Disease	0.63	Possibly damaging	0.37	Tolerated		
p.Pro151Met	VUS	-0.07	Neutral	0.2229	Benign	-5.77	Benign	0.49	Neutral	0.96	Probably damaging	0.2	Tolerated		
p.Pro151His	VUS	-0.88	Neutral	0.0925	Benign	-4.94	Benign	0.28	Neutral	0.01	Benign	0.42	Tolerated	14.78	Neutral
p.Pro151Gln	VUS	-0.19	Neutral	0.0882	Benign	-4.84	Benign	0.41	Neutral	0.77	Possibly damaging	0.33	Tolerated		
p.Pro151Leu	VUS	-3.56	Neutral	0.1004	Benign	-4.02	Benign	0.26	Neutral	0.27	Benign	0.64	Tolerated	15.41	Deleterious
p.Pro151Asp	VUS	-2.62	Neutral	0.1412	Benign	-3.26	Benign	0.48	Neutral	0.49	Possibly damaging	0.36	Tolerated		
p.Pro151Glu	VUS	-0.84	Neutral	0.1103	Benign	-3.74	Benign	0.44	Neutral	0.49	Possibly damaging	0.4	Tolerated		
p.Pro151Ala	VUS	-2.27	Neutral	0.063	Benign	-3.15	Benign	0.32	Neutral	0.13	Benign	0.67	Tolerated	11.33	Neutral
p.Pro151Gly	VUS	-8.60	Indeterminate	0.1164	Benign	-2.83	Benign	0.44	Neutral	0.33	Benign	0.47	Tolerated		
p.Pro151Val	VUS	-0.18	Neutral	0.1199	Benign	-4.25	Benign	0.31	Neutral	0.03	Benign	0.48	Tolerated		
p.Pro151Tyr	VUS	-0.04	Neutral	0.204	Benign	-5.61	Benign	0.49	Neutral	0.77	Possibly damaging	1	Tolerated		
p.Pro151Cys	VUS	-0.48	Neutral	0.2543	Benign	-5.01	Benign	0.49	Neutral	0.99	Probably damaging	0.16	Tolerated		
p.Pro151Trp	VUS	-0.23	Neutral	0.2269	Benign	-5.83	Benign	0.51	Disease	0.99	Probably damaging	0.21	Tolerated		
p.Pro151Phe	VUS	-0.37	Neutral	0.1909	Benign	-5.15	Benign	0.51	Disease	0.87	Possibly damaging	0.84	Tolerated		
p.Ser152Asn	VUS	-3.22	Neutral	0.1709	Benign	-4.88	Benign	0.21	Neutral	0.19	Benign	0.46	Tolerated		
p.Ser152Lys	VUS	-2.25	Neutral	0.3202	Benign	-4.52	Benign	0.31	Neutral	0.09	Benign	0.28	Tolerated		
p.Ser152Thr	VUS	-2.54	Neutral	0.093	Benign	-3.89	Benign	0.11	Neutral	0.00	Benign	0.38	Tolerated	3.25	Neutral
p.Ser152Arg	VUS	-1.68	Neutral	0.204	Benign	-3.04	Benign	0.29	Neutral	0.19	Benign	0.18	Tolerated		
p.Ser152Ile	VUS	-0.47	Neutral	0.1923	Benign	-4.47	Benign	0.33	Neutral	0.19	Benign	0.08	Tolerated		
p.Ser152Met	VUS	-3.57	Neutral	0.2632	Benign	-4.60	Benign	0.36	Neutral	0.83	Possibly damaging	0.06	Tolerated		
p.Ser152His	VUS	-0.63	Neutral	0.1793	Benign	-4.84	Benign	0.34	Neutral	0.83	Possibly damaging	0.11	Tolerated		
p.Ser152Gln	VUS	-0.32	Neutral	0.219	Benign	-3.80	Benign	0.30	Neutral	0.32	Benign	0.19	Tolerated		
p.Ser152Pro	VUS	-2.64	Neutral	0.0892	Benign	-3.07	Benign	0.15	Neutral	0.00	Benign	0.29	Tolerated	0.77	Neutral
p.Ser152Leu	VUS	-3.01	Neutral	0.1385	Benign	-3.57	Benign	0.23	Neutral	0.04	Benign	0.15	Tolerated	21.40	Deleterious
p.Ser152Asp	VUS	-0.38	Neutral	0.1717	Benign	-3.02	Benign	0.30	Neutral	0.19	Benign	0.43	Tolerated		
p.Ser152Glu	VUS	-1.44	Neutral	0.1777	Benign	-2.63	Benign	0.29	Neutral	0.09	Benign	0.28	Tolerated		
p.Ser152Ala	VUS	-3.53	Neutral	0.0696	Benign	-2.69	Benign	0.10	Neutral	0.00	Benign	1	Tolerated	0.00	Neutral

p.Ser152Gly	VUS	-4.94	Neutral	0.0752	Benign	-2.64	Benign	0.15	Neutral	0.05	Benign	0.79	Tolerated		
p.Ser152Val	VUS	-0.74	Neutral	0.1548	Benign	-3.64	Benign	0.25	Neutral	0.05	Benign	0.19	Tolerated		
p.Ser152Tyr	VUS	-6.00	Indeterminate	0.1686	Benign	-5.83	Benign	0.35	Neutral	0.83	Possibly damaging	0.09	Tolerated		
p.Ser152Cys	VUS	-0.14	Neutral	0.1286	Benign	-5.27	Benign	0.32	Neutral	0.62	Possibly damaging	0.11	Tolerated		
p.Ser152Trp	VUS	-0.77	Neutral	0.1637	Benign	-5.34	Benign	0.40	Neutral	0.95	Possibly damaging	0.03	Deleterious		
p.Ser152Phe	VUS	-0.82	Neutral	0.2066	Benign	-5.34	Benign	0.35	Neutral	0.62	Possibly damaging	0.07	Tolerated		
p.Asp153Asn	VUS	-3.03	Neutral	0.1258	Benign	-4.57	Benign	0.22	Neutral	0.08	Benign	1	Tolerated	25.20	Deleterious
p.Asp153Lys	VUS	-1.73	Neutral	0.2406	Benign	-4.74	Benign	0.29	Neutral	0.05	Benign	0.13	Tolerated		
p.Asp153Thr	VUS	-5.10	Neutral	0.1456	Benign	-3.57	Benign	0.29	Neutral	0.10	Benign	0.1	Tolerated		
p.Asp153Arg	VUS	-4.54	Neutral	0.1458	Benign	-3.09	Benign	0.28	Neutral	0.29	Benign	0.06	Tolerated		
p.Asp153Ser	VUS	0.00	Neutral	0.0995	Benign	-2.91	Benign	0.28	Neutral	0.05	Benign	0.2	Tolerated		
p.Asp153Ile	VUS	-0.70	Neutral	0.2505	Benign	-4.40	Benign	0.30	Neutral	0.29	Benign	0.01	Deleterious		
p.Asp153Met	VUS	-1.49	Neutral	0.4459	Ambiguous	-5.20	Benign	0.31	Neutral	0.90	Possibly damaging	0.01	Deleterious		
p.Asp153His	VUS	-2.40	Neutral	0.1339	Benign	-4.29	Benign	0.19	Neutral	0.88	Possibly damaging	0.07	Tolerated	24.40	Deleterious
p.Asp153Gln	VUS	-2.53	Neutral	0.1533	Benign	-4.09	Benign	0.27	Neutral	0.29	Benign	0.09	Tolerated		
p.Asp153Pro	VUS	0.00	Neutral	0.2242	Benign	-2.92	Benign	0.31	Neutral	0.45	Benign	0.06	Tolerated		
p.Asp153Leu	VUS	-1.93	Neutral	0.217	Benign	-3.61	Benign	0.32	Neutral	0.17	Benign	0.03	Deleterious		
p.Asp153Glu	VUS	-0.72	Neutral	0.11	Benign	-3.39	Benign	0.07	Neutral	0.00	Benign	0.17	Tolerated	0.30	Neutral
p.Asp153Ala	VUS	-0.06	Neutral	0.0941	Benign	-2.90	Benign	0.19	Neutral	0.02	Benign	0.1	Tolerated	3.18	Neutral
p.Asp153Gly	VUS	0.00	Neutral	0.0907	Benign	-2.74	Benign	0.13	Neutral	0.00	Benign	0.24	Tolerated	1.87	Neutral
p.Asp153Val	VUS	-3.27	Neutral	0.1424	Benign	-3.92	Benign	0.27	Neutral	0.13	Benign	0.02	Deleterious	6.46	Neutral
p.Asp153Tyr	VUS	-3.98	Neutral	0.1422	Benign	-4.27	Benign	0.28	Neutral	0.65	Possibly damaging	0.04	Deleterious	49.00	Deleterious
p.Asp153Cys	VUS	-2.76	Neutral	0.3314	Benign	-4.38	Benign	0.31	Neutral	0.00	Benign	0.02	Deleterious		
p.Asp153Trp	VUS	-2.73	Neutral	0.4708	Ambiguous	-5.07	Benign	0.36	Neutral	0.90	Possibly damaging	0.01	Deleterious		
p.Asp153Phe	VUS	-0.54	Neutral	0.3314	Benign	-4.49	Benign	0.31	Neutral	0.45	Benign	0.02	Deleterious		
p.Ile154Asn	VUS	-4.12	Neutral	0.1052	Benign	-3.50	Benign	0.10	Neutral	0.00	Benign	0.1	Tolerated	4.16	Neutral
p.Ile154Lys	VUS	-0.82	Neutral	0.243	Benign	-3.21	Benign	0.19	Neutral	0.00	Benign	0.13	Tolerated		
p.Ile154Thr	VUS	-4.01	Neutral	0.20	Benign	-2.18	Benign	0.05	Neutral	0.00	Benign	0.79	Tolerated	6.37	Neutral
p.Ile154Arg	VUS	-1.80	Neutral	0.1514	Benign	-1.74	Benign	0.18	Neutral	0.00	Benign	0.09	Tolerated		
p.Ile154Ser	VUS	-1.22	Neutral	0.12	Benign	-1.48	Benign	0.08	Neutral	0.00	Benign	0.29	Tolerated	7.22	Neutral
p.Ile154Met	VUS	-2.85	Neutral	0.1331	Benign	-3.99	Benign	0.08	Neutral	0.03	Benign	0.12	Tolerated	6.97	Neutral
p.Ile154His	VUS	-7.49	Indeterminate	0.1902	Benign	-3.01	Benign	0.21	Neutral	0.20	Benign	0.05	Deleterious		
p.Ile154Gln	VUS	-2.71	Neutral	0.1991	Benign	-2.54	Benign	0.23	Neutral	0.01	Benign	0.09	Tolerated		
p.Ile154Pro	VUS	-11.15	Indeterminate	0.2623	Benign	-1.45	Benign	0.23	Neutral	0.03	Benign	0.11	Tolerated		
p.Ile154Leu	VUS	-1.30	Neutral	0.0907	Benign	-2.13	Benign	0.13	Neutral	0.00	Benign	0.48	Tolerated	0.60	Neutral
p.Ile154Asp	VUS	-1.80	Neutral	0.1529	Benign	-1.86	Benign	0.17	Neutral	0.00	Benign	0.08	Tolerated		
p.Ile154Glu	VUS	-2.16	Neutral	0.1869	Benign	-1.53	Benign	0.26	Neutral	0.00	Benign	0.11	Tolerated		
p.Ile154Ala	VUS	-11.26	Indeterminate	0.1848	Benign	-1.19	Benign	0.18	Neutral	0.00	Benign	0.37	Tolerated		
p.Ile154Gly	VUS	-0.86	Neutral	0.2514	Benign	-0.95	Benign	0.14	Neutral	0.00	Benign	0.07	Tolerated		
p.Ile154Val	VUS	-5.37	Neutral	0.12	Benign	-2.12	Benign	0.05	Neutral	0.00	Benign	0.94	Tolerated	0.70	Neutral
p.Ile154Tyr	VUS	-6.33	Indeterminate	0.2394	Benign	-4.89	Benign	0.18	Neutral	0.01	Benign	0.08	Tolerated		
p.Ile154Cys	VUS	-7.96	Indeterminate	0.457	Ambiguous	-3.47	Benign	0.18	Neutral	0.20	Benign	0.07	Tolerated		
p.Ile154Trp	VUS	-5.04	Neutral	0.4093	Ambiguous	-4.07	Benign	0.20	Neutral	0.43	Benign	0.02	Deleterious		
p.Ile154Phe	VUS	-2.57	Neutral	0.102	Benign	-3.93	Benign	0.09	Neutral	0.00	Benign	0.11	Tolerated	0.84	Neutral
p.Pro155Asn	VUS	-1.38	Neutral	0.245	Benign	-4.51	Benign	0.24	Neutral	0.77	Possibly damaging	0	Deleterious		
p.Pro155Lys	VUS	-4.81	Neutral	0.2015	Benign	-3.82	Benign	0.21	Neutral	0.49	Possibly damaging	0	Deleterious		
p.Pro155Thr	VUS	0.00	Neutral	0.075	Benign	-3.48	Benign	0.14	Neutral	0.27	Benign	0	Deleterious	5.66	Neutral
p.Pro155Arg	VUS	-0.34	Neutral	0.0812	Benign	-3.02	Benign	0.16	Neutral	0.72	Possibly damaging	0	Deleterious	5.27	Neutral
p.Pro155Ser	VUS	-28.22	Indeterminate	0.0838	Benign	-2.51	Benign	0.08	Neutral	0.01	Benign	0	Deleterious	6.39	Neutral
p.Pro155Ile	VUS	-7.35	Indeterminate	0.2085	Benign	-3.53	Benign	0.26	Neutral	0.33	Benign	0	Deleterious		
p.Pro155Met	VUS	0.00	Neutral	0.2982	Benign	-4.40	Benign	0.23	Neutral	0.77	Possibly damaging	0	Deleterious		
p.Pro155His	VUS	-10.71	Indeterminate	0.123	Benign	-4.21	Benign	0.19	Neutral	0.98	Probably damaging	0	Deleterious	5.43	Neutral
p.Pro155Gln	VUS	0.00	Neutral	0.0963	Benign	-3.33	Benign	0.15	Neutral	0.87	Possibly damaging	0	Deleterious		
p.Pro155Leu	VUS	0.00	Neutral	0.125	Benign	-3.06	Benign	0.18	Neutral	0.00	Benign	0	Deleterious	6.17	Neutral
p.Pro155Asp	VUS	-32.02	Indeterminate	0.1623	Benign	-2.61	Benign	0.26	Neutral	0.49	Possibly damaging	0	Deleterious		
p.Pro155Glu	VUS	0.00	Neutral	0.1133	Benign	-2.35	Benign	0.24	Neutral	0.49	Possibly damaging	0	Deleterious		
p.Pro155Ala	VUS	0.00	Neutral	0.0586	Benign	-2.99	Benign	0.14	Neutral	0.00	Benign	0	Deleterious	5.51	Neutral
p.Pro155Gly	VUS	-0.04	Neutral	0.1214	Benign	-2.95	Benign	0.18	Neutral	0.33	Benign	0	Deleterious		
p.Pro155Val	VUS	-21.58	Indeterminate	0.138	Benign	-3.09	Benign	0.22	Neutral	0.33	Benign	0	Deleterious		
p.Pro155Tyr	VUS	-0.02	Neutral	0.2893	Benign	-5.04	Benign	0.25	Neutral	0.95	Possibly damaging	0	Deleterious		
p.Pro155Cys	VUS	0.00	Neutral	0.3012	Benign	-4.75	Benign	0.21	Neutral	0.96	Probably damaging	0	Deleterious		
p.Pro155Trp	VUS	-13.71	Indeterminate	0.3032	Benign	-4.95	Benign	0.27	Neutral	0.99	Probably damaging	0	Deleterious		
p.Pro155Phe	VUS	0.00	Neutral	0.3021	Benign	-4.46	Benign	0.25	Neutral	0.77	Possibly damaging	0	Deleterious		
p.Asp156Asn	VUS	-3.19	Neutral	0.13	Benign	-4.37	Benign	0.05	Neutral	0.00	Benign	0	Deleterious	10.02	Neutral
p.Asp156Lys	VUS	-7.54	Indeterminate	0.2314	Benign	-4.42	Benign	0.19	Neutral	0.21	Benign	0	Deleterious		
p.Asp156Thr	VUS	-1.63	Neutral	0.1663	Benign	-3.45	Benign	0.20	Neutral	0.34	Benign	0	Deleterious		
p.Asp156Arg	VUS	-4.36	Neutral	0.1677	Benign	-2.84	Benign	0.18	Neutral	0.65	Possibly damaging	0	Deleterious		
p.Asp156Ser	VUS	-0.77	Neutral	0.0915	Benign	-2.61	Benign	0.19	Neutral	0.10	Benign	0	Deleterious		
p.Asp156Ile	VUS	-2.46	Neutral	0.3162	Benign	-4.16	Benign	0.23	Neutral	0.65	Possibly damaging	0	Deleterious		
p.Asp156Met	VUS	-1.90	Neutral	0.5632	Ambiguous	-4.77	Benign	0.24	Neutral	0.98	Probably damaging	0	Deleterious		
p.Asp156His	VUS	-4.44	Neutral	0.1454	Benign	-4.09	Benign	0.10	Neutral	0.88	Possibly damaging	0	Deleterious	9.67	Neutral
p.Asp156Gln	VUS	-6.15	Indeterminate	0.1475	Benign	-3.65	Benign	0.17	Neutral	0.48	Possibly damaging	0	Deleterious		
p.Asp156Pro	VUS	-0.96	Neutral	0.2106	Benign	-2.74	Benign	0.22	Neutral	0.79	Possibly damaging	0	Deleterious		
p.Asp156Leu	VUS	-3.31	Neutral	0.2758	Benign	-2.83	Benign	0.25	Neutral	0.21	Benign	0	Deleterious		
p.Asp156Glu	VUS	-1.87	Neutral	0.11	Benign	-3.22	Benign	0.06	Neutral	0.00	Benign	0	Deleterious	4.93	Neutral
p.Asp156Ala	VUS	-9.22	Indeterminate	0.10	Benign	-2.29	Benign	0.06	Neutral	0.00	Benign	0	Deleterious	7.78	Neutral
p.Asp156Gly	VUS	-3.71	Neutral	0.12	Benign	-2.67	Benign	0.07	Neutral	0.08	Benign	0	Deleterious	8.04	Neutral
p.Asp156Val	VUS	-3.79	Neutral	0.188	Benign	-3.23	Benign	0.17	Neutral	0.16	Benign	0	Deleterious	8.63	Neutral
p.Asp156Tyr	VUS	-6.31	Indeterminate	0.1729	Benign	-4.03	Benign	0.18	Neutral	0.96	Possibly damaging	0	Deleterious	11.12	Neutral
p.Asp156Cys	VUS	-6.69	Indeterminate	0.3819	Ambiguous	-3.93	Benign	0.23	Neutral	0.93	Possibly damaging	0	Deleterious		
p.Asp156Trp	VUS	-1.51	Neutral	0.6033	Pathogenic	-4.70	Benign	0.26	Neutral	0.98	Probably damaging	0	Deleterious		
p.Asp156Phe	VUS	-1.31	Neutral	0.4633	Ambiguous	-3.52	Benign	0.22	Neutral	0.79	Possibly damaging	0	Deleterious		
