# Supplementary material for: Functional characterization of all CDKN2A missense variants and comparison to in silico models of pathogenicity
Source: bioRxiv. 2025 Feb 11:2023.12.28.573507. Originally published 2023 Dec 28. Preprint. [Version 3] doi: 10.1101/2023.12.28.573507 (PMC10793438; doi:10.1101/2023.12.28.573507)
Supplement: Supplement 8 [file media-8.pdf]

Appendix 1-table 8. Assessment of in silico variant effect prediction models.

In silico model	Accuracy (%)	Sensitivity	Specificity	Positive predictive value	Negative predictive value
CADD	45.1	0.97	0.35	0.23	0.98
Polyphen-2	39.5	0.98	0.27	0.22	0.98
SIFT	60.9	0.79	0.57	0.28	0.93
VEST	71.9	0.91	0.68	0.38	0.97
AlphaMissense	71.6	0.94	0.67	0.38	0.98
ESM1b	59.2	0.95	0.51	0.30	0.98
PrimateAI-3D	85.4	0.25	0.98	0.68	0.87
