# Supplementary material for: Functional characterization of all CDKN2A missense variants and comparison to in silico models of pathogenicity
Source: bioRxiv. 2025 Feb 11:2023.12.28.573507. Originally published 2023 Dec 28. Preprint. [Version 3] doi: 10.1101/2023.12.28.573507 (PMC10793438; doi:10.1101/2023.12.28.573507)
Supplement: Supplement 9 [file media-9.pdf]

Appendix 1-table 9. Missense somatic mutations in *CDKN2A* reported in COSMIC, TCGA, JHU, MSK-IMPACT.

CDKN2A amino acid residue	Variant	Number of patietns with mutation reported				Characteritic
		COSMIC	TCGA	JHU	MSK-IMPACT	
2	p.Glu2Lys	3	-	-	1	Neutral
4	p.Ala4Ser	1	-	-	-	Deleterious
5	p.Ala5Thr	2	-	-	-	Neutral
6	p.Gly6Glu	2	-	-	-	Neutral
7	p.Ser7Arg	1	-	-	-	Neutral
8	p.Ser8Ile	1	1	-	-	Neutral
8	p.Ser8Arg	1	1	-	-	Neutral
9	p.Met9Ile	2	-	-	-	Neutral
10	p.Glu10Val	1	-	-	1	Neutral
12	p.Ser12Leu	5	1	-	3	Indeterminate
13	p.Ala13Thr	1	1	-	-	Neutral
14	p.Asp14Glu	3	-	-	1	Neutral
14	p.Asp14Gly	2	-	-	-	Neutral
14	p.Asp14Asn	2	-	-	1	Neutral
14	p.Asp14Val	1	-	-	-	Indeterminate
16	p.Leu16Pro	4	-	-	-	Deleterious
16	p.Leu16Gln	1	1	-	-	Deleterious
16	p.Leu16Arg	1	1	-	-	Deleterious
17	p.Ala17Gly	1	-	-	-	Neutral
18	p.Thr18Ala	1	-	-	-	Indeterminate
18	p.Thr18Met	1	-	-	-	Indeterminate
19	p.Ala19Pro	1	-	-	-	Deleterious
20	p.Ala20Glu	3	1	-	-	Deleterious
20	p.Ala20Pro	2	1	-	-	Deleterious
20	p.Ala20Arg	1	-	-	-	Deleterious
20	p.Ala20Ser	2	-	-	-	Indeterminate
20	p.Ala20Thr	4	-	-	-	Indeterminate
21	p.Ala21Asp	3	-	-	1	Deleterious
21	p.Ala21Pro	1	-	-	-	Deleterious
22	p.Arg22Pro	4	1	-	-	Deleterious
23	p.Gly23Cys	1	1	-	-	Deleterious
23	p.Gly23Asp	1	1	-	-	Deleterious
23	p.Gly23Arg	2	1	1	-	Deleterious
23	p.Gly23Ser	3	-	-	-	Deleterious
23	p.Gly23Val	5	1	-	-	Deleterious
24	p.Arg24Gly	1	1	-	-	Neutral
24	p.Arg24Pro	8	-	-	1	Deleterious
24	p.Arg24Gln	1	-	-	-	Indeterminate
25	p.Val25Gly	2	-	-	-	Indeterminate
26	p.Glu26Asp	1	-	-	-	Neutral
26	p.Glu26Lys	1	-	-	-	Neutral
28	p.Val28Gly	2	-	-	-	Indeterminate
29	p.Arg29Pro	3	-	-	-	Deleterious
29	p.Arg29Trp	1	-	-	-	Neutral
30	p.Ala30Pro	1	-	-	-	Deleterious
30	p.Ala30Val	4	-	-	-	Indeterminate
31	p.Leu31Pro	1	-	-	-	Deleterious
31	p.Leu31Arg	1	1	-	-	Neutral
31	p.Leu31Val	1	1	-	-	Indeterminate
32	p.Leu32Pro	3	1	-	2	Deleterious
32	p.Leu32Gln	2	-	-	2	Indeterminate
33	p.Glu33Asp	1	-	-	-	Neutral
34	p.Ala34Asp	1	-	-	-	Neutral
35	p.Gly35Ala	1	-	-	-	Indeterminate
35	p.Gly35Glu	1	-	-	-	Indeterminate
35	p.Gly35Arg	2	-	-	-	Neutral
35	p.Gly35Val	3	-	-	-	Deleterious
35	p.Gly35Trp	2	-	-	1	Deleterious
36	p.Ala36Gly	3	-	-	-	Indeterminate

36	p.Ala36Ser	-	1	-	-	Neutral
36	p.Ala36Thr	1	-	-	-	Indeterminate
38	p.Pro38His	1	-	-	1	Deleterious
38	p.Pro38Leu	2	-	-	-	Indeterminate
38	p.Pro38Ser	1	-	-	-	Indeterminate
38	p.Pro38Thr	-	-	1	-	Indeterminate
39	p.Asn39Ile	1	-	-	1	Indeterminate
39	p.Asn39Lys	1	-	-	-	Indeterminate
40	p.Ala40Pro	1	-	-	-	Indeterminate
42	p.Asn42Asp	1	-	-	-	Indeterminate
42	p.Asn42His	4	1	-	1	Deleterious
42	p.Asn42Ile	2	1	-	-	Deleterious
42	p.Asn42Tyr	2	-	-	1	Deleterious
43	p.Ser43Ile	-	1	-	-	Indeterminate
43	p.Ser43Arg	1	-	-	-	Neutral
44	p.Tyr44Cys	1	1	-	-	Neutral
45	p.Gly45Asp	3	-	-	-	Neutral
45	p.Gly45Ser	1	-	-	-	Indeterminate
47	p.Arg47Met	1	-	-	1	Neutral
48	p.Pro48Leu	28	6	-	4	Deleterious
48	p.Pro48Gln	1	2	-	-	Deleterious
48	p.Pro48Arg	3	1	-	-	Deleterious
48	p.Pro48Ser	2	1	-	-	Neutral
49	p.Ile49Asn	2	-	-	1	Deleterious
49	p.Ile49Ser	2	-	-	-	Deleterious
49	p.Ile49Thr	3	-	-	-	Indeterminate
50	p.Gln50His	3	1	-	1	Deleterious
50	p.Gln50Leu	2	-	-	-	Indeterminate
50	p.Gln50Arg	3	1	-	2	Deleterious
51	p.Val51Ala	3	-	-	-	Neutral
51	p.Val51Asp	4	-	-	-	Deleterious
51	p.Val51Phe	2	-	1	-	Deleterious
51	p.Val51Ile	3	-	-	-	Neutral
52	p.Met52Ile	1	-	1	-	Indeterminate
52	p.Met52Lys	3	-	-	-	Deleterious
52	p.Met52Leu	1	-	-	-	Neutral
52	p.Met52Arg	3	1	-	-	Deleterious
53	p.Met53Ile	9	1	3	1	Deleterious
53	p.Met53Val	-	-	2	-	Deleterious
54	p.Met54Leu	-	-	1	-	Neutral
55	p.Gly55Cys	1	-	1	-	Deleterious
55	p.Gly55Asp	2	-	-	-	Deleterious
55	p.Gly55Arg	3	-	-	-	Deleterious
55	p.Gly55Val	3	-	1	1	Deleterious
56	p.Ser56Asn	1	-	1	-	Neutral
56	p.Ser56Arg	1	1	-	-	Neutral
57	p.Ala57Phe	1	-	-	-	Indeterminate
57	p.Ala57Pro	1	-	-	-	Neutral
57	p.Ala57Ser	2	-	-	-	Neutral
57	p.Ala57Thr	2	-	-	-	Neutral
57	p.Ala57Val	6	-	2	-	Neutral
58	p.Arg58Gln	2	-	-	-	Neutral
58	p.Val58Leu	-	-	1	-	Neutral
59	p.Val59Met	1	-	-	-	Neutral
60	p.Ala60Glu	1	-	-	-	Indeterminate
60	p.Ala60Ser	1	-	-	-	Indeterminate
60	p.Ala60Thr	-	-	1	-	Neutral
60	p.Ala60Val	5	1	-	1	Neutral
61	p.Glu61Lys	1	1	-	-	Neutral
61	p.Glu61Val	-	-	1	-	Neutral
62	p.Leu62Met	-	1	1	-	Neutral
62	p.Leu62Pro	3	-	-	2	Indeterminate
63	p.Leu63Gln	5	-	1	3	Deleterious

63	p.Leu63Arg	1	-	1	-	Deleterious
63	p.Leu63Val	1	-	-	-	Deleterious
64	p.Leu64Pro	2	1	2	-	Deleterious
65	p.Leu65Pro	1	-	-	-	Deleterious
65	p.Leu65Val	-	-	1	-	Neutral
66	p.His66Leu	1	-	-	-	Neutral
66	p.His66Arg	5	-	3	-	Neutral
66	p.His66Tyr	1	-	1	-	Neutral
67	p.Gly67Cys	2	-	-	1	Neutral
67	p.Gly67Asp	2	-	-	-	Neutral
67	p.Gly67Ser	5	-	1	-	Neutral
67	p.Gly67Val	-	1	-	-	Deleterious
68	p.Ala68Glu	2	-	-	1	Indeterminate
68	p.Ala68Gly	1	-	-	-	Neutral
68	p.Ala68Pro	2	1	1	-	Deleterious
68	p.Ala68Thr	8	-	-	1	Neutral
68	p.Ala68Val	2	-	3	-	Indeterminate
69	p.Glu69Asp	3	-	-	-	Neutral
69	p.Glu69Gly	-	-	4	-	Indeterminate
69	p.Glu69Lys	1	-	-	-	Indeterminate
69	p.Glu69Val	1	-	-	-	Indeterminate
70	p.Pro70Leu	4	-	2	2	Neutral
70	p.Pro70Arg	1	-	-	-	Neutral
70	p.Pro70Ser	1	-	-	-	Indeterminate
71	p.Asn71Asp	1	-	-	-	Indeterminate
71	p.Asn71His	-	-	1	-	Indeterminate
71	p.Asn71Ile	2	-	-	-	Deleterious
71	p.Asn71Lys	-	1	-	-	Deleterious
71	p.Asn71Ser	-	-	1	-	Deleterious
71	p.Asn71Tyr	1	-	-	-	Deleterious
72	p.Cys72Gly	1	-	-	-	Neutral
72	p.Cys72ser	-	-	1	-	Indeterminate
72	p.Cys72Tyr	-	-	1	-	Neutral
73	p.Ala73Asp	2	1	-	-	Neutral
73	p.Ala73Ser	1	-	-	-	Neutral
74	p.Asp74Ala	5	-	-	-	Deleterious
74	p.Asp74Gly	1	-	3	-	Deleterious
74	p.Asp74Asn	10	1	2	2	Deleterious
74	p.Asp74Val	3	1	1	-	Deleterious
74	p.Asp74Tyr	9	1	2	2	Deleterious
75	p.Pro75Ser	1	-	1	-	Neutral
75	p.Pro75Thr	1	-	-	-	Neutral
76	p.Ala76Gly	1	-	-	1	Neutral
76	p.Ala76Ser	1	-	-	-	Indeterminate
76	p.Ala76Thr	7	1	-	-	Neutral
76	p.Ala76Val	6	-	-	-	Neutral
77	p.Thr77Ile	1	1	1	-	Neutral
77	p.Thr77Ser	1	-	-	-	Indeterminate
78	p.Leu78Phe	-	-	2	-	Neutral
78	p.Leu78His	1	-	-	-	Neutral
79	p.Thr79Ala	1	-	-	-	Neutral
79	p.Thr79Ile	5	-	-	-	Neutral
79	p.Thr79Asn	-	-	1	-	Neutral
79	p.Thr79Pro	1	-	-	1	Deleterious
80	p.Arg80Leu	-	-	1	-	Indeterminate
80	p.Arg80Pro	-	-	1	-	Deleterious
80	p.Arg80Gln	4	1	-	-	Indeterminate
81	p.Pro81Ala	1	-	-	-	Indeterminate
81	p.Pro81His	3	1	1	-	Deleterious
81	p.Pro81Leu	28	5	6	3	Deleterious
81	p.Pro81Arg	3	-	-	1	Deleterious
81	p.Pro81Ser	2	-	1	-	Deleterious
82	p.Val82Glu	2	1	-	-	Indeterminate

82	p.Val82Gly	1	-	1	-	Indeterminate
82	p.Val82Leu	2	-	-	1	Neutral
82	p.Val82Met	8	2	1	-	Neutral
83	p.His83Asp	8	3	2	2	Deleterious
83	p.His83Leu	2	1	2	-	Deleterious
83	p.His83Asn	4	-	1	1	Deleterious
83	p.His83Pro	4	-	-	-	Deleterious
83	p.His83Gln	2	1	-	1	Deleterious
83	p.His83Arg	13	2	2	1	Deleterious
83	p.His83Tyr	139	18	17	32	Deleterious
84	p.Asp84Ala	2	-	1	1	Deleterious
84	p.Asp84Gly	15	3	2	2	Deleterious
84	p.Asp84His	3	-	1	-	Deleterious
84	p.Asp84Asn	40	10	3	4	Deleterious
84	p.Asp84Val	5	1	-	2	Deleterious
84	p.Asp84Tyr	24	4	8	5	Deleterious
85	p.Ala85Pro	5	1	1	2	Indeterminate
85	p.Ala85Ser	1	-	1	-	Neutral
85	p.Ala85Thr	3	2	-	-	Indeterminate
86	p.Ala86Asp	4	1	-	-	Deleterious
86	p.Ala86Pro	-	1	-	-	Deleterious
87	p.Arg87Leu	1	-	1	-	Indeterminate
87	p.Arg87Pro	3	-	1	2	Deleterious
87	p.Arg87Trp	3	-	2	-	Deleterious
88	p.Glu88Ala	1	-	-	-	Neutral
88	p.Glu88Asp	2	-	-	-	Indeterminate
88	p.Glu88Lys	15	5	1	3	Indeterminate
88	p.Glu88Val	1	-	-	-	Neutral
89	p.Gly89Cys	2	-	-	2	Deleterious
89	p.Gly89Asp	-	-	1	-	Deleterious
89	p.Gly89Phe	-	-	1	-	Deleterious
89	p.Gly89Ser	4	-	1	1	Deleterious
89	p.Gly89Val	2	1	-	-	Deleterious
90	p.Phe90Leu	10	1	-	1	Neutral
91	p.Leu91Gln	1	-	-	-	Neutral
92	p.Asp92Tyr	1	-	-	1	Neutral
93	p.Thr93Ala	1	-	-	-	Indeterminate
93	p.Thr93Lys	6	-	-	1	Deleterious
93	p.Thr93Met	6	-	-	1	Indeterminate
93	p.Thr93Arg	1	-	-	-	Indeterminate
94	p.Leu94Pro	2	1	-	-	Deleterious
94	p.Leu94Gln	-	1	-	-	Deleterious
95	p.Val95Ala	1	-	-	-	Neutral
95	p.Val95Leu	2	-	-	-	Neutral
95	p.Val95Met	1	-	-	-	Indeterminate
97	p.Leu97Pro	3	-	-	-	Deleterious
97	p.Leu97Arg	4	-	-	-	Deleterious
98	p.His98Pro	4	2	1	1	Deleterious
98	p.His98Tyr	2	-	-	1	Neutral
99	p.Arg99Gln	4	-	-	1	Neutral
99	p.Arg99Trp	1	-	1	-	Indeterminate
100	p.Ala100Pro	1	-	-	-	Deleterious
100	p.Ala100Ser	3	-	2	-	Neutral
100	p.Ala100Thr	2	-	-	-	Neutral
100	p.Ala100Val	2	-	-	-	Neutral
101	p.Gly101Val	4	2	4	-	Deleterious
101	p.Gly101Trp	8	1	4	1	Deleterious
102	p.Ala102Glu	7	1	2	-	Deleterious
102	p.Ala102Thr	3	-	1	-	Deleterious
102	p.Ala102Val	10	1	4	2	Indeterminate
103	p.Arg103Gln	1	-	1	-	Neutral
103	p.Arg103Trp	2	1	-	1	Neutral
104	p.Leu104Gln	1	1	-	-	Neutral

104	p.Leu104Arg	2	-	1	1	Deleterious
104	p.Leu104Val	1	-	-	-	Neutral
105	p.Asp105Asn	1	-	-	-	Neutral
106	p.Val106Met	4	-	-	-	Neutral
107	p.Arg107Cys	2	-	2	-	Indeterminate
107	p.Arg107His	2	1	-	-	Neutral
108	p.Asp108Ala	1	-	-	-	Deleterious
108	p.Asp108Gly	6	1	1	-	Deleterious
108	p.Asp108His	15	1	-	3	Deleterious
108	p.Asp108Asn	20	5	1	5	Neutral
108	p.Asp108Val	3	1	-	-	Deleterious
108	p.Asp108Tyr	39	11	5	5	Deleterious
109	p.Ala109Pro	-	-	1	-	Deleterious
109	p.Ala109Thr	2	-	-	-	Neutral
109	p.Ala109Val	1	-	-	-	Neutral
110	p.Trp110Cys	1	-	-	-	Neutral
110	p.Trp110Arg	1	-	-	-	Neutral
111	p.Gly111Asp	2	1	-	-	Neutral
111	p.Gly111Ser	1	-	-	-	Neutral
111	p.Gly111Val	1	-	1	-	Deleterious
112	p.Arg112Cys	3	-	1	-	Indeterminate
112	p.Arg112His	2	2	1	-	Neutral
112	p.Arg112Pro	2	-	-	1	Deleterious
112	p.Arg112ser	2	-	-	-	Neutral
114	p.Pro114Phe	1	-	1	1	Deleterious
114	p.Pro114His	2	-	-	-	Deleterious
114	p.Pro114Leu	78	13	11	11	Deleterious
114	p.Pro114Arg	2	-	-	-	Deleterious
114	p.Pro114Ser	4	-	-	1	Indeterminate
114	p.Pro114Thr	4	3	-	-	Deleterious
115	p.Val115Glu	1	-	-	-	Indeterminate
115	p.Val115Leu	4	-	-	-	Neutral
116	p.Asp116Asn	-	-	1	-	Neutral
116	p.Asp116Tyr	3	-	-	1	Neutral
118	p.Ala118Pro	2	1	-	-	Indeterminate
118	p.Ala118Thr	1	1	-	-	Neutral
118	p.Ala118Val	1	-	-	-	Indeterminate
119	p.Glu119Asp	2	2	-	-	Indeterminate
119	p.Glu119Lys	2	1	2	-	Neutral
119	p.Glu119Gln	2	1	-	-	Indeterminate
120	p.Glu120Ala	1	-	-	-	Neutral
120	p.Glu120Lys	4	-	-	1	Neutral
122	p.Gly122Cys	1	-	-	1	Neutral
122	p.Gly122Asp	2	-	-	-	Neutral
122	p.Gly122Ser	2	-	-	-	Neutral
122	p.Gly122Val	-	-	1	-	Neutral
123	p.His123Asn	1	1	-	1	Indeterminate
123	p.His123Gln	2	-	-	-	Neutral
124	p.Arg124His	6	-	-	2	Neutral
124	p.Arg124Cys	1	-	1	-	Neutral
125	p.Asp125His	-	-	3	-	Neutral
125	p.Asp125Asn	3	1	-	-	Neutral
126	p.Val126Ala	1	-	-	1	Neutral
126	p.Val126Asp	3	1	2	-	Deleterious
126	p.Val126Phe	3	-	-	-	Neutral
126	p.Val126Gly	-	1	-	-	Neutral
126	p.Val126Ile	3	-	-	-	Neutral
127	p.Ala127Pro	1	-	-	-	Deleterious
127	p.Ala127Ser	2	-	-	1	Neutral
128	p.Arg128Cys	-	-	-	1	Neutral
128	p.Arg128Leu	-	-	1	-	Neutral
128	p.Arg128Pro	-	-	1	-	Deleterious
128	p.Arg128Gln	2	1	-	-	Neutral

128	p.Arg128Trp	5	-	-	-	Neutral
129	p.Tyr129Cys	3	-	-	2	Neutral
129	p.Tyr129Phe	1	-	-	-	Neutral
129	p.Tyr129His	1	-	-	-	Neutral
129	p.Tyr129Asn	1	1	-	-	Indeterminate
130	p.Leu130Met	-	-	1	-	Neutral
130	p.Leu130Pro	4	1	1	-	Deleterious
130	p.Leu130Gln	8	-	-	2	Indeterminate
130	p.Leu130Arg	5	-	2	-	Deleterious
131	p.Arg131Cys	4	1	-	1	Deleterious
131	p.Arg131His	5	1	-	-	Neutral
131	p.Arg131Leu	1	-	-	-	Neutral
131	p.Arg131Pro	2	-	-	1	Indeterminate
132	p.Ala132Pro	2	-	-	-	Neutral
132	p.Ala132Val	-	1	-	-	Neutral
133	p.Ala133ser	-	-	1	-	Neutral
133	p.Ala133Thr	-	1	-	-	Neutral
134	p.Ala134Val	1	-	-	-	Neutral
135	p.Gly135Ala	1	-	-	-	Neutral
135	p.Gly135Glu	3	-	-	1	Indeterminate
136	p.Gly136Asp	2	-	-	-	Indeterminate
136	p.Gly136Arg	-	-	1	-	Neutral
137	p.Thr137Ala	1	-	-	-	Neutral
138	p.Arg138Thr	2	-	-	1	Neutral
139	p.Gly139Ser	-	-	2	-	Neutral
140	p.Ser140Cys	1	-	-	-	Neutral
140	p.Ser140Asn	1	-	-	-	Neutral
142	p.His142Asn	1	-	-	-	Neutral
142	p.His142Gln	1	-	-	-	Neutral
142	p.His142Tyr	1	-	-	-	Neutral
144	p.Arg144His	2	-	-	2	Neutral
146	p.Asp146Ala	1	-	-	-	Neutral
146	p.Asp146Glu	1	-	-	-	Neutral
146	p.Asp146Gly	-	1	-	-	Neutral
146	p.Asp146Asn	1	-	-	-	Neutral
148	p.Ala148Gly	1	-	-	-	Neutral
148	p.Ala148Thr	10	-	-	-	Neutral
148	p.Ala148Val	1	1	-	-	Indeterminate
149	p.Glu149Lys	1	-	-	-	Neutral
150	p.Gly150Ser	1	1	-	-	Neutral
150	p.Gly150Val	1	-	-	-	Neutral
153	p.Asp153Asn	9	-	1	4	Neutral
154	p.Ile154Thr	1	-	-	-	Neutral
155	p.Pro155Ser	1	-	-	-	Indeterminate

COSMIC - Catalogue Of Somatic Mutations In Cancer; TCGA - The Cancer Genome Atlas; JHU - Johns Hopkins University ; MSKCC-IMPACT - Memorial Sloan Kettering-Integrated Mutation Profiling of Actionable Cancer Targets Clinical Sequencing Cohort.
