# Supplementary material for: Functional characterization of all CDKN2A missense variants and comparison to in silico models of pathogenicity
Source: bioRxiv. 2025 Feb 11:2023.12.28.573507. Originally published 2023 Dec 28. Preprint. [Version 3] doi: 10.1101/2023.12.28.573507 (PMC10793438; doi:10.1101/2023.12.28.573507)
Supplement: Supplement 10 [file media-10.pdf]

Appendix 1-table 10. ***CDKN2A*** **missense and synonymous variants reproted in gnomAD.**

Residue	Variant	Transcript Consequence	Missense or synonymous	Benchmark	Functionally reported VUS	P value	Fucntional characterization	Allele Count	Allele Number	Allele Frequency	ClinVar Clinical Significance	ClinVar Variation ID
1	p.Met1Arg	c.2T>G	Missense			0.86	Neutral	2	1440078	1.38881E-06	Uncertain significance	660169
1	p.Met1Lys	c.2T>A	Missense			0.99	Neutral	11	1591956	6.90974E-06	Uncertain significance	487018
1	p.Met1Val	c.1A>G	Missense			0.06	Neutral	1	606364	1.64917E-06	Uncertain significance	820481
2	p.Glu2Gln	c.4G>C	Missense			1.00	Neutral	2	1592086	1.25621E-06	Uncertain significance	406722
2	p.Glu2Glu	c.6G>A	Synonymous			0.48	Neutral	3	1439810	2.08361E-06	Likely benign	483322
2	p.Glu2Gly	c.5A>G	Missense			1.00	Neutral	1	1439958	6.94E-07		
3	p.Pro3Ala	c.7C>G	Missense			0.08	Neutral	2	1439510	1.38936E-06		
3	p.Pro3Leu	c.8C>T	Missense			0.00	Deleterious	2	1446148	1.38298E-06	Uncertain significance	1448238
3	p.Pro3Pro	c.9G>A	Synonymous			0.48	Neutral	12	1598072	7.50905E-06	Likely benign	389468
3	p.Pro3Ser	c.7C>T	Missense			0.00	Deleterious	3	1591586	1.88491E-06	Uncertain significance	532281
3	p.Pro3Thr	c.7C>A	Missense			0.00	Deleterious	4	1439508	2.77873E-06		
4	p.Ala4Ala	c.12G>A	Synonymous			0.48	Neutral	1	152000	6.57895E-06	Likely benign	1101964
4	p.Ala4Ala	c.12G>T	Synonymous			0.48	Neutral	1	1448522	6.90E-07		
4	p.Ala4Gly	c.11C>G	Missense			0.01	Deleterious	5	1447740	3.45366E-06	Uncertain significance	628437
4	p.Ala4Val	c.11C>T	Missense			0.93	Neutral	1	1447738	6.91E-07	Uncertain significance	1058585
5	p.Ala5Ala	c.15G>C	Synonymous			0.48	Neutral	2	1449148	1.38012E-06		
5	p.Ala5Ala	c.15G>A	Synonymous			0.48	Neutral	2	1449148	1.38012E-06	Likely benign	2129711
5	p.Ala5Ala	c.15G>T	Synonymous			0.48	Neutral	3	1449148	2.07018E-06	Likely benign	229890
5	p.Ala5Glu	c.14C>A	Missense			0.01	Deleterious	1	151816	6.58692E-06	Uncertain significance	489864
5	p.Ala5Thr	c.13G>A	Missense			0.59	Neutral	6	1448480	4.14227E-06	Uncertain significance	233536
6	p.Gly6Glu	c.17G>A	Missense			0.61	Neutral	2	1449782	1.37952E-06	Uncertain significance	483323
6	p.Gly6Trp	c.16G>T	Missense			0.11	Neutral	1	616342	1.62248E-06	Uncertain significance	1525531
6	p.Gly6Val	c.17G>T	Missense			0.97	Neutral	1	1449780	6.90E-07	Uncertain significance	220777
7	p.Ser7Asn	c.20G>A	Missense			0.95	Neutral	1	617530	1.61935E-06		
7	p.Ser7Cys	c.19A>T	Missense			0.11	Neutral	2	1450466	1.37887E-06	Uncertain significance	489865
7	p.Ser7Gly	c.19A>G	Missense			0.99	Neutral	1	151766	6.58909E-06		
8	p.Ser8Arg	c.22A>C	Missense			0.97	Neutral	1	1451436	6.89E-07	Uncertain significance	968555
8	p.Ser8Gly	c.22A>G	Missense			0.75	Neutral	1	1451436	6.89E-07	Uncertain significance	480814
9	p.Met9Arg	c.26T>G	Missense			0.95	Neutral	7	1604276	4.36334E-06	Uncertain significance	821690
9	p.Met9Ile	c.27G>A	Missense			0.82	Neutral	2	619538	3.22821E-06	Uncertain significance	656960
9	p.Met9Leu	c.25A>T	Missense			0.99	Neutral	9	1604328	5.60983E-06	Uncertain significance	483321
9	p.Met9Lys	c.26T>A	Missense			0.85	Neutral	48	1604394	2.99178E-05	flicting interpretations of pathogen	236985
9	p.Met9Thr	c.26T>C	Missense		Functionally neutral	0.83	Neutral	2	1452556	1.37688E-06	Uncertain significance	483319
9	p.Met9Val	c.25A>G	Missense			0.73	Neutral	1	151942	6.58146E-06	Uncertain significance	483345
10	p.Glu10Asp	c.30G>C	Missense			0.23	Neutral	1	619520	1.61415E-06	Uncertain significance	822896
10	p.Glu10Asp	c.30G>T	Missense			0.23	Neutral	2	619520	3.22831E-06		
11	p.Pro11Leu	c.32C>T	Missense		Functionally neutral	0.29	Neutral	2	1454026	1.37549E-06	flicting interpretations of pathogen	584734
11	p.Pro11Pro	c.33T>G	Synonymous			0.48	Neutral	1	1454498	6.88E-07		
11	p.Pro11Pro	c.33T>C	Synonymous			0.48	Neutral	1	1454498	6.88E-07		
11	p.Pro11Ser	c.31C>T	Missense			0.21	Neutral	1	619456	1.61432E-06		
12	p.Ser12Ser	c.36G>T	Synonymous			0.48	Neutral	1	1455028	6.87E-07	Likely benign	1559185
12	p.Ser12Ser	c.36G>A	Synonymous			0.48	Neutral	2	1455028	1.37454E-06	Likely benign	1172273
13	p.Ala13Ala	c.39T>G	Synonymous			0.48	Neutral	3	1607248	1.86654E-06	Likely benign	185104
13	p.Ala13Asp	c.38C>A	Missense			1.00	Neutral	2	622070	3.21507E-06	Uncertain significance	483348
13	p.Ala13Gly	c.38C>G	Missense			1.00	Neutral	1	622070	1.60754E-06		
13	p.Ala13Pro	c.37G>C	Missense			1.00	Neutral	1	1454934	6.87E-07		
13	p.Ala13Thr	c.37G>A	Missense			0.98	Neutral	3	1454934	2.06195E-06		
14	p.Asp14Glu	c.42C>A	Missense			0.25	Neutral	2	622370	3.21352E-06	Uncertain significance	863866
15	p.Trp15Arg	c.43T>C	Missense			0.32	Neutral	3	622444	4.81971E-06		
15	p.Trp15Leu	c.44G>T	Missense			0.70	Neutral	1	833110	1.20032E-06		
16	p.Leu16Arg	c.47T>G	Missense	Pathogenic		0.00	Deleterious	1	1455866	6.87E-07	Pathogenic/Likely pathogenic	219815
16	p.Leu16Pro	c.47T>C	Missense	Pathogenic		0.00	Deleterious	3	1455866	2.06063E-06	Pathogenic/Likely pathogenic	649266
17	p.Ala17Ala	c.51C>T	Synonymous			0.48	Neutral	2	1454700	1.37485E-06	Likely benign	631355
17	p.Ala17Ala	c.51C>G	Synonymous			0.48	Neutral	3	1606690	1.86719E-06	Likely benign	532299
17	p.Ala17Ala	c.51C>A	Synonymous			0.48	Neutral	37	1606690	2.30287E-05	Benign/Likely benign	215440
17	p.Ala17Pro	c.49G>C	Missense			0.32	Neutral	1	1456126	6.87E-07		
17	p.Ala17Val	c.50C>T	Missense			0.49	Neutral	1	619864	1.61326E-06	Uncertain significance	825441
18	p.Thr18Ala	c.52A>G	Missense			0.00	Deleterious	2	620722	3.22205E-06	flicting interpretations of pathogen	920669
18	p.Thr18Lys	c.53C>A	Missense			0.00	Deleterious	1	152026	6.57782E-06	Uncertain significance	491580
18	p.Thr18Pro	c.52A>C	Missense		Functionally deleterious	0.00	Deleterious	2	620722	3.22205E-06	Uncertain significance	232702
18	p.Thr18Thr	c.54G>C	Synonymous			0.48	Neutral	1	621138	1.60995E-06	Likely benign	2560570
18	p.Thr18Thr	c.54G>T	Synonymous			0.48	Neutral	1	621138	1.60995E-06	Likely benign	514155
18	p.Thr18Thr	c.54G>A	Synonymous			0.48	Neutral	7	773146	9.05392E-06	Likely benign	1376569
19	p.Ala19Ala	c.57C>G	Synonymous			0.48	Neutral	1	1455558	6.87E-07	Likely benign	1077933
19	p.Ala19Ala	c.57C>A	Synonymous			0.48	Neutral	2	1455558	1.37404E-06		
19	p.Ala19Ala	c.57C>T	Synonymous			0.48	Neutral	9	1607400	5.5991E-06	Likely benign	414094
19	p.Ala19Thr	c.55G>A	Missense			1.00	Neutral	1	621320	1.60948E-06		
20	p.Ala20Gly	c.59C>G	Missense		Functionally deleterious	0.00	Deleterious	2	1455702	1.37391E-06	Uncertain significance	220344
20	p.Ala20Ser	c.58G>T	Missense			0.00	Deleterious	2	1455404	1.37419E-06	Uncertain significance	664488
21	p.Ala21Ala	c.63C>G	Synonymous			0.48	Neutral	1	623564	1.60368E-06		
21	p.Ala21Asp	c.62C>A	Missense			0.00	Deleterious	1	623328	1.60429E-06	Uncertain significance	1392805
22	p.Arg22Arg	c.66G>C	Synonymous			0.48	Neutral	1	152034	6.57748E-06		
22	p.Arg22Arg	c.64C>A	Synonymous			0.48	Neutral	2	1457054	1.37263E-06	Likely benign	630709
22	p.Arg22Arg	c.66G>A	Synonymous			0.48	Neutral	2	624216	3.20402E-06	Likely benign	230052
22	p.Arg22Gln	c.65G>A	Missense			1.00	Neutral	2	1456978	1.3727E-06	Uncertain significance	491581
22	p.Arg22Gly	c.64C>G	Missense			0.00	Deleterious	2	1457056	1.37263E-06	Uncertain significance	863230
23	p.Gly23Asp	c.68G>A	Missense	Pathogenic		0.00	Deleterious	2	833110	2.40064E-06	Pathogenic/Likely pathogenic	420108
24	p.Arg24Arg	c.72G>T	Synonymous			0.57	Neutral	3	1458286	2.05721E-06	Likely benign	463511
24	p.Arg24Arg	c.72G>A	Synonymous			0.57	Neutral	4	1458286	2.74295E-06		
24	p.Arg24Gln	c.71G>A	Missense			0.00	Deleterious	6	1457942	4.11539E-06	Uncertain significance	406714
24	p.Arg24Gly	c.70C>G	Missense			0.13	Neutral	1	624724	1.60071E-06	Uncertain significance	1481554
24	p.Arg24Pro	c.71G>C	Missense	Pathogenic		0.00	Deleterious	13	1609864	8.07522E-06	Pathogenic/Likely pathogenic	9415
25	p.Val25Ile	c.73G>A	Missense			0.71	Neutral	1	833110	1.20032E-06	Uncertain significance	1376284
25	p.Val25Val	c.75A>T	Synonymous			0.48	Neutral	1	1458796	6.85E-07		
25	p.Val25Val	c.75A>G	Synonymous			0.48	Neutral	11	1458796	7.54046E-06	Likely benign	827146
26	p.Glu26Gln	c.76G>C	Missense			0.17	Neutral	2	625858	3.19561E-06	Uncertain significance	919572
26	p.Glu26Glu	c.78G>A	Synonymous			0.48	Neutral	1	833108	1.20032E-06	Likely benign	1761123
26	p.Glu26Val	c.77A>T	Missense			0.87	Neutral	1	1459254	6.85E-07		
27	p.Glu27Ala	c.80A>C	Missense			0.12	Neutral	1	626382	1.59647E-06	Uncertain significance	628014
27	p.Glu27Asp	c.81G>C	Missense			0.00	Deleterious	1	626370	1.5965E-06	Uncertain significance	483354
27	p.Glu27Asp	c.81G>T	Missense			0.00	Deleterious	2	151976	1.316E-05	Uncertain significance	949317
28	p.Val28Met	c.82G>A	Missense			0.82	Neutral	1	626354	1.59654E-06	Uncertain significance	827567
28	p.Val28Val	c.84G>A	Synonymous			0.48	Neutral	2	1460032	1.36983E-06	flicting interpretations of pathogen	917571
29	p.Arg29Arg	c.87G>C	Synonymous			0.48	Neutral	1	1460268	6.85E-07	Likely benign	927182
29	p.Arg29Arg	c.87G>A	Synonymous			0.48	Neutral	20	1612530	1.24029E-05	Likely benign	230671
29	p.Arg29Pro	c.86G>C	Missense			0.00	Deleterious	1	626996	1.59491E-06	Uncertain significance	1041690
30	p.Ala30Thr	c.88G>A	Missense			0.00	Deleterious	2	833108	2.40065E-06		
30	p.Ala30Val	c.89C>T	Missense			0.01	Deleterious	3	1460210	2.0545E-06	Uncertain significance	246070
31	p.Leu31Leu	c.93G>A	Synonymous			0.48	Neutral	2	1460414	1.36947E-06		
32	p.Leu32Pro	c.95T>C	Missense	Pathogenic		0.00	Deleterious	6	833108	7.20195E-06	Pathogenic/Likely pathogenic	236992
32	p.Leu32Val	c.94C>G	Missense			0.97	Neutral	3	1460394	2.05424E-06	Uncertain significance	532278
33	p.Glu33Glu	c.99G>A	Synonymous			0.48	Neutral	1	627582	1.59342E-06		
34	p.Ala34Ala	c.102G>C	Synonymous			0.57	Neutral	33	779890	4.23137E-05		
34	p.Ala34Glu	c.101C>A	Missense			0.22	Neutral	1	1460594	6.85E-07		
34	p.Ala34Ser	c.100G>T	Missense			0.15	Neutral	1	1460706	6.85E-07		
34	p.Ala34Thr	c.100G>A	Missense			0.02	Neutral	10	1460706	6.846E-06	Uncertain significance	489862
34	p.Ala34Val	c.101C>T	Missense			0.00	Deleterious	2	1460594	1.36931E-06	Uncertain significance	483329
35	p.Gly35Ala	c.104G>C	Missense			0.01	Deleterious	64	1612992	3.96778E-05	flicting interpretations of pathogen	463481
35	p.Gly35Arg	c.103G>A	Missense		Functionally neutral	0.19	Neutral	4	1460806	2.73821E-06	Uncertain significance	483347
35	p.Gly35Glu	c.104G>A	Missense		Functionally neutral	0.00	Deleterious	2	1460806	1.36911E-06	Uncertain significance	188292
35	p.Gly35Val	c.104G>T	Missense		Functionally deleterious	0.00	Deleterious	1	1460806	6.85E-07	Uncertain significance	406709
36	p.Ala36Ala	c.108G>C	Synonymous			0.48	Neutral	5	1458686	3.42774E-06	Likely benign	822139
36	p.Ala36Pro	c.106G>C	Missense			0.00	Deleterious	1	1460710	6.85E-07		
36	p.Ala36Ser	c.106G>T	Missense			0.11	Neutral	3	1460710	2.0538E-06	Uncertain significance	532277
36	p.Ala36Thr	c.106G>A	Missense			0.00	Deleterious	7	1612882	4.34006E-06	Uncertain significance	489863
36	p.Ala36Val	c.107C>T	Missense			0.96	Neutral	7	617654	1.13332E-05	Uncertain significance	483324
37	p.Leu37Arg	c.110T>G	Missense			1.00	Neutral	4	833108	4.8013E-06	Uncertain significance	1331763
37	p.Leu37Leu	c.109C>T	Synonymous			0.48	Neutral	1	1458504	6.86E-07	Likely benign	2563917
37	p.Leu37Leu	c.111G>A	Synonymous			0.48	Neutral	1	625942	1.59759E-06	Likely benign	532301
38	p.Pro38Arg	c.113C>G	Missense			0.00	Deleterious	1	1459894	6.85E-07	Likely pathogenic	1730766
38	p.Pro38His	c.113C>A	Missense			0.00	Deleterious	1	1459894	6.85E-07		
38	p.Pro38Ser	c.112C>T	Missense			0.00	Deleterious	1	626538	1.59607E-06	Uncertain significance	822217
39	p.Asn39Lys	c.117C>A	Missense			0.00	Deleterious	1	626904	1.59514E-06	Uncertain significance	220249
40	p.Ala40Gly	c.119C>G	Missense			0.52	Neutral	1	626832	1.59532E-06		
40	p.Ala40Ser	c.118G>T	Missense			0.56	Neutral	7	1459870	4.79495E-06	Uncertain significance	491563
41	p.Pro41Ala	c.121C>G	Missense			0.83	Neutral	2	1612966	1.23995E-06	Uncertain significance	406713
41	p.Pro41Gln	c.122C>A	Missense		Functionally neutral	0.18	Neutral	6	1612890	3.72003E-06	flicting interpretations of pathogen	141111
41	p.Pro41Leu	c.122C>T	Missense			0.98	Neutral	3	1460848	2.0536E-06	Uncertain significance	1755177
41	p.Pro41Pro	c.123G>A	Synonymous			0.48	Neutral	1	627930	1.59253E-06	flicting interpretations of pathogen	923492
42	p.Asn42Asn	c.126T>C	Synonymous			0.48	Neutral	3	628174	4.77575E-06	Likely benign	1101776
42	p.Asn42Ser	c.125A>G	Missense			0.11	Neutral	3	1461208	2.0531E-06	Uncertain significance	406711
43	p.Ser43Gly	c.127A>G	Missense			0.06	Neutral	4	780364	5.12581E-06	Uncertain significance	630389
44	p.Tyr44Cys	c.131A>G	Missense			0.24	Neutral	1	833108	1.20032E-06	Uncertain significance	630012
44	p.Tyr44Tyr	c.132C>T	Synonymous			0.48	Neutral	4	1461440	2.73703E-06	Likely benign	483351
45	p.Gly45Arg	c.133G>C	Missense			0.71	Neutral	1	1461522	6.84E-07	Uncertain significance	463483
45	p.Gly45Ser	c.133G>A	Missense			0.00	Deleterious	1	1461522	6.84E-07	Uncertain significance	463482
46	p.Arg46Gln	c.137G>A	Missense			0.00	Deleterious	1	628584	1.59088E-06	Uncertain significance	631208
47	p.Arg47Arg	c.139A>C	Synonymous			0.48	Neutral	1	628580	1.59089E-06	Likely benign	1160771
48	p.Pro48Pro	c.144G>A	Synonymous			0.48	Neutral	3	780826	3.84209E-06	Likely benign	232233
48	p.Pro48Thr	c.142C>A	Missense	Pathogenic		0.00	Deleterious	1	628604	1.59083E-06	Pathogenic/Likely pathogenic	188286

49	p.Ile49Ile	c.147C>A	Synonymous			0.48	Neutral	77	1613900	4.77105E-05	Likely benign	184680
49	p.Ile49Ser	c.146T>G	Missense	Likely pathogenic		0.00	Deleterious	2	1461744	1.36823E-06	Pathogenic/Likely pathogenic	430217
49	p.Ile49Thr	c.146T>C	Missense	Likely pathogenic		0.00	Deleterious	178	1613956	0.000110288	flicting interpretations of pathogen	127523
50	p.Gln50Arg	c.149A>G	Missense		Functionally deleterious	0.00	Deleterious	1	628592	1.59086E-06	Pathogenic/Likely pathogenic	232304
50	p.Gln50Leu	c.149A>T	Missense			0.00	Deleterious	1	628592	1.59086E-06	Uncertain significance	133878
51	p.Val51Ile	c.151G>A	Missense			0.02	Neutral	7	1597372	4.3822E-06	Uncertain significance	463485
51	p.Val51Leu	c.151G>C	Missense			0.28	Neutral	5	1445136	3.45988E-06		
53	p.Met53Ile	c.159G>C	Missense	Pathogenic		0.00	Deleterious	29	1597670	1.81514E-05	Pathogenic	9414
54	p.Met54Ile	c.162G>A	Missense			0.81	Neutral	1	612304	1.63318E-06	Uncertain significance	819708
54	p.Met54Leu	c.160A>T	Missense			0.94	Neutral	2	1445492	1.38361E-06	Uncertain significance	1483043
54	p.Met54Leu	c.160A>C	Missense			0.94	Neutral	11	1597750	6.88468E-06	flicting interpretations of pathogen	463486
55	p.Gly55Ala	c.164G>C	Missense			0.00	Deleterious	1	1445362	6.92E-07	Uncertain significance	2563916
55	p.Gly55Ser	c.163G>A	Missense			0.00	Deleterious	1	612334	1.6331E-06		
55	p.Gly55Val	c.164G>T	Missense			0.00	Deleterious	1	152332	6.56461E-06	Uncertain significance	2136747
56	p.Ser56Ile	c.167G>T	Missense	Pathogenic		0.00	Deleterious	2	1445436	1.38367E-06	Pathogenic/Likely pathogenic	9425
57	p.Ala57Asp	c.170C>A	Missense			0.92	Neutral	11	1597598	6.88534E-06	Uncertain significance	483325
57	p.Ala57Gly	c.170C>G	Missense			0.45	Neutral	22	1597598	1.37707E-05	Uncertain significance	187272
57	p.Ala57Val	c.170C>T	Missense	Benign		0.89	Neutral	163	1597598	0.000102028	flicting interpretations of pathogen	220562
58	p.Arg58Gly	c.172C>G	Missense			0.98	Neutral	8	1445042	5.53617E-06	Uncertain significance	532285
59	p.Val59Gly	c.176T>G	Missense	Pathogenic		0.00	Deleterious	3	1444950	2.0762E-06	Pathogenic/Likely pathogenic	9423
59	p.Val59Met	c.175G>A	Missense			0.34	Neutral	1	612098	1.63373E-06	Uncertain significance	1007891
60	p.Ala60Thr	c.178G>A	Missense			0.44	Neutral	2	764162	2.61725E-06	Uncertain significance	406705
61	p.Glu61Asp	c.183G>C	Missense			0.56	Neutral	1	1443704	6.93E-07	Uncertain significance	236982
61	p.Glu61Lys	c.181G>A	Missense			0.87	Neutral	1	611732	1.6347E-06		
62	p.Leu62Val	c.184C>G	Missense			0.98	Neutral	1	152214	6.5697E-06	Uncertain significance	969307
63	p.Leu63Pro	c.188T>C	Missense			0.00	Deleterious	2	1443526	1.3855E-06	flicting interpretations of pathogen	1367946
66	p.His66Arg	c.197A>G	Missense		Functionally neutral	0.26	Neutral	303	1594708	0.000190003	flicting interpretations of pathogen	246117
66	p.His66Gln	c.198C>G	Missense			0.47	Neutral	9	1442770	6.238E-06	Uncertain significance	573530
67	p.Gly67Arg	c.199G>C	Missense		Functionally neutral	1.00	Neutral	4	1442608	2.77276E-06	flicting interpretations of pathogen	216272
67	p.Gly67Asp	c.200G>A	Missense			0.86	Neutral	3	761738	3.93836E-06	Uncertain significance	216273
67	p.Gly67Ser	c.199G>A	Missense			0.96	Neutral	2	1442606	1.38638E-06	Uncertain significance	925139
68	p.Ala68Gly	c.203C>G	Missense			0.38	Neutral	13	1441598	9.01777E-06	Uncertain significance	628976
68	p.Ala68Val	c.203C>T	Missense			0.00	Deleterious	1	1441598	6.94E-07	flicting interpretations of pathogen	406701
69	p.Glu69Gly	c.206A>G	Missense			0.00	Deleterious	67	1593674	4.20412E-05	flicting interpretations of pathogen	186615
70	p.Pro70Arg	c.209C>G	Missense			0.72	Neutral	1	1442188	6.93E-07	Uncertain significance	185936
70	p.Pro70Thr	c.208C>A	Missense			0.05	Neutral	4	1441902	2.77411E-06	Uncertain significance	1374577
71	p.Asn71Ser	c.212A>G	Missense	Likely pathogenic		0.00	Deleterious	6	1593656	3.76493E-06	Likely pathogenic	418121
71	p.Asn71Thr	c.212A>C	Missense			0.00	Deleterious	4	1441448	2.77499E-06	Uncertain significance	1786361
73	p.Ala73Ala	c.219C>A	Synonymous			0.57	Neutral	2	152226	1.31384E-05	Likely benign	653470
73	p.Ala73Thr	c.217G>A	Missense			0.68	Neutral	1	606262	1.64945E-06	Uncertain significance	1986070
74	p.Asp74Asn	c.220G>A	Missense			0.00	Deleterious	1	605542	1.65141E-06	Uncertain significance	483340
75	p.Pro75Leu	c.224C>T	Missense			0.29	Neutral	9	1596408	5.63766E-06	Uncertain significance	483326
75	p.Pro75Ser	c.223C>T	Missense			0.31	Neutral	1	610794	1.63721E-06	Uncertain significance	644311
76	p.Ala76Ser	c.226G>T	Missense			0.01	Deleterious	1	152148	6.57255E-06	Uncertain significance	2059365
76	p.Ala76Thr	c.226G>A	Missense			0.14	Neutral	18	1595206	1.12838E-05	Uncertain significance	489866
77	p.Thr77Ala	c.229A>G	Missense			0.01	Deleterious	1	611264	1.63595E-06	Uncertain significance	567670
77	p.Thr77Asn	c.230C>A	Missense			0.04	Neutral	2	1445234	1.38386E-06		
78	p.Leu78Phe	c.232C>T	Missense			0.98	Neutral	2	1445644	1.38347E-06	Uncertain significance	630762
79	p.Thr79Asn	c.236C>A	Missense			0.80	Neutral	3	1603784	1.87058E-06	Uncertain significance	491570
79	p.Thr79Ile	c.236C>T	Missense			0.79	Neutral	5	1451540	3.44462E-06	Uncertain significance	630548
80	p.Arg80Gln	c.239G>A	Missense			0.00	Deleterious	6	1451726	4.13301E-06	Uncertain significance	376385
80	p.Arg80Leu	c.239G>T	Missense			0.00	Deleterious	2	1451724	1.37767E-06		
80	p.Arg80Pro	c.239G>C	Missense			0.00	Deleterious	1	152196	6.57047E-06		
81	p.Pro81Leu	c.242C>T	Missense		Functionally deleterious	0.00	Deleterious	1	618752	1.61616E-06	Likely pathogenic	833629
81	p.Pro81Ser	c.241C>T	Missense			0.00	Deleterious	1	152230	6.56901E-06	Uncertain significance	664812
82	p.Val82Leu	c.244G>T	Missense			0.03	Neutral	2	1451914	1.37749E-06	Uncertain significance	406718
82	p.Val82Met	c.244G>A	Missense			0.33	Neutral	2	1451914	1.37749E-06	Uncertain significance	1063226
83	p.His83Arg	c.248A>G	Missense	Pathogenic		0.00	Deleterious	1	152338	6.56435E-06	flicting interpretations of pathogen	376379
83	p.His83Asn	c.247C>A	Missense			0.00	Deleterious	1	618816	1.61599E-06		
83	p.His83Gln	c.249C>G	Missense			0.00	Deleterious	1	1452118	6.89E-07	Pathogenic	376381
83	p.His83Gln	c.249C>A	Missense			0.00	Deleterious	2	1452116	1.3773E-06	flicting interpretations of pathogen	429110
84	p.Asp84Ala	c.251A>C	Missense		Functionally deleterious	0.00	Deleterious	7	1452176	4.82035E-06	flicting interpretations of pathogen	142882
84	p.Asp84Tyr	c.250G>T	Missense		Functionally deleterious	0.00	Deleterious	1	619038	1.61541E-06	flicting interpretations of pathogen	376306
85	p.Ala85Ser	c.253G>T	Missense			0.07	Neutral	5	771240	6.48307E-06	Uncertain significance	532289
85	p.Ala85Thr	c.253G>A	Missense			0.00	Deleterious	3	619004	4.8465E-06	Uncertain significance	236983
86	p.Ala86Ser	c.256G>T	Missense			0.15	Neutral	1	619124	1.61519E-06	Uncertain significance	1793230
86	p.Ala86Thr	c.256G>A	Missense			0.96	Neutral	3	771478	3.88864E-06		
87	p.Arg87Trp	c.259C>T	Missense	Likely pathogenic		0.00	Deleterious	6	1604616	3.73921E-06	Likely pathogenic	406707
88	p.Glu88Gln	c.262G>C	Missense			0.00	Deleterious	1	619464	1.6143E-06	Uncertain significance	619828
89	p.Gly89Ser	c.265G>A	Missense			0.00	Deleterious	4	619634	6.45542E-06	Uncertain significance	9408
91	p.Leu91Arg	c.272T>G	Missense			0.35	Neutral	1	1453016	6.88E-07		
92	p.Asp92Glu	c.276C>A	Missense			0.07	Neutral	2	619946	3.22609E-06	Uncertain significance	645261
93	p.Thr93Met	c.278C>T	Missense			0.00	Deleterious	1	619998	1.61291E-06	Uncertain significance	1796019
96	p.Val96Gly	c.287T>G	Missense			0.02	Neutral	1	1453218	6.88E-07		
96	p.Val96Leu	c.286G>T	Missense			0.00	Deleterious	1	1453266	6.88E-07	Uncertain significance	2106366
96	p.Val96Met	c.286G>A	Missense			0.62	Neutral	1	1453266	6.88E-07	Uncertain significance	821875
98	p.His98Asn	c.292C>A	Missense			0.63	Neutral	1	620158	1.61249E-06		
98	p.His98Tyr	c.292C>T	Missense			0.68	Neutral	1	620158	1.61249E-06	Uncertain significance	419560
99	p.Arg99Gln	c.296G>A	Missense			0.18	Neutral	4	1453046	2.75284E-06	flicting interpretations of pathogen	628568
99	p.Arg99Gly	c.295C>G	Missense		Functionally neutral	0.43	Neutral	7	1453094	4.81731E-06	flicting interpretations of pathogen	372062
99	p.Arg99Trp	c.295C>T	Missense			0.00	Deleterious	3	1453094	2.06456E-06	Uncertain significance	483350
100	p.Ala100Ser	c.298G>T	Missense	Benign		0.99	Neutral	98	1605346	6.1046E-05	flicting interpretations of pathogen	127524
100	p.Ala100Thr	c.298G>A	Missense			0.37	Neutral	1	1453014	6.88E-07	Uncertain significance	629701
100	p.Ala100Val	c.299C>T	Missense			1.00	Neutral	2	772064	2.59046E-06	Uncertain significance	924911
101	p.Gly101Arg	c.301G>A	Missense		Functionally neutral	0.00	Deleterious	4	1605208	2.49189E-06	Uncertain significance	463494
101	p.Gly101Arg	c.301G>C	Missense		Functionally neutral	0.00	Deleterious	7	1452982	4.81768E-06	Uncertain significance	216274
101	p.Gly101Trp	c.301G>T	Missense	Pathogenic		0.00	Deleterious	12	1605210	7.47566E-06	Pathogenic	9412
102	p.Ala102Thr	c.304G>A	Missense			0.00	Deleterious	2	772270	2.58977E-06	Uncertain significance	463495
102	p.Ala102Val	c.305C>T	Missense			0.00	Deleterious	5	1452980	3.4412E-06	Uncertain significance	234071
103	p.Arg103Gln	c.308G>A	Missense			0.51	Neutral	1	833110	1.20032E-06	Uncertain significance	1141816
103	p.Arg103Trp	c.307C>T	Missense			0.85	Neutral	12	1605324	7.47513E-06	Uncertain significance	423630
105	p.Asp105Asn	c.313G>A	Missense			0.14	Neutral	2	1453262	1.37621E-06	Uncertain significance	491573
105	p.Asp105Glu	c.315C>A	Missense			0.00	Deleterious	20	1605504	1.24571E-05	flicting interpretations of pathogen	406702
106	p.Val106Ala	c.317T>C	Missense			0.02	Neutral	2	833110	2.40064E-06	Uncertain significance	463497
106	p.Val106Gly	c.317T>G	Missense			0.00	Deleterious	1	833110	1.20032E-06		
106	p.Val106Leu	c.316G>C	Missense			0.00	Deleterious	1	1453226	6.88E-07	Uncertain significance	1318653
106	p.Val106Leu	c.316G>T	Missense			0.00	Deleterious	1	152232	6.56892E-06	Uncertain significance	1728421
106	p.Val106Met	c.316G>A	Missense			0.57	Neutral	1	1453226	6.88E-07	Uncertain significance	1392243
107	p.Arg107Cys	c.319C>T	Missense			0.00	Deleterious	3	1453408	2.06411E-06	flicting interpretations of pathogen	483352
107	p.Arg107Gly	c.319C>G	Missense			0.33	Neutral	2	1453408	1.37608E-06		
107	p.Arg107His	c.320G>A	Missense			0.32	Neutral	25	1605852	1.55681E-05	flicting interpretations of pathogen	182413
107	p.Arg107Leu	c.320G>T	Missense			0.24	Neutral	1	1453632	6.88E-07		
107	p.Arg107Ser	c.319C>A	Missense			0.09	Neutral	1	1453408	6.88E-07	Uncertain significance	463498
108	p.Asp108Val	c.323A>T	Missense			0.00	Deleterious	1	620726	1.61102E-06		
109	p.Ala109Ala	c.327C>T	Synonymous			0.57	Neutral	1	1454144	6.88E-07	Likely benign	1123298
109	p.Ala109Pro	c.325G>C	Missense		Functionally deleterious	0.00	Deleterious	19	1606256	1.18287E-05	Uncertain significance	127525
110	p.Trp110Arg	c.328T>C	Missense			0.92	Neutral	1	152190	6.57073E-06	Uncertain significance	2109926
110	p.Trp110Gly	c.328T>G	Missense			0.89	Neutral	1	621072	1.61012E-06		
111	p.Gly111Ser	c.331G>A	Missense		Functionally neutral	0.81	Neutral	6	1454378	4.12547E-06	Uncertain significance	463499
111	p.Gly111Val	c.332G>T	Missense			0.00	Deleterious	1	621180	1.60984E-06	Uncertain significance	1487529
112	p.Arg112Gly	c.334C>G	Missense	Pathogenic		0.00	Deleterious	2	1454592	1.37496E-06	Pathogenic/Likely pathogenic	233484
112	p.Arg112His	c.335G>A	Missense			0.42	Neutral	1	1454582	6.87E-07	Uncertain significance	142887
112	p.Arg112Leu	c.335G>T	Missense			0.00	Deleterious	7	1606934	4.35612E-06	Uncertain significance	823661
113	p.Leu113Val	c.337C>G	Missense			0.91	Neutral	1	152250	6.56814E-06		
114	p.Pro114Ser	c.340C>T	Missense			0.00	Deleterious	1	152346	6.56401E-06	Uncertain significance	1405931
115	p.Val115Glu	c.344T>A	Missense			0.00	Deleterious	10	1607670	6.22018E-06	Uncertain significance	246437
115	p.Val115Leu	c.343G>T	Missense			0.13	Neutral	32	1607650	1.99048E-05	Uncertain significance	419130
115	p.Val115Met	c.343G>A	Missense			0.37	Neutral	3	1455412	2.06127E-06	Uncertain significance	1469323
116	p.Asp116Glu	c.348C>G	Missense			0.30	Neutral	1	622926	1.60533E-06		
116	p.Asp116Tyr	c.346G>T	Missense			0.56	Neutral	1	622770	1.60573E-06	Uncertain significance	2563909
117	p.Leu117Gln	c.350T>A	Missense			0.07	Neutral	1	833110	1.20032E-06		
118	p.Ala118Gly	c.353C>G	Missense			0.13	Neutral	1	623472	1.60392E-06		
118	p.Ala118Thr	c.352G>A	Missense			0.03	Neutral	7	1608806	4.35105E-06	Uncertain significance	491574
118	p.Ala118Val	c.353C>T	Missense			0.01	Deleterious	1	623472	1.60392E-06	flicting interpretations of pathogen	406719
119	p.Glu119Glu	c.357G>A	Synonymous			0.48	Neutral	1	623920	1.60277E-06		
119	p.Glu119Lys	c.355G>A	Missense			0.81	Neutral	2	1456610	1.37305E-06	Uncertain significance	570565
120	p.Glu120Asp	c.360G>T	Missense			0.21	Neutral	5	1457182	3.43128E-06	Uncertain significance	823988
120	p.Glu120Gln	c.358G>C	Missense			0.06	Neutral	1	623948	1.6027E-06		
120	p.Glu120Glu	c.360G>A	Synonymous			0.57	Neutral	2	1457184	1.37251E-06	Likely benign	463502
120	p.Glu120Val	c.359A>T	Missense			0.33	Neutral	1	624016	1.60252E-06		
121	p.Leu121Arg	c.362T>G	Missense			0.00	Deleterious	1	152228	6.56909E-06		
121	p.Leu121Leu	c.361C>T	Synonymous			0.48	Neutral	146	1609492	9.07119E-05	Benign/Likely benign	630387
121	p.Leu121Met	c.361C>A	Missense			0.00	Deleterious	1	1457230	6.86E-07		
121	p.Leu121Val	c.361C>G	Missense			0.97	Neutral	29	1609610	1.80168E-05	flicting interpretations of pathogen	219905
122	p.Gly122Asp	c.365G>A	Missense			0.95	Neutral	1	1457356	6.86E-07		
122	p.Gly122Gly	c.366C>G	Synonymous			0.62	Neutral	1	624330	1.60172E-06		
122	p.Gly122Gly	c.366C>T	Synonymous			0.62	Neutral	1	624330	1.60172E-06	Likely benign	1133481
122	p.Gly122Val	c.365G>T	Missense			0.49	Neutral	38	1609606	2.36083E-05	flicting interpretations of pathogen	182419
123	p.His123Gln	c.369T>A	Missense	Benign		0.10	Neutral	359	1610194	0.000222955	flicting interpretations of pathogen	127526
123	p.His123His	c.369T>C	Synonymous			0.62	Neutral	15	1610076	9.31633E-06	Likely benign	220829
124	p.Arg124Cys	c.370C>T	Missense		Functionally neutral	0.39	Neutral	73	1610132	4.53379E-05	flicting interpretations of pathogen	231362
124	p.Arg124Gly	c.370C>G	Missense			0.00	Deleterious	9	1610014	5.59001E-06	Uncertain significance	582036
124	p.Arg124His	c.371G>A	Missense			0.80	Neutral	1	624836	1.60042E-06		

125	p.Asp125Asn	c.373G>A	Missense			0.62	Neutral	2	1610302	1.242E-06	Uncertain significance	858988
125	p.Asp125Glu	c.375T>A	Missense			0.80	Neutral	2	777462	2.57247E-06	Uncertain significance	1329079
125	p.Asp125His	c.373G>C	Missense		Functionally neutral	0.95	Neutral	569	1610420	0.000353324	flicting interpretations of pathoger	41577
126	p.Val126Asp	c.377T>A	Missense	Pathogenic		0.00	Deleterious	3	1458484	2.05693E-06	Pathogenic	9420
126	p.Val126Leu	c.376G>C	Missense			1.00	Neutral	1	1458368	6.86E-07	Uncertain significance	489868
126	p.Val126Phe	c.376G>T	Missense			0.75	Neutral	1	1458368	6.86E-07		
126	p.Val126Val	c.378C>T	Synonymous			0.48	Neutral	1	1458494	6.86E-07	Likely benign	1692141
126	p.Val126Val	c.378C>G	Synonymous			0.48	Neutral	1	1458494	6.86E-07		
127	p.Ala127Pro	c.379G>C	Missense		Functionally deleterious	0.00	Deleterious	1	1458444	6.86E-07	flicting interpretations of pathoger	571866
127	p.Ala127Ser	c.379G>T	Missense	Likely benign		0.23	Neutral	1532	1610798	0.000951081	Benign/Likely benign	41578
127	p.Ala127Val	c.380C>T	Missense			0.83	Neutral	1	152238	6.56866E-06	Uncertain significance	2030143
128	p.Arg128Gln	c.383G>A	Missense			0.93	Neutral	19	1610916	1.17945E-05	Uncertain significance	581636
128	p.Arg128Pro	c.383G>C	Missense			0.00	Deleterious	1	1458694	6.86E-07	Uncertain significance	483344
130	p.Leu130Leu	c.388C>T	Synonymous			0.48	Neutral	2	778188	2.57007E-06	flicting interpretations of pathoger	491575
131	p.Arg131Cys	c.391C>T	Missense			0.00	Deleterious	1	1459134	6.85E-07	Uncertain significance	1350948
131	p.Arg131His	c.392G>A	Missense			0.64	Neutral	2	1459184	1.37063E-06	Uncertain significance	620615
132	p.Ala132Ala	c.396G>C	Synonymous			0.48	Neutral	1	152250	6.56814E-06	Likely benign	382703
132	p.Ala132Ala	c.396G>T	Synonymous			0.48	Neutral	1	626296	1.59669E-06		
132	p.Ala132Val	c.395C>T	Missense			0.05	Neutral	5	1459290	3.42632E-06	Uncertain significance	630452
133	p.Ala133Ala	c.399T>C	Synonymous			0.48	Neutral	2	1459450	1.37038E-06		
134	p.Ala134Ala	c.402G>A	Synonymous			0.57	Neutral	1	1459422	6.85E-07	flicting interpretations of pathoger	583214
134	p.Ala134Ala	c.402G>T	Synonymous			0.57	Neutral	9	1611668	5.58428E-06	Likely benign	236989
134	p.Ala134Pro	c.400G>C	Missense			0.68	Neutral	5	1611668	3.10238E-06	Uncertain significance	495536
134	p.Ala134Val	c.401C>T	Missense			0.93	Neutral	3	626164	4.79108E-06	Uncertain significance	650117
135	p.Gly135Glu	c.404G>A	Missense			0.00	Deleterious	5	1611926	3.10188E-06	Uncertain significance	463504
135	p.Gly135Gly	c.405G>A	Synonymous			0.48	Neutral	306	1611984	0.000189828	Benign/Likely benign	383182
135	p.Gly135Val	c.404G>T	Missense			0.37	Neutral	1	1459674	6.85E-07	Uncertain significance	843913
136	p.Gly136Ala	c.407G>C	Missense			0.66	Neutral	2	152212	1.31396E-05	Uncertain significance	483335
136	p.Gly136Asp	c.407G>A	Missense			0.00	Deleterious	5	626354	7.98271E-06	Uncertain significance	925925
136	p.Gly136Ser	c.406G>A	Missense			0.03	Neutral	5	1612104	3.10154E-06	Uncertain significance	483342
137	p.Thr137Pro	c.409A>C	Missense			0.70	Neutral	1	1459840	6.85E-07		
137	p.Thr137Ser	c.410C>G	Missense			0.77	Neutral	1	626710	1.59563E-06		
138	p.Arg138Gly	c.412A>G	Missense			0.77	Neutral	15	1612210	9.304E-06	Uncertain significance	233990
139	p.Gly139Arg	c.415G>C	Missense		Functionally neutral	0.33	Neutral	27	1460010	1.8493E-05	flicting interpretations of pathoger	216276
139	p.Gly139Asp	c.416G>A	Missense			0.01	Deleterious	9	1612280	5.58216E-06	Uncertain significance	491576
139	p.Gly139Ser	c.415G>A	Missense			0.05	Neutral	4	1460010	2.73971E-06	Uncertain significance	141419
139	p.Gly139Val	c.416G>T	Missense			0.83	Neutral	1	1459922	6.85E-07	Uncertain significance	2172999
140	p.Ser140Ser	c.420T>C	Synonymous			0.48	Neutral	2	1460096	1.36977E-06	Likely benign	1738745
141	p.Asn141Asn	c.423C>T	Synonymous			0.48	Neutral	1	1460072	6.85E-07	Likely benign	924813
141	p.Asn141Asp	c.421A>G	Missense			0.87	Neutral	1	152230	6.56901E-06	Uncertain significance	631362
142	p.His142Arg	c.425A>G	Missense			1.00	Neutral	25	1612402	1.55048E-05	Uncertain significance	184564
142	p.His142Gln	c.426T>G	Missense			1.00	Neutral	1	833110	1.20032E-06	Uncertain significance	246046
142	p.His142Tyr	c.424C>T	Missense			1.00	Neutral	2	626994	3.18982E-06	Uncertain significance	824721
143	p.Ala143Ala	c.429C>A	Synonymous			0.48	Neutral	1	1460104	6.85E-07	Likely benign	414090
143	p.Ala143Gly	c.428C>G	Missense			0.56	Neutral	5	626918	7.97552E-06	Uncertain significance	630386
143	p.Ala143Thr	c.427G>A	Missense		Functionally neutral	0.72	Neutral	16	1460132	1.09579E-05	Uncertain significance	245681
144	p.Arg144Arg	c.432C>T	Synonymous			0.48	Neutral	1	627030	1.59482E-06		
144	p.Arg144Cys	c.430C>T	Missense	Likely benign		0.98	Neutral	763	1612398	0.000473208	Benign/Likely benign	41579
144	p.Arg144His	c.431G>A	Missense			0.68	Neutral	1	152236	6.56875E-06	Uncertain significance	406723
144	p.Arg144Leu	c.431G>T	Missense			0.89	Neutral	10	1612274	6.20242E-06	Uncertain significance	463505
144	p.Arg144Ser	c.430C>A	Missense			0.75	Neutral	1	152216	6.56961E-06		
145	p.Ile145Met	c.435A>G	Missense			0.12	Neutral	1	627006	1.59488E-06		
145	p.Ile145Thr	c.434T>C	Missense			0.49	Neutral	7	1460132	4.79409E-06	flicting interpretations of pathoger	182420
145	p.Ile145Val	c.433A>G	Missense			0.21	Neutral	1	1460158	6.85E-07	Uncertain significance	645545
146	p.Asp146Asn	c.436G>A	Missense			0.12	Neutral	1	1460116	6.85E-07	Uncertain significance	578446
146	p.Asp146Glu	c.438T>A	Missense			0.61	Neutral	1	833110	1.20032E-06		
146	p.Asp146His	c.436G>C	Missense			0.22	Neutral	1	1460116	6.85E-07	Uncertain significance	824834
146	p.Asp146Val	c.437A>T	Missense			0.59	Neutral	1	152216	6.56961E-06	Uncertain significance	928297
147	p.Ala147Ala	c.441C>A	Synonymous			0.48	Neutral	4	1459910	2.73989E-06	Likely benign	391388
147	p.Ala147Val	c.440C>T	Missense			0.31	Neutral	3	779052	3.85083E-06	Uncertain significance	406716
148	p.Ala148Ala	c.444G>A	Synonymous			0.48	Neutral	5	1459850	3.42501E-06	Likely benign	923208
148	p.Ala148Gly	c.443C>G	Missense			0.38	Neutral	1	1459882	6.85E-07		
148	p.Ala148Thr	c.442G>A	Missense	Likely benign		0.40	Neutral	39384	1612214	0.024428519	Benign	41580
148	p.Ala148Val	c.443C>T	Missense			0.00	Deleterious	5	1612130	3.10149E-06	Uncertain significance	231700
150	p.Gly150Asp	c.449G>A	Missense			0.30	Neutral	3	778834	3.85191E-06	Uncertain significance	483331
150	p.Gly150Gly	c.450T>C	Synonymous			0.48	Neutral	5	1459644	3.42549E-06	Likely benign	630385
150	p.Gly150Ser	c.448G>A	Missense			0.40	Neutral	1	626658	1.59577E-06		
151	p.Pro151His	c.452C>A	Missense			0.54	Neutral	1	1459568	6.85E-07		
151	p.Pro151Leu	c.452C>T	Missense			0.08	Neutral	4	1611804	2.48169E-06	Uncertain significance	406724
154	p.Ile154Asn	c.461T>A	Missense			0.06	Neutral	6	628748	9.54277E-06	Uncertain significance	491579
156	p.Asp156Ala	c.467A>C	Missense			0.00	Deleterious	1	833022	1.20045E-06		
156	p.Asp156Asn	c.466G>A	Missense			0.11	Neutral	1	1461724	6.84E-07	Uncertain significance	629192
156	p.Asp156Asp	c.468T>C	Synonymous			0.48	Neutral	4	780878	5.12244E-06	Likely benign	215633
156	p.Asp156Tyr	c.466G>T	Missense			0.01	Deleterious	2	1461722	1.36825E-06	Uncertain significance	1171898
