# Supplementary material for: Functional characterization of all CDKN2A missense variants and comparison to in silico models of pathogenicity
Source: bioRxiv. 2025 Feb 11:2023.12.28.573507. Originally published 2023 Dec 28. Preprint. [Version 3] doi: 10.1101/2023.12.28.573507 (PMC10793438; doi:10.1101/2023.12.28.573507)
Supplement: Supplement 12 [file media-12.pdf]

Appendix 1-table 12. Codon optimized *CDKN2A* sequence.

Sequence
ATGGAACCCGCCGCTGGCTCATCAATGGAACCCTCTGCAGATTGGCTCGCT
ACCGCAGCCGCGCGAGGACGAGTGGAAGAAGTCCGAGCGCTCCTTGAAG
CCGGTGCATTGCCAAATGCGCCTAACTCCTATGGACGCCGCCCAATACAAG
TTATGATGATGGGATCTGCAAGGGTAGCTGAACTTCTCCTCTTGCATGGAG
CAGAACCTAATTGTGCTGATCCAGCGACCCTGACTAGACCTGTACATGATG
CCGCGCGTGAAGGGTTTCTCGATACCCTTGTCGTCCTTCATCGAGCTGGTGC
CCGCCTCGATGTCCGGGACGCATGGGGACGGCTCCCAGTCGATCTCGCAG
AAGAACTTGGGCACAGGGACGTAGCCAGATATTTGCGTGCAGCCGCCGGT
GGTACGAGGGGATCAAATCACGCTAGAATCGACGCTGCCGAGGGCCCAA
[illegible]
