# Supplementary material for: Functional characterization of all CDKN2A missense variants and comparison to in silico models of pathogenicity
Source: bioRxiv. 2025 Feb 11:2023.12.28.573507. Originally published 2023 Dec 28. Preprint. [Version 3] doi: 10.1101/2023.12.28.573507 (PMC10793438; doi:10.1101/2023.12.28.573507)
Supplement: Supplement 13 [file media-13.pdf]

Appendix 1-table 13. Sequences of primers used in study.

Oligos	Sequence
**Primers used to generate variant *CDKN2A* expression plasmids**	
p.Ser7Gly_FWD	CGCCGCTGGCGGCTCAATGGAAC
p.Ser7Gly_REV	GGTTCCATTTAGTCTGGTATG
p.Glu26Cys_FWD	AGGACGAGTGTGCGAAGTCCGAGCGCTCCTTGAAGCCGG
p.Glu26Cys_REV	CGCGCGGCTGCGGTAGCG
p.Glu26Pro_FWD	AGGACGAGTGCCCGAAGTCCGAGCGCTCCTTGAAGCCG
p.Glu26Pro_REV	CGCGCGGCTGCGGTAGCG
p.Glu27Ala_FWD	ACGAGTGGAAGCCGTCCGAGCGCTCCTTGAAGC
p.Glu27Ala_REV	CCTCGCGCGGCTGCGGTA
p.Glu27Thr_FWD	ACGAGTGGAAACCGTCCGAGCGCTCCTTGAAGCCG
p.Glu27Thr_REV	CCTCGCGCGGCTGCGGTA
p.Glu27His_FWD	ACGAGTGGAACACGTCCGAGCGCTCCTTGAAGC
p.Glu27His_REV	CCTCGCGCGGCTGCGGTA
p.Glu27Pro_FWD	ACGAGTGGAACCCGTCCGAGCGCTCCTTGAAGCCGGTG
p.Glu27Pro_REV	CCTCGCGCGGCTGCGGTA
p.Glu27Ile_FWD	ACGAGTGGAAATCGTCCGAGCGCTCCTTGAAGCCG
p.Glu27Ile_REV	CCTCGCGCGGCTGCGGTA
p.Glu69Ala_FWD	GCATGGAGCAGCCCCTAATTGTG
p.Glu69Ala_REV	AAGAGGAGAAGTTCAGCTAC
p.Val95His_FWD	CGATACCCTTCACGTCCTTCATCGAG
p.Val95His_REV	AGAAACCCTTCACGCGCG
p.Ala100Gly_FWD	CCGCCTCGATGTCCGGGACGCATG
p.Ala100Gly_REV	GCACCGCCTCGATGAAGGACGAC
p.Arg103Lys_FWD	AGCTGGTGCCAAGCTCGATGTCC
p.Arg103Lys_REV	CGATGAAGGACGACAAGG
p.Arg103Leu_FWD	AGCTGGTGCCCTGCTCGATGTCC
p.Arg103Leu_REV	CGATGAAGGACGACAAGG
p.Arg103Gln_FWD	AGCTGGTGCCCAGCTCGATGTCC
p.Arg103Gln_REV	CGATGAAGGACGACAAGG
p.Ala109Asp_FWD	TGTCCGGGACGACTGGGGACGGCTC
p.Ala109Asp_REV	TCGAGGCGGGCACCAGCT
p.Trp110Pro_FWD	CCGGGACGCACCCGGACGGCTCCCAG
p.Trp110Pro_REV	ACATCGAGGCGGGCACCA
p.Val115Cys_FWD	ACGGCTCCCATGCGATCTCGCAGAAGAAC
p.Val115Cys_REV	CCCCATGCGTCCCGGACA
p.Gly136Cys_FWD	AGCCGCCGGTTGCACGAGGGGAT
p.Gly136Cys_REV	GCACGCAAATATCTGGCTACG
p.Pro151Gly_FWD	TGCCGAGGGCGGCAGCGACATAC
p.Pro151Gly_REV	GCGTCGATTCTAGCGTGA
p.Ile154Gly_FWD	CCCAAGCGACGGCCCAGACTAAGAATTCTCGACC
p.Ile154Gly_REV	CCCTCGGCAGCGTCGATT
p.L32P_FWD	CCGAGCGCTCCCCGAAGCCGGTG
p.L32P_REV	ACTTCTTCCACTCGTCCTCG
p.G101W_FWD	TCATCGAGCTTGGGCCCGCCTCG
p.G101W_REV	AGGACGACAAGGGTATCGAGAAAC
p.V126D_FWD	GCACAGGGACGACGCCAGATATTTG
p.V126D_REV	CCAAGTTCTTCTGCGAGATC
p.L32L_FWD	CCGAGCGCTCCTGGAAGCCGGTG
p.L32L_REV	ACTTCTTCCACTCGTCCTCGCGC
p.G101G_FWD	TCATCGAGCTGGCGCCCGCCTCG
p.G101G_REV	AGGACGACAAGGGTATCGAGAAACC
p.V126V_FWD	GCACAGGGACGTGGCCAGATATT
p.V126V_REV	CCAAGTTCTTCTGCGAGATCG
**Primers used to generate CellTag plasmids**	
Barcode 1_FWD	CTGCGGCAGCGGCGCCACCAAC
Barcode 1_REV	TACCACTTATCGTCATCGTCTTTGTAATCCATGGTGGC
Barcode 2_FWD	CGATAAGGATCATAGCGGCAGCGGCG
Barcode 2_REV	TCATCGTCTTTGTAATCC
Barcode 3_FWD	GAATGGCAGCGGCGCCACCAAC
Barcode 3_REV	TGAATCTTATCGTCATCGTCTTTGTAATCCATGGTGGC
Barcode 4_FWD	GTACGGCAGCGGCGCCACCAAC
Barcode 4_REV	TGCTCCTTATCGTCATCGTCTTTGTAATCCATGGTGGC
Barcode 5_FWD	GTACGGCAGCGGCGCCACCAAC
Barcode 5_REV	ATATCCTTATCGTCATCGTCTTTGTAATCCATGGTGGC
Barcode 6_FWD	GTTCGGCAGCGGCGCCACCAAC
Barcode 6_REV	TCAACCTTATCGTCATCGTCTTTGTAATCCATGGTGGC
Barcode 7_FWD	ACATGGCAGCGGCGCCACCAAC
Barcode 7_REV	TGATACTTATCGTCATCGTCTTTGTAATCCATGGTGGC
Barcode 8_FWD	AGCTGGCAGCGGCGCCACCAAC
Barcode 8_REV	TCGCACTTATCGTCATCGTCTTTGTAATCCATGGTGGC
Barcode 9_FWD	GACGATAAGGATTGTACCGGCAGCGG
Barcode 9_REV	ATCGTCTTTGTAATCCATGGTG
Barcode 10_FWD	GGTCGGCAGCGGCGCCACCAAC
Barcode 10_REV	CCTGGCTTATCGTCATCGTCTTTGTAATCCATGGTGGC
Barcode 11_FWD	ACGATAAGATCACGATCGGCAGCGGC
Barcode 11_REV	CATCGTCTTTGTAATCCATG
Barcode 12_FWD	CGATAAGGTGAGGAGCGGCAGCGGCG
Barcode 12_REV	TCATCGTCTTTGTAATCCATGG
Barcode 13_FWD	TATAGGCAGCGGCGCCACCAAC
Barcode 13_REV	CAACCCTTATCGTCATCGTCTTTGTAATCCATGGTGGC
Barcode 14_FWD	GATAAGGTTCGTATCGGCAGCGG
Barcode 14_REV	GTCATCGTCTTTGTAATCC
Barcode 15_FWD	GTACGGCAGCGGCGCCACCAAC
Barcode 15_REV	AGAACCTTATCGTCATCGTCTTTGTAATCCATGGTGGC
Barcode 16_FWD	AGCCGGCAGCGGCGCCACCAAC
Barcode 16_REV	ACCGCCTTATCGTCATCGTCTTTGTAATCCATGGTGGC
Barcode 17_FWD	TGATGGCAGCGGCGCCACCAAC
Barcode 17_REV	ACGTACTTATCGTCATCGTCTTTGTAATCCATGGTGGC
Barcode 18_FWD	CTCAGGCAGCGGCGCCACCAAC
Barcode 18_REV	CACTGCTTATCGTCATCGTCTTTGTAATCCATGGTGGC
Barcode 19_FWD	CTGTGGCAGCGGCGCCACCAAC
Barcode 19_REV	TTCTGCTTATCGTCATCGTCTTTGTAATCCATGGTGGC
Barcode 20_FWD	CAATGGCAGCGGCGCCACCAAC
Barcode 20_REV	TATCCCTTATCGTCATCGTCTTTGTAATCCATGGTGGC
**1st stage primers used to generate sequence libraries**	
CellTag_FWD	GTAAAACGACGGCCAGGTGTCGTGACGCGGGATCCG
CellTag_REV	CAGGAAACAGCTATGACTGTAGCCGTTGCTCTTTCAA
p.AA1-53_FWD	GTAAAACGACGGCCAGCGCTACCGGGTACAAGTCC
p.AA1-53_REV	CAGGAAACAGCTATGACTCAGCTACCCTTGCAGATCC
p.AA54-110_FWD	GTAAAACGACGGCCAGCCGCCCAATACAAGTTATG
p.AA54-110_REV	CAGGAAACAGCTATGACCCCAAGTTCTTCTGCGAGA
p.AA111-156_FWD	GTAAAACGACGGCCAGCGATACCCTTGTCGTCCTTC
p.AA111-156_REV	CAGGAAACAGCTATGACACTGCCATTTGTCTCGAGGT
**2nd stage primers used to generate sequence libraries**	
FWD1	AATGATACGGCGACCACCGAGATCTACACTCTTTCCCTACACGACGCTCTTCCGATCTATGTAAAACGACGGCCAG
FWD2	AATGATACGGCGACCACCGAGATCTACACTCTTTCCCTACACGACGCTCTTCCGATCTGATGTAAAACGACGGCCAG
FWD3	AATGATACGGCGACCACCGAGATCTACACTCTTTCCCTACACGACGCTCTTCCGATCTCGATGTAAAACGACGGCCAG
FWD4	AATGATACGGCGACCACCGAGATCTACACTCTTTCCCTACACGACGCTCTTCCGATCTTCGATGTAAAACGACGGCCAG
FWD5	AATGATACGGCGACCACCGAGATCTACACTCTTTCCCTACACGACGCTCTTCCGATCTATCGATGTAAAACGACGGCCAG
FWD6	AATGATACGGCGACCACCGAGATCTACACTCTTTCCCTACACGACGCTCTTCCGATCTGATCGATGTAAAACGACGGCCAG
FWD7	AATGATACGGCGACCACCGAGATCTACACTCTTTCCCTACACGACGCTCTTCCGATCTCGATCGATGTAAAACGACGGCCAG
FWD8	AATGATACGGCGACCACCGAGATCTACACTCTTTCCCTACACGACGCTCTTCCGATCTTCGATCGATGTAAAACGACGGCCAG
REV1	CAAGCAGAAGACGGCATACGAGATGCTGGATTGTGACTGGAGTTCAGACGTGTGCTCTTCCGATCTATCAGGAAACAGCTATGAC
REV2	CAAGCAGAAGACGGCATACGAGATTAACTCGGGTGACTGGAGTTCAGACGTGTGCTCTTCCGATCTGATCAGGAAACAGCTATGAC
REV3	CAAGCAGAAGACGGCATACGAGATTAACAGTTGTGACTGGAGTTCAGACGTGTGCTCTTCCGATCTCGATCAGGAAACAGCTATGAC
REV4	CAAGCAGAAGACGGCATACGAGATATACTCAAGTGACTGGAGTTCAGACGTGTGCTCTTCCGATCTTCGATCAGGAAACAGCTATGAC
REV5	CAAGCAGAAGACGGCATACGAGATGCTGAGAAGTGACTGGAGTTCAGACGTGTGCTCTTCCGATCTATCGATCAGGAAACAGCTATGAC
REV6	CAAGCAGAAGACGGCATACGAGATATTGGAGGGTGACTGGAGTTCAGACGTGTGCTCTTCCGATCTGATCGATCAGGAAACAGCTATGAC
REV7	CAAGCAGAAGACGGCATACGAGATTAGTCTAAGTGACTGGAGTTCAGACGTGTGCTCTTCCGATCTCGATCGATCAGGAAACAGCTATGAC
REV8	CAAGCAGAAGACGGCATACGAGATCGGTGACCGTGACTGGAGTTCAGACGTGTGCTCTTCCGATCTTCGATCGATCAGGAAACAGCTATGAC
REV9	CAAGCAGAAGACGGCATACGAGATTACAGAGGGTGACTGGAGTTCAGACGTGTGCTCTTCCGATCTATCAGGAAACAGCTATGAC
REV10	CAAGCAGAAGACGGCATACGAGATATTGTCAAGTGACTGGAGTTCAGACGTGTGCTCTTCCGATCTGATCAGGAAACAGCTATGAC
REV11	CAAGCAGAAGACGGCATACGAGATTATGTCTTGTGACTGGAGTTCAGACGTGTGCTCTTCCGATCTCGATCAGGAAACAGCTATGAC
REV12	CAAGCAGAAGACGGCATACGAGATATTGGATTGTGACTGGAGTTCAGACGTGTGCTCTTCCGATCTTCGATCAGGAAACAGCTATGAC
REV13	CAAGCAGAAGACGGCATACGAGATATACTCGGGTGACTGGAGTTCAGACGTGTGCTCTTCCGATCTATCGATCAGGAAACAGCTATGAC
REV14	CAAGCAGAAGACGGCATACGAGATTATGAGAAGTGACTGGAGTTCAGACGTGTGCTCTTCCGATCTGATCGATCAGGAAACAGCTATGAC
REV15	CAAGCAGAAGACGGCATACGAGATGCACAGTTGTGACTGGAGTTCAGACGTGTGCTCTTCCGATCTCGATCGATCAGGAAACAGCTATGAC
REV16	CAAGCAGAAGACGGCATACGAGATCGTGGATTGTGACTGGAGTTCAGACGTGTGCTCTTCCGATCTTCGATCGATCAGGAAACAGCTATGAC

FWD - forward primer; REV - reverse primer.
