# Supplementary figures and images for: Functional characterization of all CDKN2A missense variants and comparison to in silico models of pathogenicity
Source: bioRxiv. 2025 Feb 11:2023.12.28.573507. Originally published 2023 Dec 28. Preprint. [Version 3] doi: 10.1101/2023.12.28.573507 (PMC10793438; doi:10.1101/2023.12.28.573507)

Figure 1-figure supplement 1

A

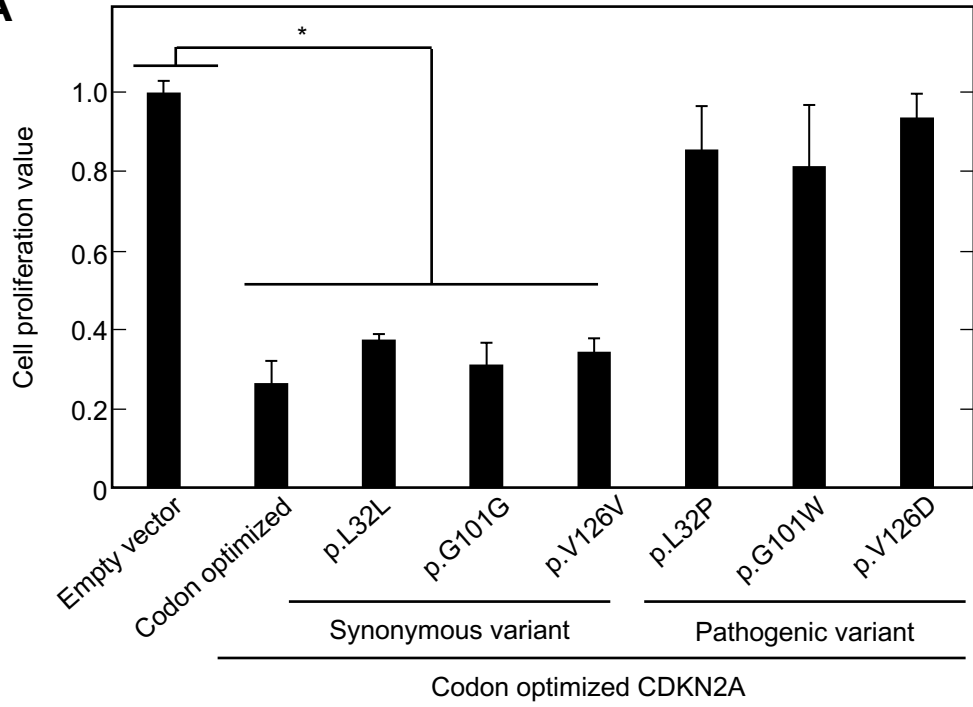

B

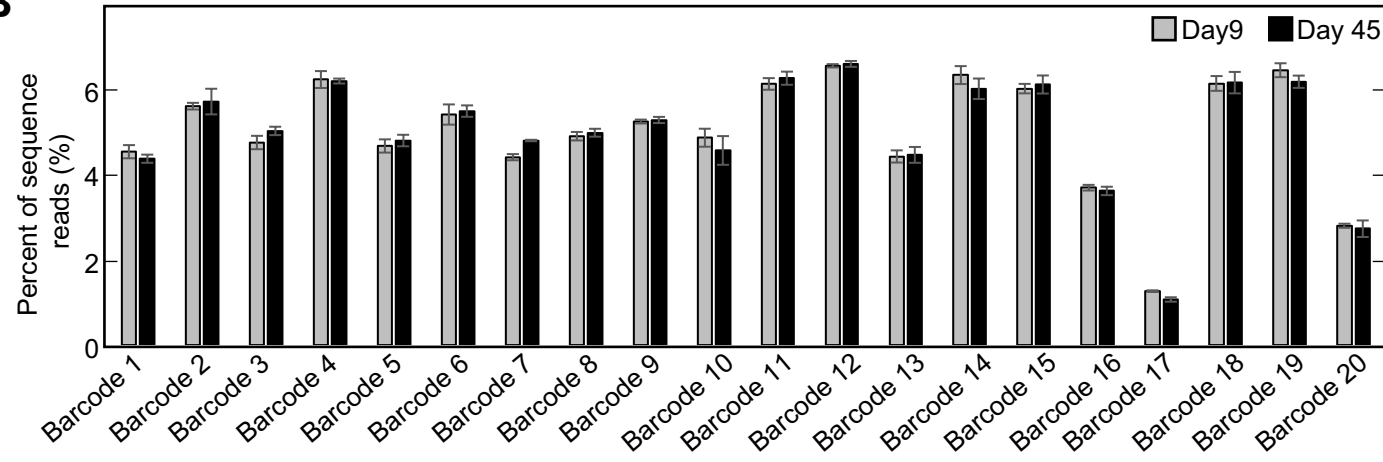

Supplement: Supplement 14 [file media-14.pdf]

Figure 1-figure supplement 2

A

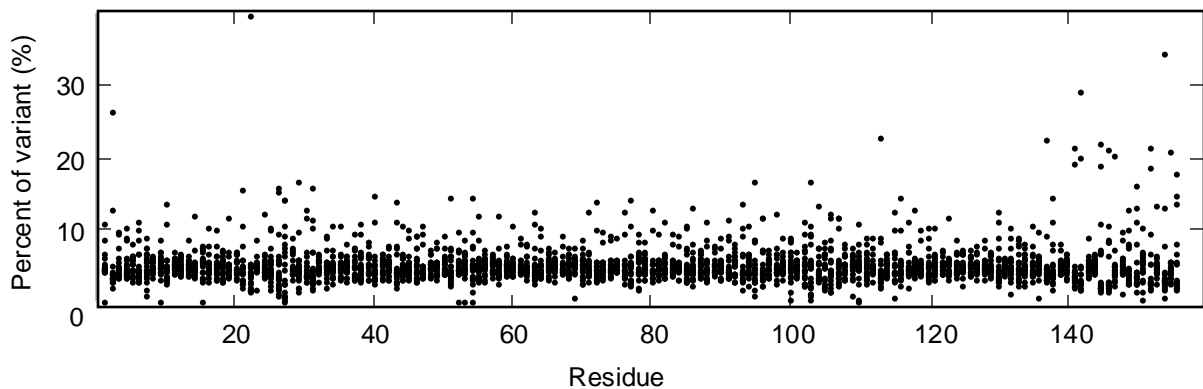

B

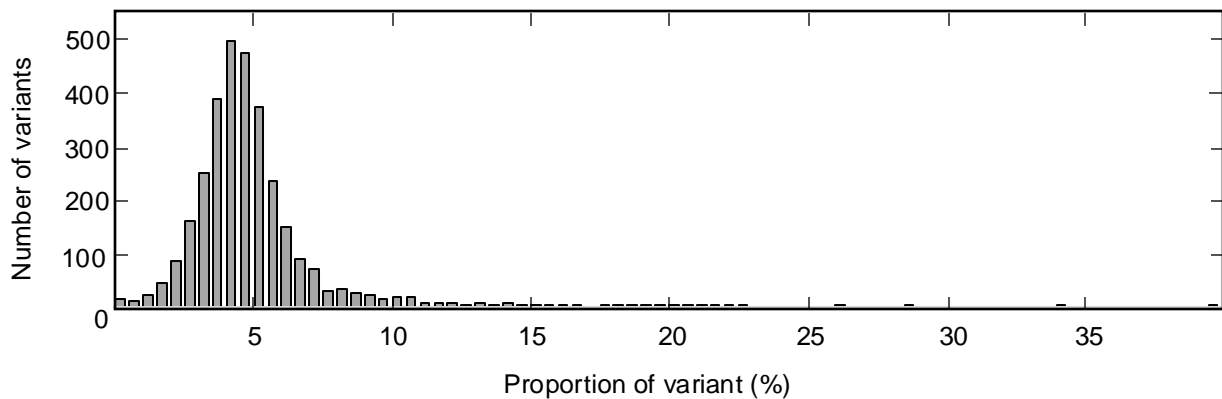

C

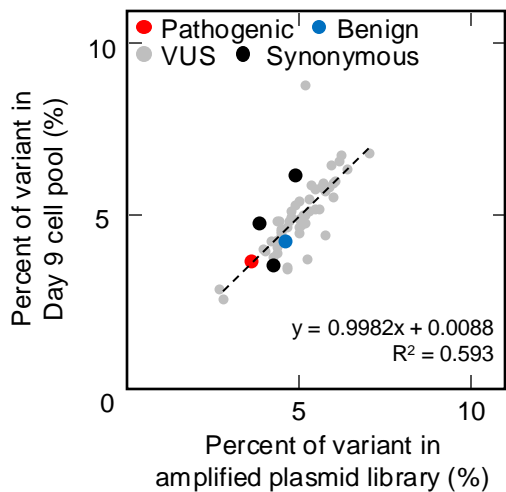

D

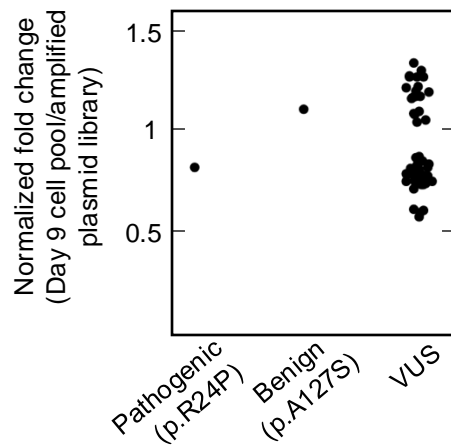

Supplement: Supplement 15 [file media-15.pdf]

Figure 2-figure supplement 1

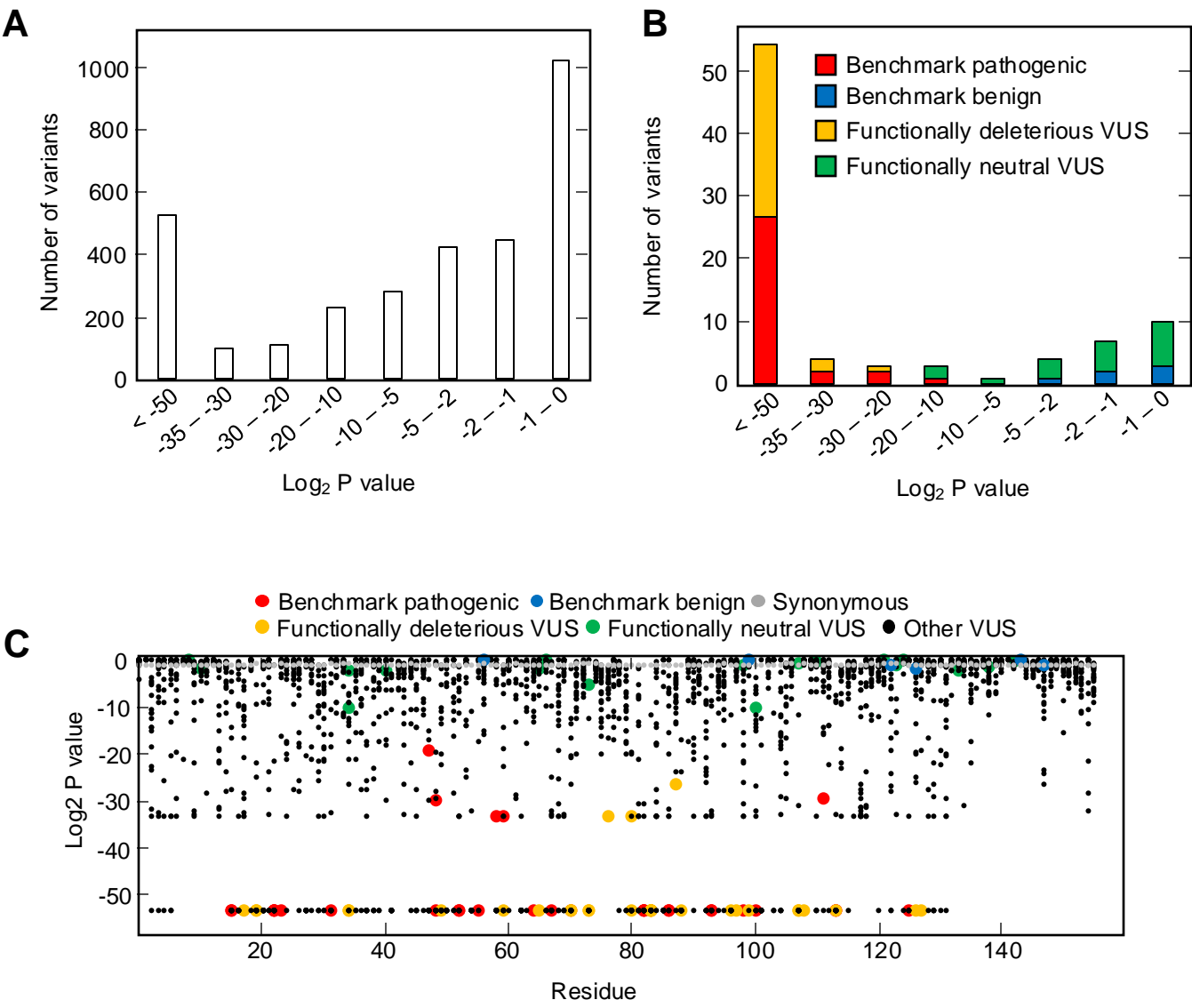

Supplement: Supplement 16 [file media-16.pdf]

Figure 2-figure supplement 2

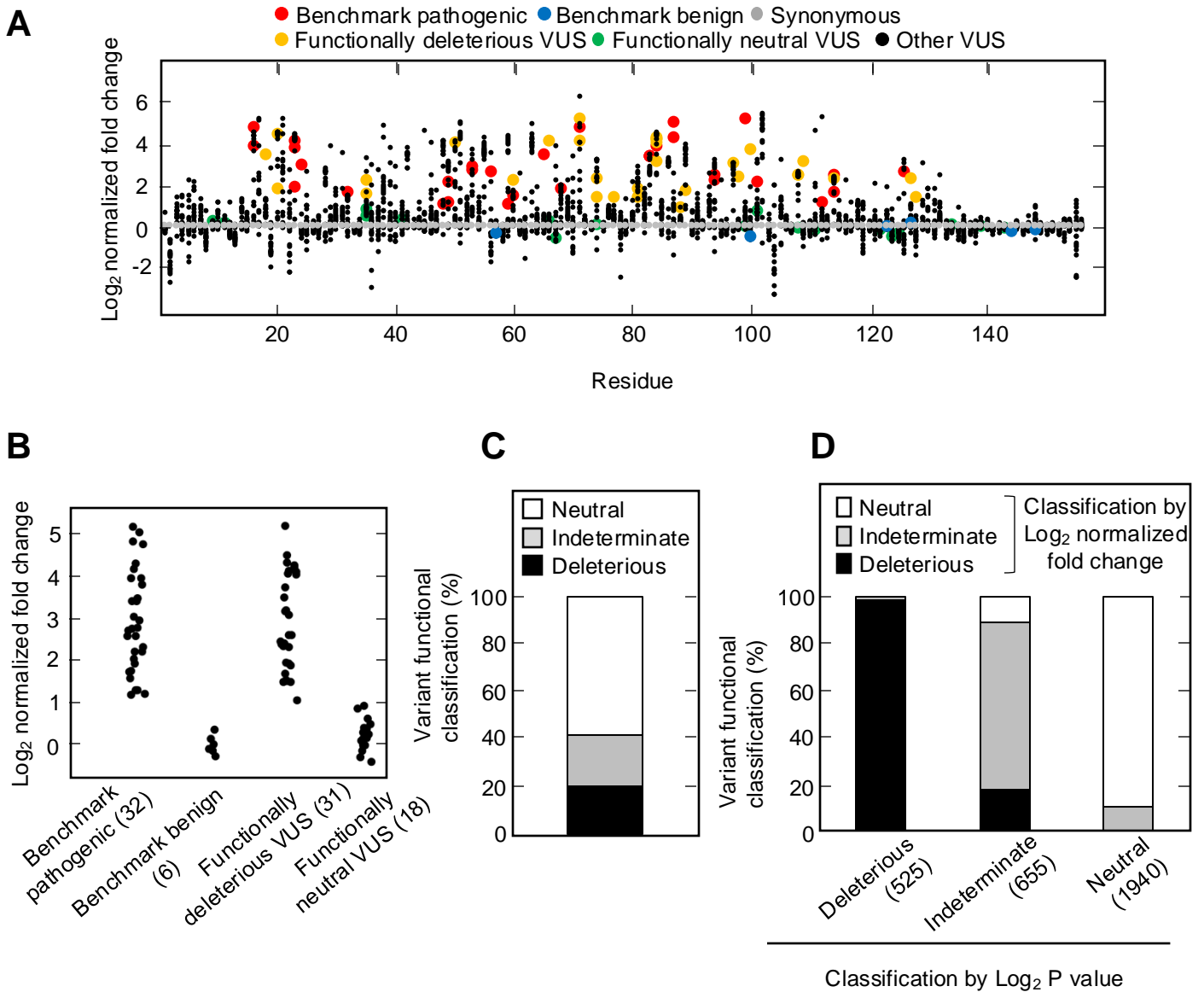

Supplement: Supplement 17 [file media-17.pdf]

Figure 2-figure supplement 3

A

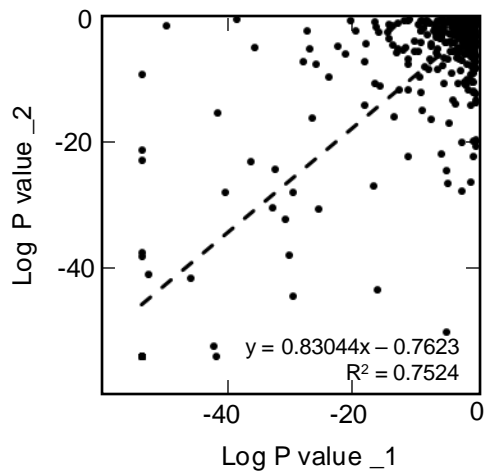

B

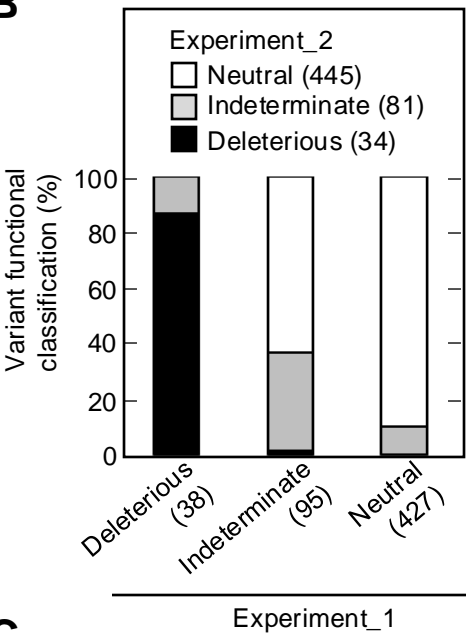

C

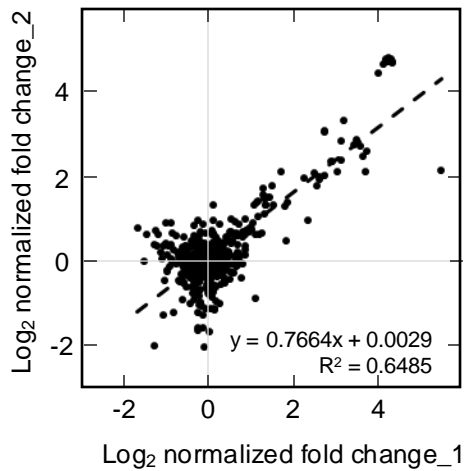

Supplement: Supplement 18 [file media-18.pdf]

Figure 2-figure supplement 4

A

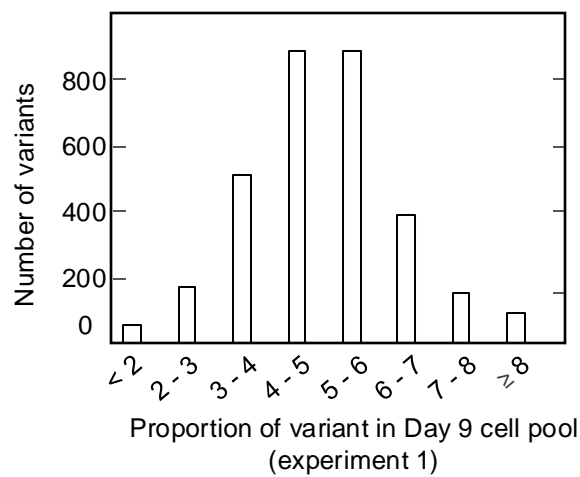

B

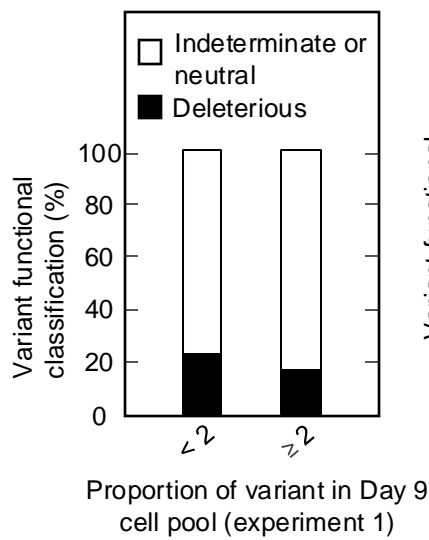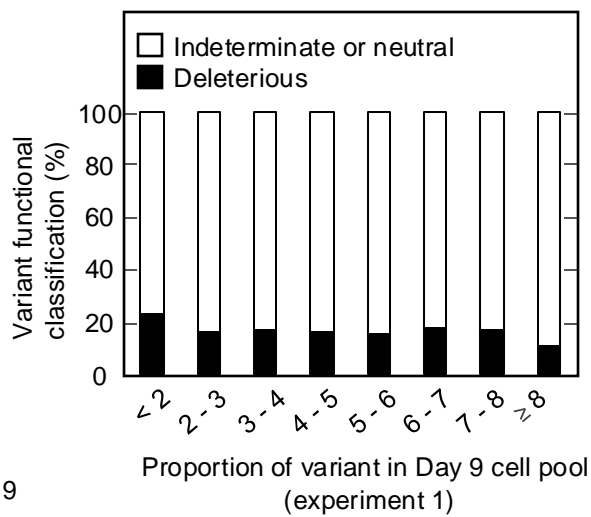

Supplement: Supplement 19 [file media-19.pdf]

Figure 2-figure supplement 5

A

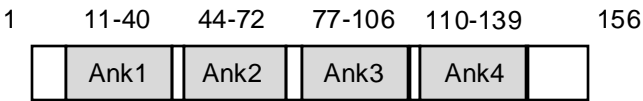

B

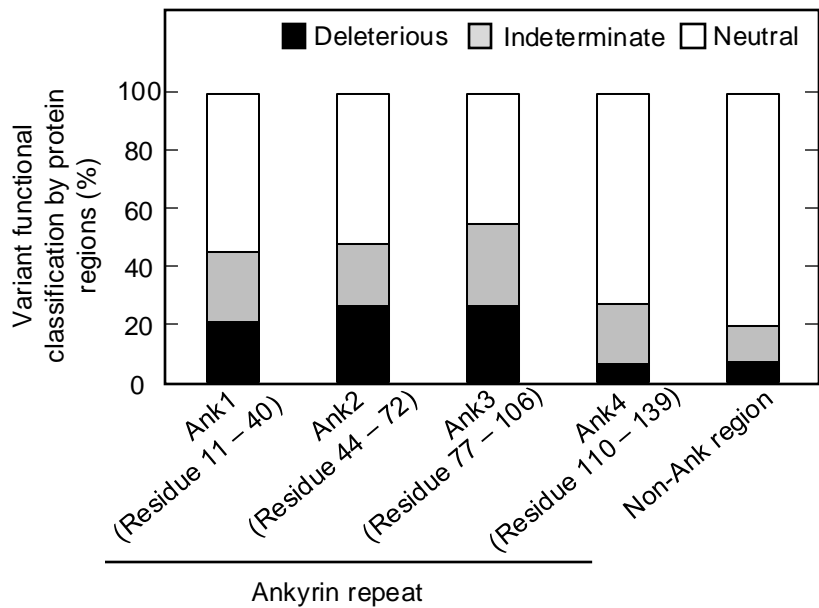

C

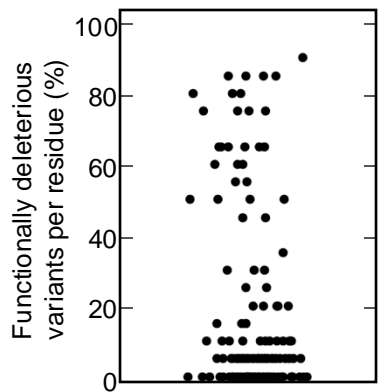

Supplement: Supplement 20 [file media-20.pdf]

Figure 3-figure supplement 1

A

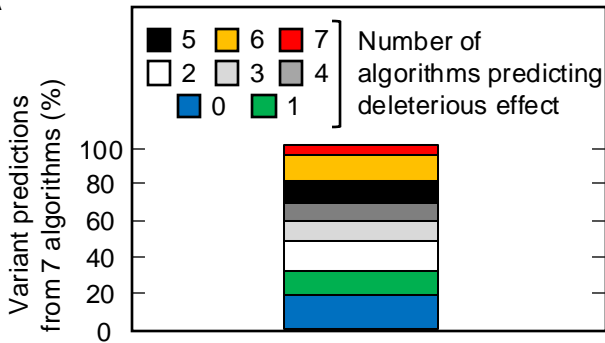

B

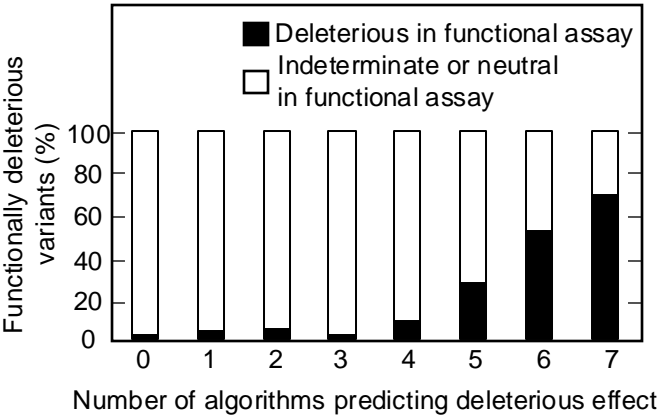

C

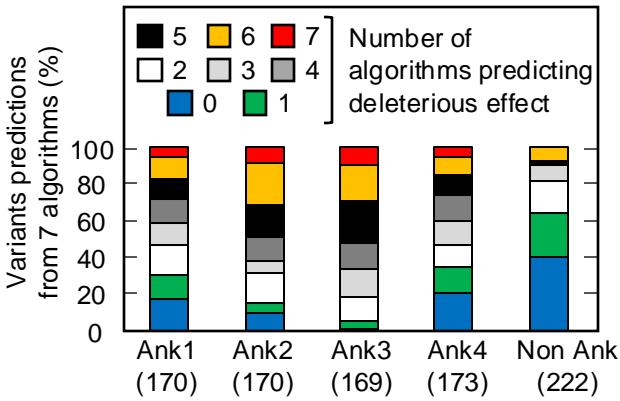

D

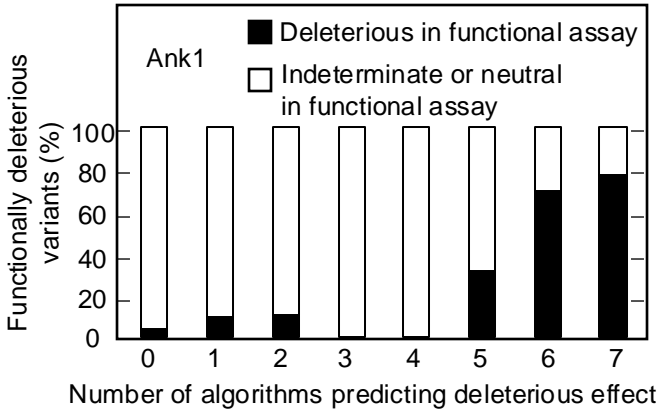

E

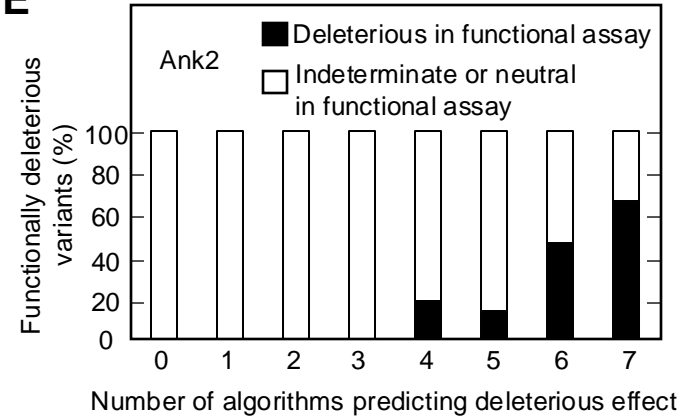

F

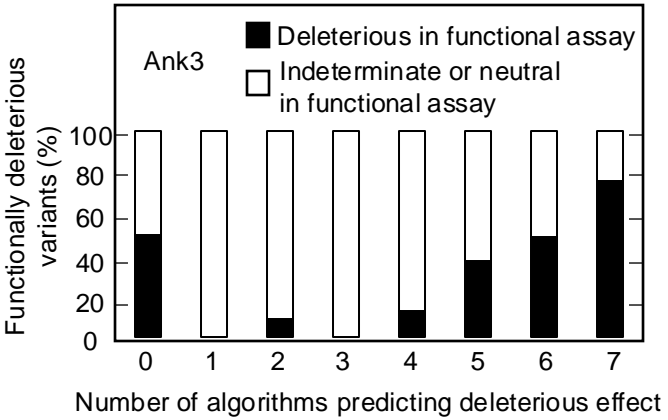

G

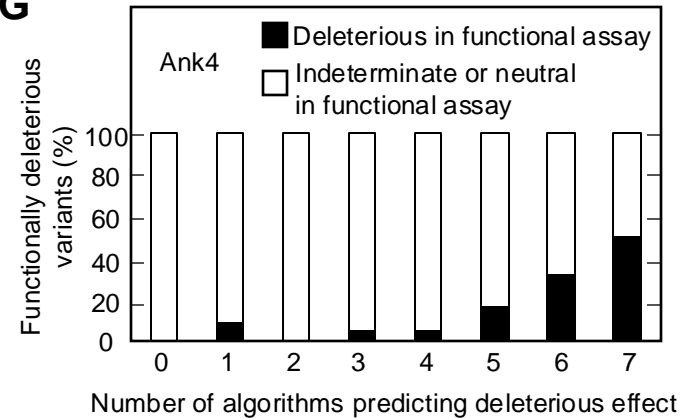

H

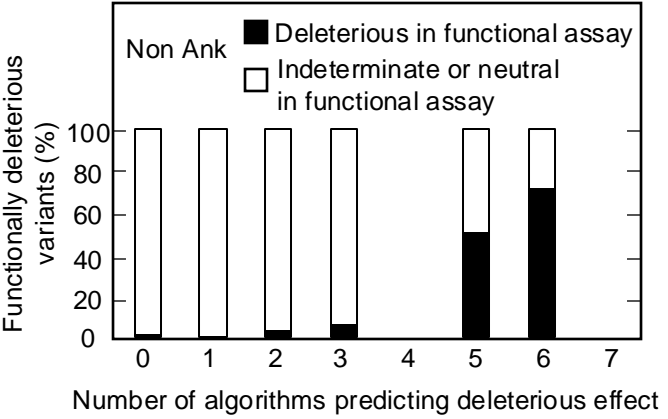

Supplement: Supplement 21 [file media-21.pdf]

Figure 3-figure supplement 2

A

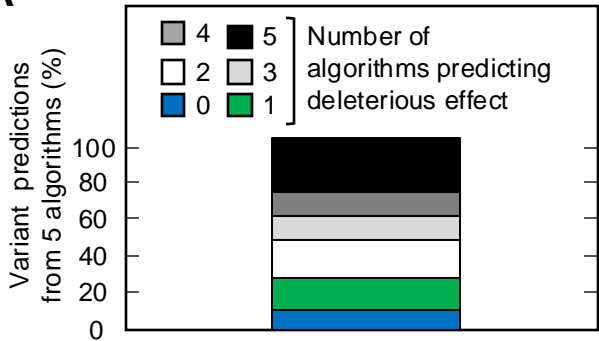

B

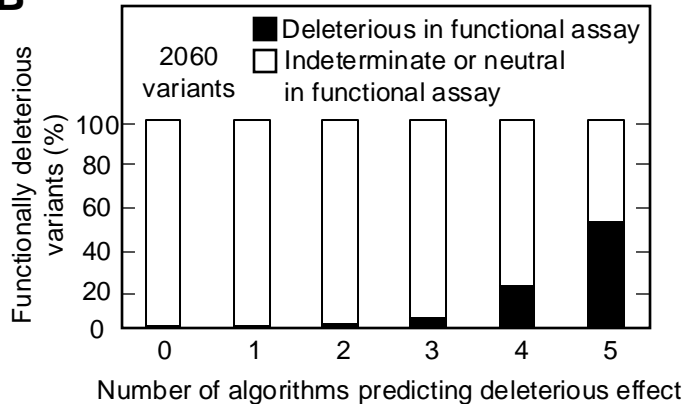

C

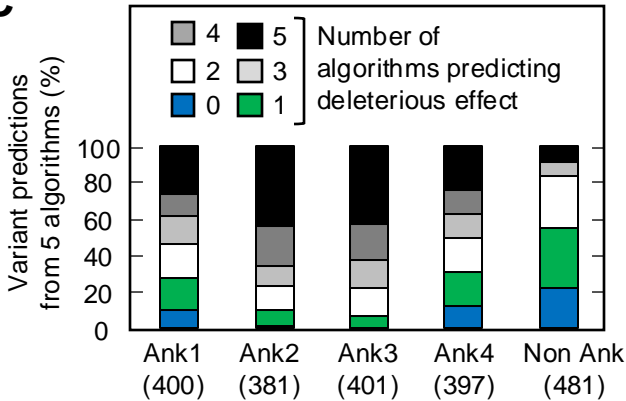

D

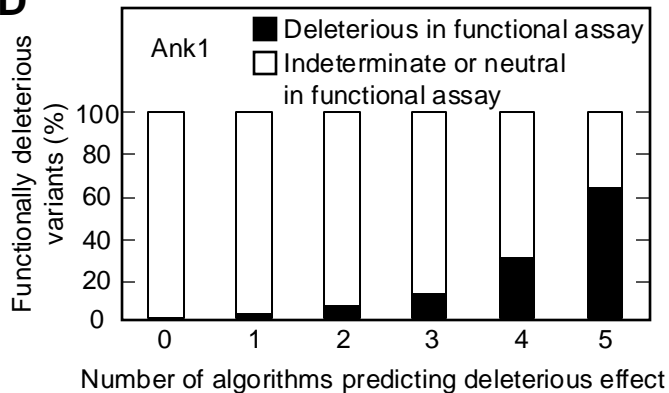

E

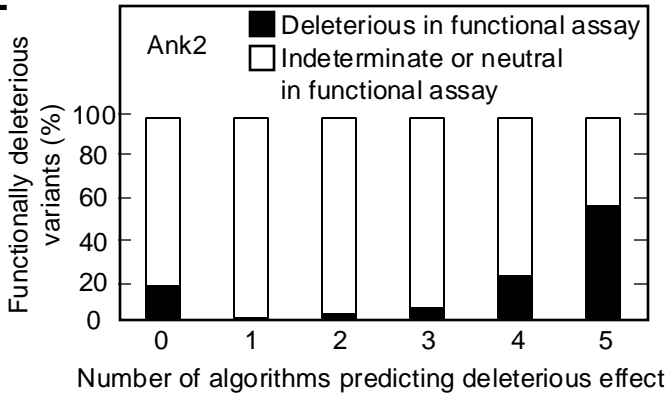

F

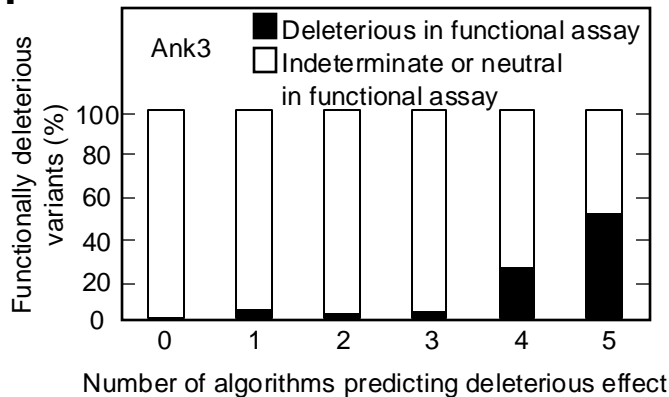

G

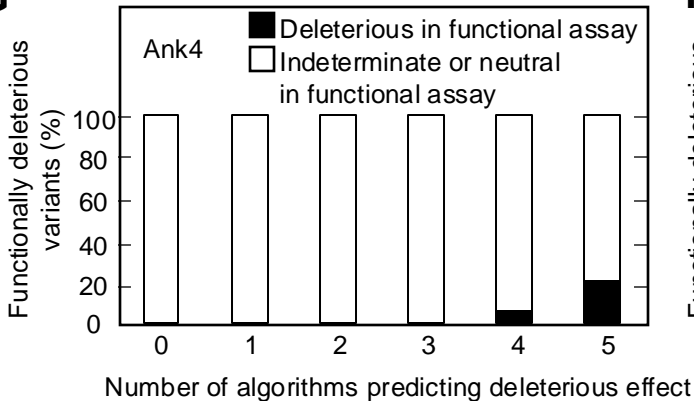

H

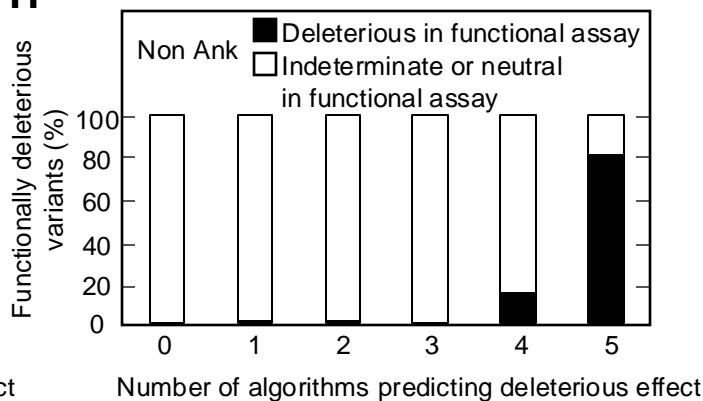

Supplement: Supplement 22 [file media-22.pdf]

Figure 4-figure supplement 1

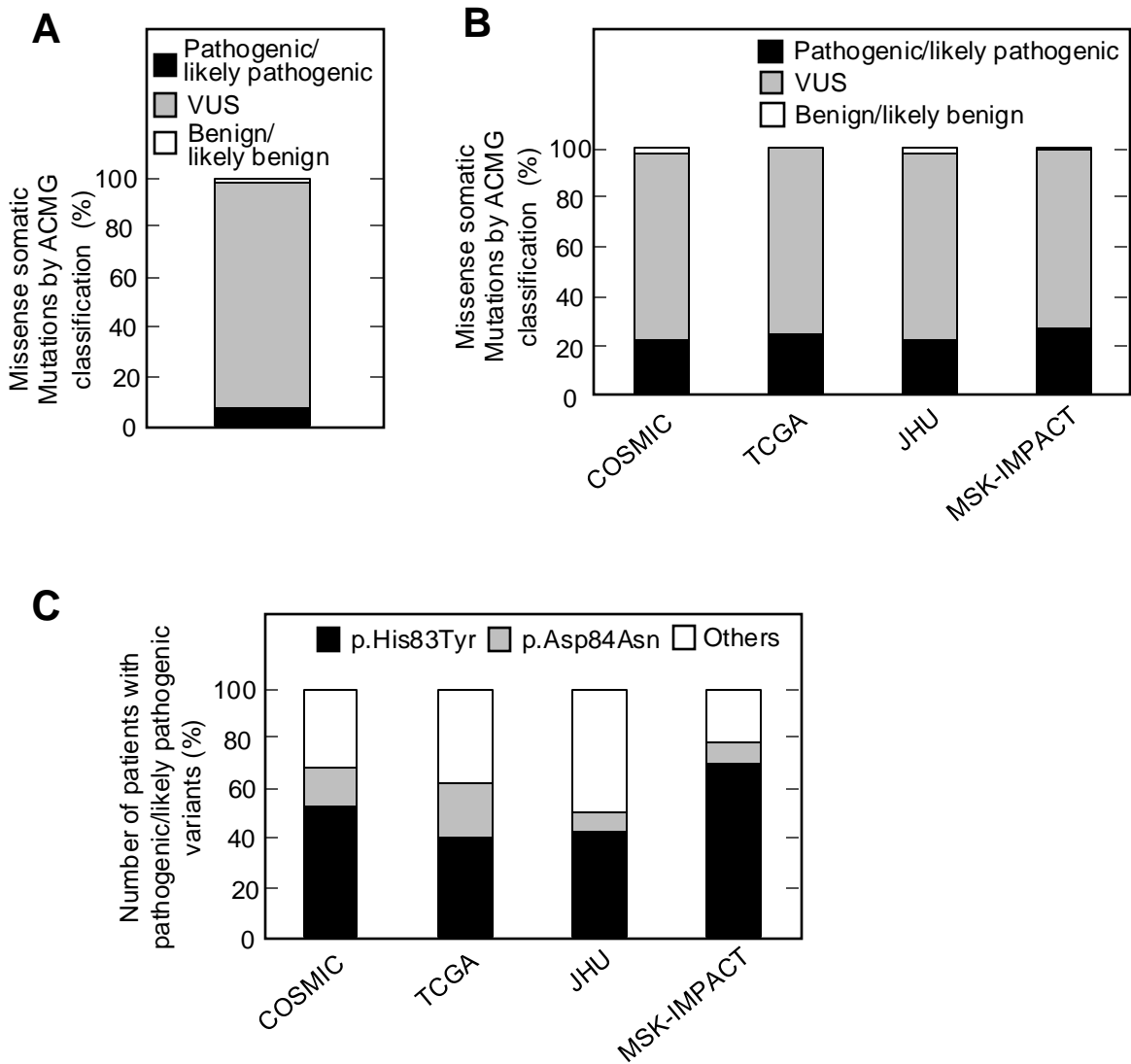

Supplement: Supplement 23 [file media-23.pdf]

Figure 4-figure supplement 2

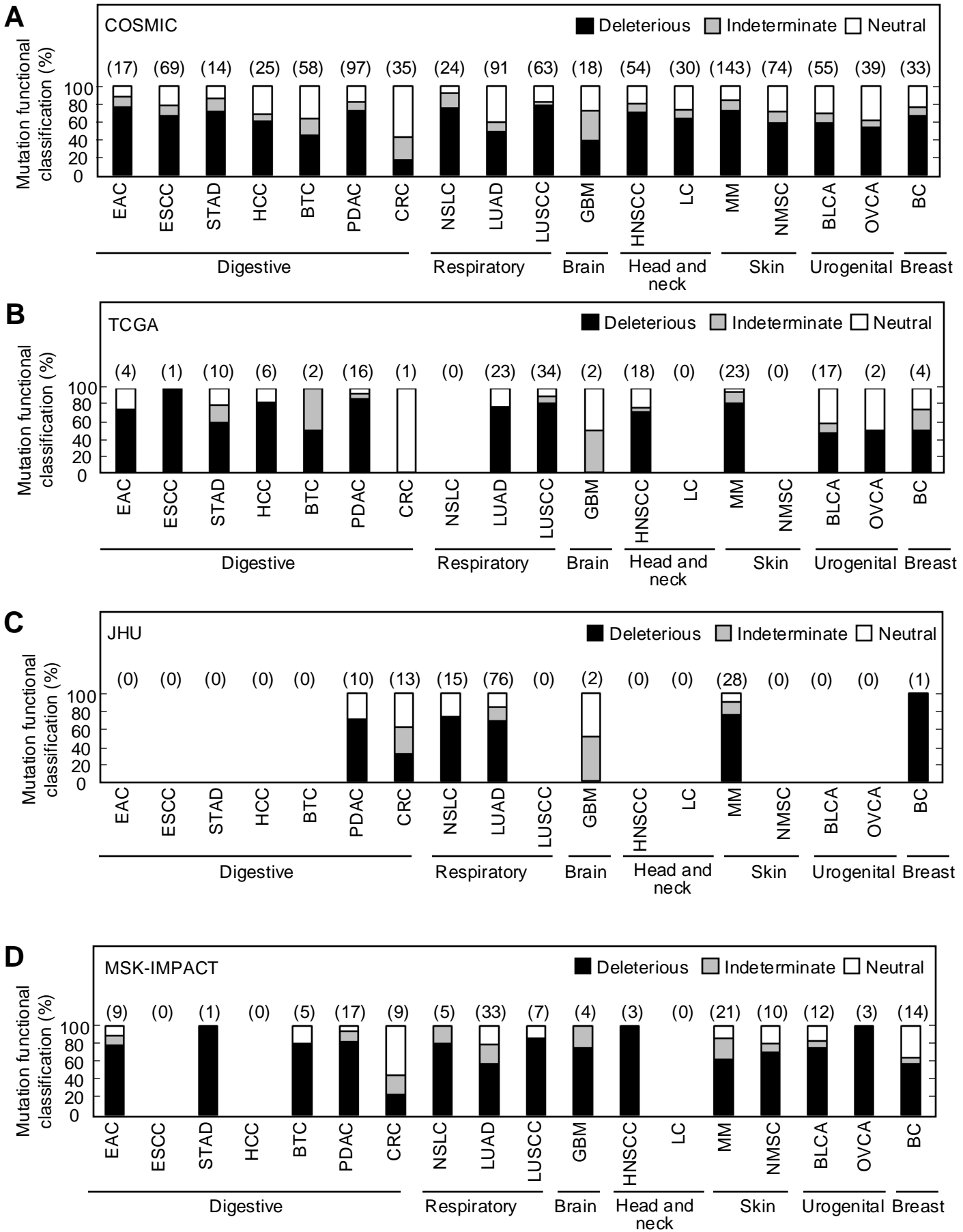

Supplement: Supplement 24 [file media-24.pdf]
