# Supplementary material for: Functional characterization of all CDKN2A missense variants and comparison to in silico models of pathogenicity
Source: bioRxiv. 2025 Feb 11:2023.12.28.573507. Originally published 2023 Dec 28. Preprint. [Version 3] doi: 10.1101/2023.12.28.573507 (PMC10793438; doi:10.1101/2023.12.28.573507)
Supplement: Supplement 25 [file NIHPP2023.12.28.573507v3-supplement-25.pdf]

bioRxiv preprint doi: https://doi.org/10.1101/2023.12.28.573507; this version posted February 11, 2025. The copyright holder for this preprint (which was not certified by peer review) is the author/funder, who has granted bioRxiv a license to display the preprint in perpetuity. It is made available under aCC-BY 4.0 International license.

Supplementary Information

Figures

Figure 1-figure supplement 1. Development and validation of high-throughput CDKN2A functional assay.

Figure 1-figure supplement 2. Data for CDKN2A plasmid library

Figure 2-figure supplement 1. P values for all possible CDKN2A missense variants.

Figure 2-figure supplement 2. Normalized fold change for all possible CDKN2A missense variants.

Figure 2-figure supplement 3. Reproducibility of CDKN2A assay

Figure 2-figure supplement 4. Proportion of variants Day 9

Figure 2-figure supplement 5. Functional characterization of all possible *CDKN2A* missense variants by ankyrin domain and residue.

Figure 3-figure supplement 1. Variant in silico predictions for 7 algorithms

Figure 3-figure supplement 2. Variant in silico predictions for 5 algorithms

Figure 4-figure supplement 1. Missense somatic mutations in *CDKN2A*.

Figure 4-figure supplement 2. Functional classification of missense somatic mutations in *CDKN2A*.

Tables

Appendix 1-table 1. Assay outputs for CellTag experiments.

Appendix 1-table 2. Proportion of each variant in the initial plasmid library.

Appendix 1-table 3. Proportion of each variant in residues R24, H66, and A127.

Appendix 1-table 4. Assay outputs and functional classifications for all possible *CDKN2A* missense and synonymous variants.

Appendix 1-table 5. Day of confluency by experiment and residue.

Appendix 1-table 6. Normalized fold change for all possible *CDKN2A* missense and synonymous variants.

Appendix 1-table 7. In silico variant effect predictions for *CDKN2A* missense variants.

Appendix 1-table 8. Assessment of in silico variant effect prediction models.

Appendix 1-table 9. Missense somatic mutations in *CDKN2A* reported in COSMIC, TCGA, JHU, MSK-IMPACT.

Appendix 1-table 10. *CDKN2A* missense and synonymous variants reported in gnomAD.

Appendix 1-table 11. *CDKN2A* missense VUSs reported in ClinVar.

Appendix 1-table 12. Codon optimized *CDKN2A* sequence.

Appendix 1-table 13. Sequences of primers used in study.

bioRxiv preprint doi: https://doi.org/10.1101/2023.12.28.573507; this version posted February 11, 2025. The copyright holder for this preprint (which was not certified by peer review) is the author/funder, who has granted bioRxiv a license to display the preprint in perpetuity. It is made available under aCC-BY 4.0 International license.

Source data

Figure 1-source data

Raw data in Figure 1

Figure 1-figure supplement 1-source data 1

Raw data in Figure 1-figure supplement 1A

Figure 1-figure supplement 1-source data 2

Raw data in Figure 1-figure supplement 1B

Figure 2-source data 1

Raw data in Figure 2B

Figure 2-figure supplement 1-source data 1

Raw data in Figure 2-figure supplement 1A and 1B

Figure 2-figure supplement 2-source data 1

Raw data in Figure 2-figure supplement 2D

Figure 2-figure supplement 3-source data 1

Raw data in Figure 2-figure supplement 3A

Figure 2-figure supplement 3-source data 2

Raw data in Figure 2-figure supplement 3B

Figure 2-figure supplement 4-source data 1

Raw data in Figure 2-figure supplement 4

Figure 2-figure supplement 5-source data 1

Raw data in Figure 2-figure supplement 5B

Figure 2-figure supplement 5-source data 2

Raw data in Figure 2-figure supplement 5C

Figure 3-source data 1

Raw data in Figure 3

Figure 3-figure supplement 1-source data 1

Raw data in Figure 3-figure supplement 1A and 1B

Figure 3-figure supplement 1-source data 2

Raw data in Figure 3-figure supplement 1C

Figure 3-figure supplement 1-source data 3

Raw data in Figure 3-figure supplement 1D - 1H

Figure 3-figure supplement 2-source data 1

Raw data in Figure 3-figure supplement 2A and 2B

Figure 3-figure supplement 2-source data 2

Raw data in Figure 3-figure supplement 2C

Figure 3-figure supplement 2-source data 3

bioRxiv preprint doi: https://doi.org/10.1101/2023.12.28.573507; this version posted February 11, 2025. The copyright holder for this preprint (which was not certified by peer review) is the author/funder, who has granted bioRxiv a license to display the preprint in perpetuity. It is made available under aCC-BY 4.0 International license.

Raw data in Figure 3-figure supplement 2D - 2H

Figure 4-source data 1

Raw data in Figure 4A and 4B

Figure 4-source data 2

Raw data in Figure 4C

Figure 4-figure supplement 1-source data 1

Raw data in Figure 4-figure supplement 1A and 1B

Figure 4-figure supplement 1-source data 2

Raw data in Figure 4-figure supplement 1C

Figure 4-figure supplement 2-source data 1

Raw data in Figure 4-figure supplement 2A - 2D

bioRxiv preprint doi: https://doi.org/10.1101/2023.12.28.573507; this version posted February 11, 2025. The copyright holder for this preprint (which was not certified by peer review) is the author/funder, who has granted bioRxiv a license to display the preprint in perpetuity. It is made available under aCC-BY 4.0 International license.

Figure -figure supplement legend

Figure 1-figure supplement 1. **Development and validation of high-throughput CDKN2A functional assay.** (**A**) Cell proliferation of PANC-1 cells stably expressing empty expression vector, codon optimized CDKN2A, one of three synonymous variants (p.L32L, p.G101G, p.V126V), or one of three pathogenic variants (p.L32P, p.G101W, p.V126D) over 14 days in culture. Cell proliferation values are given as mean of three repeats ± standard deviation normalized to PANC-1 cells that stably express empty vector. Statistically significant inhibition of cell proliferation inhibition in PANC-1 cells that stably express synonymous variants (*; P value < 0.001). (**B**) PANC-1 cells stably expressing codon optimized CDKN2A transduced with a CellTag lentiviral library of 20 nonfunctional barcodes were cultured and representation (percent of reads supporting each barcode) before (Day 9) and after a period of in vitro cell proliferation (Day 45) was determined using next generation sequencing. Percent values are given as the mean of three repeats ± standard deviation.

Figure 1-figure supplement 2. Data for CDKN2A plasmid library. (**A**) Dot plot showing proportion of each variant per residue in the plasmid libraries. (**B**) Variant proportion in plasmid libraries grouped in 0.5% increments. (**C**) Dot plot showing variant proportion in the amplified plasmid library compared to the Day 9 cell pool. (**D**) Normalized fold change of variant proportion between Day 9 cell pool and the amplified plasmid library based on ACMG classification.

Figure 2-figure supplement 1. P values for all possible *CDKN2A* missense variants. (**A**) Distribution of $\log_2$ P values for all possible *CDKN2A* missense variants. (**B**) Distribution of $\log_2$ P values for benchmark pathogenic variants (red box), benchmark benign variants (blue box), VUSs previously reported to have functionally deleterious effects (orange box), and VUSs previously reported to have functionally neutral effects (green box). (**C**) Dot plot showing $\log_2$ P value of all possible *CDKN2A* missense variants pre residue.

Figure 2-figure supplement 2. Normalized fold change for all possible *CDKN2A* missense variants. (**A**) Dot plot showing $\log_2$ normalized fold change of all possible *CDKN2A* missense variants by residue (**B**) Log_2 normalized fold change for 32 benchmark pathogenic variants, 6 benign variants, 31 VUSs previously reported to have functionally deleterious effects, and 18 VUSs previously reported to have functionally neutral effects. (**C**) Functional classifications for 3,120 *CDKN2A* variants, including 2,964 missense variants and 156 synonymous variants. Variants were classified as functionally deleterious, indeterminate function, or neutral based on $\log_2$ normalized fold change. (**D**) Comparison of functional classification of all possible *CDKN2A* missense variants by $\log_2$ P value (gamma GLM) and log normalized fold change.

Figure 2-figure supplement 3. Reproducibility of CDKN2A assay. (**A**) Dot plot showing $\log_2$ P value for 560 *CDKN2A* missense variants assayed in duplicate. (**B**) Comparison of functional classifications for 560 *CDKN2A* missense variants assayed in duplicate. (**C**) Dot plot showing $\log_2$ normalized fold change for 560 *CDKN2A* missense variants assayed in duplicate.

Figure 2-figure supplement 4. Proportion of variants Day 9. (**A**) Proportion of all possible 2,964 *CDKN2A* missense variants in the Day 9 cell pool (replicate 1 if duplicated). (**B**) Percent of functionally deleterious variants (black box), variants of indeterminate function, and functionally neutral variants (white box) by variant proportion in the Day 9 cell pool (replicate 1 if duplicated). Left graph variants grouped as < 2% and ≥ 2% in Day 9 Cell Pool. Right graph, variants grouped as < 2%, 1% intervals from 2% to 8%, ≥ 8% in the Day 9 cell pool.

bioRxiv preprint doi: https://doi.org/10.1101/2023.12.28.573507; this version posted February 11, 2025. The copyright holder for this preprint (which was not certified by peer review) is the author/funder, who has granted bioRxiv a license to display the preprint in perpetuity. It is made available under aCC-BY 4.0 International license.

Figure 2-figure supplement 5. Functional characterization of all possible *CDKN2A* missense variants by ankyrin domain and residue. (**A**) Schematic representation of CDKN2A with ankyrin repeats 1-4 represented. (**B**) Percent of functionally deleterious (black box), indeterminate function (gray box), and functionally neutral variants (white box) within ankyrin repeats and non-ankyrin repeat regions of CDKN2A. Ank; Ankyrin repeat. (**C**) Dot plot showing distribution of percent functionally deleterious missense variants per residue.

Figure 3-figure supplement 1. Combinational prediction for 7 algorithms. (**A**) Number of algorithms predicting deleterious effect for 904 *CDKN2A* missense variants with predictions from 7 algorithms. (**B**) Percent of functionally deleterious (black box) and indeterminate function or functionally neutral (white box) variants grouped by the number of algorithms predicting deleterious effect. (**C**) Number of algorithms predicting deleterious effect for 904 *CDKN2A* missense variants grouped by ankyrin repeats and non-ankyrin repeat regions. (**D** - **H**) Percent of functionally deleterious (black box) and indeterminate function or functionally neutral (white box) variants grouped by the number of algorithms predicting deleterious effect in Ank1 (**D**), Ank2 (**E**), Ank3 (**F**), Ank4 (**G**), and non-ankyrins repeat regions (**H**) of CDKN2A.

Figure 3-figure supplement 2. Combinational prediction for 5 algorithms. (**A**) Number of algorithms predicting deleterious effect for 2,060 *CDKN2A* missense variants with predictions from 5 algorithms. (**B**) Percent of functionally deleterious (black box) and indeterminate function or functionally neutral (white box) variants grouped by the number of algorithms predicting deleterious effect. (**C**) Number of algorithms predicting deleterious effect for 2,060 *CDKN2A* missense variants grouped by ankyrin repeats and non-ankyrin repeat regions. (**D** - **H**) Percent of functionally deleterious (black box) and indeterminate function or functionally neutral (white box) variants grouped by the number of algorithms predicting deleterious effect in Ank1 (**D**), Ank2 (**E**), Ank3 (**F**), Ank4 (**G**), and non-ankyrins repeat regions (**H**) of CDKN2A.

Figure 4-figure supplement 1. Missense somatic mutations in *CDKN2A*. (**A**) Percent of missense somatic mutations in *CDKN2A* reported in either COSMIC, TCGA, JHU, or MSK-IMPACT that were classified as pathogenic or likely pathogenic (black box), VUS (gray box), or benign or likely benign (white box) using ACMG interpretation guidelines. (**B**) Percent of missense somatic mutations in *CDKN2A* that were classified as pathogenic or likely pathogenic (black box), VUS (gray box), or benign or likely benign (white box) using ACMG interpretation guidelines grouped by mutation database. (**C**) Number of patients with a pathogenic or likely pathogenic missense somatic mutation grouped by mutation database. Patients with p.His83Tyr mutation (black box), patients with p.Asp84Asn mutations (grep box), and patients with other mutations highlighted. COSMIC; the Catalogue Of Somatic Mutations In Cancer, TCGA; The Cancer Genome Atlas, JHU; The Johns Hopkins University School of Medicine, MSK-IMPACT; Memorial Sloan Kettering-Integrated Mutation Profiling of Actionable Cancer Targets.

Figure 4-figure supplement 2. Functional classification of missense somatic mutations in *CDKN2A*. Percent of missense somatic mutations in *CDKN2A* reported in either COSMIC (A), TCGA (B), JHU (C), or MSK-IMPACT (D) that were classified as functionally deleterious (black box), indeterminate (gray box), or functionally neutral (white box) in our CDKN2A functional assay grouped by tumor type. The number of missense somatic mutations for each tumor type given in parentheses. COSMIC; the Catalogue Of Somatic Mutations In Cancer, TCGA; The Cancer Genome Atlas, JHU; The Johns Hopkins University School of Medicine, MSK-IMPACT; Memorial Sloan Kettering-Integrated Mutation Profiling of Actionable Cancer Targets.
