# Supplementary material for: Functional characterization of all CDKN2A missense variants and comparison to in silico models of pathogenicity
Source: bioRxiv. 2024 Sep 22:2023.12.28.573507. Originally published 2023 Dec 28. Preprint. [Version 2] doi: 10.1101/2023.12.28.573507 (PMC10793438; doi:10.1101/2023.12.28.573507)

Figure 2-figure supplement 2

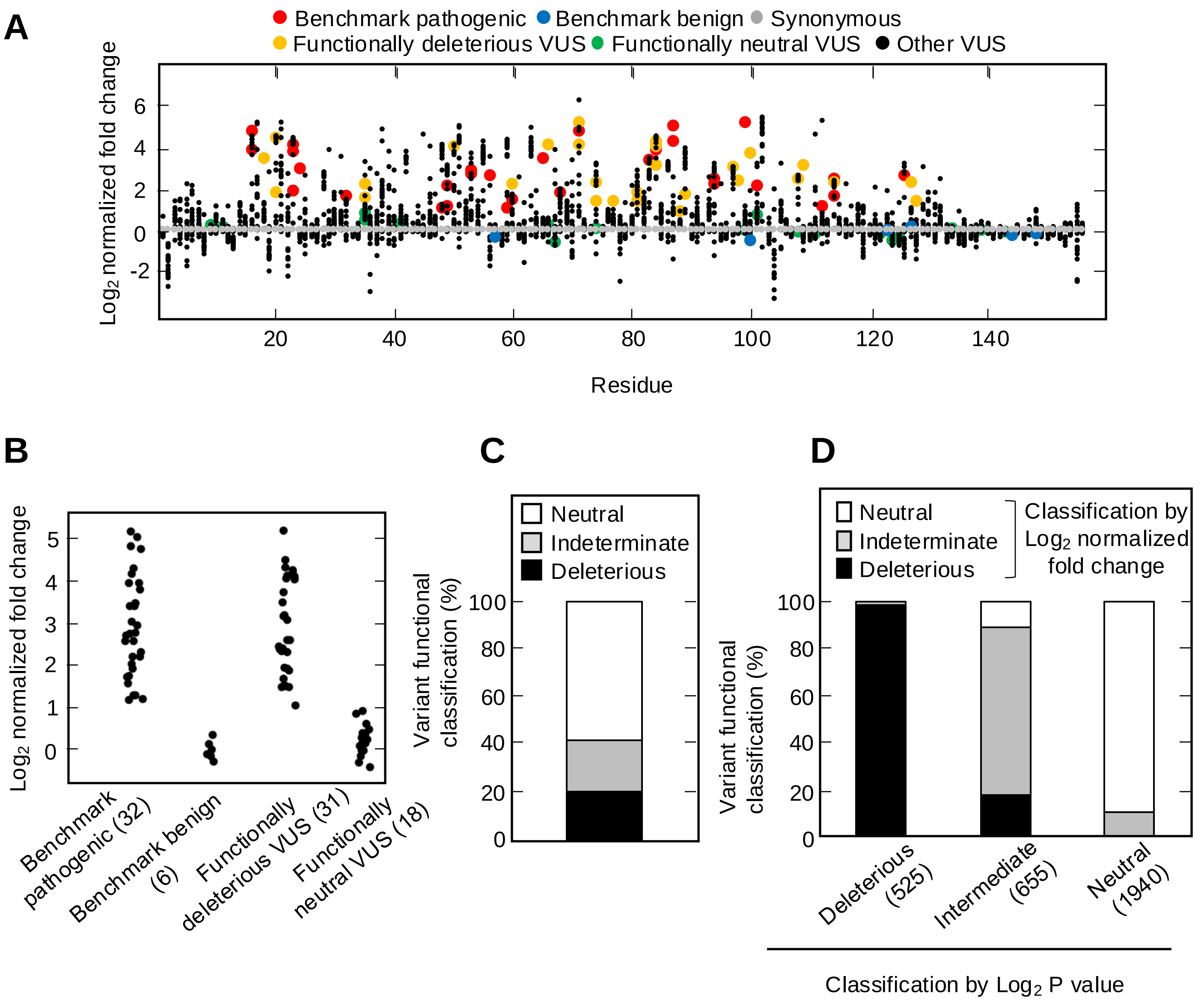

Supplement: Supplementary file 1 [file media-17.pdf]
